# Supplementary material for: Enantioselective synthesis of [4]helicenes by organocatalyzed intermolecular C-H amination
Source: Nat Commun. 2024 Jan 25;15:732. doi: 10.1038/s41467-024-45049-w (PMC10810882; doi:10.1038/s41467-024-45049-w)
Supplement: Supplementary file 1 — Supplementary Information [file 41467_2024_45049_MOESM1_ESM.pdf]

Supplementary Information

Enantioselective Synthesis of [4]Helicenes by Organocatalyzed Intermolecular C-H Amination

Xihong Liu[1]*, Boyan Zhu[1], Xiaoyong Zhang[2], Hanwen Zhu[1], Jingying Zhang[1], Anqi Chu[1], Fujun Wang, and Rui Wang[1]*

[1]*Key Laboratory of Preclinical Study for New Drugs of Gansu Province, School of Basic Medical Sciences & Research Unit of Peptide Science, Chinese Academy of Medical Sciences, 2019RU066, Lanzhou University, Lanzhou 730000, P. R. China*
[2]*Institute of Systems and Physical Biology, Shenzhen Bay Laboratory, Shenzhen 518055, P. R. China*

*Corresponding authors: liuxihong@lzu.edu.cn; wangrui@lzu.edu.cn

Table of Contents

1 Supplementary Methods……………………………………………………………………S1
1.1 General information………………………………………………………………...S1
1.2 Substrates and catalyst synthesis…………………………………………………...S1
1.3 General procedure for enantioselective synthesis of 1,12-disubstituted [4]helicenes **3**…….S5
1.4 General procedure for methylation of chiral 1,12-disubstituted [4]helicenes **3**…………..…S6
1.5 Characterization data of helically chiral products **3** and **4**…………………………...S8
1.6 Unsuccessful examples……………………………………………………………… S23
2 Supplementary Discussion…………………………………………………………………S24
2.1 Scale-up experiment of **3a** and **3ab**……………………..…………………………....S24
2.2 Synthetic transformations of **3a, 4a** and **4ab**………………………………………….S24
2.3 DFT calculations on the enantiomerization process of **1a**, **1j** and **3a**………………………S27
2.4 DFT calculations on the rotation barriers of the newly formed C-N bond in **3a** and **4a**…….S28
2.5 The mechanistic studies……………………………………………………….……...S28
2.6 Determination of the enantiomerization barrier of **6**…………………………………….S33
2.7 Representative CD spectra…………………………………………………………...S33
2.8 X-Ray structures of **4ad** and **6**………………………………………….......................S34
2.9 HPLC of racemic and enantioenriched products **3** and **4**……………………………...S37
2.10 NMR spectra………………………………………………………………...……..…S117
3 Supplementary References………………………………………………………………S238

1 Supplementary Methods

1.1 General Information

Unless stated otherwise, all reactions were carried out in flame dried glassware. All solvents were dried according to established procedures. Chemicals were purchased from commercial suppliers and used without further purification. Reactions were monitored by thin layer chromatography (TLC), column chromatography purifications were carried out using silica gel. NMR spectra were recorded on a Bruker 300MHz or 600 MHz instrument and internally referenced to tetramethylsilane signal or residual protic solvent signals. Data for ^{1}H NMR are recorded as follows: chemical shift, integration, multiplicity (br = broad, s = singlet, d = doublet, t = triplet, q = quartet, m = multiplet cm = complex multiplet) and coupling constant in Hertz (Hz). Data for ^{13}C NMR are reported in terms of chemical shift (δ, ppm). Optical rotations were reported as follows: $[\alpha]_D^{rt}$ (c: g/100mL, in $CHCl_3$). Melting points were measured on a SCW X-4 and values are uncorrected. High resolution mass spectra (HRMS) were obtained by the ESI ionization sources. The ee values determination was carried out using chiral high-performance liquid chromatography (HPLC) with Daicel Chiracel column on Waters with a 996 UV-detector. Diazodicarboxamides **2** were prepared according to literature methods, and their characterization data match the reported data.[1,2]

1.2 Substrates and catalyst synthesis

1.2.1 Synthesis of polycyclic phenols 1[3]

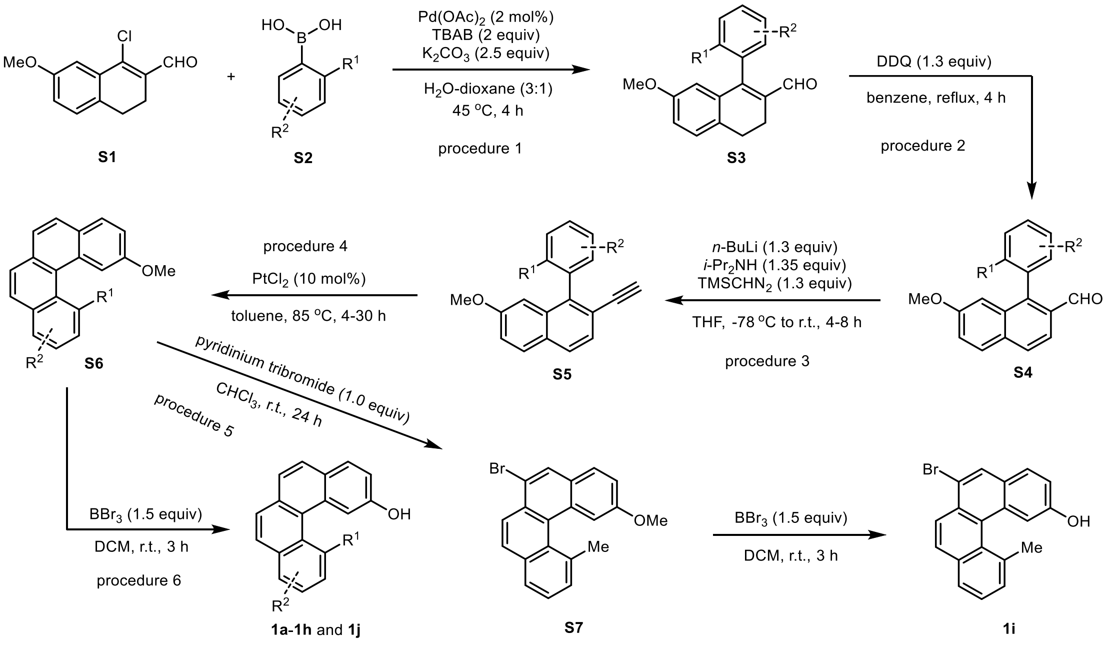

Supplementary Fig. 1. General procedures for the synthesis of polycyclic phenols **1a-1j**.

Procedure 1: A mixture of **S1** (15 mmol, 1.0 equiv), *ortho*-substituted phenylboric acid **S2** (16.5 mmol, 1.1 equiv), tetrabutylammonium bromide (30 mmol, 2.0 equiv), palladium acetate (0.3 mmol, 0.02 equiv) and potassium carbonate (37.5 mmol, 2.5 equiv) in water (30 mL) and 1,4-dioxane (10 mL) was stirred for 4 h under argon atmosphere at 45 °C. After the complete consumption of **S1**, the reaction mixture was diluted with water and extracted with EtOAc for three times. The combined

organic layers were dried over anhydrous Na_2SO_4, filtered, and concentrated *in vacuo*. The crude product was purified by silica column chromatography to afford **S3**.

Procedure 2: A suspension of **S3** (1.0 equiv) and DDQ (1.3 equiv) in benzene (0.2 M) was stirred under reflux for 4 h. Then the reaction mixture was filtered through a pad of celite and the solvent was evaporated. The residue was dissolved in EtOAc and washed with 1 M NaOH. The organic layer was dried and concentrated *in vacuo*. The crude product was purified by silica column chromatography to afford **S4**.

Procedure 3: To a solution of diisopropylamine (1.35 equiv) in THF was added *n*-BuLi (1.3 equiv) at 0 °C under argon atmosphere and the resulting mixture was stirred for 10 min. Then the reaction mixture was cooled to -78 °C and $TMSCHN_2$ (1.3 equiv) was added dropwise. After stirring for 30 min, a solution of **S4** (1.0 equiv) in THF was further added dropwise and the reaction system was then stirred at room temperature for 4-8 h. The reaction was quenched with water and the aqueous layer was extracted with EtOAc for three times. The combined organic layers were washed with brine, dried over anhydrous Na_2SO_4, and concentrated *in vacuo*. The crude product was purified by silica column chromatography to afford **S5**.

Procedure 4: A mixture of **S5** (1.0 equiv) and $PtCl_2$ (0.1 equiv) was dissolved in toluene (0.1 M) and stirred for 4-30 h at 80 °C under argon atmosphere. After the complete consumption of **S5**, the reaction mixture was filtered through a short plug of a silica gel and the filtrate was concentrated under reduced pressure. The resulting residue was purified by silica column chromatography to afford **S6**.

Procedure 5: To a solution of 11-methoxy-1-methylbenzo[*c*]phenanthrene (1.0 equiv) in $CHCl_3$ (0.2 M) was added pyridinium tribromide (1.0 equiv) and the resulting reaction mixture was stirred at room temperature for 24 h. Then 10% $Na_2S_2O_3$ was added and the aqueous layer was extracted with $CHCl_3$ for three times. The combined organic layers were washed with brine, dried over anhydrous Na_2SO_4, and concentrated under reduced pressure. The crude product was purified by silica column chromatography to afford **S7**.

Procedure 6: To a solution of **S6** or **S7** (1.0 equiv) in CH_2Cl_2 was added BBr_3 (1.5 equiv) at 0 °C. The mixture was stirred at room temperature until the complete consumption of the starting material. Then the reaction mixture was cooled to 0 °C and quenched with sat. $NaHCO_3$. The aqueous layer was extracted with CH_2Cl_2 for three times. The combined organic layers were washed with brine, dried over anhydrous Na_2SO_4, and concentrated under reduced pressure. The crude product was purified by silica column chromatography to afford the corresponding polycyclic phenol **1**.

1.2.2 Characterization data of substrates 1

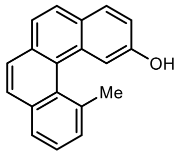

1a

White solid, 36% overall yield; **^{1}H NMR** (300 MHz, $CDCl_3$) δ 7.85 (d, *J* = 8.6 Hz, 2H), 7.83 – 7.75 (m, 2H), 7.68 (d, *J* = 8.5 Hz, 2H), 7.53 (t, *J* = 7.4 Hz, 1H), 7.45 (d, *J* = 7.1 Hz, 1H), 7.38 (s, 1H), 7.12 (dd, *J* = 8.7, 2.3 Hz, 1H), 5.18 (s, 1H), 2.40 (s, 3H) ppm; **^{13}C NMR** (75 MHz, $CDCl_3$) δ 153.28, 135.26, 133.89, 132.32, 131.72, 129.73, 129.71, 129.32, 127.87, 127.35, 127.15, 126.10, 125.91, 125.73, 125.51, 123.82, 116.07, 113.37, 24.80 ppm; **HRMS** (ESI): $C_{19}H_{14}NaO$ $[M+Na]^+$ calcd: 281.0937, found: 281.0930.

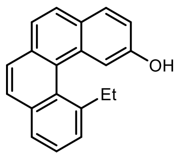

1b

White solid, 33% overall yield; **^{1}H NMR** (300 MHz, $CDCl_3$) δ 7.85 (d, *J* = 8.7 Hz, 2H), 7.78 (d, *J* = 6.9 Hz, 2H), 7.68 (d, *J* = 8.5 Hz, 2H), 7.58 (d, *J* = 4.7 Hz, 2H), 7.54 (s, 1H), 7.12 (d, *J* = 8.7 Hz, 1H), 5.06 (s, 1H), 3.08 (dq, *J* = 14.8, 7.5 Hz, 1H), 2.80 (dq, *J* = 14.7, 7.4 Hz, 1H), 0.81 (t, *J* = 7.4 Hz, 3H) ppm; **^{13}C NMR** (75 MHz, $CDCl_3$) δ 152.12, 141.09, 132.68, 131.10, 130.95, 128.24, 127.41, 126.74, 126.26, 126.16, 125.95, 124.99, 124.89, 124.84, 124.42, 122.78, 115.00, 111.78, 28.82, 15.39 ppm; **HRMS** (ESI): $C_{20}H_{16}NaO$ $[M+Na]^+$ calcd: 295.1093, found: 295.1085.

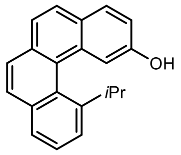

1c

White solid, 40% overall yield; **^{1}H NMR** (300 MHz, $CDCl_3$) δ 7.88 – 7.79 (m, 2H), 7.75 (d, *J* = 4.1 Hz, 2H), 7.67 (ddd, *J* = 9.4, 4.9, 2.5 Hz, 4H), 7.60 (t, *J* = 7.3 Hz, 1H), 7.09 (dd, *J* = 8.8, 2.0 Hz, 1H), 4.92 (s, 1H), 3.65 (p, *J* = 6.8 Hz, 1H), 1.66 (d, *J* = 6.8 Hz, 3H), 0.46 (d, *J* = 6.7 Hz, 3H) ppm; **^{13}C NMR** (75 MHz, $CDCl_3$) δ 153.10, 147.64, 134.01, 132.46, 132.11, 129.26, 127.81, 127.59, 127.07, 126.84, 126.46, 126.02, 125.86, 125.55, 124.07, 124.03, 116.19, 112.71, 32.50, 28.21, 21.04 ppm; **HRMS** (ESI): $C_{21}H_{19}O$ $[M+H]^+$ calcd: 287.1430, found: 287.1423.

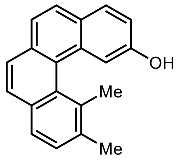

1d

White solid, 28% overall yield; **^{1}H NMR** (300 MHz, $CDCl_3$) δ 7.81 (d, *J* = 8.5 Hz, 2H), 7.76 – 7.56 (m, 4H), 7.41 (d, *J* = 8.0 Hz, 2H), 7.07 (d, *J* = 9.0 Hz, 1H), 5.21 (s, 1H), 2.47 (s, 3H), 2.24 (s, 3H) ppm; **^{13}C NMR** (75 MHz, $CDCl_3$) δ 153.21, 136.28, 133.21, 132.52, 132.32, 132.16, 129.98, 129.35, 128.61, 127.68, 127.20, 126.96, 125.91, 125.22, 125.19, 123.95, 115.92, 113.04, 22.67, 20.90 ppm; **HRMS** (ESI): $C_{20}H_{17}O$ $[M+H]^+$ calcd: 273.1274, found: 273.1267.

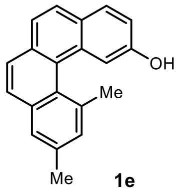

1e

White solid, 33% overall yield; **^{1}H NMR** (300 MHz, $CDCl_3$) δ 7.84 (t, *J* = 7.9 Hz, 2H), 7.79 – 7.62 (m, 3H), 7.59 (s, 1H), 7.39 (s, 1H), 7.31 (s, 1H), 7.18 – 7.07 (m, 1H), 5.10 (s, 1H), 2.55 (s, 3H), 2.39 (s, 3H) ppm; **^{13}C NMR** (75 MHz, $CDCl_3$) δ 153.71, 135.92, 135.62, 134.68, 132.42, 132.20, 132.14, 129.77, 128.43, 128.08, 127.91, 127.23, 126.76, 126.48, 125.64, 124.41, 116.51, 113.97, 25.14, 21.79 ppm; **HRMS** (ESI): $C_{20}H_{17}O$ $[M+H]^+$ calcd: 273.1274, found: 273.1265.

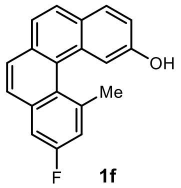

1f

White solid, 15% overall yield; **^{1}H NMR** (300 MHz, $CDCl_3$) δ 7.85 (dd, *J* = 8.6, 5.1 Hz, 2H), 7.78 – 7.60 (m, 3H), 7.43 (d, *J* = 7.9 Hz, 1H), 7.34 (s, 1H), 7.22 (d, *J* = 6.2 Hz, 1H), 7.14 (d, *J* = 8.3 Hz, 1H), 5.31 (s, 1H), 2.39 (s, 3H) ppm; **^{13}C NMR** (75 MHz, $CDCl_3$) δ 153.36, 138.26 (J_{C-F} = 7.9 Hz), 134.94 (J_{C-F} = 9.0 Hz), 131.75, 131.48, 129.39, 127.49, 127.28, 127.23, 127.15, 127.01, 126.67 (J_{C-F} = 1.6 Hz), 126.23, 123.75, 118.14 (J_{C-F} = 23.0 Hz), 116.33, 113.26, 109.38 (J_{C-F} = 20.3 Hz), 24.90 (J_{C-F} = 1.8 Hz) ppm; **^{19}F NMR** (282 MHz, $CDCl_3$) δ -116.84 ppm; **HRMS** (ESI): $C_{19}H_{13}FNaO$ $[M+Na]^+$ calcd: 299.0843, found: 299.0829.

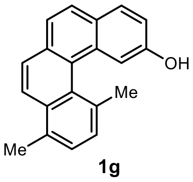

1g

White solid, 22% overall yield; **^{1}H NMR** (300 MHz, $CDCl_3$) δ 8.02 (d, *J* = 8.6 Hz, 1H), 7.88 (d, *J* = 3.5 Hz, 1H), 7.85 (d, *J* = 3.4 Hz, 1H), 7.80 – 7.64 (m, 2H), 7.48 – 7.31 (m, 3H), 7.13 (d, *J* = 8.7 Hz, 1H), 5.02 (s, 1H), 2.79 (s, 3H), 2.37 (s, 3H) ppm; **^{13}C NMR** (75 MHz, $CDCl_3$) δ 153.73, 142.71, 134.31, 132.72, 132.57, 129.87, 129.04, 128.37, 127.89, 127.80, 127.57, 126.62, 126.52, 126.05, 124.41, 116.62, 113.41, 30.45, 17.01 ppm; **HRMS** (ESI): $C_{20}H_{16}NaO$ $[M+Na]^+$ calcd: 295.1093, found: 295.1086.

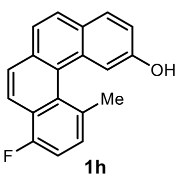

1h

White solid, 9% overall yield; **^{1}H NMR** (300 MHz, $CDCl_3$) δ 8.10 (d, *J* = 8.6 Hz, 1H), 7.85 (dd, *J* = 8.0, 3.2 Hz, 2H), 7.70 (dd, *J* = 18.1, 8.6 Hz, 2H), 7.34 (d, *J* = 6.1 Hz, 2H), 7.26 – 7.19 (m, 1H), 7.15 (t, *J* = 8.9 Hz, 1H), 5.42 (s, 1H), 2.33 (s, 3H) ppm; **^{13}C NMR** (75 MHz, $CDCl_3$) δ 153.42, 132.61, 131.51, 130.97 ($J_{C\text{-}F}$ = 3.3 Hz), 130.73 ($J_{C\text{-}F}$ = 3.7 Hz), 129.57, 129.46, 129.35, 127.69, 127.52, 126.46 ($J_{C\text{-}F}$ = 2.0 Hz), 125.81 ($J_{C\text{-}F}$ = 2.3 Hz), 123.72, 123.11 ($J_{C\text{-}F}$ = 15.0 Hz), 119.58 ($J_{C\text{-}F}$ = 7.4 Hz), 116.33, 113.51, 110.17 ($J_{C\text{-}F}$ = 20.2 Hz), 24.39 ppm; **^{19}F NMR** (282 MHz, $CDCl_3$) δ -126.68 ppm; **HRMS** (ESI): $C_{19}H_{13}FNaO$ $[M+Na]^+$ calcd: 299.0843, found: 299.0833.

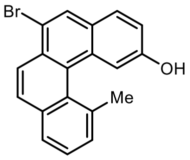

1i

White solid, 34% overall yield; **^{1}H NMR** (300 MHz, $CDCl_3$) δ 8.16 (d, J = 8.4 Hz, 2H), 7.83 (dd, J = 12.6, 8.3 Hz, 2H), 7.74 (d, J = 8.7 Hz, 1H), 7.56 (t, J = 7.5 Hz, 1H), 7.45 (d, J = 7.2 Hz, 1H), 7.29 (d, J = 2.5 Hz, 1H), 7.11 (dd, J = 8.7, 2.5 Hz, 1H), 5.24 (s, 1H), 2.33 (s, 3H) ppm; **^{13}C NMR** (75 MHz, $CDCl_3$) δ 153.51, 135.46, 133.83, 130.90, 130.56, 130.08, 130.02, 129.18, 128.72, 128.37, 127.94, 127.25, 126.33, 125.39, 124.50, 118.23, 116.78, 113.51, 24.69 ppm.

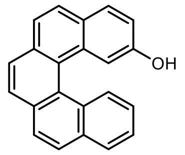

1j

White solid, 26% overall yield; **^{1}H NMR** (300 MHz, DMSO-d_6) δ 9.59 (s, 1H), 8.42 (d, *J* = 8.6 Hz, 1H), 8.18 – 7.82 (m, 7H), 7.82 – 7.62 (m, 2H), 7.57 (t, *J* = 7.4 Hz, 1H), 7.35 (t, *J* = 7.7 Hz, 1H), 7.14 (d, *J* = 8.3 Hz, 1H) ppm; **^{13}C NMR** (75 MHz, DMSO-d_6) δ 154.69, 132.41, 132.20, 131.77, 131.63, 129.57, 129.54, 128.32, 127.90, 127.45, 127.31, 127.17, 127.06, 126.38, 126.29, 126.25, 124.99, 124.50, 122.92, 117.87, 111.51 ppm; **HRMS** (ESI): $C_{22}H_{14}NaO$ $[M+Na]^+$ calcd: 317.0937, found: 317.0925.

1.2.3 Synthesis of 12-methylbenzo[*c*]phenanthren-2-amine

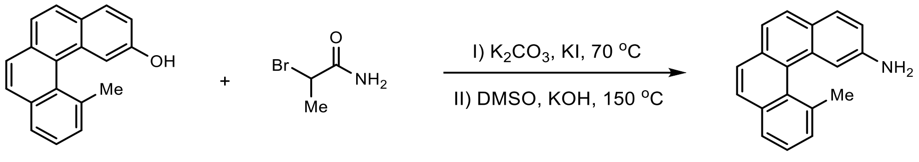

1a   1a'

To a suspension of **1a** (1.0 mmol), K_2CO_3 (3.0 mmol) and KI (0.1 mmol) in DMSO (5.0 mL) was added 2-bromopropanamide (3.0 mmol). The resulting solution was stirred at 70 °C until the complete consumption of **1a**. Then, KOH (4.0 mmol) was added and the reaction mixture was further stirred for 10 h at 150 °C. The reaction was quenched by water and the aqueous layer was extracted with ethyl acetate for three times. The combined organic layers were washed with brine, dried over anhydrous Na_2SO_4, and concentrated under reduced pressure. The crude product was purified by silica column chromatography to afford 12-methylbenzo[*c*]phenanthren-2-amine **1a'**. 38% yield, brown solid. **^{1}H NMR** (300 MHz, $CDCl_3$) δ 7.78 (dd, *J* = 11.1, 5.6 Hz, 4H), 7.67 (d, *J* = 8.4 Hz, 1H), 7.63 – 7.40 (m, 3H), 7.20 (s, 1H), 6.97 (d, *J* = 8.5 Hz, 1H), 3.89 (s, 2H), 2.42 (s, 3H) ppm; **^{13}C NMR** (75 MHz, $CDCl_3$) δ 143.99, 135.40, 133.86, 132.46, 131.85, 129.95, 129.47, 128.78, 127.52, 127.26, 126.31, 126.00, 125.85, 125.43, 122.55, 116.42, 112.83, 24.78 ppm; **HRMS** (ESI): $C_{19}H_{16}N$ $[M+H]^+$ calcd: 258.1277, found: 258.1283.

1.2.4 Preparation of catalyst C6

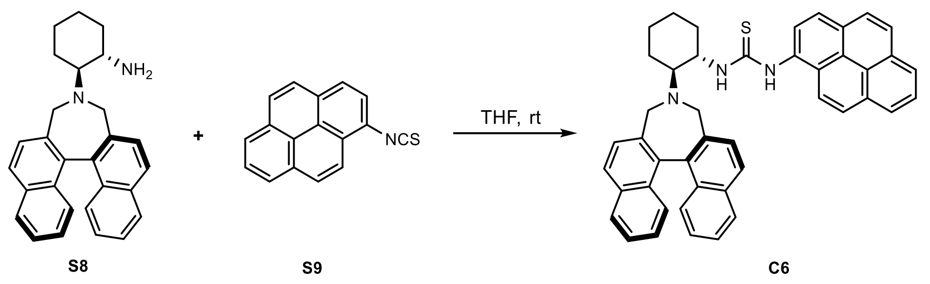

To a solution of (1*S*,2*S*)-cyclohexane-1,2-diamine derived intermediate **S8**[4] (0.78 g, 2.0 mmol) in THF (10 mL) was added 1-isothiocyanatopyrene **S9**[5] (0.62 g, 2.4 mmol) and the reaction mixture was stirred for 8 h at room temperature. Upon the complete consumption of **S8**, the reaction mixture was concentrated and the crude product was purified by flash chromatograph on silica gel (petroleum ether/ethyl acetate = 2/1 to 1/1) to give pure catalyst **C6** (79% yield) as a pale yellow solid. **^{1}H NMR** (300 MHz, $CDCl_3$) δ 8.13 (dd, J = 13.3, 8.4 Hz, 3H), 7.85 (dtd, J = 33.9, 18.3, 16.6, 8.6 Hz, 10H), 7.58 (d, J = 8.3 Hz, 2H), 7.52 – 7.35 (m, 5H), 7.34 – 7.01 (m, 3H), 6.33 (d, J = 6.2 Hz, 1H), 4.30 (dt, J = 15.8, 8.8 Hz, 1H), 3.65 – 3.14 (m, 4H), 2.65 (d, J = 12.3 Hz, 1H), 2.55 – 2.24 (m, 1H), 1.59 (q, J = 18.0, 17.2 Hz, 4H), 1.24 (dd, J = 14.7, 10.4 Hz, 2H), 1.02 (dt, J = 39.7, 12.2 Hz, 2H) ppm; **^{13}C NMR** (75 MHz, $CDCl_3$) δ 134.64, 132.93, 131.27, 130.92, 130.71, 128.66, 128.25, 127.97, 127.82, 127.45, 127.36, 126.81, 126.40, 125.75, 125.68, 125.62, 125.35, 125.21, 125.04, 124.18, 121.60, 69.90, 56.59, 53.43, 32.95, 27.80, 25.54, 24.53 ppm.

1.3 General procedure for enantioselective synthesis of 1,12-disubstituted [4]helicenes

1.3.1 General procedure of preparing racemic samples

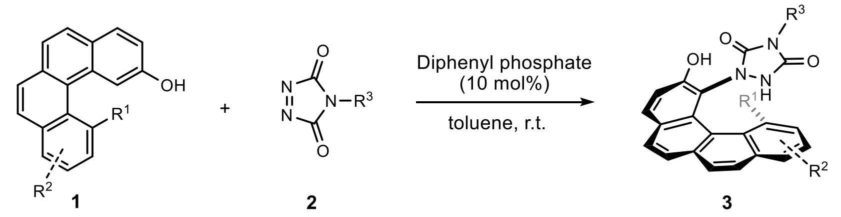

To a mixture of substituted 2-hydroxybenzo[*c*]phenanthrenes **1** (0.05 mmol) and diazodicarboxamides **2** (0.05 mmol) in toluene (0.5 mL) was added diphenyl phosphate (0.005 mmol). The resulting solution was stirred at room temperature for 1 h (monitored by TLC). Upon completion, the residue was directly purified by silica gel column chromatography to afford racemic products **3**.

1.3.2 General procedure of preparing chiral samples

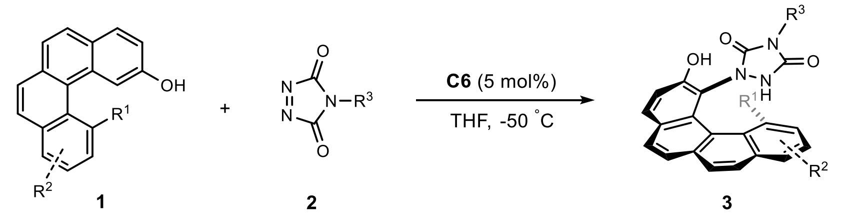

The mixture of substituted polycyclic phenols **1** (0.2 mmol, 1.0 equiv) and catalyst **C6** (0.01 mmol) was dissolved in 1.0 mL of THF and stirred at room temperature for 10 min. Then, the solution was cooled to -50 °C and stirred for another 10 min before diazodicarboxamides **2** (0.1 mmol, 0.5 equiv)

was added. The resulting mixture was stirred at this temperature until the complete consumption of **2**. After monitored by TLC, the solvent was removed under reduced pressure, and the residue was purified by silica gel column chromatography to afford the desired products **3**. (It should be noted that the configurationally unstable C-N axis of products **3** make their NMR spectra too messy to clearly record the corresponding data. Therefore, double methylated helicenes **4** bearing a configurationally stable C-N stereogenic axis were synthesized for better NMR spectra collection.)

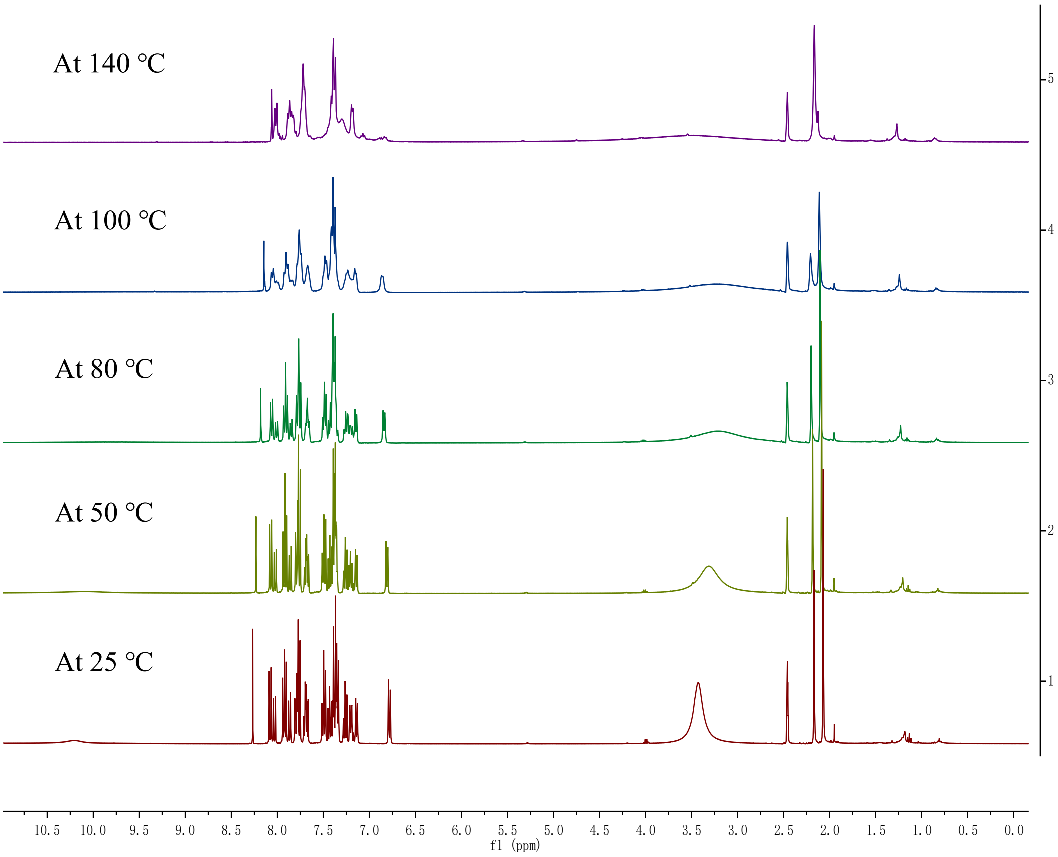

Supplementary Fig. 2. ^{1}H NMR spectra of **3a** in *d6*-DMSO at varying temperatures.

1.4 General procedure for methylation of chiral 1,12-disubstituted [4]helicenes 3

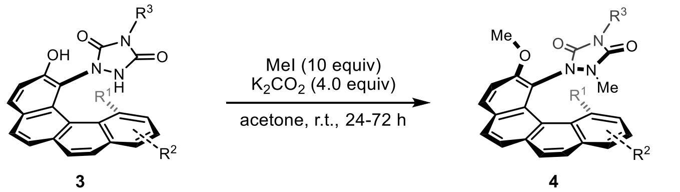

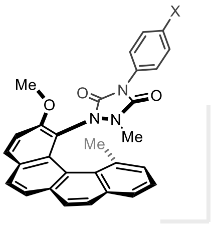

4a, X = H, 90% yield, 6.1:1 dr, 96.5:3.5 er
4b, X = Me, 97% yield, 5.4:1 dr, 98:2 er
4c, X = OMe, 78% yield, 4.8:1 dr, 98:2 er
4d, X = OCF_3, 92% yield, 5.6:1 dr, 98:2 er
4e, X = F, 95% yield, 5.4:1 dr, 97.5:2.5 er
4f, X = Cl, 92% yield, 5:1 dr, 96:4 er
4g, X = Br, 76% yield, 11:1 dr, 97:3 er
4h, X = I, 82% yield, 11:1 dr, 96:4 er
4i, X = *n*Bu, 91% yield, 6.5:1 dr, 97:3 er
4j, X = Ph, 92% yield, 4.9:1 dr, 95.5:4.5 er

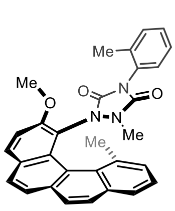

4k, 90% yield
4.8:1 dr, 99:1 er

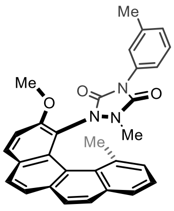

4l, 91% yield
5.2:1 dr, 96.5:3.5 er

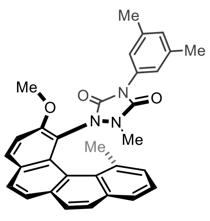

4m, 93% yield
4.6:1 dr, 95.5:4.5 er

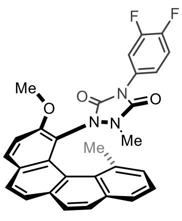

4n, 98% yield
5.5:1 dr, 96.5:3.5 er

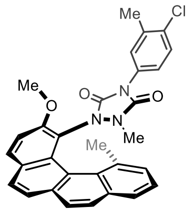

4o, 96% yield
5:1 dr, 94.5:5.5 er

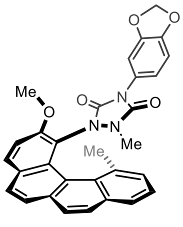

4p, 88% yield
4.5:1 dr, 96.5:3.5 er

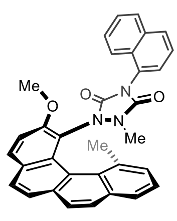

4q, 93% yield
6.5:1 dr, 99:1 er

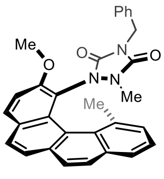

4r, 97% yield
6.4:1 dr, 96:4 er

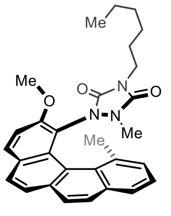

4s, 92% yield
5.4:1 dr, 96:4 er

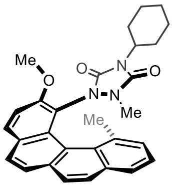

4t, 88% yield
5.4:1 dr, 98:2 er

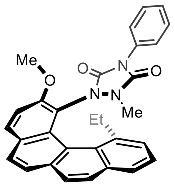

4u, 79% yield
6:1 dr, 98:2 er

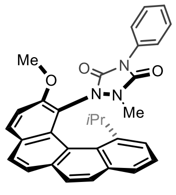

4v, 94% yield
4.6:1 dr, 98.5:1.5 er

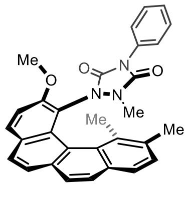

4w, 95% yield
18:1 dr, 92.5:7.5 er

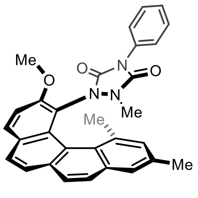

4x, 86% yield
4.9:1 dr, 85:15 er

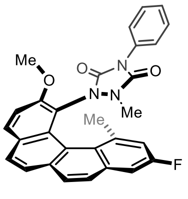

4y, 93% yield
5.3:1 dr, 92:8 er

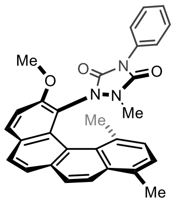

4z, 88% yield
2.7:1 dr, 95:5 er

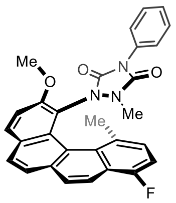

4aa, 92% yield
6.3:1 dr, 96:4 er

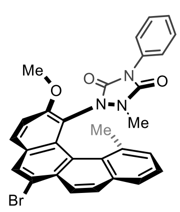

4ab, 86% yield
3.3:1 dr, 98:2 er

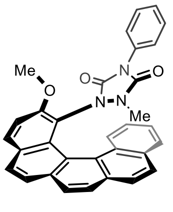

4ac, 85% yield
5:1 dr, 88.5:11.5 er

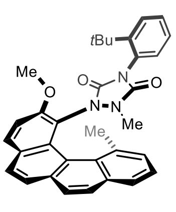

4ad, 95% yield
6:1 dr, 97.5:2.5 er

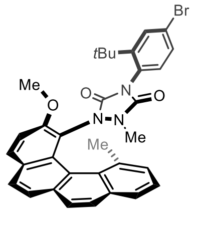

4ae, 96% yield
6.6:1 dr, 97.5:2.5 er

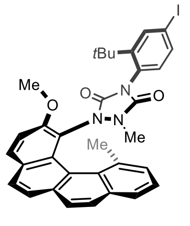

4af, 97% yield
7.2:1 dr, 96.5:3.5 er

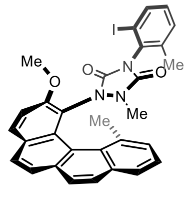

4ag, 95% yield
4.5:1 dr, 98.5:1.5 er

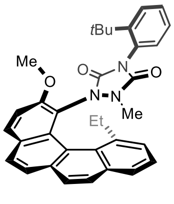

4ah, 91% yield
6.2:1 dr, 97.5:2.5 er

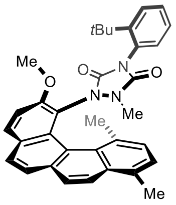

4ai, 88% yield
4.3:1 dr, 97:3 er

Supplementary Fig. 3. Double methylation of enantioenriched helicenes **3.**

To a mixture of helicenes **3** (1.0 equiv) and iodomethane (10 equiv) in acetone (0.1 M) was added potassium carbonate (4.0 equiv) at room temperature. The reaction mixture was stirred until the complete consumption of the starting material, the progress of which was monitored by TLC analysis. Then the solvent was removed under vacuum and the residue was purified by silica gel chromatography to afford pure products **4**.

1.5 Characterization data of 3 and 4

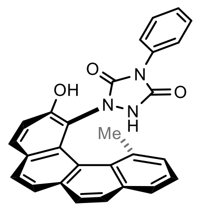

3a

Prepared according to the general procedure on a 0.2 mmol scale and purified by flash chromatography (petroleum ether/ethyl acetate = 4/1 to 2/1). White solid, 39.4 mg, 45% yield, 96.5:3.5 er; **HPLC analysis**: Chiralcel AD-H (*n*-hexane/*i*-PrOH(1‰ TFA) = 7/3, flow rate = 1.0 mL/min), t_{major} = 6.92 min, t_{minor} = 14.88 min; **HRMS** (ESI): $C_{27}H_{20}N_3O_3$ $[M+H]^+$ calcd: 434.1499, found: 434.1497.

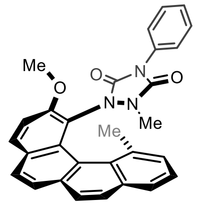

4a

White solid, m.p. 246 – 247 °C, 90% yield, 6.1:1 dr, 96.5:3.5 er; $[\alpha]_D^{24}$ = -495 (c = 1.0 in $CHCl_3$); **HPLC analysis**: Chiralcel IA-H (*n*-hexane/*i*-PrOH(1‰ TFA) = 7/3, flow rate = 1.0 mL/min), t_{major} = 8.41 min, t_{minor} = 24.89 min; **^{1}H NMR** (300 MHz, $CDCl_3$) δ 8.15 (d, *J* = 9.0 Hz, 1H), 7.88 (dd, *J* = 8.5, 4.0 Hz, 2H), 7.82 – 7.60 (m, 3H), 7.62 – 7.43 (m, 6H), 7.37 (q, *J* = 4.7 Hz, 1H), 7.25 (d, *J* = 6.1 Hz, 1H), 4.03 (s, 3H), 2.25 (s, 3H), 1.42 (s, 3H) ppm; **^{13}C NMR** (75 MHz, $CDCl_3$) δ 155.21, 151.21, 148.03, 136.17, 133.43, 132.29, 131.95, 131.09, 130.77, 129.81, 128.95, 128.89, 127.69, 127.62, 127.58, 126.73, 126.19, 125.64, 124.48, 124.38, 124.27, 122.75, 117.16, 112.82, 57.01, 29.71, 23.03 ppm; **HRMS** (ESI): $C_{29}H_{24}N_3O_3$ $[M+H]^+$ calcd: 462.1812, found: 462.1800.

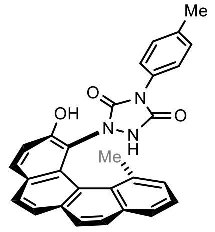

3b

Prepared according to the general procedure on a 0.2 mmol scale and purified by flash chromatography (petroleum ether/ethyl acetate = 4/1 to 2/1). White solid, 32.7 mg, 37% yield, 97.5:2.5 er; **HPLC analysis**: Chiralcel AD-H (*n*-hexane/*i*-PrOH(1‰ TFA) = 7/3, flow rate = 1.0 mL/min), t_{major} = 9.92 min, t_{minor} = 23.48 min; **HRMS** (ESI): $C_{28}H_{22}N_3O_3$ $[M+H]^+$ calcd: 448.1656, found: 448.1644.

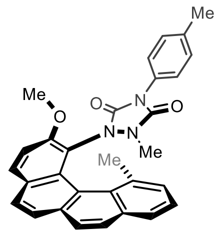

4b

White solid, m.p. 256 – 257 °C, 97% yield, 5.4:1 dr, 98:2 er; $[\alpha]_D^{24}$ = -486 (c = 1.0 in $CHCl_3$); **HPLC analysis**: Chiralcel IA-H (*n*-hexane/*i*-PrOH(1‰ TFA) = 7/3, flow rate = 1.0 mL/min), t_{major} = 11.33 min, t_{minor} = 40.98 min; **^{1}H NMR** (300 MHz, $CDCl_3$) δ 8.13 (d, *J* = 9.0 Hz, 1H), 8.01 – 7.82 (m, 2H), 7.82 – 7.58 (m, 3H), 7.62 – 7.40 (m, 2H), 7.41 – 7.01 (m, 5H), 4.02 (s, 3H), 2.40 (s, 3H), 2.24 (s, 3H), 1.41 (s, 3H) ppm; **^{13}C NMR** (75 MHz, $CDCl_3$) δ 155.25, 151.45, 148.26, 137.60, 136.25, 133.45, 132.32, 131.07, 130.83, 129.89, 129.56, 129.33, 128.97, 127.72, 127.61, 126.75, 126.21, 125.60, 124.50, 124.40, 124.28, 122.82, 117.35, 112.88, 57.04, 29.76, 23.07, 21.24 ppm; **HRMS** (ESI): $C_{30}H_{26}N_3O_3$ $[M+H]^+$ calcd: 476.1969, found: 476.1958.

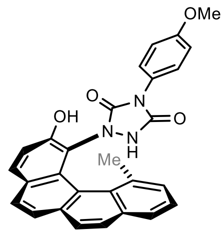

3c

Prepared according to the general procedure on a 0.2 mmol scale and purified by flash chromatography (petroleum ether/ethyl acetate = 2/1 to 1/1). White solid, 38.3 mg, 41% yield, 97.5:2.5 er; **HPLC analysis**: Chiralcel AD-H (*n*-hexane/*i*-PrOH(1‰ TFA) = 6/4, flow rate = 1.0 mL/min), t_{major} = 8.69 min, t_{minor} = 15.25 min; **HRMS** (ESI): $C_{28}H_{21}N_3NaO_4$ $[M+Na]^+$ calcd: 486.1424, found: 486.1422.

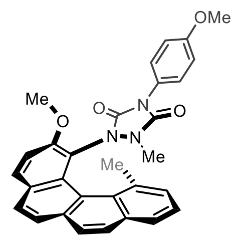

4c

White solid, m.p. 243 – 244 °C, 78% yield, 4.8:1 dr, 98:2 er; $[\alpha]_D^{24}$ = -467 (c = 1.0 in $CHCl_3$); **HPLC analysis**: Chiralcel IA-H (*n*-hexane/*i*-PrOH(1‰ TFA) = 6/4, flow rate = 1.0 mL/min), t_{major} = 10.66 min, t_{minor} = 26.24 min; **^{1}H NMR** (300 MHz, $CDCl_3$) δ 8.14 (d, *J* = 8.9 Hz, 1H), 7.86 (s, 2H), 7.82 – 7.62 (m, 3H), 7.41 (dd, *J* = 24.0, 8.7 Hz, 4H), 7.25 (s, 1H), 7.01 (d, *J* = 8.5 Hz, 2H), 4.03 (s, 3H), 3.85 (s, 3H), 2.25 (s, 3H), 1.41 (s, 3H) ppm; **^{13}C NMR** (75 MHz, $CDCl_3$) δ 158.92, 155.24, 151.57, 148.43, 136.29, 133.45, 132.33, 131.07, 130.84, 129.93, 128.97, 127.69, 127.61, 127.16, 126.75, 126.17, 124.70, 124.51, 124.41, 124.29, 122.83, 117.38, 114.29, 112.88, 57.04, 55.55, 29.76, 23.08 ppm; **HRMS** (ESI): $C_{30}H_{26}N_3O_4$ $[M+H]^+$ calcd: 492.1918, found: 492.1910.

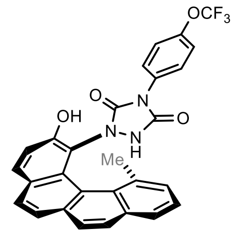

3d

Prepared according to the general procedure on a 0.2 mmol scale and purified by flash chromatography (petroleum ether/ethyl acetate = 4/1 to 2/1). White solid, 41.0 mg, 40% yield, 98:2 er; **HPLC analysis**: Chiralcel AD-H (*n*-hexane/*i*-PrOH(1‰ TFA) = 7/3, flow rate = 1.0 mL/min), t_{major} = 9.29 min, t_{minor} = 14.13 min; **HRMS** (ESI): $C_{28}H_{18}F_3N_3NaO_4$ $[M+Na]^+$ calcd: 540.1142, found: 540.1143.

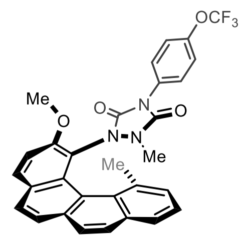

4d

White solid, m.p. 163 – 164 °C, 92% yield, 5.6:1 dr, 98:2 er; $[\alpha]_D^{24}$ = -476 (c = 1.0 in $CHCl_3$); **HPLC analysis**: Chiralcel IA-H (*n*-hexane/*i*-PrOH(1‰ TFA) = 7/3, flow rate = 1.0 mL/min), t_{major} = 7.75 min, t_{minor} = 13.22 min; **^{1}H NMR** (300 MHz, $CDCl_3$) δ 8.16 (d, *J* = 9.0 Hz, 1H), 7.88 (d, *J* = 7.7 Hz, 2H), 7.83 – 7.64 (m, 3H), 7.57 (d, *J* = 8.3 Hz, 2H), 7.46 (d, *J* = 8.9 Hz, 2H), 7.34 (d, *J* = 8.2 Hz, 2H), 7.29 – 7.06 (m, 1H), 4.04 (s, 3H), 2.23 (s, 3H), 1.42 (s, 3H) ppm; **^{13}C NMR** (75 MHz, $CDCl_3$) δ 155.22, 150.66, 147.94, 147.44, 136.06, 133.53, 132.35, 131.27, 130.70, 130.54, 129.62, 129.01, 127.62, 127.59, 126.80, 126.12, 124.56, 124.50, 124.34, 122.64, 121.48, 116.64, 112.76, 57.01, 29.64, 22.99 ppm; **^{19}F NMR** (282 MHz, $CDCl_3$) δ -57.80 ppm; **HRMS** (ESI): $C_{30}H_{23}F_3N_3O_4$ $[M+H]^+$ calcd: 546.1635, found: 546.1623.

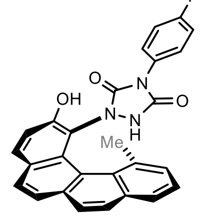

3e

Prepared according to the general procedure on a 0.2 mmol scale and purified by flash chromatography (petroleum ether/ethyl acetate = 4/1 to 2/1). White solid, 37.3 mg, 41% yield, 97.5:2.5 er; **HPLC analysis**: Chiralcel AD-H (*n*-hexane/*i*-PrOH(1‰ TFA) = 7/3, flow rate = 1.0 mL/min), t_{major} = 9.50 min, t_{minor} = 12.24 min; **HRMS** (ESI): $C_{27}H_{18}FN_3NaO_3$ $[M+Na]^+$ calcd: 474.1224, found: 474.1222.

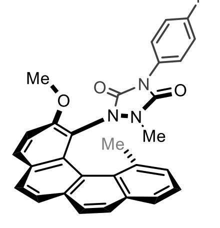

4e

White solid, m.p. 252 – 253 °C, 95% yield, 5.4:1 dr, 97.5:2.5 er; $[\alpha]_D^{24}$ = - 535 (c = 1.0 in $CHCl_3$); **HPLC analysis**: Chiralcel IA-H (*n*-hexane/EtOH(1‰ TFA) = 7/3, flow rate = 1.0 mL/min), t_{major} = 10.01 min, t_{minor} = 15.53 min; **^{1}H NMR** (300 MHz, $CDCl_3$) δ 8.15 (d, *J* = 8.9 Hz, 1H), 7.88 (dd, *J* = 8.6, 3.6 Hz, 2H), 7.82 – 7.58 (m, 3H), 7.58 – 7.31 (m, 4H), 7.19 (q, *J* = 9.1, 8.2 Hz, 3H), 4.03 (s, 3H), 2.23 (s, 3H), 1.42 (s, 3H) ppm; **^{13}C NMR** (75 MHz, $CDCl_3$) δ 155.20, 150.98, 147.76, 136.12, 133.48, 132.32, 131.18, 130.73, 129.68, 128.97, 127.61, 127.57, 127.47, 127.35, 126.76, 126.09, 124.53, 124.46, 124.30, 122.68, 116.86, 115.98,

115.68, 112.78, 57.00, 29.66, 23.00 ppm; **^{19}F NMR** (282 MHz, $CDCl_3$) δ -113.76 ppm; **HRMS** (ESI): $C_{29}H_{23}FN_3O_3$ $[M+H]^+$ calcd: 480.1718, found: 480.1711.

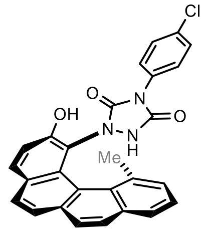

3f

Prepared according to the general procedure on a 0.2 mmol scale and purified by flash chromatography (petroleum ether/ethyl acetate = 4/1 to 2/1). White solid, 41.8 mg, 45% yield, 96:4 er; **HPLC analysis**: Chiralcel AD-H (*n*-hexane/*i*-PrOH(1‰ TFA) = 7/3, flow rate = 1.0 mL/min), t_{major} = 11.73 min, t_{minor} = 17.25 min; **HRMS** (ESI): $C_{27}H_{18}ClN_3NaO_3$ $[M+Na]^+$ calcd: 490.0929, found: 490.0918.

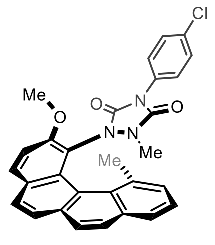

4f

White solid, m.p. 253 – 254 °C, 92% yield, 5:1 dr, 96:4 er; $[\alpha]_D^{24}$ = -453 (c = 1.0 in $CHCl_3$); **HPLC analysis**: Chiralcel IA-H (*n*-hexane/EtOH(1‰ TFA) = 7/3, flow rate = 1.0 mL/min), t_{major} = 10.52 min, t_{minor} = 18.75 min; **^{1}H NMR** (600 MHz, $CDCl_3$) δ 8.15 (d, *J* = 8.9 Hz, 1H), 7.88 (t, *J* = 7.7 Hz, 2H), 7.78 (d, *J* = 7.8 Hz, 1H), 7.72 (d, *J* = 8.3 Hz, 1H), 7.68 (d, *J* = 8.2 Hz, 1H), 7.55 – 7.35 (m, 6H), 7.21 (d, *J* = 7.0 Hz, 1H), 4.03 (s, 3H), 2.21 (s, 3H), 1.42 (s, 3H) ppm; **^{13}C NMR** (151 MHz, $CDCl_3$) δ 155.26, 150.71, 147.40, 136.04, 133.54, 133.15, 132.37, 131.24, 130.72, 130.62, 129.59, 129.14, 129.07, 129.03, 127.67, 127.61, 126.81, 126.66, 126.14, 124.57, 124.52, 124.36, 122.68, 116.71, 112.80, 57.04, 29.69, 23.01 ppm; **HRMS** (ESI): $C_{29}H_{22}ClN_3NaO_3$ $[M+Na]^+$ calcd: 518.1242, found: 518.1247.

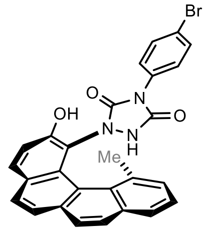

3g

Prepared according to the general procedure on a 0.2 mmol scale and purified by flash chromatography (petroleum ether/ethyl acetate = 4/1 to 2/1). White solid, 40.7 mg, 40% yield, 96.5:3.5 er; **HPLC analysis**: Chiralcel AD-H (*n*-hexane/*i*-PrOH(1‰ TFA) = 7/3, flow rate = 1.0 mL/min), t_{major} = 13.17 min, t_{minor} = 19.93 min; **HRMS** (ESI): $C_{27}H_{18}BrN_3NaO_3$ $[M+Na]^+$ calcd: 534.0424, found: 534.0416.

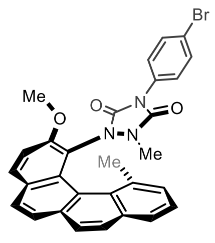

4g

White solid, m.p. 243 – 244 °C, 76% yield, 11:1 dr, 97:3 er; $[\alpha]_D^{24}$ = -414 (c = 1.0 in $CHCl_3$); **HPLC analysis**: Chiralcel IA-H (*n*-hexane/*i*-PrOH(1‰ TFA) = 7/3, flow rate = 1.0 mL/min), t_{major} = 11.28 min, t_{minor} = 34.98 min; **^{1}H NMR** (300 MHz, $CDCl_3$) δ 8.15 (d, *J* = 9.0 Hz, 1H), 7.88 (d, *J* = 7.8 Hz, 2H), 7.82 – 7.65 (m, 3H), 7.61 (d, *J* = 8.3 Hz, 2H), 7.44 (t, *J* = 9.8 Hz, 4H), 7.20 (d, *J* = 7.2 Hz, 1H), 4.03 (s, 3H), 2.21 (s, 3H), 1.41 (s, 3H) ppm; **^{13}C NMR** (75 MHz, $CDCl_3$) δ 155.25, 150.61, 147.27, 136.00, 133.53, 132.35, 132.02, 131.23, 131.14, 130.69, 129.54, 129.03, 127.66, 127.59, 126.92, 126.80, 126.13, 124.56, 124.51, 124.35, 122.66, 121.12, 116.64, 112.77, 57.03, 29.68, 22.99 ppm; **HRMS** (ESI): $C_{29}H_{23}BrN_3O_3$ $[M+H]^+$ calcd: 540.0917, found: 540.0902.

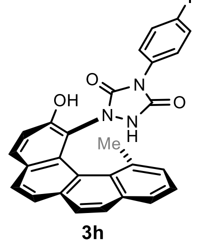

3h

Prepared according to the general procedure on a 0.2 mmol scale and purified by flash chromatography (petroleum ether/ethyl acetate = 4/1 to 2/1). White solid, 41.3 mg, 37% yield, 96:4 er; **HPLC analysis**: Chiralcel AD-H (*n*-hexane/*i*-PrOH(1‰ TFA) = 7/3, flow rate = 1.0 mL/min), t_{major} = 14.83 min, t_{minor} = 20.92 min; **HRMS** (ESI): $C_{27}H_{18}IN_3NaO_3$ $[M+Na]^+$ calcd: 582.0285, found: 582.0270.

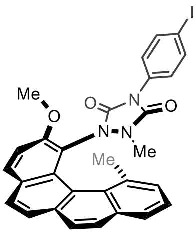

4h

White solid, m.p. 137 – 138 °C, 82% yield, 11:1 dr, 96:4 er; $[\alpha]_D^{24}$ = -303 (c = 1.0 in $CHCl_3$); **HPLC analysis**: Chiralcel IA-H (*n*-hexane/*i*-PrOH(1‰ TFA) = 7/3, flow rate = 1.0 mL/min), t_{major} = 11.65 min, t_{minor} = 32.22 min; **^{1}H NMR** (300 MHz, $CDCl_3$) δ 8.16 (d, *J* = 8.9 Hz, 1H), 8.04 – 7.61 (m, 7H), 7.57 – 7.37 (m, 2H), 7.37 – 7.24 (m, 2H), 7.20 (d, *J* = 6.9 Hz, 1H), 4.03 (s, 3H), 2.21 (s, 3H), 1.41 (s, 3H) ppm; **^{13}C NMR** (75 MHz, $CDCl_3$) δ 155.22, 150.53, 147.18, 137.96, 135.97, 133.50, 132.32, 131.85, 131.19, 130.66, 129.51, 129.00, 127.64, 127.56, 127.08, 126.77, 126.11, 124.52, 124.48, 124.32, 122.63, 116.61, 112.74, 92.50, 57.01, 29.65, 22.95 ppm; **HRMS** (ESI): $C_{29}H_{23}IN_3O_3$ $[M+H]^+$ calcd: 588.0779, found: 588.0759.

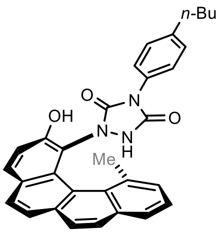

3i

Prepared according to the general procedure on a 0.2 mmol scale and purified by flash chromatography (petroleum ether/ethyl acetate = 4/1 to 2/1). White solid, 45.3 mg, 46% yield, 97:3 er; **HPLC analysis**: Chiralcel AD-H (*n*-hexane/*i*-PrOH(1‰ TFA) = 7/3, flow rate = 1.0 mL/min), t_{major} = 10.39 min, t_{minor} = 29.62 min; **HRMS** (ESI): $C_{31}H_{27}N_3NaO_3$ $[M+Na]^+$ calcd: 512.1945, found: 512.1947.

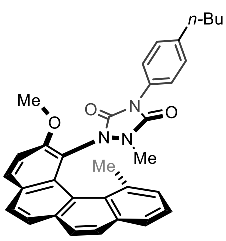

4i

White solid, m.p. 97 – 98 °C, 91% yield, 6.5:1 dr, 97:3 er; $[\alpha]_D^{24}$ = -470 (c = 1.0 in $CHCl_3$); **HPLC analysis**: Chiralcel IA-H (*n*-hexane/EtOH(1‰ TFA) = 7/3, flow rate = 1.0 mL/min), t_{major} = 10.71 min, t_{minor} = 21.39 min; **^{1}H NMR** (600 MHz, $CDCl_3$) δ 8.13 (d, *J* = 8.9 Hz, 1H), 7.86 (t, *J* = 8.6 Hz, 2H), 7.77 (d, *J* = 7.9 Hz, 1H), 7.71 (d, *J* = 8.3 Hz, 1H), 7.67 (d, *J* = 8.3 Hz, 1H), 7.46 (q, *J* = 9.0, 8.2 Hz, 2H), 7.37 (d, *J* = 7.9 Hz, 2H), 7.27 (dd, *J* = 14.6, 7.6 Hz, 3H), 4.02 (s, 3H), 2.65 (t, *J* = 7.7 Hz, 2H), 2.25 (s, 3H), 1.68 – 1.57 (m, 2H), 1.41 (s, 3H), 1.40 – 1.33 (m, 2H), 0.95 (t, *J* = 7.3 Hz, 3H) ppm; **^{13}C NMR** (151 MHz, $CDCl_3$) δ 155.23, 151.48, 148.39, 142.51, 136.28, 133.42, 132.30, 131.03, 130.83, 129.96, 129.46, 128.93, 128.91, 127.70, 127.61, 126.71, 126.20, 125.52, 124.47, 124.34, 124.26, 122.83, 117.48, 112.90, 57.02, 35.35, 33.52, 29.73, 23.04, 22.35, 13.95 ppm; **HRMS** (ESI): $C_{33}H_{31}N_3NaO_3$ $[M+Na]^+$ calcd: 540.2258, found: 540.2265.

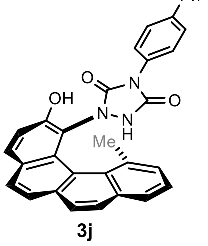

3j

Prepared according to the general procedure on a 0.2 mmol scale and purified by flash chromatography (petroleum ether/ethyl acetate = 4/1 to 2/1). White solid, 45.2 mg, 44% yield, 95.5:4.5 er; **HPLC analysis**: Chiralcel AD-H (*n*-hexane/*i*-PrOH(1‰ TFA) = 7/3, flow rate = 1.0 mL/min), t_{major} = 20.00 min, t_{minor} = 23.90 min; **HRMS** (ESI): $C_{33}H_{23}N_3NaO_3$ $[M+Na]^+$ calcd: 532.1632, found: 532.1630.

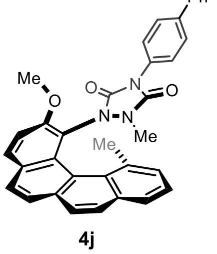

4j

White solid, m.p. 154 – 155 °C, 92% yield, 4.9:1 dr; 95.5:4.5 er; $[\alpha]_D^{24}$ = -374 (c = 1.0 in $CHCl_3$); **HPLC analysis**: Chiralcel IA-H (*n*-hexane/*i*-PrOH(1‰ TFA) = 7/3, flow rate = 1.0 mL/min), t_{major} = 14.68 min, t_{minor} = 31.72 min; **^{1}H NMR** (300 MHz, $CDCl_3$) δ 8.14 (d, *J* = 8.9 Hz, 1H), 7.87 (t, *J* = 7.9 Hz, 2H), 7.79 (d, *J* = 7.7 Hz, 1H), 7.75 – 7.65 (m, 4H), 7.60 (ddd, *J* = 10.6, 8.0, 1.7 Hz, 4H), 7.54 – 7.31 (m, 5H), 7.31 – 7.20 (m, 1H), 4.03 (s, 3H), 2.26 (s, 3H), 1.43 (s, 3H) ppm; **^{13}C NMR** (75 MHz, $CDCl_3$) δ 155.29, 151.18, 147.95, 140.61, 136.18, 133.51, 132.37, 131.18, 130.81, 129.78, 129.03, 128.88, 127.75, 127.63, 127.53, 127.28,

126.80, 126.25, 125.86, 124.55, 124.48, 124.33, 122.78, 117.07, 112.87, 57.07, 29.78, 23.08 ppm; **HRMS** (ESI): $C_{35}H_{28}N_3O_3$ $[M+H]^+$ calcd: 538.2125, found: 538.2114.

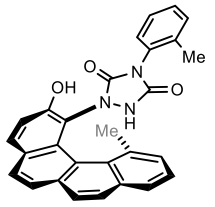

3k

Prepared according to the general procedure on a 0.2 mmol scale and purified by flash chromatography (petroleum ether/ethyl acetate = 4/1 to 2/1). White solid, 36.9 mg, 41% yield, 98:2 er; **HPLC analysis**: Chiralcel AD-H (*n*-hexane/*i*-PrOH(1‰ TFA) = 7/3, flow rate = 1.0 mL/min), t_{major} = 4.58 min, t_{minor} = 11.05 min; **HRMS** (ESI): $C_{28}H_{22}N_3O_3$ $[M+H]^+$ calcd: 448.1656, found: 448.1658.

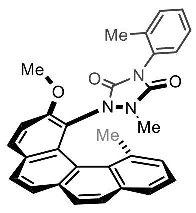

4k

White solid, m.p. 271 – 272 °C, 90% yield, 4.8:1 dr, 99:1 er; $[\alpha]_D^{24}$ = -521 (c = 1.0 in $CHCl_3$); **HPLC analysis**: Chiralcel IA-H (*n*-hexane/EtOH(1‰ TFA) = 7/3, flow rate = 1.0 mL/min), t_{major} = 8.11 min, t_{minor} = 20.00 min; **^{1}H NMR** (300 MHz, $CDCl_3$, two rotamers) δ 8.15 (d, *J* = 8.9 Hz, 1H), 7.99 – 7.62 (m, 5H), 7.50 (dd, *J* = 35.1, 8.3 Hz, 2H), 7.39 – 6.85 (m, 5H), 4.00 (s, 3H), 2.31 (s, 3H), 2.26 (d, *J* = 71.5 Hz, 3H), 1.39 (s, 3H) ppm; **^{13}C NMR** (75 MHz, $CDCl_3$, two rotamers) δ 155.09, 154.89, 152.01, 151.74, 151.55, 148.97, 137.96, 137.22, 136.89, 136.72, 133.44, 133.36, 132.31, 132.22, 131.52, 131.38, 131.15, 131.08, 130.98, 130.38, 130.29, 129.32, 128.81, 128.68, 128.56, 128.38, 127.78, 127.65, 127.62, 127.41, 126.87, 126.74, 126.30, 126.19, 124.80, 124.66, 124.27, 123.86, 123.20, 122.98, 119.98, 117.70, 113.03, 112.83, 56.96, 56.79, 29.41, 23.57, 23.29, 17.88, 17.47 ppm; **HRMS** (ESI): $C_{30}H_{26}N_3O_3$ $[M+H]^+$ calcd: 476.1969, found: 476.1966.

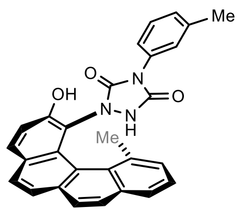

3l

Prepared according to the general procedure on a 0.2 mmol scale and purified by flash chromatography (petroleum ether/ethyl acetate = 4/1 to 2/1). White solid, 39.7 mg, 44% yield, 96:4 er; **HPLC analysis**: Chiralcel AD-H (*n*-hexane/*i*-PrOH(1‰ TFA) = 7/3, flow rate = 1.0 mL/min), t_{major} = 5.54 min, t_{minor} = 7.93 min; **HRMS** (ESI): $C_{28}H_{21}N_3NaO_3$ $[M+Na]^+$ calcd: 470.1475, found: 470.1471.

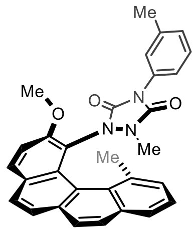

4l

White solid, m.p. 224 – 225 °C, 91% yield, 5.2:1 dr, 96.5:3.5 er; $[\alpha]_D^{24}$ = -516 (c = 1.0 in $CHCl_3$); **HPLC analysis**: Chiralcel AD-H (*n*-hexane/*i*-PrOH(1‰ TFA) = 7/3, flow rate = 1.0 mL/min), t_{major} = 6.42 min, t_{minor} = 13.59 min; **^{1}H NMR** (300 MHz, $CDCl_3$) δ 8.14 (d, *J* = 9.0 Hz, 1H), 7.98 – 7.81 (m, 2H), 7.78 (d, *J* = 7.9 Hz, 1H), 7.69 (dd, *J* = 12.6, 8.5 Hz, 2H), 7.57 – 7.41 (m, 2H), 7.37 (t, *J* = 8.4 Hz, 1H), 7.26 (d, *J* = 7.1 Hz, 3H), 7.18 (d, *J* = 7.5 Hz, 1H), 4.03 (s, 3H), 2.43 (s, 3H), 2.25 (s, 3H), 1.41 (s, 3H) ppm; **^{13}C NMR** (75 MHz, $CDCl_3$) δ 155.21, 151.36, 148.23, 138.80, 136.23, 133.42, 132.29, 131.77, 131.05, 130.81, 129.88, 128.94, 128.71, 128.56, 127.72, 127.58, 126.71, 126.38, 126.17, 124.47, 124.36, 124.25, 122.89, 122.79, 117.35, 112.84, 57.02, 29.72, 23.04, 21.48 ppm; **HRMS** (ESI): $C_{30}H_{26}N_3O_3$ $[M+H]^+$ calcd: 476.1969, found: 476.1959.

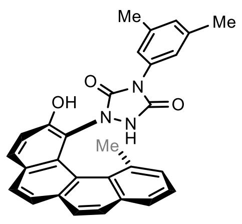

3m

Prepared according to the general procedure on a 0.2 mmol scale and purified by flash chromatography (petroleum ether/ethyl acetate = 4/1 to 2/1). White solid, 39.1 mg, 42% yield, 95.5:4.5 er; **HPLC analysis**: Chiralcel AD-H (*n*-hexane/*i*-PrOH(1‰ TFA) = 7/3, flow rate = 1.0 mL/min), t_{major} = 4.57 min, t_{minor} = 5.37 min; **HRMS** (ESI): $C_{29}H_{23}N_3NaO_3$ $[M+Na]^+$ calcd: 484.1632, found: 484.1633.

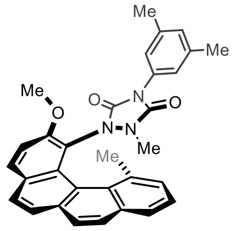

4m

White solid, m.p. 237 – 238 °C, 93% yield, 4.6:1 dr, 95.5:4.5 er; $[\alpha]_D^{24}$ = -508 (c = 1.0 in $CHCl_3$); **HPLC analysis**: Chiralcel IA-H (*n*-hexane/EtOH(1‰ TFA) = 7/3, flow rate = 1.0 mL/min), t_{major} = 6.19 min, t_{minor} = 7.71 min; **^{1}H NMR** (600 MHz, $CDCl_3$) δ 8.14 (d, *J* = 8.9 Hz, 1H), 7.87 (dd, *J* = 10.8, 8.3 Hz, 2H), 7.79 (d, *J* = 7.9 Hz, 1H), 7.71 (d, *J* = 8.3 Hz, 1H), 7.67 (d, *J* = 8.3 Hz, 1H), 7.56 – 7.39 (m, 2H), 7.33 – 7.21 (m, 1H), 7.05 (s, 2H), 7.00 (s, 1H), 4.03 (s, 3H), 2.39 (s, 6H), 2.25 (s, 3H), 1.41 (s, 3H) ppm; **^{13}C NMR** (151 MHz, $CDCl_3$) δ 155.22, 151.53, 148.43, 138.58, 136.30, 133.41, 132.29, 131.56, 131.03, 130.84, 129.96, 129.66, 128.93, 127.76, 127.59, 126.71, 126.16, 124.47, 124.36, 124.25, 123.68, 122.83, 117.53, 112.85, 57.05, 29.73, 23.06, 21.38 ppm; **HRMS** (ESI): $C_{33}H_{27}N_3NaO_3$ $[M+Na]^+$ calcd: 512.1945, found: 512.1952.

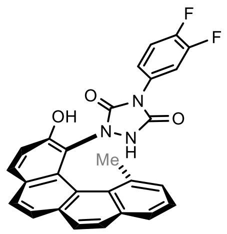

3n

Prepared according to the general procedure on a 0.2 mmol scale and purified by flash chromatography (petroleum ether/ethyl acetate = 4/1 to 2/1). White solid, 26.0 mg, 28% yield, 97:3 er; **HPLC analysis**: Chiralcel IA-H (*n*-hexane/*i*-PrOH(1‰ TFA) = 8/2, flow rate = 1.0 mL/min), t_{minor} = 13.72 min, t_{major} = 24.86 min; **HRMS** (ESI): $C_{27}H_{17}F_2N_3NaO_3$ $[M+Na]^+$ calcd: 492.1130, found: 492.1130.

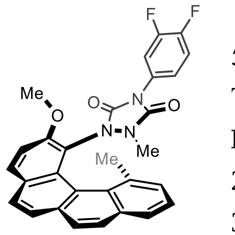

4n

White solid, m.p. 207 – 208 °C, 98% yield, 5.5:1 dr, 96.5:3.5 er; $[\alpha]_D^{24}$ = -555 (c = 1.0 in $CHCl_3$); **HPLC analysis**: Chiralcel IA-H (*n*-hexane/EtOH(1‰ TFA) = 7/3, flow rate = 1.0 mL/min), t_{major} = 8.46 min, t_{minor} = 10.74 min; **^{1}H NMR** (300 MHz, $CDCl_3$) δ 8.15 (d, *J* = 9.0 Hz, 1H), 7.88 (dd, *J* = 8.5, 3.2 Hz, 2H), 7.83 – 7.62 (m, 3H), 7.44 (t, *J* = 8.2 Hz, 3H), 7.38 – 7.04 (m, 3H), 4.03 (s, 3H), 2.21 (s, 3H), 1.41 (s, 3H) ppm; **^{13}C NMR** (75 MHz, $CDCl_3$) δ 155.25, 150.41, 147.13, 135.98, 133.58, 132.39, 131.34, 130.68, 129.49, 129.05, δ 128.28 (dd, J_{C-F} = 8.2, 3.8 Hz), 127.62, 127.60, 126.85, 126.10, 124.60, 124.38, 122.60, 121.60 (dd, J_{C-F} = 6.3, 4.1 Hz), 117.37, 117.13, 116.41, 115.19, 114.92, 112.76, 57.03, 29.64, 22.99 ppm; **^{19}F NMR** (282 MHz, $CDCl_3$) δ -135.54 (d, *J* = 21.4 Hz), -138.28 (d, *J* = 21.7 Hz) ppm; **HRMS** (ESI): $C_{29}H_{22}F_2N_3O_3$ $[M+H]^+$ calcd: 498.1624, found: 498.1607.

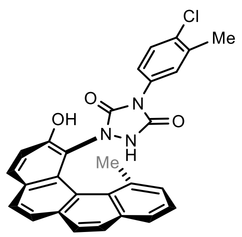

3o

Prepared according to the general procedure on a 0.2 mmol scale and purified by flash chromatography (petroleum ether/ethyl acetate = 4/1 to 2/1). White solid, 44.6 mg, 46% yield, 94.5:5.5 er; **HPLC analysis**: Chiralcel IA-H (*n*-hexane/*i*-PrOH(1‰ TFA) = 8/2, flow rate = 1.0 mL/min), t_{minor} = 14.74 min, t_{major} = 20.80 min; **HRMS** (ESI): $C_{28}H_{20}ClN_3NaO_3$ $[M+Na]^+$ calcd: 504.1085, found: 504.1075.

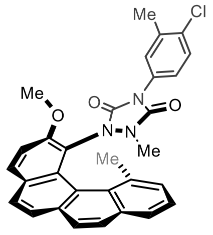

4o

White solid, m.p. 181 – 182 °C, 96% yield, 5:1 dr, 94.5:5.5 er; $[\alpha]_D^{24}$ = -388 (c = 1.0 in $CHCl_3$); **HPLC analysis**: Chiralcel IA-H (*n*-hexane/EtOH(1‰ TFA) = 7/3, flow rate = 1.0 mL/min), t_{major} = 8.36 min, t_{minor} = 12.05 min; **^{1}H NMR** (300 MHz, $CDCl_3$) δ 8.15 (d, *J* = 8.8 Hz, 1H), 7.88 (d, *J* = 8.4 Hz, 2H), 7.79 (d, *J* = 8.0 Hz, 1H), 7.76 – 7.61 (m, 2H), 7.46 (d, *J* = 8.0 Hz, 3H), 7.37 (s, 1H), 7.34 – 7.14 (m, 2H), 4.03 (s, 3H), 2.45 (s, 3H), 2.22 (s, 3H), 1.41 (s, 3H) ppm; **^{13}C NMR** (75 MHz, $CDCl_3$) δ 155.22, 150.86, 147.59, 136.80, 136.07, 133.49, 132.32, 131.16, 130.72, 130.32, 129.63, 129.41, 128.99, 127.86, 127.67, 127.57, 126.76, 126.09, 124.52, 124.47, 124.31, 124.28, 122.68, 116.84, 112.77, 57.02, 29.67, 22.99, 20.31 ppm; **HRMS** (ESI): $C_{30}H_{25}ClN_3O_3$ $[M+H]^+$ calcd: 510.1579, found: 510.1561.

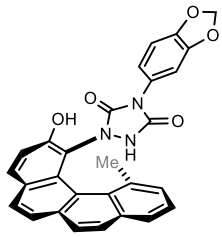

3p

Prepared according to the general procedure on a 0.2 mmol scale and purified by flash chromatography (petroleum ether/ethyl acetate = 4/1 to 2/1). White solid, 43.9 mg, 46% yield, 96:4 er; **HPLC analysis**: Chiralcel AD-H (*n*-hexane/*i*-PrOH(1‰ TFA) = 7/3, flow rate = 1.0 mL/min), t_{major} = 11.64 min, t_{minor} = 21.70 min; **HRMS** (ESI): $C_{28}H_{20}N_3O_5$ $[M+H]^+$ calcd: 478.1397, found: 478.1397.

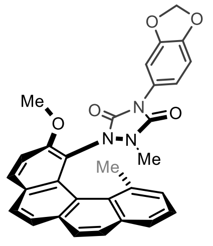

4p

White solid, m.p. 249 – 250 °C, 88% yield, 4.5:1 dr, 96.5:3.5 er; $[\alpha]_D^{24}$ = -480 (c = 1.0 in $CHCl_3$); **HPLC analysis**: Chiralcel IA-H (*n*-hexane/EtOH(1‰ TFA) = 7/3, flow rate = 1.0 mL/min), t_{major} = 15.47 min, t_{minor} = 29.39 min; **^{1}H NMR** (300 MHz, $CDCl_3$) δ 8.13 (d, *J* = 8.9 Hz, 1H), 7.86 (t, *J* = 7.0 Hz, 2H), 7.78 (d, *J* = 7.9 Hz, 1H), 7.74 – 7.58 (m, 2H), 7.57 – 7.34 (m, 2H), 7.26 (d, *J* = 7.9 Hz, 1H), 6.92 (d, *J* = 7.0 Hz, 3H), 6.01 (s, 2H), 4.01 (s, 3H), 2.24 (s, 3H), 1.40 (s, 3H) ppm; **^{13}C NMR** (75 MHz, $CDCl_3$) δ 155.23, 151.37, 148.28, 147.85, 147.18, 136.31, 133.48, 132.35, 131.16, 130.82, 129.92, 128.98, 127.68, 127.61, 126.78, 126.19, 125.47, 124.56, 124.46, 124.31, 122.79, 119.90, 117.20, 112.87, 108.27, 107.50, 101.67, 57.04, 29.73, 23.09 ppm; **HRMS** (ESI): $C_{30}H_{24}N_3O_3$ $[M+H]^+$ calcd: 506.1710, found: 506.1692.

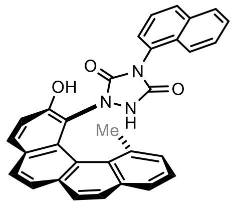

3q

Prepared according to the general procedure on a 0.2 mmol scale and purified by flash chromatography (petroleum ether/ethyl acetate = 4/1 to 2/1). White solid, 40.4 mg, 42% yield, 99:1 er; **HPLC analysis**: Chiralcel AD-H (*n*-hexane/*i*-PrOH(1‰ TFA) = 7/3, flow rate = 1.0 mL/min), t_{major} = 5.43 min, t_{minor} = 17.58 min; **HRMS** (ESI): $C_{31}H_{22}N_3O_3$ $[M+H]^+$ calcd: 484.1656, found: 484.1651.

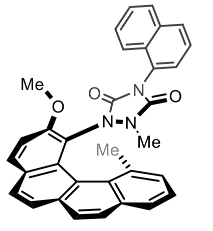

4q

White solid, m.p. 277 – 278 °C, 93% yield, 6.5:1 dr, 99:1 er; $[\alpha]_D^{24}$ = -323 (c = 1.0 in $CHCl_3$); **HPLC analysis**: Chiralcel IA-H (*n*-hexane/*i*-PrOH(1‰ TFA) = 7/3, flow rate = 1.0 mL/min), t_{major} = 10.64 min, t_{minor} = 16.07 min; **^{1}H NMR** (600 MHz, $CDCl_3$, two rotamers) δ 8.15 (d, *J* = 8.9 Hz, 1H), 8.04 – 7.77 (m, 5H), 7.75 – 7.67 (m, 2H), 7.66 – 7.42 (m, 5H), 7.41 – 7.20 (m, 2H), 4.05 (d, *J* = 13.9 Hz, 3H), 2.35 (d, *J* = 18.3 Hz, 3H), 1.46 (d, *J* = 9.6 Hz, 3H) ppm; **^{13}C NMR** (151 MHz, $CDCl_3$, two rotamers) δ 155.10, 155.04, 152.01, 151.96, 151.71, 150.20, 138.08, 137.55, 134.53, 134.50, 133.48, 133.45, 132.48, 132.38, 132.10, 131.59, 131.47, 131.41, 131.38, 131.16, 130.37, 130.21, 129.85, 128.82, 128.79, 128.43, 128.30, 127.96, 127.88,

127.81, 127.77, 127.74, 127.41, 126.89, 126.85, 126.79, 126.77, 126.65, 126.53, 126.36, 126.30, 126.27, 125.53, 125.33, 124.88, 124.72, 124.35, 124.33, 124.31, 124.24, 124.12, 123.21, 123.10, 123.07, 122.48, 119.46, 118.42, 113.06, 57.04, 56.92, 29.51, 29.44, 23.56, 23.40 ppm; **HRMS** (ESI): $C_{33}H_{25}N_3NaO_3$ [M+Na]$^+$ calcd: 534.1788, found: 534.1793.

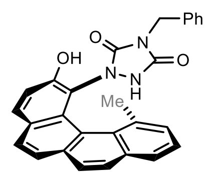

3r

Prepared according to the general procedure on a 0.2 mmol scale and purified by flash chromatography (petroleum ether/ethyl acetate = 7/1 to 4/1). White solid, 34.2 mg, 38% yield, 96:4 er; **HPLC analysis**: Chiralcel AD-H (*n*-hexane/*i*-PrOH(1‰ TFA) = 7/3, flow rate = 1.0 mL/min), t_{major} = 7.34 min, t_{minor} = 17.47 min; **HRMS** (ESI): $C_{28}H_{21}N_3NaO_3$ [M+Na]$^+$ calcd: 470.1475, found: 470.1470.

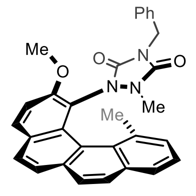

4r

White solid, m.p. 216 – 217 °C, 97% yield, 6.4:1 dr, 96:4 er; $[\alpha]_D^{24}$ = -579 (c = 1.0 in $CHCl_3$); **HPLC analysis**: Chiralcel IA-H (*n*-hexane/EtOH(1‰ TFA) = 7/3, flow rate = 1.0 mL/min), t_{major} = 7.41 min, t_{minor} = 14.43 min; **^{1}H NMR** (600 MHz, $CDCl_3$) δ 8.08 (dd, *J* = 9.0, 2.8 Hz, 1H), 7.82 (dd, *J* = 7.2, 4.2 Hz, 2H), 7.64 (ddd, *J* = 22.9, 8.4, 2.6 Hz, 3H), 7.46 (d, *J* = 6.0 Hz, 2H), 7.44 – 7.30 (m, 4H), 7.25 (td, *J* = 7.4, 2.6 Hz, 1H), 7.02 (d, *J* = 5.5 Hz, 1H), 4.88 – 4.46 (m, 2H), 3.73 (s, 3H), 2.27 (s, 3H), 1.31 (s, 3H) ppm; **^{13}C NMR** (151 MHz, $CDCl_3$) δ 154.95, 152.57, 151.20, 136.74, 136.10, 133.30, 132.16, 131.14, 131.00, 130.98, 129.01, 128.74, 128.49, 127.78, 127.61, 127.51, 126.65, 126.24, 124.52, 124.17, 123.90, 122.97, 118.77, 112.79, 56.59, 42.89, 29.43, 23.27 ppm; **HRMS** (ESI): $C_{30}H_{25}N_3NaO_3$ [M+Na]$^+$ calcd: 498.1788, found: 498.1792.

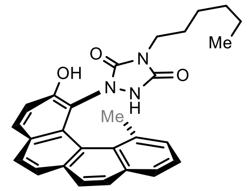

3s

Prepared according to the general procedure on a 0.2 mmol scale and purified by flash chromatography (petroleum ether/ethyl acetate = 7/1 to 4/1). White solid, 38.5 mg, 44% yield, 96:4 er; **HPLC analysis**: Chiralcel AD-H (*n*-hexane/*i*-PrOH(1‰ TFA) = 7/3, flow rate = 1.0 mL/min), t_{major} = 4.77 min, t_{minor} = 6.82 min; **HRMS** (ESI): $C_{27}H_{28}N_3O_3$ [M+H]$^+$ calcd: 442.2125, found: 442.2124.

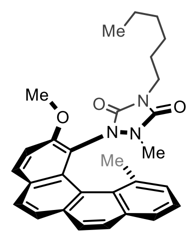

4s

White solid, m.p. 118 – 119 °C, 92% yield, 5.4:1 dr, 96:4 er; $[\alpha]_D^{24}$ = -607 (c = 1.0 in $CHCl_3$); **HPLC analysis**: Chiralcel AS-H (*n*-hexane/EtOH(1‰ TFA) = 7/3, flow rate = 1.0 mL/min), t_{minor} = 4.26 min, t_{major} = 5.13 min; **^{1}H NMR** (300 MHz, $CDCl_3$) δ 8.11 (d, *J* = 9.0 Hz, 1H), 7.84 (d, *J* = 8.4 Hz, 2H), 7.78 – 7.56 (m, 3H), 7.53 – 7.31 (m, 2H), 7.25 (d, *J* = 7.2 Hz, 1H), 3.95 (s, 3H), 3.45 (dt, *J* = 9.5, 6.8 Hz, 2H), 2.28 (s, 3H), 1.77 – 1.50 (m, 2H), 1.35 (s, 6H), 1.31 (s, 3H), 0.93 (t, *J* = 5.7 Hz, 1H) ppm; **^{13}C NMR** (75 MHz, $CDCl_3$) δ 155.09, 152.99, 150.85, 136.69, 133.32, 132.21, 131.04, 130.77, 128.79, 127.64, 127.55, 126.67, 126.12, 124.53, 124.19, 124.13, 122.98, 118.51, 112.89, 56.86, 39.51, 31.54, 29.55, 28.00, 26.35, 23.30, 22.63, 14.15 ppm; **HRMS** (ESI): $C_{29}H_{32}N_3O_3$ [M+H]$^+$ calcd: 470.2438, found: 470.2432.

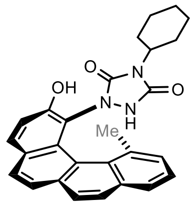

3t

Prepared according to the general procedure on a 0.2 mmol scale and purified by flash chromatography (petroleum ether/ethyl acetate = 7/1 to 4/1). White solid, 37.9 mg, 43% yield, 98:2 er; **HPLC analysis**: Chiralcel AD-H (*n*-hexane/*i*-PrOH(1‰ TFA) = 7/3, flow rate = 1.0 mL/min), t_{major} = 3.95 min, t_{minor} = 6.88 min; **HRMS** (ESI): $C_{27}H_{25}N_3NaO_3$ $[M+Na]^+$ calcd: 462.1788, found: 462.1786.

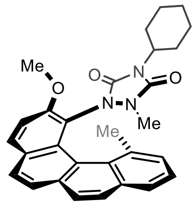

4t

White solid, m.p. 311 – 312 °C, 88% yield, 5.4:1 dr, 98:2 er; $[\alpha]_D^{24}$ = -629 (c = 1.0 in $CHCl_3$); **HPLC analysis**: Chiralcel IA-H (*n*-hexane/EtOH(1‰ TFA) = 19/1, flow rate = 1.0 mL/min), t_{major} = 12.12 min, t_{minor} = 17.18 min; **^{1}H NMR** (600 MHz, $CDCl_3$) δ 8.10 (d, *J* = 8.9 Hz, 1H), 7.82 (d, *J* = 8.3 Hz, 2H), 7.76 – 7.59 (m, 3H), 7.50 – 7.34 (m, 2H), 7.26 (d, *J* = 4.7 Hz, 1H), 3.94 (s, 3H), 3.85 (tt, *J* = 12.4, 4.0 Hz, 1H), 2.30 (s, 3H), 2.25 – 2.07 (m, 2H), 1.96 – 1.79 (m, 2H), 1.79 – 1.60 (m, 3H), 1.42 – 1.28 (m, 3H), 1.27 (s, 3H) ppm; **^{13}C NMR** (151 MHz, $CDCl_3$) δ 154.96, 152.88, 150.82, 136.98, 133.27, 132.17, 131.11, 131.09, 130.99, 128.70, 127.68, 127.54, 126.64, 126.17, 124.52, 124.15, 123.99, 123.06, 119.00, 112.98, 56.84, 51.67, 29.45, 29.25, 29.06, 25.87, 25.85, 25.07, 23.31 ppm; **HRMS** (ESI): $C_{29}H_{30}N_3O_3$ $[M+H]^+$ calcd: 468.2282, found: 468.2290.

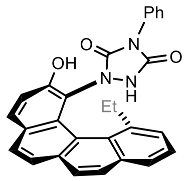

3u

Prepared according to the general procedure on a 0.2 mmol scale and purified by flash chromatography (petroleum ether/ethyl acetate = 4/1 to 2/1). White solid, 44.1 mg, 49% yield, 98:2 er; **HPLC analysis**: Chiralcel AD-H (*n*-hexane/*i*-PrOH(1‰ TFA) = 7/3, flow rate = 1.0 mL/min), t_{major} = 5.87 min, t_{minor} = 13.60 min; **HRMS** (ESI): $C_{28}H_{22}N_3O_3$ $[M+H]^+$ calcd: 448.1656, found: 448.1646.

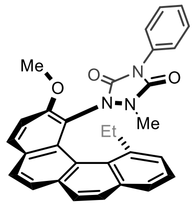

4u

White solid, m.p. 208 – 209 °C, 79% yield, 6:1 dr, 98:2 er; $[\alpha]_D^{24}$ = -558 (c = 1.0 in $CHCl_3$); **HPLC analysis**: Chiralcel AD-H (*n*-hexane/*i*-PrOH(1‰ TFA) = 7/3, flow rate = 1.0 mL/min), t_{major} = 6.75 min, t_{minor} = 18.90 min; **^{1}H NMR** (300 MHz, $CDCl_3$) δ 8.14 (d, *J* = 8.9 Hz, 1H), 7.87 (d, *J* = 8.3 Hz, 2H), 7.72 (dt, *J* = 23.8, 8.1 Hz, 3H), 7.60 – 7.33 (m, 6H), 7.27 (d, *J* = 7.1 Hz, 2H), 4.03 (s, 3H), 2.98 (dt, *J* = 15.1, 7.6 Hz, 1H), 2.87 – 2.50 (m, 1H), 1.44 (s, 3H), 0.33 (t, *J* = 7.5 Hz, 3H) ppm; **^{13}C NMR** (75 MHz, $CDCl_3$) δ 155.00, 151.19, 148.09, 142.90, 133.26, 132.45, 132.06, 131.08, 129.99, 129.30, 129.02, 128.89, 127.62, 127.56, 126.68, 126.54, 125.58, 125.49, 124.58, 124.52, 124.37, 122.48, 117.14, 112.73, 56.93, 29.59, 28.88, 15.33 ppm; **HRMS** (ESI): $C_{30}H_{26}N_3O_3$ $[M+H]^+$ calcd: 476.1969, found: 476.1956.

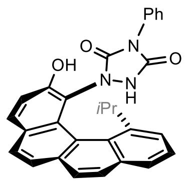

3v

Prepared according to the general procedure on a 0.2 mmol scale and purified by flash chromatography (petroleum ether/ethyl acetate = 4/1 to 2/1). White solid, 43.5 mg, 47% yield, 98.5:1.5 er; **HPLC analysis**: Chiralcel AD-H (*n*-hexane/*i*-PrOH(1‰ TFA) = 7/3, flow rate = 1.0 mL/min), t_{major} = 6.04 min, t_{minor} = 9.38 min; **HRMS** (ESI): $C_{29}H_{24}N_3O_3$ $[M+H]^+$ calcd: 462.1812, found: 462.1806.

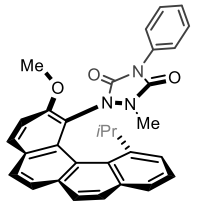

4v

White solid, m.p. 212 – 213 °C, 94% yield, 4.6:1 dr, 98.5:1.5 er; $[\alpha]_D^{24}$ = -706 (c = 1.0 in $CHCl_3$); **HPLC analysis**: Chiralcel AD-H (*n*-hexane/*i*-PrOH(1‰ TFA) = 7/3, flow rate = 1.0 mL/min), t_{major} = 5.36 min, t_{minor} = 18.04 min; **^{1}H NMR** (300 MHz, $CDCl_3$) δ 8.11 (d, *J* = 8.9 Hz, 1H), 7.85 (d, *J* = 8.3 Hz, 2H), 7.80 – 7.58 (m, 3H), 7.57 – 7.04 (m, 8H), 3.99 (s, 3H), 3.68 (p, *J* = 6.7 Hz, 1H), 1.45 (s, 3H), 1.20 (d, *J* = 6.4 Hz, 2H), -0.14 (d, *J* = 6.7 Hz, 2H) ppm; **^{13}C NMR** (75 MHz, $CDCl_3$) δ 154.80, 151.31, 148.31, 146.14, 132.78, 132.56, 132.16, 130.89, 129.88, 129.39, 129.09, 128.92, 127.76, 127.56, 126.62, 126.30, 125.55, 124.71, 124.51, 124.47, 123.00, 121.43, 117.53, 112.78, 56.95, 30.31, 29.59, 24.07, 20.83 ppm; **HRMS** (ESI): $C_{31}H_{28}N_3O_3$ $[M+H]^+$ calcd: 490.2125, found: 490.2131.

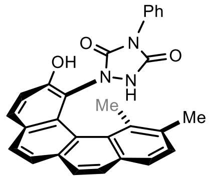

3w

Prepared according to the general procedure on a 0.2 mmol scale and purified by flash chromatography (petroleum ether/ethyl acetate = 4/1 to 2/1). White solid, 43.4 mg, 49% yield, 92.5:7.5 er; **HPLC analysis**: Chiralcel AD-H (*n*-hexane/*i*-PrOH(1‰ TFA) = 7/3, flow rate = 1.0 mL/min), t_{major} = 7.26 min, t_{minor} = 11.67 min; **HRMS** (ESI): $C_{28}H_{21}N_3NaO_3$ $[M+Na]^+$ calcd: 470.1475, found: 470.1473.

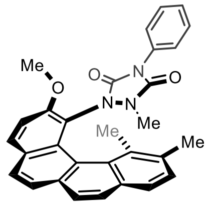

4w

White solid, m.p. 225 – 226 °C, 95% yield, 18:1 dr, 92.5:7.5 er; $[\alpha]_D^{24}$ = -730 (c = 1.0 in $CHCl_3$); **HPLC analysis**: Chiralcel IA-H (*n*-hexane/EtOH(1‰ TFA) = 7/3, flow rate = 1.0 mL/min), t_{major} = 8.04 min, t_{minor} = 13.77 min; **^{1}H NMR** (300 MHz, $CDCl_3$) δ 8.11 (d, *J* = 8.9 Hz, 1H), 7.84 (d, *J* = 8.3 Hz, 2H), 7.78 – 7.66 (m, 2H), 7.61 (d, *J* = 8.3 Hz, 1H), 7.56 – 7.25 (m, 7H), 4.04 (s, 3H), 2.14 (s, 3H), 2.05 (s, 3H), 1.39 (s, 3H) ppm; **^{13}C NMR** (75 MHz, $CDCl_3$) δ 155.38, 151.14, 147.55, 135.23, 134.11, 133.56, 132.09, 131.02, 130.87, 129.92, 129.07, 128.83, 128.78, 127.44, 127.41, 126.54, 125.22, 124.38, 123.96, 123.61, 122.52, 116.68, 112.70, 57.02, 29.79, 20.24, 19.65 ppm; **HRMS** (ESI): $C_{30}H_{26}N_3O_3$ $[M+H]^+$ calcd: 476.1969, found: 476.1958.

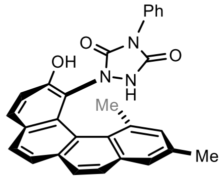

3x

Prepared according to the general procedure on a 0.2 mmol scale and purified by flash chromatography (petroleum ether/ethyl acetate = 4/1 to 2/1). White solid, 44.0 mg, 49% yield, 85:15 er; **HPLC analysis**: Chiralcel AD-H (*n*-hexane/*i*-PrOH(1‰ TFA) = 7/3, flow rate = 1.0 mL/min), t_{major} = 5.72 min, t_{minor} = 14.10 min; **HRMS** (ESI): $C_{28}H_{21}N_3NaO_3$ $[M+Na]^+$ calcd: 470.1475, found: 470.1472.

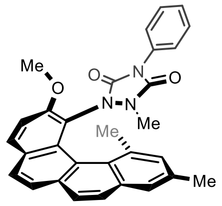

4x

White solid, m.p. 197 – 198 °C, 86% yield, 4.9:1 dr, 85:15 er; $[\alpha]_D^{24}$ = -326 (c = 1.0 in $CHCl_3$); **HPLC analysis**: Chiralcel AD-H (*n*-hexane/EtOH(1‰ TFA) = 7/3, flow rate = 1.0 mL/min), t_{major} = 8.13 min, t_{minor} = 16.48 min; **^{1}H NMR** (300 MHz, $CDCl_3$) δ 8.10 (d, *J* = 8.9 Hz, 1H), 7.80 (d, *J* = 7.5 Hz, 2H), 7.65 (dd, *J* = 15.7, 8.3 Hz, 2H), 7.59 – 7.28 (m, 7H), 7.09 (s, 1H), 4.01 (s, 3H), 2.44 (s, 3H), 2.22 (s, 3H), 1.42 (s, 3H) ppm; **^{13}C NMR** (75 MHz, $CDCl_3$) δ 155.14, 151.14, 147.98, 136.06, 135.80, 132.98, 132.61, 132.01, 131.00, 129.75, 129.05, 128.84, 128.66, 127.60, 127.54, 126.20, 125.50, 124.47, 124.31, 123.92, 122.92, 117.13, 112.71, 56.98, 29.63, 22.93, 21.30 ppm; **HRMS** (ESI): $C_{30}H_{26}N_3O_3$ $[M+H]^+$ calcd: 476.1969, found: 476.1970.

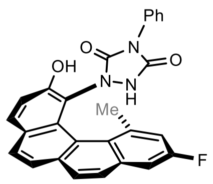

3y

Prepared according to the general procedure on a 0.2 mmol scale and purified by flash chromatography (petroleum ether/ethyl acetate = 4/1 to 2/1). White solid, 40.9 mg, 45% yield, 92:8 er; **HPLC analysis**: Chiralcel AD-H (*n*-hexane/*i*-PrOH(1‰ TFA) = 7/3, flow rate = 1.0 mL/min), t_{major} = 6.80 min, t_{minor} = 12.37 min; **HRMS** (ESI): $C_{27}H_{18}FN_3NaO_3$ $[M+Na]^+$ calcd: 474.1224, found: 474.1227.

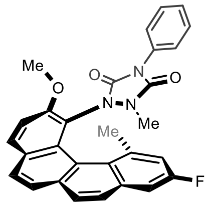

4y

White solid, m.p. 237 – 238 °C, 93% yield, 5.3:1 dr, 92:8 er; $[\alpha]_D^{24}$ = -460 (c = 1.0 in $CHCl_3$); **HPLC analysis**: Chiralcel AD-H (*n*-hexane/EtOH(1‰ TFA) = 7/3, flow rate = 1.0 mL/min), t_{major} = 9.43 min, t_{minor} = 16.10 min; **^{1}H NMR** (300 MHz, $CDCl_3$) δ 8.13 (d, *J* = 1.3 Hz, 1H), 7.84 (dd, *J* = 14.3, 7.9 Hz, 2H), 7.71 (d, *J* = 8.9 Hz, 2H), 7.57 – 7.30 (m, 7H), 7.02 (d, *J* = 9.2 Hz, 1H), 4.04 (s, 3H), 2.25 (s, 3H), 1.44 (s, 3H) ppm; **^{13}C NMR** (75 MHz, $CDCl_3$) δ 155.22, 151.38, 148.46, 139.44 ($J_{C\text{-}F}$ = 8.2 Hz), 133.27, 133.15, 132.90, 131.74, 131.22, 129.70, 129.03, 128.34 ($J_{C\text{-}F}$ = 4.4 Hz), 127.82, 127.74, 126.64, 125.83, 125.78, 124.22, 122.89, 117.38, 116.66, 116.35, 113.09, 108.18 ($J_{C\text{-}F}$ = 20.6 Hz), 57.04, 29.78, 23.19 ($J_{C\text{-}F}$ = 1.7 Hz) ppm; **^{19}F NMR** (282 MHz, $CDCl_3$) δ -115.37 ppm; **HRMS** (ESI): $C_{29}H_{23}FN_3O_3$ $[M+H]^+$ calcd: 480.1718, found: 480.1696.

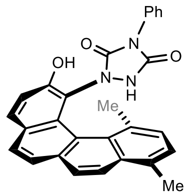

3z

Prepared according to the general procedure on a 0.2 mmol scale and purified by flash chromatography (petroleum ether/ethyl acetate = 4/1 to 2/1). White solid, 43.5 mg, 49% yield, 95:5 er; **HPLC analysis**: Chiralcel AD-H (*n*-hexane/*i*-PrOH(1‰ TFA) = 7/3, flow rate = 1.0 mL/min), t_{major} = 5.54 min, t_{minor} = 9.49 min; **HRMS** (ESI): $C_{28}H_{21}N_3NaO_3$ $[M+Na]^+$ calcd: 470.1475, found: 470.1476.

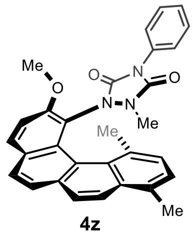

4z

White solid, m.p. 227 – 228 °C, 88% yield, 2.7:1 dr, 95:5 er; $[\alpha]_D^{24}$ = -465 (c = 1.0 in $CHCl_3$); **HPLC analysis**: Chiralcel IA-H (*n*-hexane/*i*-PrOH(1‰ TFA) = 7/3, flow rate = 1.0 mL/min), t_{major} = 6.32 min, t_{minor} = 13.49 min; **^{1}H NMR** (300 MHz, $CDCl_3$) δ 8.10 (t, *J* = 9.4 Hz, 2H), 7.84 (d, *J* = 8.3 Hz, 1H), 7.71 (d, *J* = 8.4 Hz, 2H), 7.59 – 7.33 (m, 6H), 7.29 (d, *J* = 7.1 Hz, 1H), 7.15 (d, *J* = 7.5 Hz, 1H), 4.01 (s, 3H), 2.78 (s, 3H), 2.21 (s, 3H), 1.40 (s, 3H) ppm; **^{13}C NMR** (75 MHz, $CDCl_3$) δ 155.20, 151.34, 148.49, 134.26, 133.07, 132.08, 131.38, 131.18, 131.12, 130.45, 130.31, 128.95, 127.66, 127.59, 127.37, 127.32, 126.70, 125.74, 125.40, 125.01, 124.28, 124.21, 123.19, 117.62, 112.83, 57.03, 29.69, 23.13, 19.49 ppm; **HRMS** (ESI): $C_{30}H_{26}N_3O_3$ $[M+H]^+$ calcd: 476.1969, found: 476.1957.

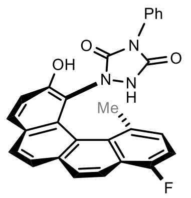

3aa

Prepared according to the general procedure on a 0.2 mmol scale and purified by flash chromatography (petroleum ether/ethyl acetate = 4/1 to 2/1). White solid, 43.6 mg, 48% yield, 95.5:4.5 er; **HPLC analysis**: Chiralcel AD-H (*n*-hexane/*i*-PrOH(1‰ TFA) = 7/3, flow rate = 1.0 mL/min), t_{major} = 6.48 min, t_{minor} = 7.54 min; **HRMS** (ESI): $C_{27}H_{18}FN_3NaO_3$ $[M+Na]^+$ calcd: 474.1224, found: 474.1223.

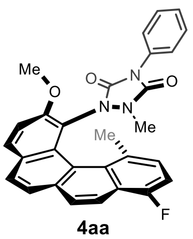

4aa

White solid, m.p. 243 – 244 °C, 92% yield, 6.3:1 dr, 96:4 er; $[\alpha]_D^{24}$ = -564 (c = 1.0 in $CHCl_3$); **HPLC analysis**: Chiralcel IC-H (*n*-hexane/*i*-PrOH(1‰ TFA) = 7/3, flow rate = 1.0 mL/min), t_{minor} = 11.39 min, t_{major} = 20.10 min; **^{1}H NMR** (300 MHz, $CDCl_3$) δ 8.17 (dd, *J* = 12.8, 8.7 Hz, 2H), 7.89 (d, *J* = 8.3 Hz, 1H), 7.73 (t, *J* = 7.6 Hz, 2H), 7.62 – 7.29 (m, 6H), 7.16 (d, *J* = 7.9 Hz, 2H), 4.02 (s, 3H), 2.20 (s, 3H), 1.44 (s, 3H) ppm; **^{13}C NMR** (75 MHz, $CDCl_3$) δ 155.31, 151.37, 148.44, 133.75, 132.07 (J_{C-F} = 3.8 Hz), 131.96 (J_{C-F} = 3.3 Hz), 131.90, 131.16, 129.84, 128.95, 127.76, 127.70, 127.63, 127.53, 127.35, 125.59, 125.16, 124.18, 122.52 (J_{C-F} = 2.5 Hz), 121.81 (J_{C-F} = 15.6 Hz), 121.05 (J_{C-F} = 6.5 Hz), 117.55, 113.11, 110.40 (J_{C-F} = 19.6 Hz), 57.00, 29.86, 22.76 ppm; **^{19}F NMR** (282 MHz, $CDCl_3$) δ -127.42 ppm; **HRMS** (ESI): $C_{29}H_{23}FN_3O_3$ $[M+H]^+$ calcd: 480.1718, found: 480.1707.

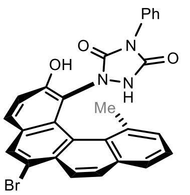

3ab

Prepared according to the general procedure on a 0.2 mmol scale and purified by flash chromatography (petroleum ether/ethyl acetate = 4/1 to 2/1). White solid, 49.6 mg, 49% yield, 98:2 er; **HPLC analysis**: Chiralcel AD-H (*n*-hexane/*i*-PrOH(1‰ TFA) = 7/3, flow rate = 1.0 mL/min), t_{major} = 6.60 min, t_{minor} = 12.33 min; **HRMS** (ESI): $C_{27}H_{19}BrN_3O_3$ $[M+H]^+$ calcd: 512.0604, found: 512.0596.

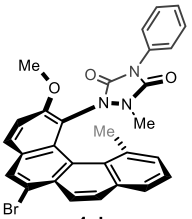

4ab

White solid, m.p. 226 – 227 °C, 86% yield, 3.3:1 dr, 98:2 er; $[\alpha]_D^{24}$ = -367 (c = 1.0 in $CHCl_3$); **HPLC analysis**: Chiralcel IC-H (*n*-hexane/EtOH(1‰ TFA) = 7/3, flow rate = 1.0 mL/min), t_{minor} = 8.12 min, t_{major} = 11.10 min; **^{1}H NMR** (300 MHz, $CDCl_3$) δ 8.28 – 7.91 (m, 4H), 7.81 (d, *J* = 7.9 Hz, 1H), 7.61 – 7.32 (m, 7H), 7.31 – 7.08 (m, 1H), 4.03 (s, 3H), 2.20 (s, 3H), 1.44 (s, 3H) ppm; **^{13}C NMR** (75 MHz, $CDCl_3$) δ 155.41, 151.26, 147.92, 136.36, 132.34, 131.97, 131.84, 130.16, 129.81, 129.55, 128.94, 128.91, 128.29, 127.67, 127.42, 126.88, 125.55, 125.29, 124.61, 124.39, 123.32, 118.77, 117.34, 113.61, 57.04, 29.93, 23.05 ppm; **HRMS** (ESI): $C_{29}H_{23}BrN_3O_3$ $[M+H]^+$ calcd: 540.0917, found: 540.0894.

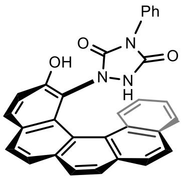

3ac

Prepared according to the general procedure on a 0.2 mmol scale and purified by flash chromatography (petroleum ether/ethyl acetate = 4/1 to 2/1). Yellow solid, 41.3 mg, 44% yield, 88:12 er; **HPLC analysis**: Chiralcel IA-H (*n*-hexane/*i*-PrOH(1‰ TFA) = 8/2, flow rate = 1.0 mL/min), t_{major} = 22.20 min, t_{minor} = 26.38 min; **HRMS** (ESI): $C_{30}H_{19}N_3NaO_3$ $[M+Na]^+$ calcd: 492.1319, found: 492.1314.

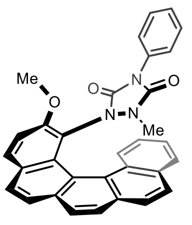

4ac

Yellow solid, m.p. 220 – 221 °C, 85% yield, 5:1 dr, 88.5:11.5 er; $[\alpha]_D^{24}$ = -1251 (c = 1.0 in $CHCl_3$); **HPLC analysis**: Chiralcel AD-H (*n*-hexane/*i*-PrOH(1‰ TFA) = 7/3, flow rate = 1.0 mL/min), t_{major} = 10.19 min, t_{minor} = 43.32 min; **^{1}H NMR** (300 MHz, $CDCl_3$) δ 8.13 (d, *J* = 8.9 Hz, 1H), 7.94 (t, *J* = 8.7 Hz, 2H), 7.82 (q, *J* = 8.8, 8.2 Hz, 4H), 7.70 (dd, *J* = 15.2, 8.0 Hz, 2H), 7.40 (t, *J* = 8.8 Hz, 4H), 7.27 (t, *J* = 8.6 Hz, 4H), 3.91 (s, 3H), 1.52 (s, 3H) ppm; **^{13}C NMR** (75 MHz, $CDCl_3$) δ 155.45, 151.61, 146.08, 133.77, 131.92, 130.95, 130.72, 130.42, 130.29, 130.20, 128.89, 128.55, 128.40, 128.18, 127.64, 127.44, 127.22, 127.14, 126.84, 126.47, 126.31, 125.64, 125.15, 124.94, 124.66, 121.72, 117.14, 113.44, 56.83, 29.94 ppm; **HRMS** (ESI): $C_{32}H_{24}N_3O_3$ $[M+H]^+$ calcd: 498.1812, found: 498.1798.

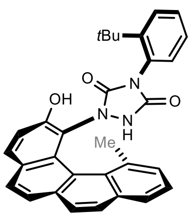

3ad

Prepared according to the general procedure on a 0.2 mmol scale and purified by flash chromatography (petroleum ether/ethyl acetate = 7/1 to 4/1). White solid, 47.9 mg, 49% yield, 97.5:2.5 er; **HPLC analysis**: Chiralcel AD-H (*n*-hexane/*i*-PrOH(1‰ TFA) = 7/3, flow rate = 1.0 mL/min), t_{major} = 3.85 min, t_{minor} = 5.90 min; **HRMS** (ESI): $C_{31}H_{28}N_3O_3$ $[M+H]^+$ calcd: 490.2125, found: 490.2122.

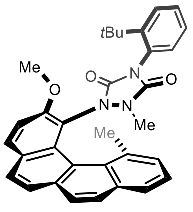

4ad

White solid, m.p. 321 – 322 °C, 95% yield, 6:1 dr, 97.5:2.5 er; $[\alpha]_D^{24}$ = -374 (c = 1.0 in $CHCl_3$); **HPLC analysis**: Chiralcel AD-H (*n*-hexane/*i*-PrOH(1‰ TFA) = 7/3, flow rate = 1.0 mL/min), t_{major} = 4.30 min, t_{minor} = 7.55 min; **^{1}H NMR** (300 MHz, $CDCl_3$) δ 8.13 (d, *J* = 8.9 Hz, 1H), 7.97 – 7.75 (m, 3H), 7.63 (dt, *J* = 28.4, 7.9 Hz, 4H), 7.50 – 7.27 (m, 4H), 6.82 (d, *J* = 7.5 Hz, 1H), 3.99 (s, 3H), 2.31 (s, 3H), 1.42 (s, 9H), 1.36 (s, 3H) ppm; **^{13}C NMR** (75 MHz, $CDCl_3$) δ 155.06, 152.69, 149.44, 149.37, 137.35, 133.50, 132.37, 131.56, 131.46, 131.22, 130.03, 129.79, 128.91, 128.51, 127.85, 127.62, 127.36, 126.81, 126.06, 124.73, 124.55, 124.26, 123.12, 117.44, 112.77, 56.51, 35.77, 31.36, 29.46, 23.57 ppm; **HRMS** (ESI): $C_{33}H_{32}N_3O_3$ $[M+H]^+$ calcd: 518.2438, found: 518.2421.

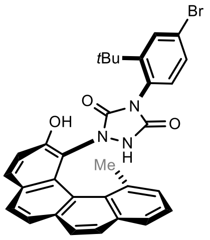

3ae

Prepared according to the general procedure on a 0.2 mmol scale and purified by flash chromatography (petroleum ether/ethyl acetate = 7/1 to 2/1). White solid, 54.1 mg, 48% yield, 97.5:2.5 er; **HPLC analysis**: Chiralcel AD-H (*n*-hexane/*i*-PrOH(1‰ TFA) = 7/3, flow rate = 1.0 mL/min), t_{major} = 3.64 min, t_{minor} = 5.42 min; **HRMS** (ESI): $C_{31}H_{27}BrN_3O_3$ $[M+H]^+$ calcd: 568.1230, found: 568.1215.

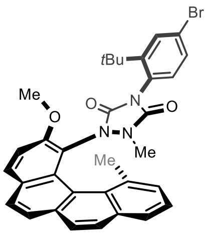

4ae

White solid, m.p. 269 – 270 °C, 96% yield, 6.6:1 dr, 97.5:2.5 er; $[\alpha]_D^{24}$ = -293 (c = 1.0 in $CHCl_3$); **HPLC analysis**: Chiralcel IA-H (*n*-hexane/EtOH(1‰ TFA) = 7/3, flow rate = 1.0 mL/min), t_{major} = 5.48 min, t_{minor} = 8.87 min; **^{1}H NMR** (300 MHz, $CDCl_3$) δ 8.13 (d, *J* = 9.0 Hz, 1H), 7.83 (dt, *J* = 14.1, 8.1 Hz, 3H), 7.68 (t, *J* = 7.9 Hz, 3H), 7.57 (t, *J* = 7.5 Hz, 1H), 7.49 (d, *J* = 8.3 Hz, 1H), 7.43 (d, *J* = 8.9 Hz, 1H), 7.33 (d, *J* = 7.1 Hz, 1H), 6.70 (d, *J* = 8.3 Hz, 1H), 3.98 (s, 3H), 2.29 (s, 3H), 1.40 (s, 9H), 1.35 (s, 3H) ppm; **^{13}C NMR** (75 MHz, $CDCl_3$) δ 155.03, 152.13, 151.87, 148.89, 137.35, 133.57, 133.19, 132.43, 131.91, 131.61, 131.30, 131.15, 130.57, 129.23, 128.95, 127.74, 127.63, 126.87, 125.94, 124.82, 124.67, 124.32, 124.17, 123.01, 117.04, 112.70, 56.51, 36.01, 31.15, 29.38, 23.54 ppm; **HRMS** (ESI): $C_{33}H_{31}BrN_3O_3$ $[M+H]^+$ calcd: 596.1543, found: 596.1523.

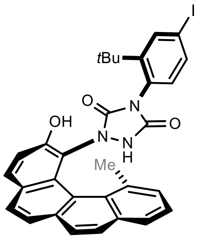

3af

Prepared according to the general procedure on a 0.2 mmol scale and purified by flash chromatography (petroleum ether/ethyl acetate = 7/1 to 2/1). White solid, 59.3 mg, 48% yield, 96.5:3.5 er; **HPLC analysis**: Chiralcel AD-H (*n*-hexane/*i*-PrOH(1‰ TFA) = 7/3, flow rate = 1.0 mL/min), t_{major} = 3.89 min, t_{minor} = 5.75 min; **HRMS** (ESI): $C_{31}H_{27}IN_3O_3$ $[M+H]^+$ calcd: 616.1092, found: 616.1079.

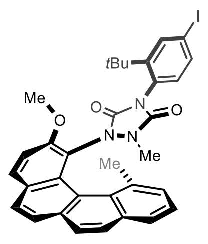

4af

White solid, m.p. 249 – 250 °C, 97% yield, 7.2:1 dr, 96.5:3.5 er; $[\alpha]_D^{24}$ = -226 (c = 1.0 in $CHCl_3$); **HPLC analysis**: Chiralcel IA-H (*n*-hexane/EtOH(1‰ TFA) = 7/3, flow rate = 1.0 mL/min), t_{major} = 5.43 min, t_{minor} = 8.85 min; **^{1}H NMR** (300 MHz, $CDCl_3$) δ 8.14 (d, *J* = 8.9 Hz, 1H), 7.99 – 7.75 (m, 4H), 7.68 (t, *J* = 8.2 Hz, 3H), 7.57 (t, *J* = 7.5 Hz, 1H), 7.43 (d, *J* = 8.9 Hz, 1H), 7.32 (d, *J* = 7.1 Hz, 1H), 6.55 (d, *J* = 8.2 Hz, 1H), 3.98 (s, 3H), 2.29 (s, 3H), 1.39 (s, 9H), 1.35 (s, 3H) ppm; **^{13}C NMR** (75 MHz, $CDCl_3$) δ 155.02, 152.10, 151.95, 148.87, 137.96, 137.34, 136.62, 133.56, 133.33, 132.42, 131.60, 131.30, 131.15, 130.02, 128.95, 127.74, 127.62, 126.86, 125.94, 124.81, 124.66, 124.32, 123.01, 117.05, 112.70, 96.55, 56.52, 35.87, 31.17, 29.39, 23.54 ppm; **HRMS** (ESI): $C_{33}H_{31}IN_3O_3$ $[M+H]^+$ calcd: 644.1405, found: 644.1377.

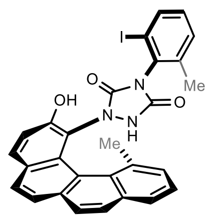

3ag

Prepared according to the general procedure on a 0.2 mmol scale and purified by flash chromatography (petroleum ether/ethyl acetate = 7/1 to 2/1). White solid, 55.0 mg, 48% yield, 98.5:1.5 er; **HPLC analysis**: Chiralcel IA-H (*n*-hexane/*i*-PrOH(1‰ TFA) = 7/3, flow rate = 1.0 mL/min), t_{major} = 6.12 min, t_{minor} = 13.72 min; **HRMS** (ESI): $C_{28}H_{20}IN_3NaO_3$ $[M+Na]^+$ calcd: 596.0442, found: 596.0440.

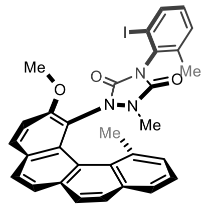

4ag

White solid, m.p. 284 – 285 °C, 95% yield, 4.5:1 dr, 98.5:1.5 er; $[\alpha]_D^{24}$ = -340 (c = 1.0 in $CHCl_3$); **HPLC analysis**: Chiralcel IA-H (*n*-hexane/EtOH(1‰ TFA) = 7/3, flow rate = 1.0 mL/min), t_{major} = 10.34 min, t_{minor} = 26.26 min; **^{1}H NMR** (300 MHz, Chloroform-*d*, two rotamers) δ 8.12 (d, *J* = 9.0 Hz, 1H), 7.97 – 7.07 (m, 10H), 6.88 (dd, *J* = 34.3, 8.3 Hz, 1H), 3.97 (s, 3H), 2.27 (s, 3H), δ 2.22 (d, *J* = 78.9 Hz, 3H), 1.38 (s, 3H) ppm; **^{13}C NMR** (75 MHz, $CDCl_3$, two rotamers) δ 155.11, 154.90, 151.47, 151.22, 151.01, 148.37, 140.12, 140.07, 139.36, 139.19, 137.93, 137.17, 136.13, 135.98, 133.56, 133.48, 132.41, 132.13, 131.73, 131.58, 131.38, 130.95, 130.91, 130.38, 130.31, 130.15, 128.90, 128.78, 127.83, 127.70, 127.64, 127.40, 126.86, 126.34, 126.17, 124.92, 124.79, 124.45, 124.37, 124.02, 123.15, 122.92, 119.58, 117.28, 113.03, 112.82, 95.27, 57.02, 56.86, 29.40, 23.62, 23.32, 17.70, 17.30 ppm; **HRMS** (ESI): $C_{30}H_{25}IN_3O_3$ $[M+H]^+$ calcd: 602.0935, found: 602.0899.

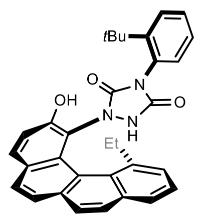

3ah

Prepared according to the general procedure on a 0.2 mmol scale and purified by flash chromatography (petroleum ether/ethyl acetate = 7/1 to 4/1). White solid, 44.9 mg, 45% yield, 97.5:2.5 er; **HPLC analysis**: Chiralcel IA-H (*n*-hexane/*i*-PrOH(1‰ TFA) = 7/3, flow rate = 1.0 mL/min), t_{major} = 3.71 min, t_{minor} = 5.30 min; **HRMS** (ESI): $C_{32}H_{30}N_3O_3$ $[M+H]^+$ calcd: 504.2282, found: 504.2276.

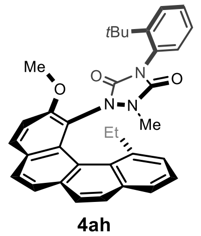

4ah

White solid, m.p. 222 – 223 °C, 91% yield, 6.2:1 dr, 97.5:2.5 er; $[\alpha]_D^{24}$ = -415 (c = 1.0 in $CHCl_3$); **HPLC analysis**: Chiralcel AD-H (*n*-hexane/*i*-PrOH(1‰ TFA) = 8/2, flow rate = 1.0 mL/min), t_{major} = 5.21 min, t_{minor} = 13.89 min; **^{1}H NMR** (300 MHz, Chloroform-*d*) δ 8.12 (d, *J* = 9.0 Hz, 1H), 7.83 (dt, *J* = 15.9, 7.9 Hz, 3H), 7.74 – 7.50 (m, 4H), 7.39 (q, *J* = 8.6, 8.0 Hz, 4H), 6.86 (d, *J* = 7.4 Hz, 1H), 3.98 (s, 3H), 3.08 (h, *J* = 7.5 Hz, 1H), 2.84 (dq, *J* = 15.1, 7.4 Hz, 1H), 1.42 (s, 9H), 1.38 (s, 3H) ppm; **^{13}C NMR** (75 MHz, $CDCl_3$) δ 154.84, 152.78, 149.53, 149.48,

144.01, 133.28, 132.52, 131.68, 131.42, 130.15, 129.79, 128.97, 128.46, 127.66, 127.33, 126.76, 126.39, 125.69, 124.82, 124.68, 124.34, 122.87, 117.52, 112.65, 56.42, 35.76, 31.34, 29.42, 29.37, 15.22 ppm; **HRMS** (ESI): $C_{34}H_{34}N_3O_3$ $[M+H]^+$ calcd: 532.2595, found: 532.2577.

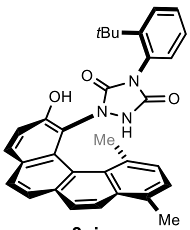

3ai

Prepared according to the general procedure on a 0.2 mmol scale and purified by flash chromatography (petroleum ether/ethyl acetate = 7/1 to 4/1). White solid, 46.0 mg, 46% yield, 97:3 er; **HPLC analysis**: Chiralcel IA-H (*n*-hexane/*i*-PrOH(1‰ TFA) = 7/3, flow rate = 1.0 mL/min), t_{major} = 4.16 min, t_{minor} = 7.93 min; **HRMS** (ESI): $C_{32}H_{30}N_3O_3$ $[M+H]^+$ calcd: 504.2282, found: 504.2277.

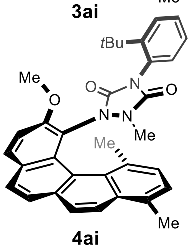

4ai

White solid, m.p. 251 – 252 °C, 88% yield, 4.3:1 dr, 97:3 er; $[\alpha]_D^{24}$ = -280 (c = 1.0 in $CHCl_3$); **HPLC analysis**: Chiralcel AD-H (*n*-hexane/*i*-PrOH(1‰ TFA) = 8/2, flow rate = 1.0 mL/min), t_{major} = 4.72 min, t_{minor} = 7.84 min; **^{1}H NMR** (300 MHz, $CDCl_3$) δ 8.10 (t, *J* = 9.1 Hz, 2H), 7.83 (d, *J* = 8.4 Hz, 1H), 7.70 (d, *J* = 8.4 Hz, 2H), 7.57 (d, *J* = 7.7 Hz, 1H), 7.39 (dd, *J* = 15.9, 8.1 Hz, 4H), 7.25 (d, *J* = 7.8 Hz, 1H), 6.87 (d, *J* = 7.3 Hz, 1H), 3.98 (s, 3H), 2.81 (s, 3H), 2.26 (s, 3H), 1.42 (s, 9H), 1.36 (s, 3H) ppm; **^{13}C NMR** (75 MHz, $CDCl_3$) δ 155.02, 152.80, 149.56, 149.47, 135.33, 133.12, 131.73, 131.64, 131.55, 131.45, 130.53, 130.11, 129.78, 128.45, 127.61, 127.51, 127.40, 127.15, 126.76, 124.97, 124.51, 124.19, 123.49, 117.69, 112.71, 56.50, 35.77, 31.36, 29.48, 23.59, 19.68 ppm; **HRMS** (ESI): $C_{34}H_{34}N_3O_3$ $[M+H]^+$ calcd: 532.2595, found: 532.2573.

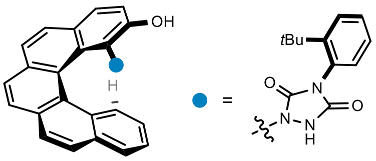

3aj

Prepared according to the general procedure on a 0.2 mmol scale and purified by flash chromatography (petroleum ether/ethyl acetate = 7/1 to 4/1). White solid, m.p. 219 – 220 °C, 41.5 mg, 40% yield, 96.5:3.5 er; $[\alpha]_D^{24}$ = -1280 (c = 1.0 in $CHCl_3$); **HPLC analysis**: Chiralcel AD-H (*n*-hexane/*i*-PrOH(1‰ TFA) = 7/3, flow rate = 1.0 mL/min), t_{major} = 5.25 min, t_{minor} = 9.42 min; **^{1}H NMR** (300 MHz, $CDCl_3$) δ 8.72 – 7.61 (m, 10H), 7.59 – 7.24 (m, 5H), 7.18 – 6.80 (m, 1H), 6.70 (d, *J* = 7.1 Hz, 1H), 6.36 – 3.31 (m, 1H), 1.14 (s, 9H) ppm; **^{13}C NMR** (75 MHz, $CDCl_3$) δ 152.09, 151.92, 148.90, 148.82, 134.31, 131.08, 130.88, 130.80, 130.72, 130.42, 129.76, 129.31, 129.02, 128.88, 127.84, 127.78, 127.44, 127.34, 127.26, 127.05, 126.40, 126.25, 125.27, 125.10, 121.16, 120.11, 118.79, 35.61, 31.35 ppm; **HRMS** (ESI): $C_{34}H_{28}N_3O_3$ $[M+H]^+$ calcd: 526.2125, found: 526.2128.

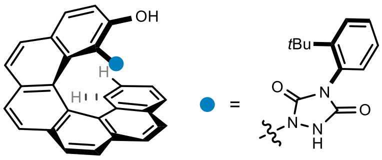

3ak

Prepared according to the general procedure on a 0.2 mmol scale and purified by flash chromatography (petroleum ether/ethyl acetate = 7/1 to 4/1). Pale yellow solid, m.p. 204 – 205 °C, 43.2 mg, 38% yield, 95.5:4.5 er; $[\alpha]_D^{24}$ = -2316 (c = 1.0 in $CHCl_3$); **HPLC analysis**: Chiralcel OD-H (*n*-hexane/*i*-PrOH(1‰ TFA) = 7/3, flow rate = 1.0 mL/min), t_{major} = 4.44 min, t_{minor} = 5.75 min; **^{1}H NMR** (300 MHz, $CDCl_3$) δ 8.25 – 7.82 (m, 10H), 7.77 (d, *J* = 8.0 Hz, 1H), 7.61 – 7.26(m, 4H), 7.19 (d, *J* = 7.5 Hz, 1H), 7.12 (d, *J* = 8.7 Hz, 1H), 6.92 (d, *J* = 8.6 Hz, 1H), 6.55 (t, *J* = 7.7 Hz, 1H), 6.09 – 4.41 (m, 1H), 1.06 (s, 9H) ppm; **^{13}C NMR** (75 MHz, $CDCl_3$) δ 151.88, 151.79, 149.29, 148.87, 133.56, 133.42, 131.77, 131.45, 130.49, 130.36, 130.00, 129.51,

129.27, 128.84, 128.63, 128.26, 127.80, 127.74, 127.42, 127.30, 127.07, 126.77, 125.97, 125.59, 125.50, 125.15, 125.05, 124.08, 123.19, 121.36, 120.76, 118.31, 35.61, 31.27 ppm; **HRMS** (ESI): $C_{38}H_{29}N_3NaO_3$ $[M+Na]^+$ calcd: 598.2101, found: 5598.2115.

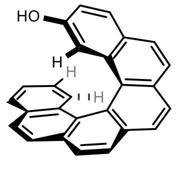

1k

Pale yellow solid, 35.4 mg (recovered), 85:15 er; **HPLC analysis**: Chiralcel IA-H (*n*-hexane/*i*-PrOH(1‰ TFA) = 8/2, flow rate = 1.0 mL/min), t_{major} = 7.16 min, t_{minor} = 24.10 min; **^{1}H NMR** (300 MHz, $CDCl_3$) δ 8.02 – 7.89 (m, 4H), 7.89 – 7.75 (m, 5H), 7.72 (d, J = 8.5 Hz, 1H), 7.65 (d, J = 8.6 Hz, 1H), 7.28 (d, J = 7.5 Hz, 1H), 6.90 (s, 1H), 6.85 – 6.69 (m, 2H), 4.23 (s, 1H) ppm; **^{13}C NMR** (75 MHz, $CDCl_3$) δ 153.05, 132.97, 131.87, 131.56, 131.29, 130.04, 129.44, 127.74, 127.65, 127.50, 127.41, 127.25, 127.02, 126.88, 126.37, 125.96, 124.81, 124.20, 123.93, 115.97, 111.61 ppm; **HRMS** (ESI): $C_{26}H_{17}O$ $[M+H]^+$ calcd: 345.1274, found: 345.1280.

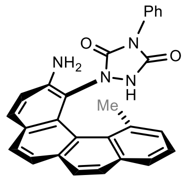

3a'

Yellow solid, m.p. 221 – 222 °C, 25% yield, 51:49 er; **HPLC analysis**: Chiralcel AD-H (*n*-hexane/*i*-PrOH = 1/1, flow rate = 1.0 mL/min), t_{major} = 6.60 min, t_{minor} = 19.84 min; **^{1}H NMR** (300 MHz, $CDCl_3$) δ 7.92 – 7.78 (m, 3H), 7.68 (t, J = 9.5 Hz, 3H), 7.39 – 7.25 (m, 7H), 7.02 (d, J = 8.6 Hz, 1H), 4.82 (s, 3H), 2.10 (s, 3H) ppm; **^{13}C NMR** (75 MHz, $CDCl_3$) δ 150.19, 147.10, 142.21, 135.07, 134.01, 132.52, 131.07, 130.61, 129.57, 128.99, 128.87, 128.04, 127.71, 127.64, 127.34, 126.27, 125.88, 125.58, 125.22, 124.92, 123.40, 121.16, 118.72, 115.17, 22.23 ppm; **HRMS** (ESI): $C_{27}H_{21}N_4O_2$ $[M+H]^+$ calcd: 433.1659, found: 433.1658.

1.6 Unsuccessful examples

Compared with diazodicarboxamides **2**, azodicarboxylate **2'** showed weaker reactivity and failed to react with polycyclic phenol **1a** under the current catalytic system (Supplementary Fig. 4a). In addition, 2-aminobenzo[*c*]phenanthrene derivative **1a'** was also recruited to undergo the current protocol. However, the corresponding amination product **3a'** was obtained with only 2% ee (Supplementary Fig. 4b).

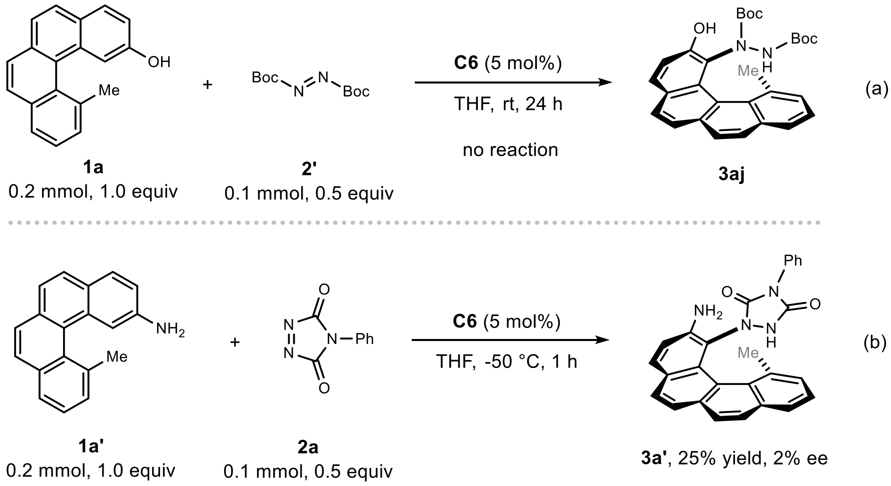

Supplementary Fig. 4. Further investigation of substrate scope**.**

2 Supplementary Discussion

2.1 Scale-up experiments of 3a and 3ab

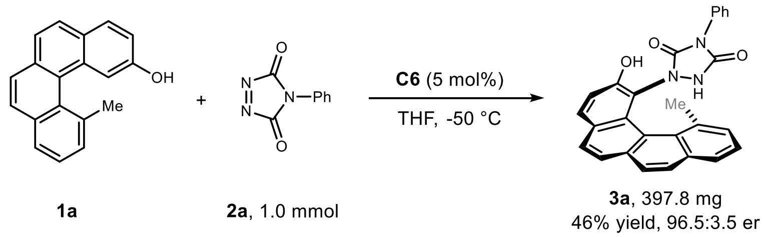

1a **2a**, 1.0 mmol **3a**, 397.8 mg 46% yield, 96.5:3.5 er

To a solution of 12-methylbenzo[*c*]phenanthren-2-ol **1a** (516.2 mg, 2.0 mmol, 1.0 equiv) and **C6** (65.1 mg, 0.1 mmol) in THF (10 mL) was added diazodicarboxamide **2a** (175.0 mg, 1.0 mmol, 0.5 equiv) at -50 °C. The resulting mixture was stirred at this temperature until the complete consumption of **2a**. After monitored by TLC, the solvent was removed under reduced pressure, and the residue was purified by silica gel column chromatography to give product **3a** in 46% yield with 96.5:3.5 er.

Scale-up experiment of **3ab** was conducted following the similar procedure with that of **3a**.

2.2 Synthetic transformations of 3a, 4a and 4ab

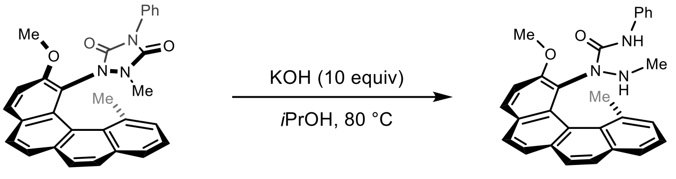

4a **5**

To a solution of **4a** (0.1 mmol, 46.1 mg) in isopropanol (1.0 mL) was added sodium hydroxide (1.0 mmol, 10 equiv). The resulting mixture was stirred at 80 °C under argon atmosphere for 15 h, the process of which was monitored by TLC analysis. After cooling, the mixture was diluted with water, the aqueous layer was extracted with EtOAc for three times, and the combined organic layers were washed with brine, dried, and concentrated. The residue was then purified by flash chromatography (eluting with hexane/ethyl acetate = 7:1) to give **5** in 51% yield. Yellow solid, 22.2 mg, 97:3 er; **HPLC analysis**: Chiralcel IA-H (*n*-hexane/*i*-PrOH = 8/2, flow rate = 1.0 mL/min), t_{major} = 5.33 min, t_{minor} = 6.37 min; **^{1}H NMR** (300 MHz, $CDCl_3$) δ 8.20 (s, 1H), 8.01 (d, *J* = 8.8 Hz, 1H), 7.93 – 7.76 (m, 2H), 7.67 (ddd, *J* = 13.1, 7.2, 1.7 Hz, 3H), 7.52 (d, *J* = 7.9 Hz, 2H), 7.47 – 7.27 (m, 4H), 7.14 (d, *J* = 7.1 Hz, 1H), 7.03 (t, *J* = 7.4 Hz, 1H), 3.98 (s, 3H), 2.28 (s, 3H), 2.11 (q, *J* = 5.9 Hz, 1H), 1.32 (d, *J* = 4.2 Hz, 3H) ppm; **^{13}C NMR** (75 MHz, $CDCl_3$) δ 155.40, 153.02, 139.74, 138.59, 133.10, 132.35, 128.81, 128.78, 128.25, 127.99, 127.91, 127.72, 127.05, 126.29, 125.40, 123.46, 123.39, 122.82, 122.63, 121.98, 118.99, 113.53, 56.94, 33.80, 23.19 ppm; **HRMS** (ESI): $C_{28}H_{26}N_3O_2$ $[M+H]^+$ calcd: 436.2020, found: 436.2016.

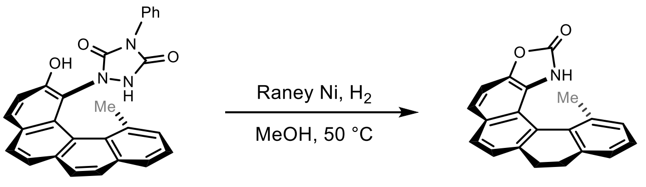

3a **6**

To a solution of **3a** (0.1 mmol, 43.3 mg) in methanol (1.0 mL) was added Raney nickel (~200 mg) under an atmosphere of H_2. The mixture was stirred at 50 °C until the complete consumption of the start material (monitored by TLC). Then the reaction mixture was filtered through a pad of Celite and the filtrate was evaporated in vacuo. The residue was purified by column chromatography on silica gel (eluting with hexane/ethyl acetate = 30:1) to afford **6** in 69% yield. White solid, 20.7 mg, 97.5:2.5 er; **HPLC analysis**: Chiralcel IA-H (*n*-hexane/*i*-PrOH = 8/2, flow rate = 1.0 mL/min), t_{minor} = 5.70 min, t_{major} = 8.97 min; **^{1}H NMR** (300 MHz, $CDCl_3$) δ 7.80 (d, *J* = 8.3 Hz, 1H), 7.68 (d, *J* = 8.8 Hz, 1H), 7.54 (s, 1H), 7.46 – 7.35 (m, 3H), 7.33 – 7.07 (m, 3H), 2.98 – 2.53 (m, 4H), 2.03 (s, 3H) ppm; **^{13}C NMR** (75 MHz, $CDCl_3$) δ 153.88, 141.59, 141.48, 140.87, 134.52, 133.73, 130.63, 130.05, 128.53, 128.15, 128.00, 125.37, 125.34, 123.93, 122.81, 118.17, 109.94, 30.64, 30.58, 20.34 ppm; **HRMS** (ESI): $C_{20}H_{15}NNaO_2$ $[M+Na]^+$ calcd: 324.0995, found: 324.0997.

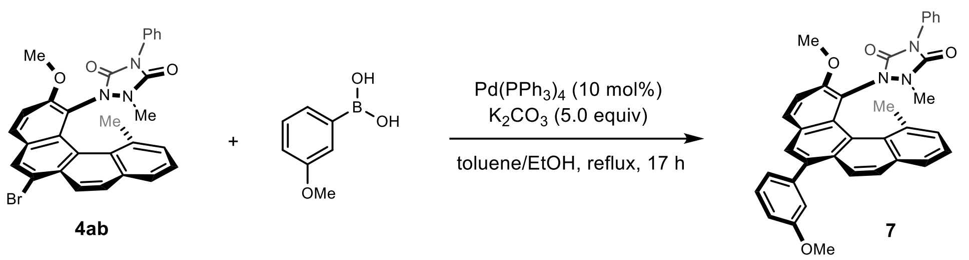

A suspension of **4ab** (0.1 mmol, 1.0 equiv), (3-methoxyphenyl)boronic acid (0.3 mmol, 3.0 equiv), $Pd(PPh_3)_4$ (0.01 mmol, 0.1 equiv) and K_2CO_3 (0.5 mmol, 5.0 equiv) in toluene/EtOH (1:1, 3.0 mL) was refluxed for 17 h under argon atmosphere. After cooling, the solvent was evaporated under vacuo and the residue was purified by silica gel column chromatography (eluting with hexane/ethyl acetate = 4:1) to give arylation product **7** in 70% yield. Pale yellow solid, m.p. 115 – 116 °C, 39.7 mg, 97:3 er, 7:1 dr; $[\alpha]_D^{24}$ = -217 (c = 1.0 in $CHCl_3$); **HPLC analysis**: Chiralcel IC-H (*n*-hexane/EtOH(1‰ TFA) = 7/3, flow rate = 1.0 mL/min), t_{minor} = 10.24 min, t_{major} = 13.80 min; **^{1}H NMR** (300 MHz, $CDCl_3$) δ 8.16 (d, *J* = 9.0 Hz, 1H), 7.87 – 7.73 (m, 4H), 7.52 – 7.43 (m, 7H), 7.38 (d, *J* = 4.4 Hz, 1H), 7.27 (d, *J* = 8.8 Hz, 1H), 7.18 – 7.09 (m, 2H), 7.03 (d, *J* = 8.2 Hz, 1H), 4.04 (s, 3H), 3.87 (s, 3H), 2.28 (s, 3H), 1.48 (s, 3H) ppm; **^{13}C NMR** (75 MHz, $CDCl_3$) δ 169.32, 161.11, 156.75, 152.70, 149.62, 142.68, 137.93, 133.97, 133.73, 133.53, 132.76, 132.45, 132.36, 130.96, 130.46, 130.35, 129.95, 129.35, 129.18, 128.48, 128.41, 127.96, 127.19, 126.90, 125.67, 125.22, 124.97, 124.57, 124.32, 118.55, 117.61, 114.85, 114.76, 58.62, 56.93, 31.31, 24.65 ppm; **HRMS** (ESI): $C_{36}H_{30}N_3O_4$ $[M+H]^+$ calcd: 568.2231, found: 568.2241.

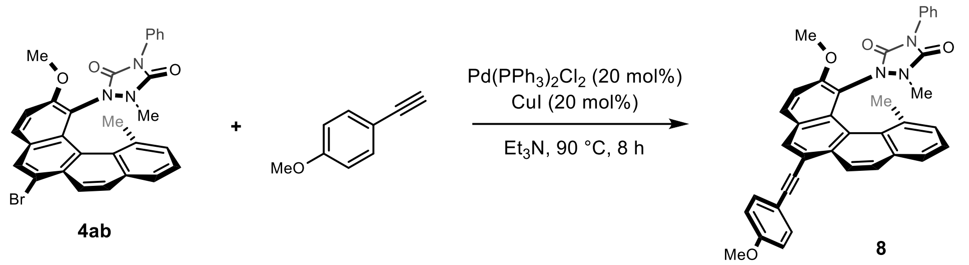

To a mixture of **4ab** (0.1 mmol, 1.0 equiv), $Pd(PPh_3)_2Cl_2$ (0.02 mmol, 20 mol%) and CuI (0.02 mmol, 20 mol%) was added Et_3N (2.0 mL) and 1-ethynyl-4-methoxybenzene (0.3 mmol, 3.0 equiv) successively under argon atmosphere. The resulting reaction mixture was then heated at 90 °C for 8 h. After cooling, the solution was concentrated in vacuo and the residue was subjected to silica gel

column chromatography (eluting with hexane/ethyl acetate = 4:1) to afford **8** in 60% yield. Pale yellow solid, m.p. 211 – 212 °C, 35.5 mg, 97:3 er, 17:1 dr; $[\alpha]_D^{24}$ = -69 (c = 1.0 in $CHCl_3$); **HPLC analysis**: Chiralcel IC-H (*n*-hexane/EtOH(1‰ TFA) = 7/3, flow rate = 1.0 mL/min), t_{minor} = 10.77 min, t_{major} = 17.72 min; **^{1}H NMR** (300 MHz, $CDCl_3$) δ 8.32 (d, *J* = 8.5 Hz, 1H), 8.19 – 8.05 (m, 2H), 7.97 (d, *J* = 8.5 Hz, 1H), 7.81 (d, *J* = 7.8 Hz, 1H), 7.62 (d, *J* = 7.9 Hz, 2H), 7.52 – 7.41 (m, 6H), 7.37 (d, *J* = 4.2 Hz, 1H), 7.26 (d, *J* = 7.1 Hz, 1H), 6.95 (d, *J* = 7.9 Hz, 2H), 4.02 (s, 3H), 3.85 (s, 3H), 2.24 (s, 3H), 1.45 (s, 3H) ppm; **^{13}C NMR** (75 MHz, $CDCl_3$) δ 159.94, 155.70, 151.23, 147.95, 136.25, 133.23, 133.13, 132.42, 131.95, 131.02, 130.71, 130.61, 129.45, 129.39, 128.95, 127.99, 127.69, 126.91, 126.52, 125.65, 124.49, 123.15, 122.61, 117.76, 117.19, 115.23, 114.22, 113.27, 94.36, 85.77, 57.04, 55.41, 29.85, 23.12 ppm; **HRMS** (ESI): $C_{38}H_{30}N_3O_4$ $[M+Na]^+$ calcd: 592.2231, found: 592.2240.

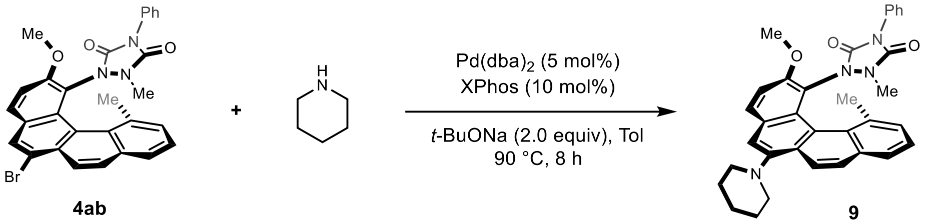

To a mixture of **4ab** (0.1 mmol, 1.0 equiv), $Pd(dba)_2$ (0.005 mmol, 5.0 mol%), XPhos (0.01 mmol, 10 mol%), and sodium *tert*-butoxide (0.2 mmol, 2.0 equiv) was added toluene (1.0 mL) and piperidine (0.15 mmol, 1.5 equiv) under argon atmosphere. The resulting reaction mixture was then stirred at 90 °C for 8 h. After cooling, the mixture was directly purified by flash chromatography (eluting with hexane/ethyl acetate = 4:1) to give **9** in 64% yield. Yellow solid, m.p. 140 – 141 °C, 34.8 mg, 97:3 er, 9:1 dr; $[\alpha]_D^{24}$ = -256 (c = 1.0 in $CHCl_3$); **HPLC analysis**: Chiralcel IC-H (*n*-hexane/EtOH(1‰ TFA) = 7/3, flow rate = 1.0 mL/min), t_{minor} = 8.44 min, t_{major} = 11.45 min; **^{1}H NMR** (300 MHz, $CDCl_3$) δ 8.11 (d, *J* = 8.6 Hz, 1H), 8.03 (d, *J* = 9.0 Hz, 1H), 7.84 (d, *J* = 8.5 Hz, 1H), 7.76 (d, *J* = 7.6 Hz, 1H), 7.51 – 7.32 (m, 8H), 7.24 (d, *J* = 8.2 Hz, 1H), 3.99 (s, 3H), 3.55 – 2.78 (m, 4H), 2.26 (s, 3H), 2.04 – 1.65 (m, 6H), 1.42 (s, 3H) ppm; **^{13}C NMR** (75 MHz, $CDCl_3$) δ 154.01, 150.94, 148.10, 147.60, 136.24, 132.12, 132.07, 131.25, 130.48, 128.90, 128.22, 127.90, 127.58, 127.36, 126.99, 126.14, 125.69, 124.80, 123.95, 120.61, 116.83, 113.39, 113.07, 57.07, 31.61, 29.46, 26.66, 24.51, 23.09, 22.68, 14.16 ppm; **HRMS** (ESI): $C_{34}H_{33}N_4O_3$ $[M+Na]^+$ calcd: 545.2547, found: 545.2559.

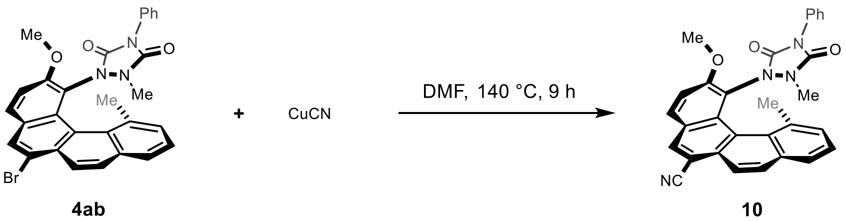

A solution of **4ab** (0.1 mmol, 1.0 equiv) and CuCN (0.5 mmol, 5.0 equiv) in dry DMF was degassed for 20 min and then stirred at 140 °C for 9 h. After cooling to room temperature, the reaction was quenched with 8% NH_4OH in water and extracted with EtOAc. The combined solvent was evaporated and the residue was purified by flash chromatography (eluting with hexane/ethyl acetate = 2:1) to give **10** in 76% yield. Pale yellow solid, m.p. 211 – 212 °C, 37.1 mg, 97:3 er, >20:1 dr; $[\alpha]_D^{24}$ = -516 (c = 1.0 in $CHCl_3$); **HPLC analysis**: Chiralcel IA-H (*n*-hexane/EtOH(1‰ TFA) = 1/1,

flow rate = 1.0 mL/min), t_{major} = 7.44 min, t_{minor} = 13.21 min; **^{1}H NMR** (300 MHz, $CDCl_3$) δ 8.36 (s, 1H), 8.22 (d, *J* = 9.0 Hz, 1H), 8.07 (s, 2H), 7.85 (d, *J* = 7.9 Hz, 1H), 7.58 – 7.45 (m, 6H), 7.38 (t, *J* = 6.4 Hz, 1H), 7.30 (d, *J* = 7.1 Hz, 1H), 4.07 (s, 3H), 2.20 (s, 3H), 1.44 (s, 3H) ppm; **^{13}C NMR** (75 MHz, $CDCl_3$) δ 157.50, 151.45, 147.94, 136.28, 134.09, 132.63, 132.06, 131.72, 131.29, 131.06, 130.87, 130.45, 129.01, 128.67, 127.85, 127.29, 125.55, 124.81, 123.23, 121.04, 117.74, 117.51, 113.87, 106.70, 57.15, 30.19, 22.99 ppm; **HRMS** (ESI): $C_{30}H_{23}N_4O_3$ $[M+Na]^+$ calcd: 487.1765, found: 487.1770.

2.3 DFT calculations on the enantiomerization process of 1a, 1j and 3a

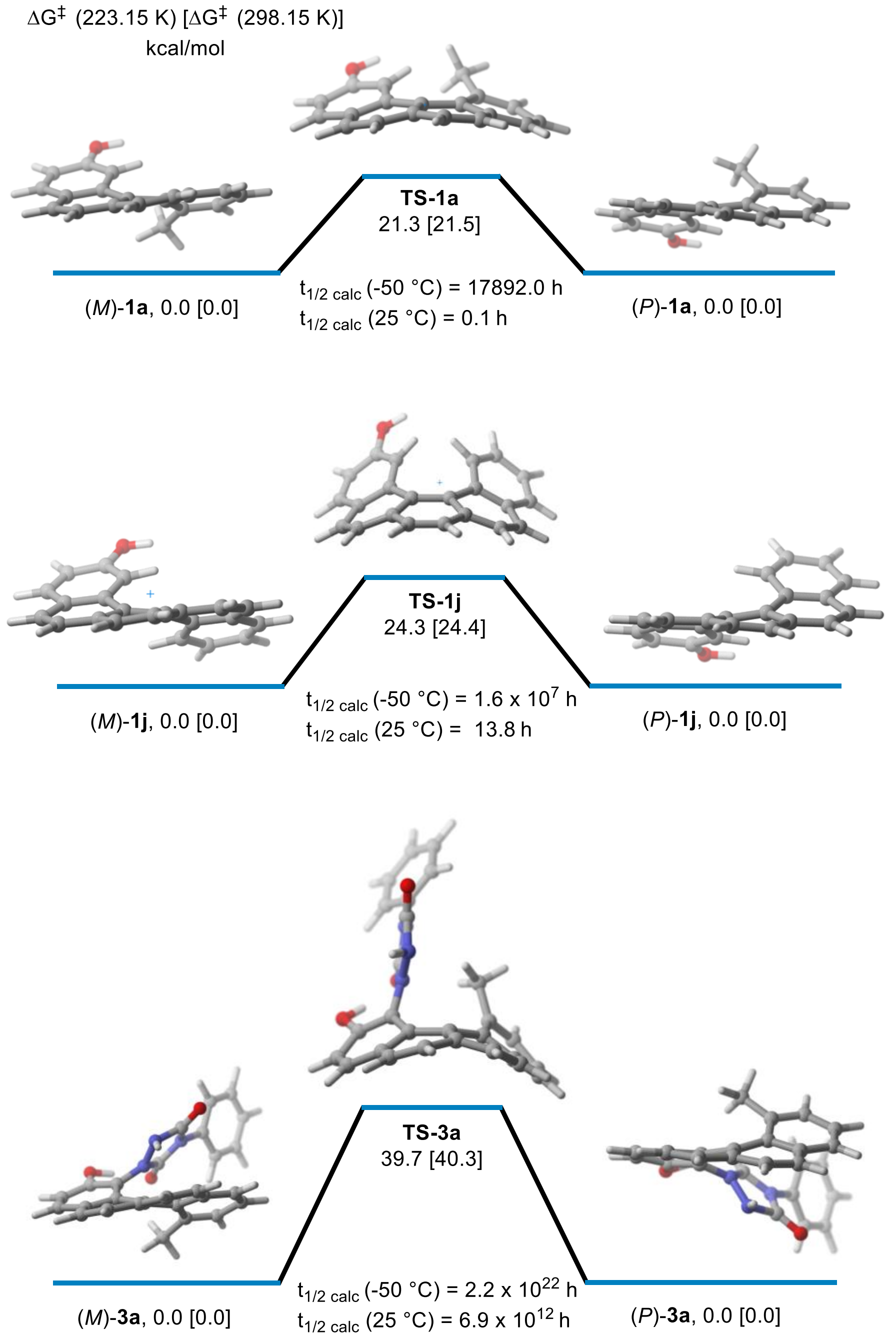

Supplementary Fig. 5. Gibbs free energy (kcal/mol) diagram for the helical chirality conversion of **1a**, **1j** and **3a**. In terms of reversible first-order reactions, $t_{1/2}$ is defined as the time at which the

concentration of reactant is halfway to its equilibrium value, and calculated as Ln(2)/(k_f+k_b). For racemization here, the forward (k_f) and backward (k_b) rate constant share the same value, and can be obtained from Erying equation, $k = (k_b T/h)\exp(-\Delta G^{\ddagger}/RT)$, in which k_b is Boltzmann constant, h is Planck constant, R is gas constant and $\Delta G^{\ddagger}$ is the free energy barrier at temperature T.

The calculated energy barriers ($\Delta G_{calc}^{\ddagger}$) for the enantiomerization process of **1a**, **1j** and **3a** are ~21.5 kcal/mol, ~24.4 kcal/mol and ~40.3 kcal/mol at room temperature, respectively (Supplementary Fig. 5). Obviously, 2-[4]helicenol **1c** and 2-[5]helicenol **1j** are all configurationally unstable, but low temperature can efficiently hamper the interconversion of their enantiomers. In comparison, 1,12-disubstituted [4]helicene **3a** was determined to be configurationally stable, with a significantly increased energy barrier for the enantiomerization process and a half-life of 6.9×10^{12} h at room temperature.

2.4 DFT calculations on the rotation barriers of the newly formed C-N bond in 3a and 4a

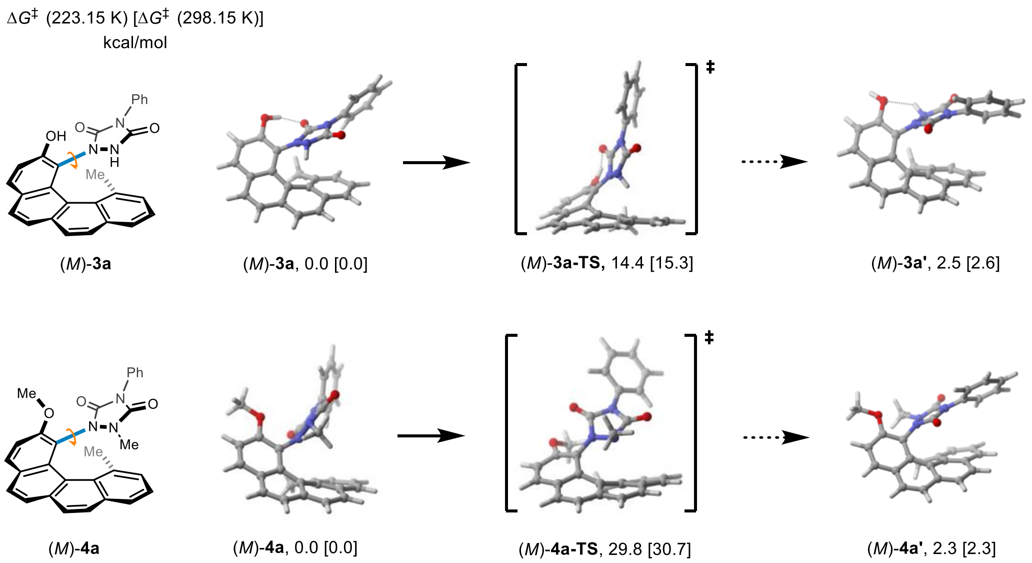

Supplementary Fig. 6. DFT calculated rotation barriers (kcal/mol) of the axial C-N bond in **3a** and **4a**.

DFT calculations reveled that while C-N bond rotation in **3a** undergoes a medium barrier of 14.4 kcal/mol, bond rotation in **4a** is kinetically hindered with a higher barrier of 29.8 kcal/mol, due to the presence of methyl substitutes.

2.5 The mechanistic studies

2.5.1 Control experiments

2-Helicenols (**1a-1j**) employed in this study are all configurationally unstable helicoidal structures. Among which, 2-[4]helicenol **1c** bearing a relatively bulky isopropyl group at its 12-position and 2-[5]helicenol **1j** were found that can be resolved by chiral HPLC at room temperature. Thus, the reactions of **1c** and **1j** were chosen to gain an insight into the mechanism of the current protocol. Both **1c** and **1j** reacted smoothly at -50 °C with **2a** to deliver the corresponding products, and the recovered **1c** and **1j** were found to be with 88.5:11.5 and 79.5:20.5 er, respectively (Supplementary Fig. 7). In addition, the racemization of recovered **1c** and **1j** was observed and completed within 5 days at room temperature. These results suggested a kinetic resolution process of configurationally

unstable [4]- and [5]-helicenes under low temperature.

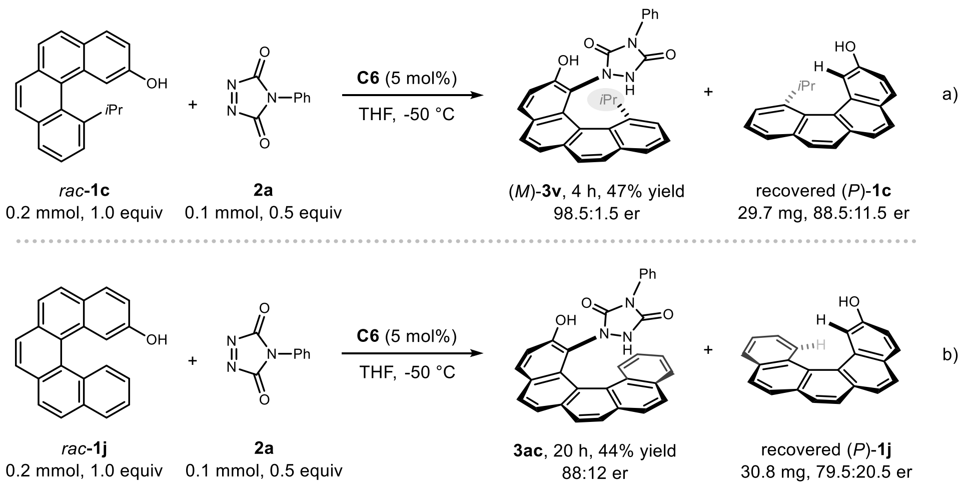

Supplementary Fig. 7. Control experiments.

HPLC result of recovered 1c

HPLC condition: Chiralcel AD-H, *n*-hexane/*i*-PrOH (1‰ TFA) = 7/3, flow rate = 1.0 mL/min.

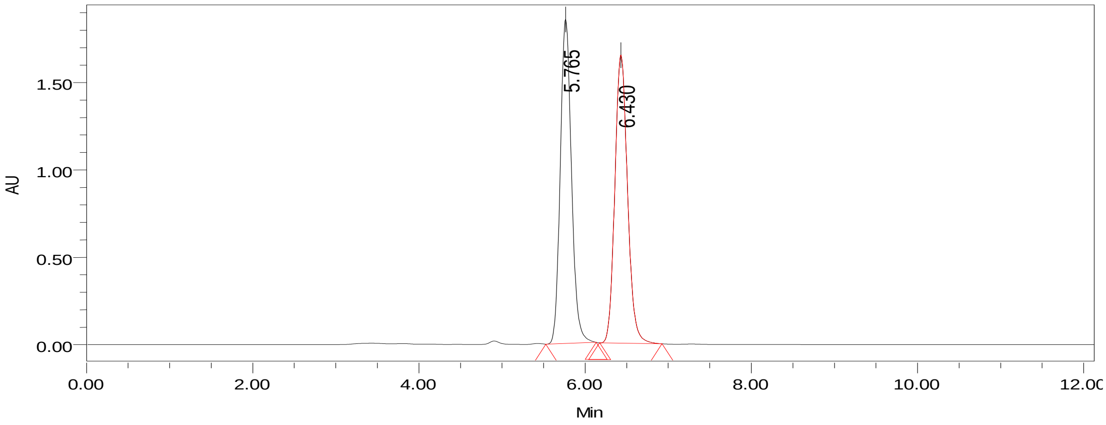

Entry	Retention time	Area	Area (%)	Height	Int type
1	5.765	17345436	49.82	1856002	bb
2	6.430	17469615	50.18	1650615	bb

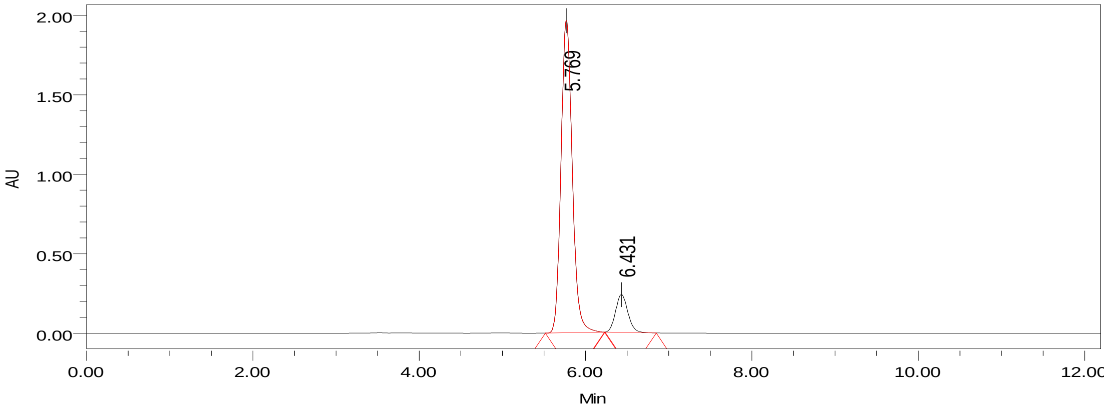

Entry	Retention time	Area	Area (%)	Height	Int type
1	5.769	18493948	88.37	1966336	bb
2	6.431	2434305	11.63	237874	bb

HPLC result of recovered 1j

HPLC condition: Chiralcel IA-H, *n*-hexane/*i*-PrOH (1‰ TFA) = 7/3, flow rate = 1.0 mL/min.

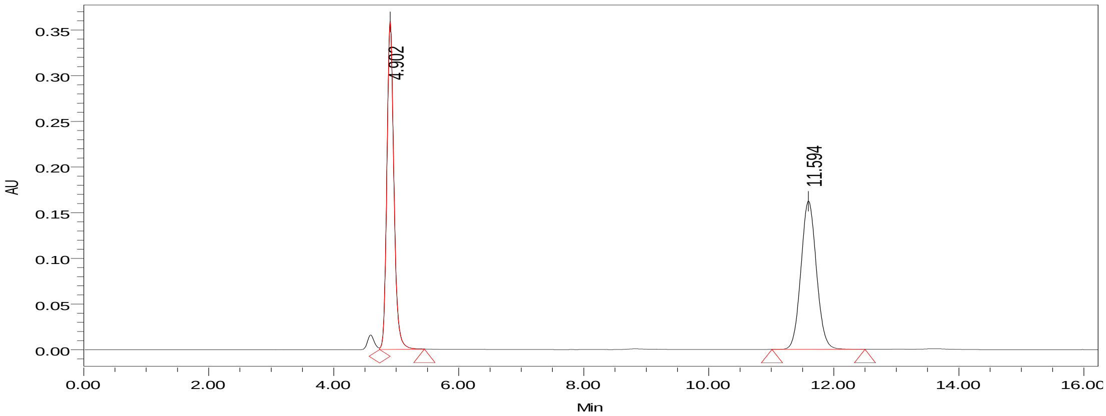

Entry	Retention time	Area	Area (%)	Height	Int type
1	4.902	2804913	50.08	359333	vb
2	11.594	2795941	49.92	162348	bb

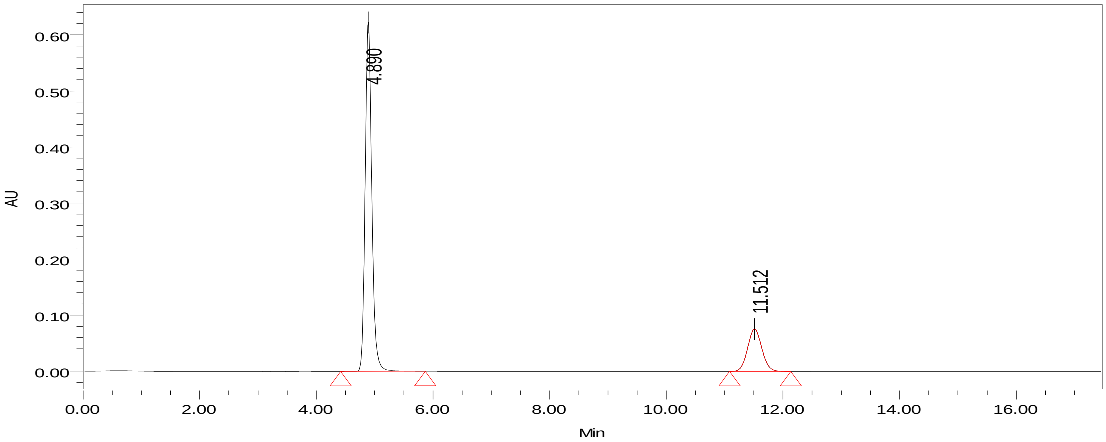

Entry	Retention time	Area	Area (%)	Height	Int type
1	4.890	4927937	79.34	623015	bb
2	11.512	1283584	20.66	75558	bb

2.5.2 DFT calculated transition states

Based on the control experiments and absolute configuration of amination products, we speculated that catalyst **C6** could selectively activate (*M*)-**1** and diazodicarboxamides **2** under low temperature (-50 °C). Therefore, two activation models, namely Brønsted acid-hydrogen bonding model (Mode A) and ion pair-hydrogen bonding model (Mode B) were taken into consideration to explain the observed helicoselectivity preference (Supplementary Fig. 8). Thus, DFT calculations were carried out to optimize the complexes between the polycyclic phenolate, diazodicarboxamide **2a** and protonated catalyst **C6**. **TS-*M***, corresponding to activation Mode A, gives access to the major product observed experimentally and is the lowest energy TS. As for the activation Mode B, the

lowest energy TS is 7.3 kcal/mol higher in energy (**TS-*M'***). The calculations revealed that catalyst **C6** brings the two reactants into close proximity via hydrogen bonding interactions and locks the relative orientation of the two substrates through their non-covalent interactions with the naphthalene skeleton and the pyrenyl substituents in the catalyst. In the most favorable transition state **TS-*M***, the bifunctional catalyst **C6** utilizes the thiourea N-H group to stabilize the anionic nucleophile (*M*)-**1a**, while the alkylammonium ion acts as a Brønsted acid to activate the diazodicarboxamide **2a**. The reaction with (*P*)-**1a** is relatively disfavored by 1.9 kcal/mol in the current catalytic system, largely due to the absence of C-H---π interactions between **1a** and the pyrenyl substituent of catalyst **C6** (**TS-*P***).

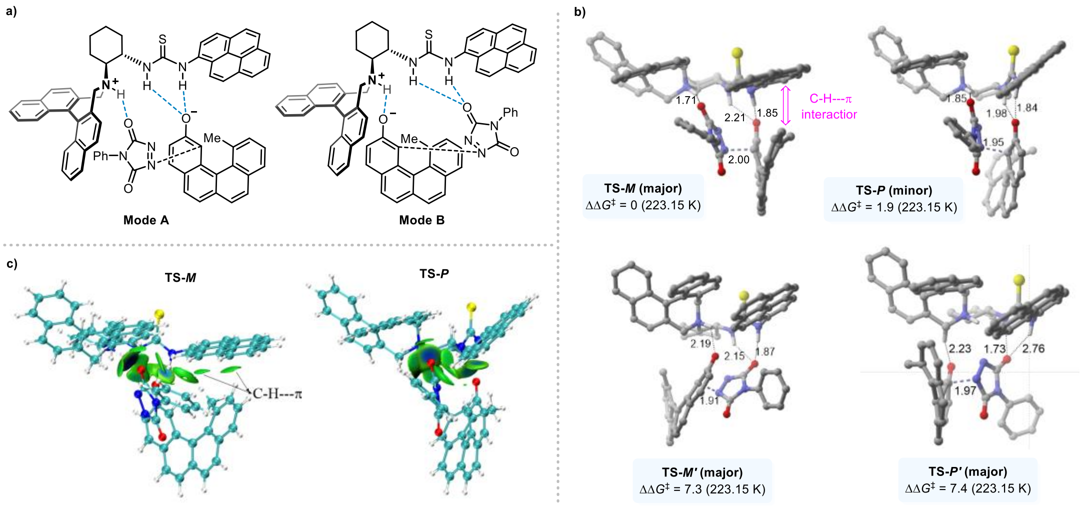

Supplementary Fig. 8. (a) Possible activation modes. (b) DFT calculated nucleophilic addition transition states with their relative free energies (in kcal/mol) and geometrical parameters (in Å). (c) IGMH (the independent gradient model based on Hirshfeld partition) plot of **TS-*M*** and **TS-*P***. Green color region indicates *van der Waals* interaction.

To gain insight into the origin of high diastereoselectivity, the complexes between (*M*)-polycyclic phenolate, diazodicarboxamide **2u** and protonated catalyst **C6** were further optimized by DFT calculations (Supplementary Fig. 9). In the transition states **TS-*MS*** and **TS-*MR***, the phenyl ring of the diazodicarboxamide compound **2u** tends to be oriented away from the terminal ring of the polycyclic phenolate, in contrast to the arrangement observed in **TS-*M***. It should be noted that the transition state **TS-*MS***, which leads to the major (*M,S*)-configured product, is thermodynamically more favored than **TS-*MR*** by 3.3 kcal/mol. This preference arises from the decreased steric repulsion between the *tert*-butyl group on **2u** and the polycyclic phenolate.

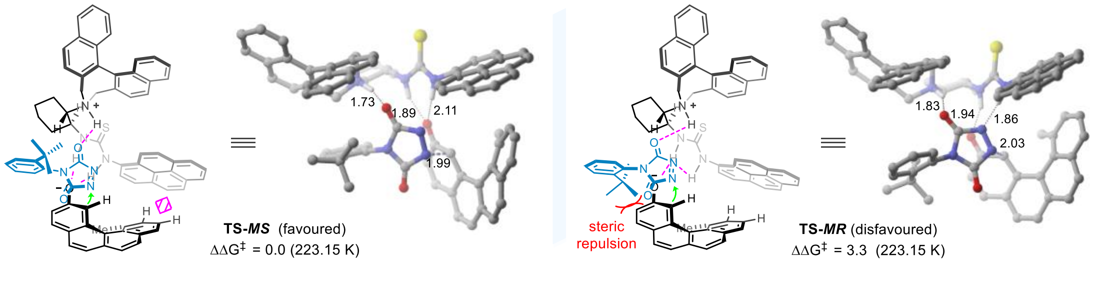

Supplementary Fig. 9. Computational study on the induction of C-N axial chirality. Transition states with their relative free energies (in kcal/mol) and geometrical parameters (in Å) are shown.

Computational Methods

Density functional theory (DFT) calculations involved in this work were performed using *Gaussian16* software.[6] Structural optimizations were conducted in the implicit tetrahydrofuran phase with the dispersion (D3) corrected M06L functional and 6-31G(d) basis set. The continuum solvation SMD model[7] was used to model the solvent (THF) effect. Reactants and intermediates were optimized to have no imaginary frequency, while transition states have only one imaginary frequency. The electronic energy of each structure was refined by single point energy calculations at the SMD M11-L/6-311+G(d,p) level. The final energetic values were obtained with thermodynamic values corrected at 223.15 K and the aqueous standard state 1 M. Since the low frequency vibrations could not be well captured by the harmonic oscillator model in the free energies calculation,[8,9] those frequencies lower than 50 cm^{-1} were raised to 50 cm^{-1} when deriving the thermodynamics data. The independent gradient model based on Hirshfeld partition (IGMH)[10] as implemented in *Multiwfn* package[11] was used to analysis the interactions between the substrate and the catalyst.

Supplementary Table 1. Absolute electronic energy and free energies at 223.15 K in the SMD(THF) solution.

Entry	E(SMD)	G_{smd}(50 cm^{-1})
**1a**	-807.7531625	-807.5045425
**TS-1a**	-807.7199117	-807.4705877
**1j**	-922.0875978	-921.8215028
**TS-1j**	-922.04839	-921.782829
**3a**	-1430.355596	-1429.984399
**TS-3a**	-1430.292674	-1429.921079
**(*M*)-3a-TS**	-1430.332461	-1429.961459
**(*M*)-3a’**	-1430.350952	-1429.980426
**(*M*)-4a**	-1508.947856	-1508.522976
**(*M*)-4a-TS**	-1508.900801	-1508.475458
**(*M*)-4a’**	-1508.944747	-1508.519384
**TS-*M***	-3730.073144	-3729.013408
**TS-*P***	-3730.071289	-3729.010354
**TS-*M’***	-3730.062145	-3729.001721
**TS-*P’***	-3730.062769	-3729.001538

2.6 Determination of the enantiomerization barrier of 6

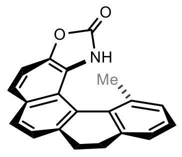

6

Kinetic of enantiomerization of **6** in mesitylene[12-14]

About 1 mg of the enantioenriched **6** was dissolved in mesitylene and heated at 160 °C. The change in enantiomeric excess over time was monitored via chiral HPLC.

Time (seconds)	Enantiomeric Excess (ee)	First Order Racemization (ln[ee_0/ee])
0	96.5	0
3600	94.8	0.01777
7200	92.6	0.04125
14400	88.9	0.08203
21600	85.7	0.11869
32400	79.7	0.19127

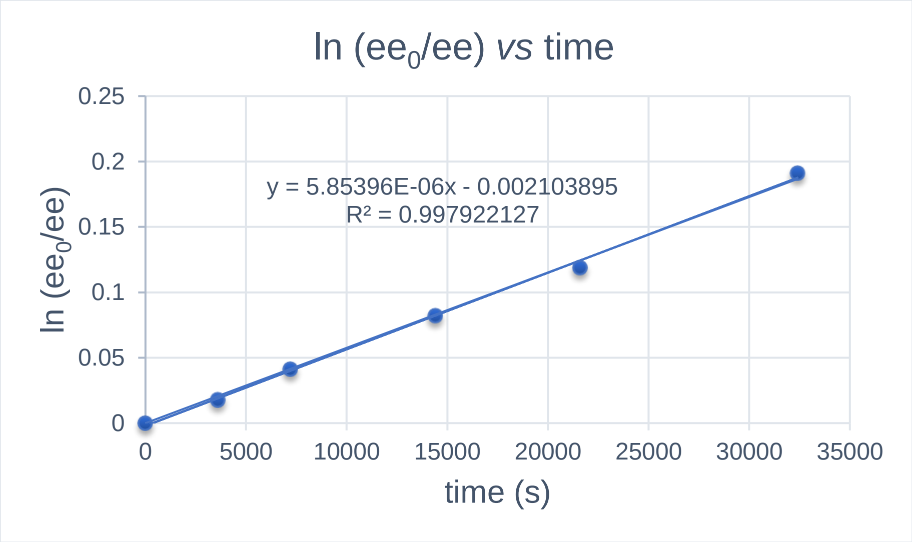

$k_{racemization}$ = 5.8540 × 10^{-6} s^{-1} (160 °C, mesitylene)
$k_{enantiomerization}$ = 2.9270 × 10^{-6} s^{-1} (160 °C, mesitylene)
$\Delta G^{\ddagger}_{enantiomerization}$ = 153.3 kJ/mol = 36.6 kcal/mol (160 °C, mesitylene)
$t_{1/2}^{rac}$ = 1.1841 × 10^5 s = 1.9735 × 10^3 min = 32.9 h (160 °C, mesitylene)

It is noteworthy that no change in ee value of *M*-**5** (94% ee) was observed, even after heating it for 12 h at 185 °C in DMSO. This observation indicates that the configuration of [4]helicene **5** is more thermally stable than that of helicene **6**.

2.7 Representative CD spectra

The circular dichroism spectra of (*M*)-**3a**, (*M*)-**3r**, and (*M*)-**3ac** were recorded. A significant agreement between the CD signatures of these helicenes and those of (*M*,*S*)-**3ad** was observed.

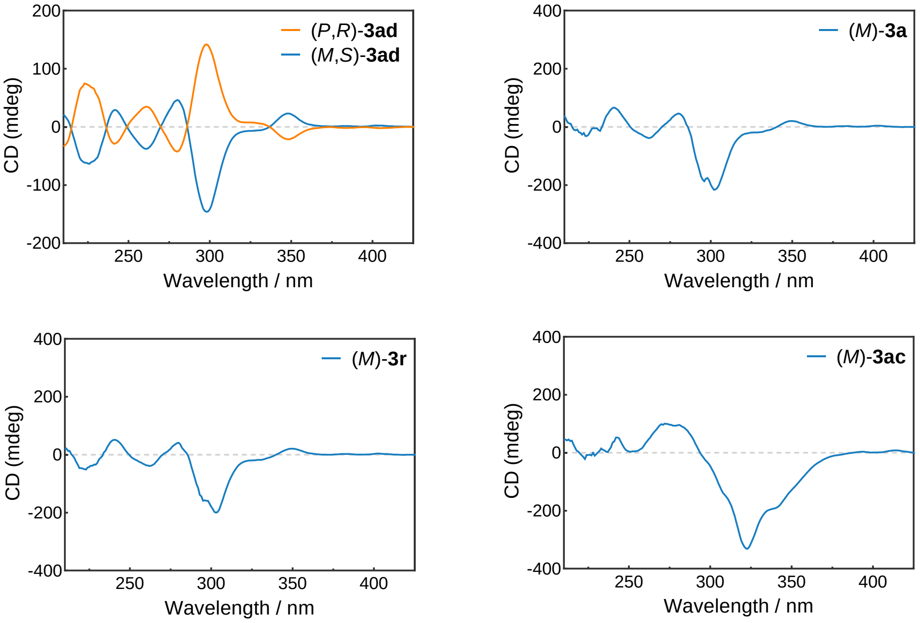

Supplementary Fig. 10. Circular dichroic (CD) spectra of (*M*,*S*)/(*P*,*R*)-**3ad**, (*M*)-**3a**, (*M*)-**3r**, and (*M*)-**3ac** in ethanol (1.0×10^{-3} M) at room temperature.

2.8 X-Ray structures of 4ad and 6

Crystal data and structure refinement for 4ad (CCDC 2179781)

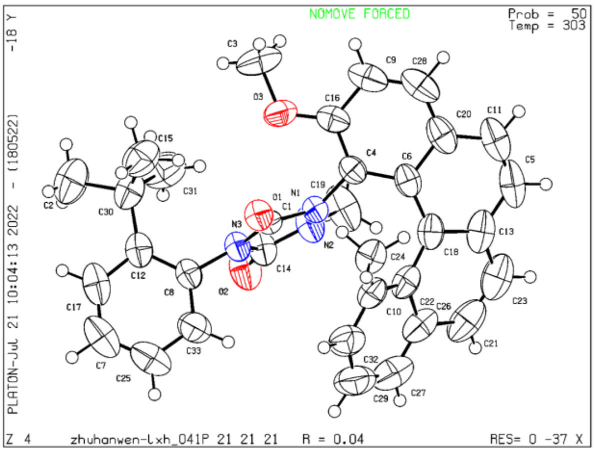

50% probability ellipsoids

Empirical formula	$C_{33}H_{31}N_3O_3$
Formula weight	517.61
Temperature/K	303.38(10)
Crystal system	orthorhombic
Space group	$P2_12_12_1$
a/Å	13.4071(2)
b/Å	15.1648(2)
c/Å	13.7356(2)
α/°	90
β/°	90
γ/°	90

Volume/Å^3	2792.67(7)
Z	4
ρ_{calc}g/cm^3	1.231
μ/mm^{-1}	0.633
F(000)	1096.0
Crystal size/mm^3	0.11 × 0.07 × 0.05
Radiation	Cu Kα (λ = 1.54184)
2Θ range for data collection/°	8.686 to 152.44
Index ranges	-16 ≤ h ≤ 16, -15 ≤ k ≤ 18, -15 ≤ l ≤ 17
Reflections collected	16736
Independent reflections	5557 [R_{int} = 0.0421, R_{sigma} = 0.0402]
Data/restraints/parameters	5557/0/358
Goodness-of-fit on F^2	1.062
Final R indexes [I>=2σ (I)]	R_1 = 0.0449, wR_2 = 0.1174
Final R indexes [all data]	R_1 = 0.0492, wR_2 = 0.1214
Largest diff. peak/hole / e Å^{-3}	0.17/-0.18
Flack parameter	0.07(13)

Crystal data and structure refinement for 6 (CCDC 2179811)

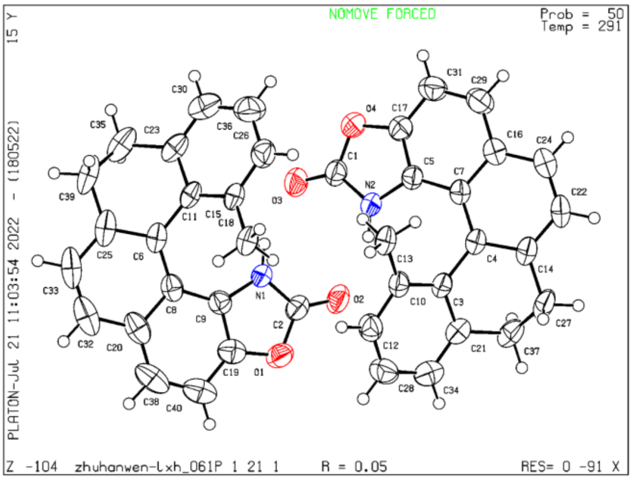

50% probability ellipsoids

Empirical formula	$C_{40}H_{30}N_2O_4$
Formula weight	602.66
Temperature/K	291(2)
Crystal system	monoclinic
Space group	$P2_1$
a/Å	10.34940(10)
b/Å	8.63050(10)
c/Å	17.1328(2)
α/°	90
β/°	96.2490(10)
γ/°	90
Volume/Å^3	1521.22(3)

Z	2
ρcalcg/cm^3	1.316
μ/mm^{-1}	0.681
F(000)	632.0
Crystal size/mm^3	0.08 × 0.05 × 0.04
Radiation	Cu Kα (λ = 1.54184)
2Θ range for data collection/°	5.188 to 155.002
Index ranges	-12 ≤ h ≤ 12, -10 ≤ k ≤ 10, -21 ≤ l ≤ 19
Reflections collected	18546
Independent reflections	6073 [R_{int} = 0.0718, R_{sigma} = 0.0460]
Data/restraints/parameters	6073/1/417
Goodness-of-fit on F^2	1.047
Final R indexes [I>=2σ (I)]	R_1 = 0.0476, wR_2 = 0.1145
Final R indexes [all data]	R_1 = 0.0502, wR_2 = 0.1167
Largest diff. peak/hole / e Å^{-3}	0.25/-0.30
Flack parameter	0.19(13)

2.9 HPLC of racemic and enantioenriched products 3 and 4

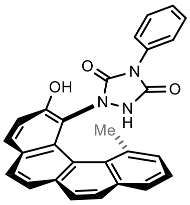

3a

HPLC condition: Chiralcel AD-H, *n*-hexane/*i*-PrOH(1‰ TFA) = 7/3, flow rate = 1.0 mL/min.

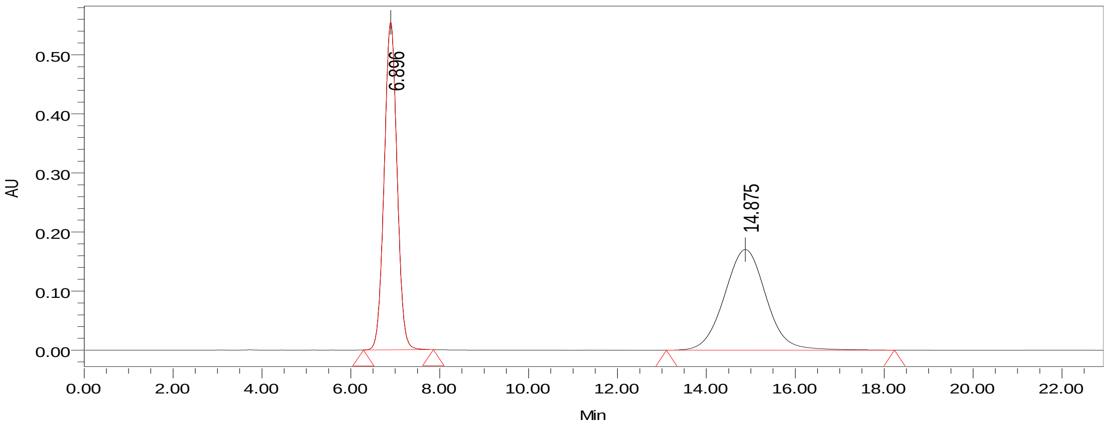

Entry	Retention time	Area	Area (%)	Height	Int type
1	6.896	11180320	50.20	554571	bb
2	14.875	11089386	49.80	170388	bb

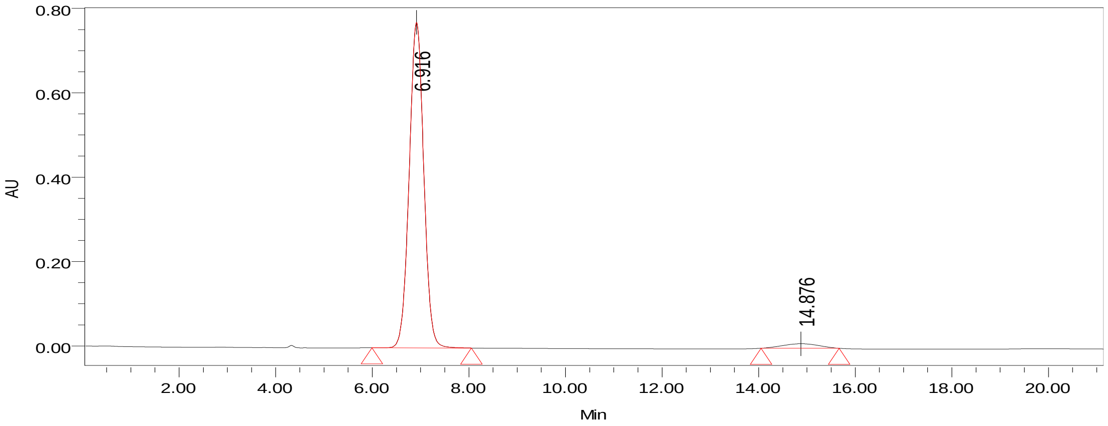

Entry	Retention time	Area	Area (%)	Height	Int type
1	6.916	16168210	96.68	771580	bb
2	14.876	554646	3.32	10732	bb

Supplementary Fig. 11. HPLC chromatograms of compound **3a.**

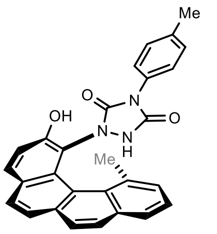

3b

HPLC condition: Chiralcel AD-H, *n*-hexane/*i*-PrOH(1‰ TFA) = 7/3, flow rate = 1.0 mL/min.

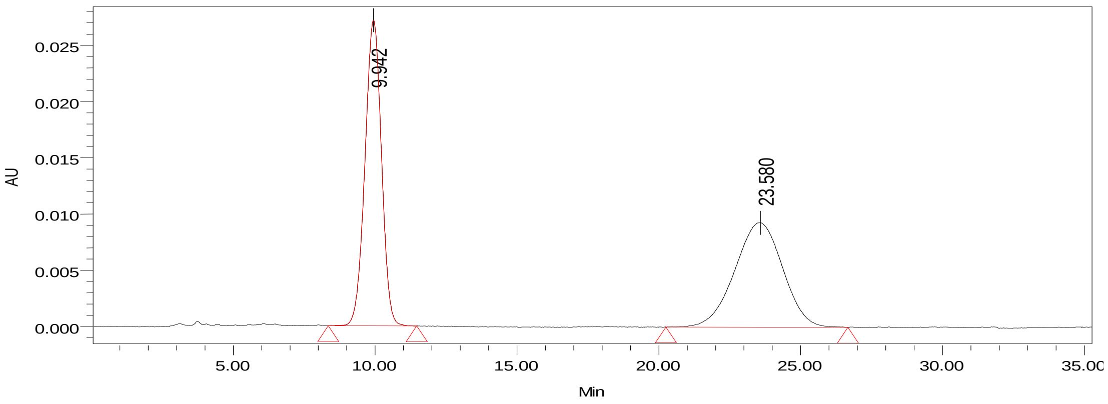

Entry	Retention time	Area	Area (%)	Height	Int type
1	9.942	1099080	50.14	27166	bb
2	23.580	1093027	49.86	9287	bb

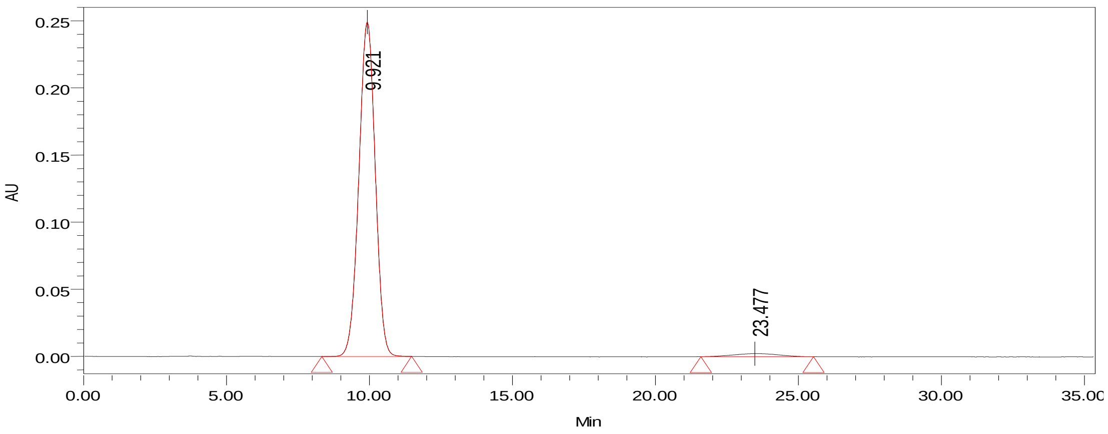

Entry	Retention time	Area	Area (%)	Height	Int type
1	9.921	9960335	97.57	249065	bb
2	23.477	247892	2.43	2278	bb

Supplementary Fig. 12. HPLC chromatograms of compound **3b.**

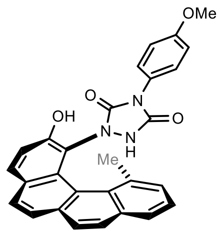

3c

HPLC condition: Chiralcel AD-H, *n*-hexane/*i*-PrOH(1‰ TFA) = 6/4, flow rate = 1.0 mL/min.

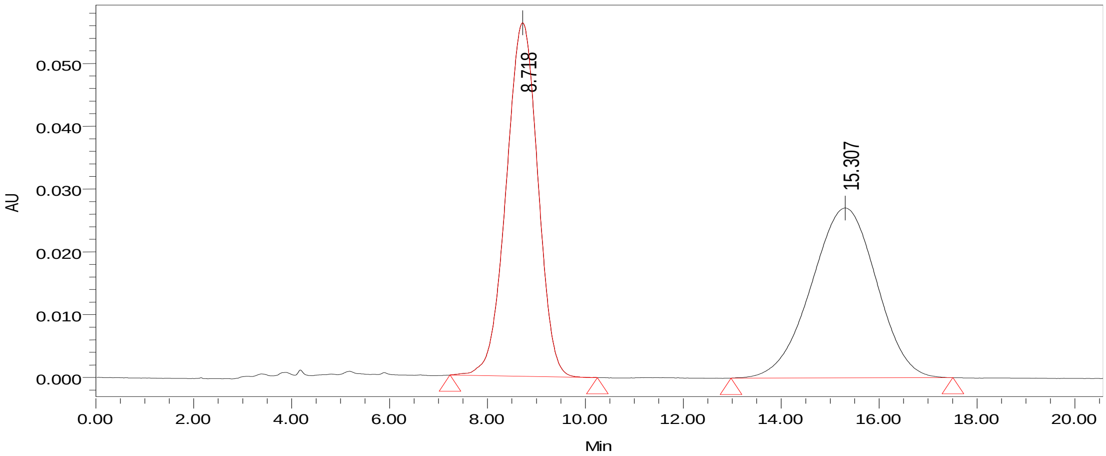

Entry	Retention time	Area	Area (%)	Height	Int type
1	8.718	2531382	50.27	56281	bb
2	15.307	2504368	49.73	27051	bb

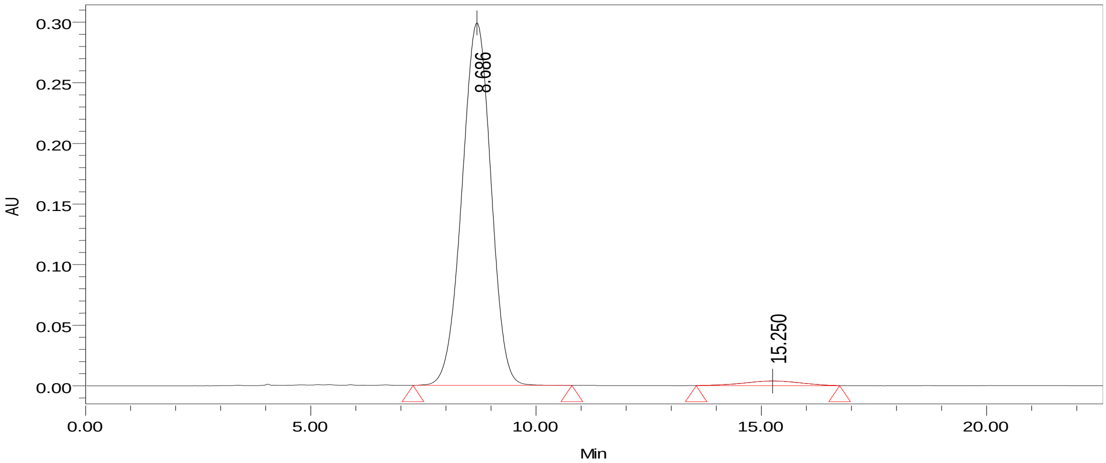

Entry	Retention time	Area	Area (%)	Height	Int type
1	8.686	13419557	97.62	299037	bb
2	15.250	327800	2.38	3727	bb

Supplementary Fig. 13. HPLC chromatograms of compound **3c.**

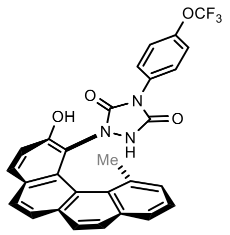

3d

HPLC condition: Chiralcel AD-H, *n*-hexane/*i*-PrOH(1‰ TFA) = 7/3, flow rate = 1.0 mL/min.

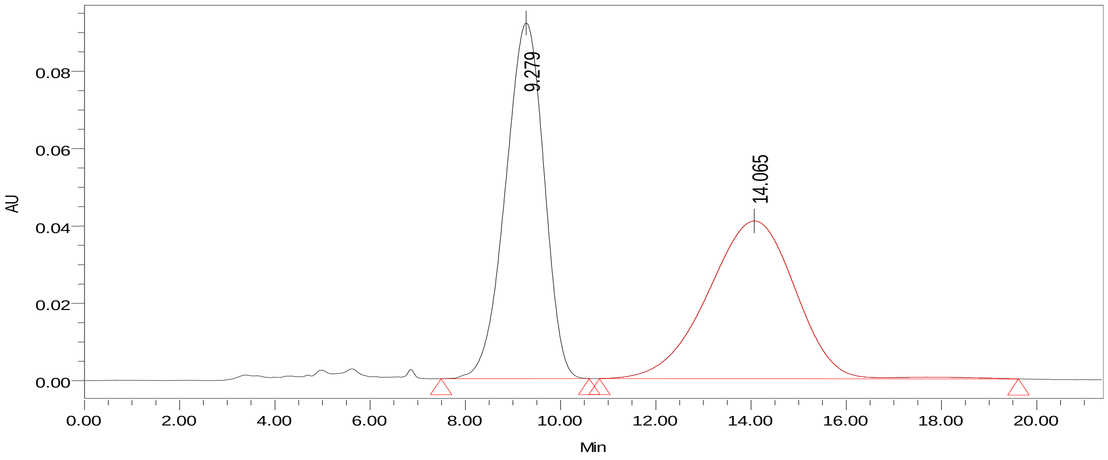

Entry	Retention time	Area	Area (%)	Height	Int type
1	9.279	5175476	49.84	91956	bb
2	14.065	5208610	50.16	40775	bb

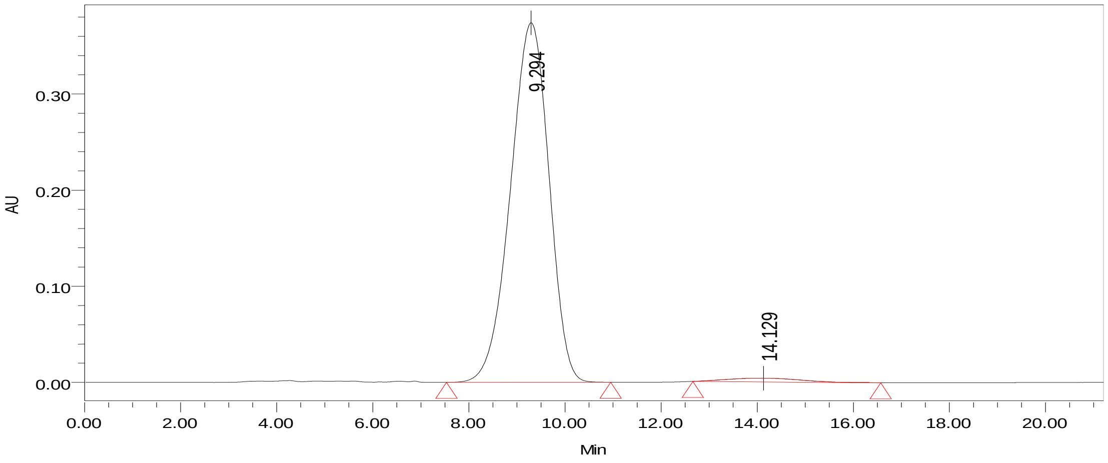

Entry	Retention time	Area	Area (%)	Height	Int type
1	9.294	20858432	98.12	373971	bb
2	14.129	398920	1.88	3903	bb

Supplementary Fig. 14. HPLC chromatograms of compound **3d.**

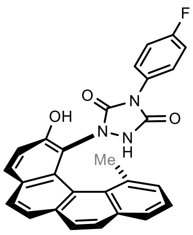

3e

HPLC condition: Chiralcel AD-H, *n*-hexane/*i*-PrOH(1‰ TFA) = 7/3, flow rate = 1.0 mL/min.

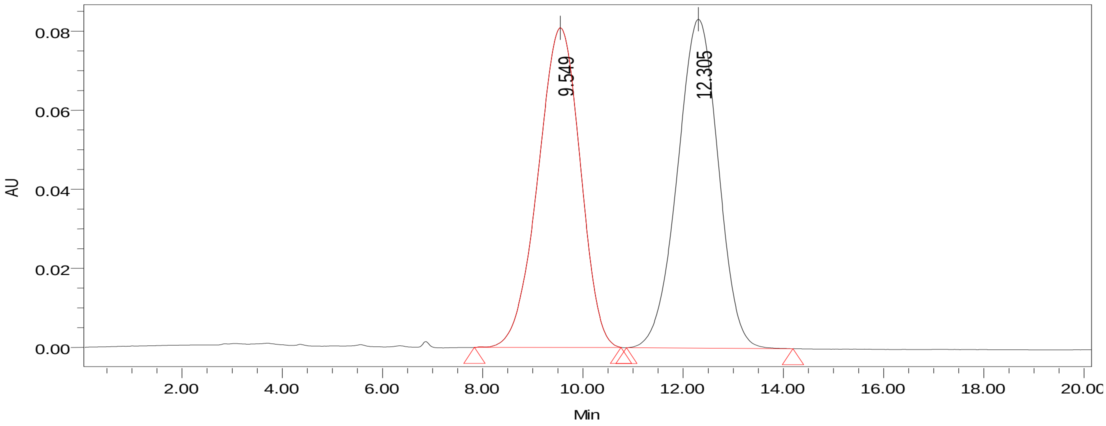

Entry	Retention time	Area	Area (%)	Height	Int type
1	9.549	4732211	50.13	80841	bb
2	12.305	4707911	49.87	83173	bb

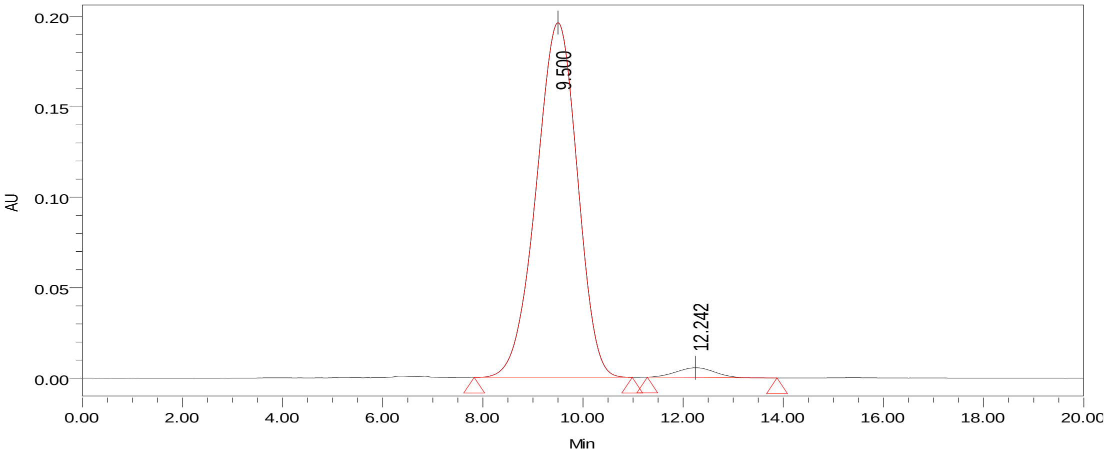

Entry	Retention time	Area	Area (%)	Height	Int type
1	9.500	11195322	97.51	196011	bb
2	12.242	286370	2.49	5375	bb

Supplementary Fig. 15. HPLC chromatograms of compound **3e.**

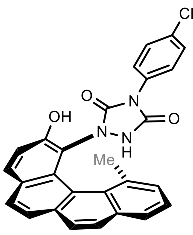

3f

HPLC condition: Chiralcel AD-H, *n*-hexane/*i*-PrOH(1‰ TFA) = 7/3, flow rate = 1.0 mL/min.

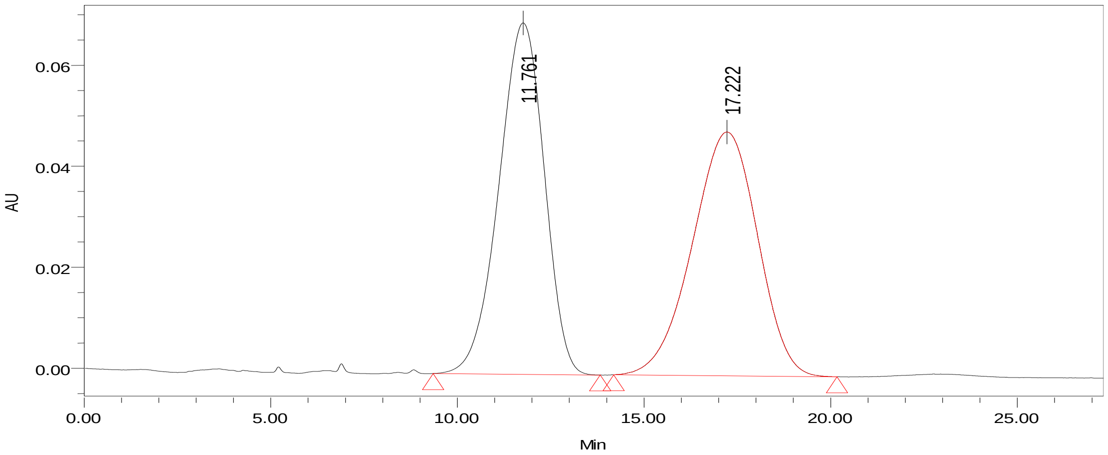

Entry	Retention time	Area	Area (%)	Height	Int type
1	11.761	5892961	50.14	69588	bb
2	17.222	5860536	49.86	48278	bb

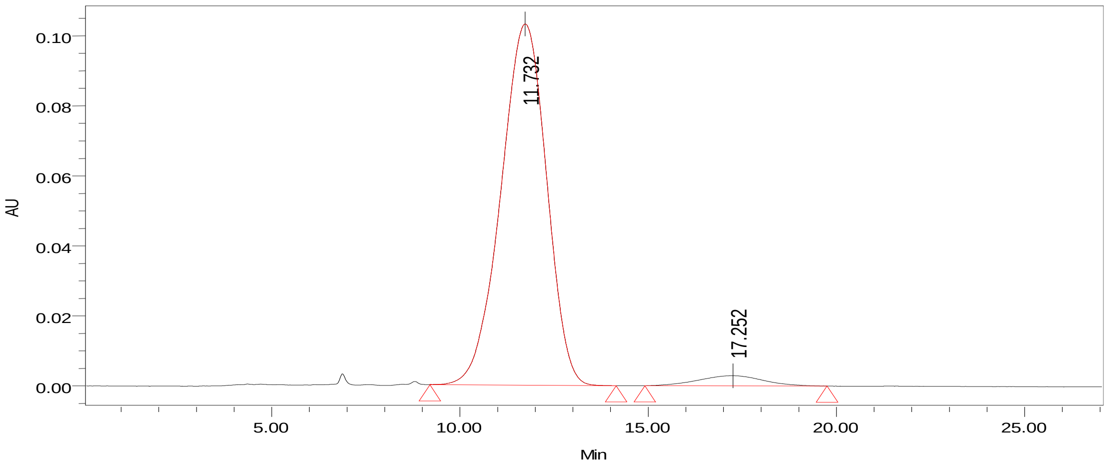

Entry	Retention time	Area	Area (%)	Height	Int type
1	11.732	8710247	96.18	103177	bb
2	17.252	346171	3.82	2914	bb

Supplementary Fig. 16. HPLC chromatograms of compound **3f.**

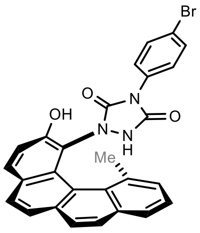

3g

HPLC condition: Chiralcel AD-H, *n*-hexane/*i*-PrOH(1‰ TFA) = 7/3, flow rate = 1.0 mL/min.

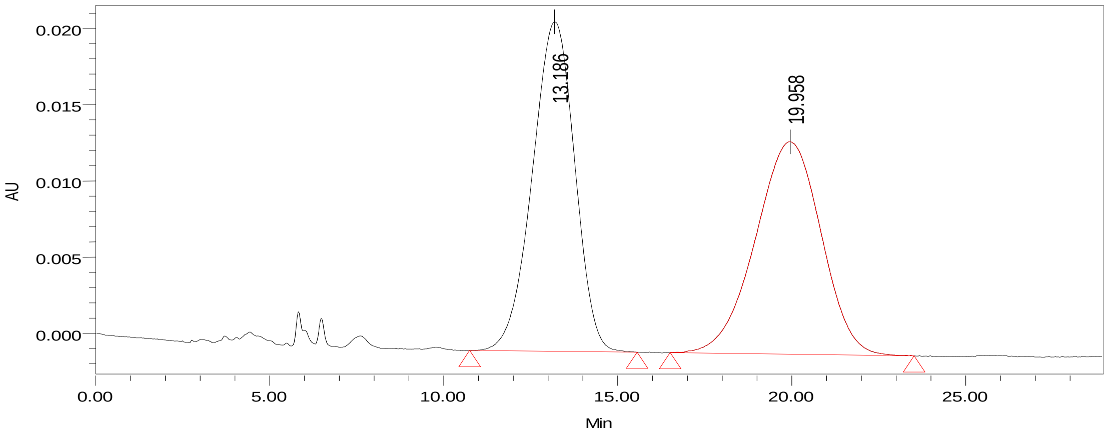

Entry	Retention time	Area	Area (%)	Height	Int type
1	13.186	1833453	50.04	21599	bb
2	19.958	1830644	49.96	13933	bb

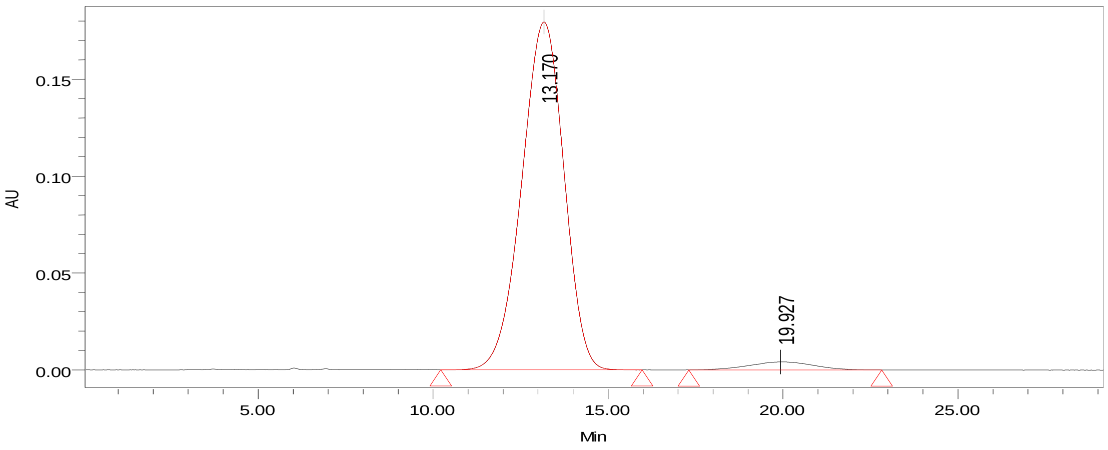

Entry	Retention time	Area	Area (%)	Height	Int type
1	13.170	15079095	96.59	179436	bb
2	19.927	533095	3.41	4159	bb

Supplementary Fig. 17. HPLC chromatograms of compound **3g.**

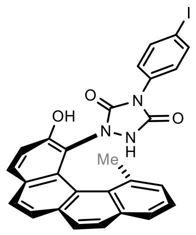

3h

HPLC condition: Chiralcel AD-H, *n*-hexane/*i*-PrOH(1‰ TFA) = 7/3, flow rate = 1.0 mL/min.

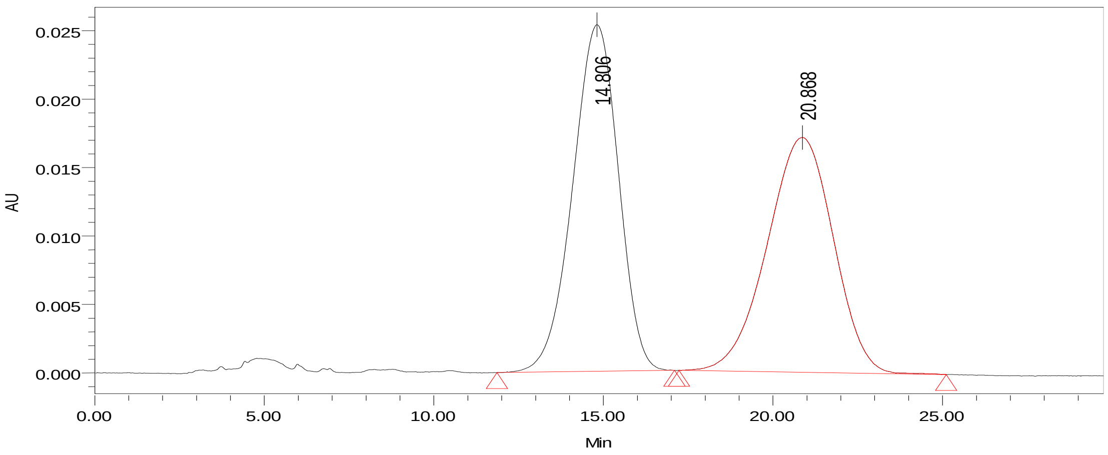

Entry	Retention time	Area	Area (%)	Height	Int type
1	14.806	2377297	50.52	25326	bb
2	20.868	2328468	49.48	17147	bb

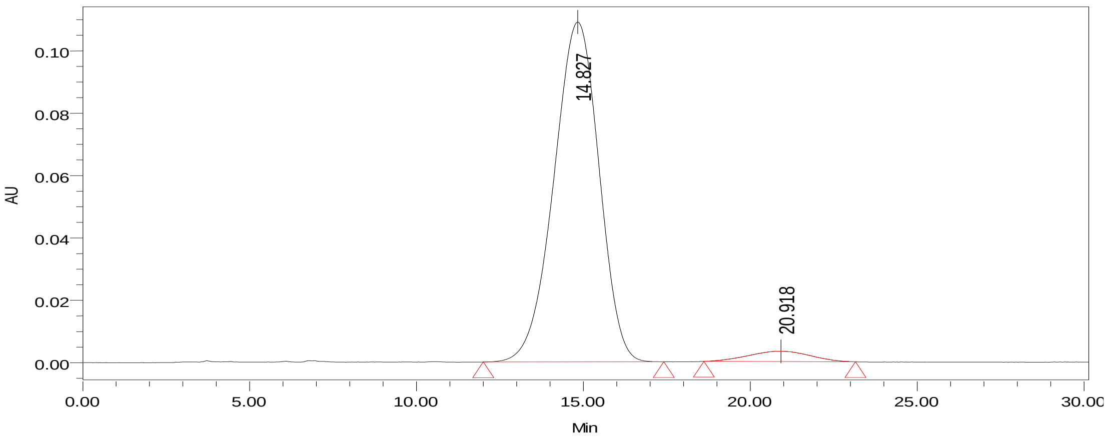

Entry	Retention time	Area	Area (%)	Height	Int type
1	14.827	10274327	96.01	108983	bb
2	20.918	427193	3.99	3337	bb

Supplementary Fig. 18. HPLC chromatograms of compound **3h.**

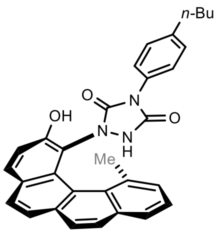

3i

HPLC condition: Chiralcel AD-H, *n*-hexane/*i*-PrOH(1‰ TFA) = 7/3, flow rate = 1.0 mL/min.

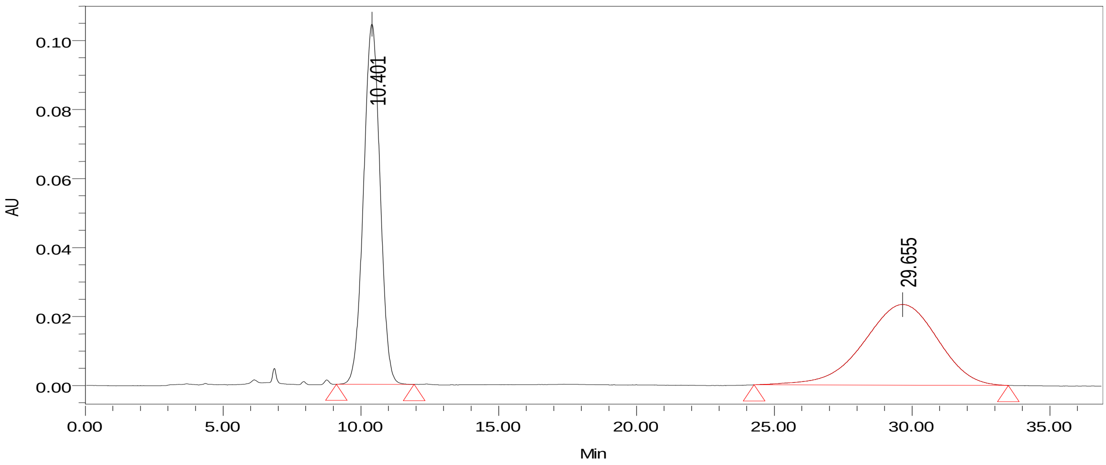

Entry	Retention time	Area	Area (%)	Height	Int type
1	10.401	4368437	49.96	104363	bb
2	29.655	4375822	50.04	23387	bb

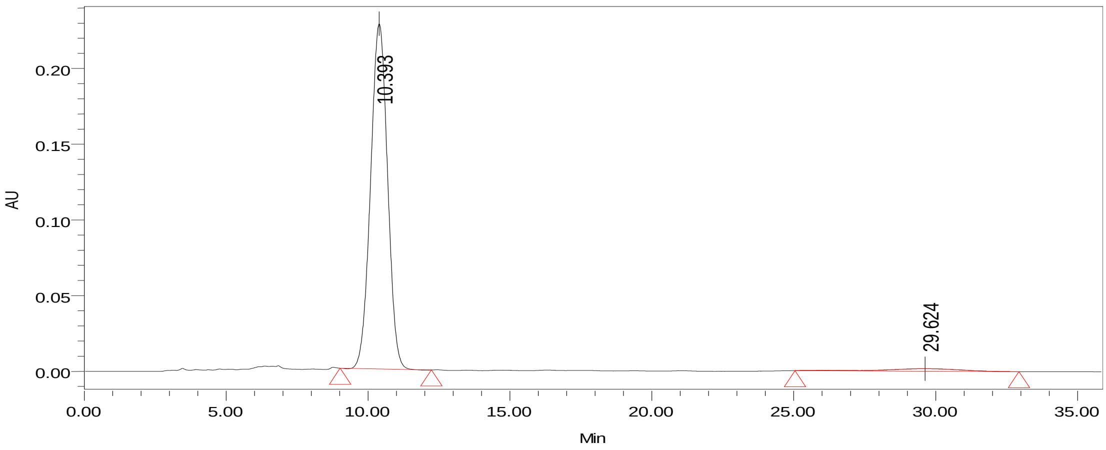

Entry	Retention time	Area	Area (%)	Height	Int type
1	10.393	9495976	96.91	227918	bb
2	29.624	303027	3.09	1677	bb

Supplementary Fig. 19. HPLC chromatograms of compound **3i.**

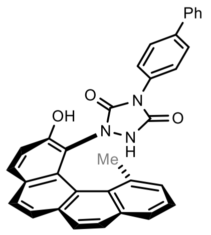

3j

HPLC condition: Chiralcel AD-H, *n*-hexane/*i*-PrOH(1‰ TFA) = 7/3, flow rate = 1.0 mL/min.

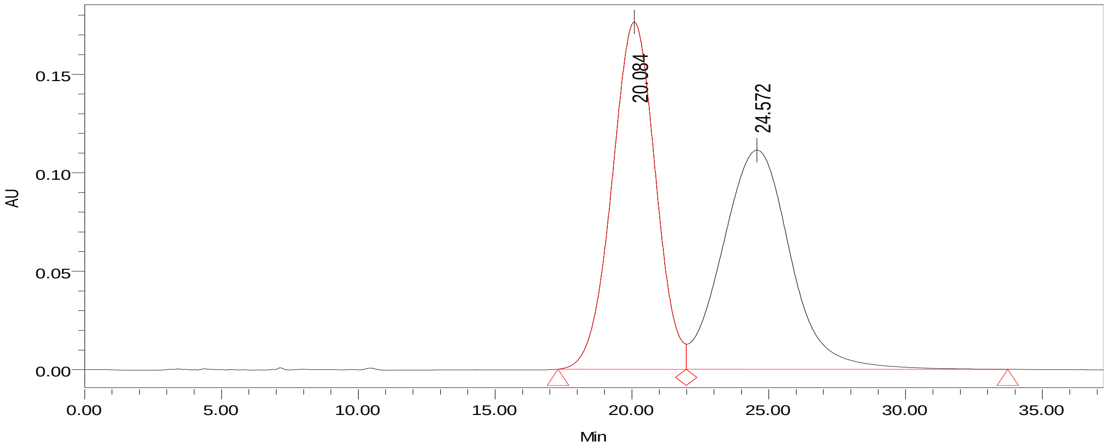

Entry	Retention time	Area	Area (%)	Height	Int type
1	20.084	19473487	50.46	176232	bv
2	24.572	19119252	49.54	111251	vb

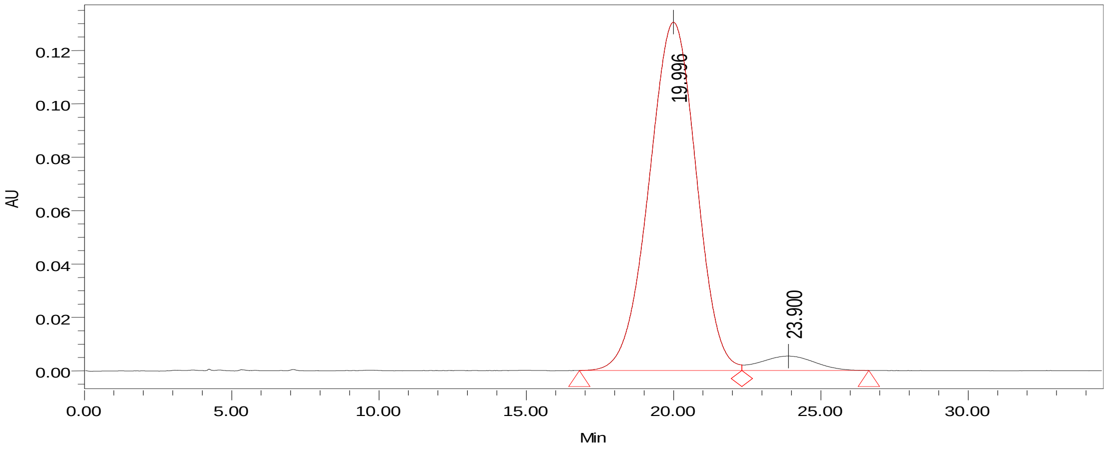

Entry	Retention time	Area	Area (%)	Height	Int type
1	19.996	14565051	95.49	130392	bv
2	23.900	687354	4.51	5373	vb

Supplementary Fig. 20. HPLC chromatograms of compound **3j.**

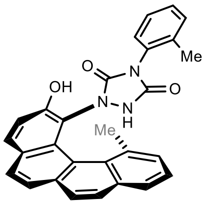

3k

HPLC condition: Chiralcel AD-H, *n*-hexane/*i*-PrOH(1‰ TFA) = 7/3, flow rate = 1.0 mL/min.

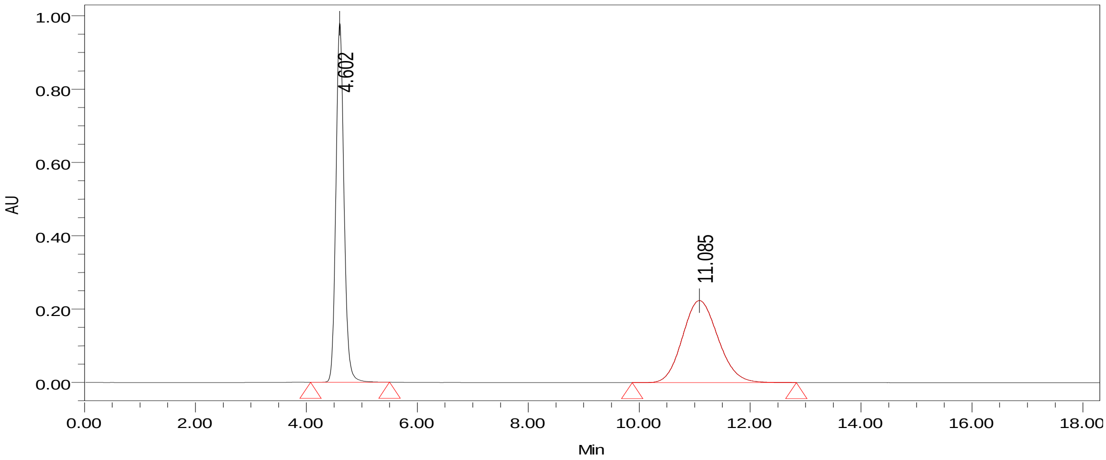

Entry	Retention time	Area	Area (%)	Height	Int type
1	4.602	9940410	49.98	980845	bb
2	11.085	9950161	50.02	224000	bb

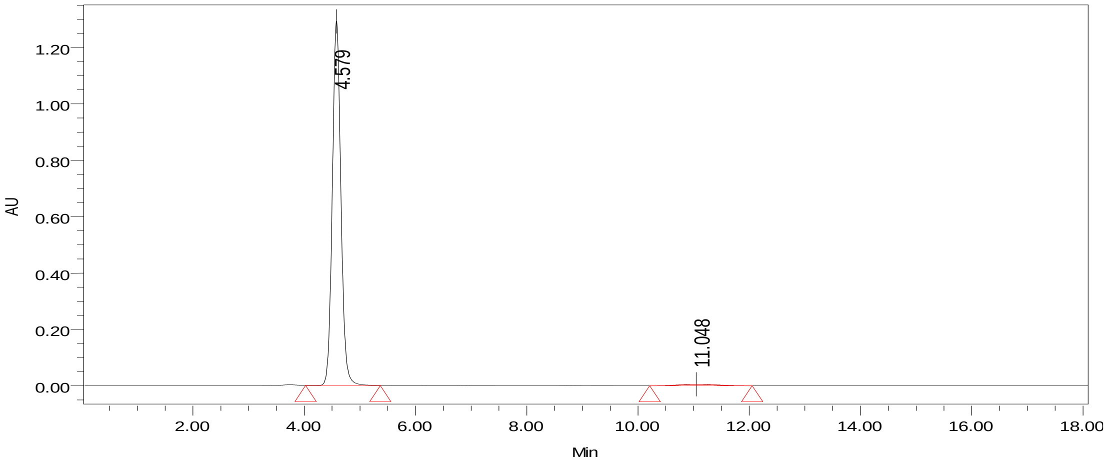

Entry	Retention time	Area	Area (%)	Height	Int type
1	4.579	13920217	98.18	1294162	bb
2	11.048	257819	1.82	5637	bb

Supplementary Fig. 21. HPLC chromatograms of compound **3k.**

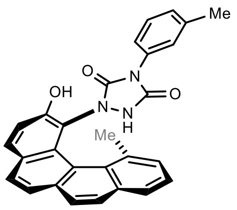

3l

HPLC condition: Chiralcel AD-H, *n*-hexane/*i*-PrOH(1‰ TFA) = 7/3, flow rate = 1.0 mL/min.

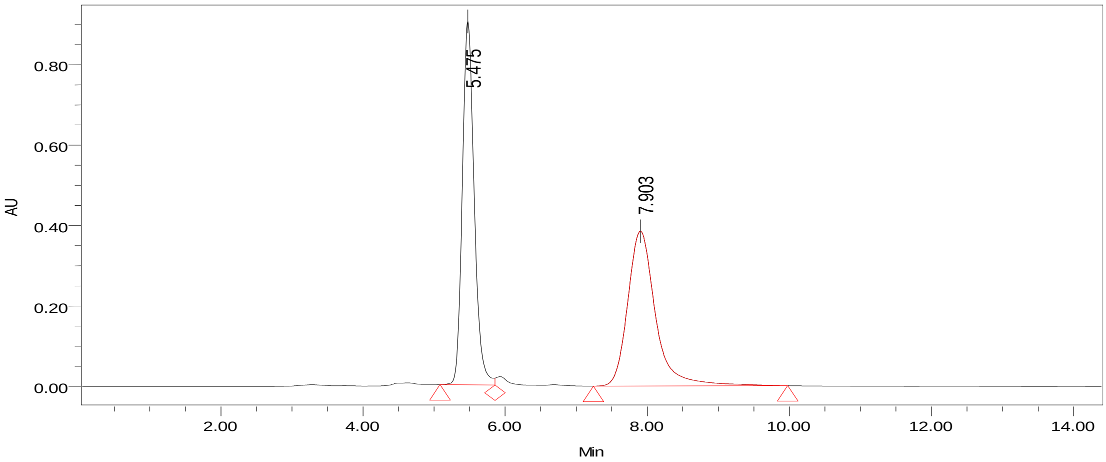

Entry	Retention time	Area	Area (%)	Height	Int type
1	5.475	10510842	50.06	903464	bv
2	7.903	10487197	49.94	385064	bb

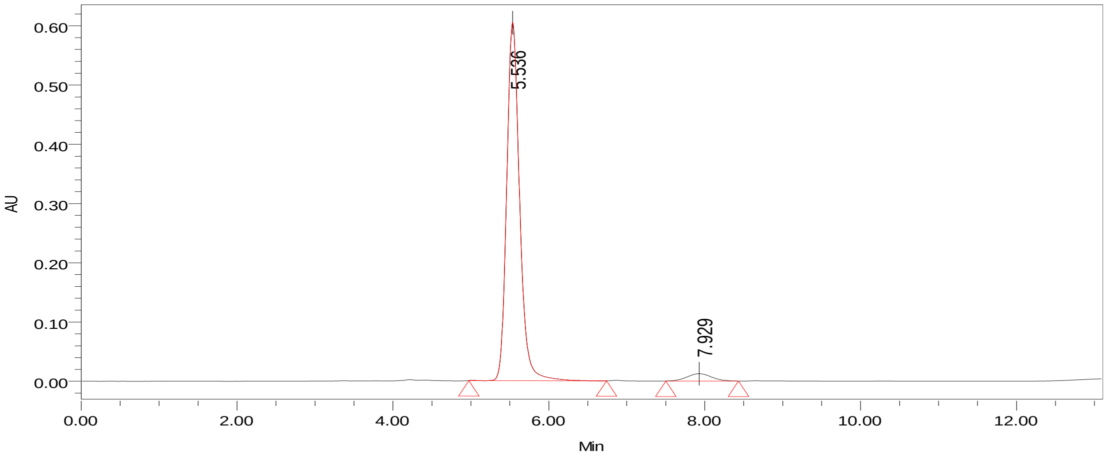

Entry	Retention time	Area	Area (%)	Height	Int type
1	5.536	6966456	96.31	604650	bb
2	7.929	266858	3.69	12358	bb

Supplementary Fig. 22. HPLC chromatograms of compound **3l.**

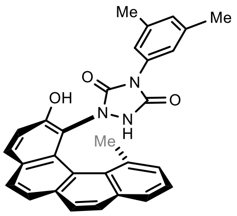

3m

HPLC condition: Chiralcel AD-H, *n*-hexane/*i*-PrOH(1‰ TFA) = 7/3, flow rate = 1.0 mL/min.

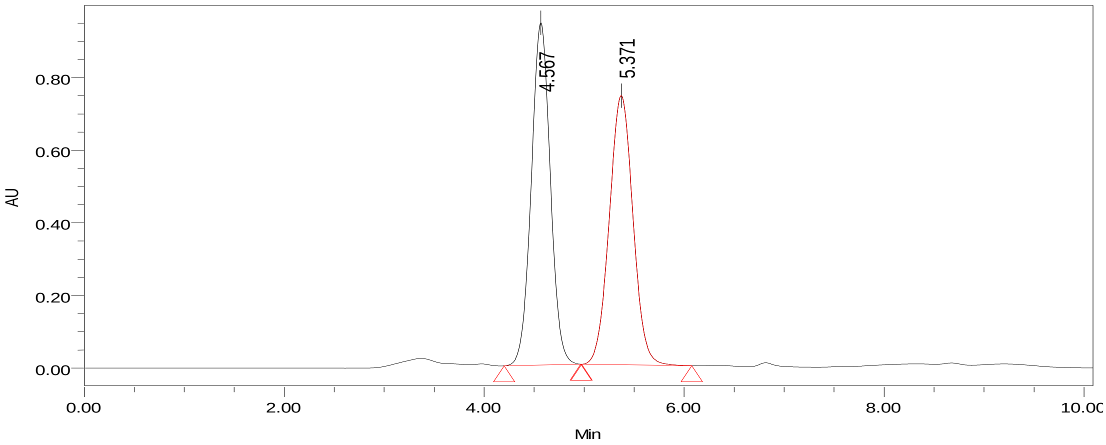

Entry	Retention time	Area	Area (%)	Height	Int type
1	4.567	12480038	50.51	943083	bb
2	5.371	12228998	49.49	741982	bb

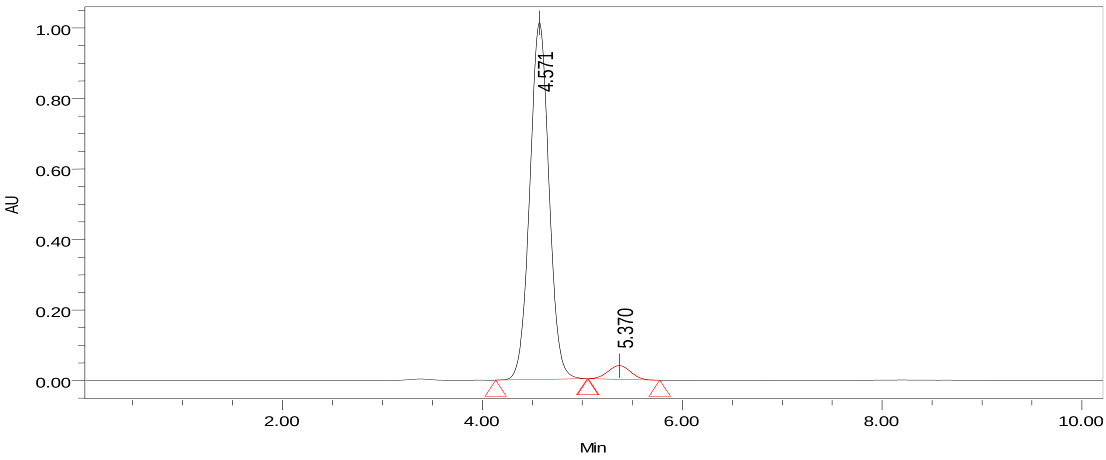

Entry	Retention time	Area	Area (%)	Height	Int type
1	4.571	13543138	95.68	1012570	bb
2	5.370	611026	4.32	38981	bb

Supplementary Fig. 23. HPLC chromatograms of compound **3m.**

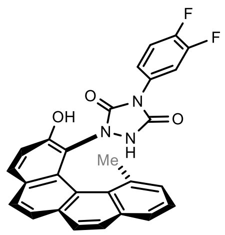

3n

HPLC condition: Chiralcel IA-H, *n*-hexane/*i*-PrOH(1‰ TFA) = 8/2, flow rate = 1.0 mL/min.

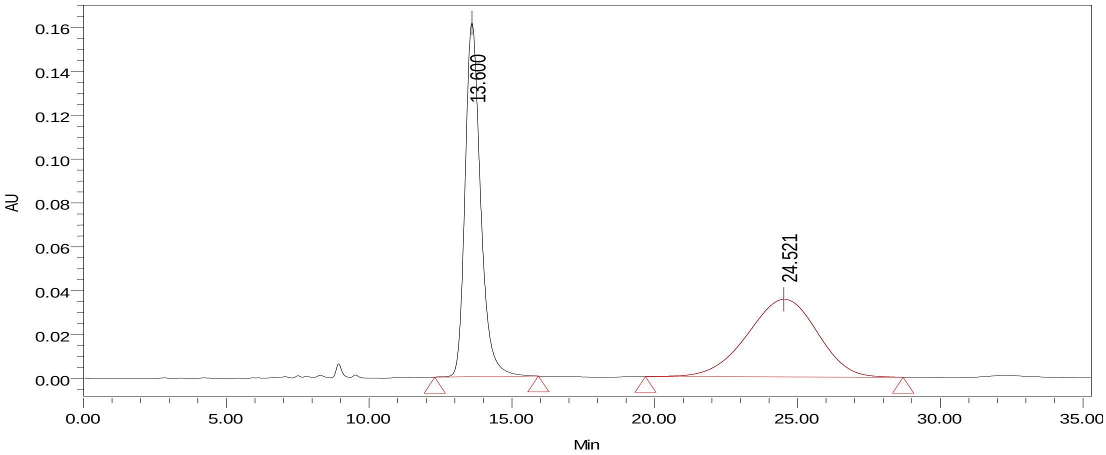

Entry	Retention time	Area	Area (%)	Height	Int type
1	13.600	6118730	50.27	161252	bb
2	24.521	6052165	49.73	35385	bb

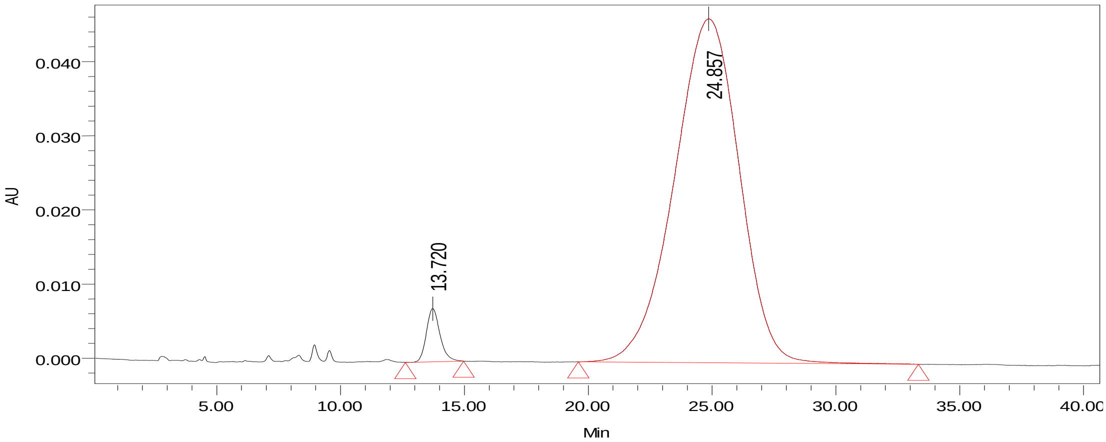

Entry	Retention time	Area	Area (%)	Height	Int type
1	13.720	273701	3.13	7180	bb
2	24.857	8462373	96.87	46376	bb

Supplementary Fig. 24. HPLC chromatograms of compound **3n.**

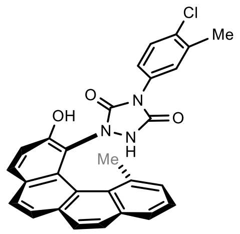

3o

HPLC condition: Chiralcel IA-H, *n*-hexane/*i*-PrOH(1‰ TFA) = 8/2, flow rate = 1.0 mL/min.

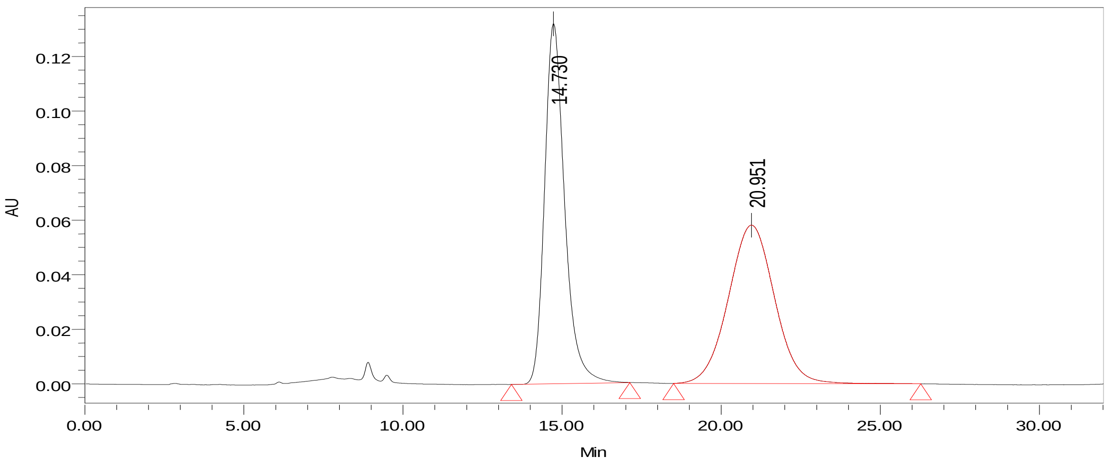

Entry	Retention time	Area	Area (%)	Height	Int type
1	14.730	5781517	49.72	131958	bb
2	20.951	5846381	50.28	58081	bb

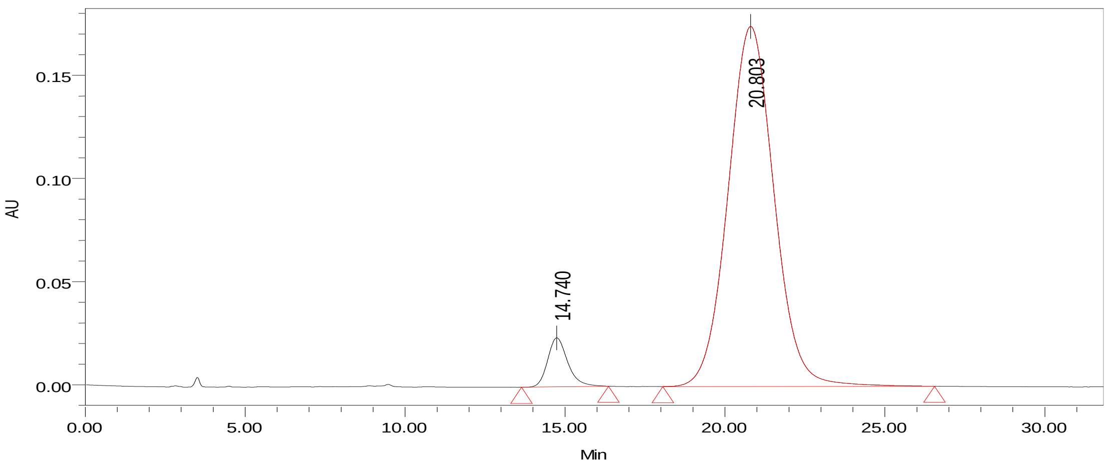

Entry	Retention time	Area	Area (%)	Height	Int type
1	14.740	1036396	5.60	23737	bb
2	20.803	17461327	94.40	174433	bb

Supplementary Fig. 25. HPLC chromatograms of compound **3o.**

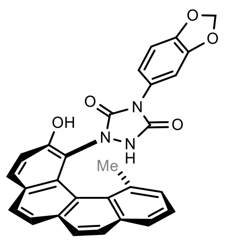

3p

HPLC condition: Chiralcel AD-H, *n*-hexane/*i*-PrOH(1‰ TFA) = 7/3, flow rate = 1.0 mL/min.

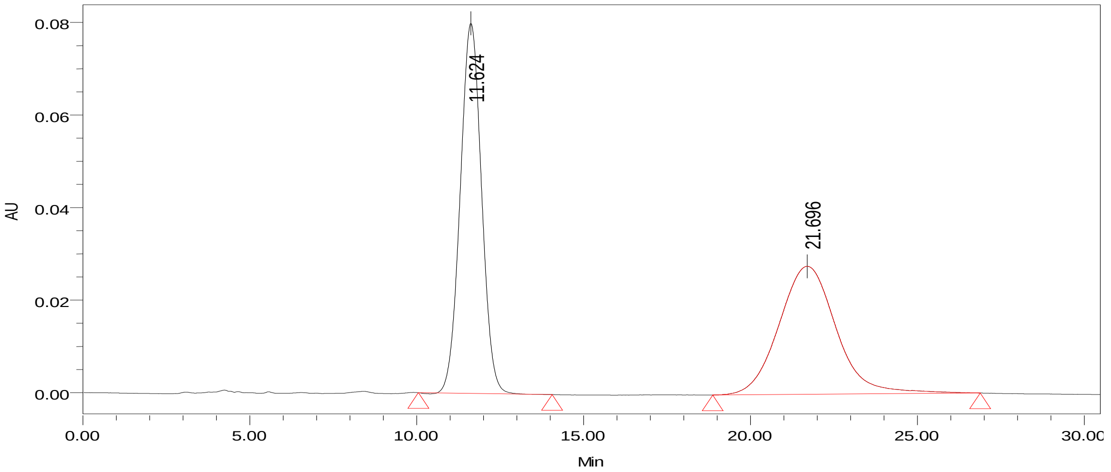

Entry	Retention time	Area	Area (%)	Height	Int type
1	11.624	3557600	51.96	79993	bb
2	21.696	3289611	48.04	27676	bb

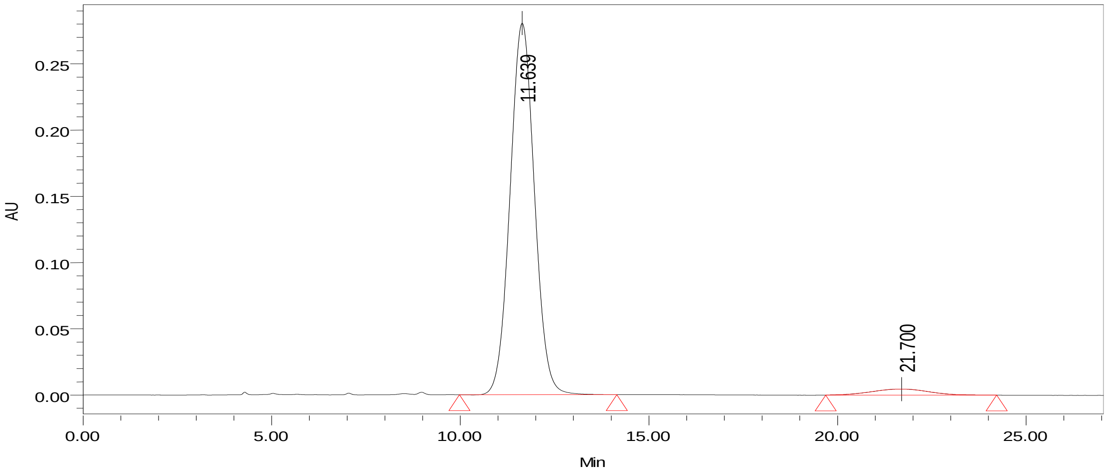

Entry	Retention time	Area	Area (%)	Height	Int type
1	11.639	12603967	96.33	280477	bb
2	21.700	480379	3.67	4603	bb

Supplementary Fig. 26. HPLC chromatograms of compound **3p.**

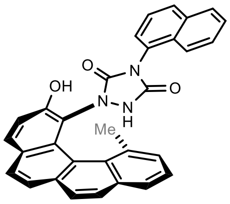

3q

HPLC condition: Chiralcel AD-H, *n*-hexane/*i*-PrOH(1‰ TFA) = 7/3, flow rate = 1.0 mL/min.

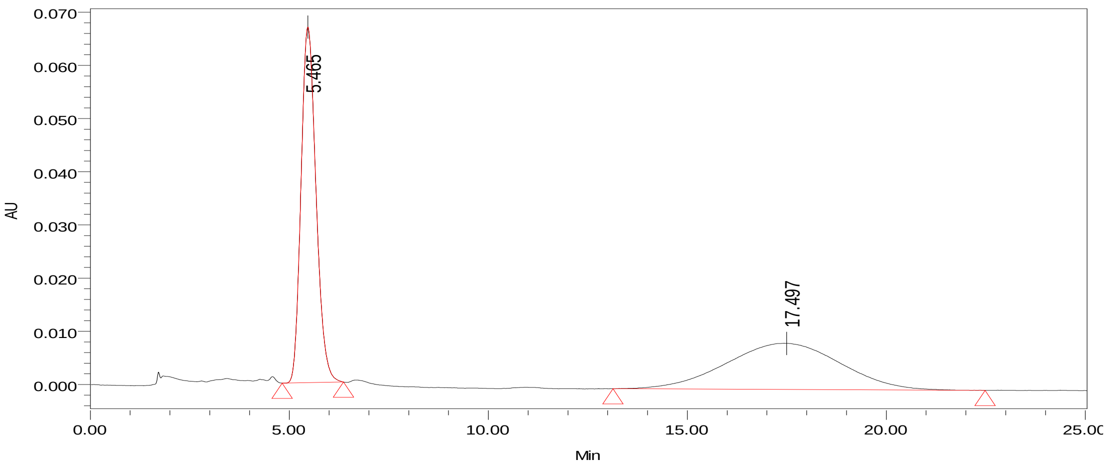

Entry	Retention time	Area	Area (%)	Height	Int type
1	5.465	1752058	50.17	66862	bb
2	17.497	1740229	49.83	8689	bb

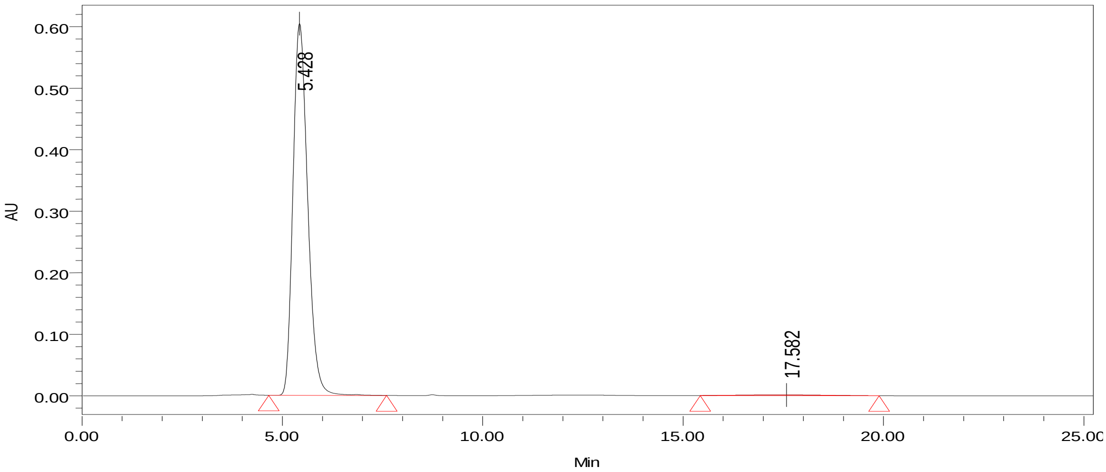

Entry	Retention time	Area	Area (%)	Height	Int type
1	5.428	15423868	98.93	604168	bb
2	17.582	167203	1.07	1100	bb

Supplementary Fig. 27. HPLC chromatograms of compound **3q.**

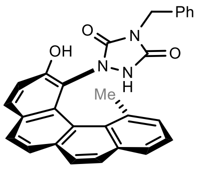

3r

HPLC condition: Chiralcel AD-H, *n*-hexane/*i*-PrOH(1‰ TFA) = 7/3, flow rate = 1.0 mL/min.

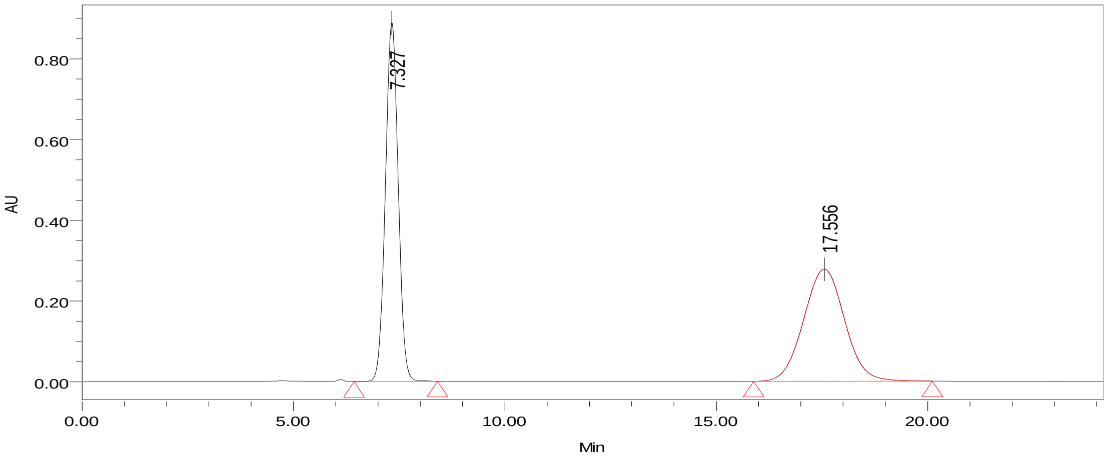

Entry	Retention time	Area	Area (%)	Height	Int type
1	7.327	18756185	50.09	888120	bb
2	17.556	18689013	49.91	277751	bb

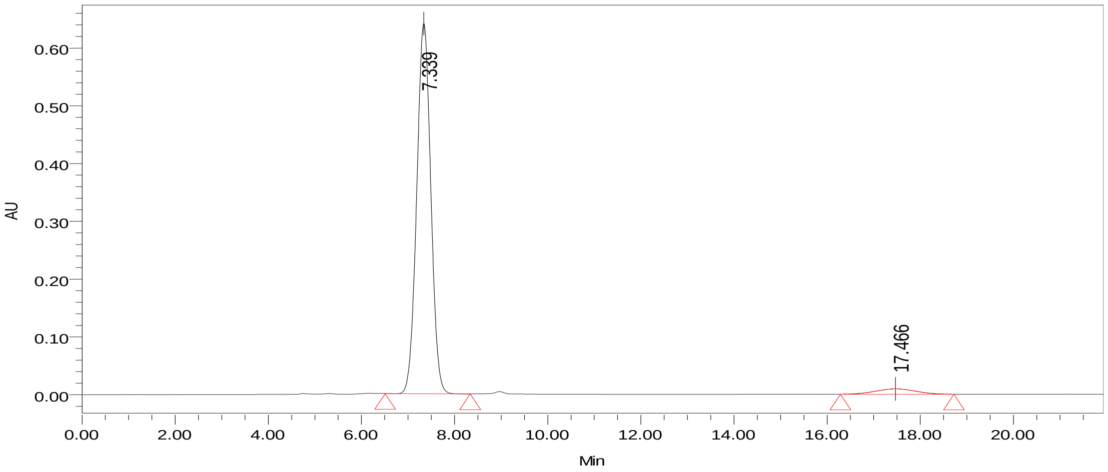

Entry	Retention time	Area	Area (%)	Height	Int type
1	7.339	13464779	96.06	640767	bb
2	17.466	552790	3.94	9290	bb

Supplementary Fig. 28. HPLC chromatograms of compound **3r.**

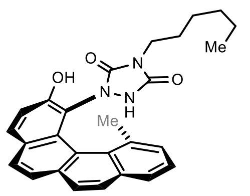

3s

HPLC condition: Chiralcel AD-H, *n*-hexane/*i*-PrOH(1‰ TFA) = 7/3, flow rate = 1.0 mL/min.

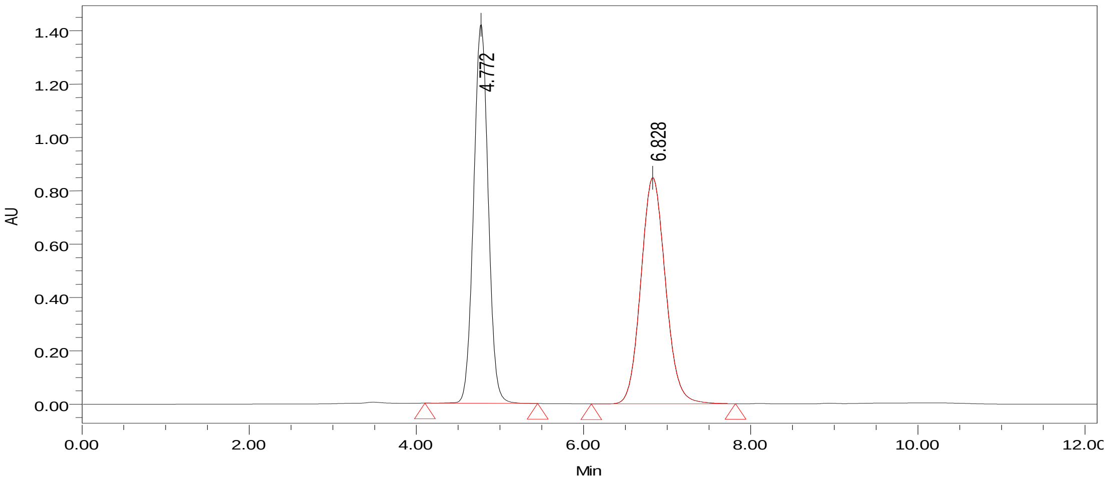

Entry	Retention time	Area	Area (%)	Height	Int type
1	4.772	17207149	49.84	1420057	bb
2	6.828	17321072	50.16	847726	bb

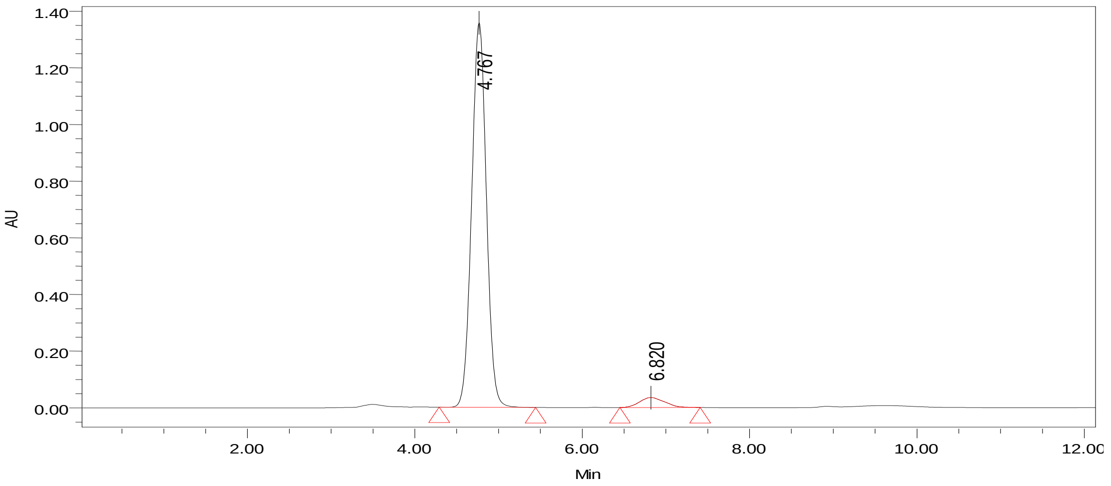

Entry	Retention time	Area	Area (%)	Height	Int type
1	4.767	16579732	95.78	1357616	bb
2	6.820	730404	4.22	35165	bb

Supplementary Fig. 29. HPLC chromatograms of compound **3s.**

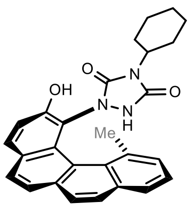

3t

HPLC condition: Chiralcel AD-H, *n*-hexane/*i*-PrOH(1‰ TFA) = 7/3, flow rate = 1.0 mL/min.

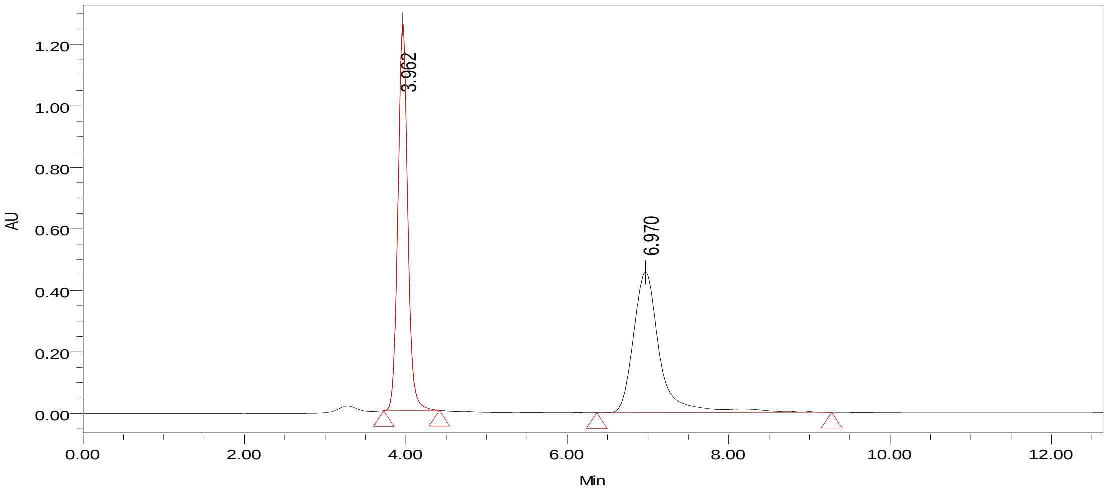

Entry	Retention time	Area	Area (%)	Height	Int type
1	3.962	10582137	50.32	1258804	bb
2	6.970	10446654	49.68	456619	bb

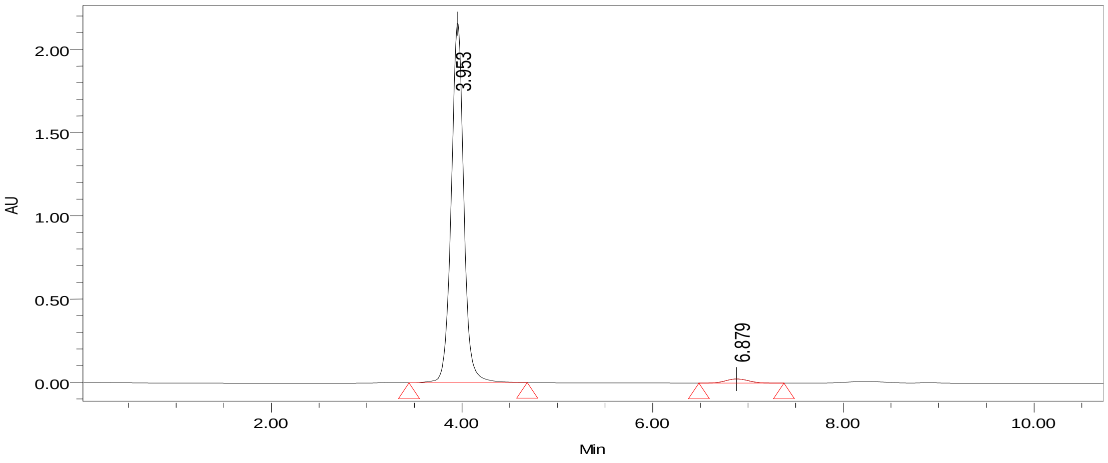

Entry	Retention time	Area	Area (%)	Height	Int type
1	3.953	19790711	97.96	2161296	bb
2	6.879	413024	2.04	24772	bb

Supplementary Fig. 30. HPLC chromatograms of compound **3t.**

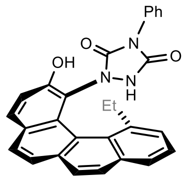

3u

HPLC condition: Chiralcel AD-H, *n*-hexane/*i*-PrOH(1‰ TFA) = 7/3, flow rate = 1.0 mL/min.

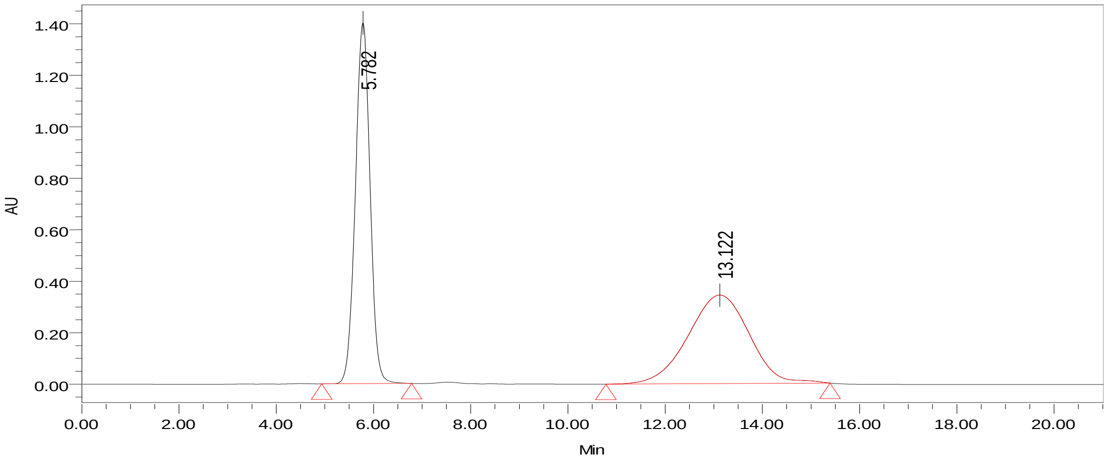

Entry	Retention time	Area	Area (%)	Height	Int type
1	5.782	29466832	49.52	1401872	bb
2	13.122	30040813	50.48	344024	bb

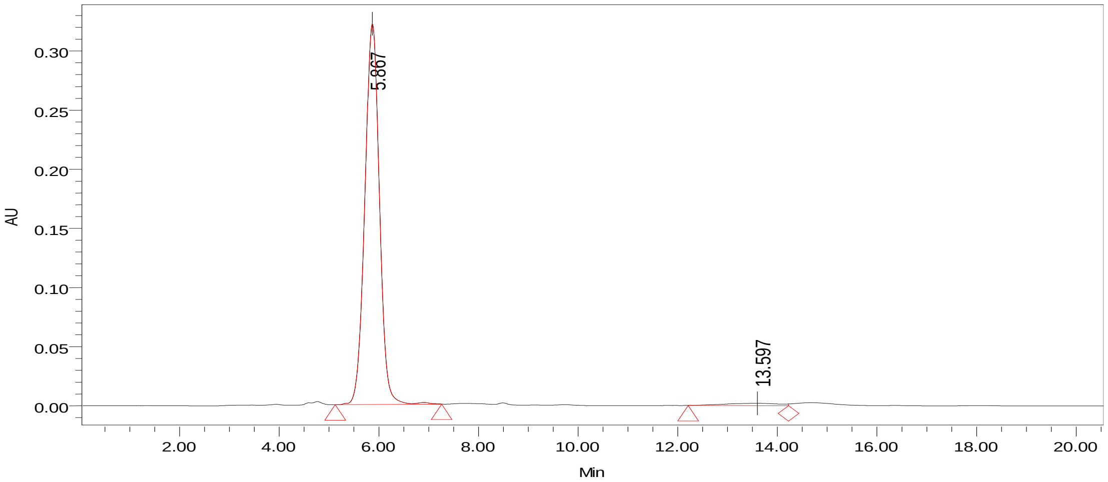

Entry	Retention time	Area	Area (%)	Height	Int type
1	5.867	6515106	97.78	321900	bb
2	13.597	148003	2.22	1943	bv

Supplementary Fig. 31. HPLC chromatograms of compound **3u.**

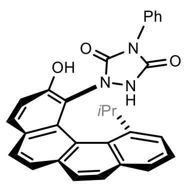

3v

HPLC condition: Chiralcel AD-H, *n*-hexane/*i*-PrOH(1‰ TFA) = 7/3, flow rate = 1.0 mL/min.

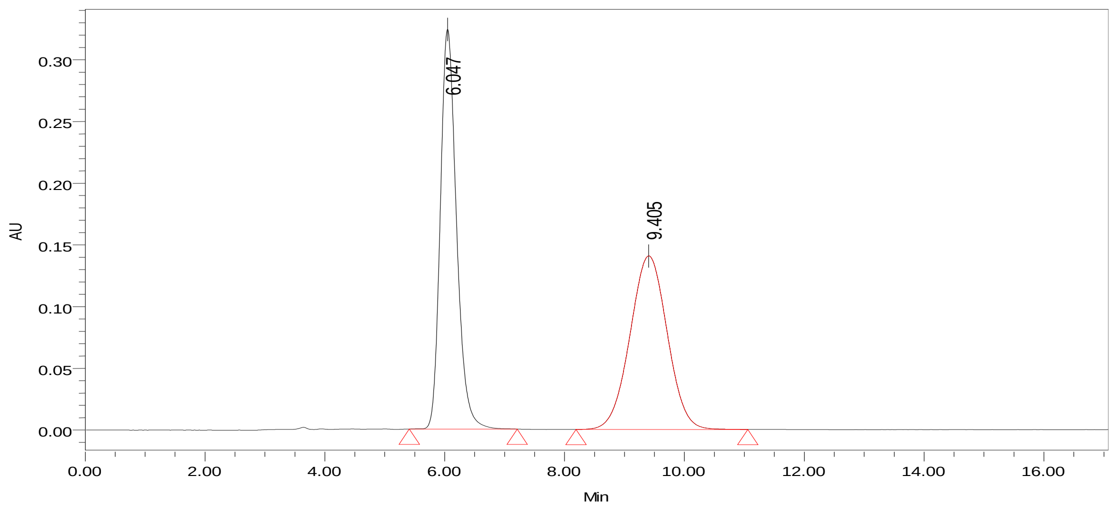

Entry	Retention time	Area	Area (%)	Height	Int type
1	6.047	6073204	50.03	323955	bb
2	9.405	6066106	49.97	140592	bb

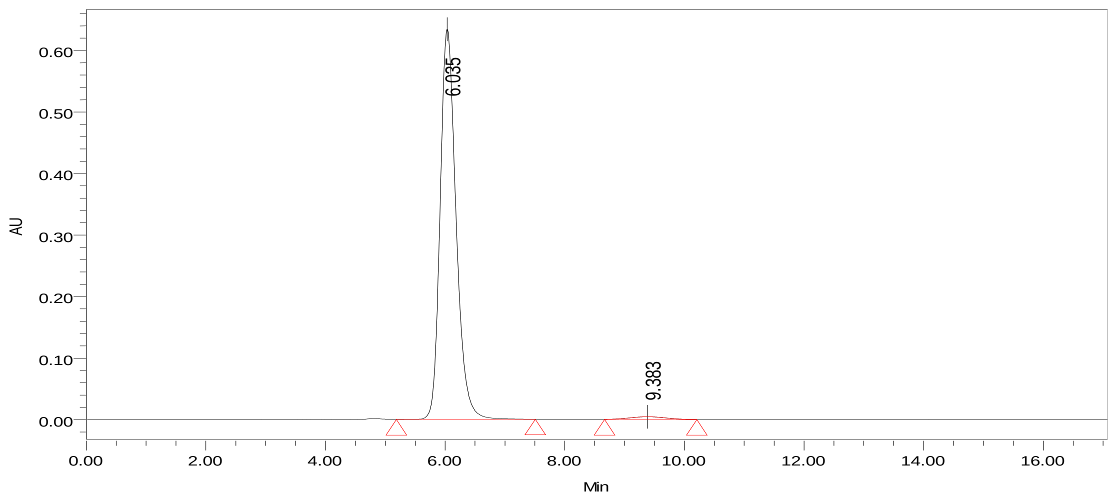

Entry	Retention time	Area	Area (%)	Height	Int type
1	6.035	11742462	98.48	634319	bb
2	9.383	181203	1.52	4455	bb

Supplementary Fig. 32. HPLC chromatograms of compound **3v.**

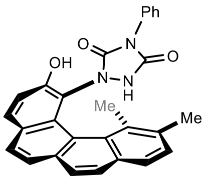

3w

HPLC condition: Chiralcel AD-H, *n*-hexane/*i*-PrOH(1‰ TFA) = 7/3, flow rate = 1.0 mL/min.

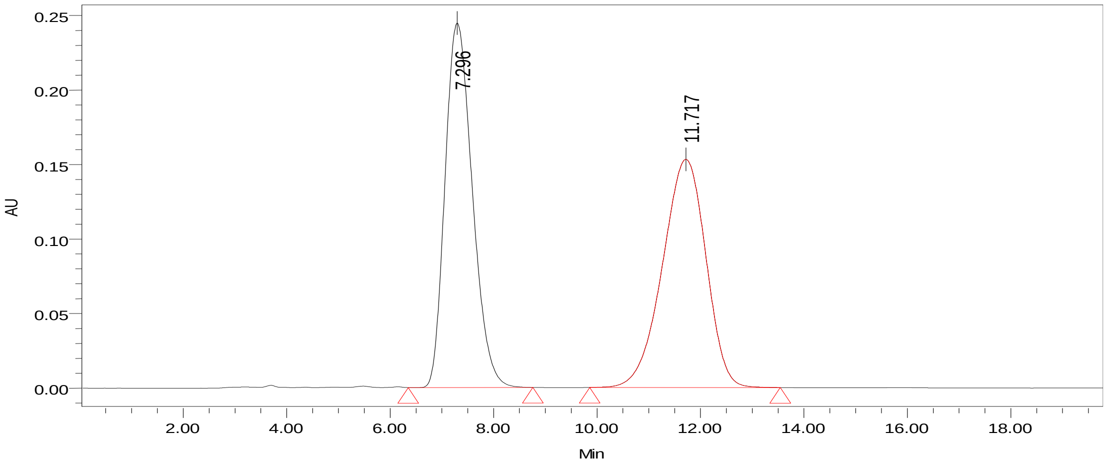

Entry	Retention time	Area	Area (%)	Height	Int type
1	7.296	8971847	50.06	244580	bb
2	11.717	8951571	49.94	153074	bb

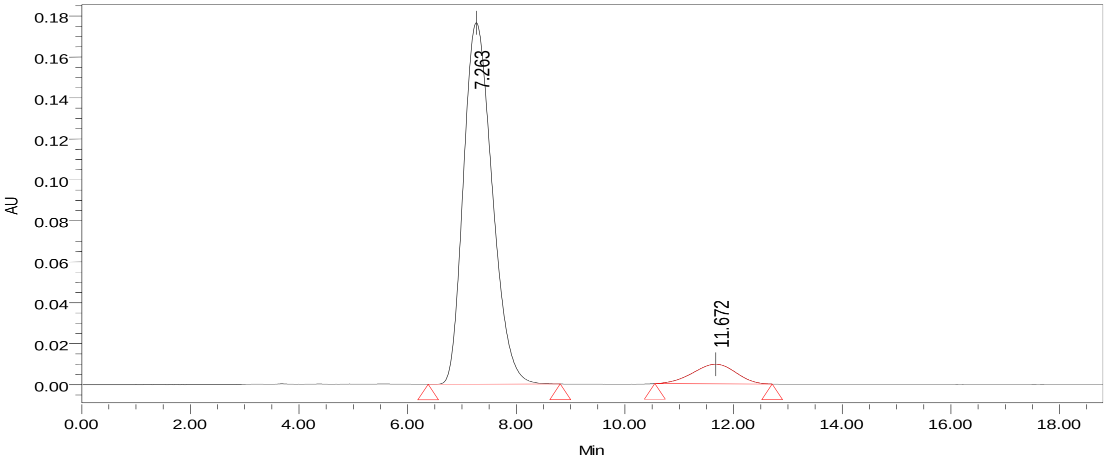

Entry	Retention time	Area	Area (%)	Height	Int type
1	7.263	6520341	92.52	176498	bb
2	11.672	527254	7.48	9579	bb

Supplementary Fig. 33. HPLC chromatograms of compound **3w.**

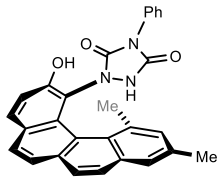

3x

HPLC condition: Chiralcel AD-H, *n*-hexane/*i*-PrOH(1‰ TFA) = 7/3, flow rate = 1.0 mL/min.

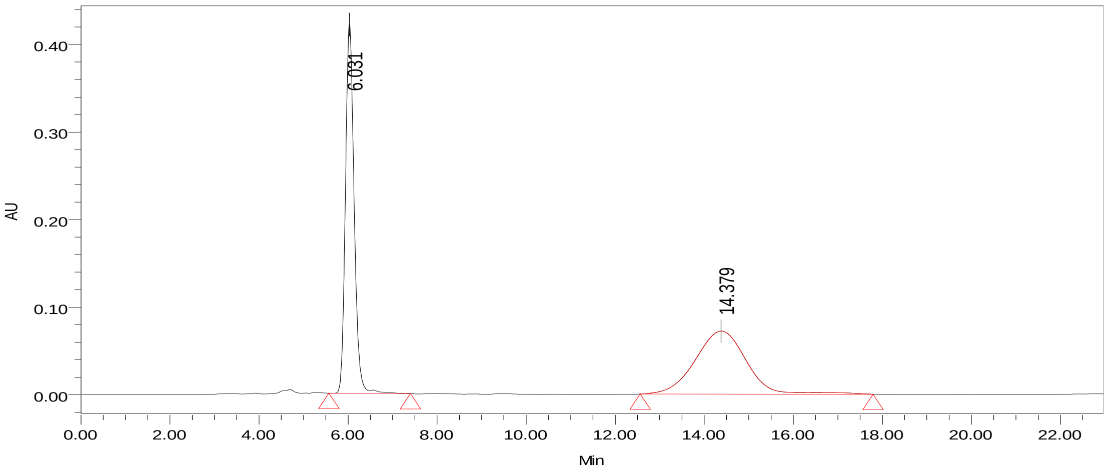

Entry	Retention time	Area	Area (%)	Height	Int type
1	6.031	5741828	49.98	421555	bb
2	14.379	5746750	50.02	71968	bb

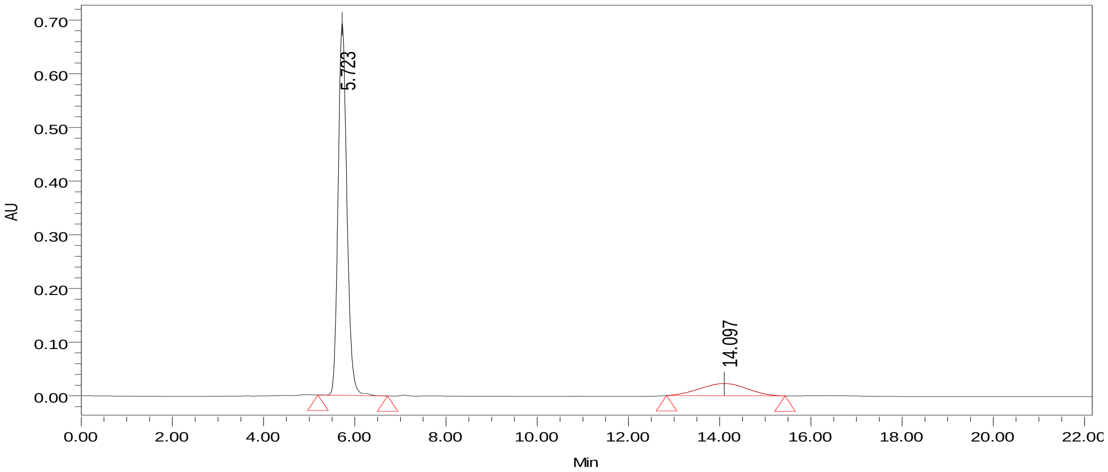

Entry	Retention time	Area	Area (%)	Height	Int type
1	5.723	9335195	85.07	692579	bb
2	14.097	1637994	14.93	22675	bb

Supplementary Fig. 34. HPLC chromatograms of compound **3x.**

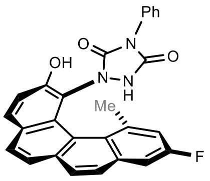

3y

HPLC condition: Chiralcel AD-H, *n*-hexane/*i*-PrOH(1‰ TFA) = 7/3, flow rate = 1.0 mL/min.

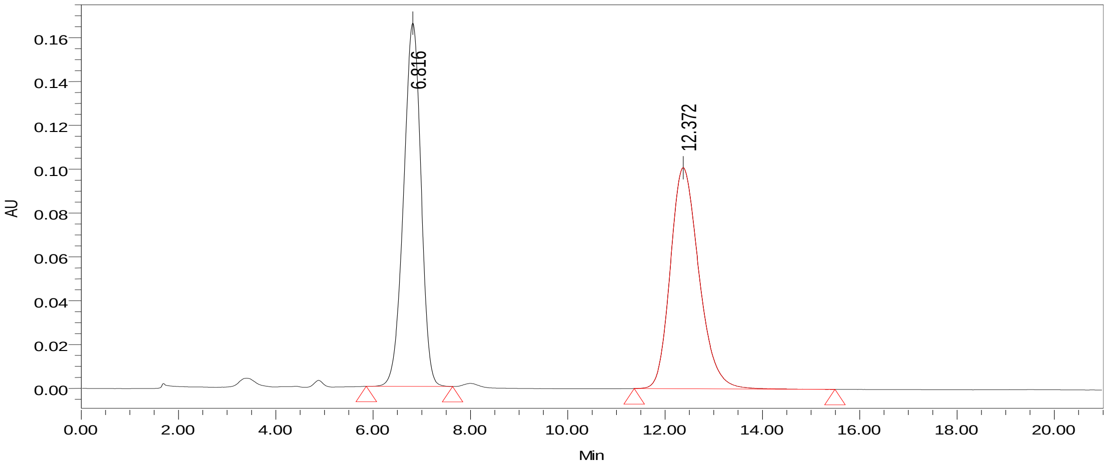

Entry	Retention time	Area	Area (%)	Height	Int type
1	6.816	4129478	50.04	165753	bb
2	12.372	4123517	49.96	100856	bb

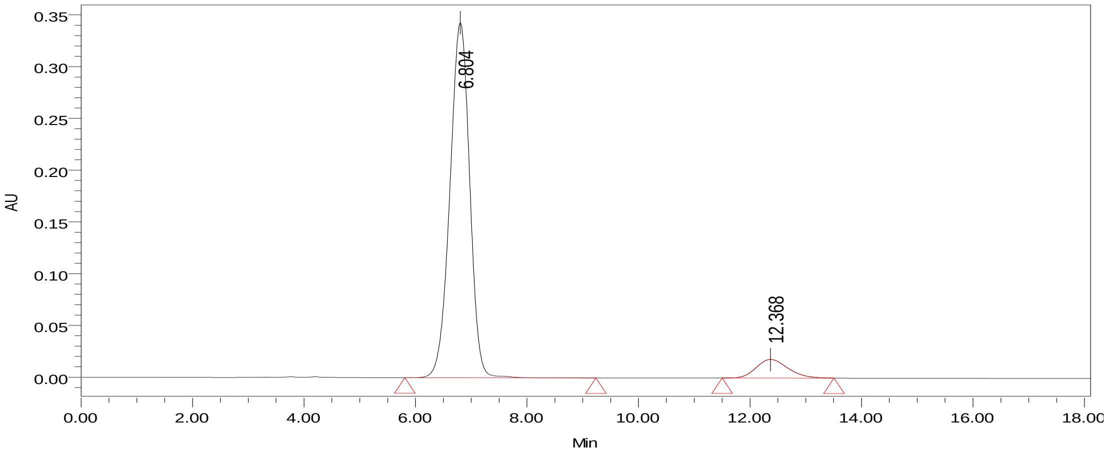

Entry	Retention time	Area	Area (%)	Height	Int type
1	6.804	8451122	92.24	342985	bb
2	12.368	710750	7.76	17986	bb

Supplementary Fig. 35. HPLC chromatograms of compound **3y.**

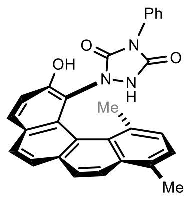

3z

HPLC condition: Chiralcel AD-H, *n*-hexane/*i*-PrOH(1‰ TFA) = 7/3, flow rate = 1.0 mL/min.

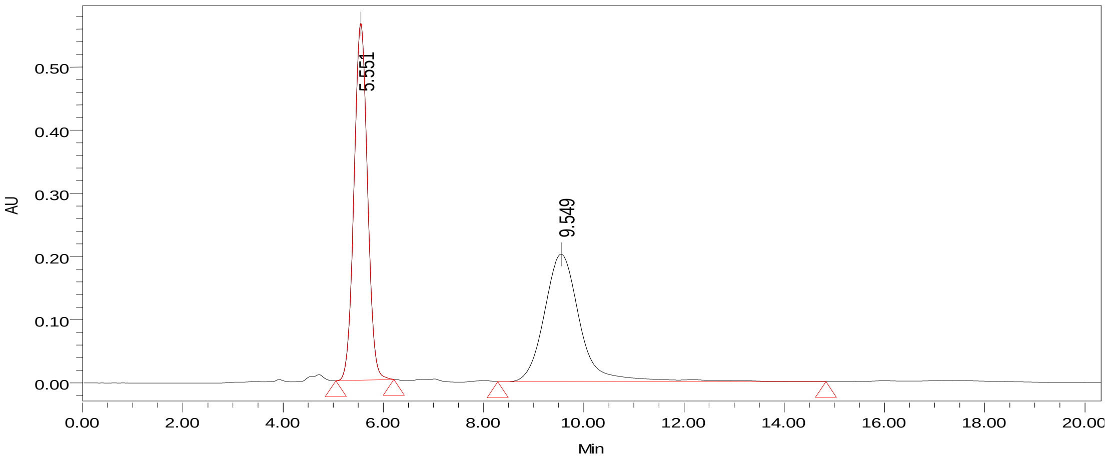

Entry	Retention time	Area	Area (%)	Height	Int type
1	5.551	10542781	51.22	564253	bb
2	9.549	10038900	48.78	201579	bb

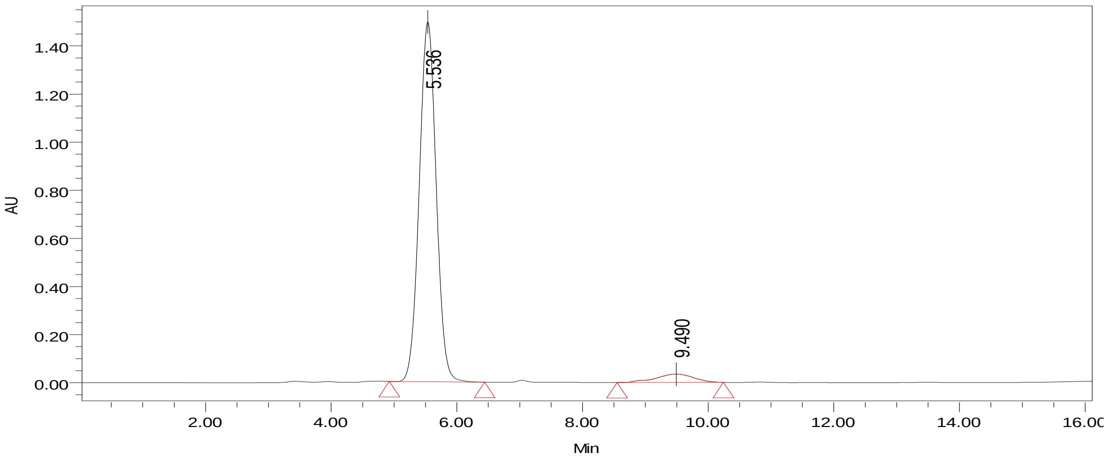

Entry	Retention time	Area	Area (%)	Height	Int type
1	5.536	28040201	95.10	1495701	bb
2	9.490	1443482	4.90	34696	bb

Supplementary Fig. 36. HPLC chromatograms of compound **3z.**

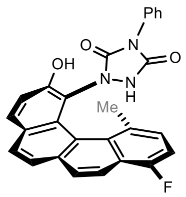

3aa

HPLC condition: Chiralcel AD-H, *n*-hexane/*i*-PrOH(1‰ TFA) = 7/3, flow rate = 1.0 mL/min.

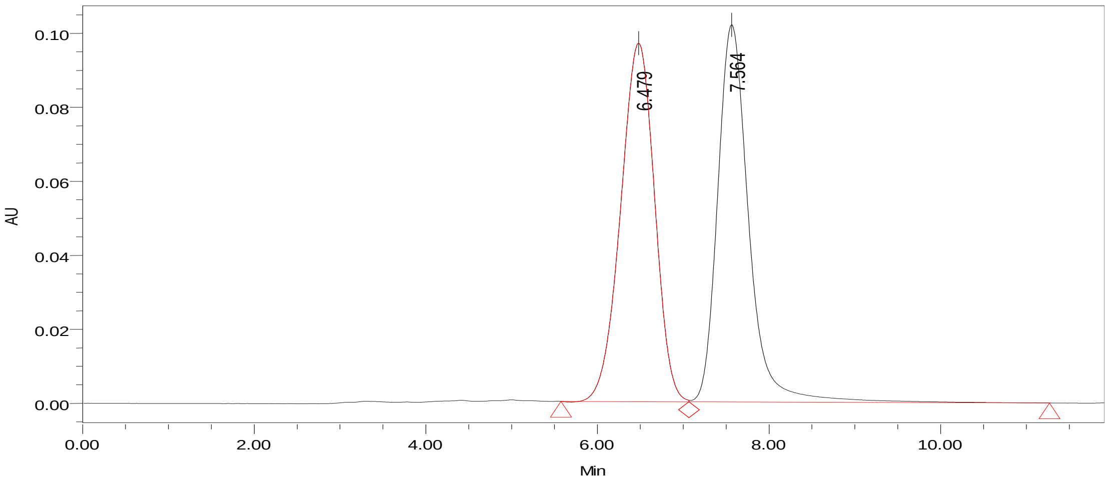

Entry	Retention time	Area	Area (%)	Height	Int type
1	6.479	2666226	50.84	96893	bv
2	7.564	2578096	49.16	101971	vb

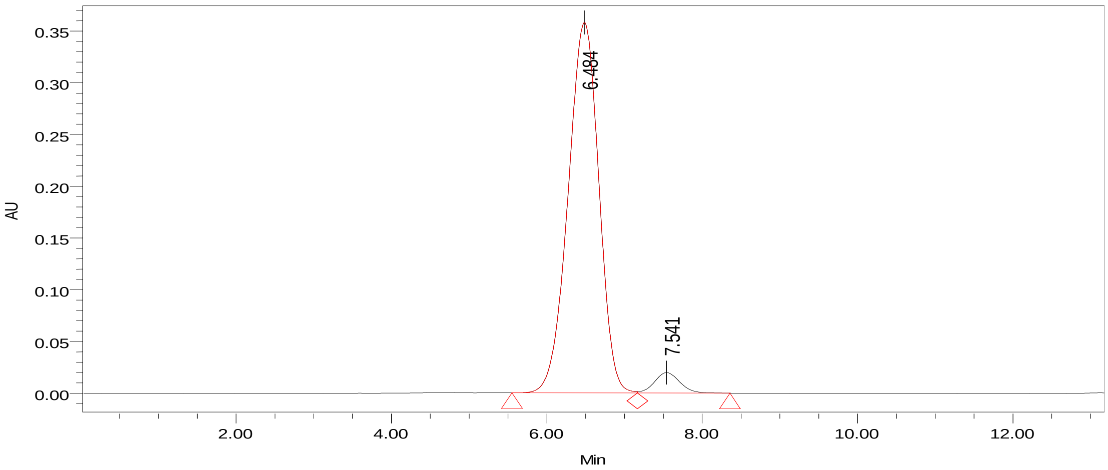

Entry	Retention time	Area	Area (%)	Height	Int type
1	6.484	9862570	95.64	358067	bv
2	7.541	449651	4.36	19750	vb

Supplementary Fig. 37. HPLC chromatograms of compound **3aa.**

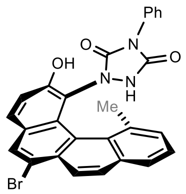

3ab

HPLC condition: Chiralcel AD-H, *n*-hexane/*i*-PrOH(1‰ TFA) = 7/3, flow rate = 1.0 mL/min.

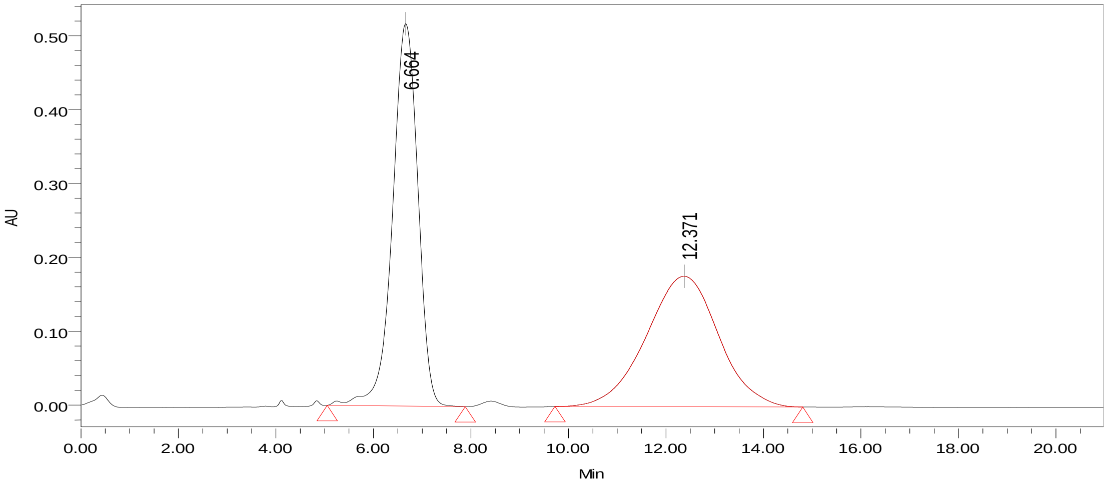

Entry	Retention time	Area	Area (%)	Height	Int type
1	6.664	18818113	50.77	517597	bb
2	12.371	18250054	49.23	176717	bb

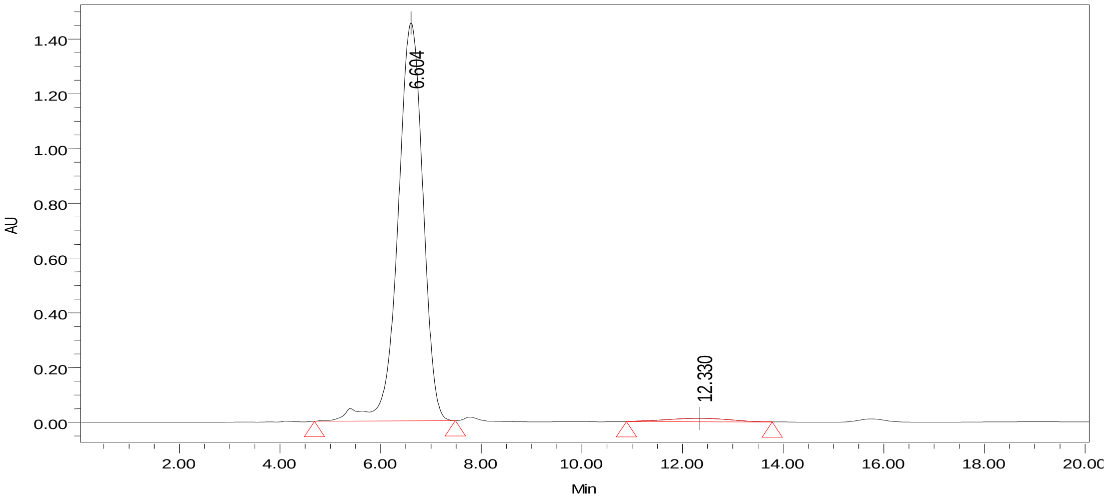

Entry	Retention time	Area	Area (%)	Height	Int type
1	6.604	50699701	97.92	1453708	bb
2	12.330	1076597	2.08	12147	bb

Supplementary Fig. 38. HPLC chromatograms of compound **3ab.**

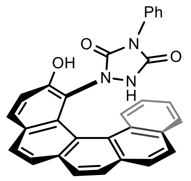

3ac

HPLC condition: Chiralcel IA-H, *n*-hexane/*i*-PrOH(1‰ TFA) = 8/2, flow rate = 1.0 mL/min.

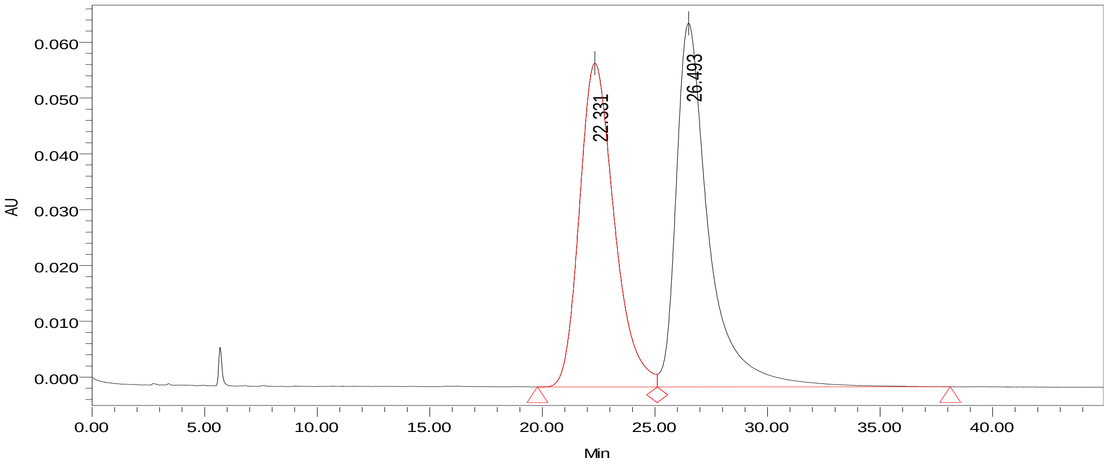

Entry	Retention time	Area	Area (%)	Height	Int type
1	22.331	6238549	48.72	58007	bv
2	26.493	6567131	51.28	65166	vb

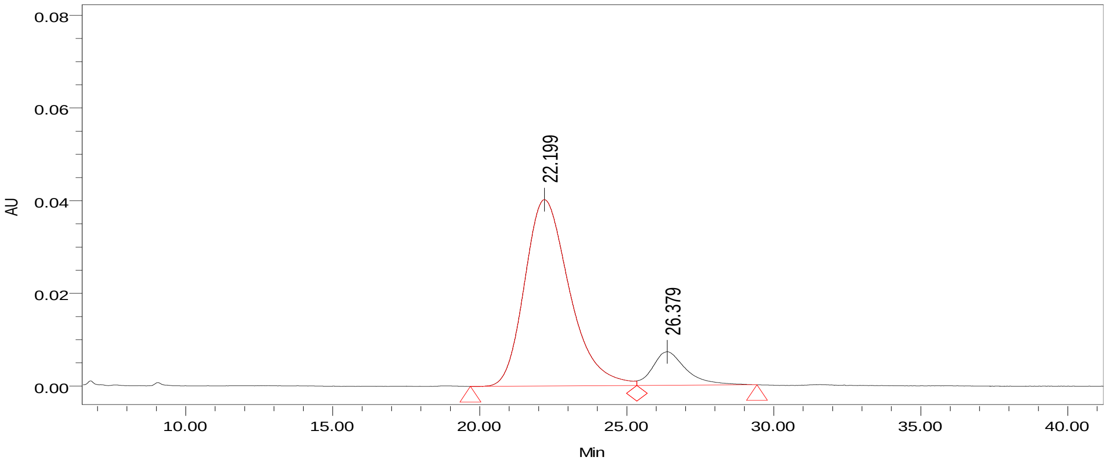

Entry	Retention time	Area	Area (%)	Height	Int type
1	22.199	4271064	88.13	40228	bv
2	26.379	575205	11.87	7244	vb

Supplementary Fig. 39. HPLC chromatograms of compound **3ac.**

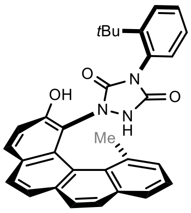

3ad

HPLC condition: Chiralcel AD-H, *n*-hexane/*i*-PrOH(1‰ TFA) = 7/3, flow rate = 1.0 mL/min.

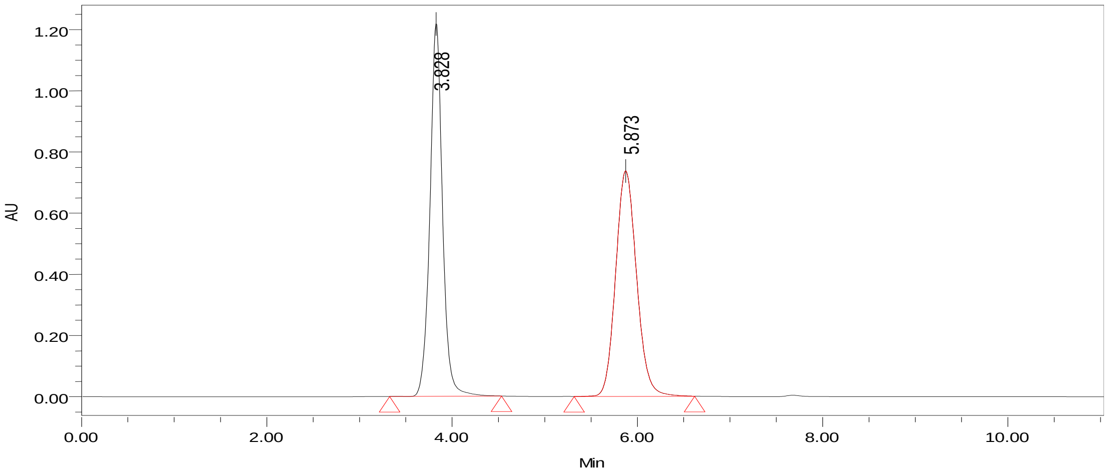

Entry	Retention time	Area	Area (%)	Height	Int type
1	3.828	11490073	49.81	1219802	bb
2	5.873	11575581	50.19	737347	bb

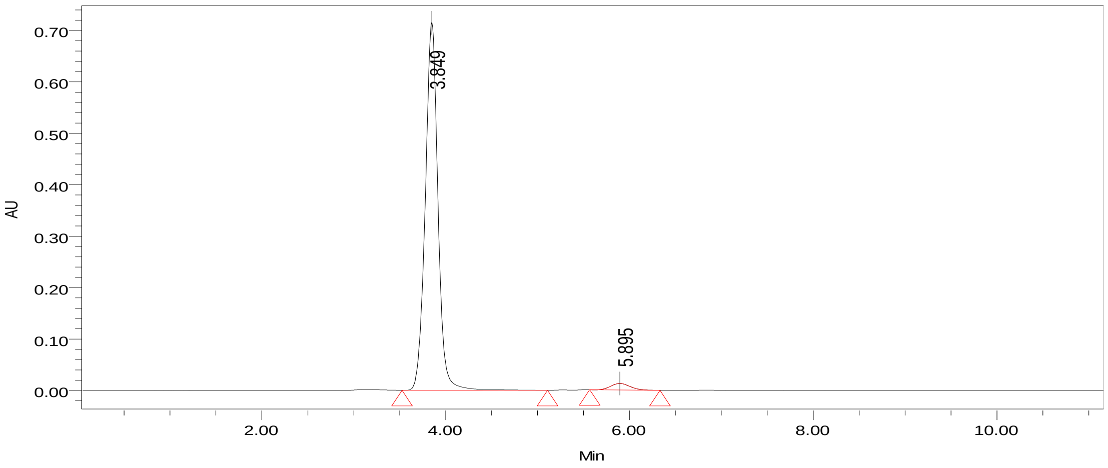

Entry	Retention time	Area	Area (%)	Height	Int type
1	3.849	6870149	97.38	715419	bb
2	5.895	185072	2.62	12677	bb

Supplementary Fig. 40. HPLC chromatograms of compound **3ad.**

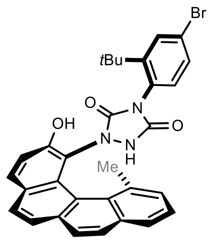

3ae

HPLC condition: Chiralcel AD-H, *n*-hexane/*i*-PrOH(1‰ TFA) = 7/3, flow rate = 1.0 mL/min.

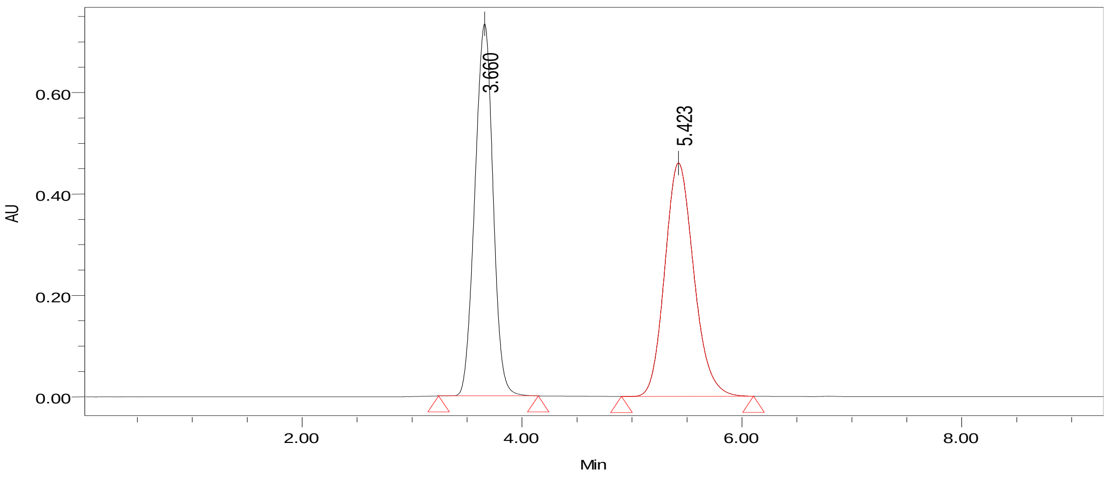

Entry	Retention time	Area	Area (%)	Height	Int type
1	3.660	8423418	49.99	733430	bb
2	5.423	8428067	50.01	460264	bb

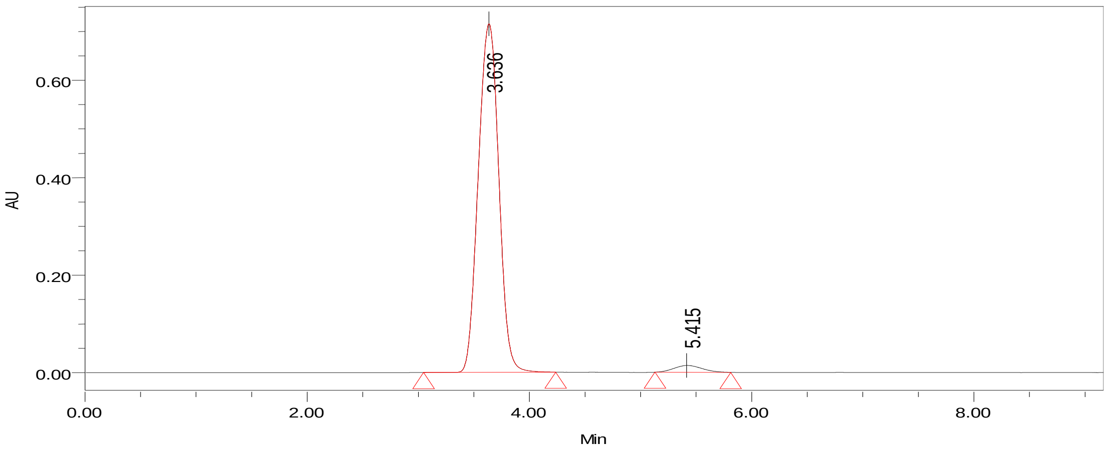

Entry	Retention time	Area	Area (%)	Height	Int type
1	3.636	9181026	97.41	714998	bb
2	5.415	243685	2.59	13986	bb

Supplementary Fig. 41. HPLC chromatograms of compound **3ae.**

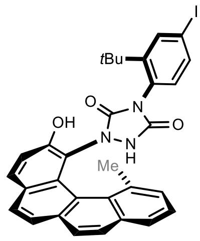

3af

HPLC condition: Chiralcel AD-H, *n*-hexane/*i*-PrOH(1‰ TFA) = 7/3, flow rate = 1.0 mL/min.

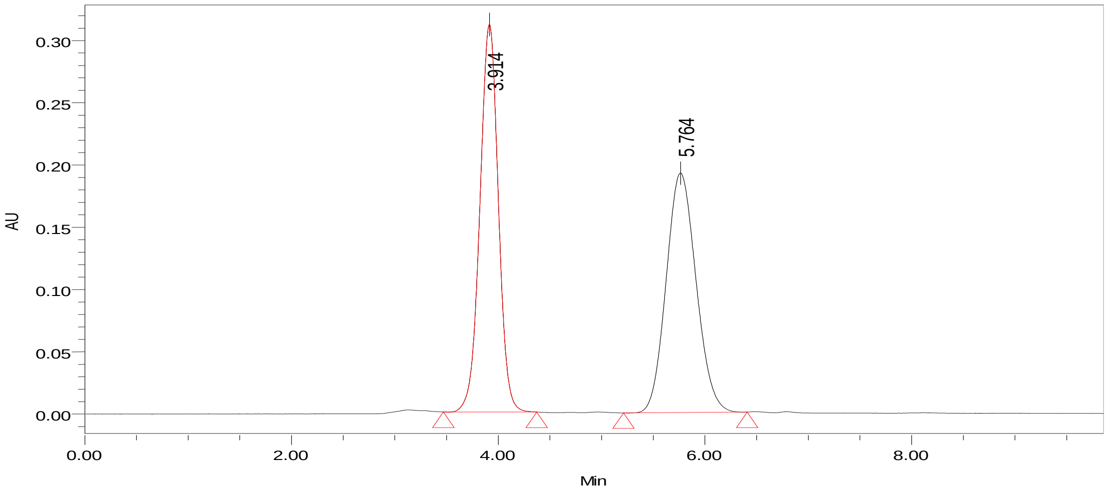

Entry	Retention time	Area	Area (%)	Height	Int type
1	3.914	3866242	49.97	311334	bb
2	5.764	3871253	50.03	192431	bb

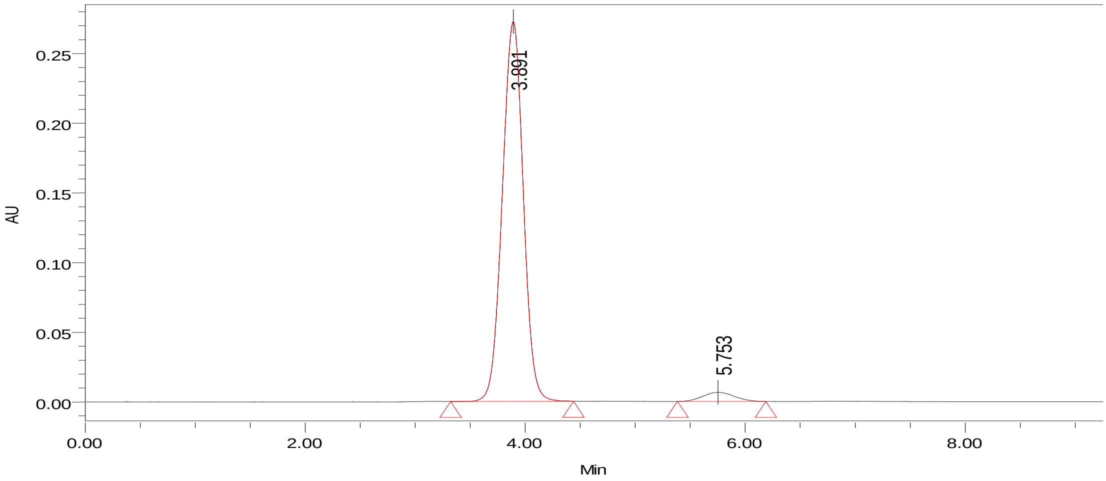

Entry	Retention time	Area	Area (%)	Height	Int type
1	3.891	3604255	96.52	272500	bb
2	5.753	129806	3.48	6590	bb

Supplementary Fig. 42. HPLC chromatograms of compound **3af.**

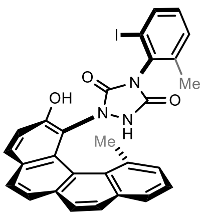

3ag

HPLC condition: Chiralcel IA-H, *n*-hexane/*i*-PrOH(1‰ TFA) = 7/3, flow rate = 1.0 mL/min.

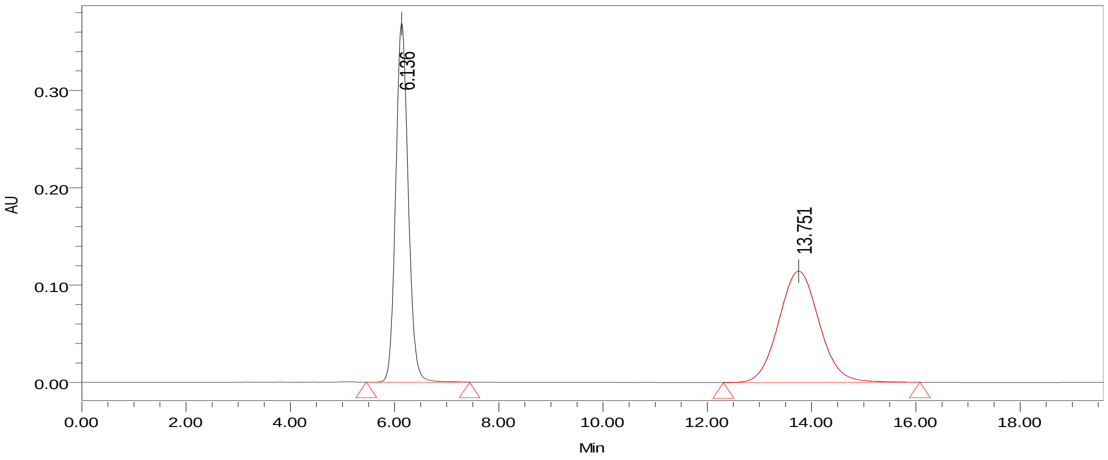

Entry	Retention time	Area	Area (%)	Height	Int type
1	6.136	6167177	50.02	368737	bb
2	13.751	6161188	49.98	114314	bb

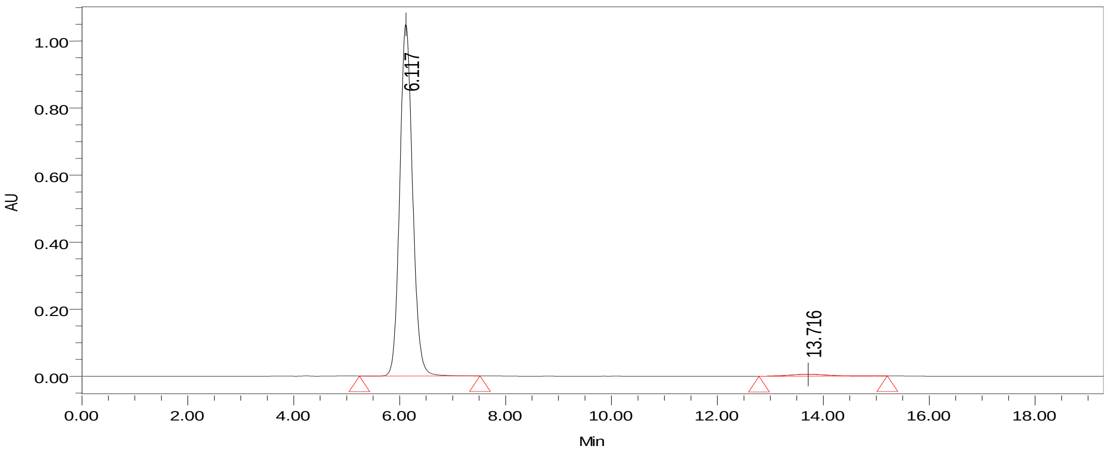

Entry	Retention time	Area	Area (%)	Height	Int type
1	6.117	17678412	98.48	1048877	bb
2	13.716	272438	1.52	5424	bb

Supplementary Fig. 43. HPLC chromatograms of compound **3ag.**

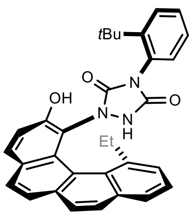

3ah

HPLC condition: Chiralcel IA-H, *n*-hexane/*i*-PrOH(1‰ TFA) = 7/3, flow rate = 1.0 mL/min.

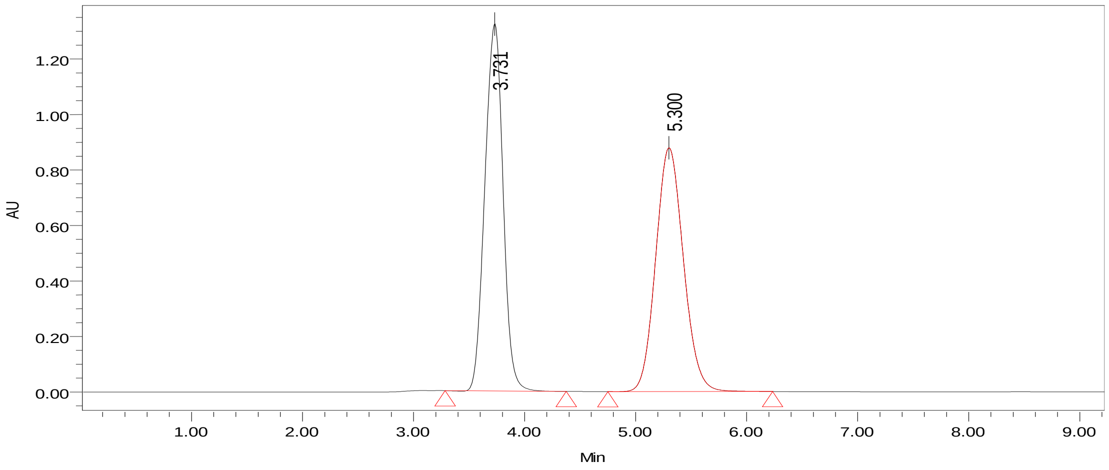

Entry	Retention time	Area	Area (%)	Height	Int type
1	3.731	15089614	49.75	1324094	bb
2	5.300	15239226	50.25	879023	bb

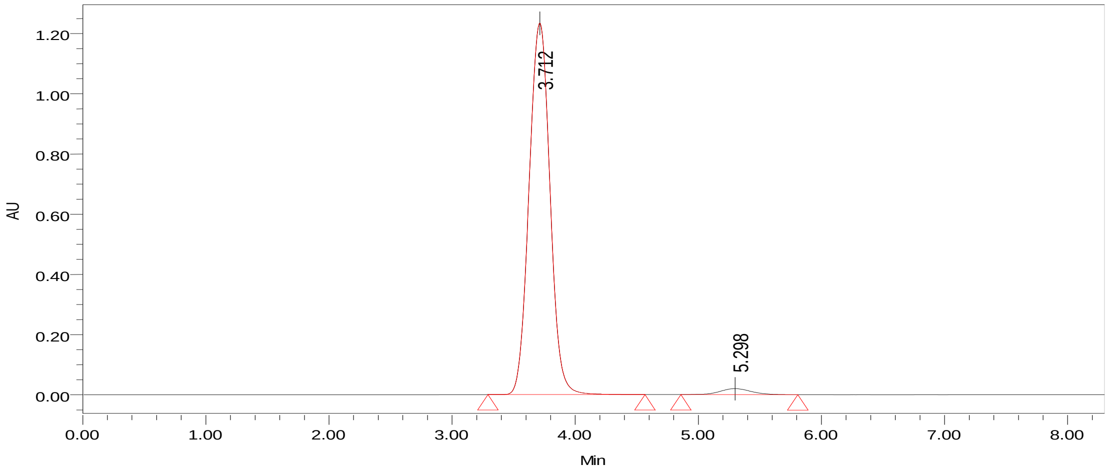

Entry	Retention time	Area	Area (%)	Height	Int type
1	3.712	14564239	97.63	1234987	bb
2	5.298	352856	2.37	19988	bb

Supplementary Fig. 44. HPLC chromatograms of compound **3ah.**

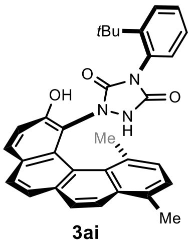

3ai

HPLC condition: Chiralcel IA-H, *n*-hexane/*i*-PrOH(1‰ TFA) = 7/3, flow rate = 1.0 mL/min.

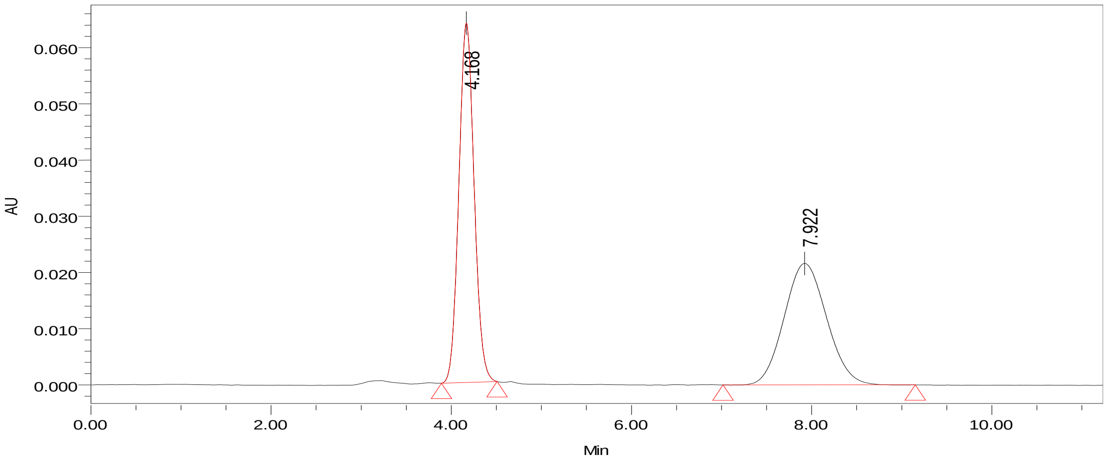

Entry	Retention time	Area	Area (%)	Height	Int type
1	4.168	755147	51.60	63929	bb
2	7.922	708207	48.40	21609	bb

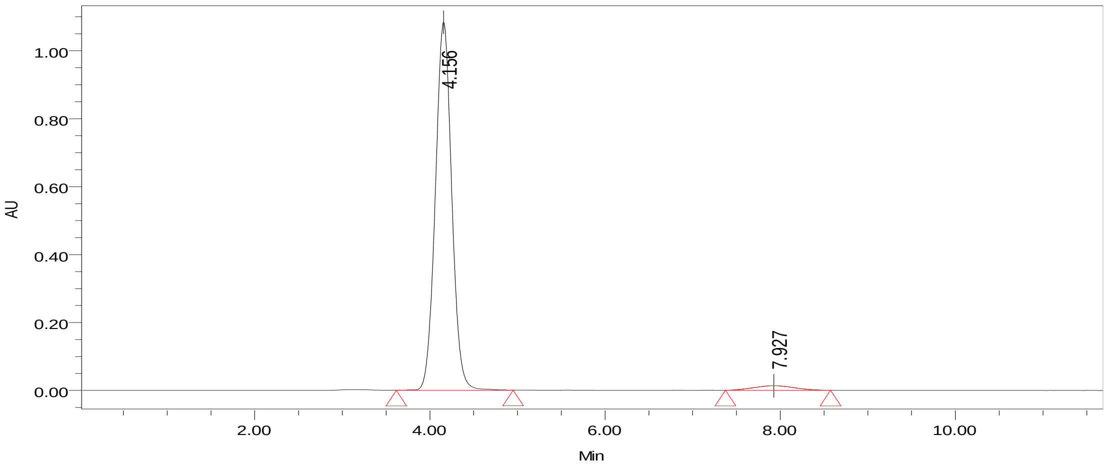

Entry	Retention time	Area	Area (%)	Height	Int type
1	4.156	14064143	97.00	1083548	bb
2	7.927	435118	3.00	13430	bb

Supplementary Fig. 45. HPLC chromatograms of compound **3ai.**

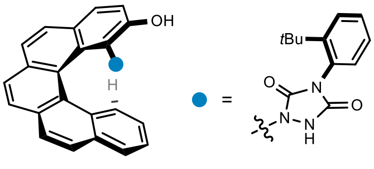

3aj

HPLC condition: Chiralcel AD-H, *n*-hexane/*i*-PrOH(1‰ TFA) = 7/3, flow rate = 1.0 mL/min.

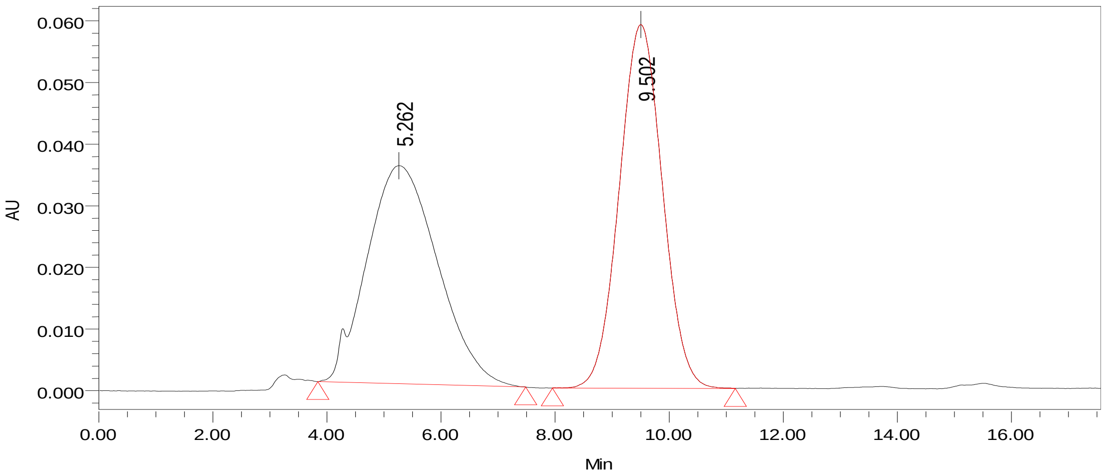

Entry	Retention time	Area	Area (%)	Height	Int type
1	5.262	3061047	49.68	35382	bb
2	9.502	3100839	50.32	58989	bb

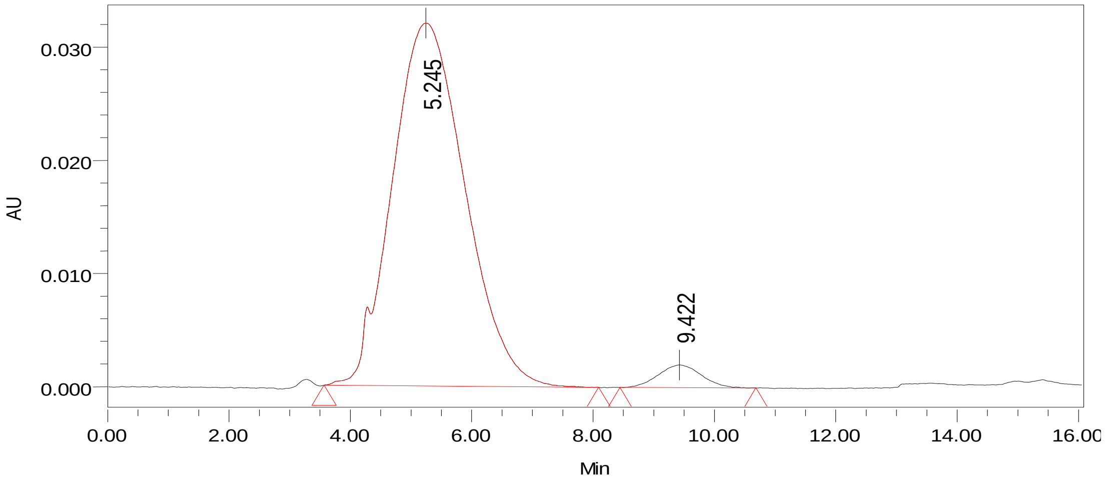

Entry	Retention time	Area	Area (%)	Height	Int type
1	5.245	2673320	96.46	32063	bb
2	9.422	98003	3.54	1998	bb

Supplementary Fig. 46. HPLC chromatograms of compound **3aj.**

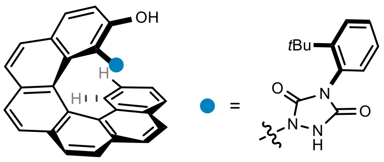

3ak

HPLC condition: Chiralcel OD-H, *n*-hexane/*i*-PrOH(1‰ TFA) = 7/3, flow rate = 1.0 mL/min.

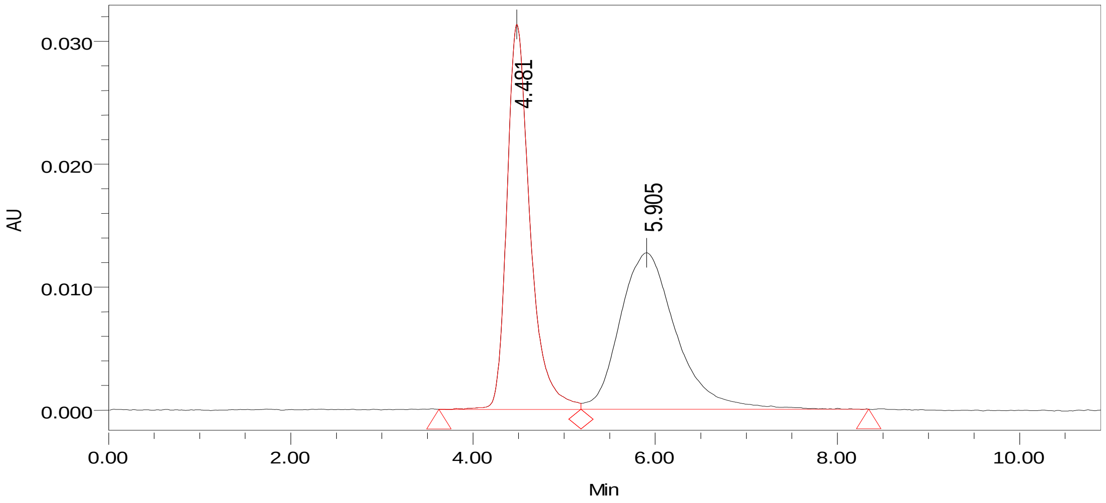

Entry	Retention time	Area	Area (%)	Height	Int type
1	4.481	556543	50.42	31316	bv
2	5.905	547175	49.58	12726	vb

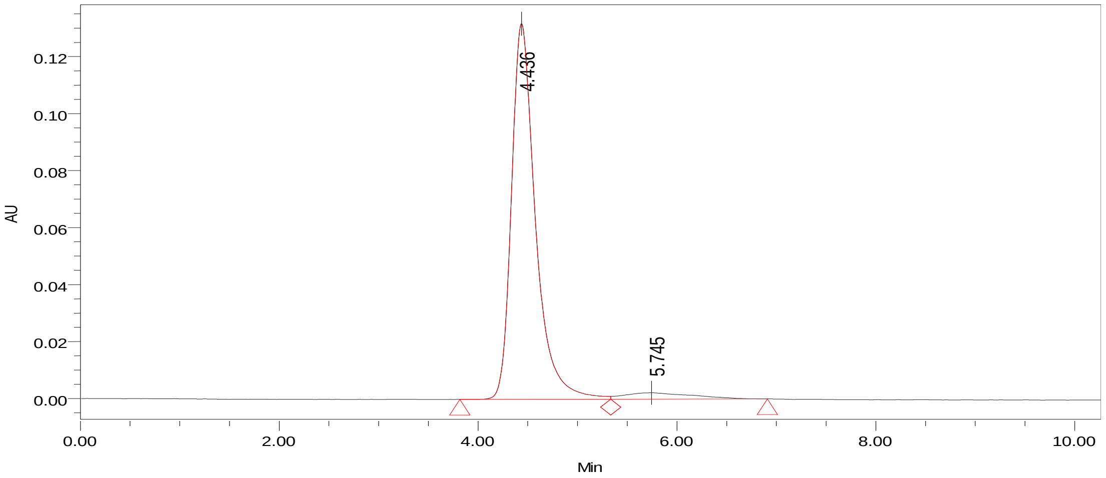

Entry	Retention time	Area	Area (%)	Height	Int type
1	4.436	2213152	95.47	131937	bv
2	5.745	105126	4.53	2225	vb

Supplementary Fig. 47. HPLC chromatograms of compound **3ak.**

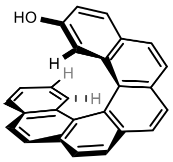

1k

HPLC condition: Chiralcel IA-H, *n*-hexane/*i*-PrOH(1‰ TFA) = 8/2, flow rate = 1.0 mL/min.

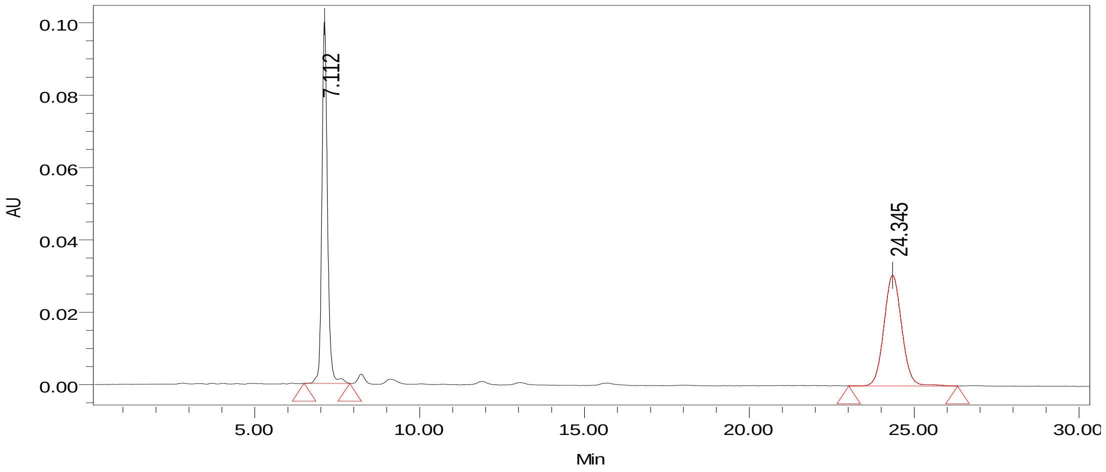

Entry	Retention time	Area	Area (%)	Height	Int type
1	7.112	1181939	50.70	100126	bb
2	24.345	1149363	49.30	30583	bb

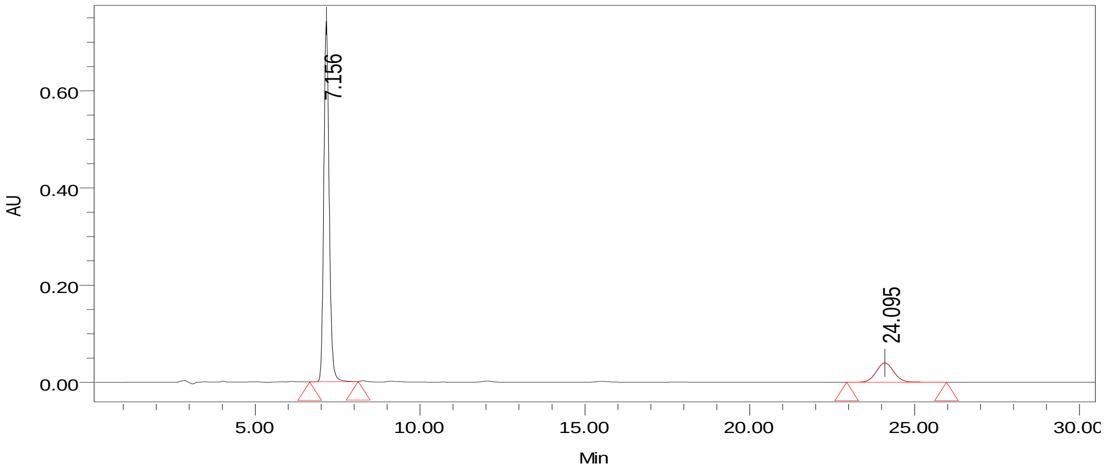

Entry	Retention time	Area	Area (%)	Height	Int type
1	7.156	8394104	85.11	743394	bb
2	24.095	1469126	14.89	39733	bb

Supplementary Fig. 48. HPLC chromatograms of compound **1k.**

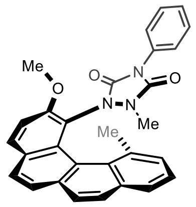

4a

HPLC condition: Chiralcel IA-H, *n*-hexane/*i*-PrOH(1‰ TFA) = 7/3, flow rate = 1.0 mL/min.

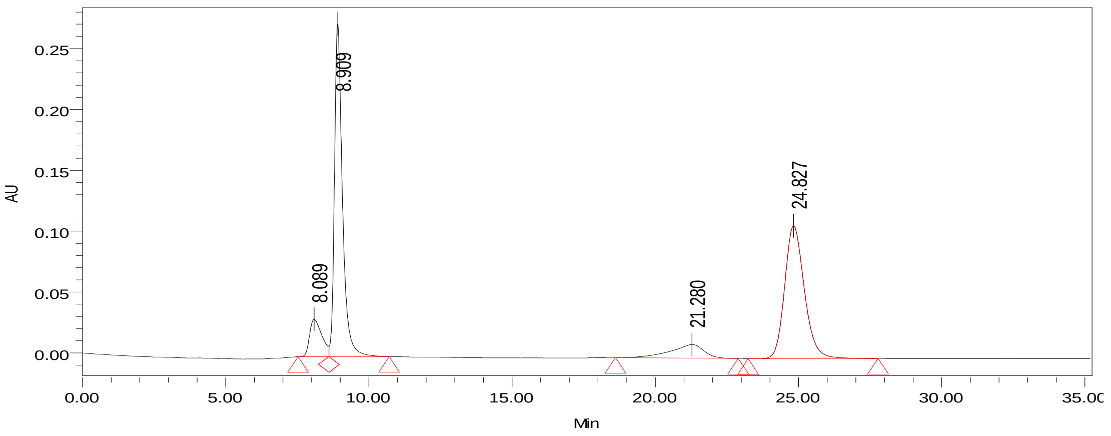

Entry	Retention time	Area	Area (%)	Height	Int type
1	8.089	919226	7.44	30675	bv
2	8.909	5311853	43.00	273055	vb
3	21.280	914765	7.41	11206	bb
4	24.827	5206541	42.15	109184	bb

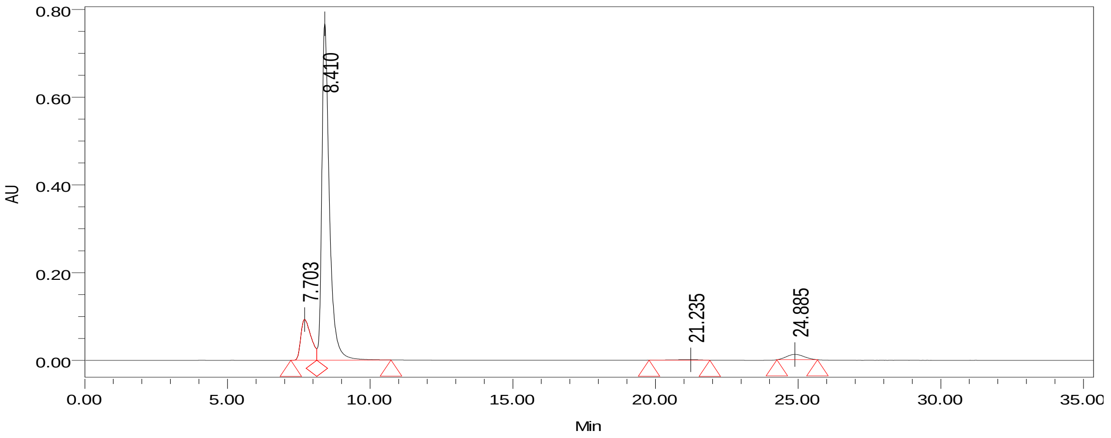

Entry	Retention time	Area	Area (%)	Height	Int type
1	7.703	2339210	13.71	93136	bv
2	8.410	14134817	82.86	767099	vb
3	21.235	66329	0.39	1043	bb
4	24.885	517761	3.04	12151	bb

Supplementary Fig. 49. HPLC chromatograms of compound **4a.**

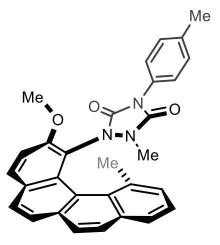

4b

HPLC condition: Chiralcel IA-H, *n*-hexane/*i*-PrOH(1‰ TFA) = 7/3, flow rate = 1.0 mL/min.

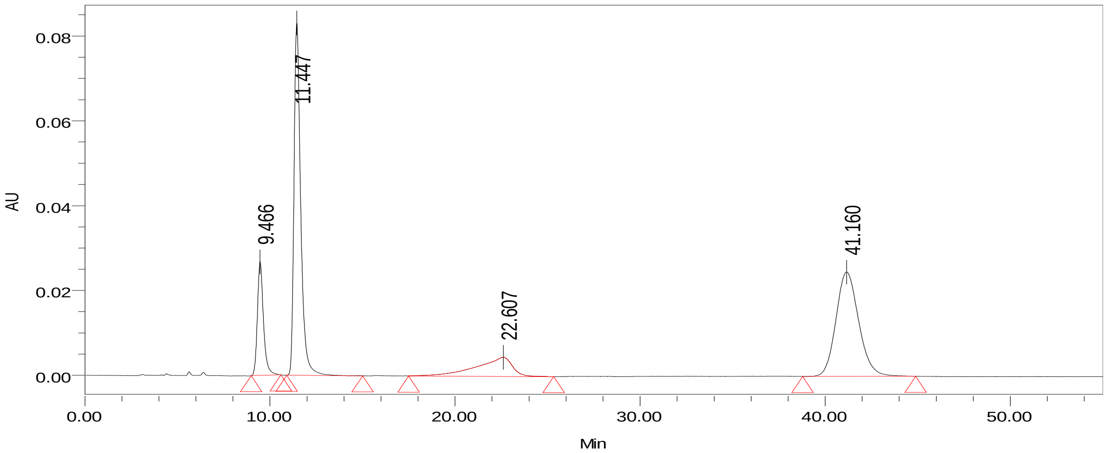

Entry	Retention time	Area	Area (%)	Height	Int type
1	9.466	610970	11.31	26800	bb
2	11.447	2095296	38.80	83147	bb
3	22.607	596637	11.05	4490	bb
4	41.160	2098029	38.85	24594	bb

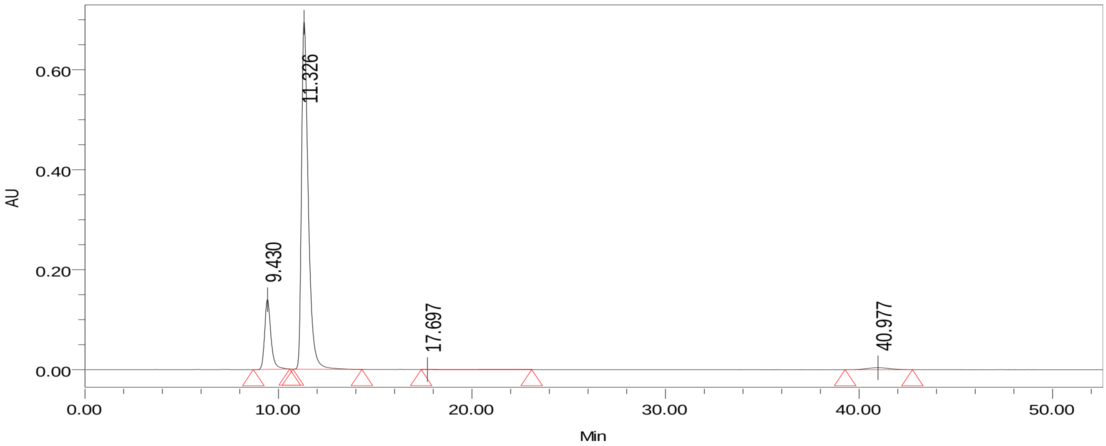

Entry	Retention time	Area	Area (%)	Height	Int type
1	9.430	3144108	15.38	139905	bb
2	11.326	16912219	82.73	694055	bb
3	17.697	62038	0.30	417	bb
4	40.977	325107	1.59	3992	bb

Supplementary Fig. 50. HPLC chromatograms of compound **4b.**

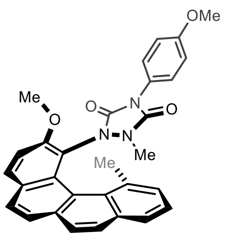

4c

HPLC condition: Chiralcel IA-H, *n*-hexane/*i*-PrOH(1‰ TFA) = 6/4, flow rate = 1.0 mL/min.

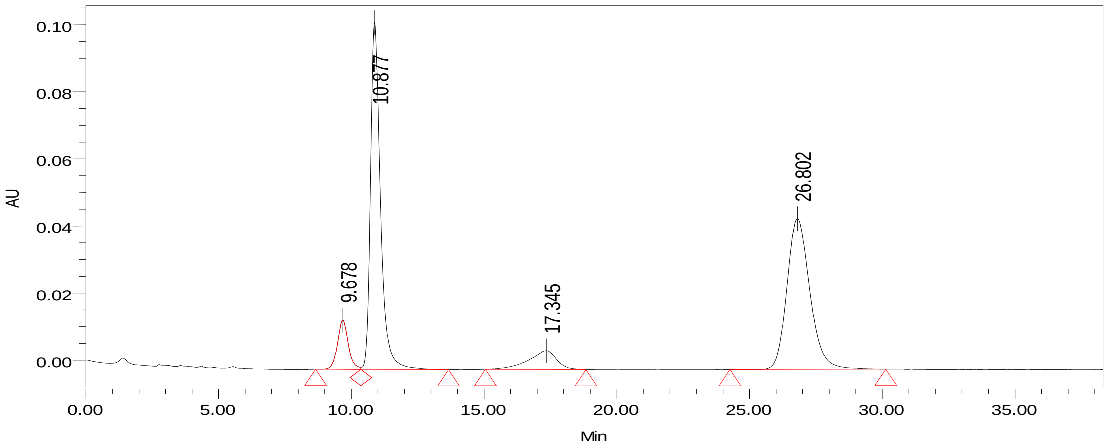

Entry	Retention time	Area	Area (%)	Height	Int type
1	9.678	413855	6.64	14655	bv
2	10.877	2708956	43.49	103361	vb
3	17.345	405956	6.52	5529	bb
4	26.802	2699572	43.34	44922	bb

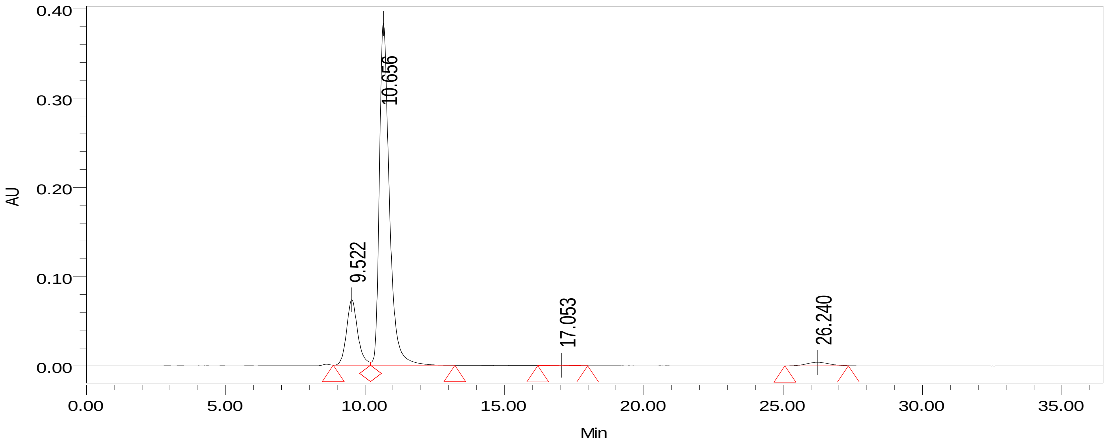

Entry	Retention time	Area	Area (%)	Height	Int type
1	9.522	2035937	17.16	73393	bv
2	10.656	9586417	80.80	383096	vb
3	17.053	24481	0.21	514	bb
4	26.240	216925	1.83	3903	bb

Supplementary Fig. 51. HPLC chromatograms of compound **4c.**

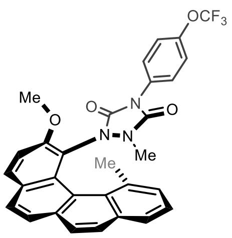

4d

HPLC condition: Chiralcel IA-H, *n*-hexane/EtOH(1‰ TFA) = 7/3, flow rate = 1.0 mL/min.

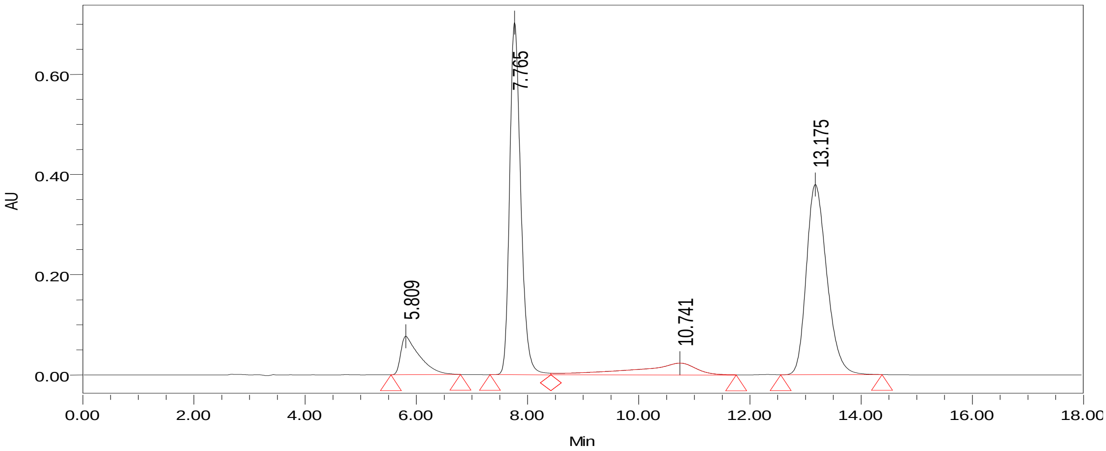

Entry	Retention time	Area	Area (%)	Height	Int type
1	5.809	1767221	7.69	76815	bb
2	7.765	9773347	42.51	702870	bv
3	10.741	1714643	7.46	23313	vb
4	13.175	9735005	42.34	379908	bb

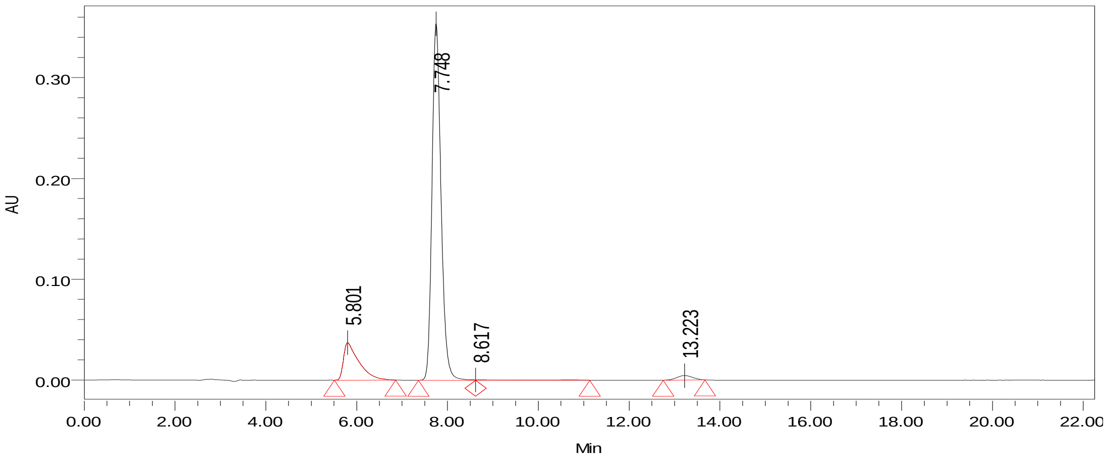

Entry	Retention time	Area	Area (%)	Height	Int type
1	5.801	869294	14.75	37208	bb
2	7.748	4895120	83.06	353713	bv
3	8.617	21969	0.37	317	vb
4	13.223	107284	1.82	4459	bb

Supplementary Fig. 52. HPLC chromatograms of compound **4d.**

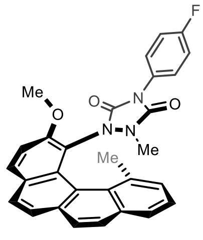

4e

HPLC condition: Chiralcel IA-H, *n*-hexane/EtOH(1‰ TFA) = 7/3, flow rate = 1.0 mL/min.

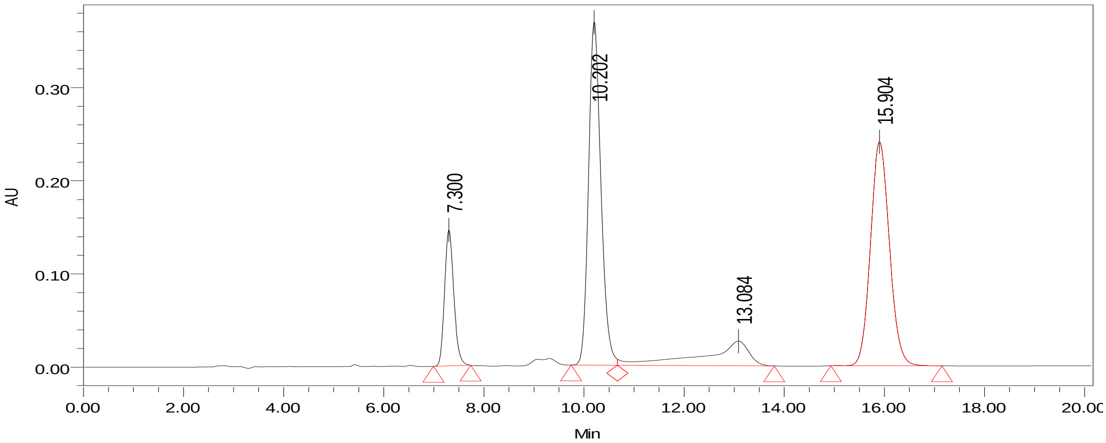

Entry	Retention time	Area	Area (%)	Height	Int type
1	7.300	1868036	11.51	146019	bb
2	10.202	6343269	39.10	368342	bv
3	13.084	1687026	10.40	26525	vb
4	15.904	6324321	38.98	240507	bb

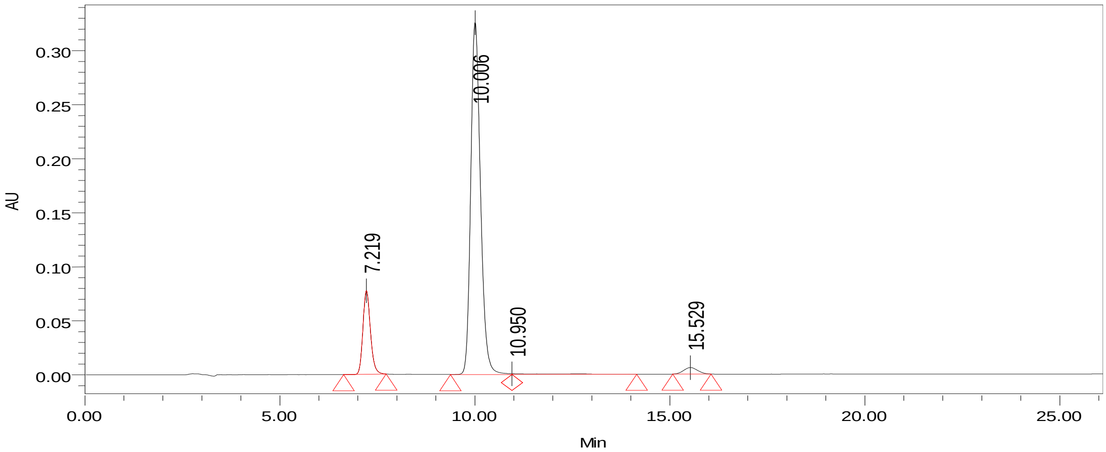

Entry	Retention time	Area	Area (%)	Height	Int type
1	7.219	1033446	15.03	77421	bb
2	10.006	5651819	82.19	325695	bv
3	10.950	41383	0.60	585	vb
4	15.529	150274	2.19	6042	bb

Supplementary Fig. 53. HPLC chromatograms of compound **4e.**

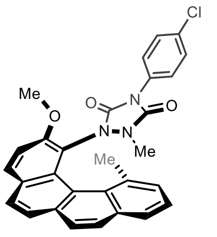

4f

HPLC condition: Chiralcel IA-H, *n*-hexane/EtOH(1‰ TFA) = 7/3, flow rate = 1.0 mL/min.

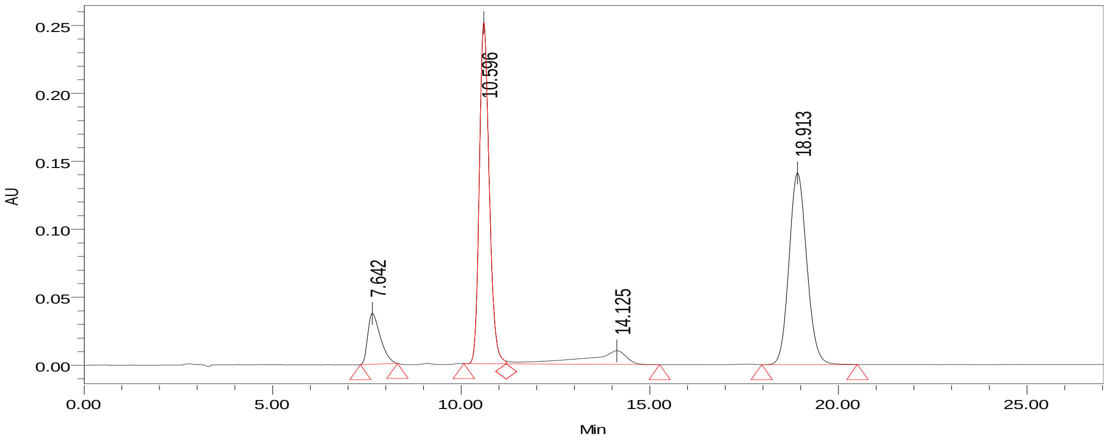

Entry	Retention time	Area	Area (%)	Height	Int type
1	7.642	870084	8.02	37539	bb
2	10.596	4584686	42.28	251159	bv
3	14.125	789943	7.29	10053	vb
4	18.913	4597910	42.41	141018	bb

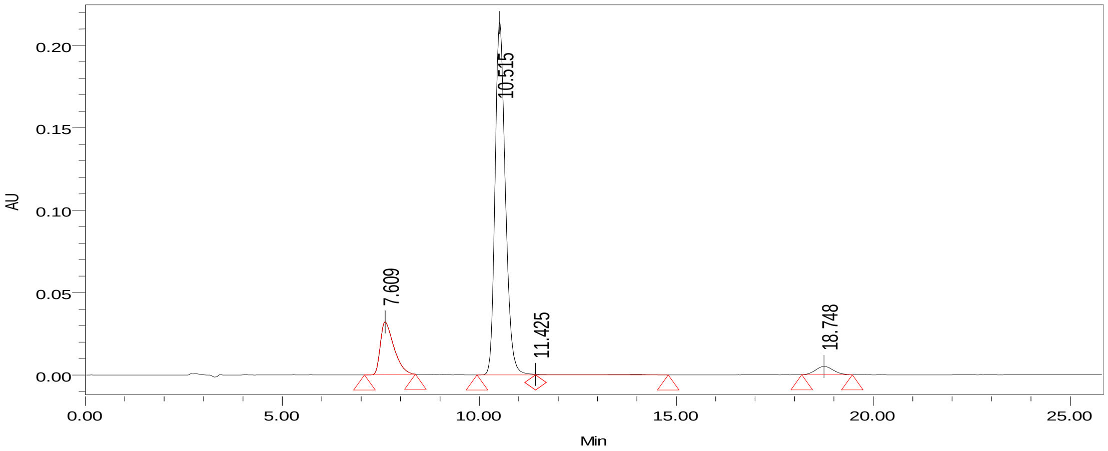

Entry	Retention time	Area	Area (%)	Height	Int type
1	7.609	763144	15.70	31996	bb
2	10.515	3901795	80.26	213785	bv
3	11.425	40465	0.83	388	vb
4	18.748	155964	3.21	5024	bb

Supplementary Fig. 54. HPLC chromatograms of compound **4f.**

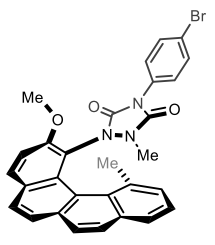

4g

HPLC condition: Chiralcel IA-H, *n*-hexane/*i*-PrOH(1‰ TFA) = 7/3, flow rate = 1.0 mL/min.

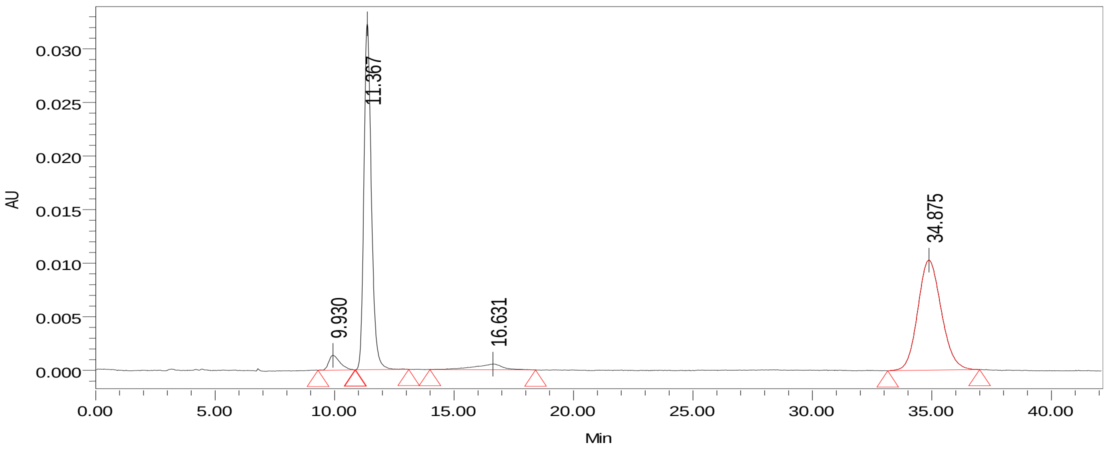

Entry	Retention time	Area	Area (%)	Height	Int type
1	9.930	44359	2.97	1376	bb
2	11.367	701163	46.96	32262	bb
3	16.631	44546	2.98	527	bb
4	34.875	703025	47.09	10278	bb

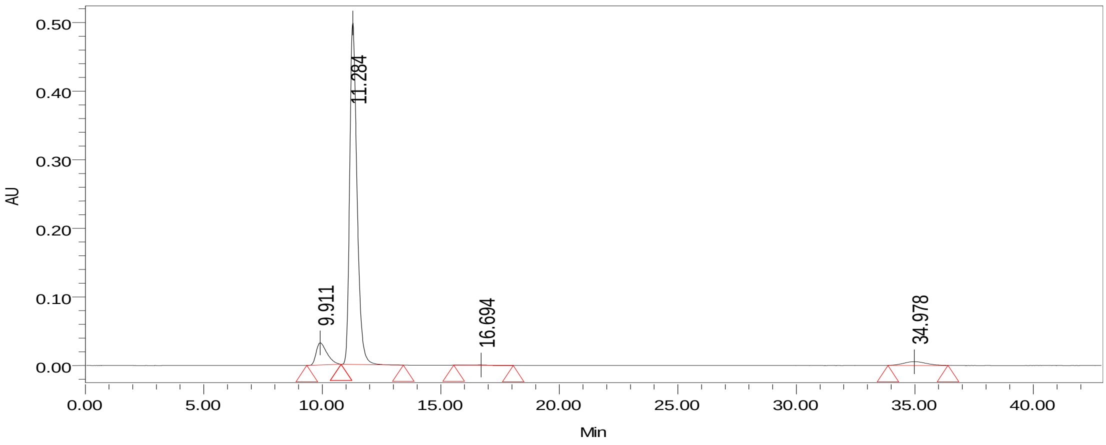

Entry	Retention time	Area	Area (%)	Height	Int type
1	9.911	1019577	8.42	32319	bb
2	11.284	10707329	88.47	497872	bb
3	16.694	18593	0.15	409	bb
4	34.978	356749	2.95	5466	bb

Supplementary Fig. 55. HPLC chromatograms of compound **4g.**

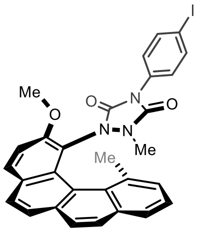

4h

HPLC condition: Chiralcel IA-H, *n*-hexane/*i*-PrOH(1‰ TFA) = 7/3, flow rate = 1.0 mL/min.

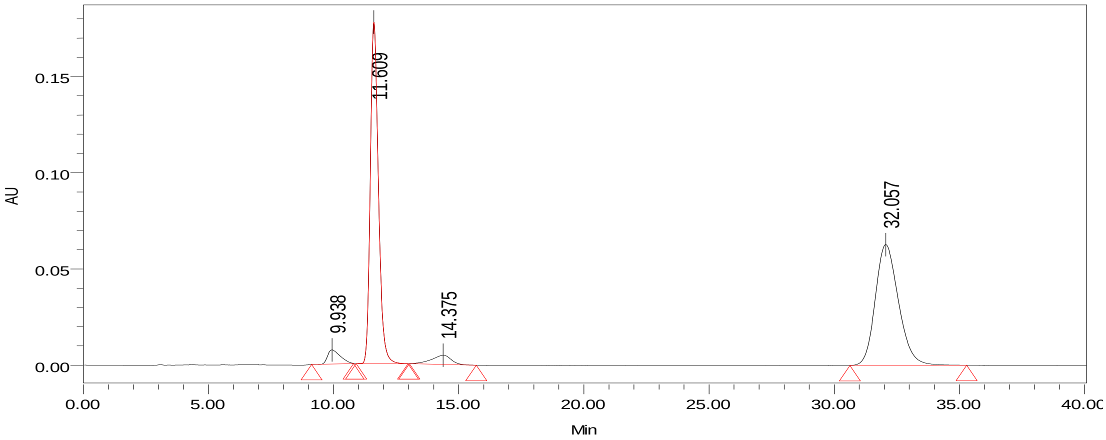

Entry	Retention time	Area	Area (%)	Height	Int type
1	9.938	256132	3.00	7302	bb
2	11.609	3979467	46.61	177560	bb
3	14.375	257966	3.02	4798	bb
4	32.057	4043979	47.37	62774	bb

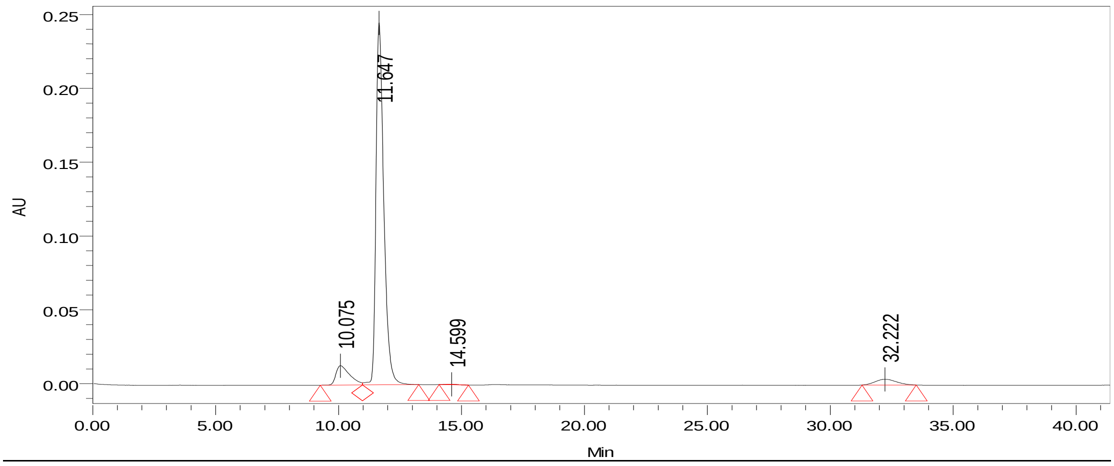

Entry	Retention time	Area	Area (%)	Height	Int type
1	10.075	510186	8.23	13127	bv
2	11.647	5443555	87.84	245111	vb
3	14.599	12859	0.21	384	bb
4	32.222	230560	3.72	3883	bb

Supplementary Fig. 56. HPLC chromatograms of compound **4h.**

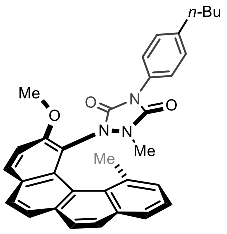

4i

HPLC condition: Chiralcel IA-H, *n*-hexane/EtOH(1‰ TFA) = 7/3, flow rate = 1.0 mL/min.

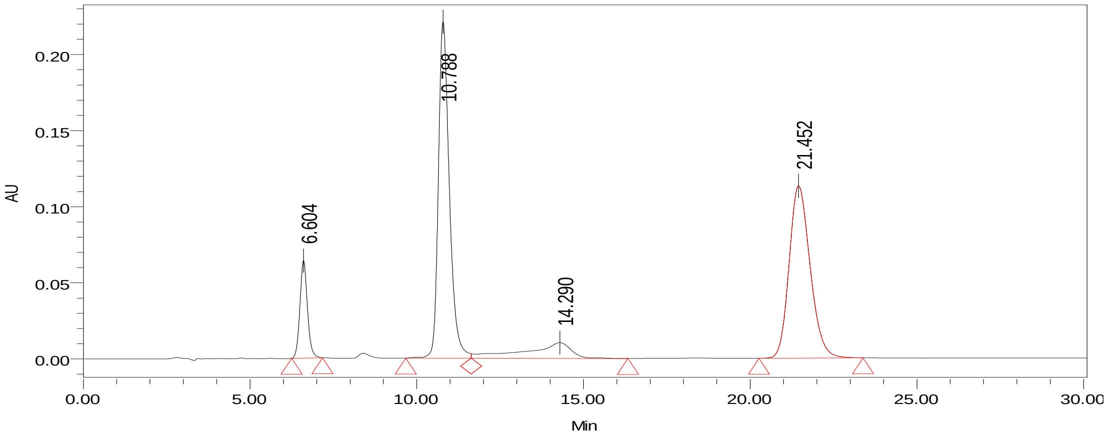

Entry	Retention time	Area	Area (%)	Height	Int type
1	6.604	1000212	8.32	64124	bb
2	10.788	5075740	42.25	221055	bv
3	14.290	983436	8.19	10312	vb
4	21.452	4955233	41.24	113136	bb

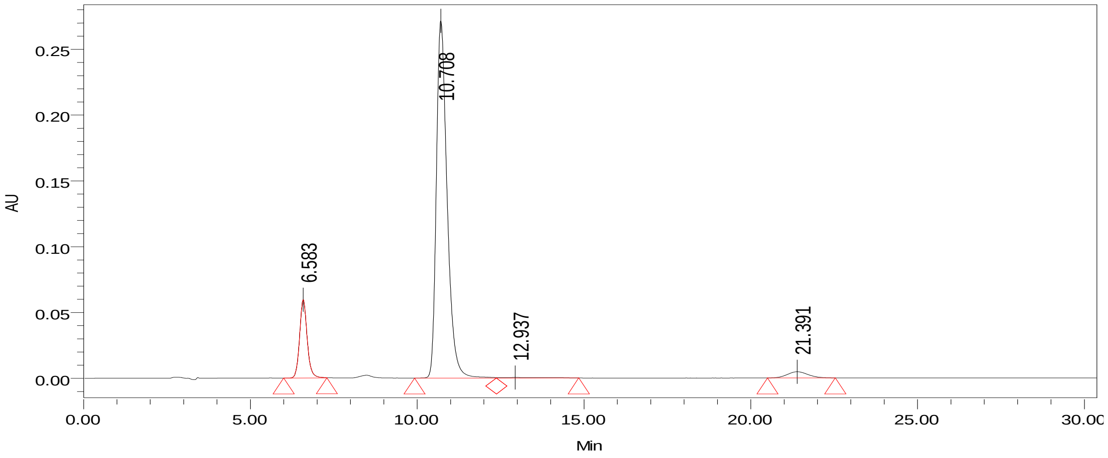

Entry	Retention time	Area	Area (%)	Height	Int type
1	6.583	933388	12.83	59489	bb
2	10.708	6100731	83.88	271343	bv
3	12.937	38581	0.53	391	vb
4	21.391	200596	2.76	4667	bb

Supplementary Fig. 57. HPLC chromatograms of compound **4i.**

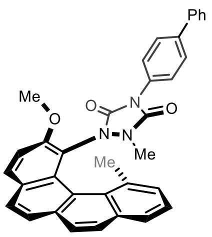

4j

HPLC condition: Chiralcel IA-H, *n*-hexane/*i*-PrOH(1‰ TFA) = 7/3, flow rate = 1.0 mL/min.

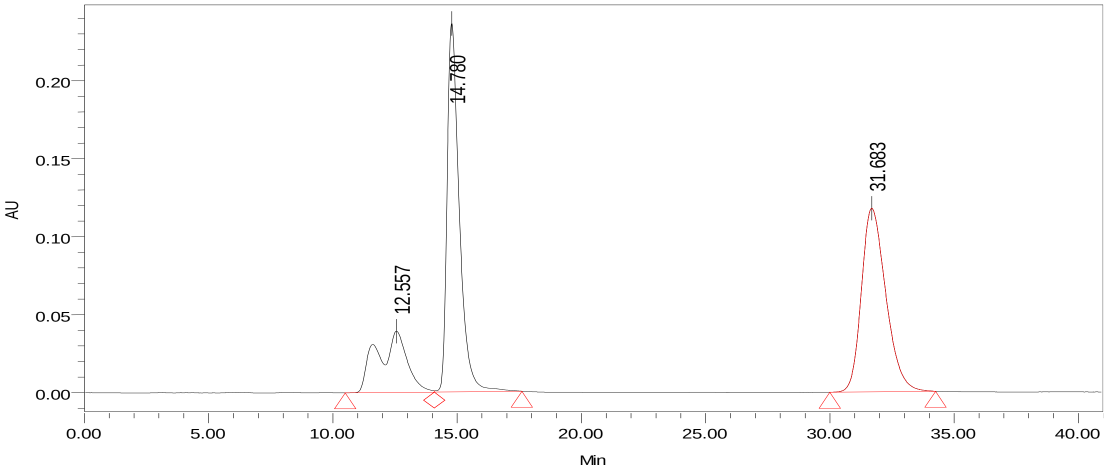

Entry	Retention time	Area	Area (%)	Height	Int type
1	12.557	3279389	16.87	39240	bv
2	14.780	8104642	41.70	236305	vb
3	31.683	8050815	41.42	117730	bb

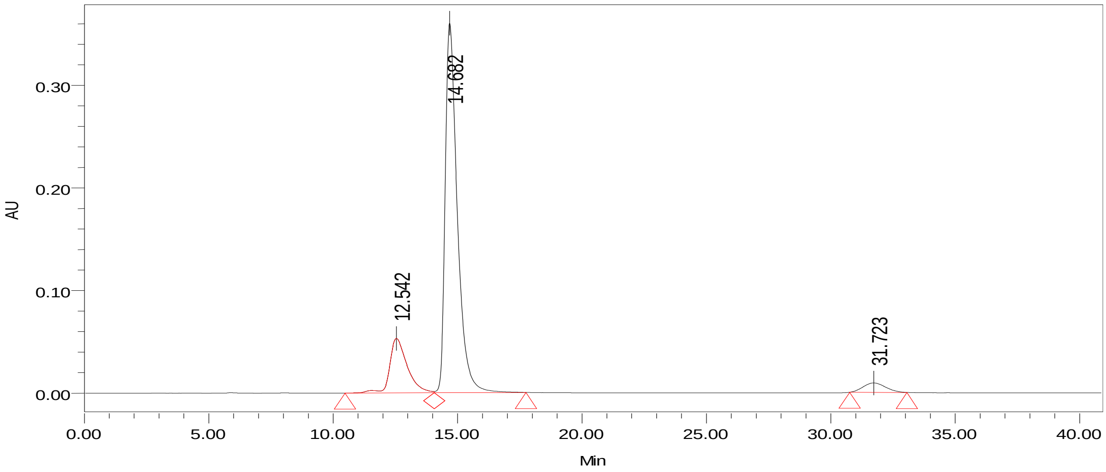

Entry	Retention time	Area	Area (%)	Height	Int type
1	12.542	2586714	16.87	53032	bv
2	14.682	12176481	79.41	360176	vb
3	31.723	570864	3.72	9149	bb

Supplementary Fig. 58. HPLC chromatograms of compound **4j.**

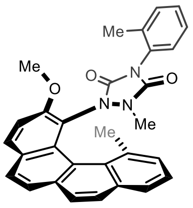

4k

HPLC condition: Chiralcel AD-H, *n*-hexane/EtOH(1‰ TFA) = 7/3, flow rate = 1.0 mL/min.

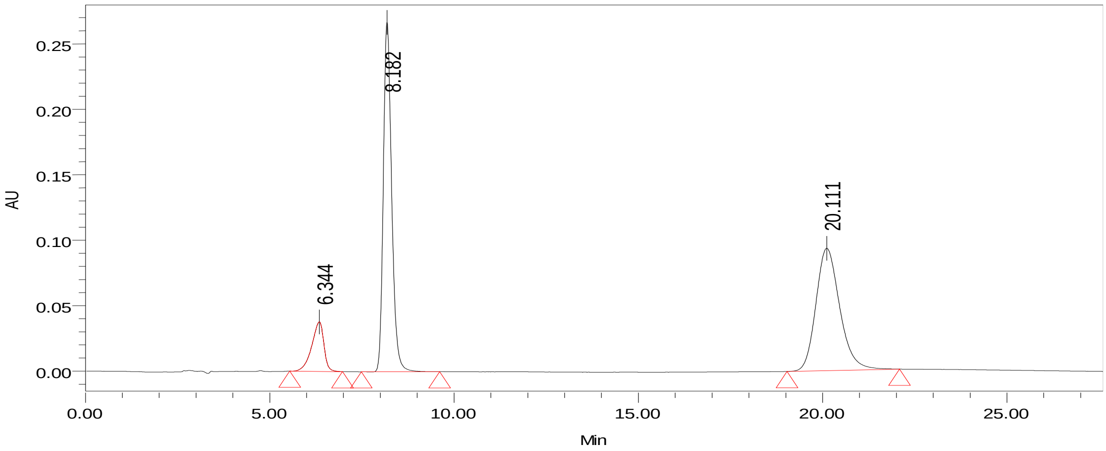

Entry	Retention time	Area	Area (%)	Height	Int type
1	6.344	866485	9.36	37872	bb
2	8.182	4195650	45.31	266880	bb
3	20.111	4198318	45.34	93516	bb

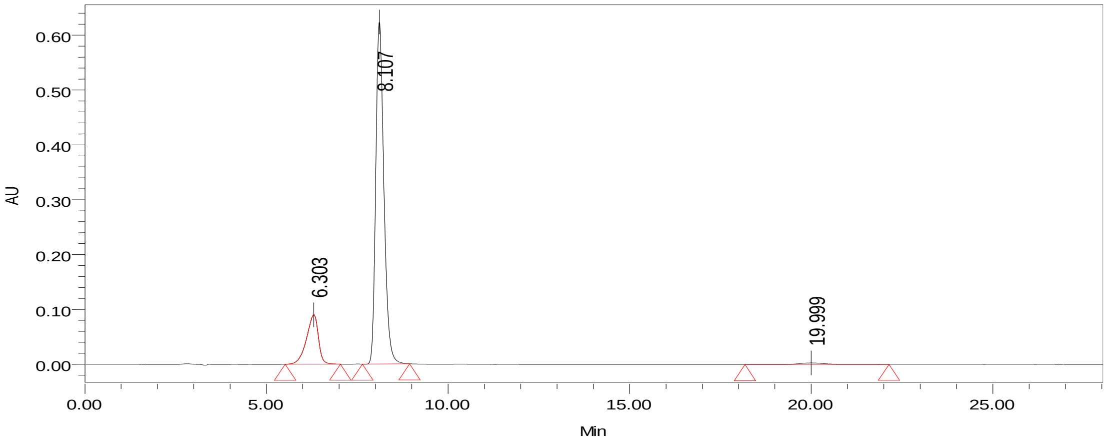

Entry	Retention time	Area	Area (%)	Height	Int type
1	6.303	2057191	17.28	90259	bb
2	8.107	9725628	81.70	624014	bb
3	19.999	120546	1.01	2689	bb

Supplementary Fig. 59. HPLC chromatograms of compound **4k.**

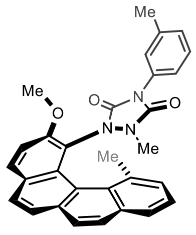

4l

HPLC condition: Chiralcel AD-H, *n*-hexane/*i*-PrOH (1‰ TFA) = 7/3, flow rate = 1.0 mL/min.

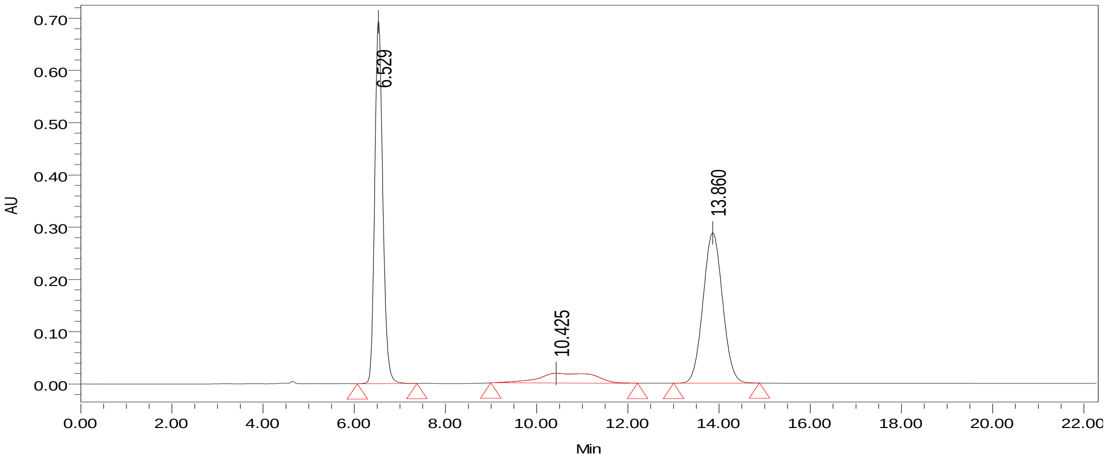

Entry	Retention time	Area	Area (%)	Height	Int type
1	6.529	8533422	45.68	693318	bb
2	10.425	1610730	8.62	18436	bb
3	13.860	8535802	45.69	287608	bb

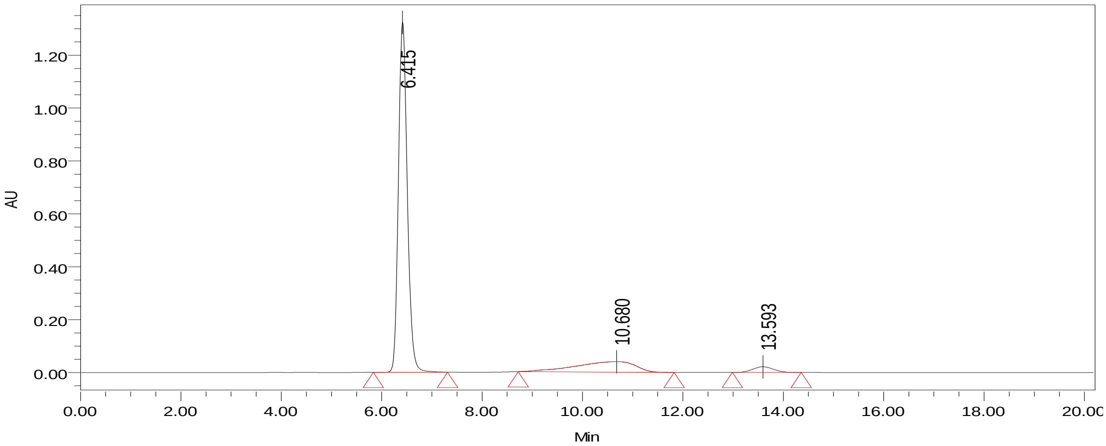

Entry	Retention time	Area	Area (%)	Height	Int type
1	6.415	16240183	80.72	1323150	bb
2	10.680	3253391	16.17	39588	bb
3	13.593	624624	3.10	21511	bb

Supplementary Fig. 60. HPLC chromatograms of compound **4l.**

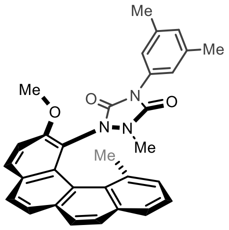

4m

HPLC condition: Chiralcel IA-H, *n*-hexane/EtOH (1‰ TFA) = 7/3, flow rate = 1.0 mL/min.

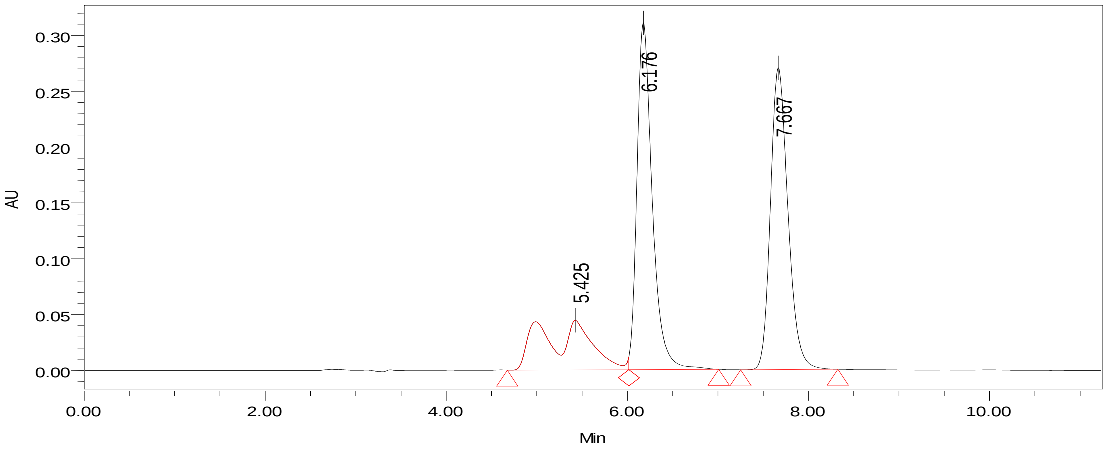

Entry	Retention time	Area	Area (%)	Height	Int type
1	5.425	1644337	18.40	44360	bv
2	6.176	3651039	40.86	310708	vb
3	7.667	3639822	40.74	270308	bb

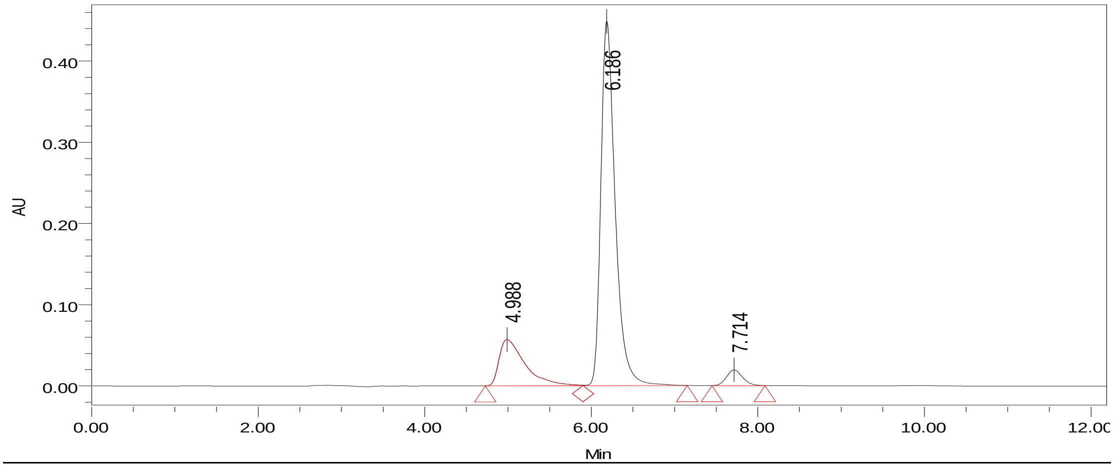

Entry	Retention time	Area	Area (%)	Height	Int type
1	4.988	1229314	17.92	56974	bv
2	6.186	5370445	78.31	449019	vb
3	7.714	258349	3.77	19330	bb

Supplementary Fig. 61. HPLC chromatograms of compound **4m.**

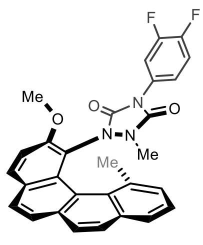

4n

HPLC condition: Chiralcel IA-H, *n*-hexane/EtOH (1‰ TFA) = 7/3, flow rate = 1.0 mL/min.

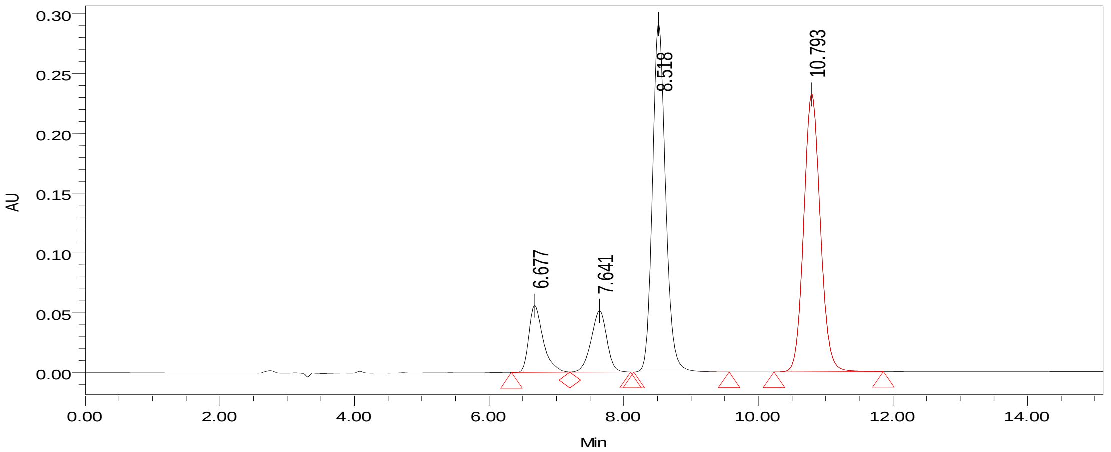

Entry	Retention time	Area	Area (%)	Height	Int type
1	6.677	827257	8.64	55909	bv
2	7.641	828523	8.66	51279	vb
3	8.518	3946330	41.23	291148	bb
4	10.793	3969337	41.47	232036	bb

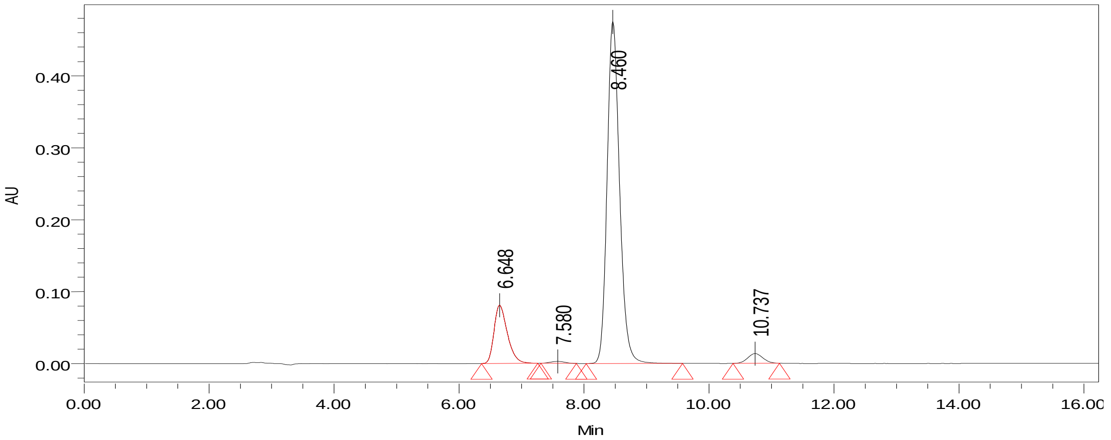

Entry	Retention time	Area	Area (%)	Height	Int type
1	6.648	1202681	14.91	81019	bb
2	7.580	44554	0.55	2760	bb
3	8.460	6591313	81.70	475043	bb
4	10.737	229186	2.84	13571	bb

Supplementary Fig. 62. HPLC chromatograms of compound **4n.**

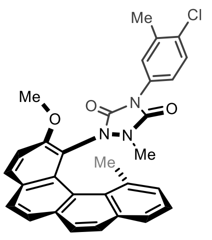

4o

HPLC condition: Chiralcel IA-H, *n*-hexane/EtOH (1‰ TFA) = 7/3, flow rate = 1.0 mL/min.

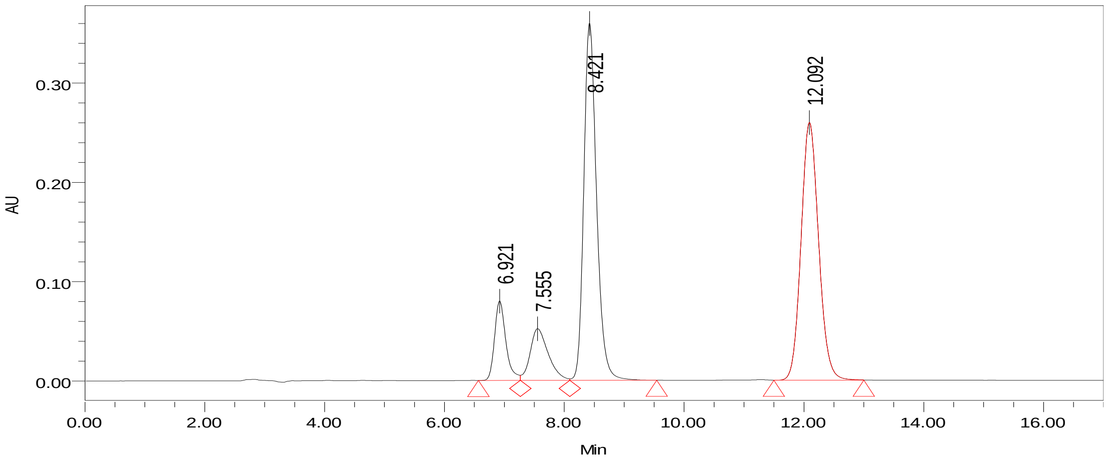

Entry	Retention time	Area	Area (%)	Height	Int type
1	6.921	1054319	8.27	80056	bv
2	7.555	1092379	8.57	52176	vv
3	8.421	5315833	41.69	359751	vb
4	12.092	5289068	41.48	259529	bb

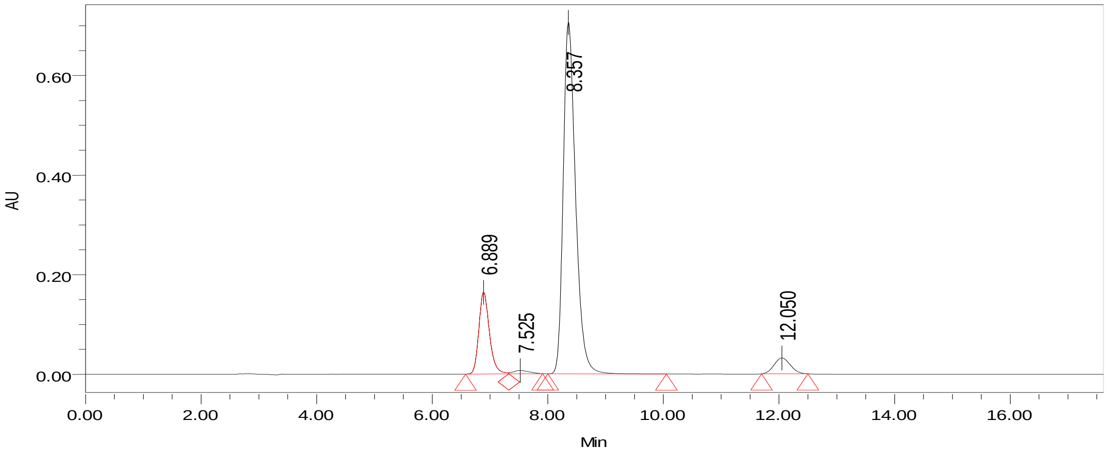

Entry	Retention time	Area	Area (%)	Height	Int type
1	6.889	2090280	15.69	164429	bv
2	7.525	128678	0.97	6616	vb
3	8.357	10476980	78.67	706128	bb
4	12.050	622470	4.67	31903	bb

Supplementary Fig. 63. HPLC chromatograms of compound **4o.**

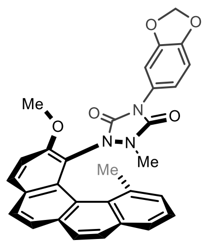

4p

HPLC condition: Chiralcel IA-H, *n*-hexane/EtOH (1‰ TFA) = 7/3, flow rate = 1.0 mL/min.

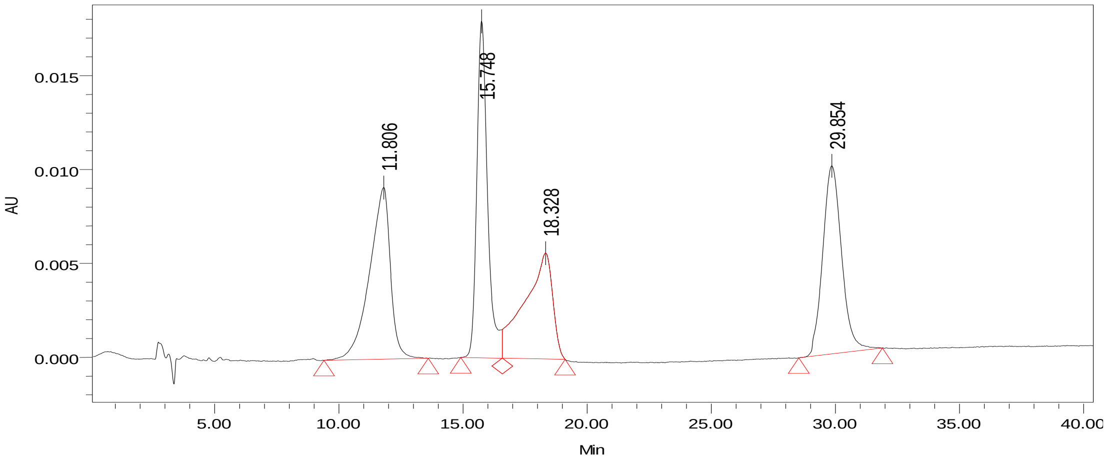

Entry	Retention time	Area	Area (%)	Height	Int type
1	11.806	534638	26.16	9143	bb
2	15.748	536699	26.26	17922	bv
3	18.328	443355	21.69	5639	vb
4	29.854	529114	25.89	10018	bb

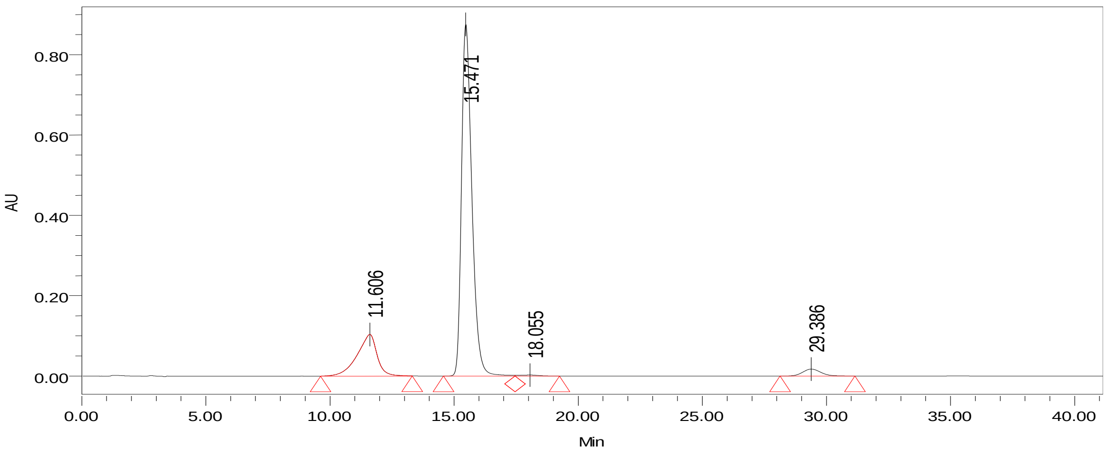

Entry	Retention time	Area	Area (%)	Height	Int type
1	11.606	5740320	17.86	103610	bb
2	15.471	25386942	78.99	875635	bv
3	18.055	142619	0.44	2659	vb
4	29.386	871105	2.71	17301	bb

Supplementary Fig. 64. HPLC chromatograms of compound **4p.**

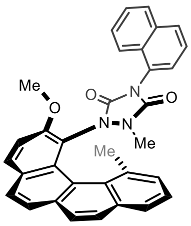

4q

HPLC condition: Chiralcel IA-H, *n*-hexane/*i*-PrOH (1‰ TFA) = 7/3, flow rate = 1.0 mL/min.

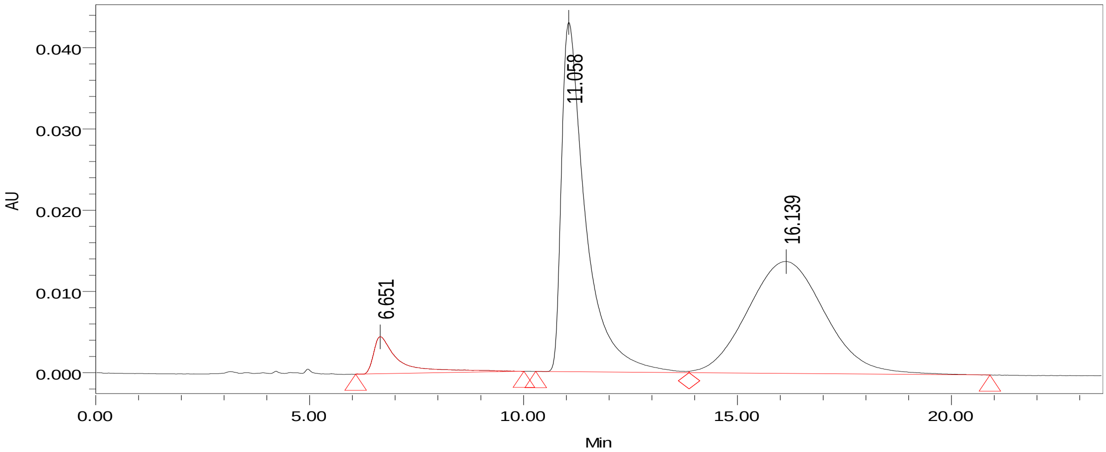

Entry	Retention time	Area	Area (%)	Height	Int type
1	6.651	193931	5.37	4563	bb
2	11.058	1691961	46.82	43006	bv
3	16.139	1727921	47.81	13773	vb

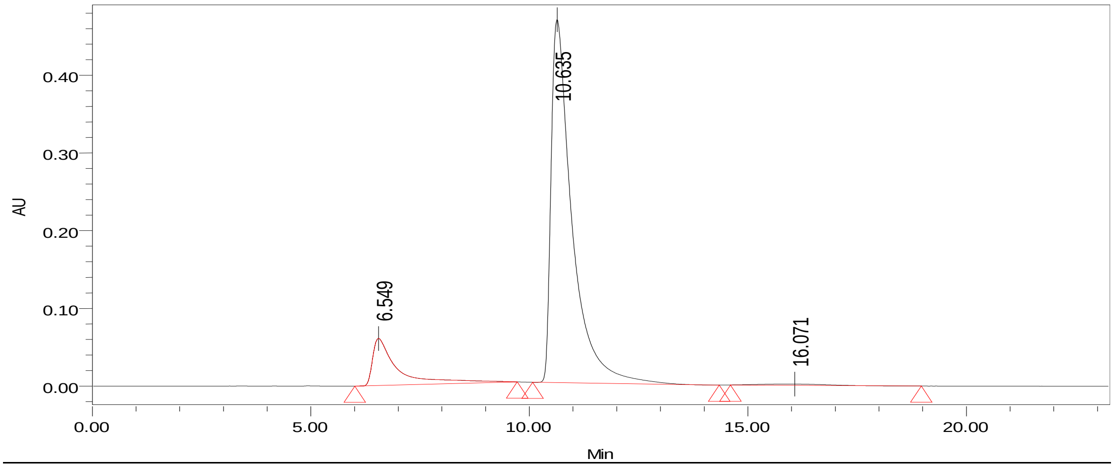

Entry	Retention time	Area	Area (%)	Height	Int type
1	6.549	2412673	13.28	60522	bb
2	10.635	15582774	85.78	467134	bb
3	16.071	170457	0.94	1685	bb

Supplementary Fig. 65. HPLC chromatograms of compound **4q.**

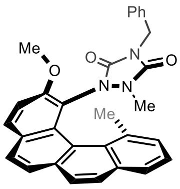

4r

HPLC condition: Chiralcel IA-H, *n*-hexane/EtOH (1‰ TFA) = 7/3, flow rate = 1.0 mL/min.

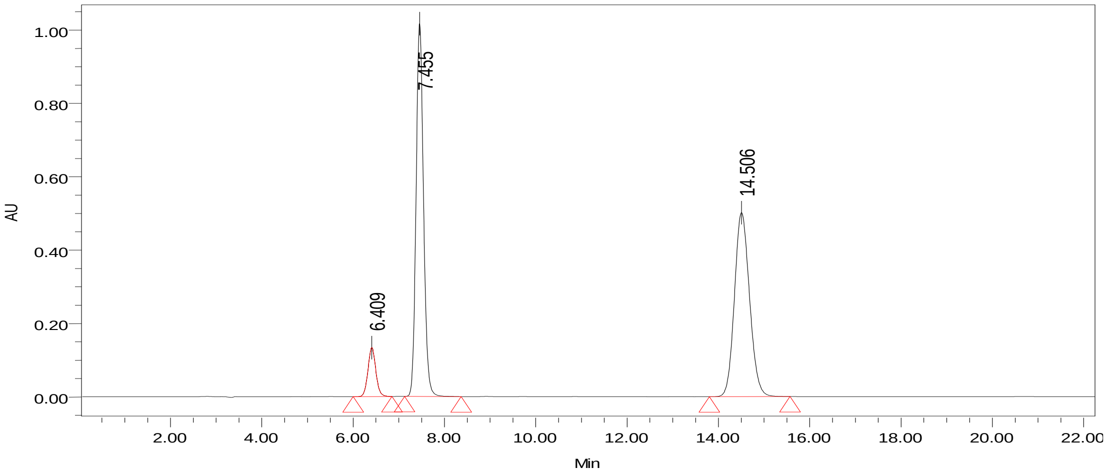

Entry	Retention time	Area	Area (%)	Height	Int type
1	6.409	1613632	6.54	133911	bb
2	7.455	11513925	46.69	1017565	bb
3	14.506	11531973	46.76	501952	bb

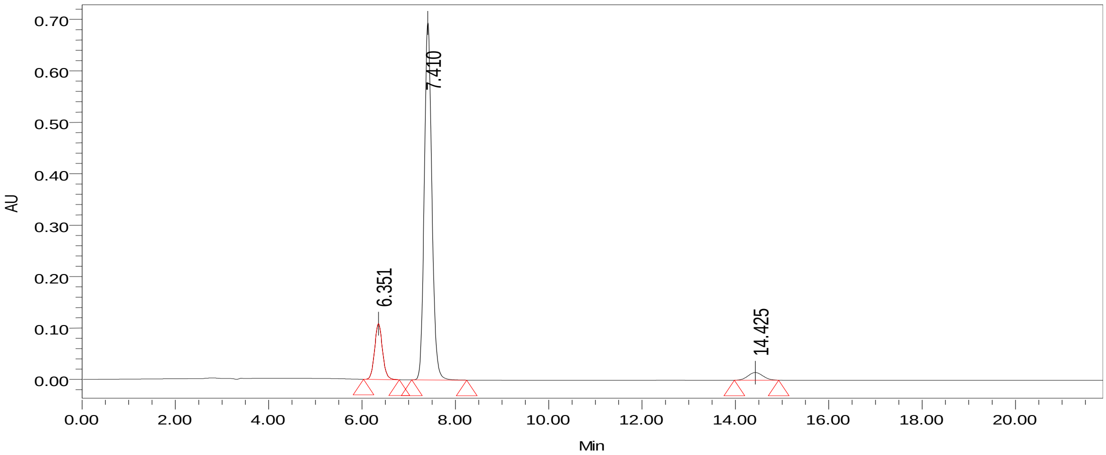

Entry	Retention time	Area	Area (%)	Height	Int type
1	6.351	1279163	13.52	108777	bb
2	7.410	7847342	82.96	694924	bb
3	14.425	332729	3.52	15009	bb

Supplementary Fig. 66. HPLC chromatograms of compound **4r.**

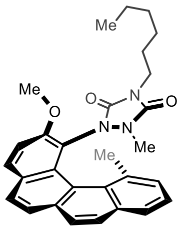

4s

HPLC condition: Chiralcel AS-H, *n*-hexane/EtOH (1‰ TFA) = 7/3, flow rate = 1.0 mL/min.

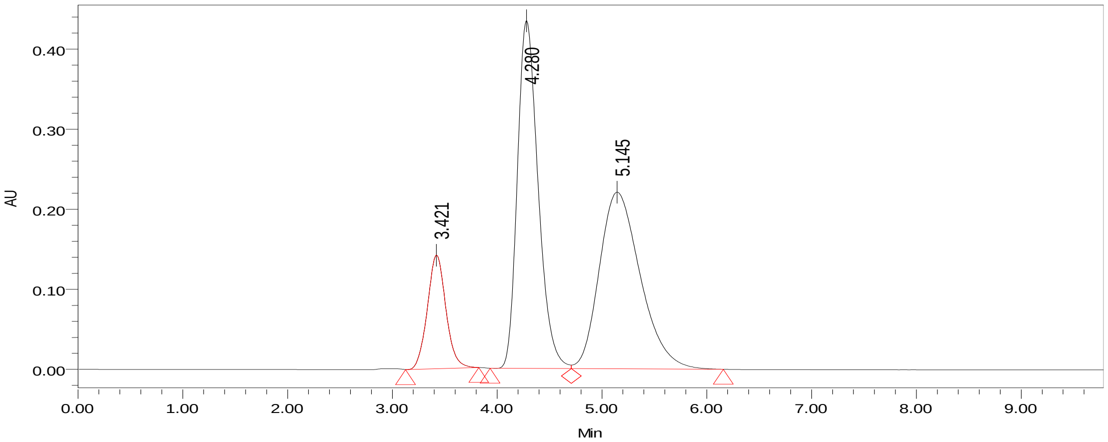

Entry	Retention time	Area	Area (%)	Height	Int type
1	3.421	1742089	12.68	141866	bb
2	4.280	5993627	43.63	434499	bv
3	5.145	6001345	43.69	220666	vb

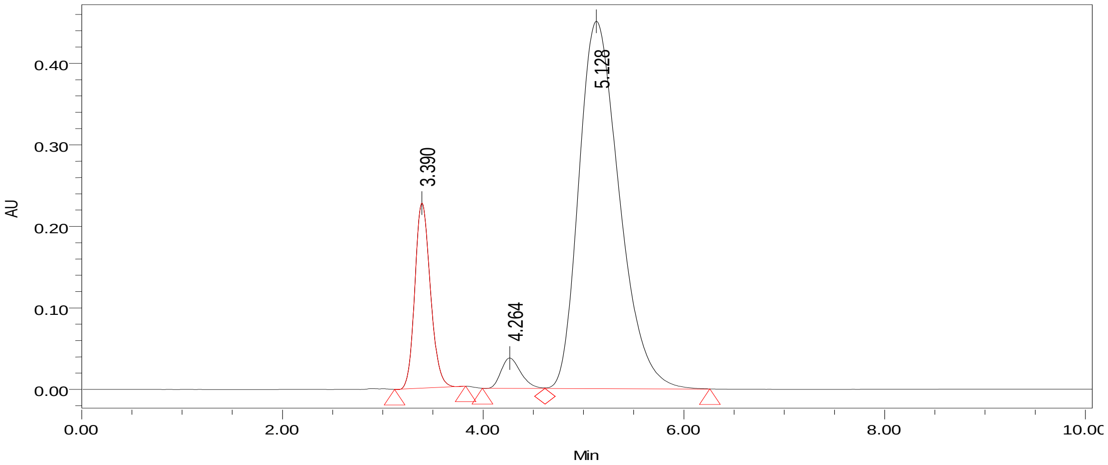

Entry	Retention time	Area	Area (%)	Height	Int type
1	3.390	2448390	15.71	227210	bb
2	4.264	505081	3.24	37270	bv
3	5.128	12635514	81.05	450687	vb

Supplementary Fig. 67. HPLC chromatograms of compound **4s.**

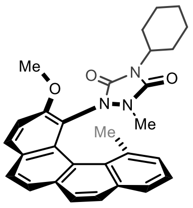

4t

HPLC condition: Chiralcel IA-H, *n*-hexane/EtOH (1‰ TFA) = 19/1, flow rate = 1.0 mL/min.

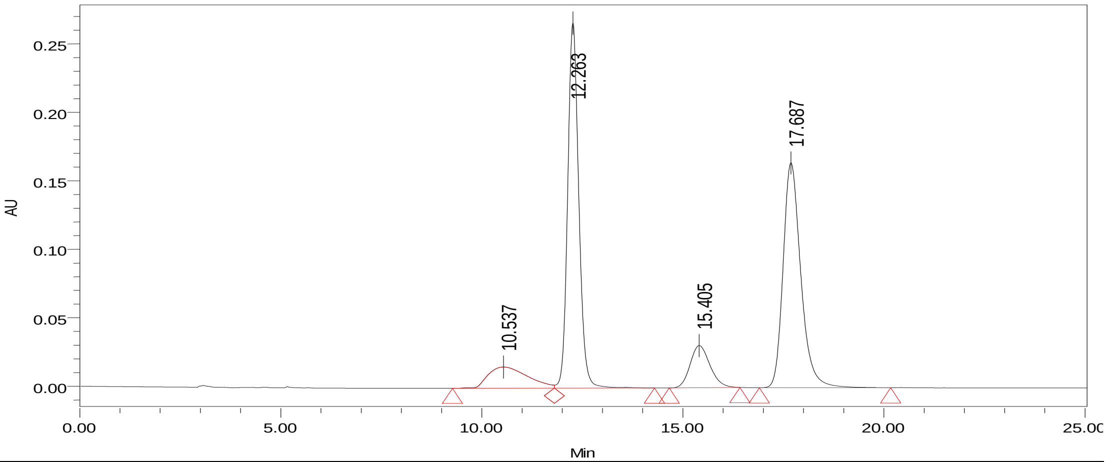

Entry	Retention time	Area	Area (%)	Height	Int type
1	10.537	1054154	8.59	15494	bv
2	12.263	5125477	41.78	266525	vb
3	15.405	1058302	8.63	30817	bb
4	17.687	5029525	41.00	164215	bb

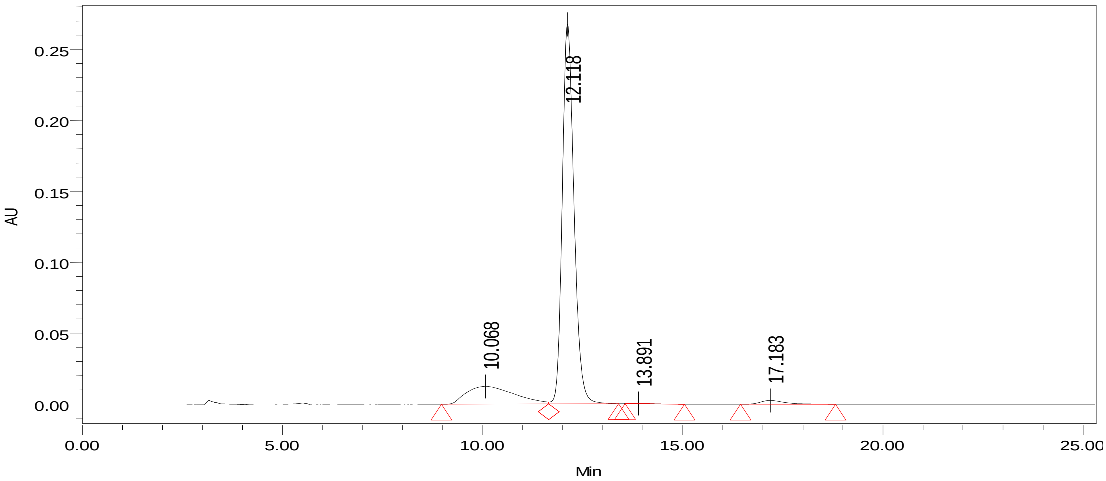

Entry	Retention time	Area	Area (%)	Height	Int type
1	10.068	1030670	15.61	12441	bv
2	12.118	5454104	82.61	267502	vb
3	13.891	8487	0.13	259	bb
4	17.183	109205	1.65	2676	bb

Supplementary Fig. 68. HPLC chromatograms of compound **4t.**

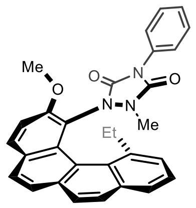

4u

HPLC condition: Chiralcel AD-H, *n*-hexane/*i*-PrOH (1‰ TFA) = 7/3, flow rate = 1.0 mL/min.

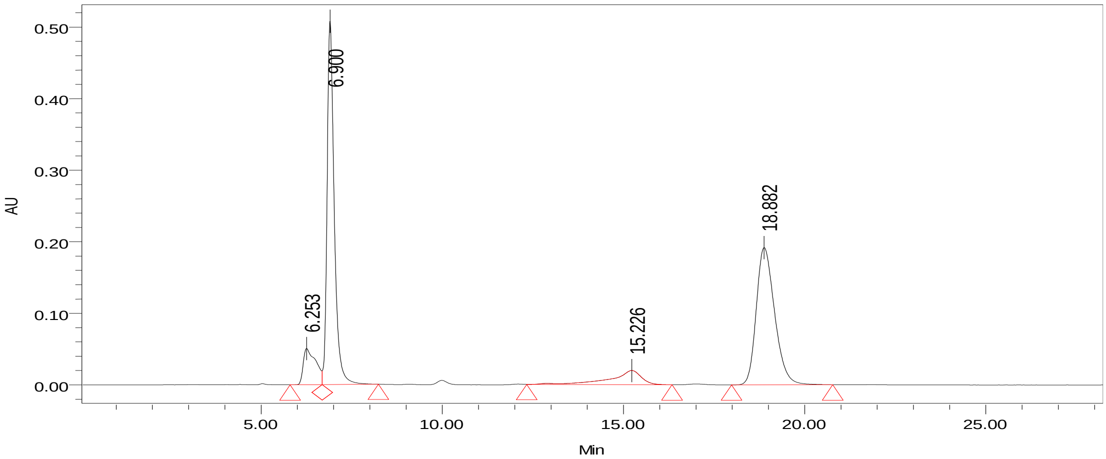

Entry	Retention time	Area	Area (%)	Height	Int type
1	6.253	1219204	7.57	50647	bv
2	6.900	6955810	43.19	507722	vb
3	15.226	1172302	7.28	19842	bb
4	18.882	6756660	41.96	191911	bb

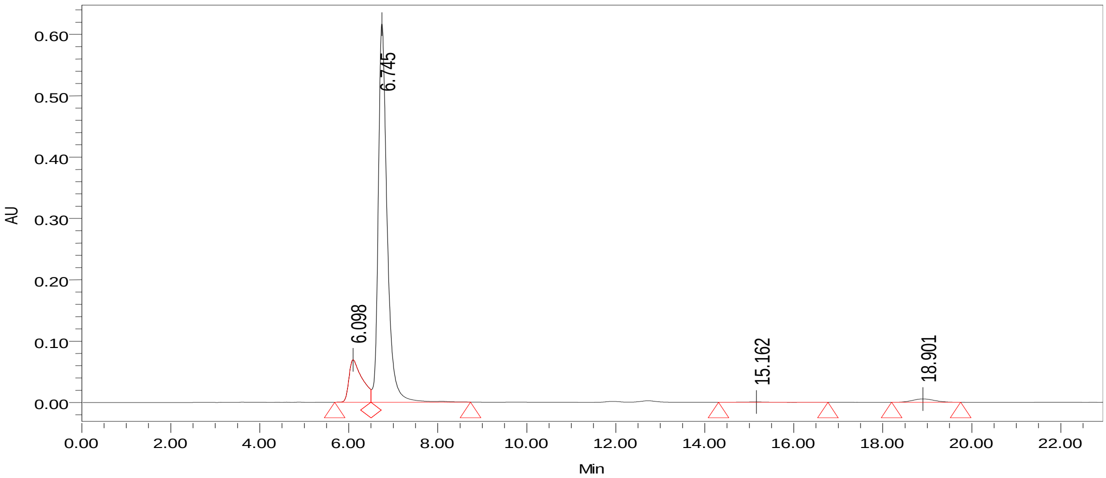

Entry	Retention time	Area	Area (%)	Height	Int type
1	6.098	1446565	14.11	69203	bv
2	6.745	8588560	83.78	616952	vb
3	15.162	22721	0.22	426	bb
4	18.901	193110	1.88	5563	bb

Supplementary Fig. 69. HPLC chromatograms of compound **4u.**

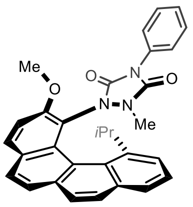

4v

HPLC condition: Chiralcel AD-H, *n*-hexane/*i*-PrOH (1‰ TFA) = 7/3, flow rate = 1.0 mL/min.

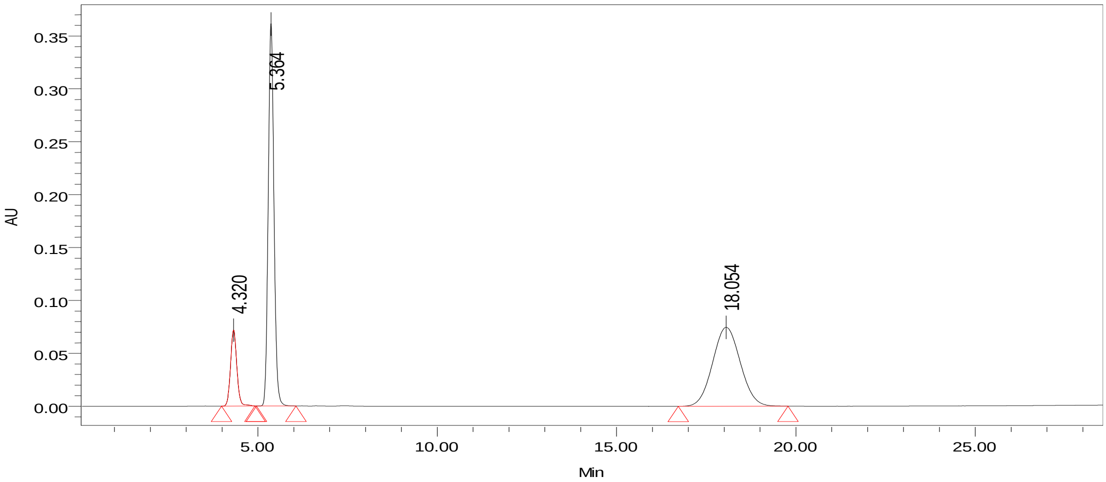

Entry	Retention time	Area	Area (%)	Height	Int type
1	4.320	876572	9.87	71944	bb
2	5.364	4001491	45.04	361438	bb
3	18.054	4005606	45.09	74608	bb

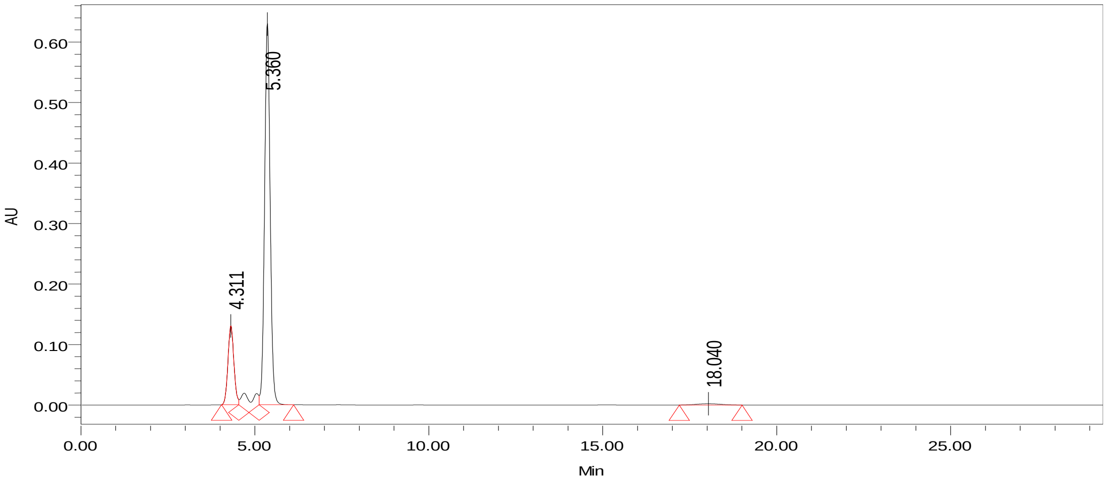

Entry	Retention time	Area	Area (%)	Height	Int type
1	4.311	1553891	17.84	130527	bv
2	5.360	7046582	80.91	629989	vb
3	18.040	109144	1.25	2181	bb

Supplementary Fig. 70. HPLC chromatograms of compound **4v.**

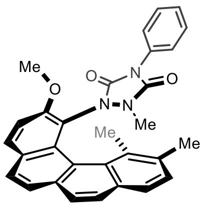

4w

HPLC condition: Chiralcel IA-H, *n*-hexane/EtOH (1‰ TFA) = 7/3, flow rate = 1.0 mL/min.

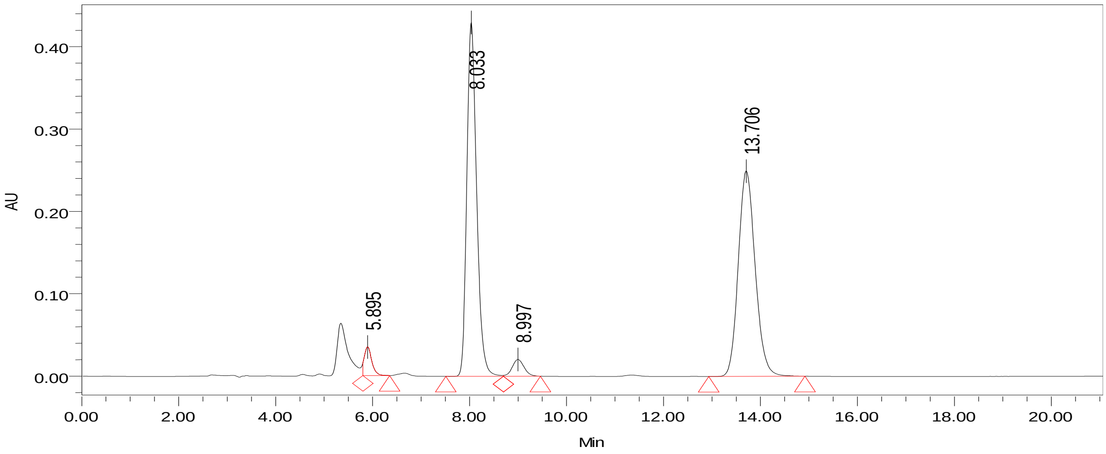

Entry	Retention time	Area	Area (%)	Height	Int type
1	5.895	369758	2.89	34884	vb
2	8.033	6048724	47.22	429728	bv
3	8.997	346663	2.71	20439	vb
4	13.706	6044560	47.19	249406	bb

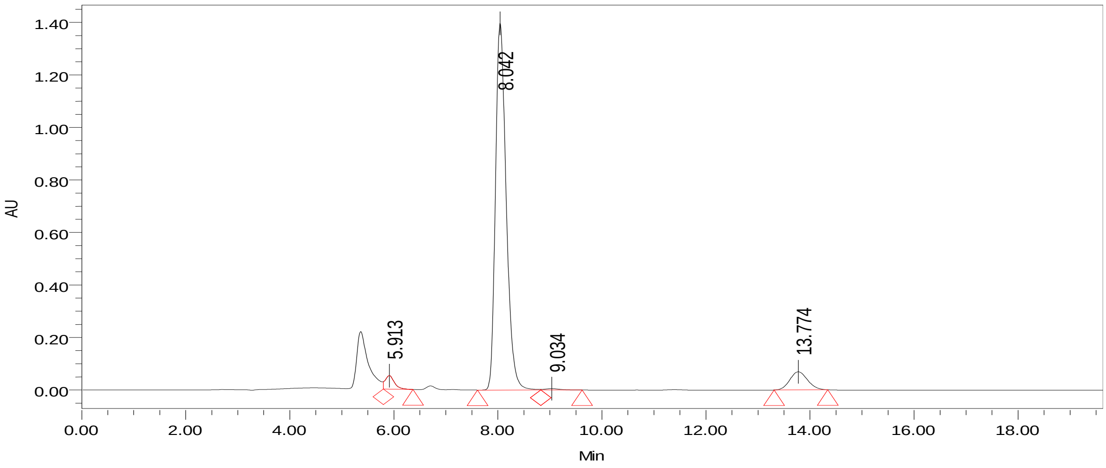

Entry	Retention time	Area	Area (%)	Height	Int type
1	5.913	633124	2.82	51855	vb
2	8.042	20047423	89.40	1396675	bv
3	9.034	107363	0.48	5314	vb
4	13.774	1636228	7.30	69146	bb

Supplementary Fig. 71. HPLC chromatograms of compound **4w.**

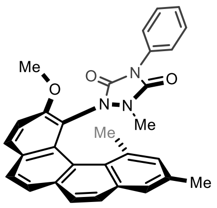

4x

HPLC condition: Chiralcel AD-H, *n*-hexane/EtOH (1‰ TFA) = 7/3, flow rate = 1.0 mL/min.

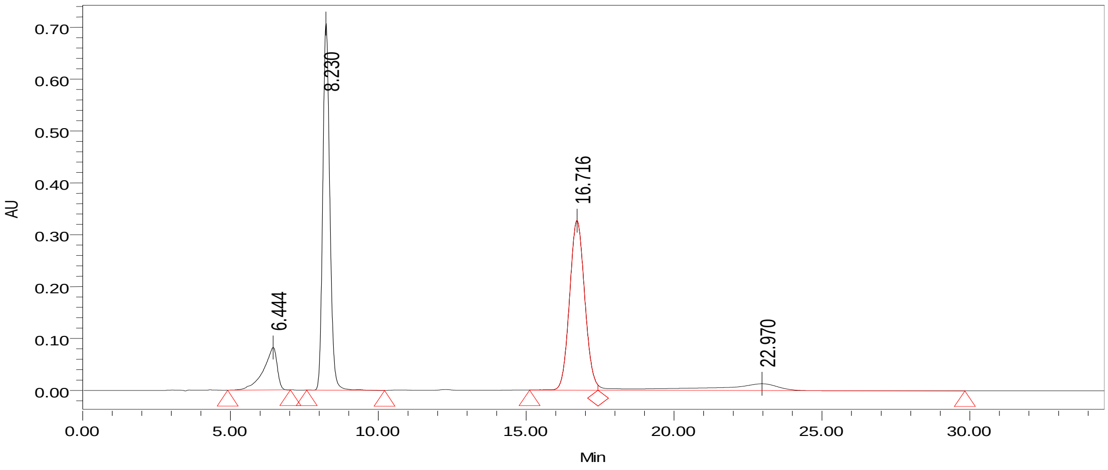

Entry	Retention time	Area	Area (%)	Height	Int type
1	6.444	2649531	9.44	82197	bb
2	8.230	11682028	41.63	706764	bb
3	16.716	11615842	41.40	326708	bv
4	22.970	2113090	7.53	12811	vb

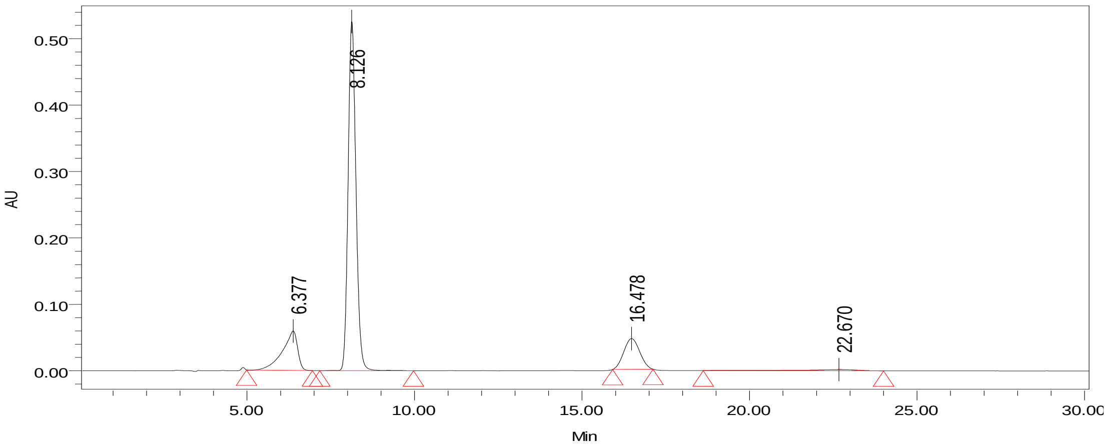

Entry	Retention time	Area	Area (%)	Height	Int type
1	6.377	1867939	15.33	59256	bb
2	8.126	8584727	70.45	525687	bb
3	16.478	1534534	12.59	46347	bb
4	22.670	198883	1.63	1819	bb

Supplementary Fig. 72. HPLC chromatograms of compound **4x.**

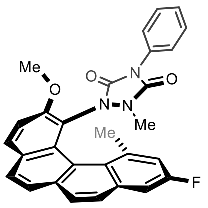

4y

HPLC condition: Chiralcel AD-H, *n*-hexane/EtOH (1‰ TFA) = 7/3, flow rate = 1.0 mL/min.

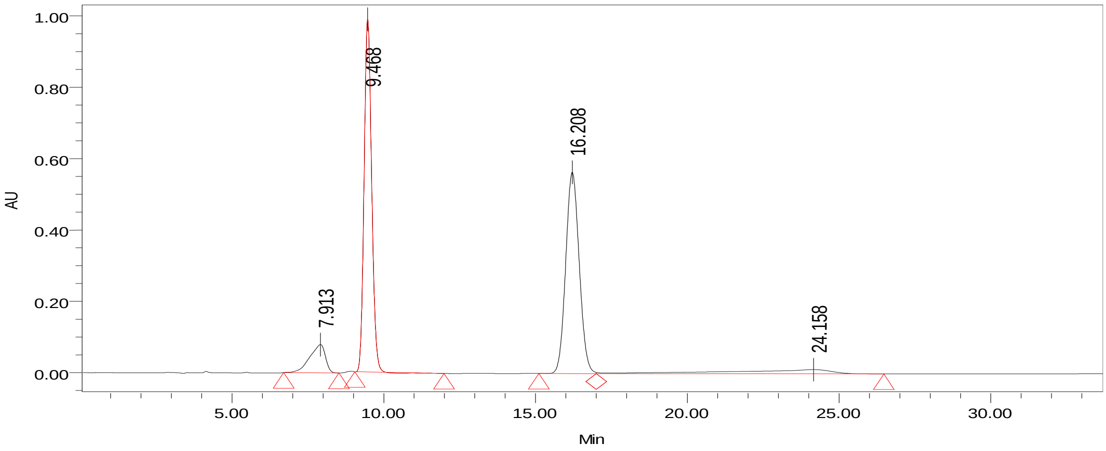

Entry	Retention time	Area	Area (%)	Height	Int type
1	7.913	2856238	7.11	79398	bb
2	9.468	17200688	42.82	988479	bb
3	16.208	17444640	43.42	564026	bv
4	24.158	2671741	6.65	11722	vb

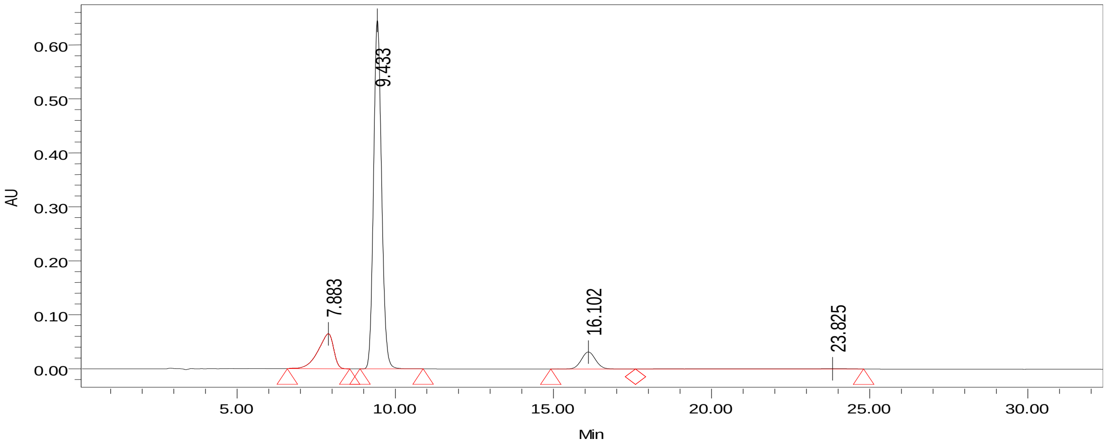

Entry	Retention time	Area	Area (%)	Height	Int type
1	7.883	2221439	15.10	64950	bb
2	9.433	11400767	77.49	645430	bb
3	16.102	979605	6.66	31546	bv
4	23.825	110180	0.75	535	vb

Supplementary Fig. 73. HPLC chromatograms of compound **4y.**

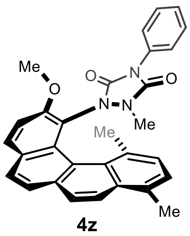

4z

HPLC condition: Chiralcel IA-H, *n*-hexane/*i*-PrOH (1‰ TFA) = 7/3, flow rate = 1.0 mL/min.

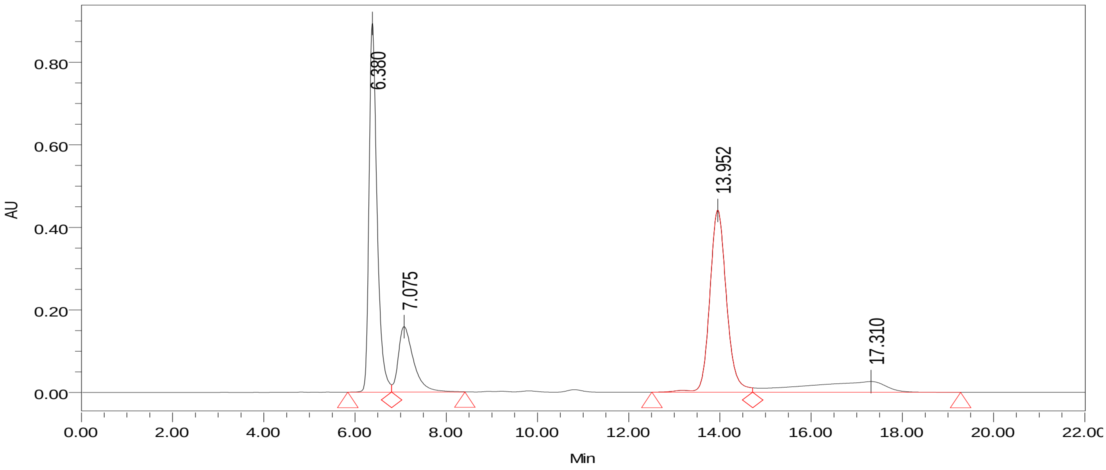

Entry	Retention time	Area	Area (%)	Height	Int type
1	6.380	10966309	37.65	894049	bv
2	7.075	3679316	12.63	158656	vb
3	13.952	11252705	38.63	440859	bv
4	17.310	3232267	11.10	26050	vb

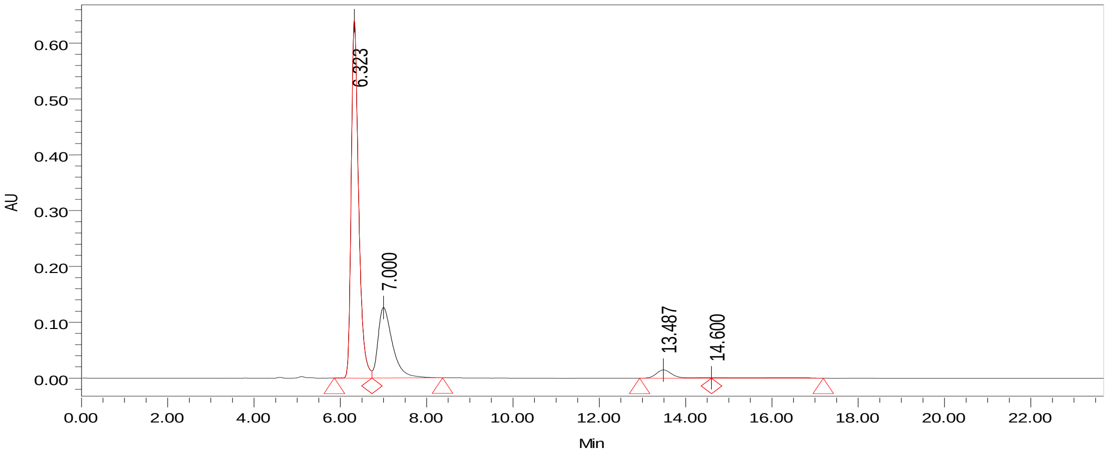

Entry	Retention time	Area	Area (%)	Height	Int type
1	6.323	7688018	69.10	640377	bv
2	7.000	2934826	26.38	126270	vb
3	13.487	393030	3.53	14850	bv
4	14.600	109621	0.99	999	vb

Supplementary Fig. 74. HPLC chromatograms of compound **4z.**

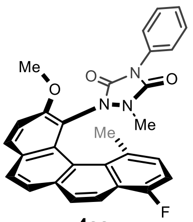

4aa

HPLC condition: Chiralcel IC-H, *n*-hexane/*i*-PrOH (1‰ TFA) = 7/3, flow rate = 1.0 mL/min.

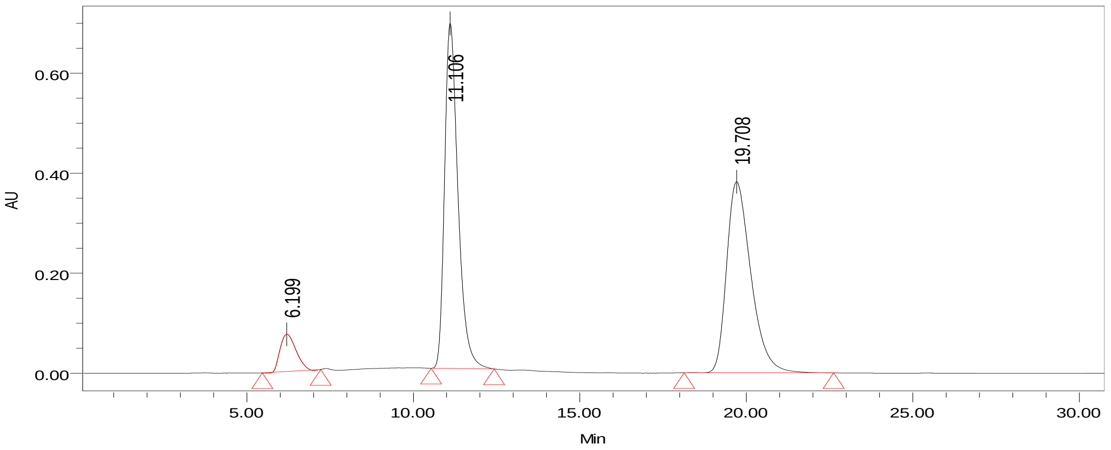

Entry	Retention time	Area	Area (%)	Height	Int type
1	6.199	2496127	6.05	74499	bb
2	11.106	19344224	46.88	690082	bb
3	19.708	19422512	47.07	382068	bb

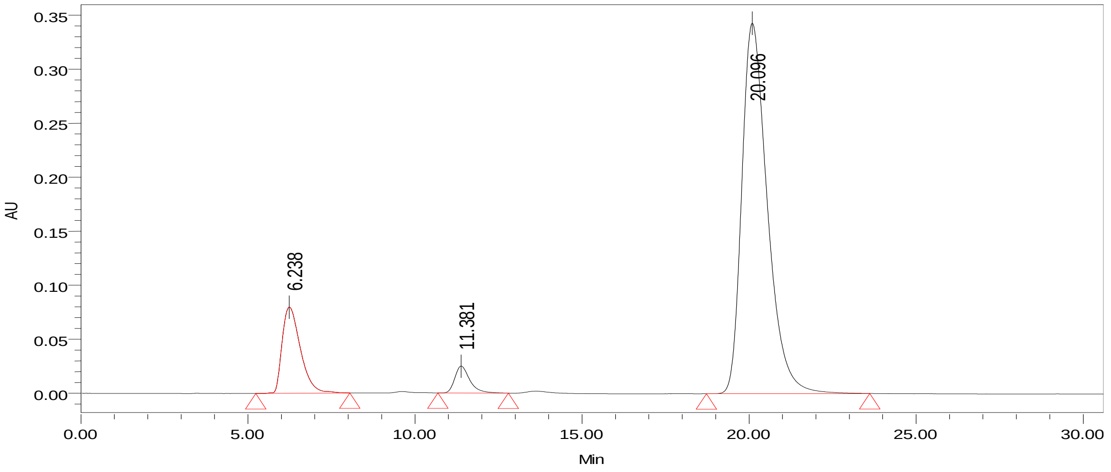

Entry	Retention time	Area	Area (%)	Height	Int type
1	6.238	3031129	13.73	79766	bb
2	11.381	778009	3.53	24952	bb
3	20.096	18260676	82.74	342932	bb

Supplementary Fig. 75. HPLC chromatograms of compound **4aa.**

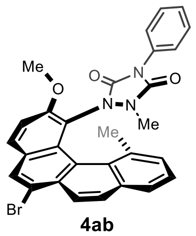

4ab

HPLC condition: Chiralcel IC-H, *n*-hexane/EtOH (1‰ TFA) = 7/3, flow rate = 1.0 mL/min.

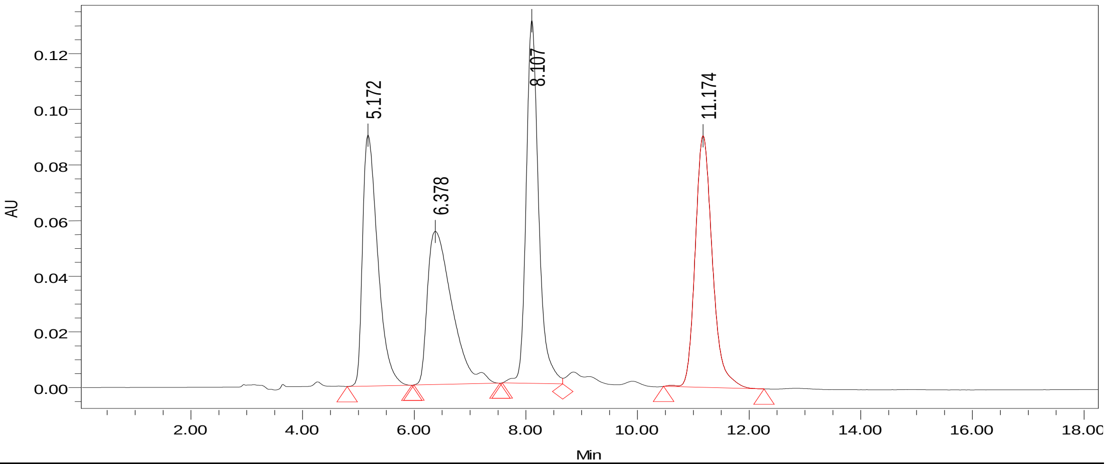

Entry	Retention time	Area	Area (%)	Height	Int type
1	5.172	1705223	23.19	90203	bb
2	6.378	1682698	22.88	55037	bb
3	8.107	2030082	27.61	130419	bv
4	11.174	1935949	26.33	90452	bb

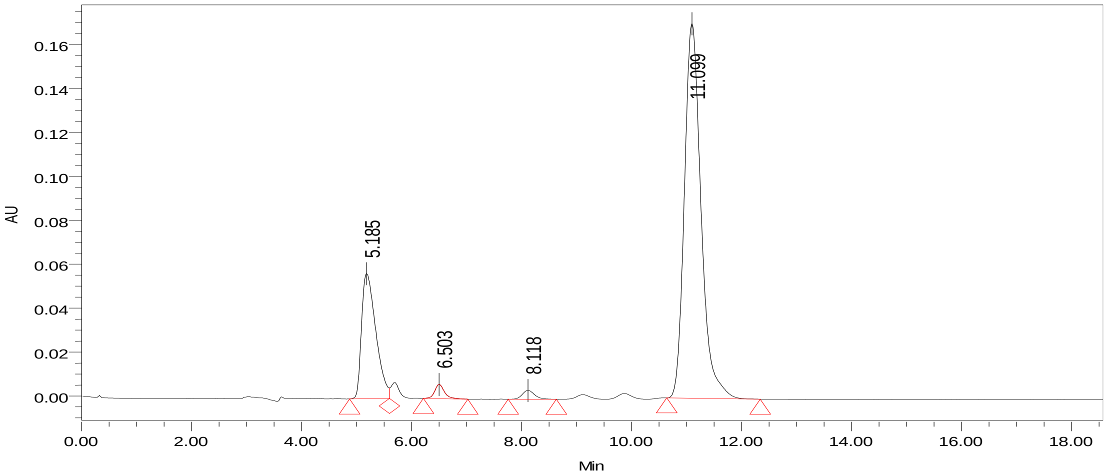

Entry	Retention time	Area	Area (%)	Height	Int type
1	5.185	1029455	21.58	56950	bv
2	6.503	81306	1.70	6505	bb
3	8.118	63040	1.32	4033	bb
4	11.099	3595778	75.39	170562	bb

Supplementary Fig. 76. HPLC chromatograms of compound **4ab.**

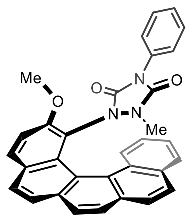

4ac

HPLC condition: Chiralcel AD-H, *n*-hexane/*i*-PrOH (1‰ TFA) = 7/3, flow rate = 1.0 mL/min.

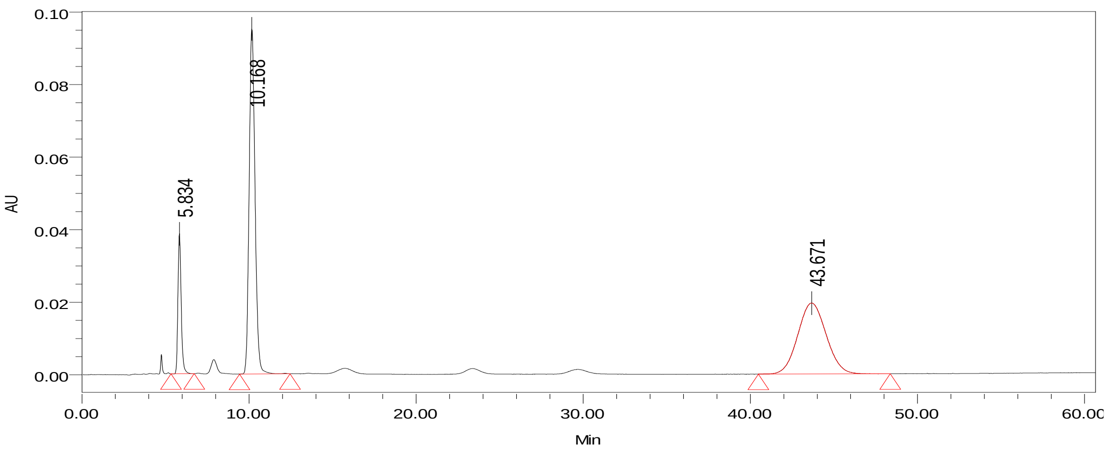

Entry	Retention time	Area	Area (%)	Height	Int type
1	5.834	528499	10.26	38601	bb
2	10.168	2318504	44.99	95141	bb
3	43.671	2306141	44.75	19566	bb

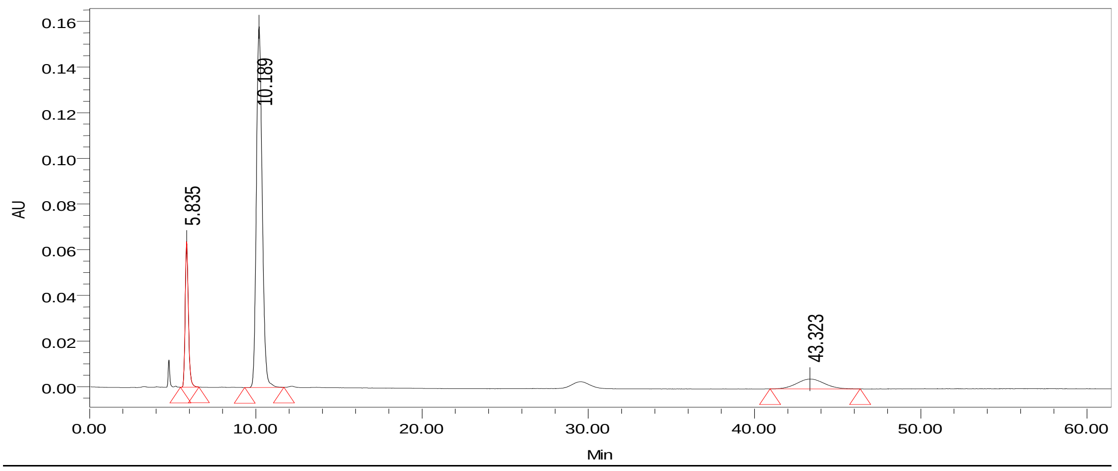

Entry	Retention time	Area	Area (%)	Height	Int type
1	5.835	859745	16.58	63701	bb
2	10.189	3831900	73.89	158071	bb
3	43.323	494457	9.53	4288	bb

Supplementary Fig. 77. HPLC chromatograms of compound **4ac.**

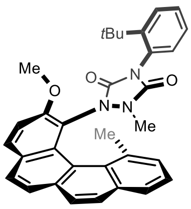

4ad

HPLC condition: Chiralcel AD-H, *n*-hexane/*i*-PrOH (1‰ TFA) = 7/3, flow rate = 1.0 mL/min.

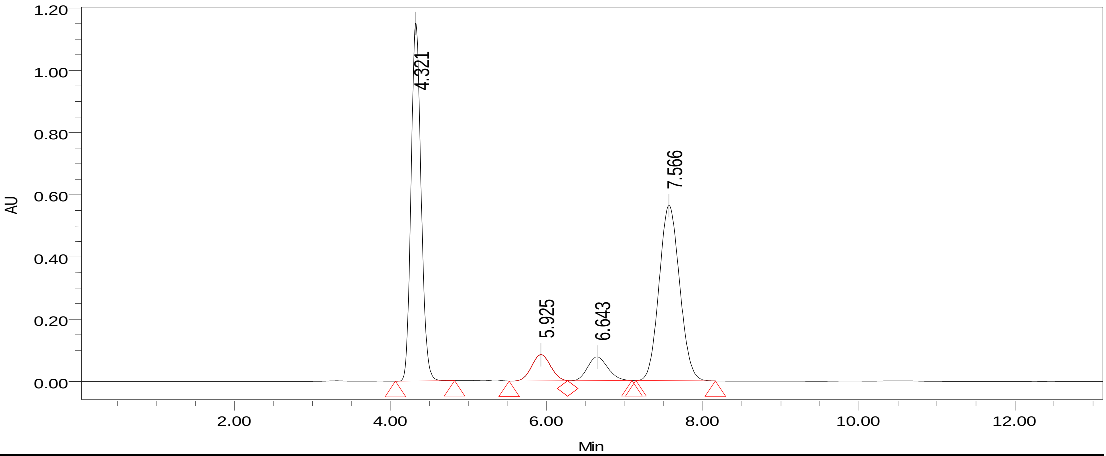

Entry	Retention time	Area	Area (%)	Height	Int type
1	4.321	10284498	43.95	1151460	bb
2	5.925	1422953	6.08	84447	bv
3	6.643	1365099	5.83	76130	vb
4	7.566	10330451	44.14	563099	bb

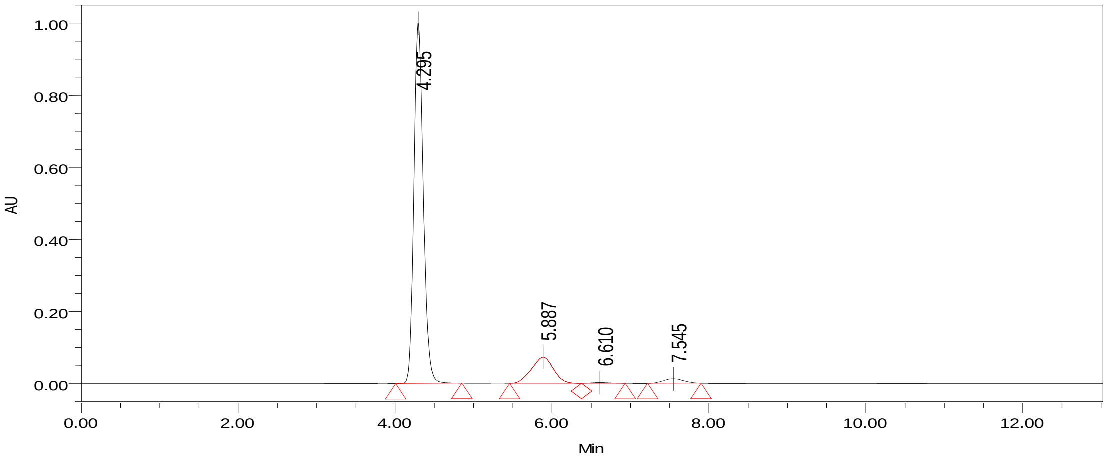

Entry	Retention time	Area	Area (%)	Height	Int type
1	4.295	8344285	83.38	1001412	bb
2	5.887	1401781	14.01	72425	bv
3	6.610	36482	0.36	2275	vb
4	7.545	225360	2.25	12573	bb

Supplementary Fig. 78. HPLC chromatograms of compound **4ad.**

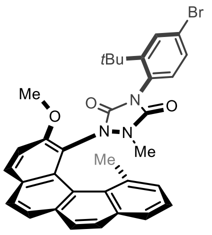

4ae

HPLC condition: Chiralcel IA-H, *n*-hexane/EtOH (1‰ TFA) = 7/3, flow rate = 1.0 mL/min.

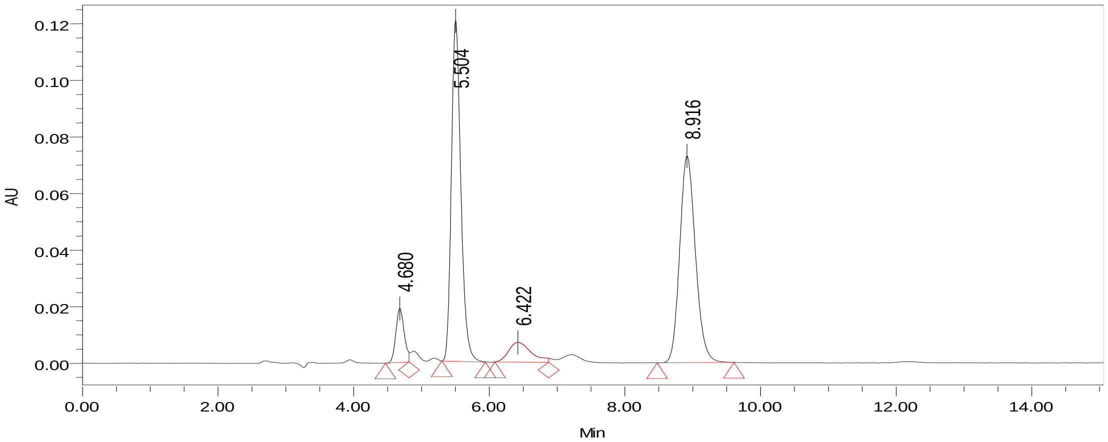

Entry	Retention time	Area	Area (%)	Height	Int type
1	4.680	162482	6.09	19099	bv
2	5.504	1167293	43.76	120553	bb
3	6.422	160347	6.01	6971	bv
4	8.916	1177635	44.14	73087	bb

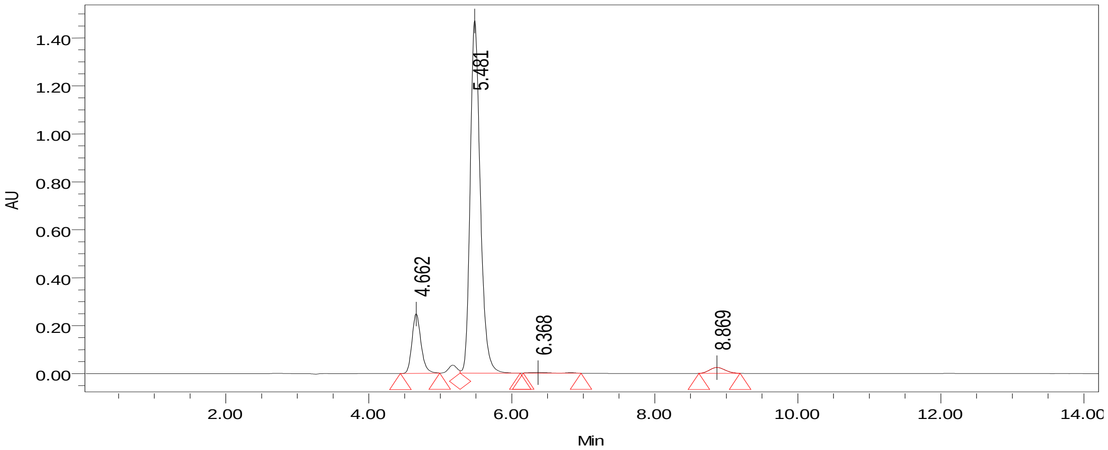

Entry	Retention time	Area	Area (%)	Height	Int type
1	4.662	2176888	12.80	248141	bb
2	5.481	14401843	84.71	1469898	vb
3	6.368	53704	0.32	2083	bb
4	8.869	369912	2.18	24428	bb

Supplementary Fig. 79. HPLC chromatograms of compound **4ae.**

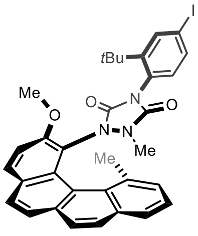

4af

HPLC condition: Chiralcel IA-H, *n*-hexane/EtOH (1‰ TFA) = 7/3, flow rate = 1.0 mL/min.

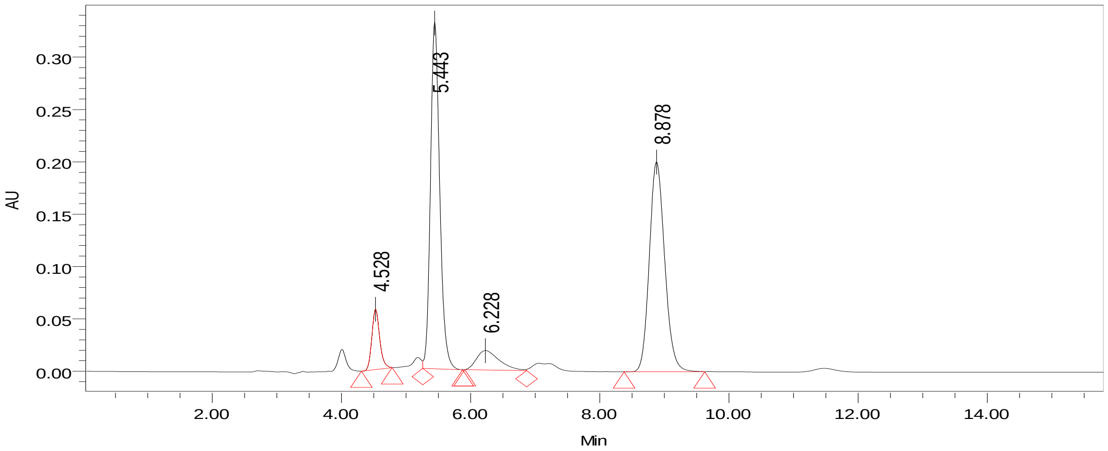

Entry	Retention time	Area	Area (%)	Height	Int type
1	4.528	487690	6.46	57641	bb
2	5.443	3295535	43.63	330752	vb
3	6.228	454039	6.01	18529	bv
4	8.878	3315490	43.90	200407	bb

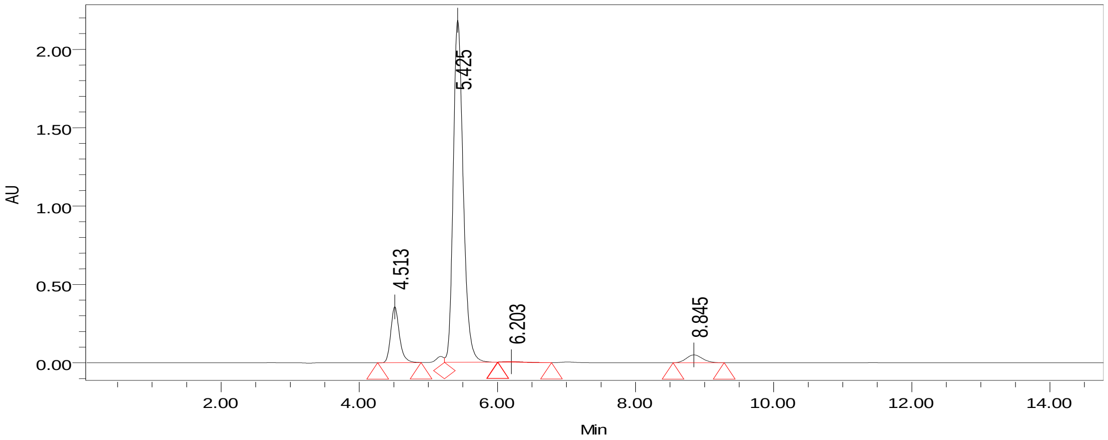

Entry	Retention time	Area	Area (%)	Height	Int type
1	4.513	3102173	11.94	356899	bb
2	5.425	22007389	84.68	2181764	vb
3	6.203	67910	0.26	3939	bb
4	8.845	811537	3.12	50223	bb

Supplementary Fig. 80. HPLC chromatograms of compound **4af.**

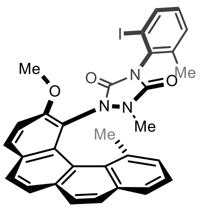

4ag

HPLC condition: Chiralcel IA-H, *n*-hexane/EtOH (1‰ TFA) = 7/3, flow rate = 1.0 mL/min.

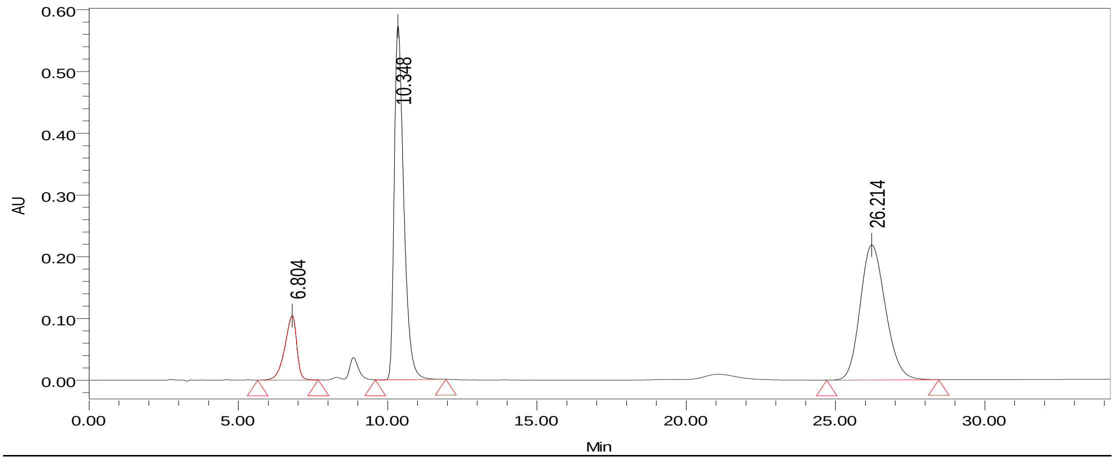

Entry	Retention time	Area	Area (%)	Height	Int type
1	6.804	2891542	10.13	104355	bb
2	10.348	12788693	44.79	572548	bb
3	26.214	12873980	45.09	218696	bb

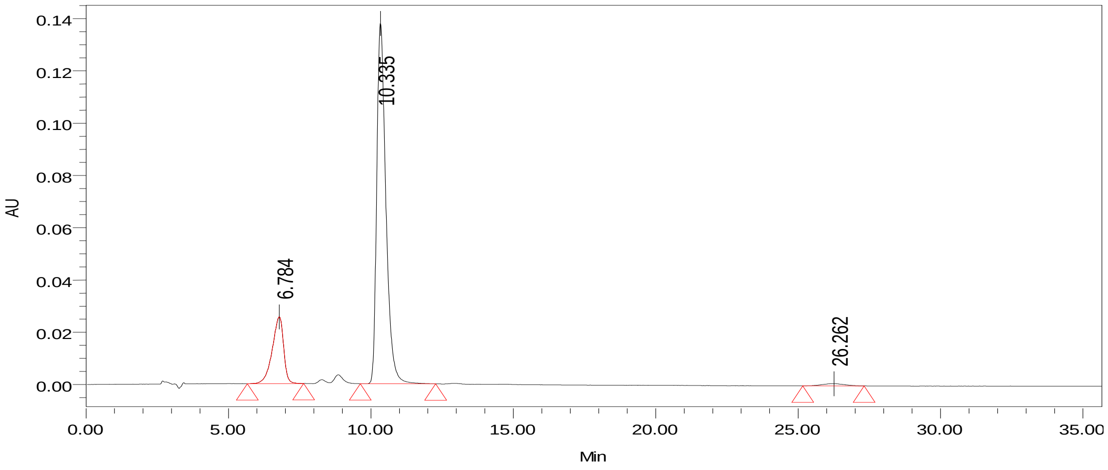

Entry	Retention time	Area	Area (%)	Height	Int type
1	6.784	704702	18.35	25555	bb
2	10.335	3090341	80.48	137882	bb
3	26.262	44902	1.17	810	bb

Supplementary Fig. 81. HPLC chromatograms of compound **4ag.**

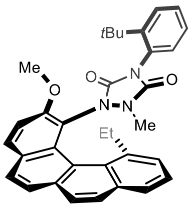

4ah

HPLC condition: Chiralcel AD-H, *n*-hexane/*i*-PrOH (1‰ TFA) = 8/2, flow rate = 1.0 mL/min.

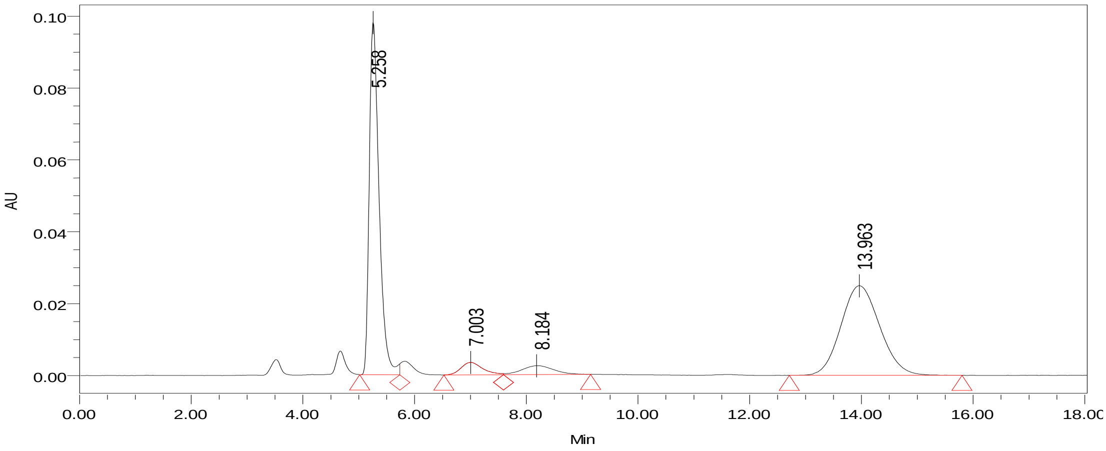

Entry	Retention time	Area	Area (%)	Height	Int type
1	5.258	1202296	46.34	97996	bv
2	7.003	92515	3.57	3453	bv
3	8.184	97365	3.75	2464	vb
4	13.963	1202235	46.34	24926	bb

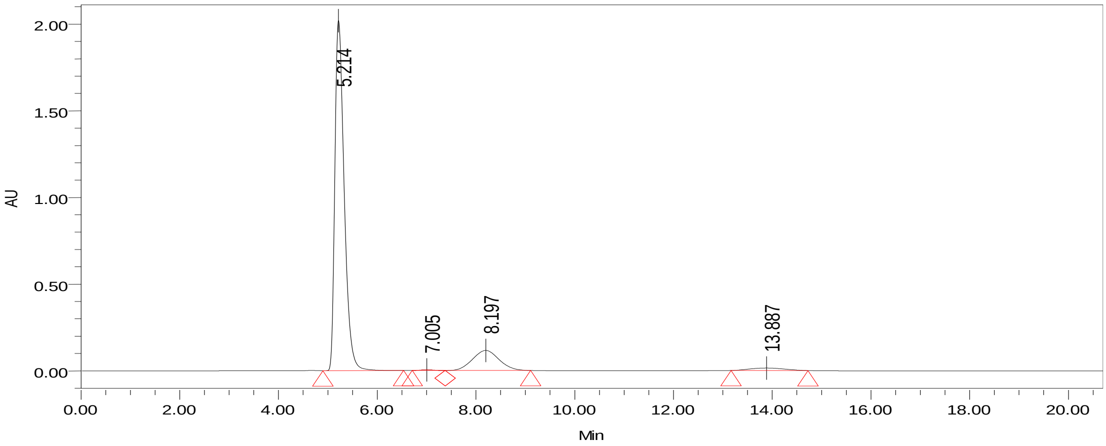

Entry	Retention time	Area	Area (%)	Height	Int type
1	5.214	26551058	83.89	2019950	bb
2	7.005	65732	0.21	3417	bv
3	8.197	4328205	13.68	115659	vb
4	13.887	703536	2.22	14281	bb

Supplementary Fig. 82. HPLC chromatograms of compound **4ah.**

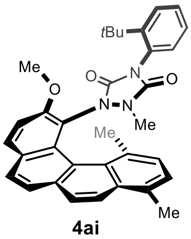

4ai

HPLC condition: Chiralcel AD-H, *n*-hexane/*i*-PrOH (1‰ TFA) = 8/2, flow rate = 1.0 mL/min.

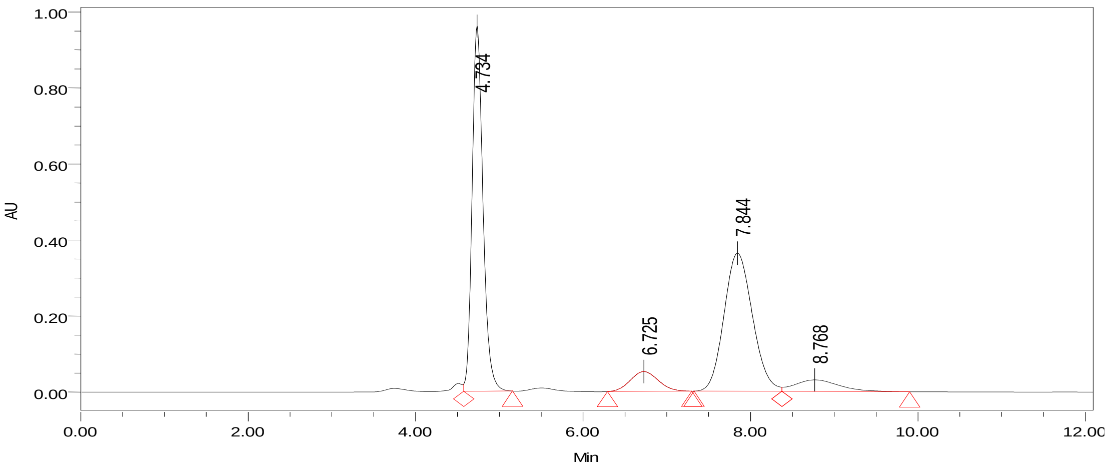

Entry	Retention time	Area	Area (%)	Height	Int type
1	4.734	8690229	44.33	961228	vb
2	6.725	1205527	6.15	52096	bb
3	7.844	8581800	43.78	363430	bv
4	8.768	1125009	5.74	30511	vb

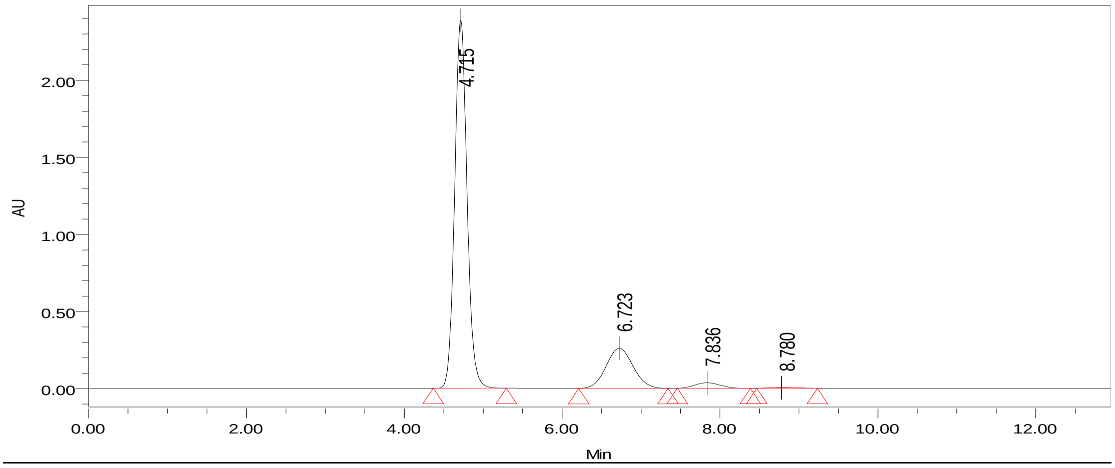

Entry	Retention time	Area	Area (%)	Height	Int type
1	4.715	25992076	78.63	2388053	bb
2	6.723	6169846	18.67	260582	bb
3	7.836	796717	2.41	35562	bb
4	8.780	96732	0.29	3551	bb

Supplementary Fig. 83. HPLC chromatograms of compound **4ai.**

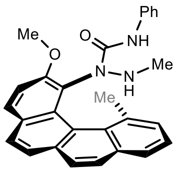

5

HPLC condition: Chiralcel IA-H, *n*-hexane/*i*-PrOH = 8/2, flow rate = 1.0 mL/min.

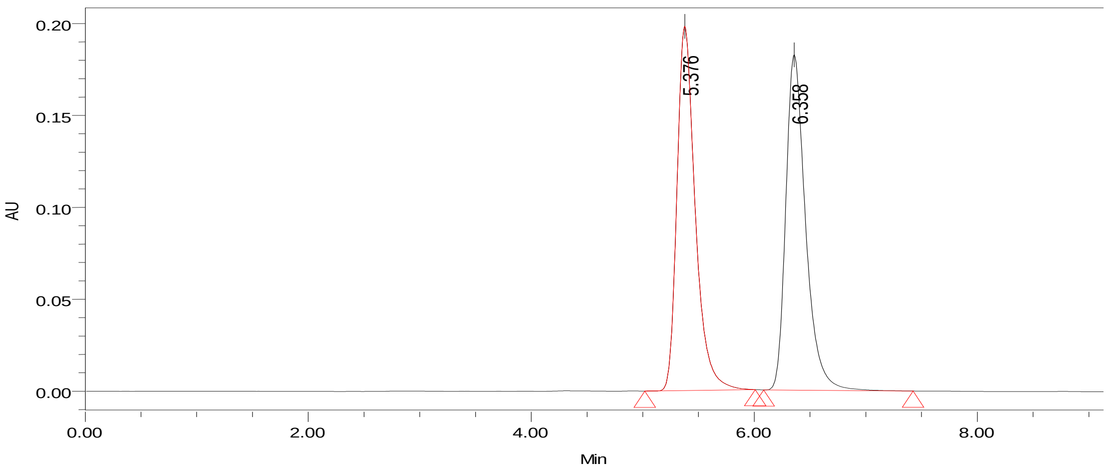

Entry	Retention time	Area	Area (%)	Height	Int type
1	5.376	2261310	50.06	198264	bb
2	6.358	2255954	49.94	182532	bb

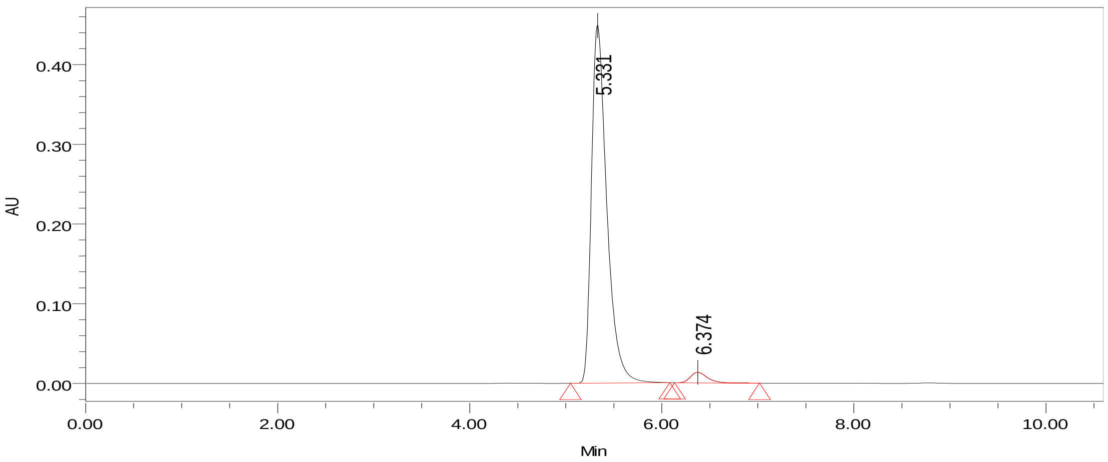

Entry	Retention time	Area	Area (%)	Height	Int type
1	5.331	5000844	96.83	449237	bb
2	6.374	163555	3.17	13310	bb

Supplementary Fig. 84. HPLC chromatograms of compound **5.**

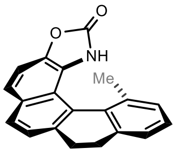

6

HPLC condition: Chiralcel IA-H, *n*-hexane/*i*-PrOH = 8/2, flow rate = 1.0 mL/min.

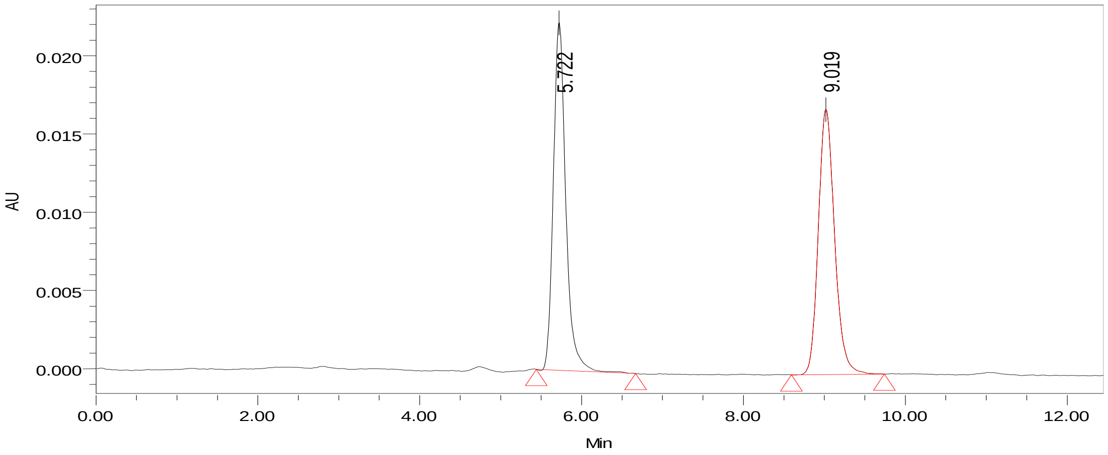

Entry	Retention time	Area	Area (%)	Height	Int type
1	5.722	237304	49.95	22249	bb
2	9.019	237767	50.05	16954	bb

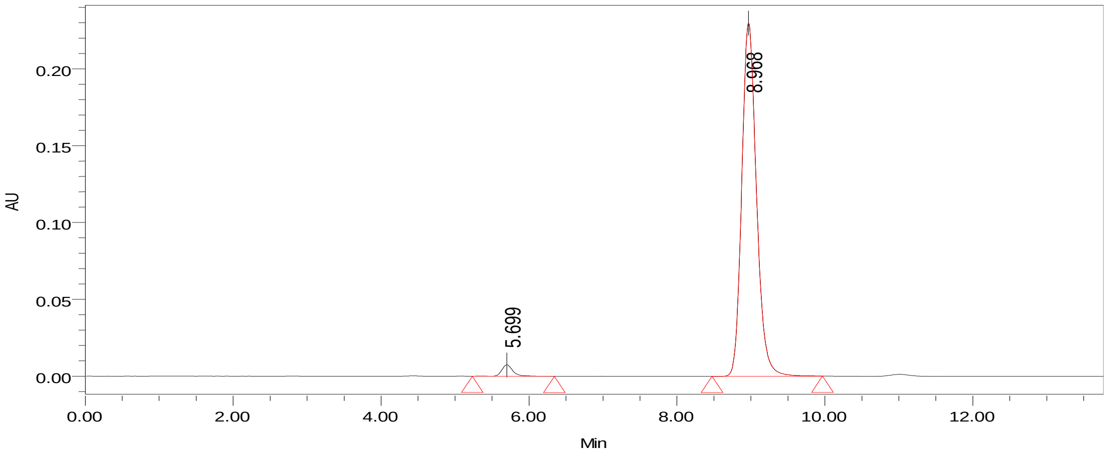

Entry	Retention time	Area	Area (%)	Height	Int type
1	5.699	77775	2.39	7428	bb
2	8.968	3176132	97.61	230021	bb

Supplementary Fig. 85. HPLC chromatograms of compound **6.**

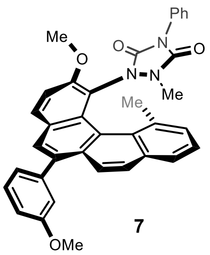

7

HPLC condition: Chiralcel IC-H, *n*-hexane/EtOH (1‰ TFA) = 7/3, flow rate = 1.0 mL/min.

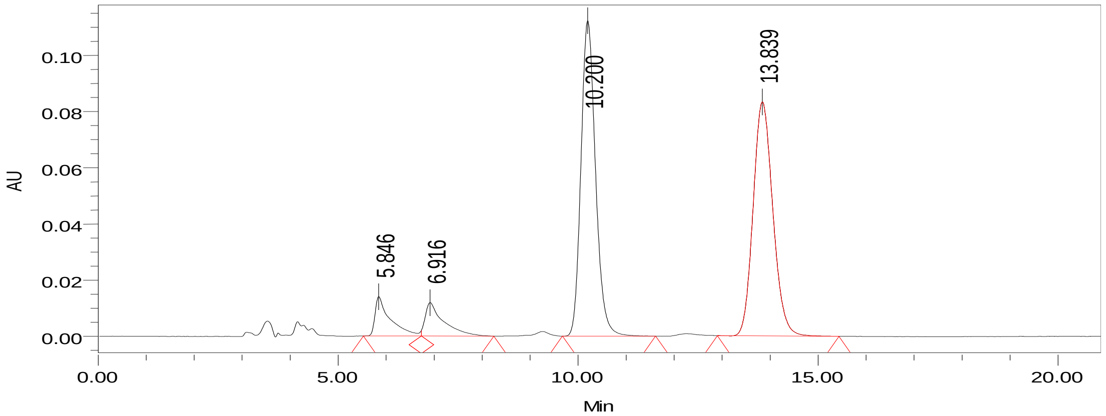

Entry	Retention time	Area	Area (%)	Height	Int type
1	5.846	311099	5.78	14051	bv
2	6.916	315791	5.87	11926	vb
3	10.200	2370977	44.09	112313	bb
4	13.839	2380077	44.26	83304	bb

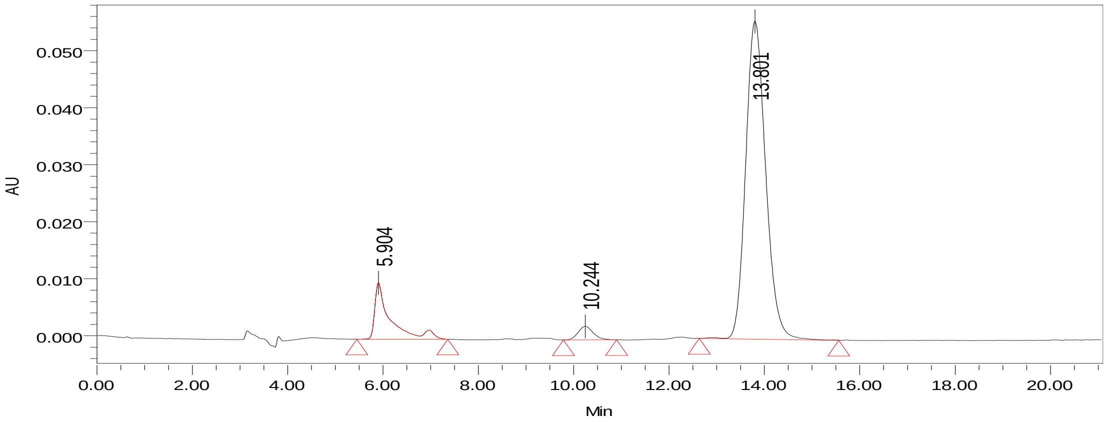

Entry	Retention time	Area	Area (%)	Height	Int type
1	5.904	224380	11.77	9904	bb
2	10.244	53146	2.79	2388	bb
3	13.801	1628798	85.44	55766	bb

Supplementary Fig. 86. HPLC chromatograms of compound **7.**

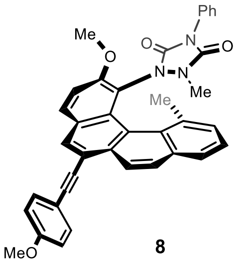

8

HPLC condition: Chiralcel IC-H, *n*-hexane/EtOH (1‰ TFA) = 7/3, flow rate = 1.0 mL/min.

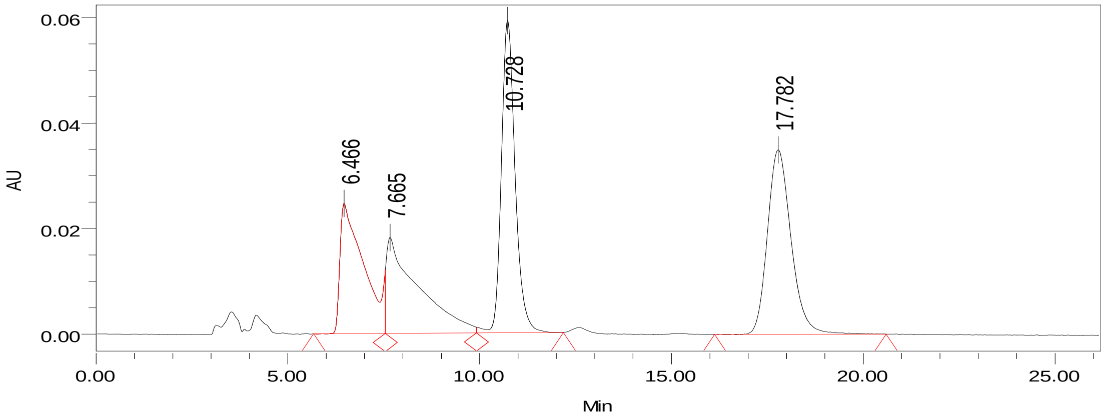

Entry	Retention time	Area	Area (%)	Height	Int type
1	6.466	1056638	20.76	24686	bv
2	7.665	1052003	20.67	18186	vv
3	10.728	1480913	29.10	59182	vb
4	17.782	1499091	29.46	34959	bb

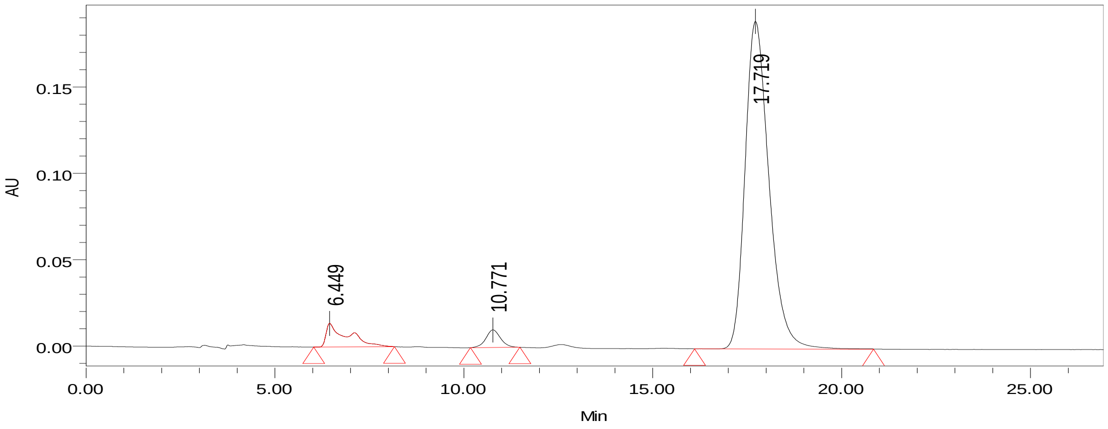

Entry	Retention time	Area	Area (%)	Height	Int type
1	6.449	540373	5.90	13724	bb
2	10.771	273869	2.99	10192	bb
3	17.719	8350770	91.12	189594	bb

Supplementary Fig. 87. HPLC chromatograms of compound **8.**

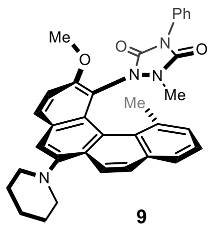

9

HPLC condition: Chiralcel IC-H, *n*-hexane/EtOH (1‰ TFA) = 7/3, flow rate = 1.0 mL/min.

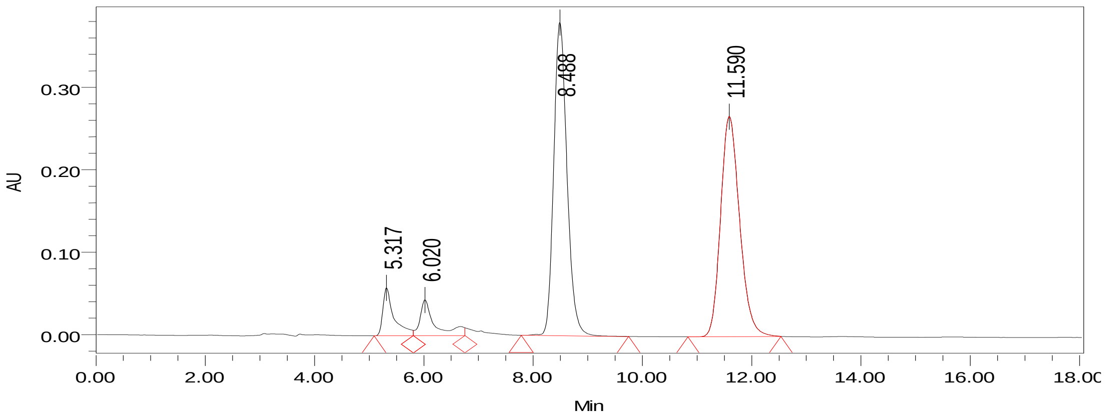

Entry	Retention time	Area	Area (%)	Height	Int type
1	5.317	826650	5.68	58196	bv
2	6.020	824000	5.66	43405	vv
3	8.488	6431751	44.20	380194	bb
4	11.590	6469000	44.46	267082	bb

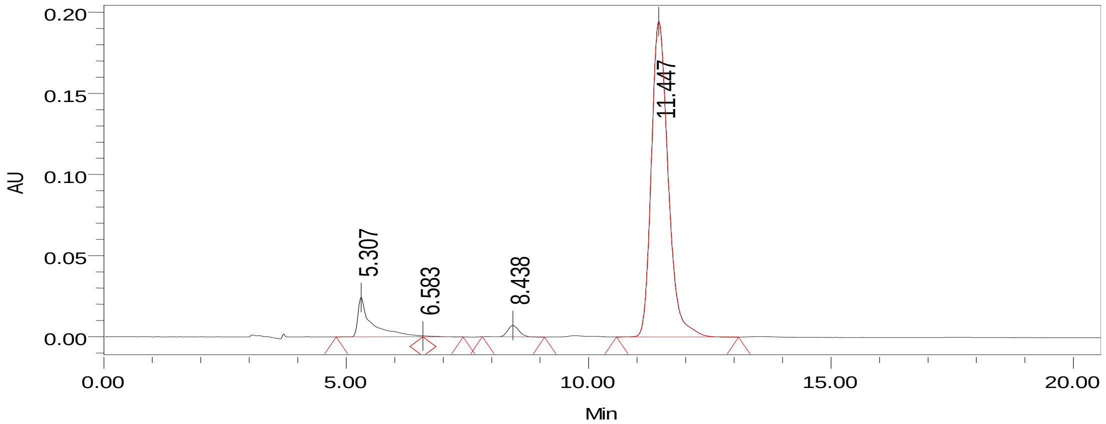

Entry	Retention time	Area	Area (%)	Height	Int type
1	5.307	495549	9.30	24360	bv
2	6.583	12717	0.24	706	vb
3	8.438	117468	2.20	7019	bb
4	11.447	4705351	88.26	194580	bb

Supplementary Fig. 88. HPLC chromatograms of compound **9.**

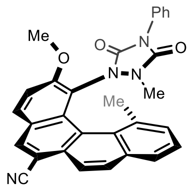

10

HPLC condition: Chiralcel IA-H, *n*-hexane/EtOH (1‰ TFA) = 1/1, flow rate = 1.0 mL/min.

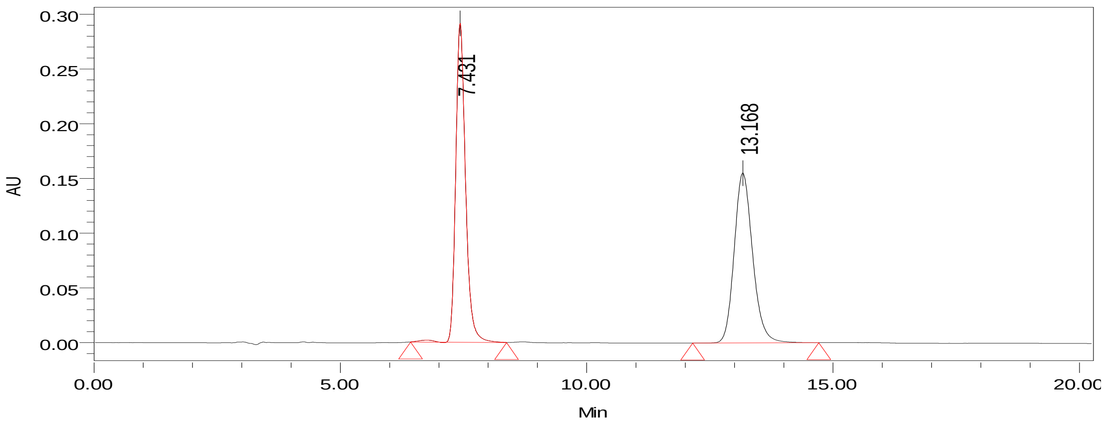

Entry	Retention time	Area	Area (%)	Height	Int type
1	7.431	4065181	50.14	291337	bb
2	13.168	4042736	49.86	155215	bb

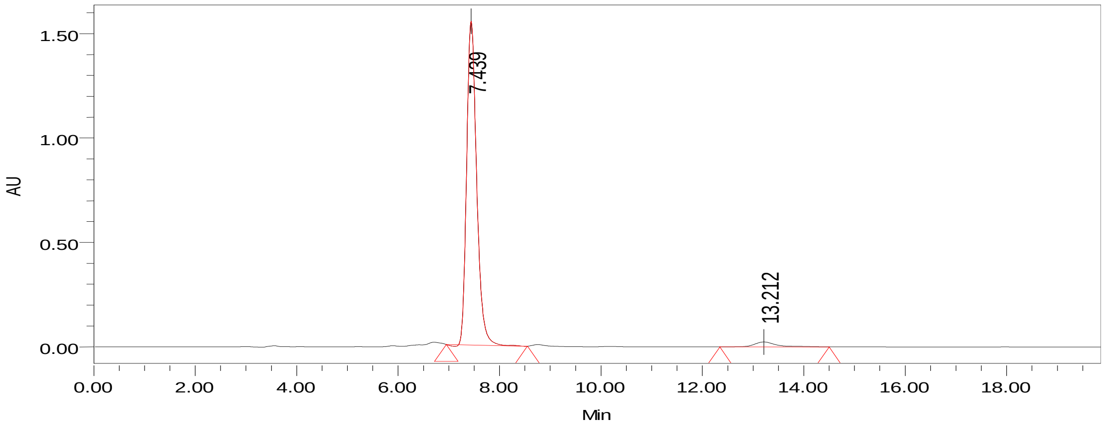

Entry	Retention time	Area	Area (%)	Height	Int type
1	7.439	21393760	96.86	1551580	bb
2	13.212	692859	3.14	23262	bb

Supplementary Fig. 89. HPLC chromatograms of compound **10.**

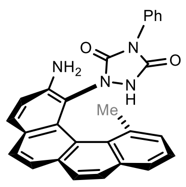

3a'

HPLC condition: Chiralcel AD-H, *n*-hexane/*i*-PrOH = 1/1, flow rate = 1.0 mL/min.

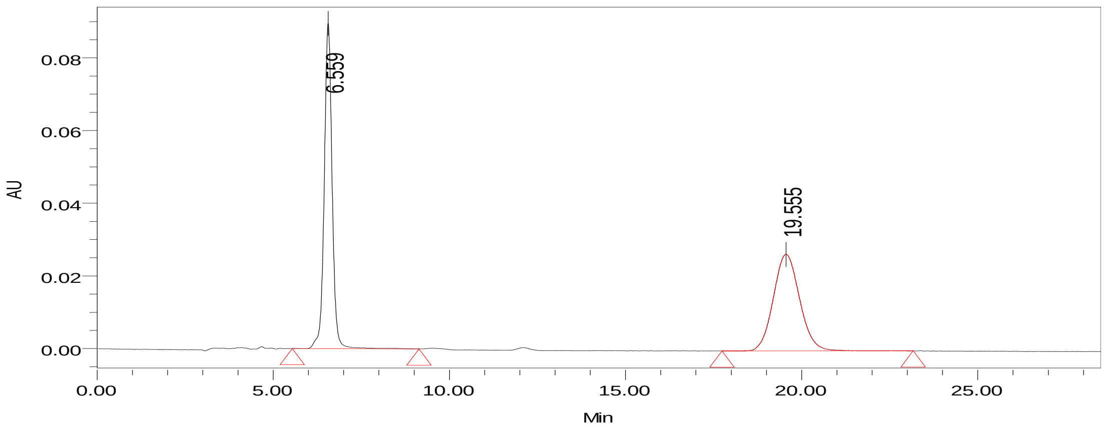

Entry	Retention time	Area	Area (%)	Height	Int type
1	6.559	1401522	50.43	89464	bb
2	19.555	1377405	49.57	26586	bb

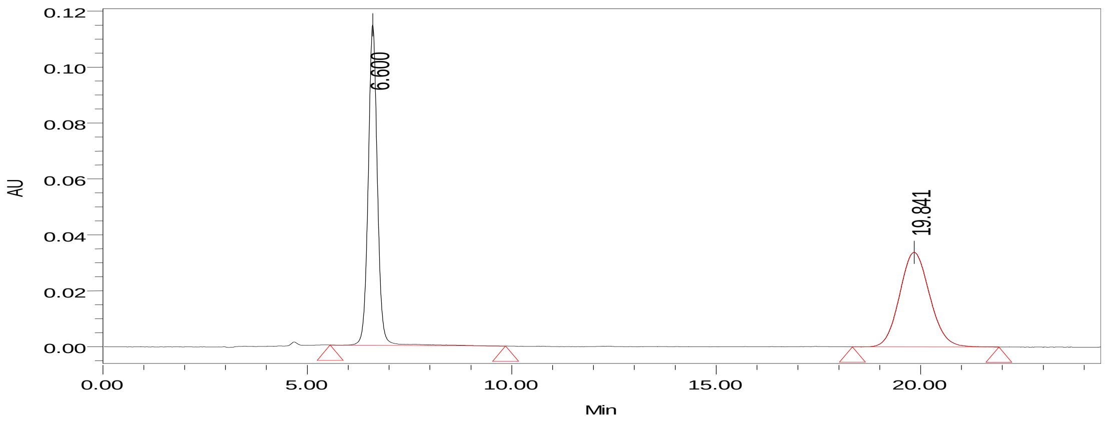

Entry	Retention time	Area	Area (%)	Height	Int type
1	6.600	1804619	51.00	114510	bb
2	19.841	1733803	49.00	33815	bb

Supplementary Fig. 90. HPLC chromatograms of compound **3a'.**

2.10 NMR spectra

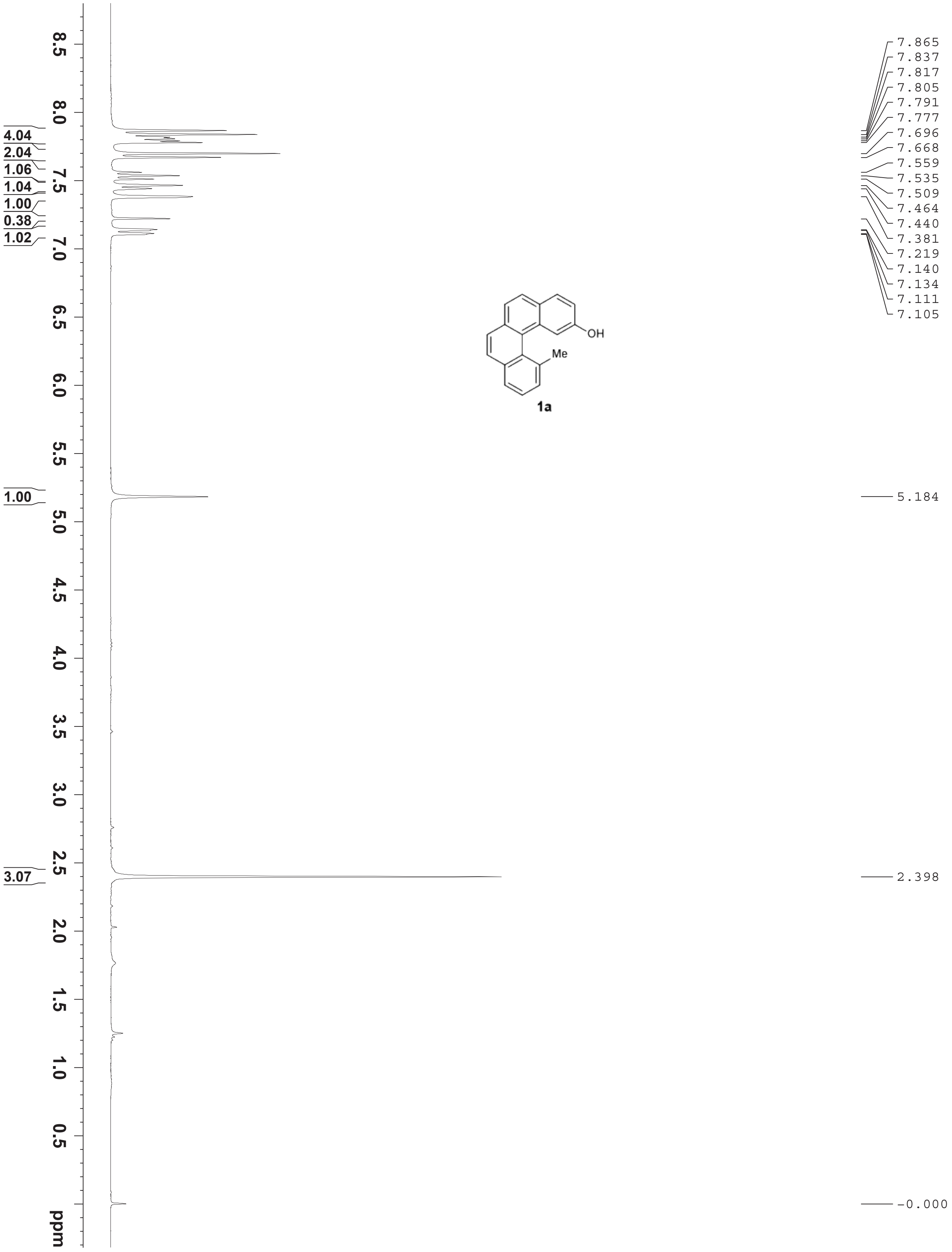

Supplementary Fig. 91. ^{1}H NMR of compound **1a** (300 MHz, $CDCl_3$)

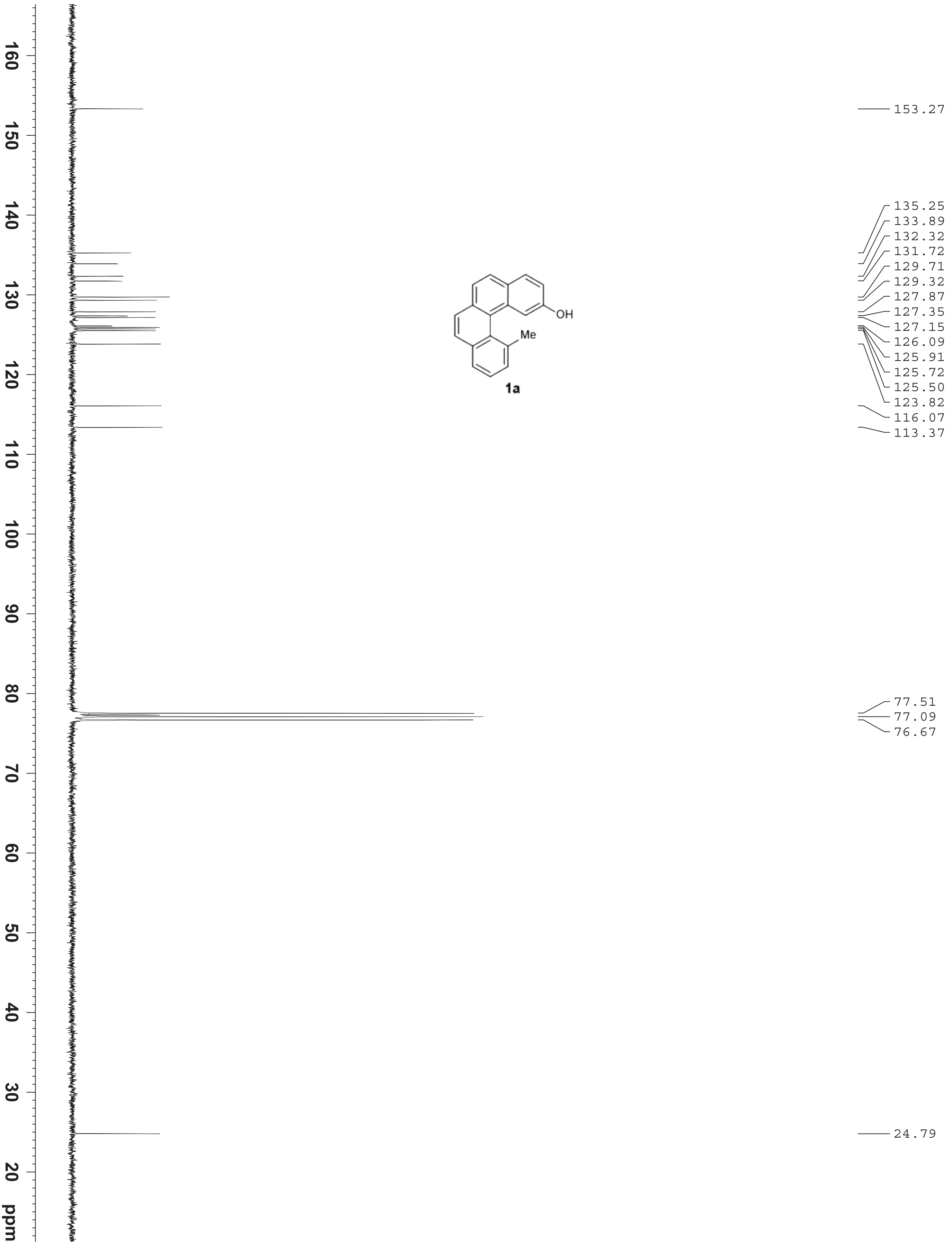

Supplementary Fig. 92. ^{13}C NMR of compound **1a** (75 MHz, $CDCl_3$)

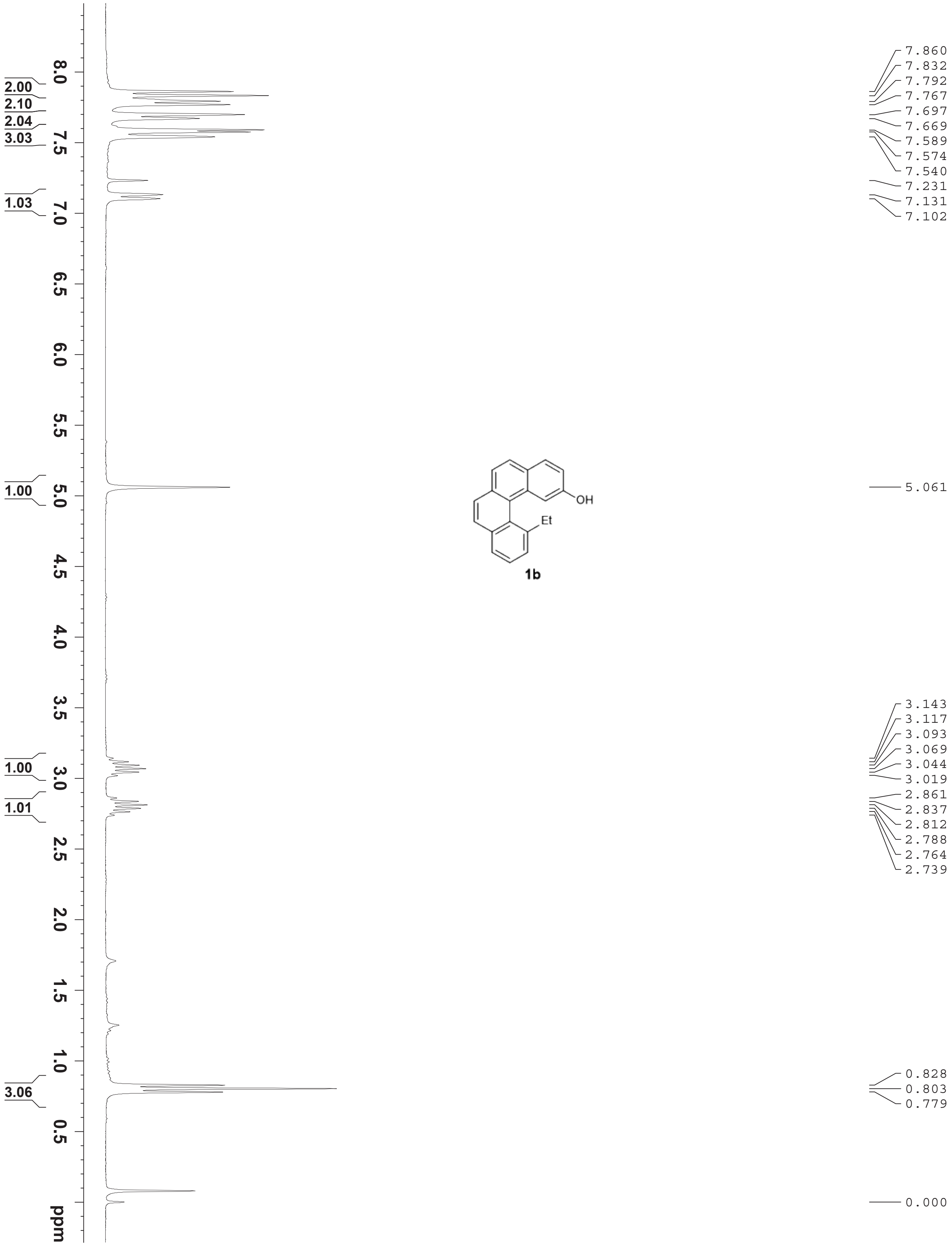

Supplementary Fig. 93. ^{1}H NMR of compound **1b** (300 MHz, $CDCl_3$)

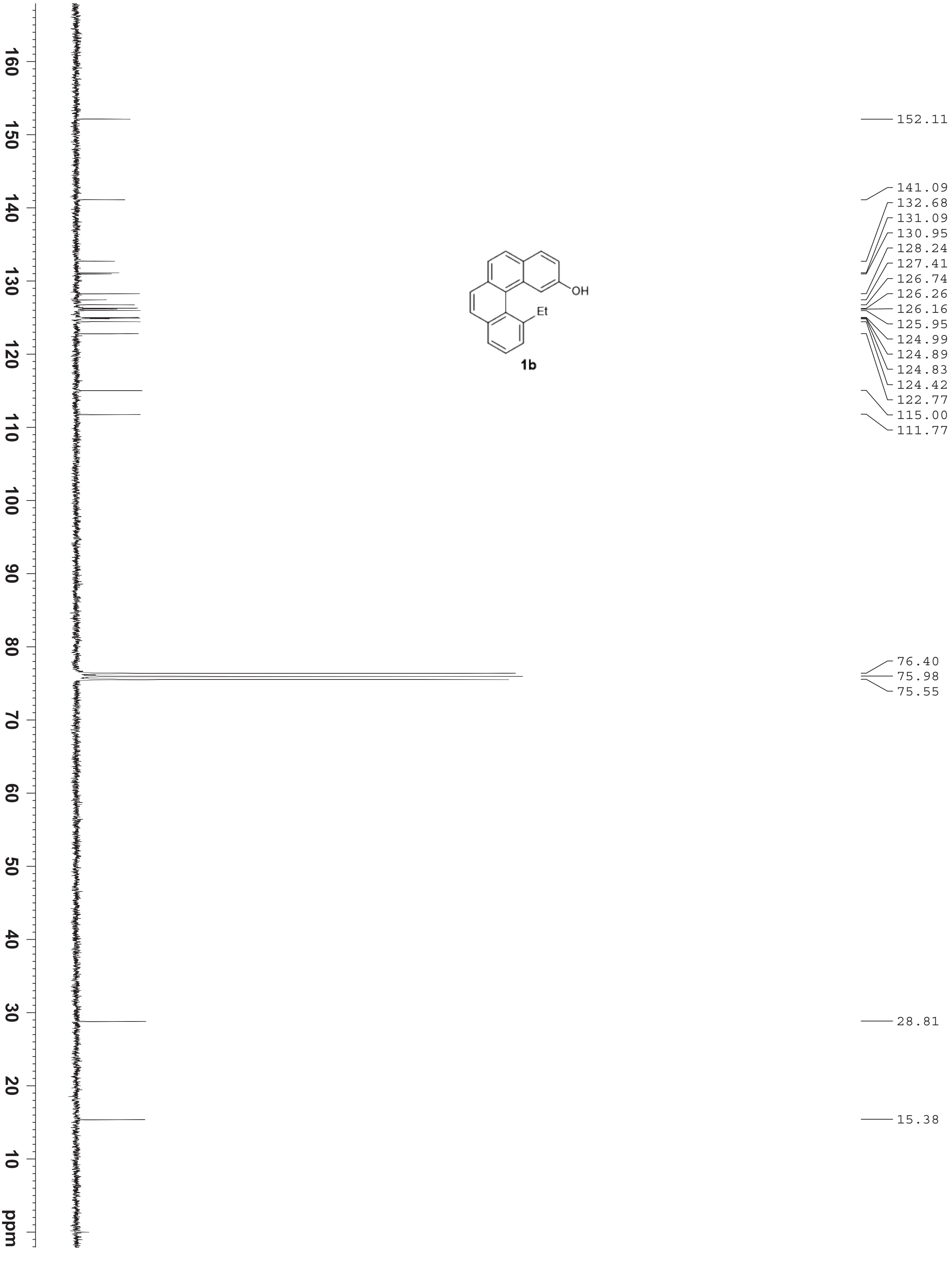

Supplementary Fig. 94. ^{13}C NMR of compound **1b** (75 MHz, $CDCl_3$)

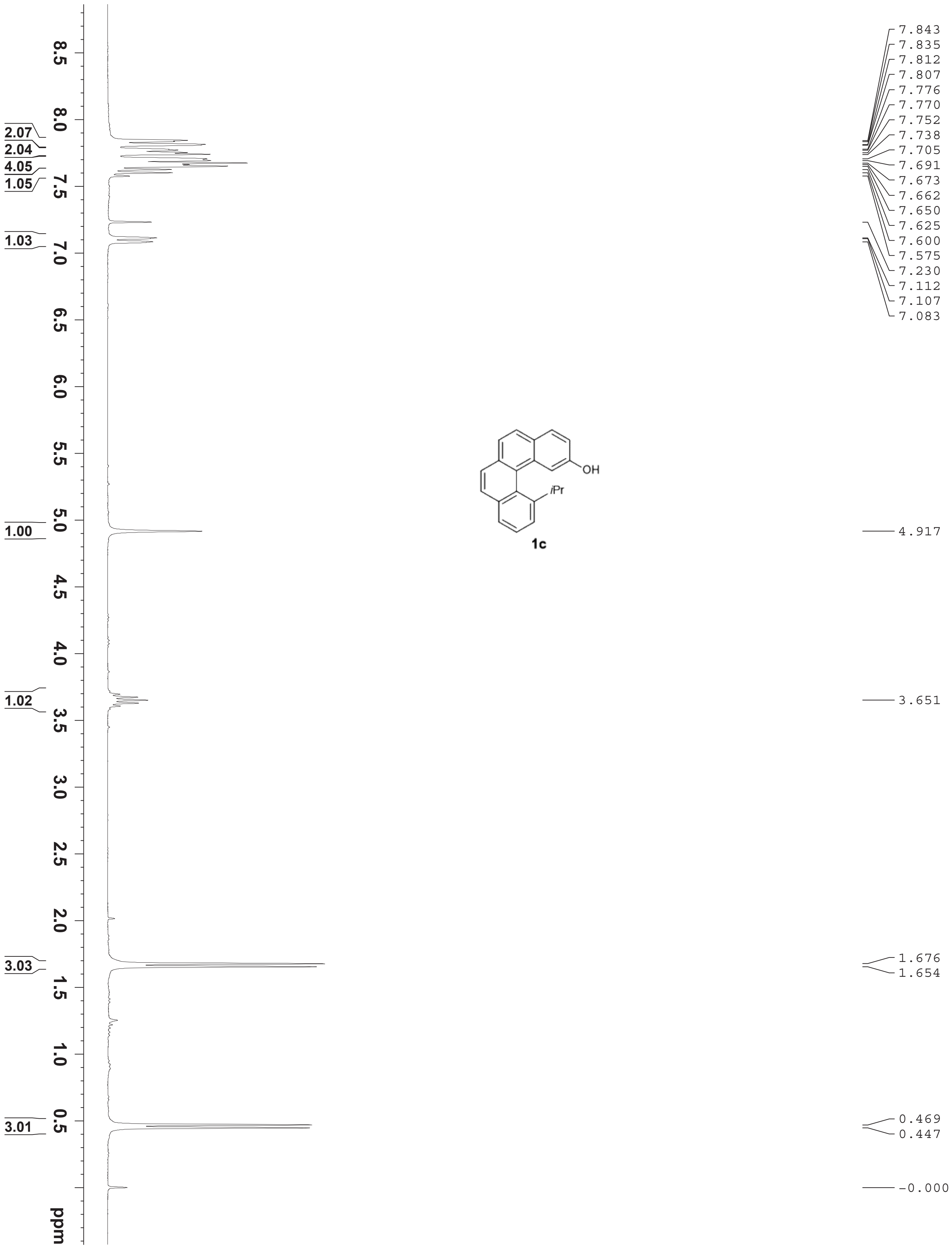

Supplementary Fig. 95. ^{1}H NMR of compound **1c** (300 MHz, $CDCl_3$)

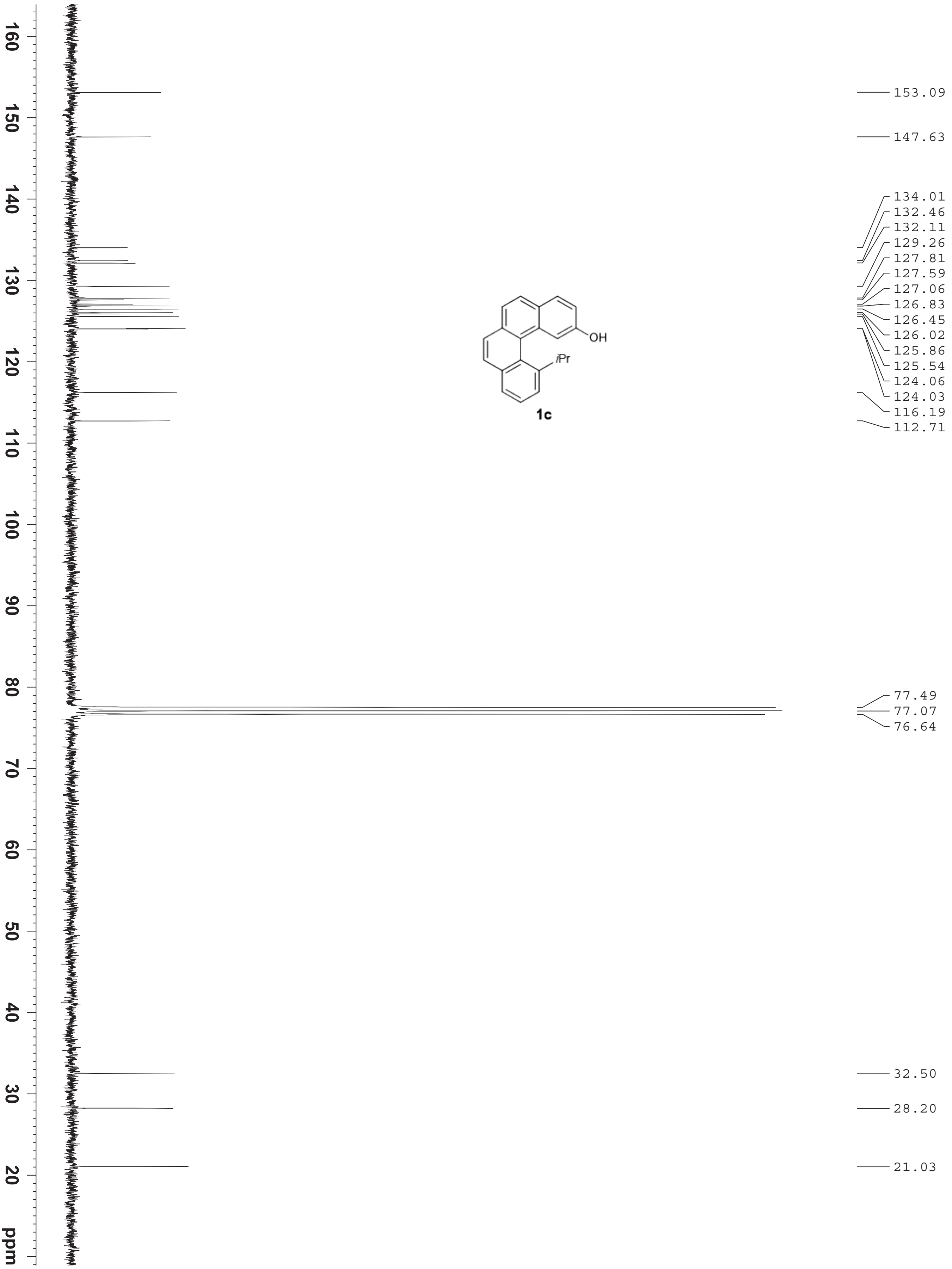

Supplementary Fig. 96. ^{13}C NMR of compound **1c** (75 MHz, $CDCl_3$)

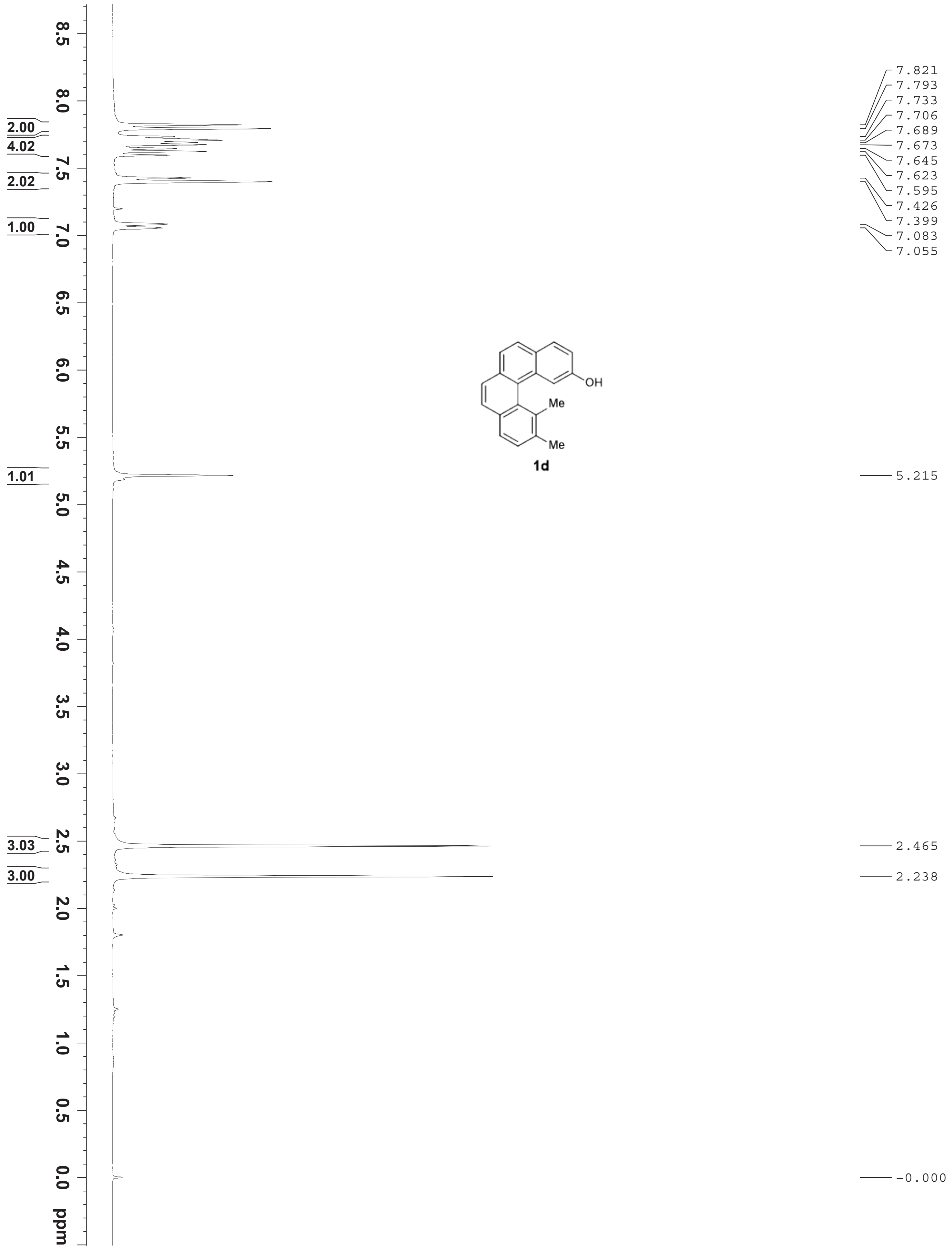

Supplementary Fig. 97. ^{1}H NMR of compound **1d** (300 MHz, $CDCl_3$)

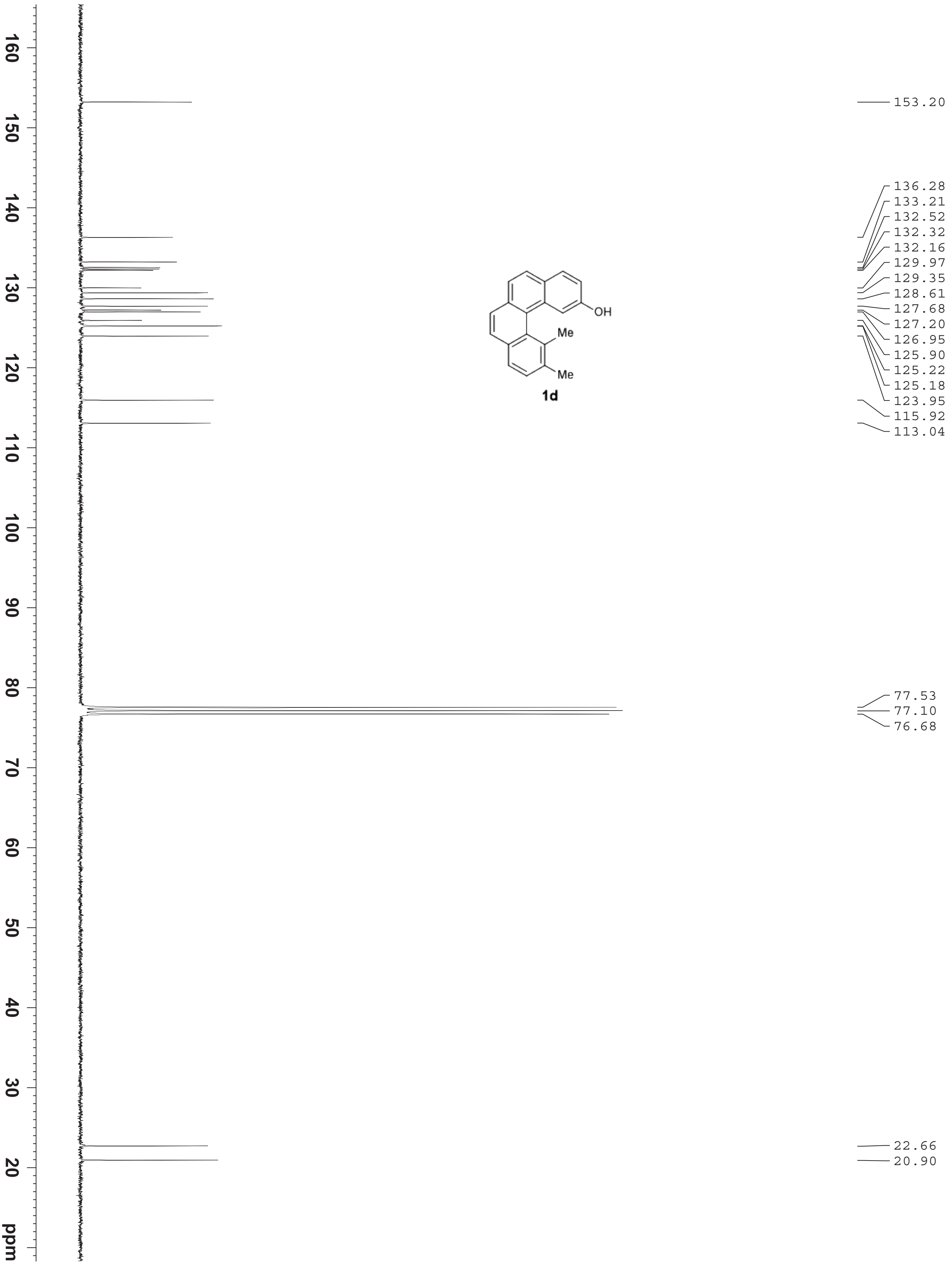

Supplementary Fig. 98. ^{13}C NMR of compound **1d** (75 MHz, $CDCl_3$)

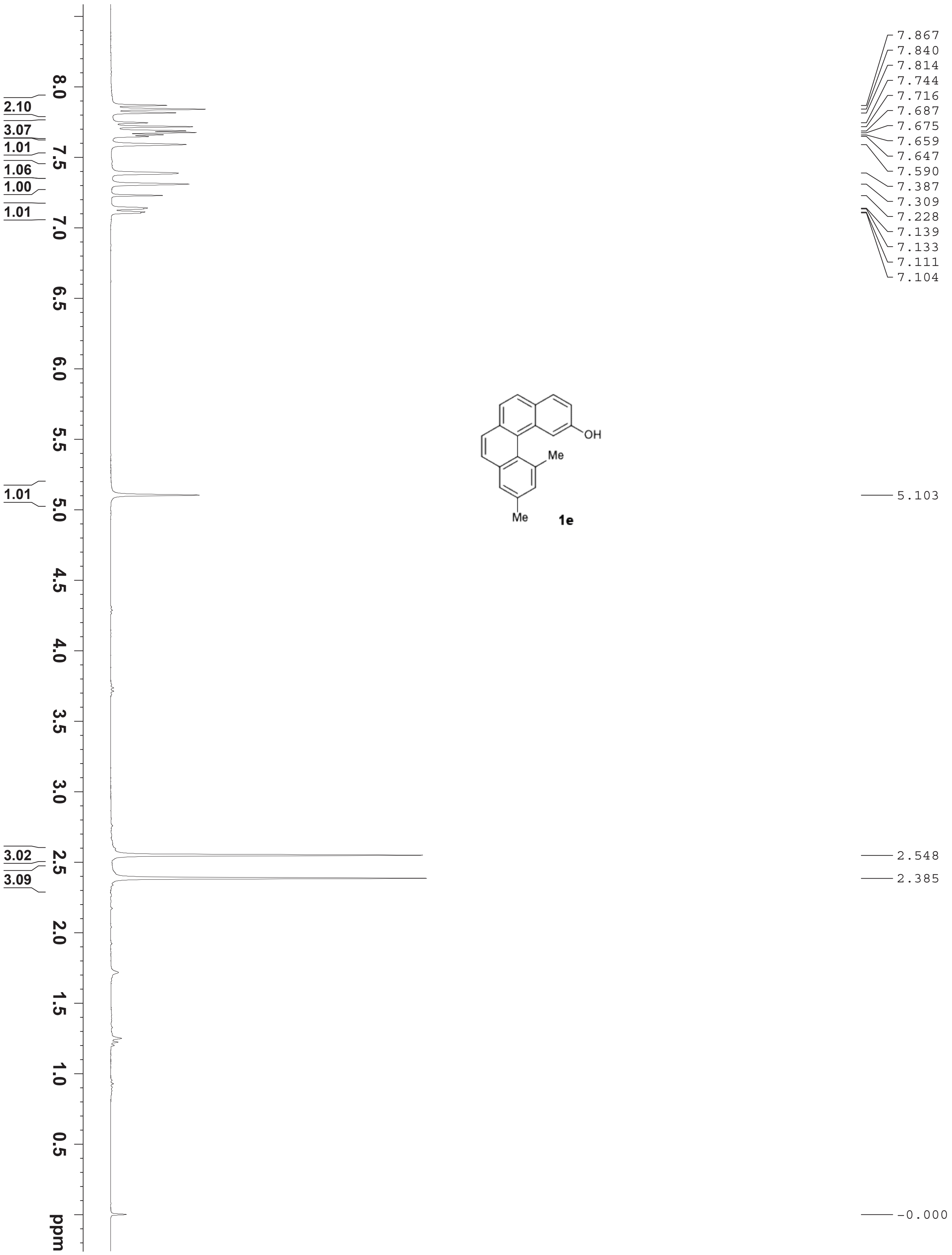

Supplementary Fig. 99. ^{1}H NMR of compound **1e** (300 MHz, $CDCl_3$)

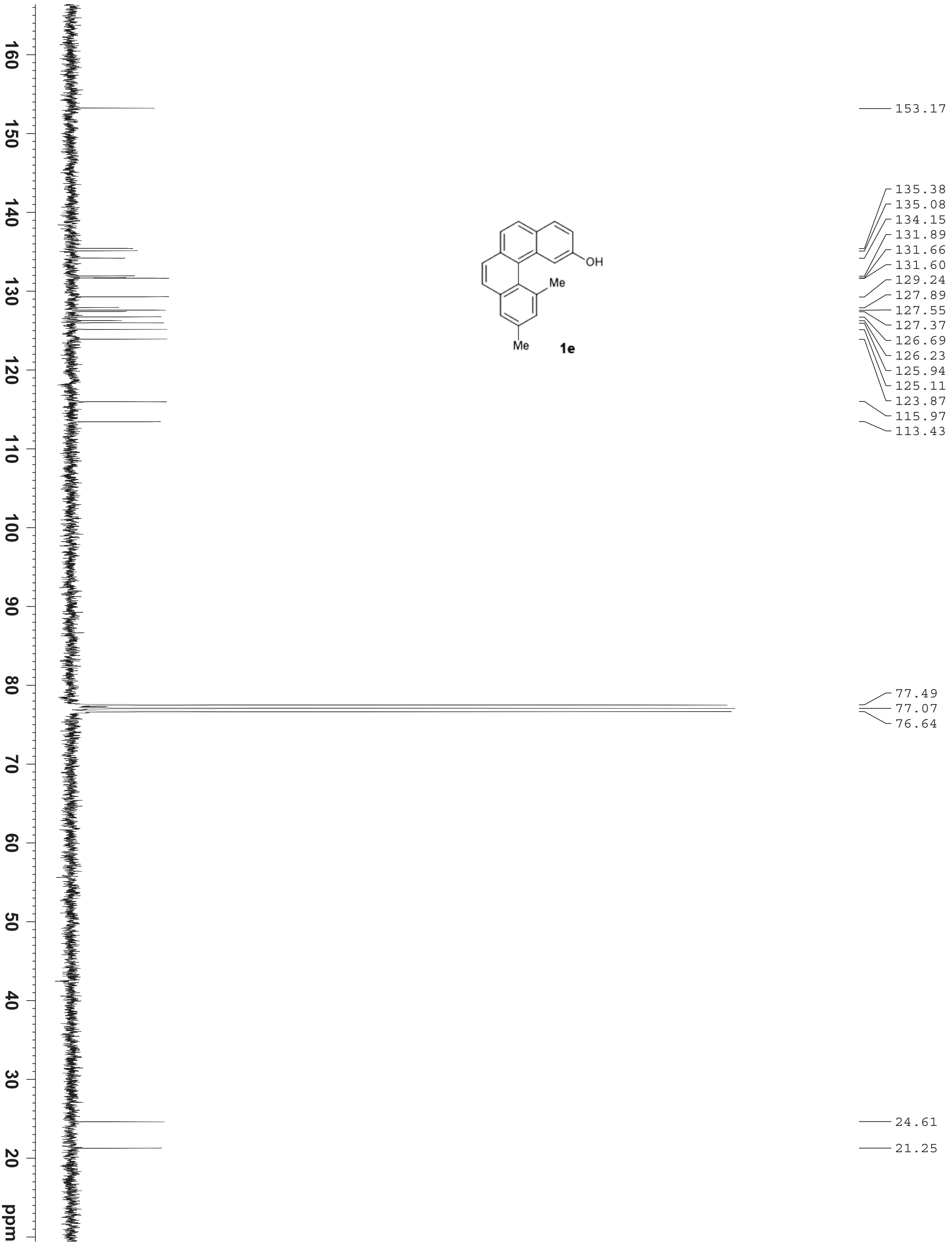

Supplementary Fig. 100. ^{13}C NMR of compound **1e** (75 MHz, $CDCl_3$)

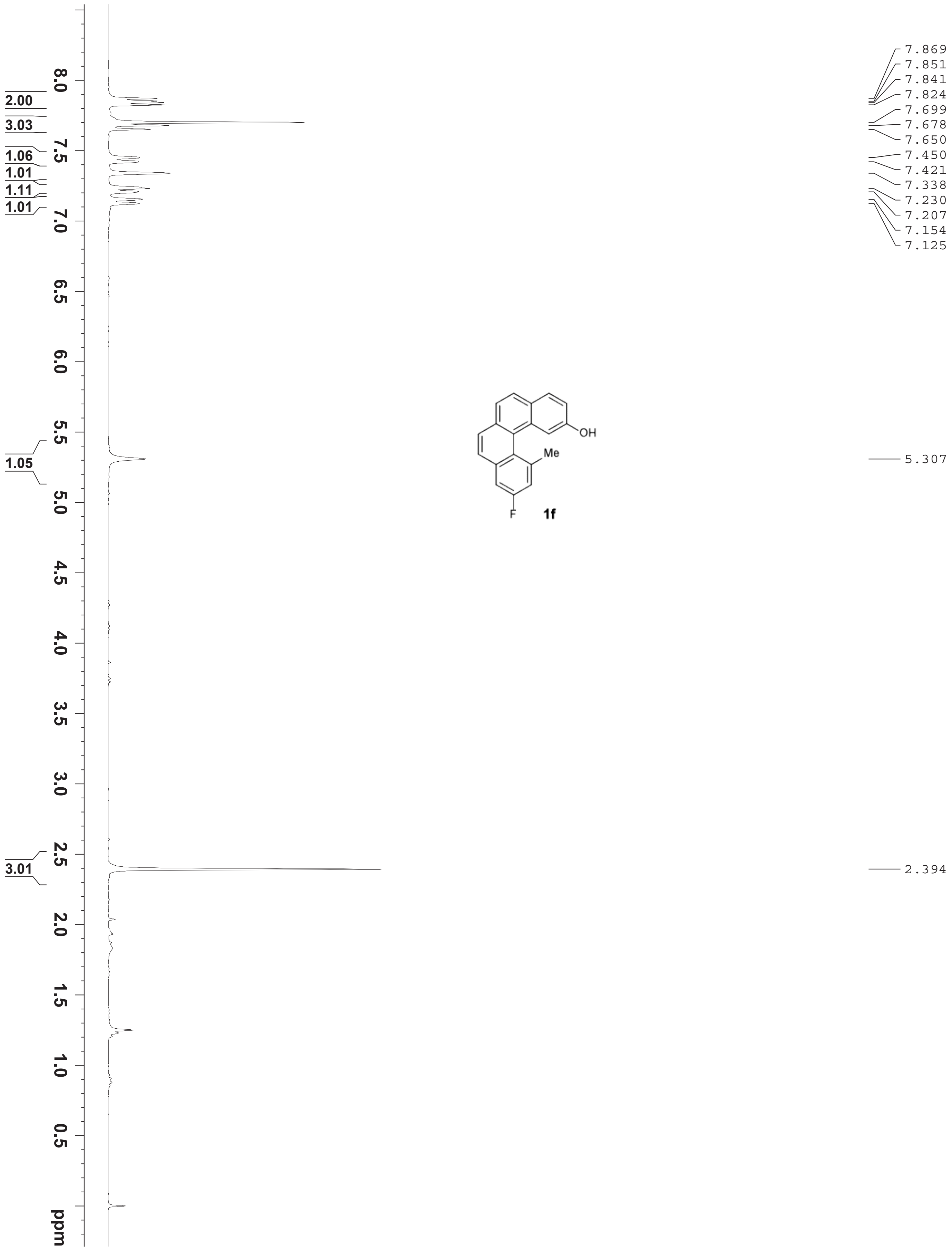

Supplementary Fig. 101. ^{1}H NMR of compound **1f** (300 MHz, $CDCl_3$)

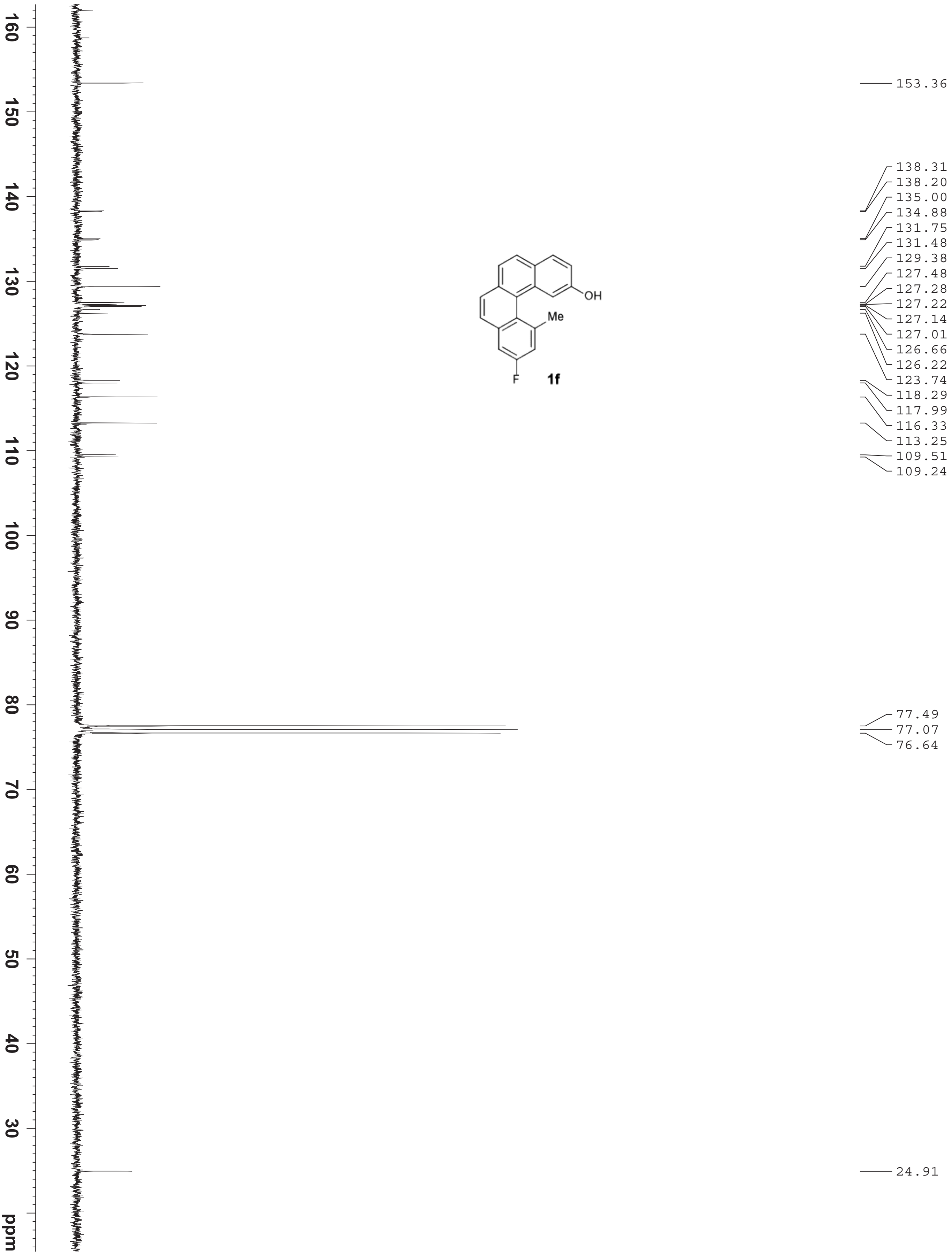

Supplementary Fig. 102. ^{13}C NMR of compound **1f** (75 MHz, $CDCl_3$)

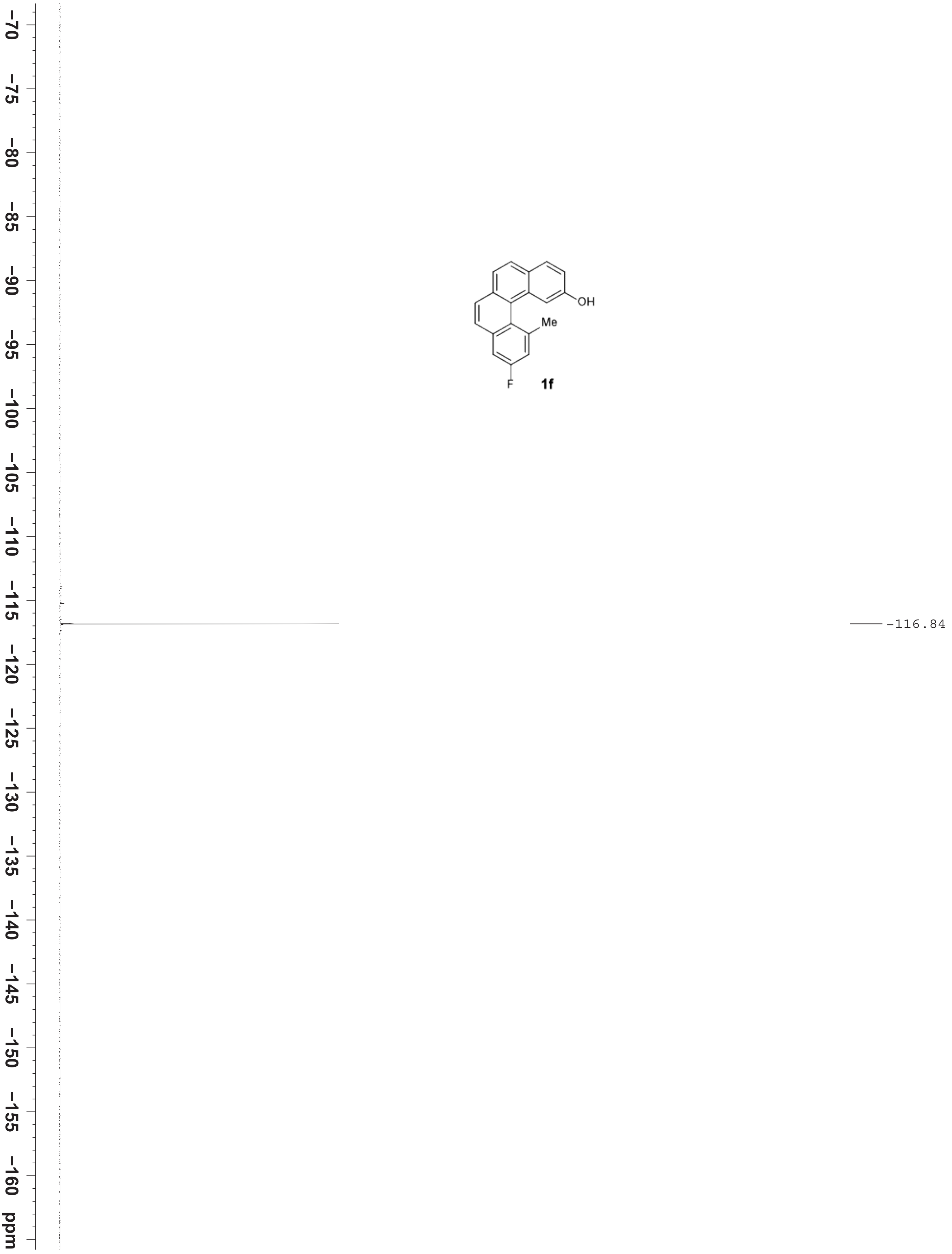

Supplementary Fig. 103. ^{19}F NMR of compound **1f** (282 MHz, $CDCl_3$)

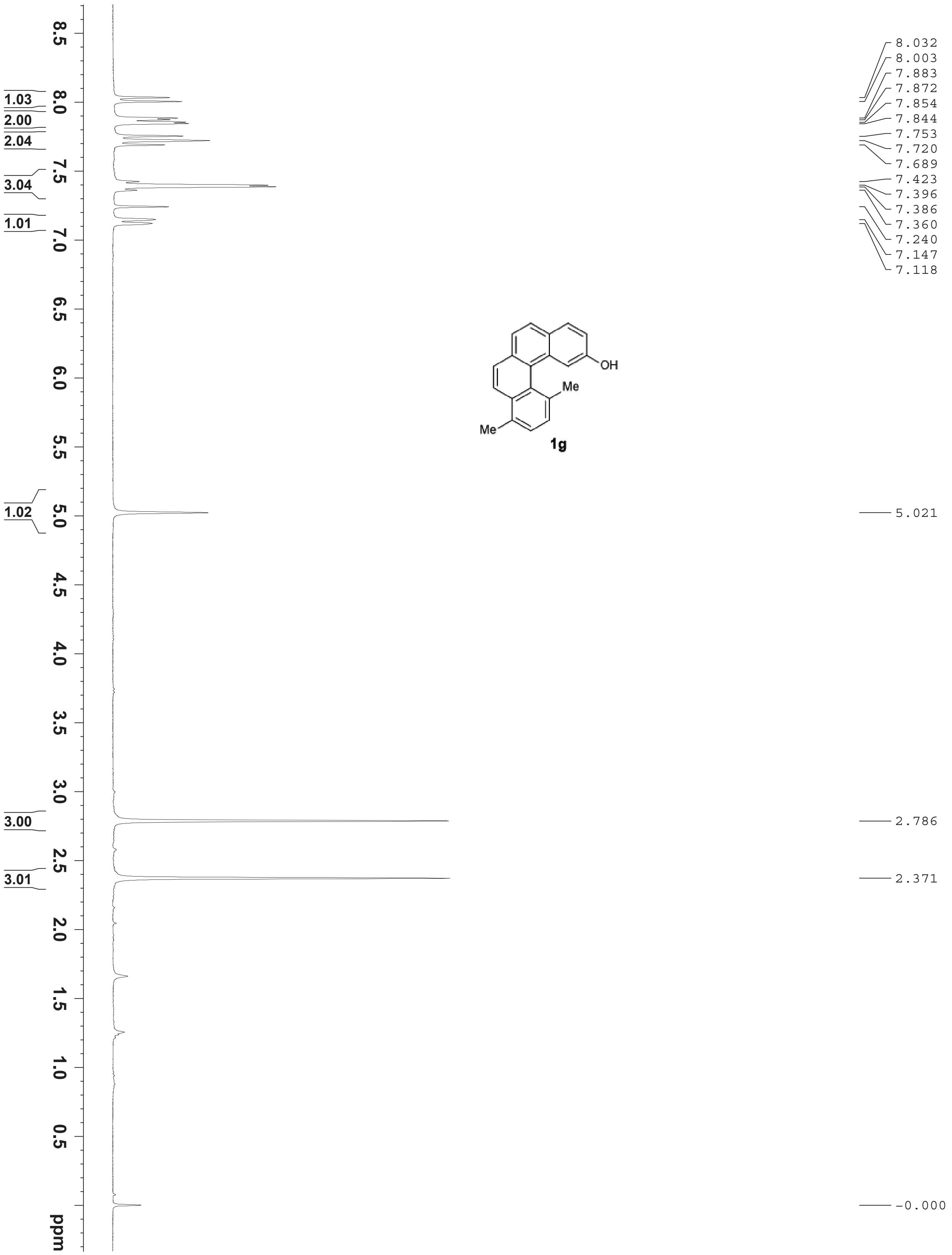

Supplementary Fig. 104. ^{1}H NMR of compound **1g** (300 MHz, $CDCl_3$)

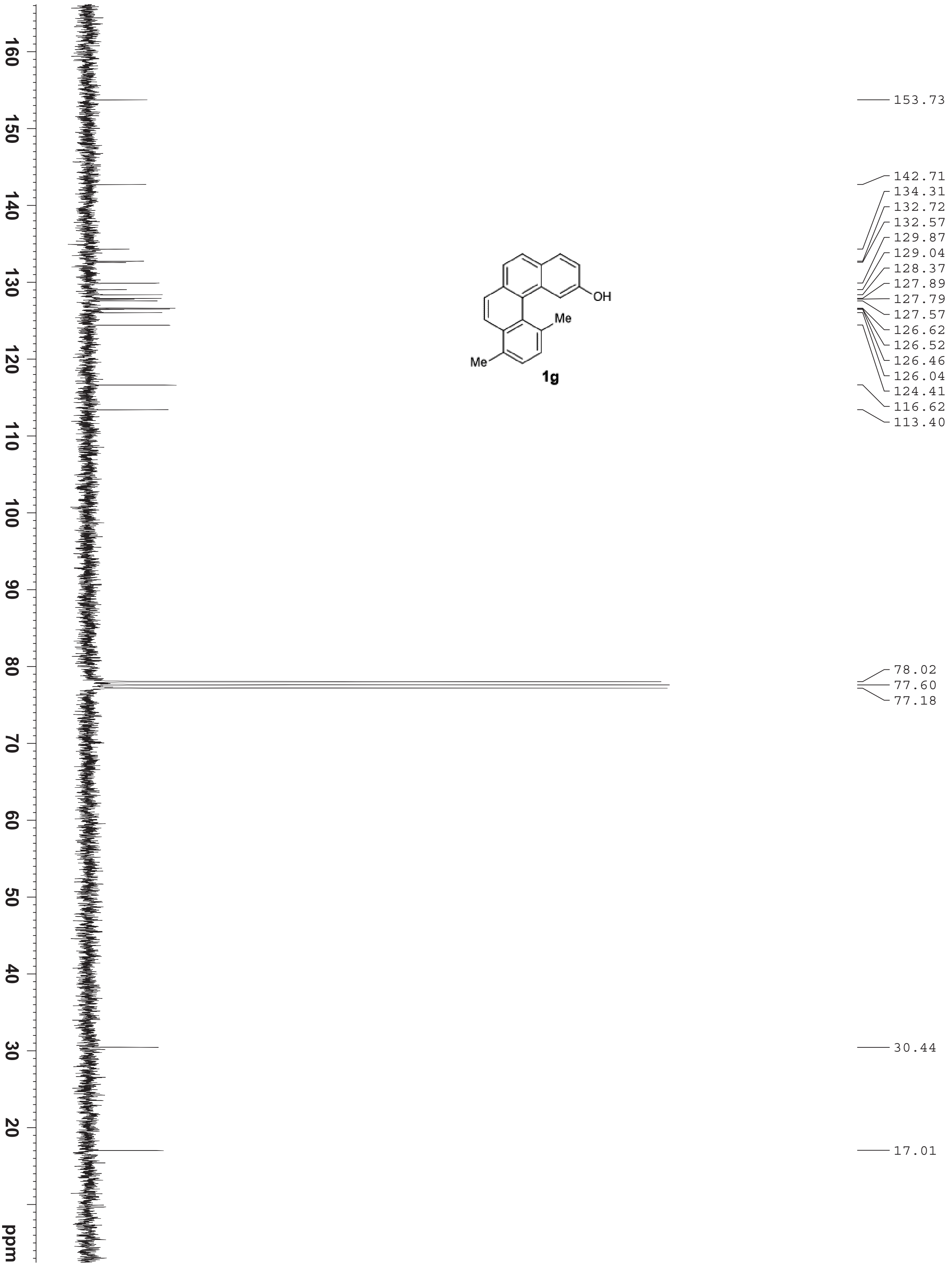

Supplementary Fig. 105. ^{13}C NMR of compound **1g** (75 MHz, $CDCl_3$)

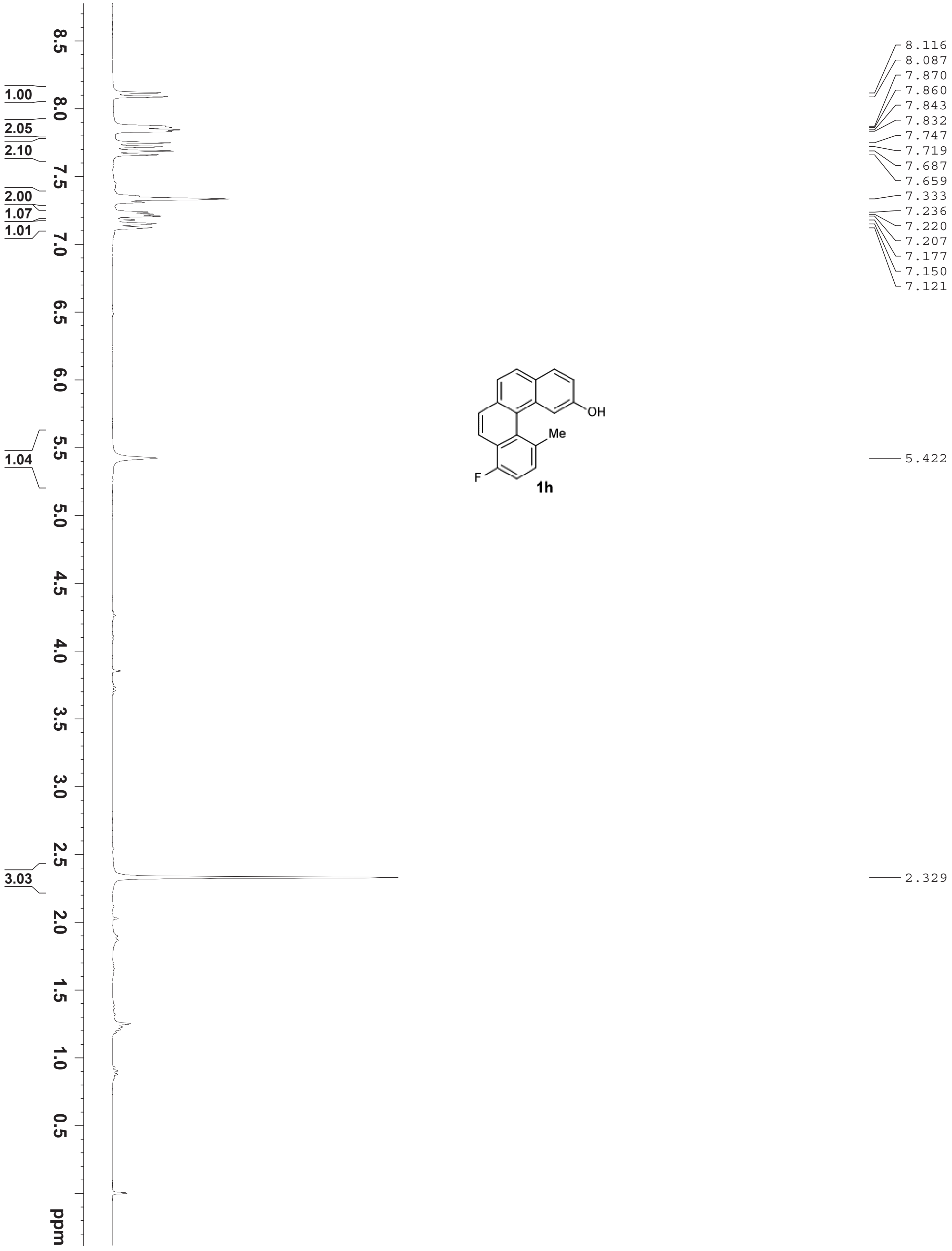

Supplementary Fig. 106. ^{1}H NMR of compound **1h** (300 MHz, $CDCl_3$)

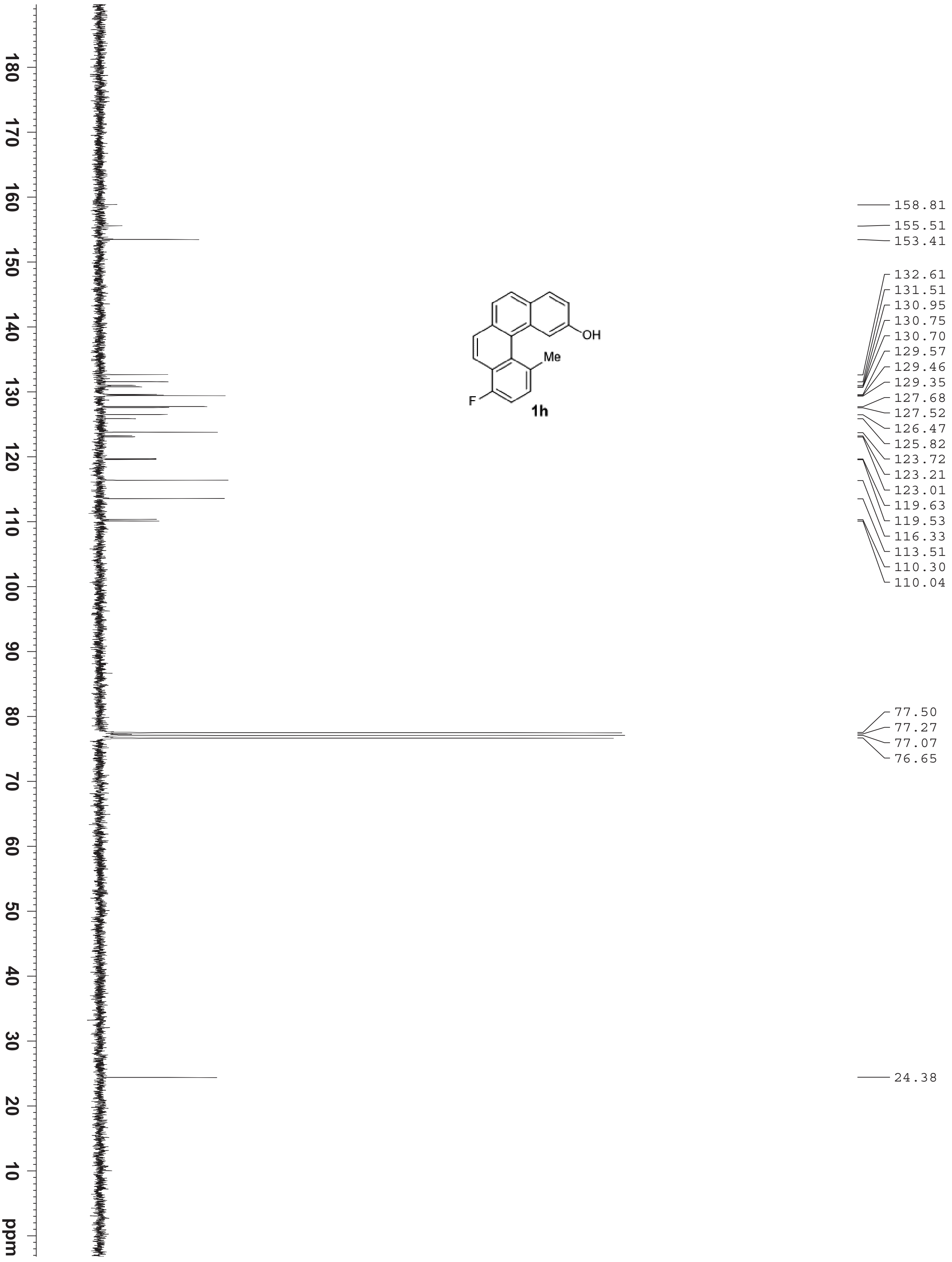

Supplementary Fig. 107. ^{13}C NMR of compound **1h** (75 MHz, $CDCl_3$)

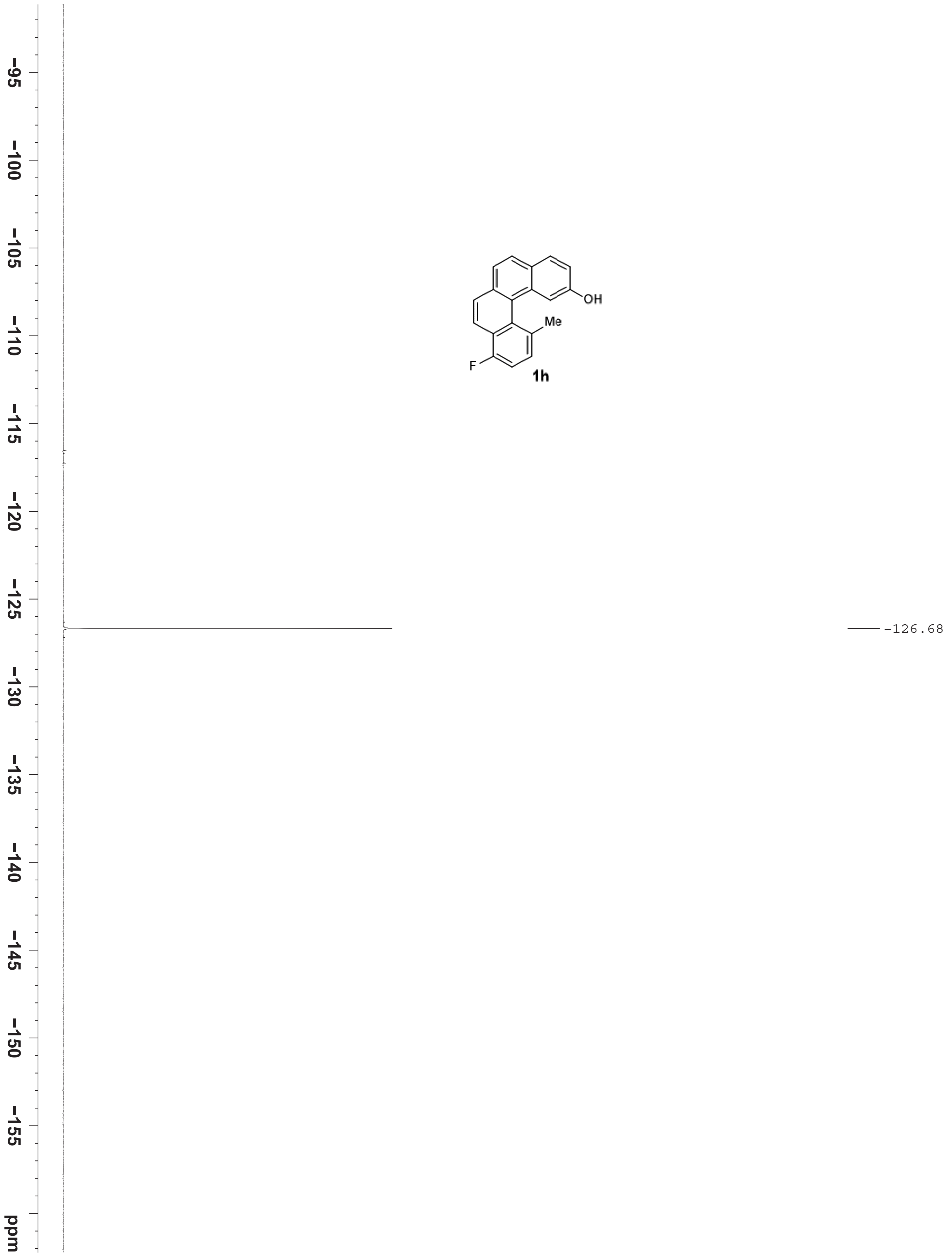

Supplementary Fig. 108. ^{19}F NMR of compound **1h** (282 MHz, $CDCl_3$)

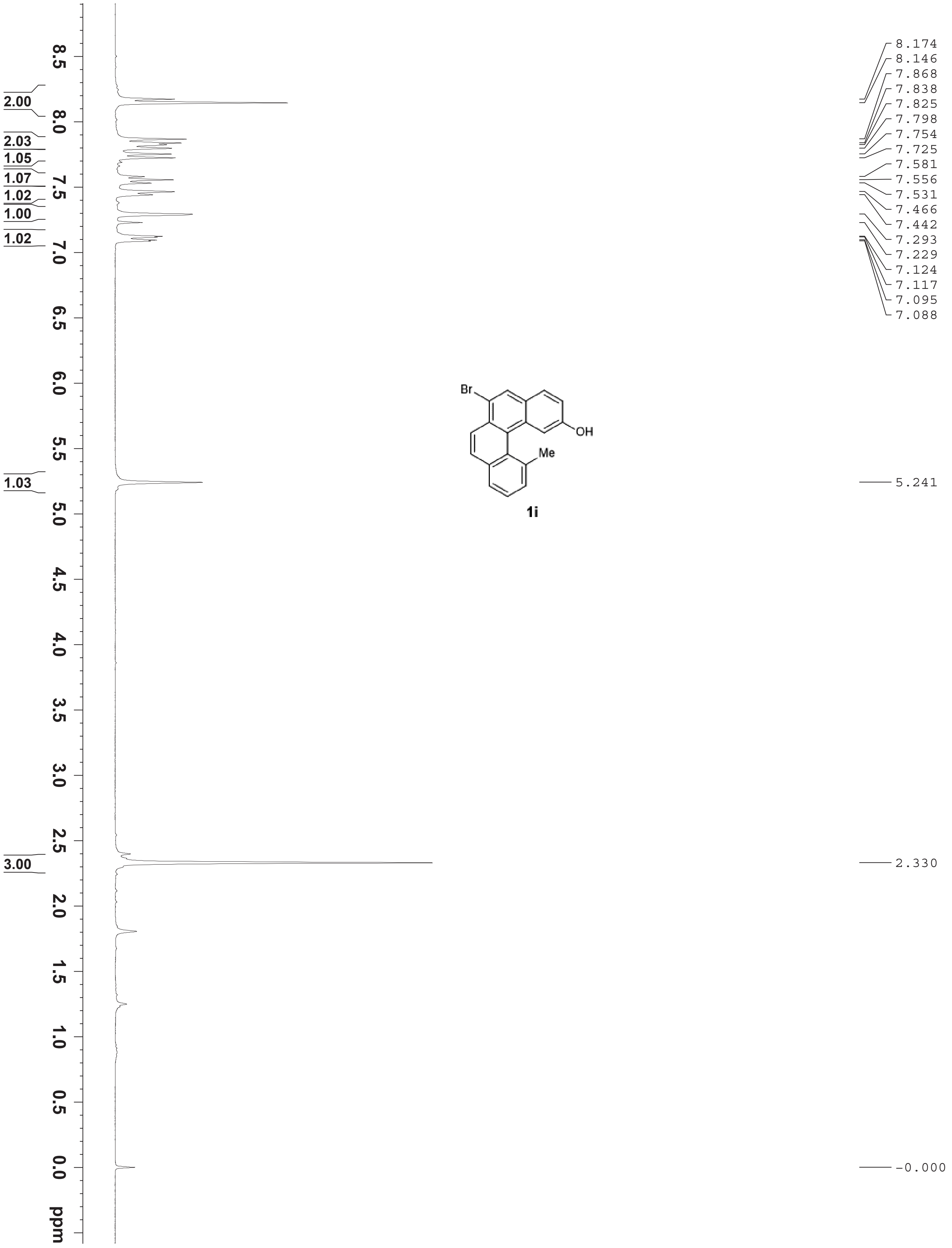

Supplementary Fig. 109. ^{1}H NMR of compound **1i** (300 MHz, $CDCl_3$)

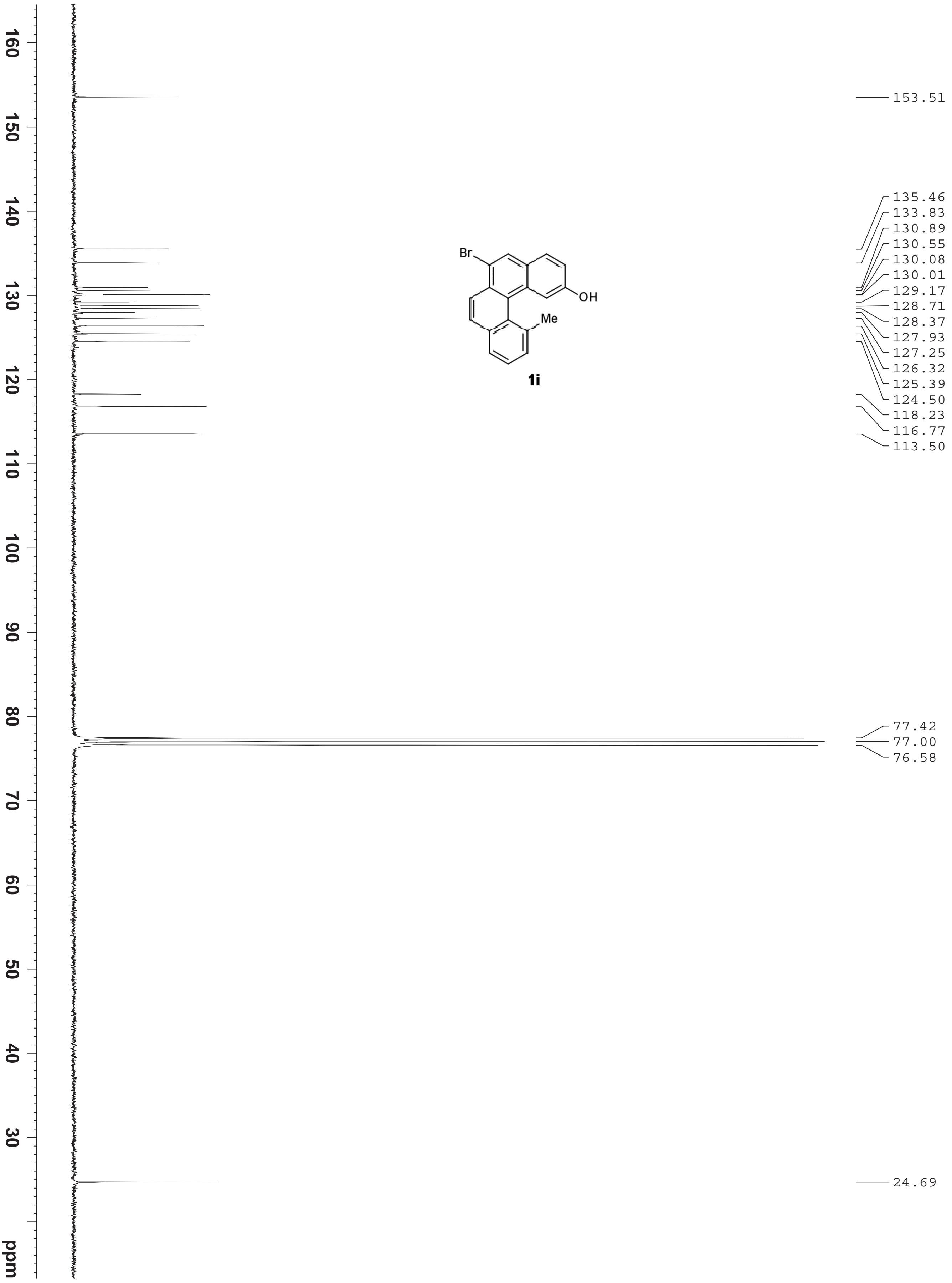

Supplementary Fig. 110. ^{13}C NMR of compound **1i** (75 MHz, $CDCl_3$)

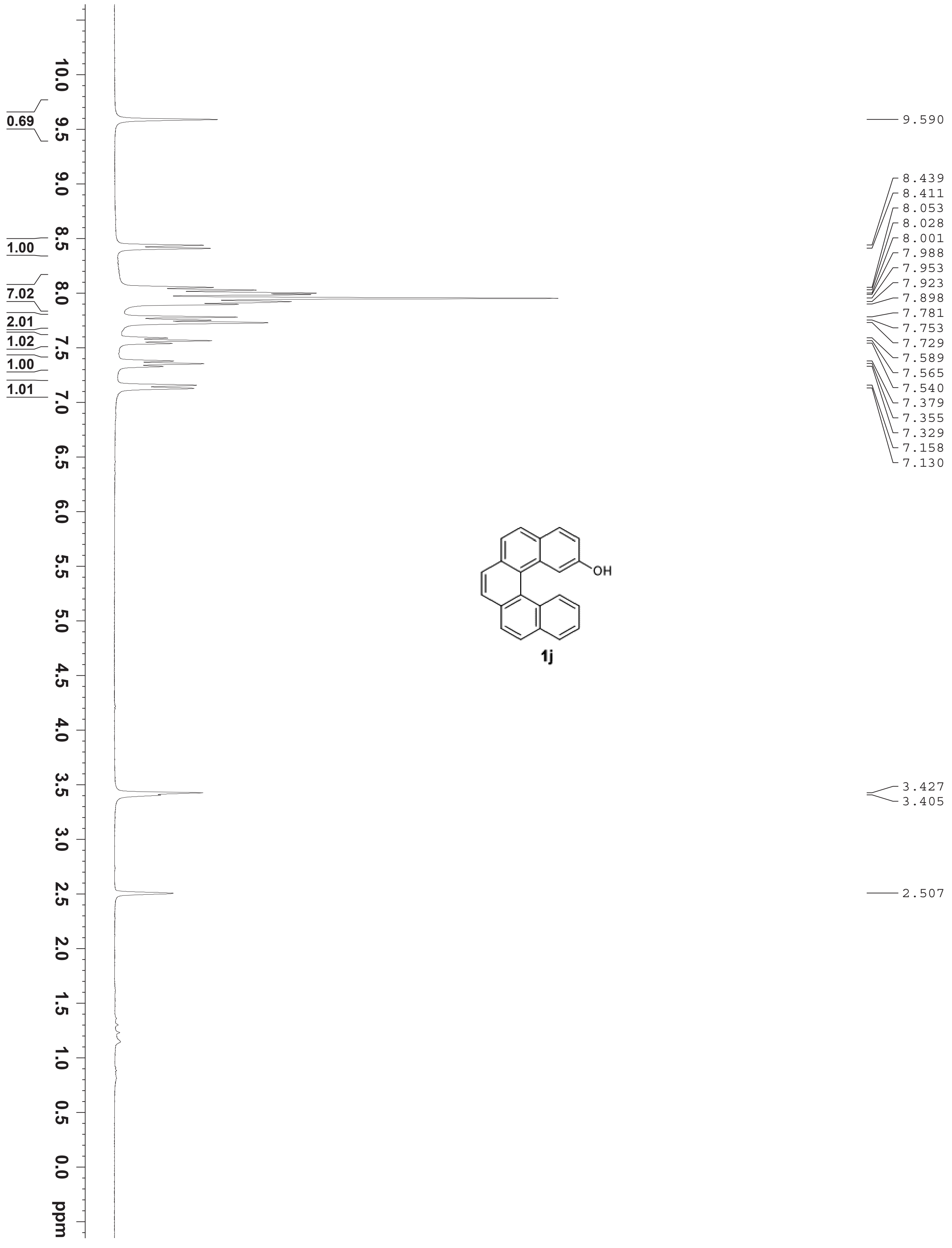

Supplementary Fig. 111. ^{1}H NMR of compound **1j** (300 MHz, DMSO-d_6)

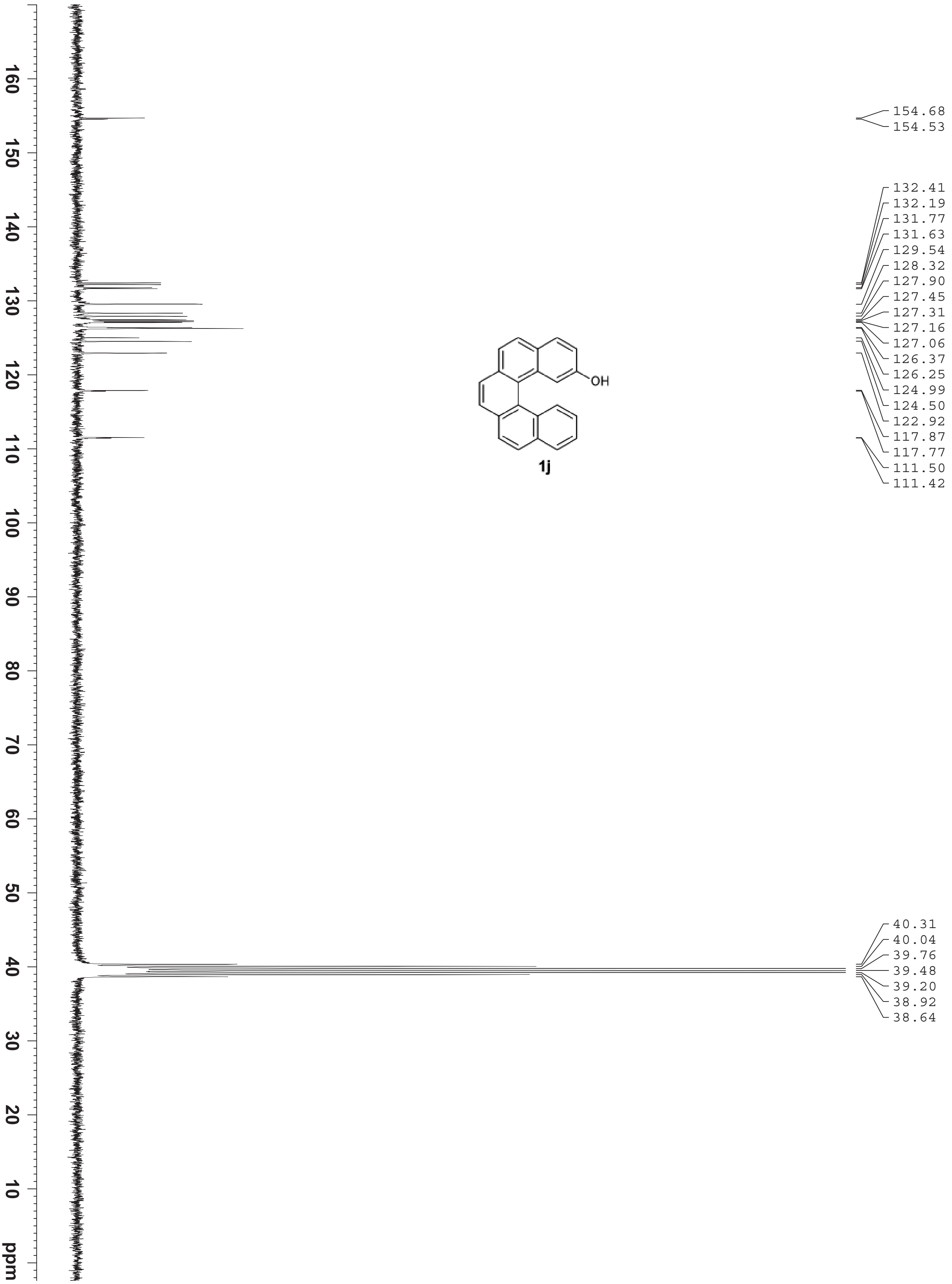

Supplementary Fig. 112. ^{13}C NMR of compound **1j** (75 MHz, DMSO-d_6)

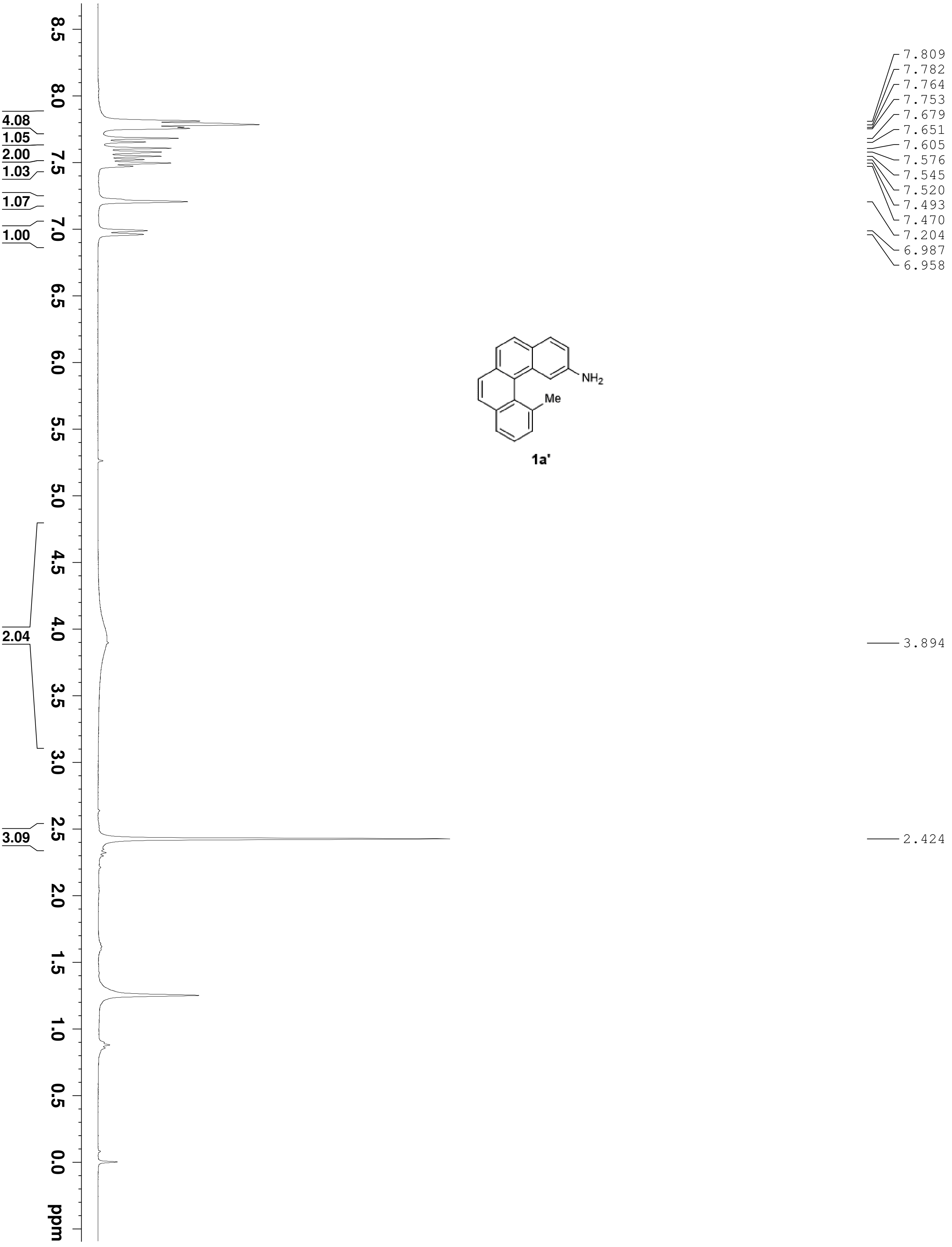

Supplementary Fig. 113. ^{1}H NMR of compound **1a'** (300 MHz, $CDCl_3$)

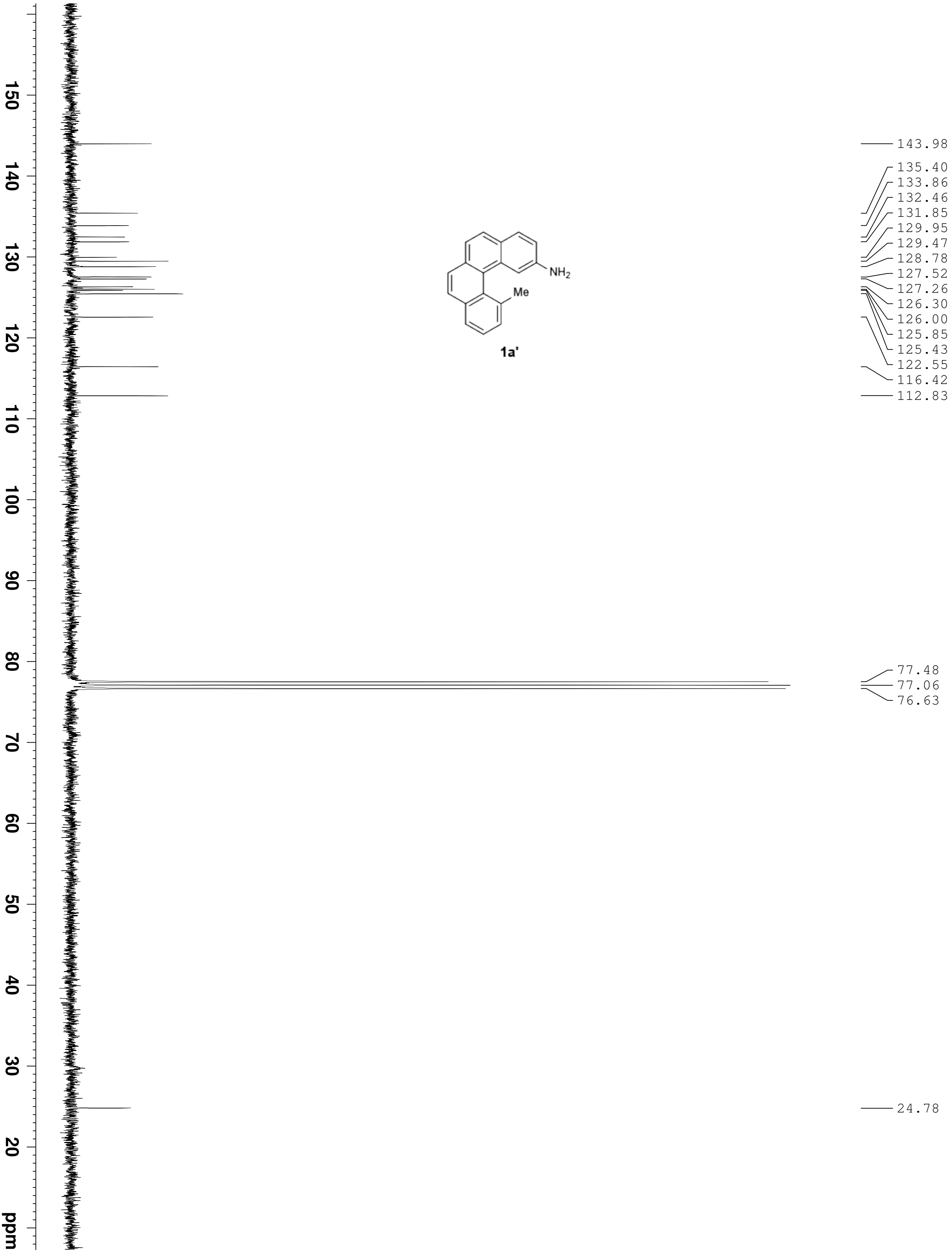

Supplementary Fig. 114. ^{13}C NMR of compound **1a'** (75 MHz, $CDCl_3$)

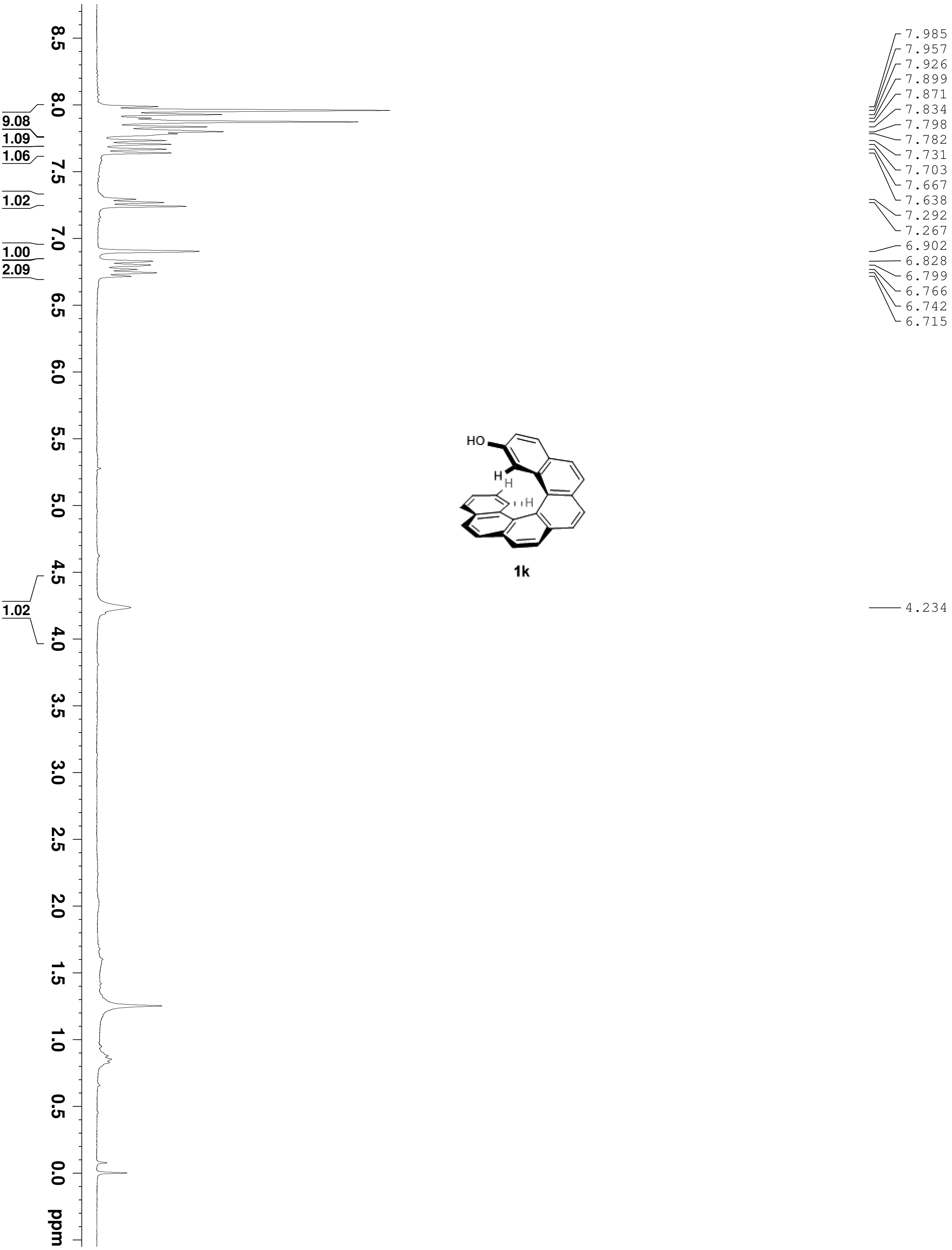

Supplementary Fig. 115. ^{1}H NMR of compound **1k** (300 MHz, $CDCl_3$)

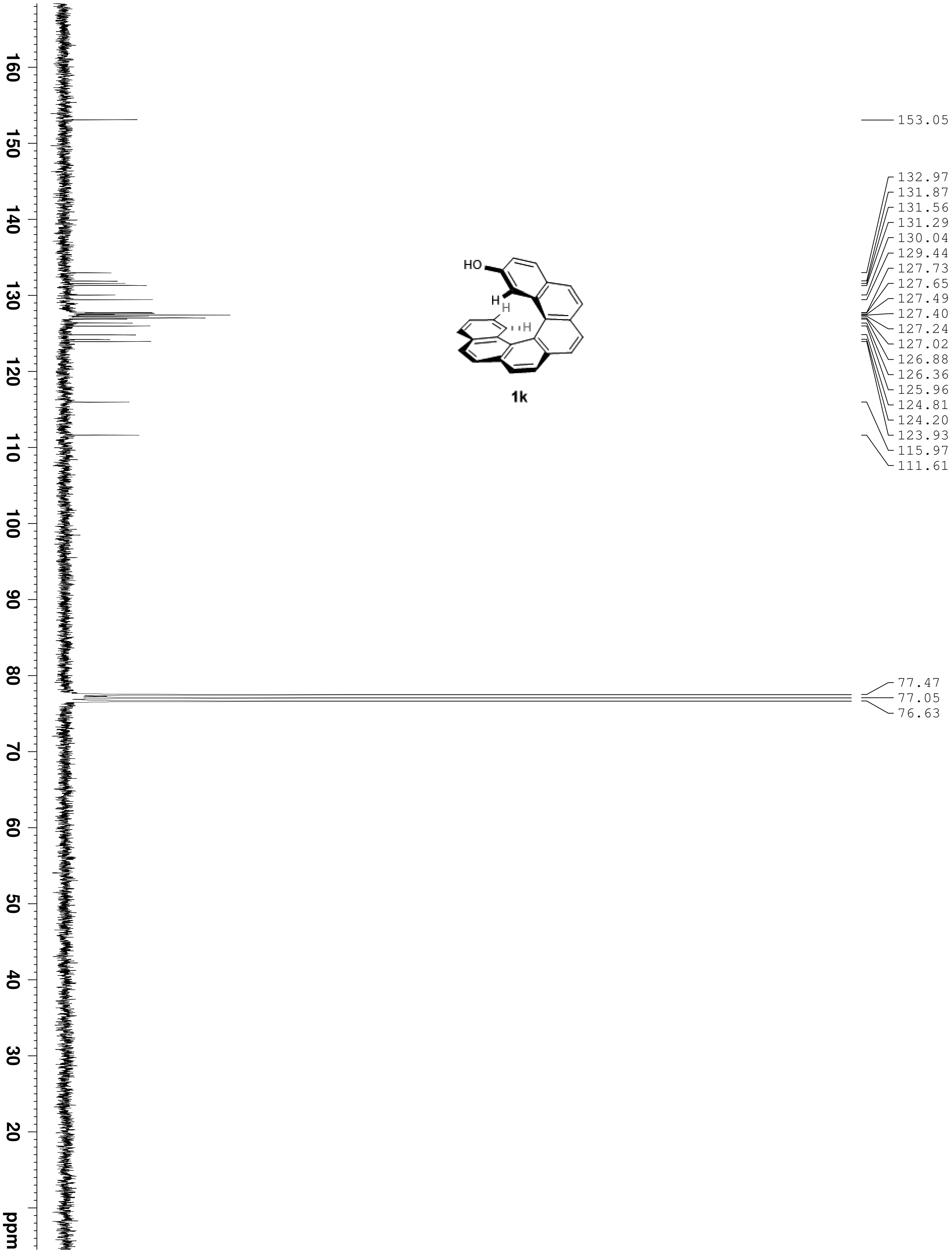

Supplementary Fig. 116. ^{13}C NMR of compound **1k** (75 MHz, $CDCl_3$)

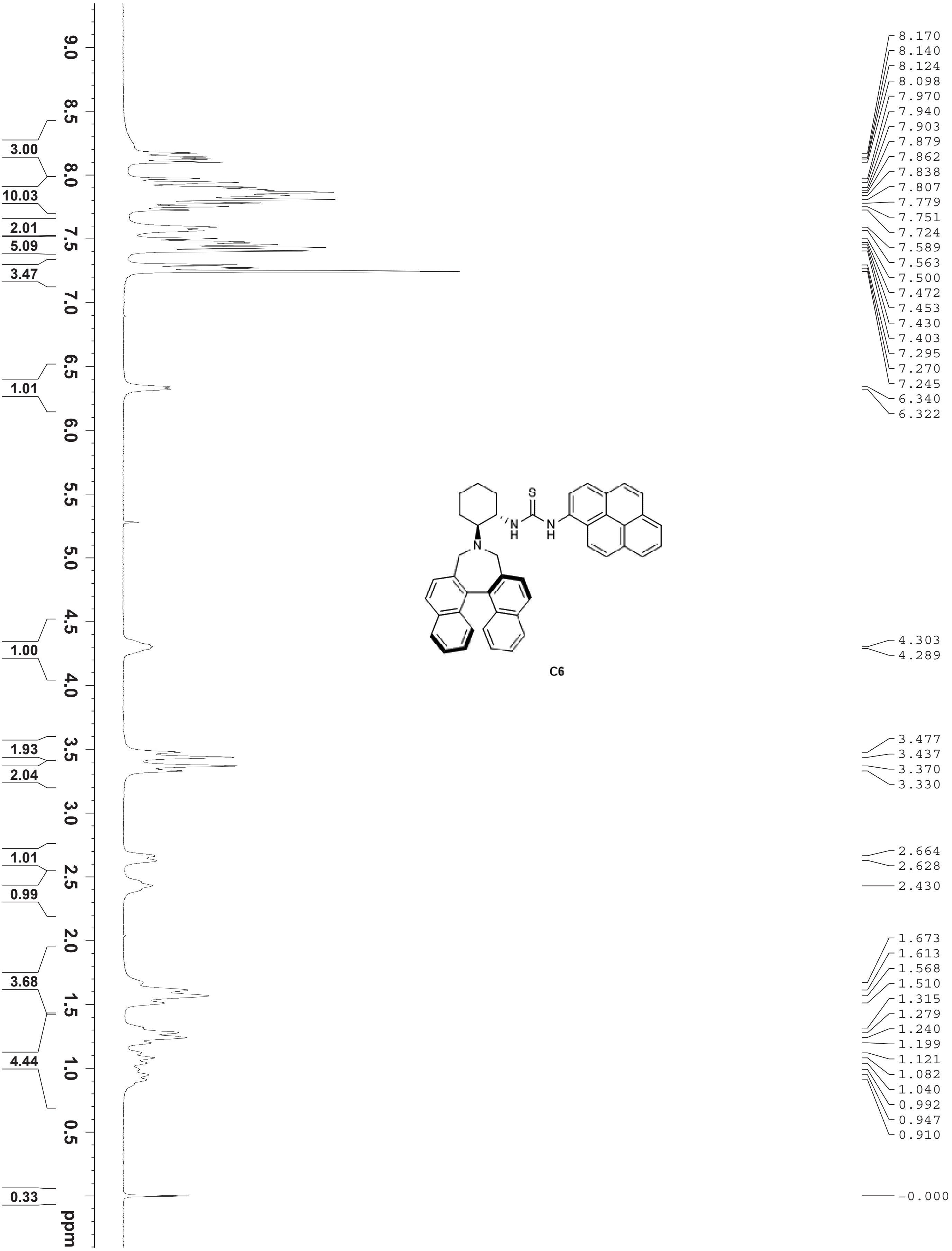

Supplementary Fig. 117. ^{1}H NMR of compound **C6** (300 MHz, $CDCl_3$)

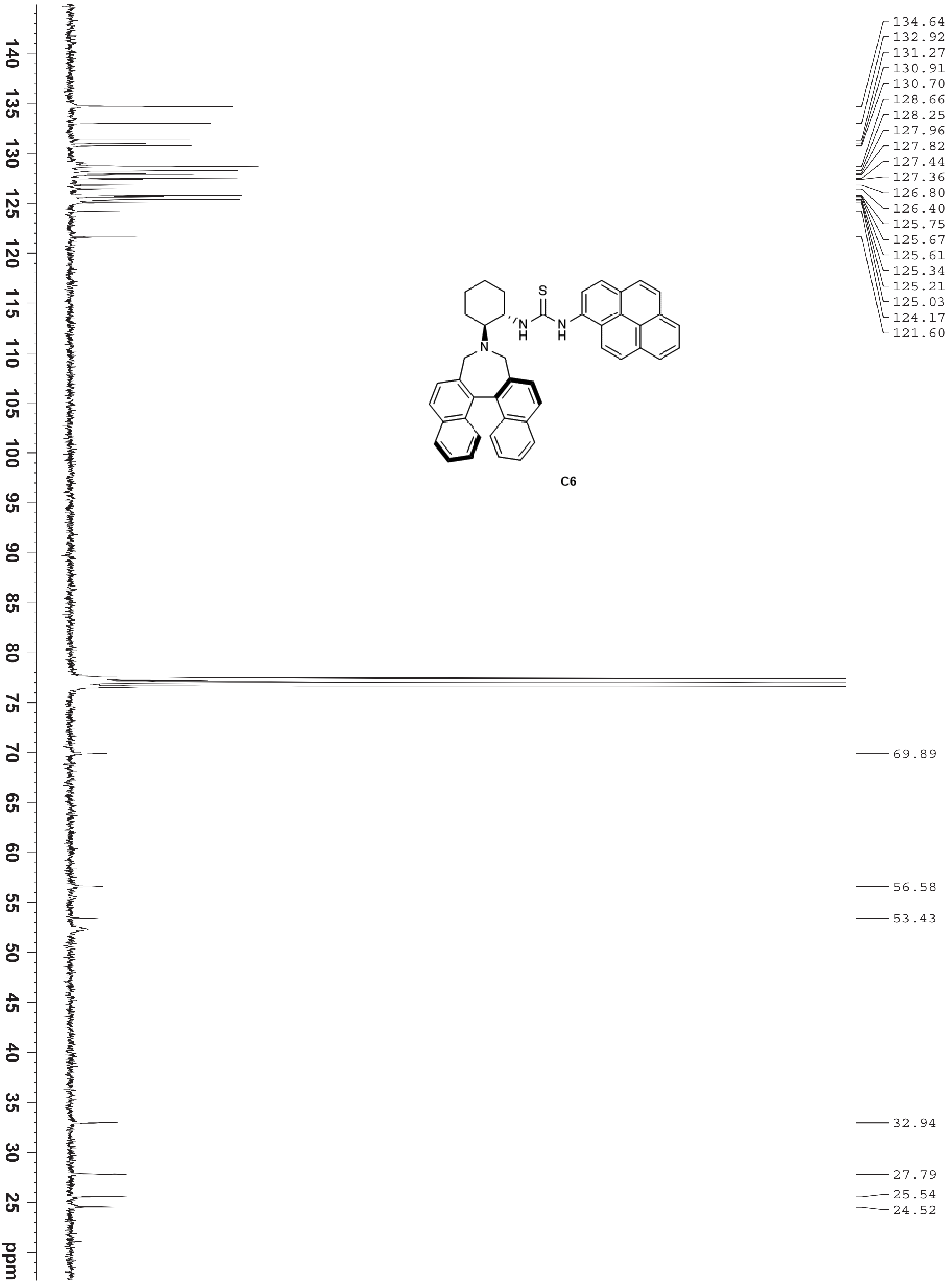

Supplementary Fig. 118. ^{13}C NMR of compound **C6** (75 MHz, $CDCl_3$)

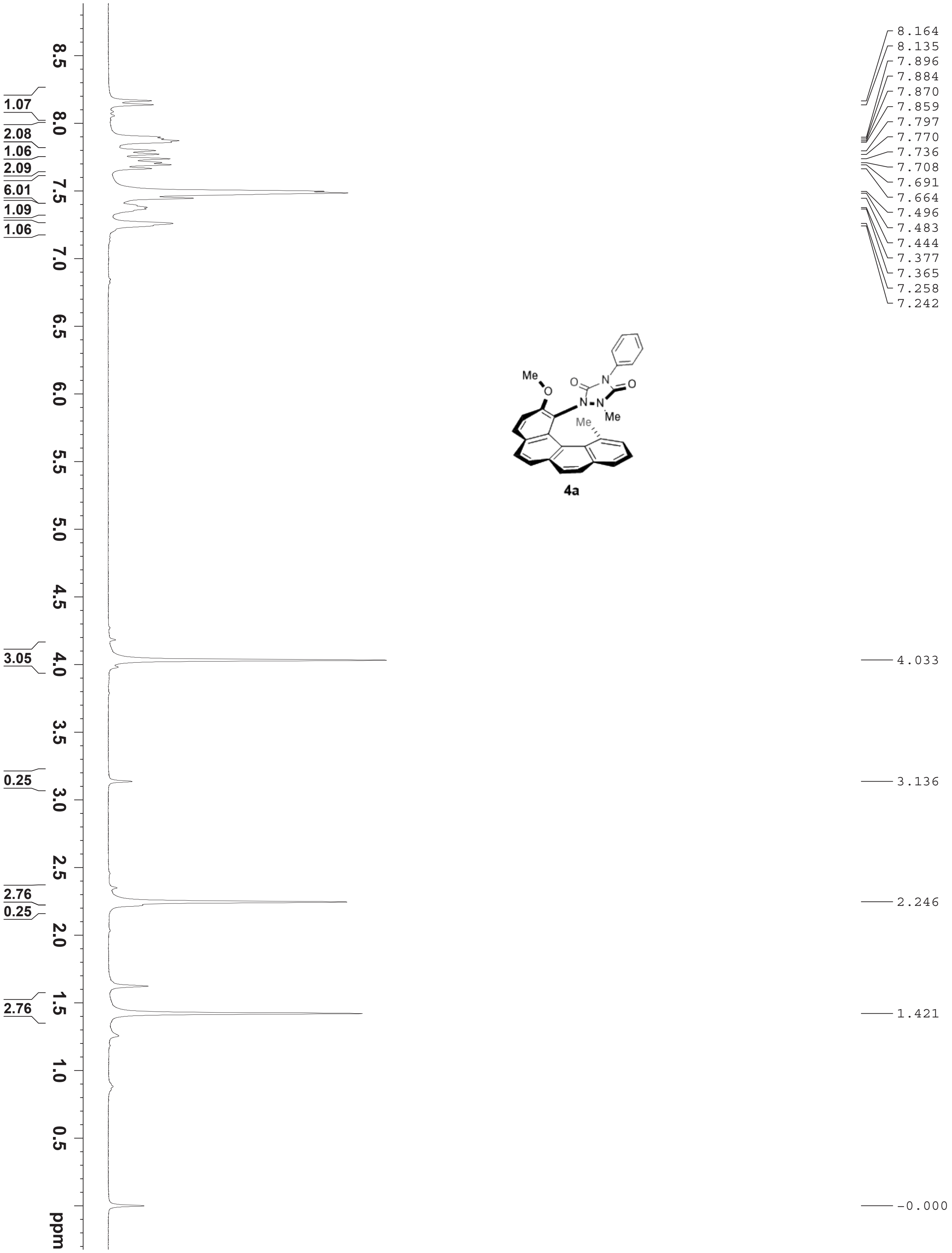

Supplementary Fig. 119. ^{1}H NMR of compound **4a** (300 MHz, $CDCl_3$)

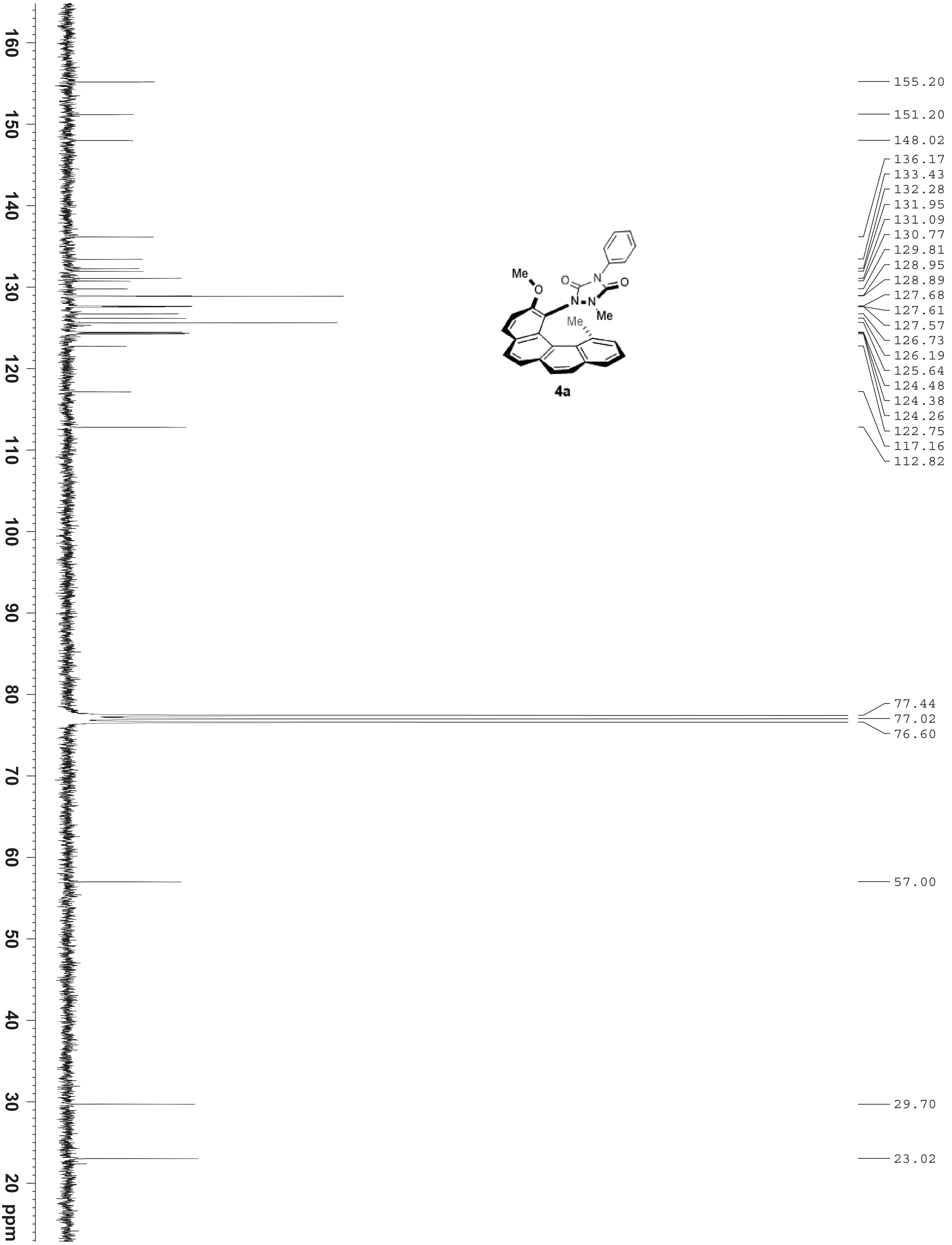

Supplementary Fig. 120. ^{13}C NMR of compound **4a** (75 MHz, $CDCl_3$)

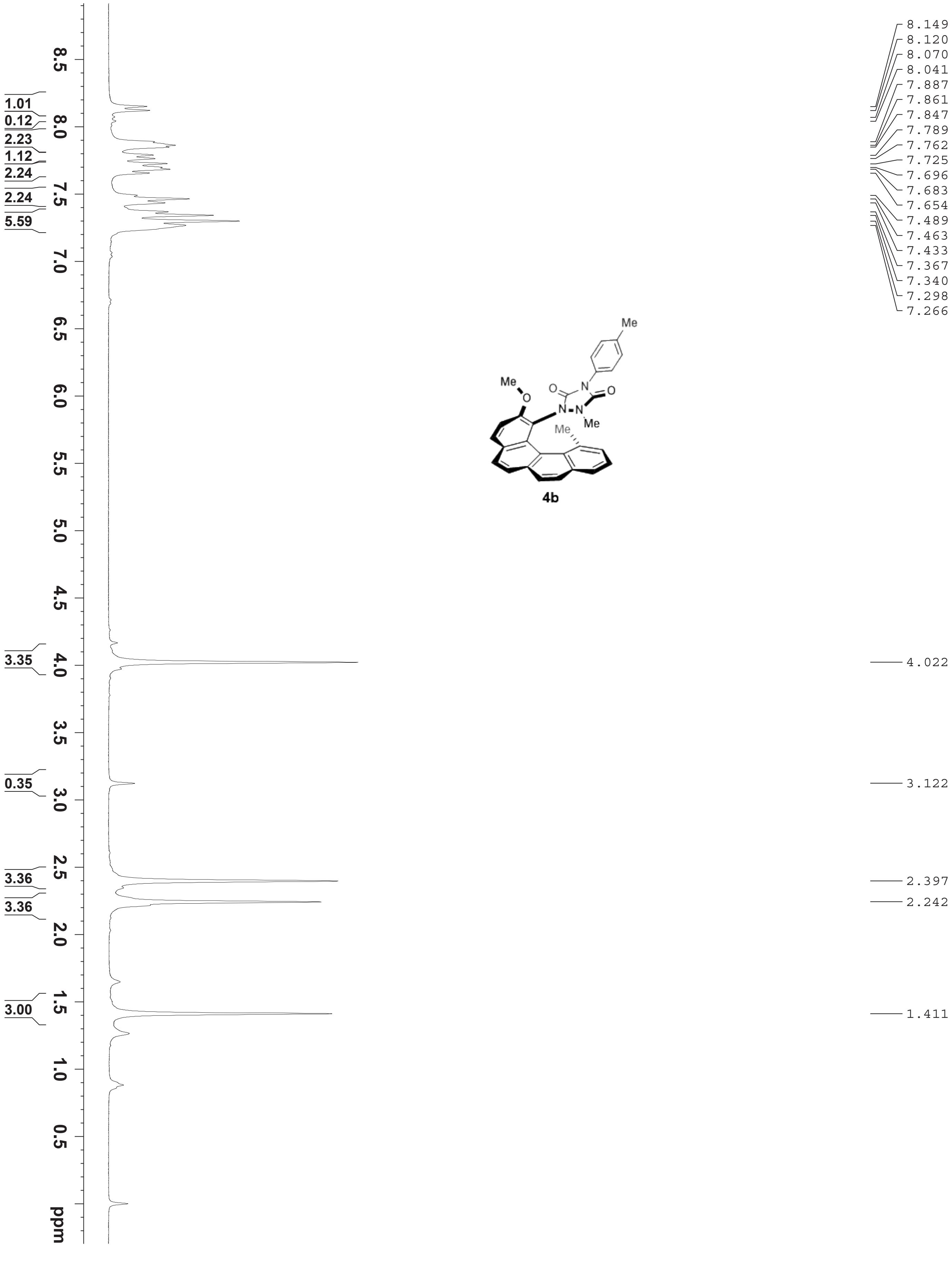

Supplementary Fig. 121. ^{1}H NMR of compound **4b** (300 MHz, $CDCl_3$)

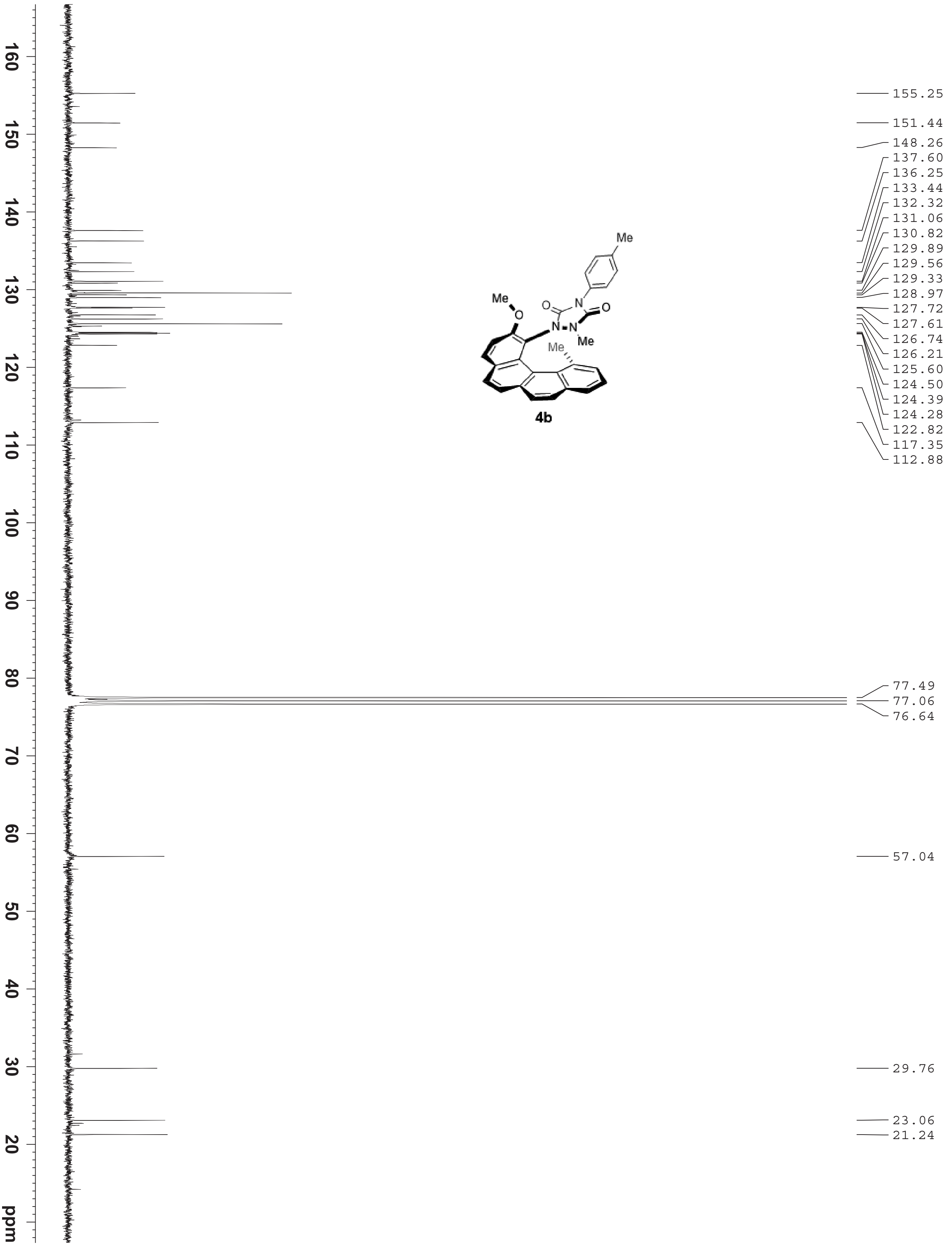

Supplementary Fig. 122. ^{13}C NMR of compound **4b** (75 MHz, $CDCl_3$)

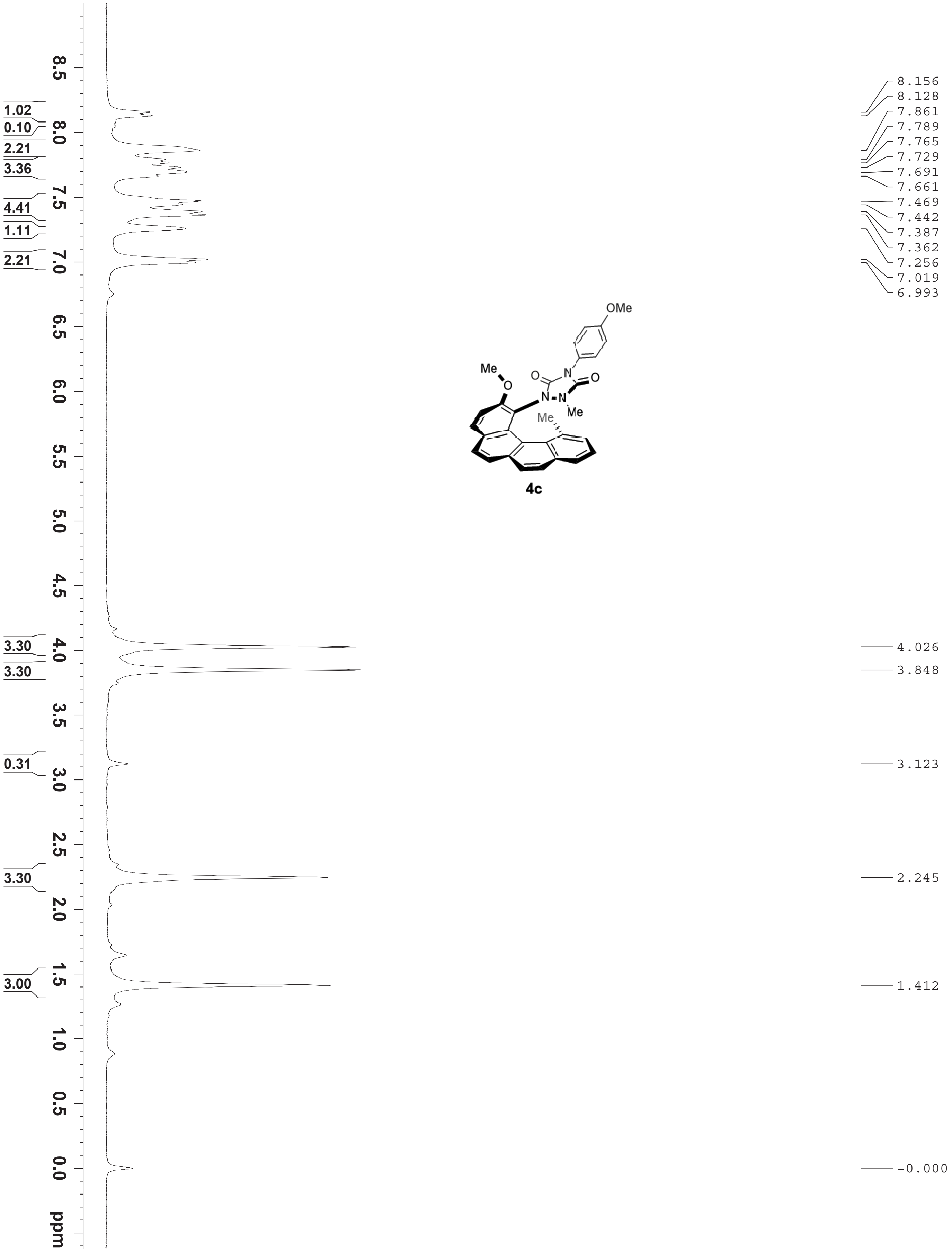

Supplementary Fig. 123. ^{1}H NMR of compound **4c** (300 MHz, $CDCl_3$)

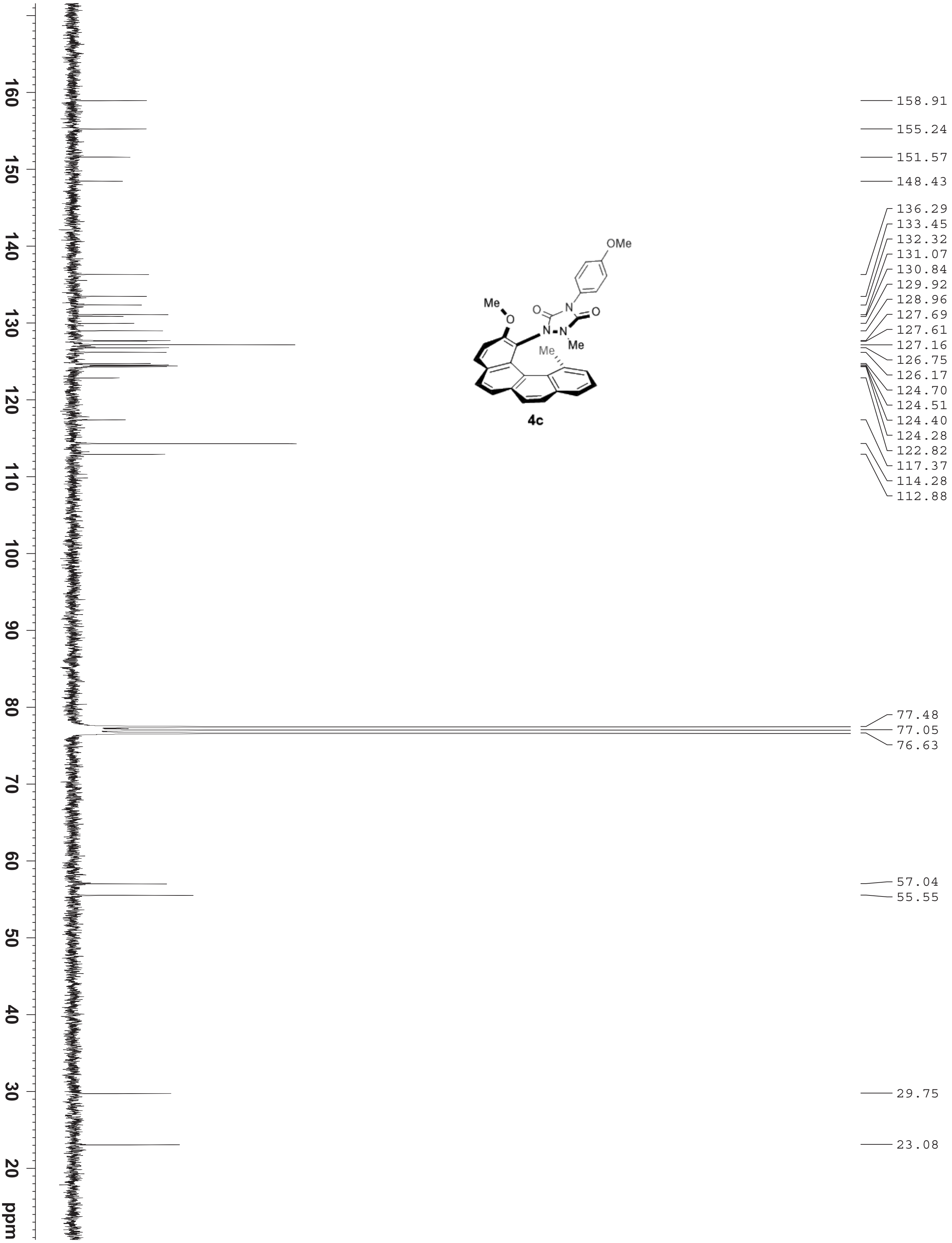

Supplementary Fig. 124. ^{13}C NMR of compound **4c** (75 MHz, $CDCl_3$)

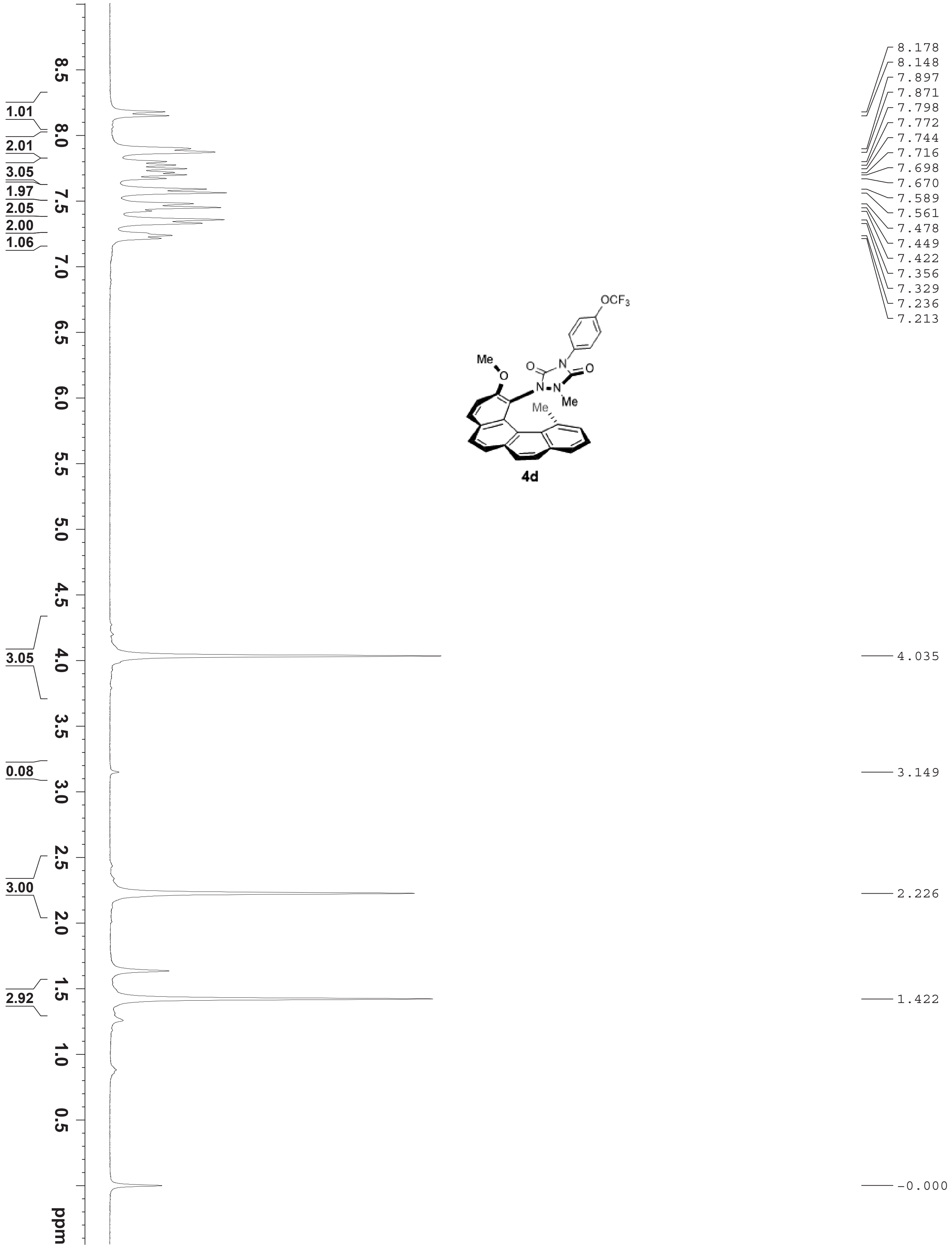

Supplementary Fig. 125. ^{1}H NMR of compound **4d** (300 MHz, $CDCl_3$)

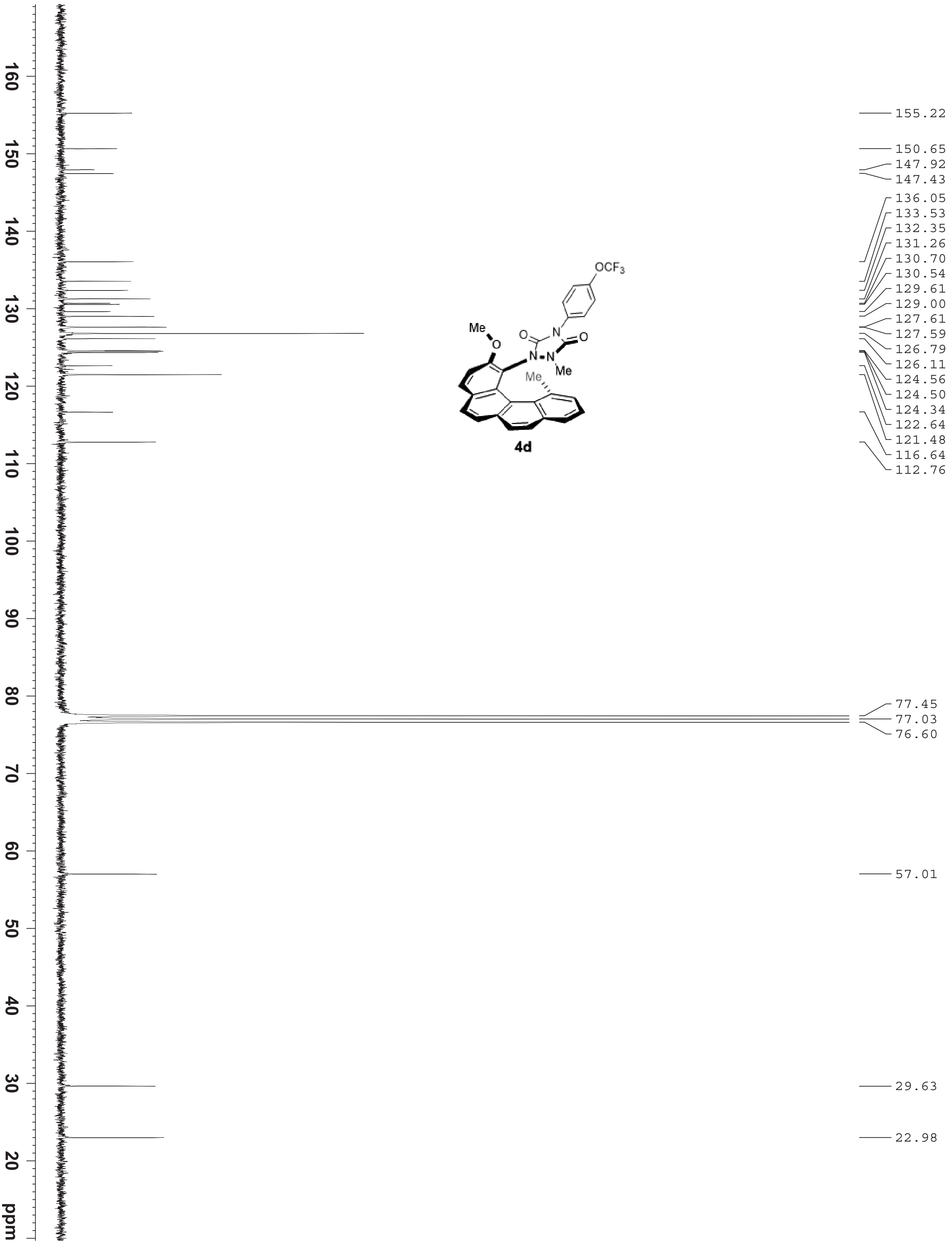

Supplementary Fig. 126. ^{13}C NMR of compound **4d** (75 MHz, $CDCl_3$)

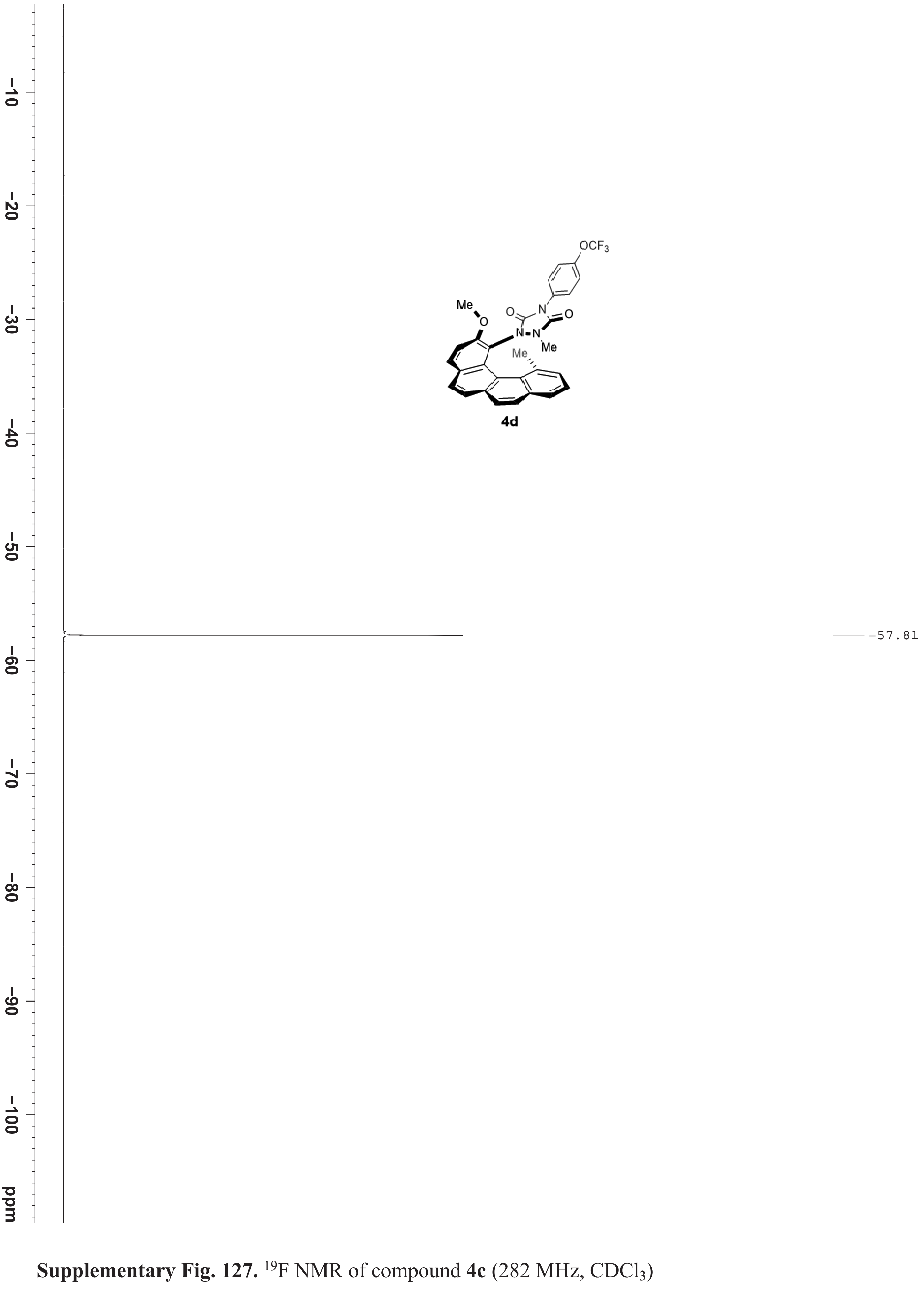

Supplementary Fig. 127. ^{19}F NMR of compound **4c** (282 MHz, $CDCl_3$)

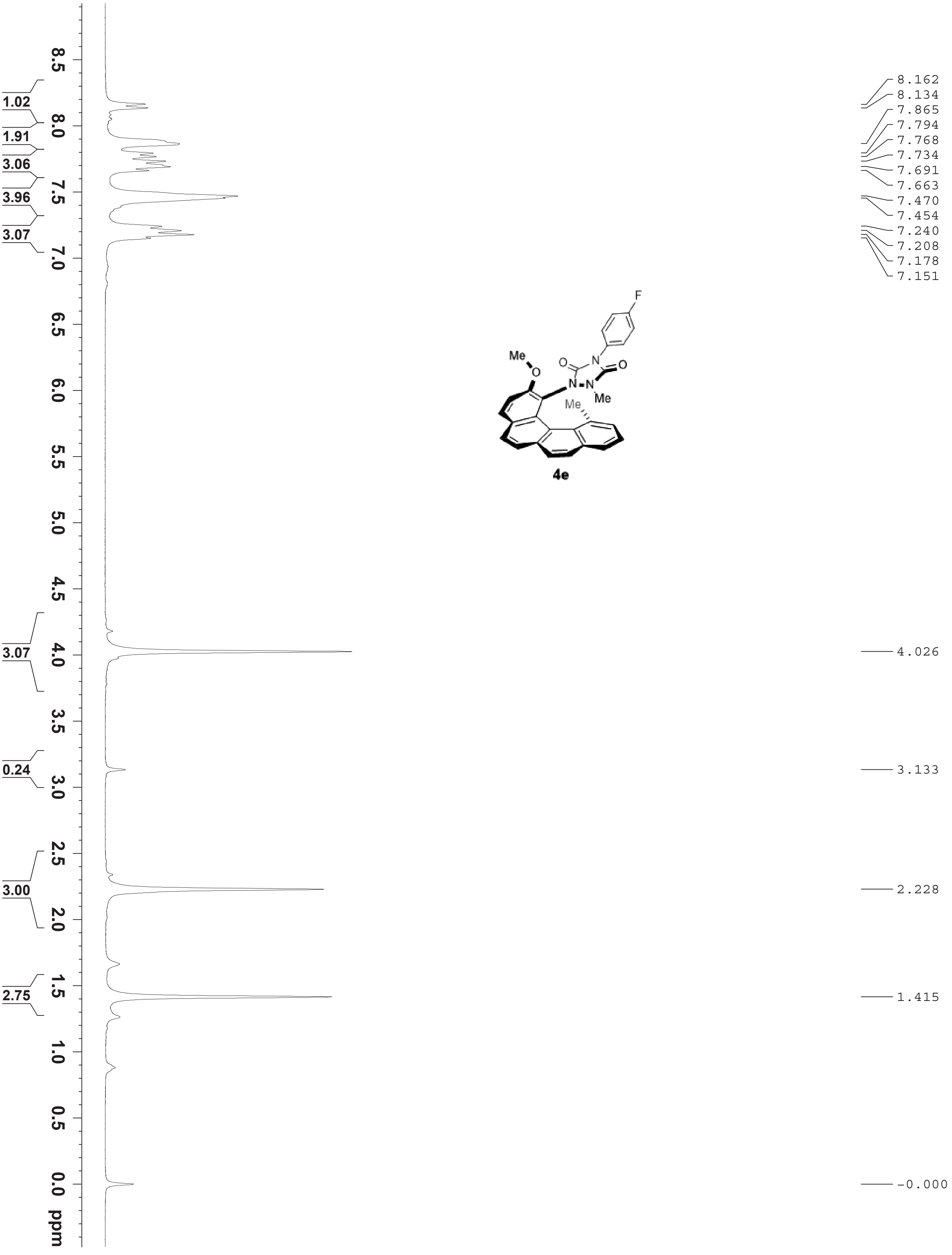

Supplementary Fig. 128. ^{1}H NMR of compound **4e** (300 MHz, $CDCl_3$)

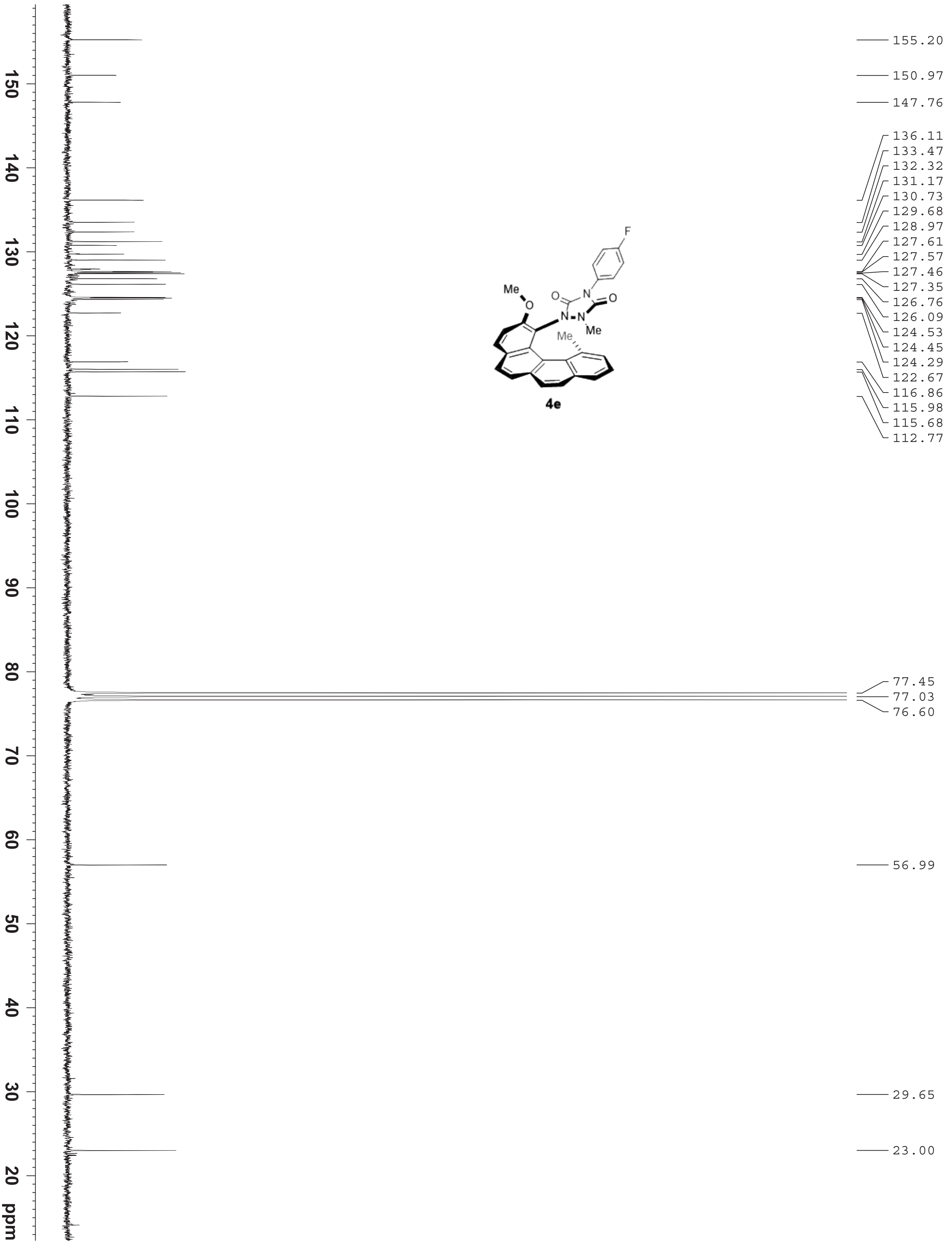

Supplementary Fig. 129. ^{13}C NMR of compound **4e** (75 MHz, $CDCl_3$)

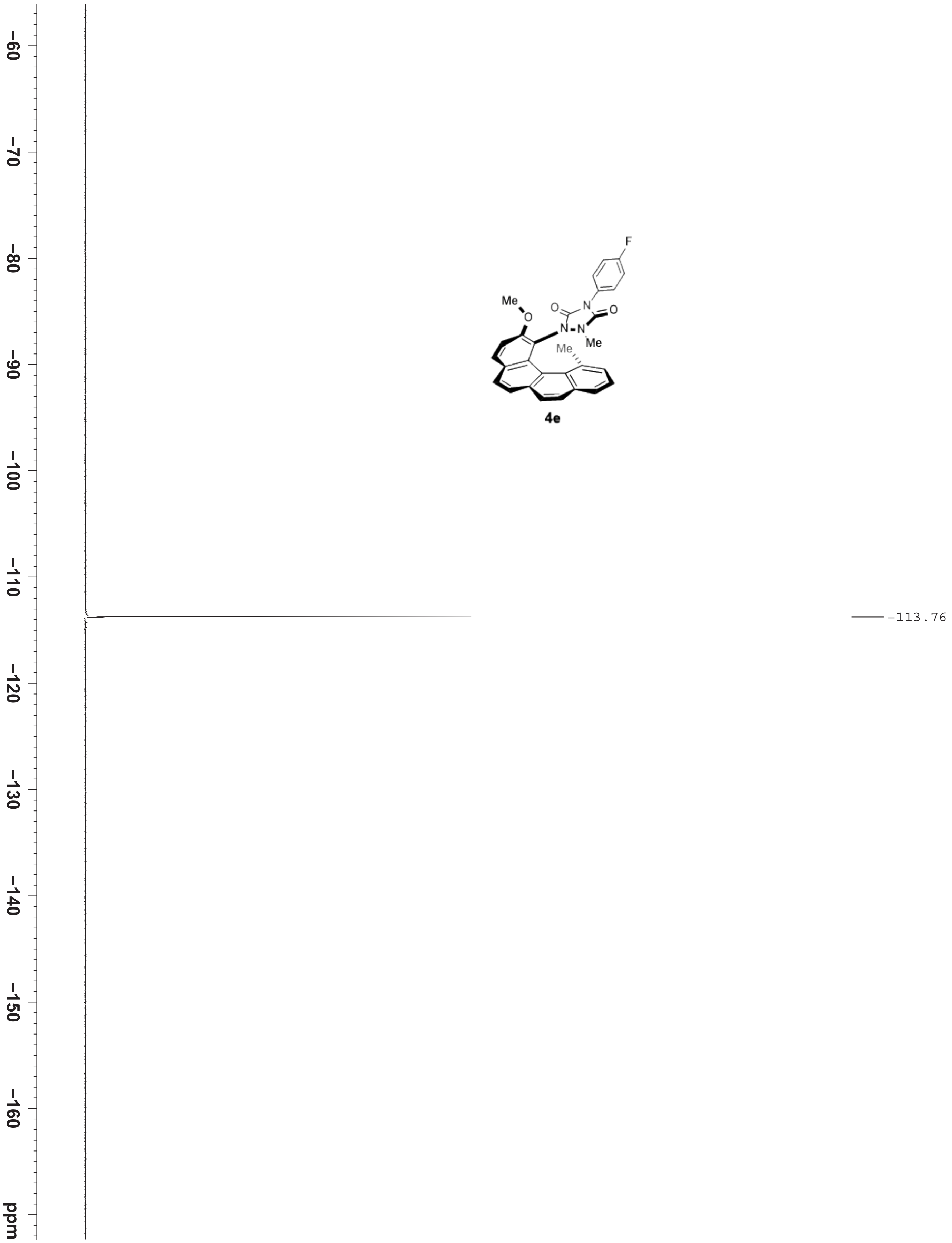

Supplementary Fig. 130. ^{19}F NMR of compound **4e** (282 MHz, $CDCl_3$)

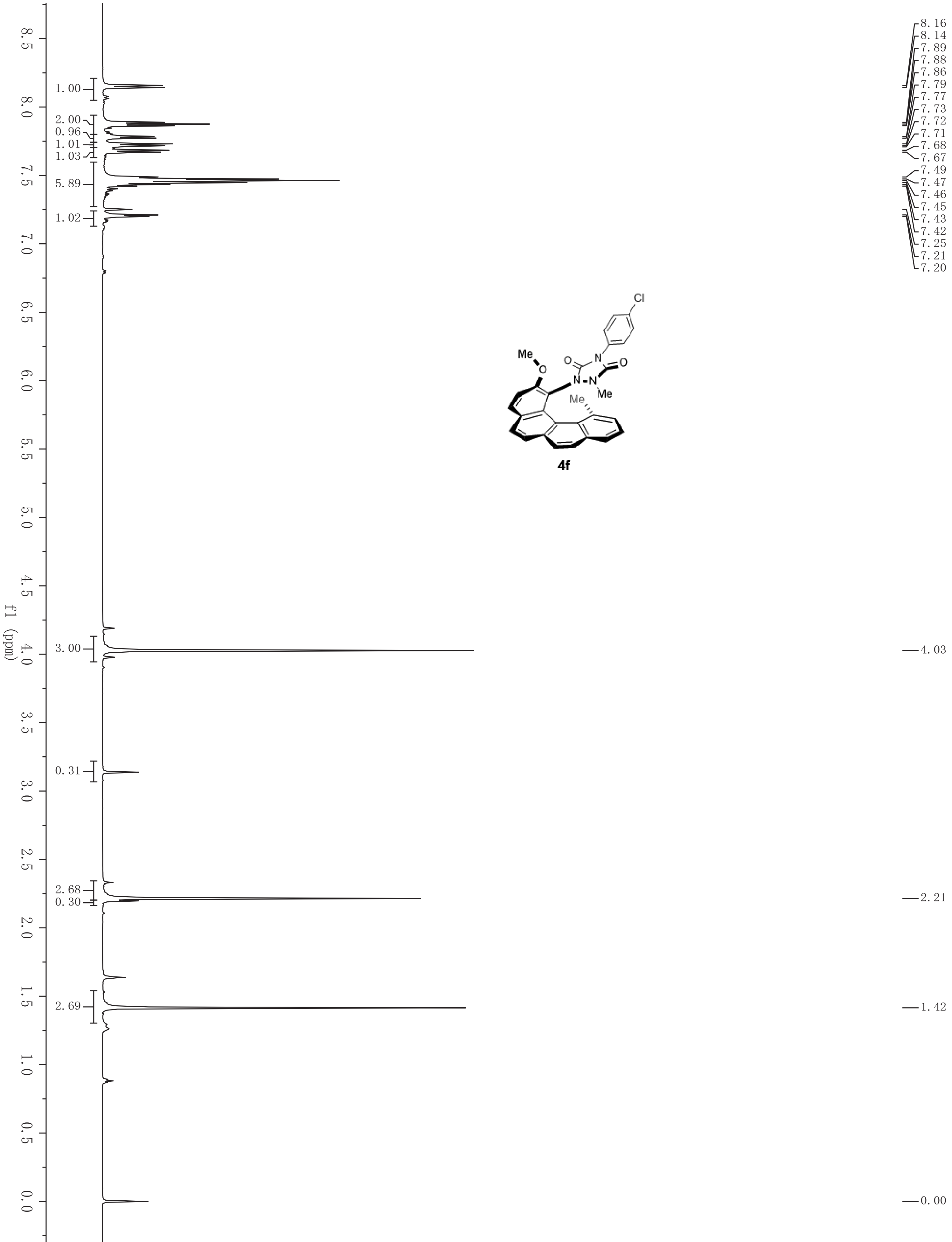

Supplementary Fig. 131. ^{1}H NMR of compound **4f** (600 MHz, $CDCl_3$)

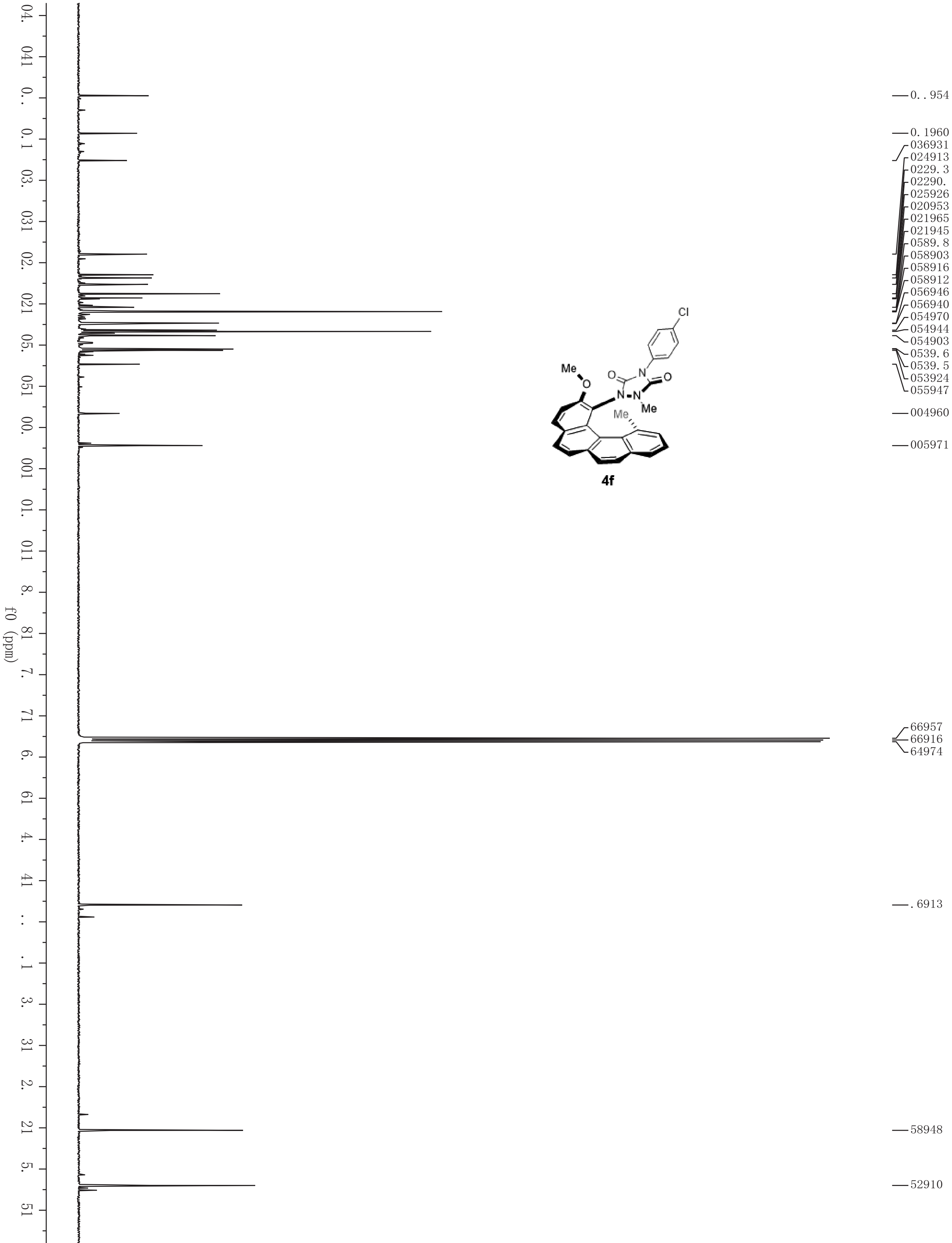

Supplementary Fig. 132. ^{13}C NMR of compound **4f** (151 MHz, $CDCl_3$)

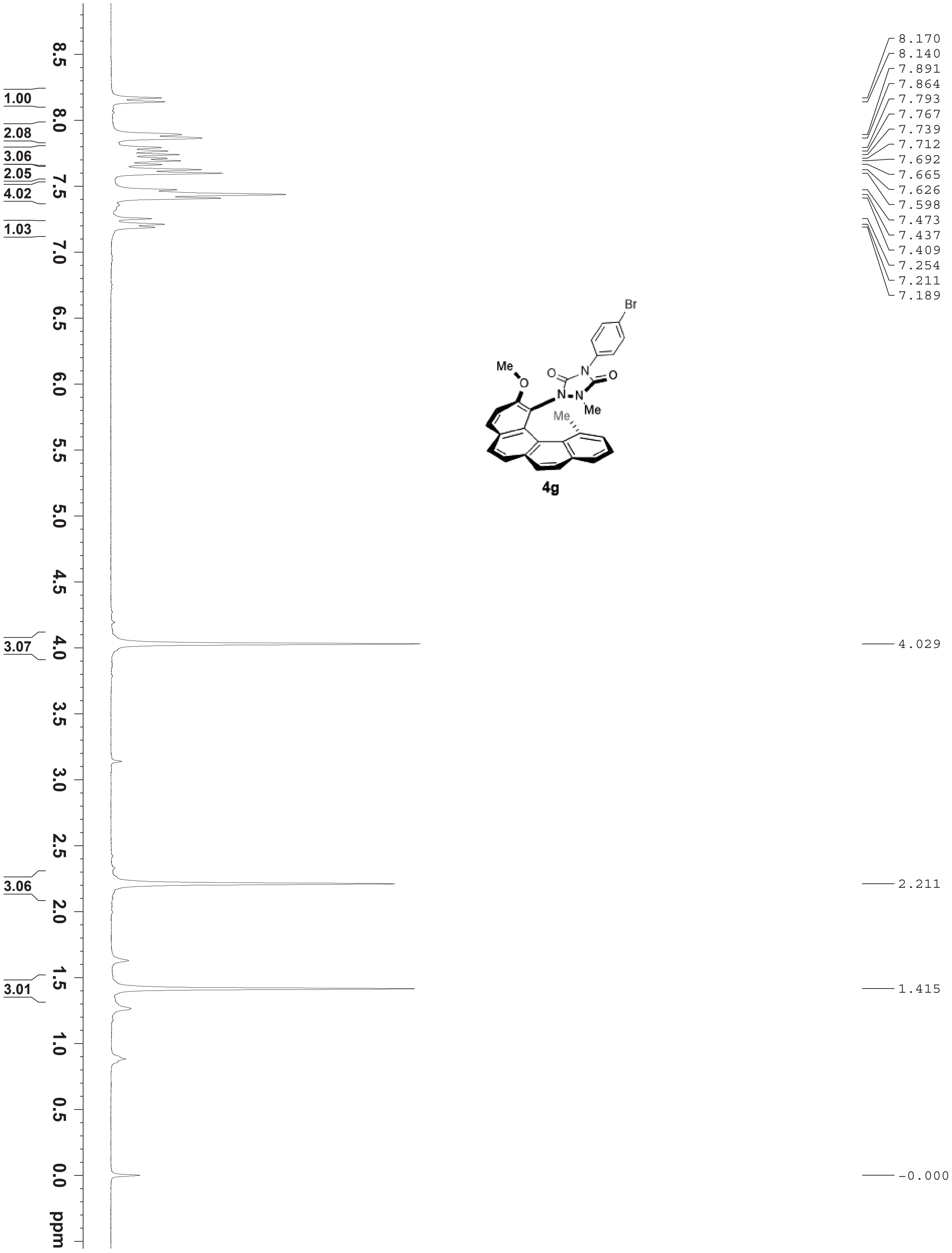

Supplementary Fig. 133. ^{1}H NMR of compound **4g** (300 MHz, $CDCl_3$)

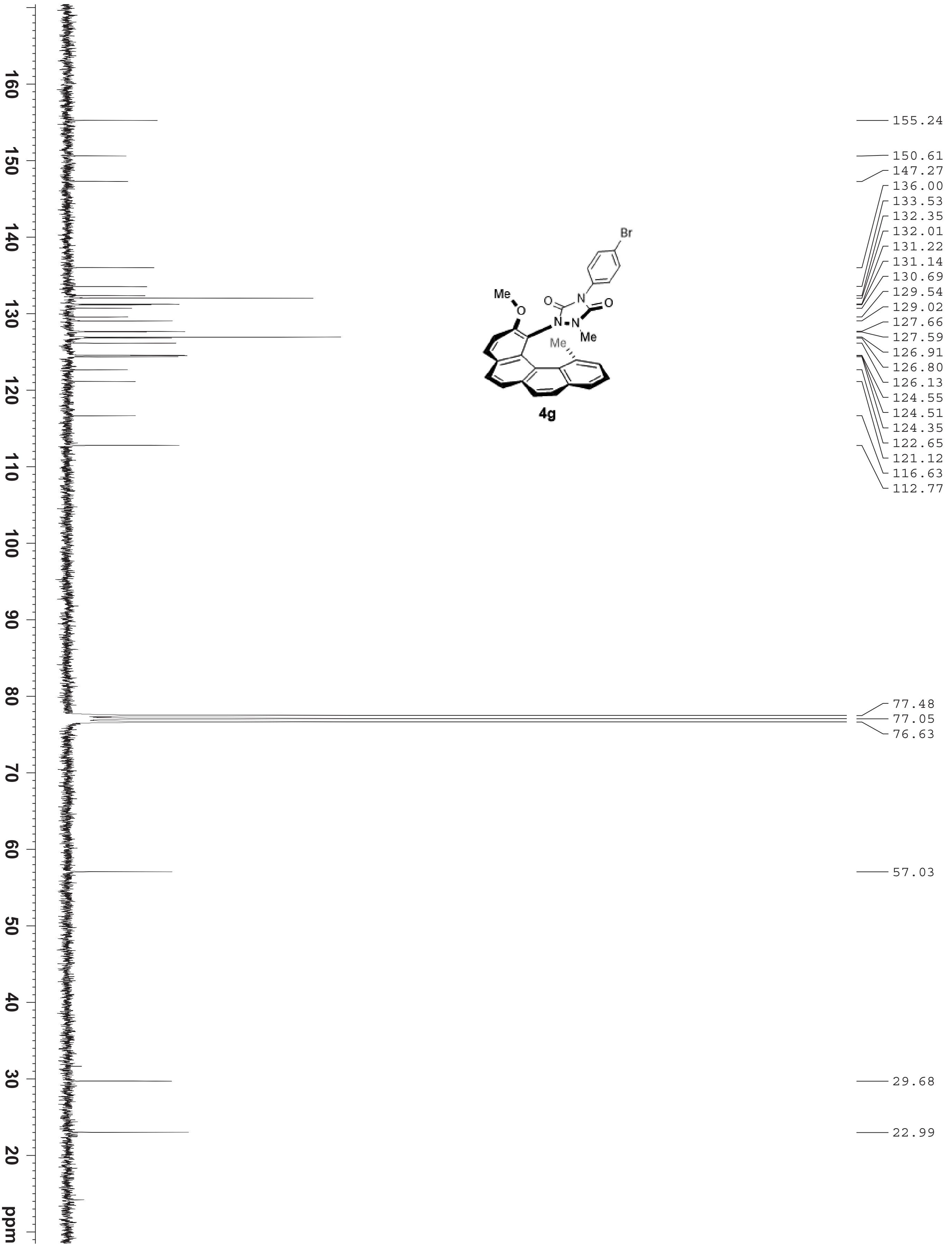

Supplementary Fig. 134. ^{13}C NMR of compound **4g** (75 MHz, $CDCl_3$)

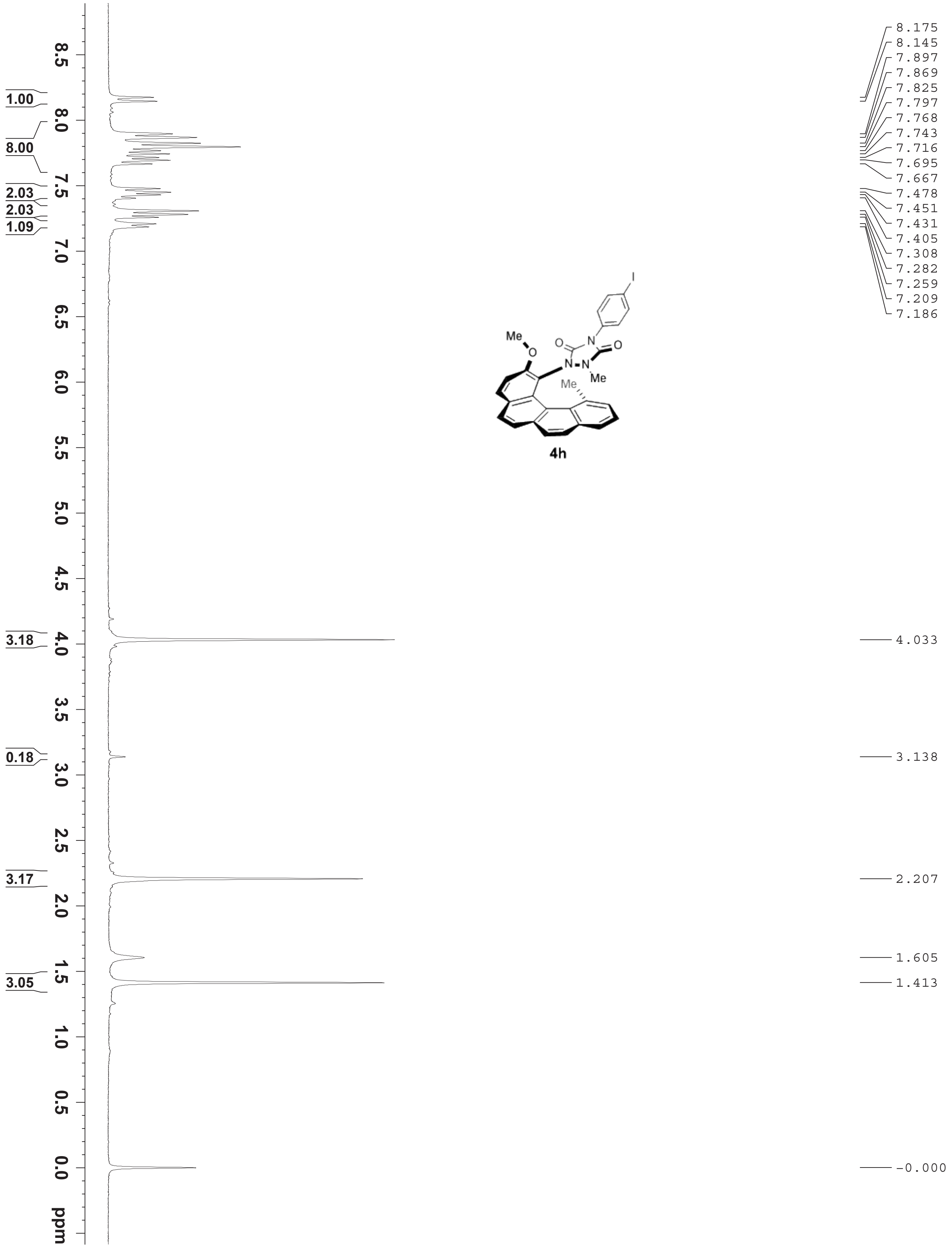

Supplementary Fig. 135. ^{1}H NMR of compound **4h** (300 MHz, $CDCl_3$)

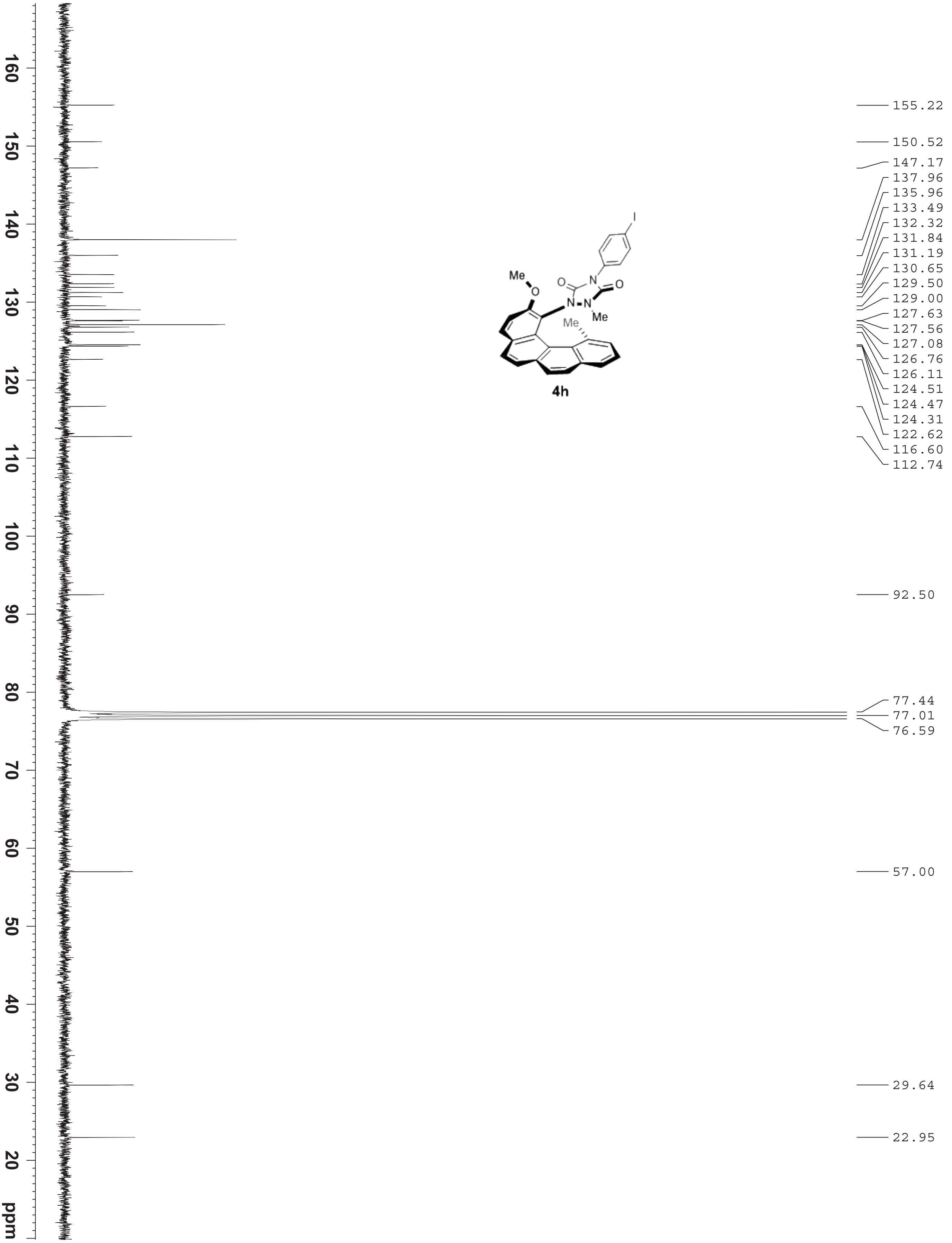

Supplementary Fig. 136. ^{13}C NMR of compound **4h** (75 MHz, $CDCl_3$)

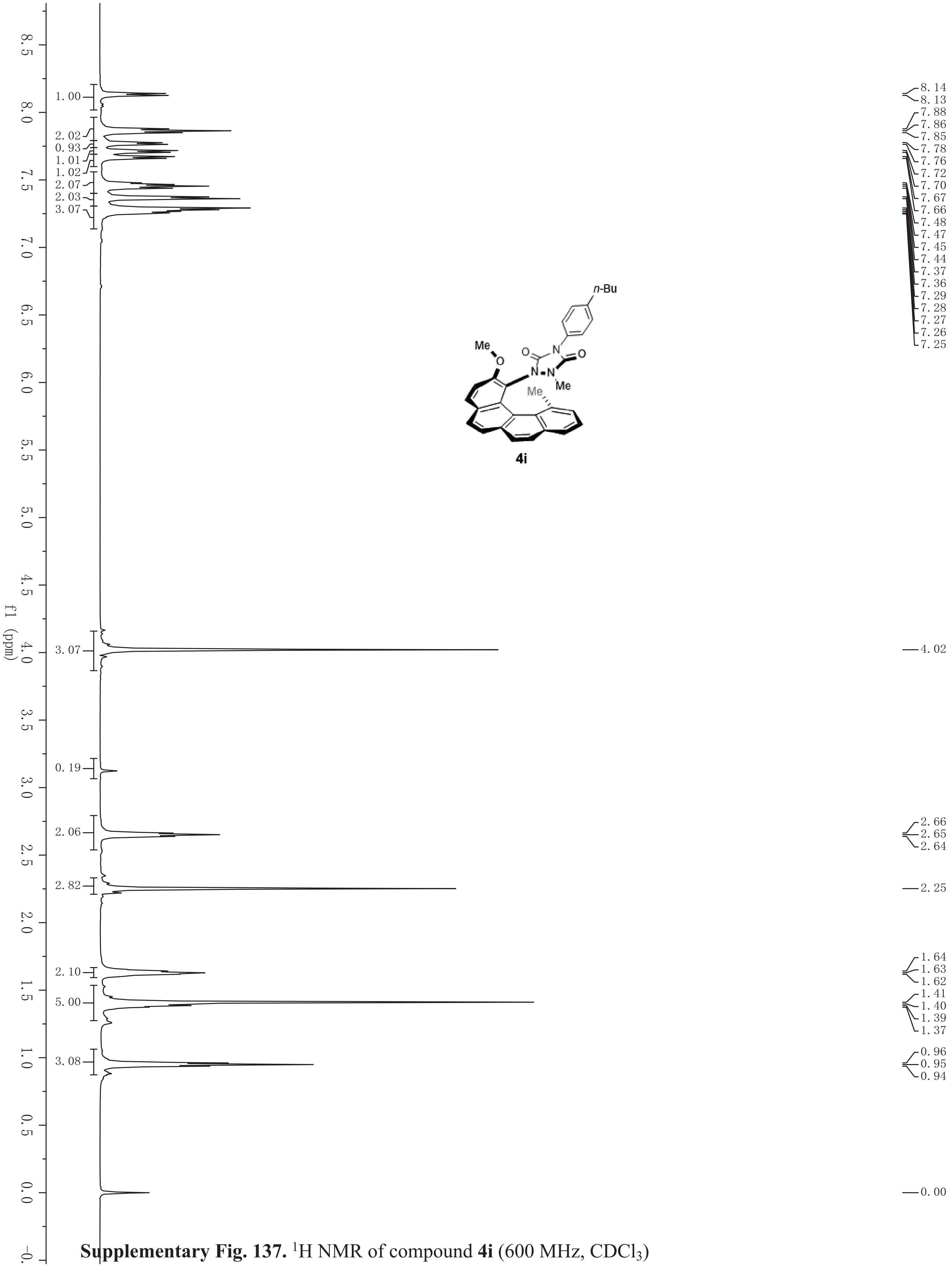

Supplementary Fig. 137. ^{1}H NMR of compound **4i** (600 MHz, $CDCl_3$)

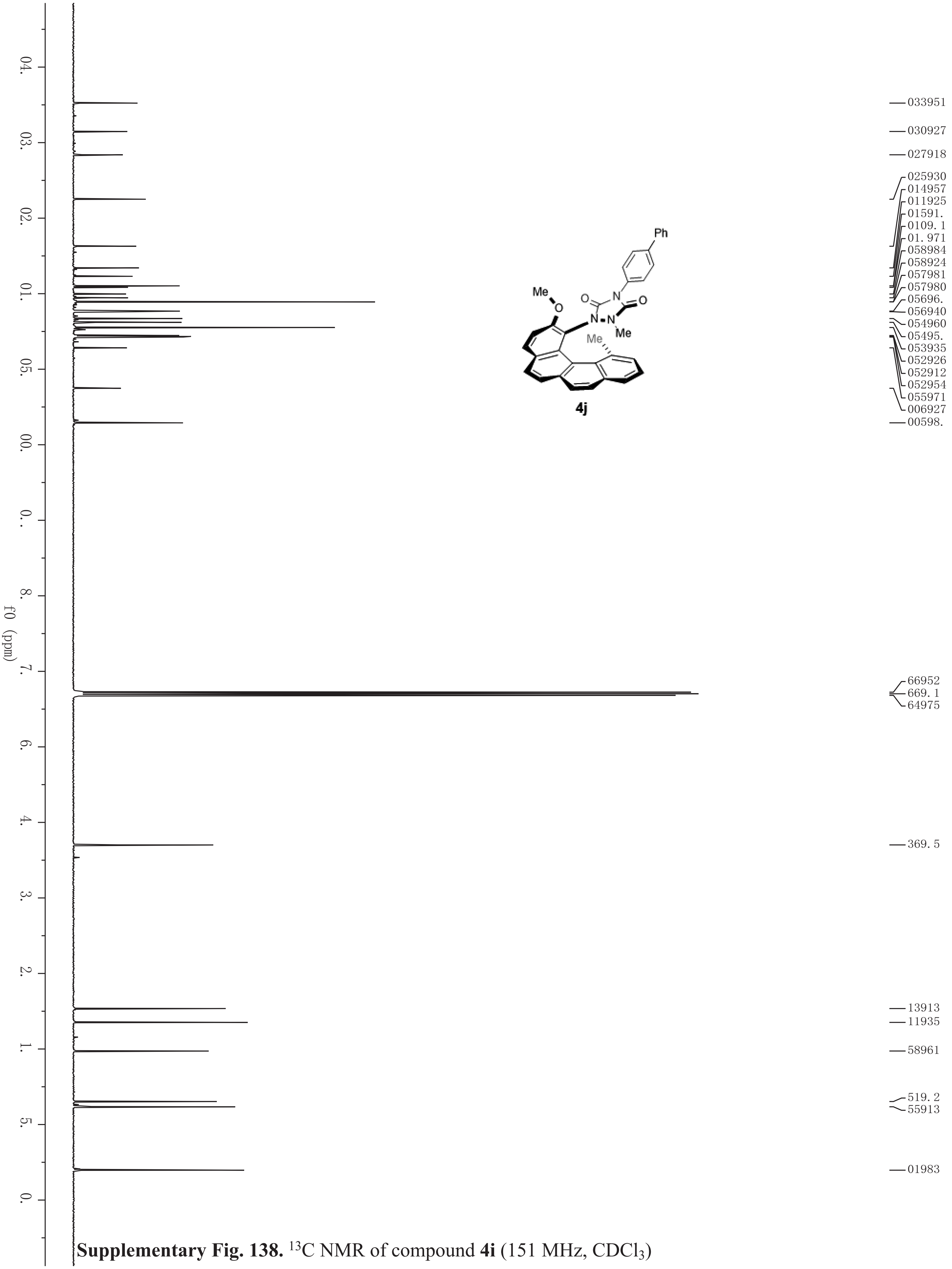

Supplementary Fig. 138. ^{13}C NMR of compound **4i** (151 MHz, $CDCl_3$)

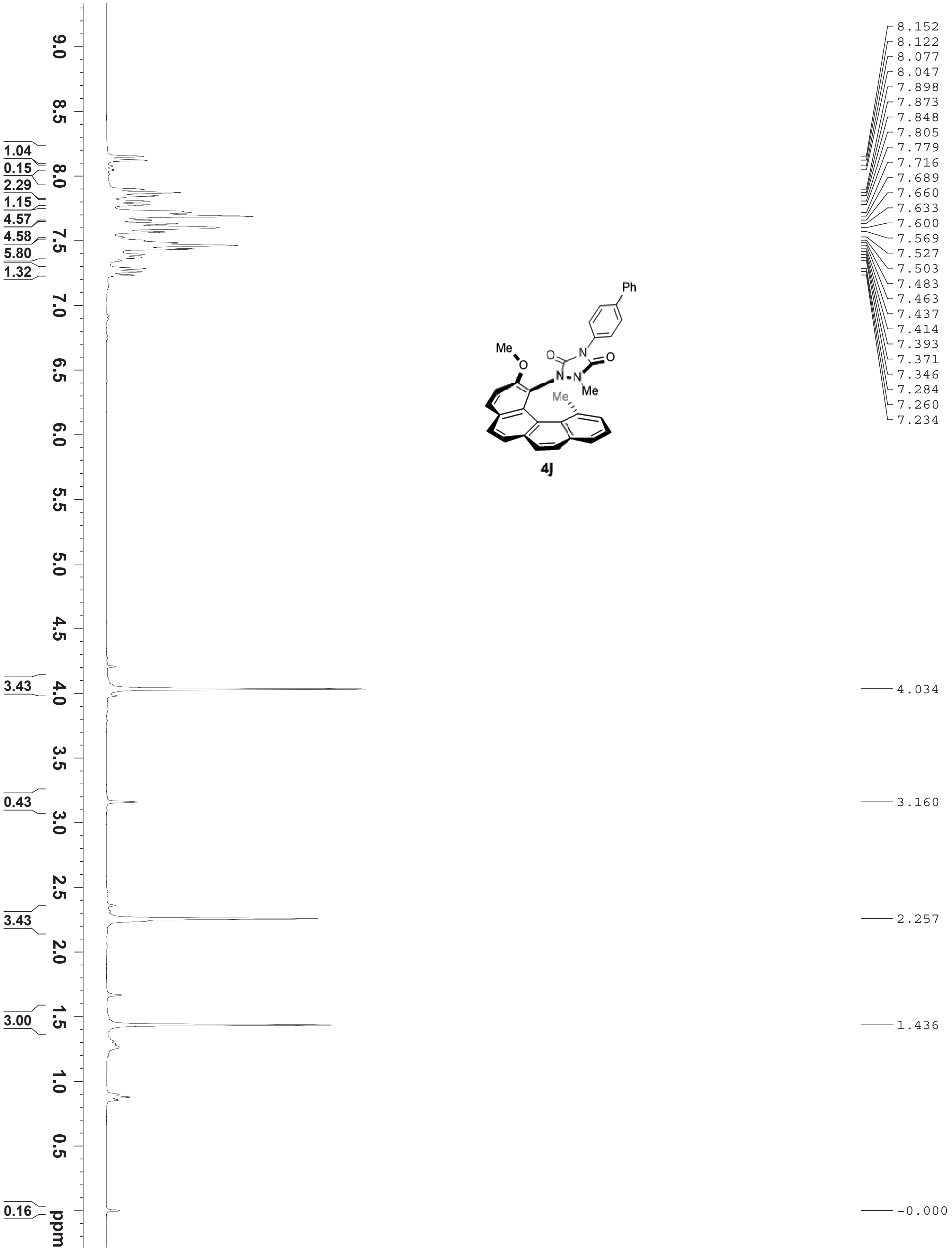

Supplementary Fig. 139. ^{1}H NMR of compound **4j** (300 MHz, $CDCl_3$)

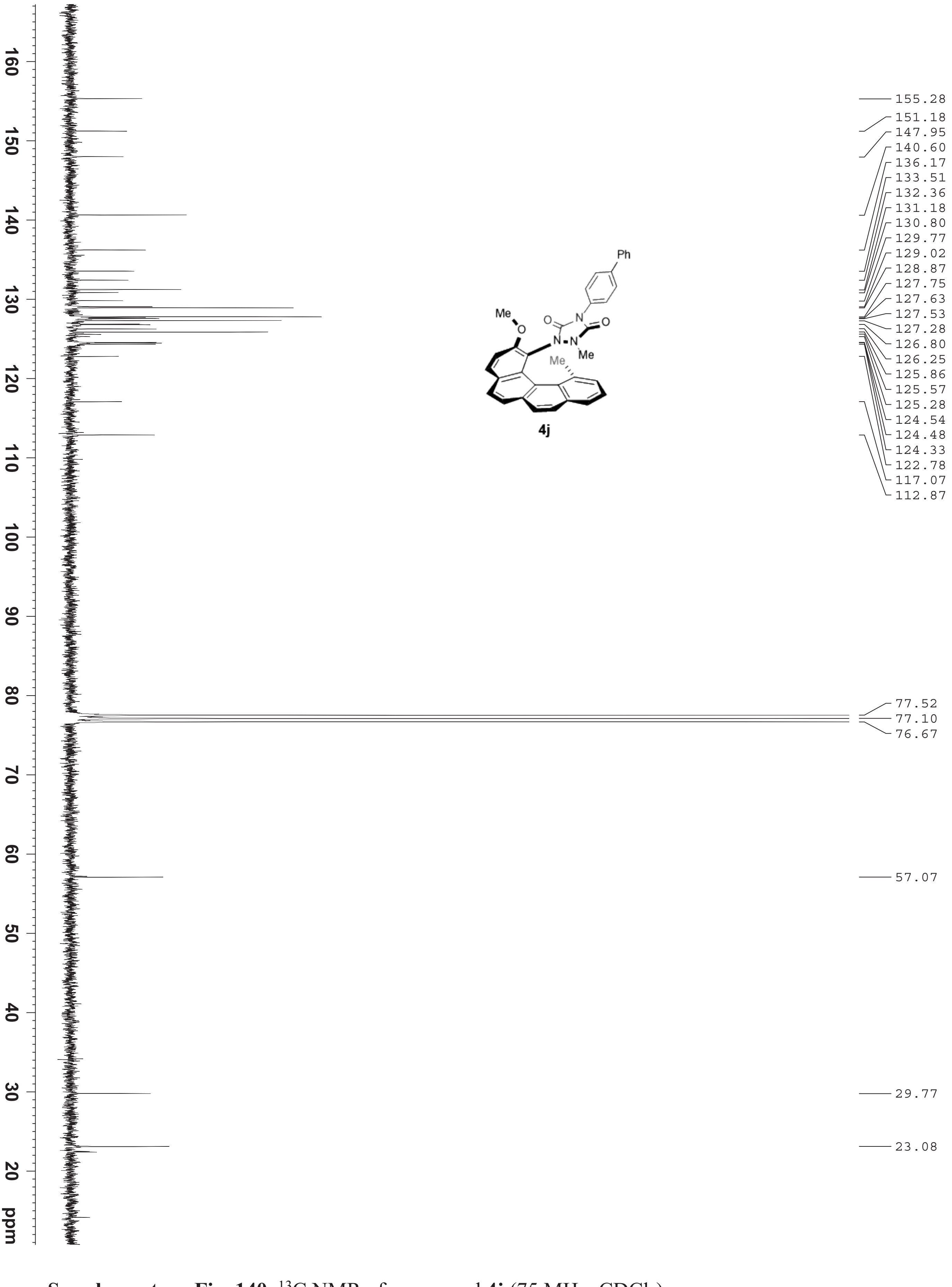

Supplementary Fig. 140. ^{13}C NMR of compound **4j** (75 MHz, $CDCl_3$)

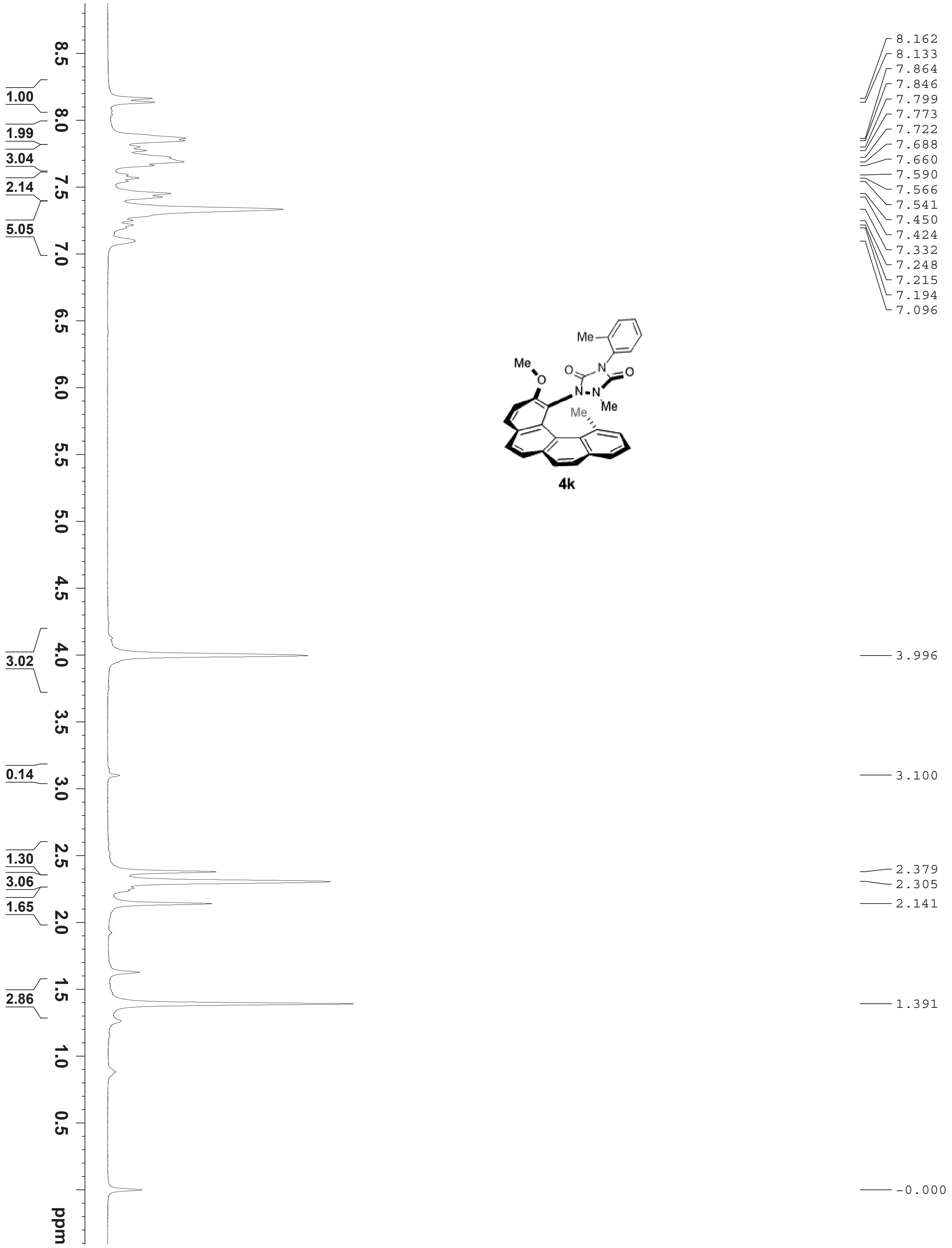

Supplementary Fig. 141. ^{1}H NMR of compound **4k** (300 MHz, $CDCl_3$)

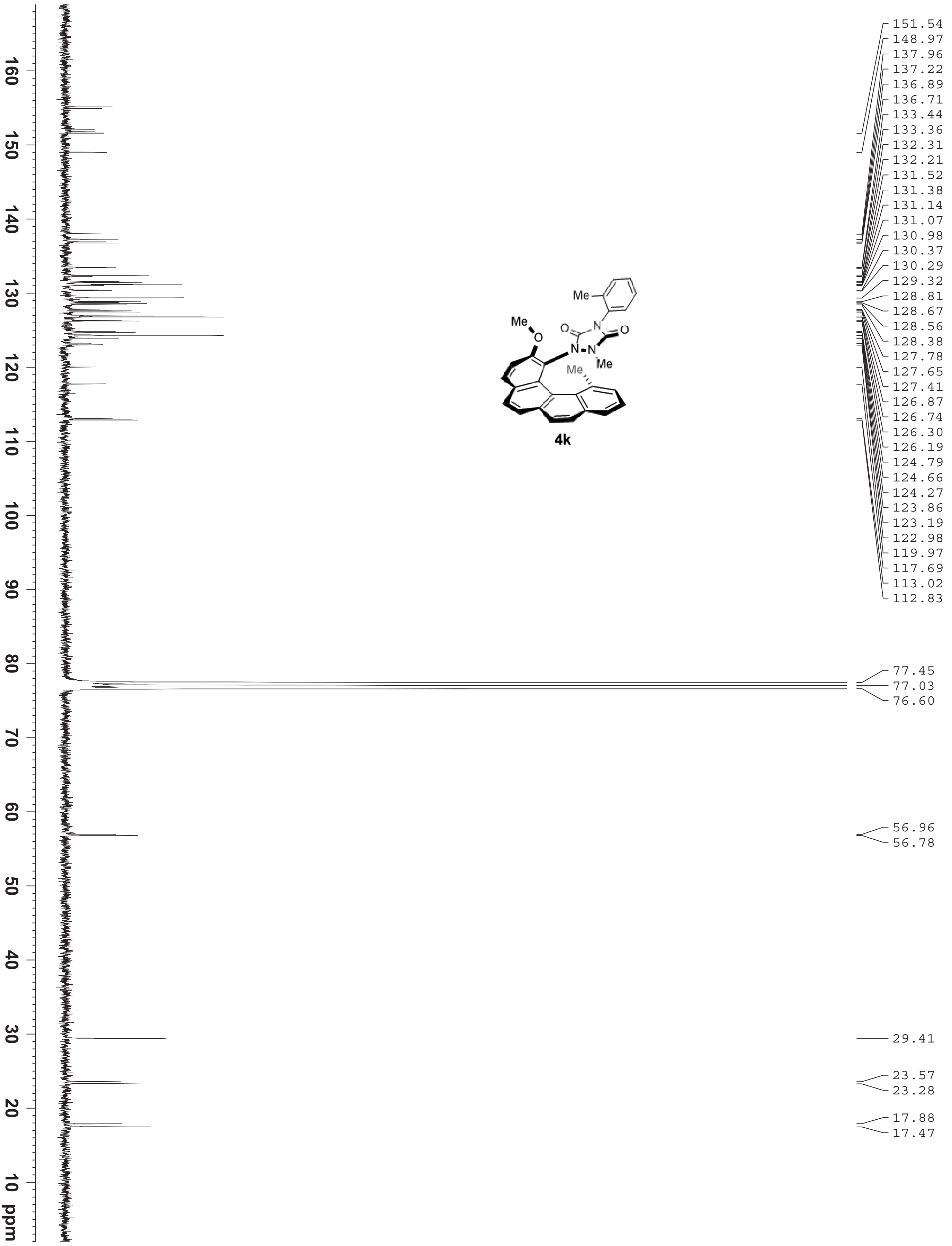

Supplementary Fig. 142. ^{13}C NMR of compound **4k** (75 MHz, $CDCl_3$)

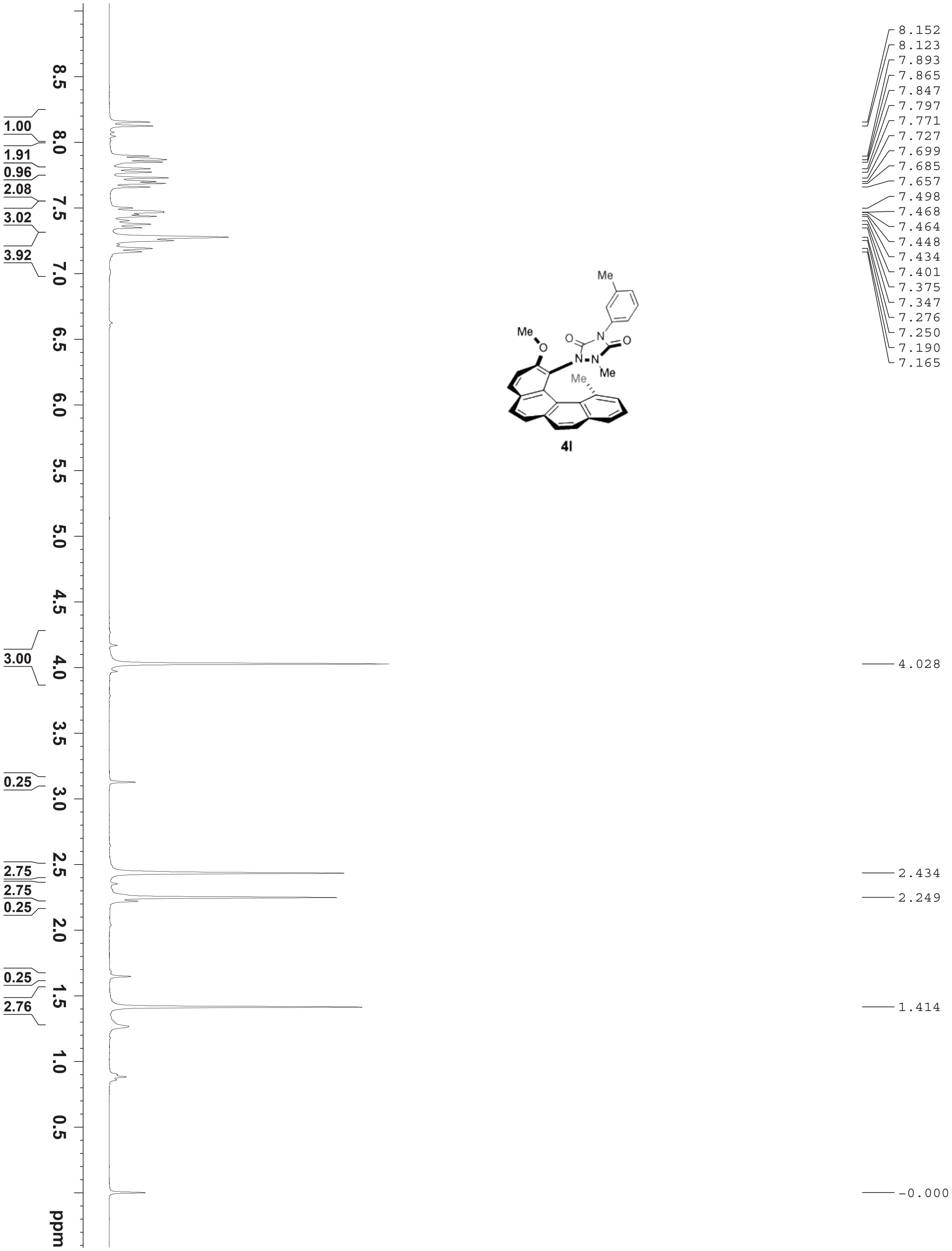

Supplementary Fig. 143. ^{1}H NMR of compound **4l** (300 MHz, $CDCl_3$)

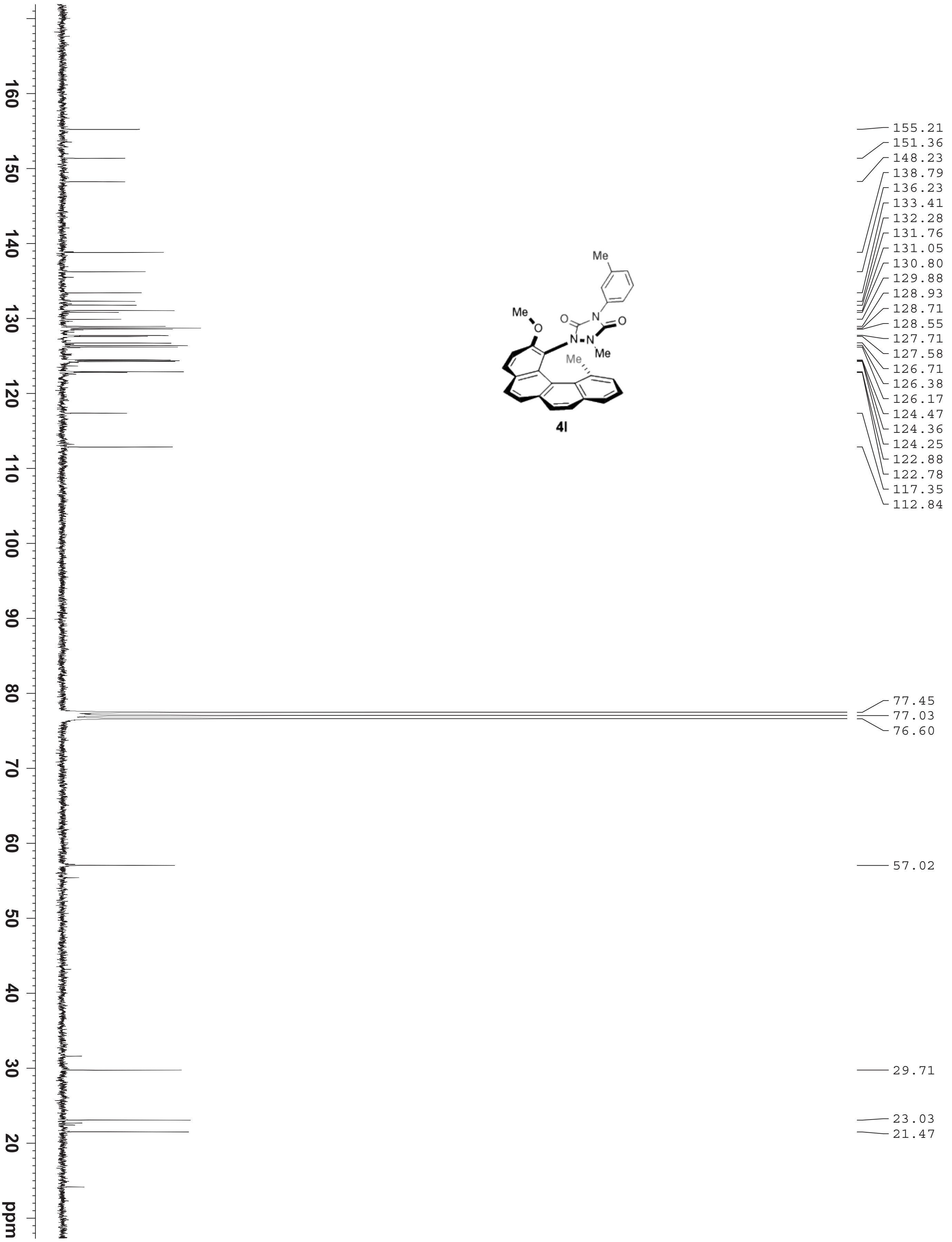

Supplementary Fig. 144. ^{13}C NMR of compound **4l** (75 MHz, $CDCl_3$)

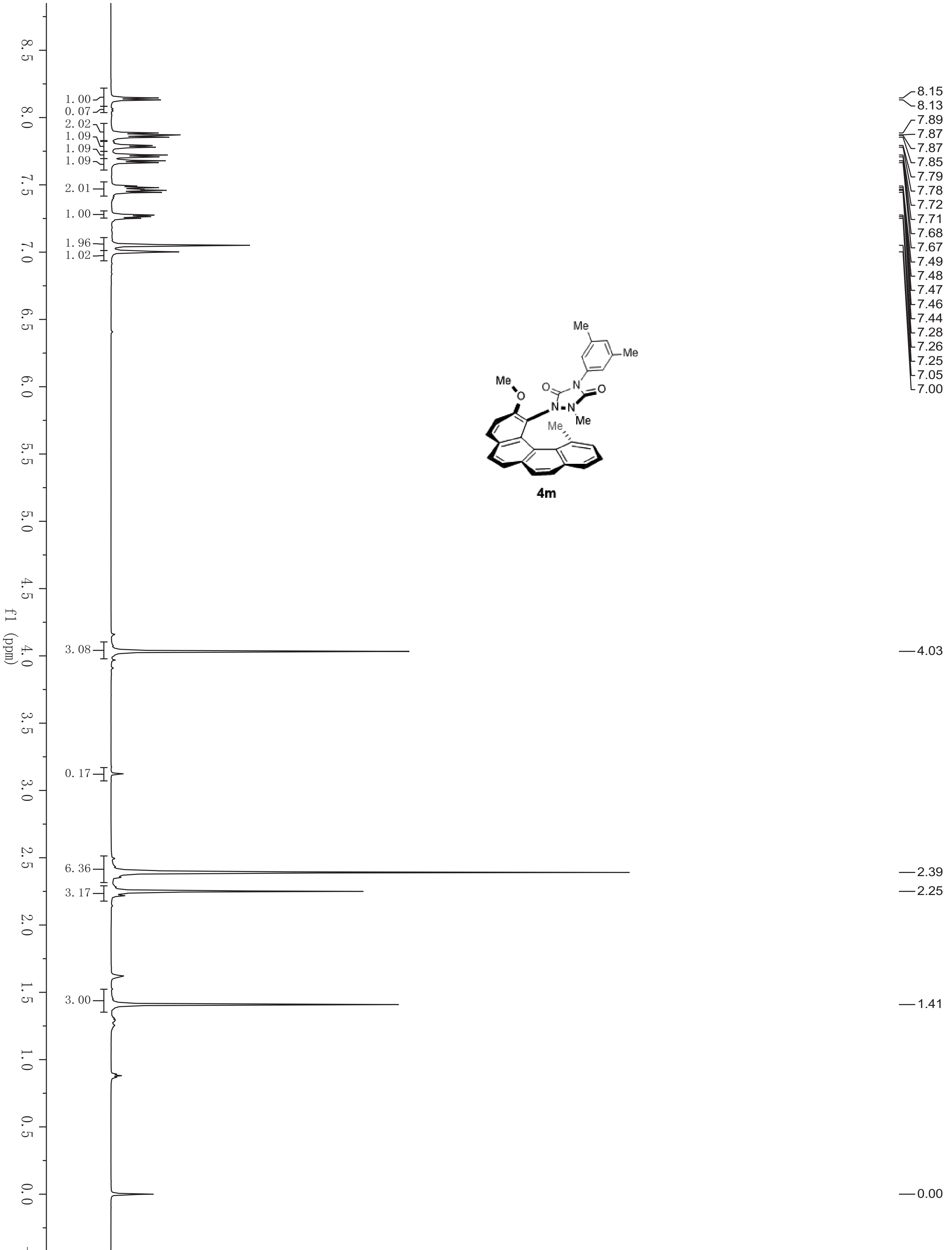

Supplementary Fig. 145. ^{1}H NMR of compound **4m** (600 MHz, $CDCl_3$)

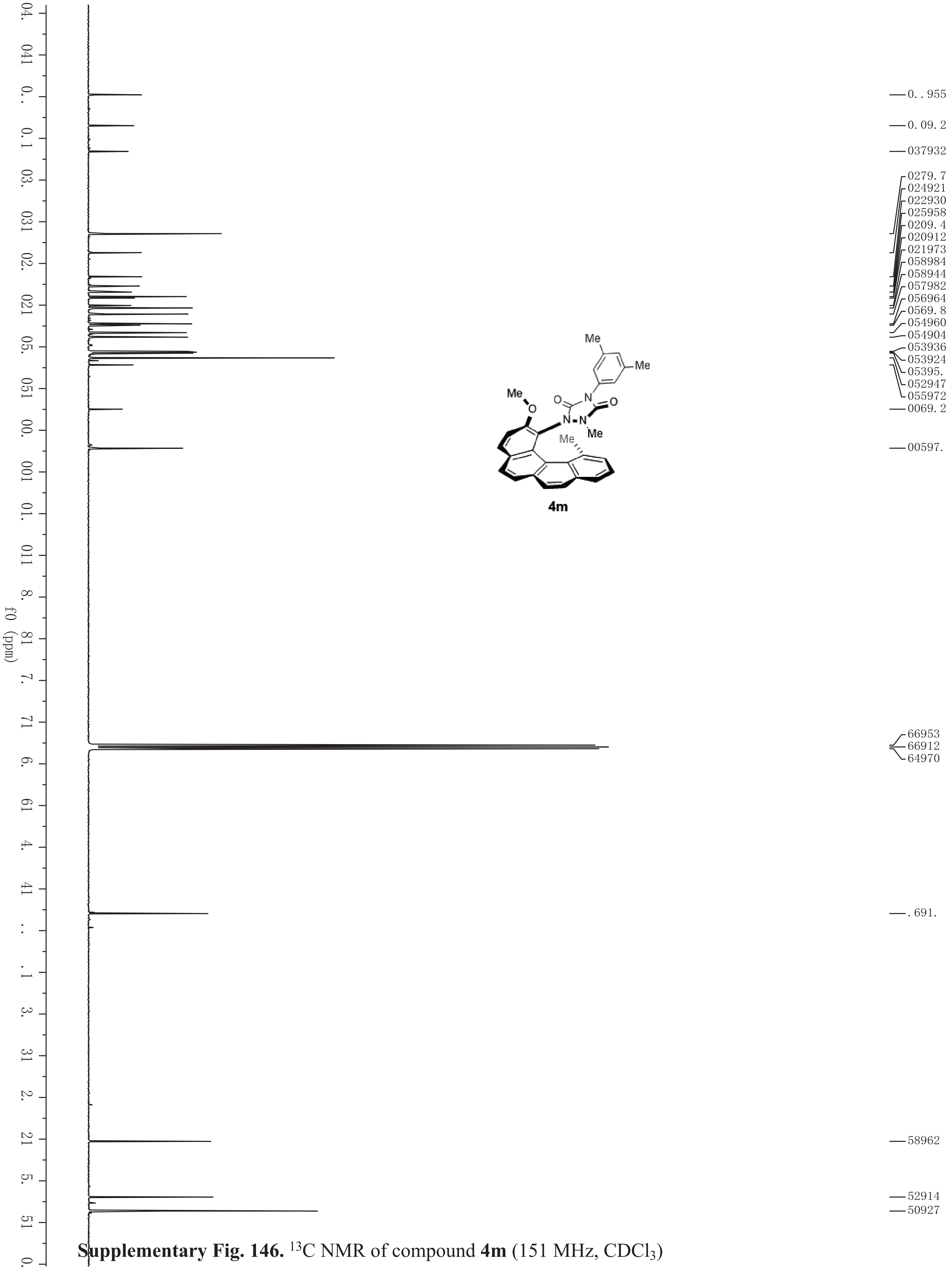

Supplementary Fig. 146. ^{13}C NMR of compound **4m** (151 MHz, $CDCl_3$)

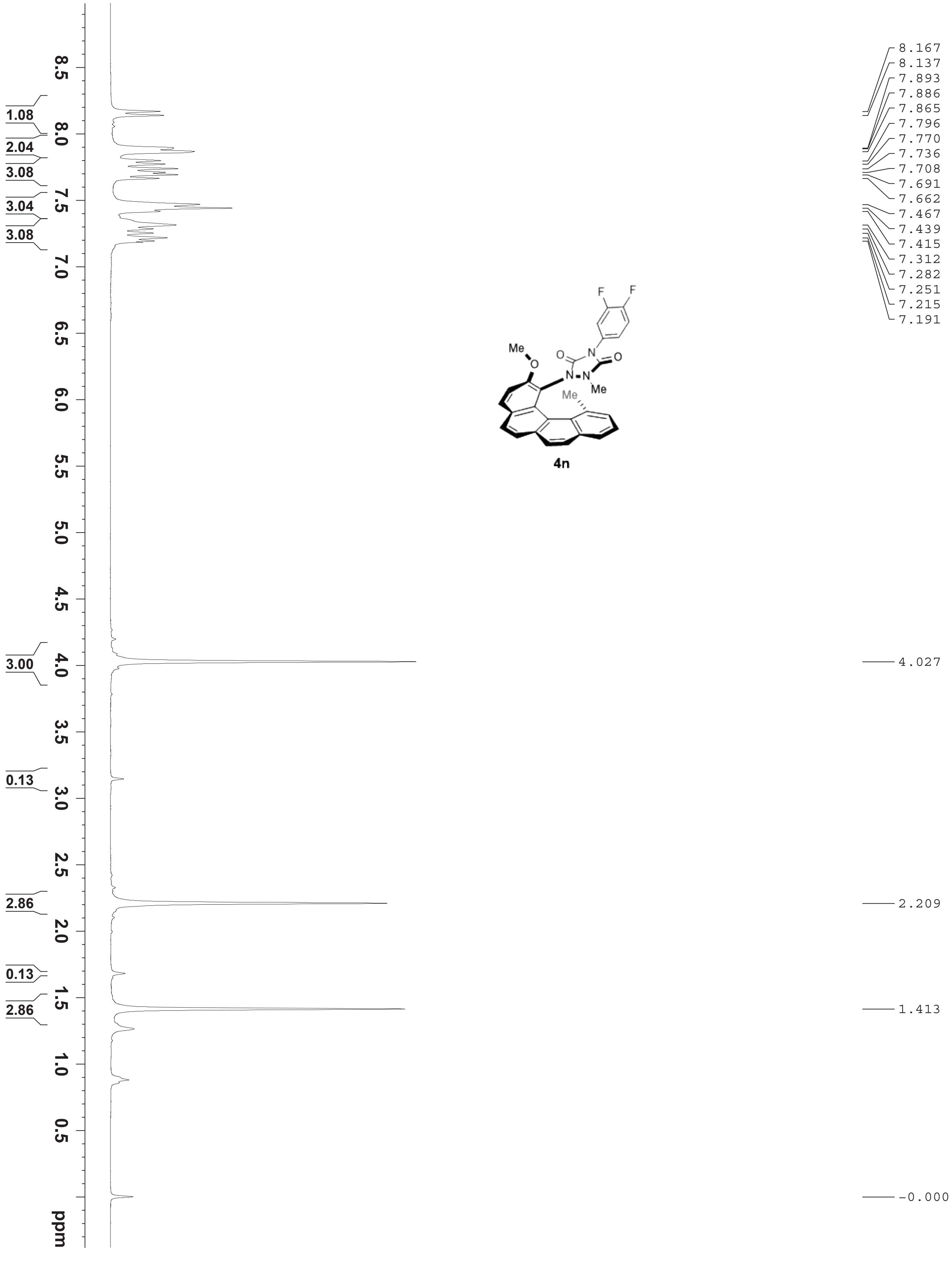

Supplementary Fig. 147. ^{1}H NMR of compound **4n** (300 MHz, $CDCl_3$)

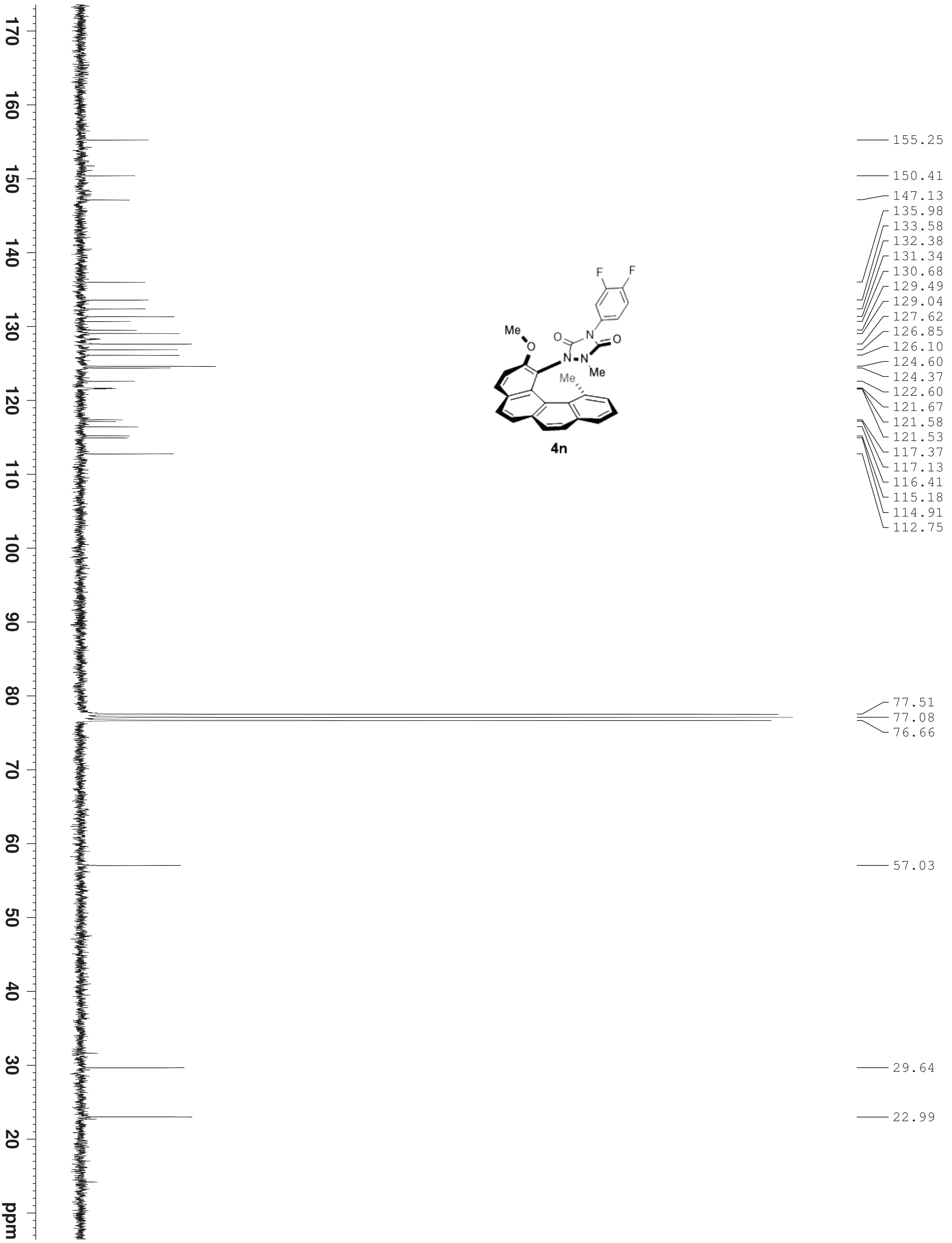

Supplementary Fig. 148. ^{13}C NMR of compound **4n** (75 MHz, $CDCl_3$)

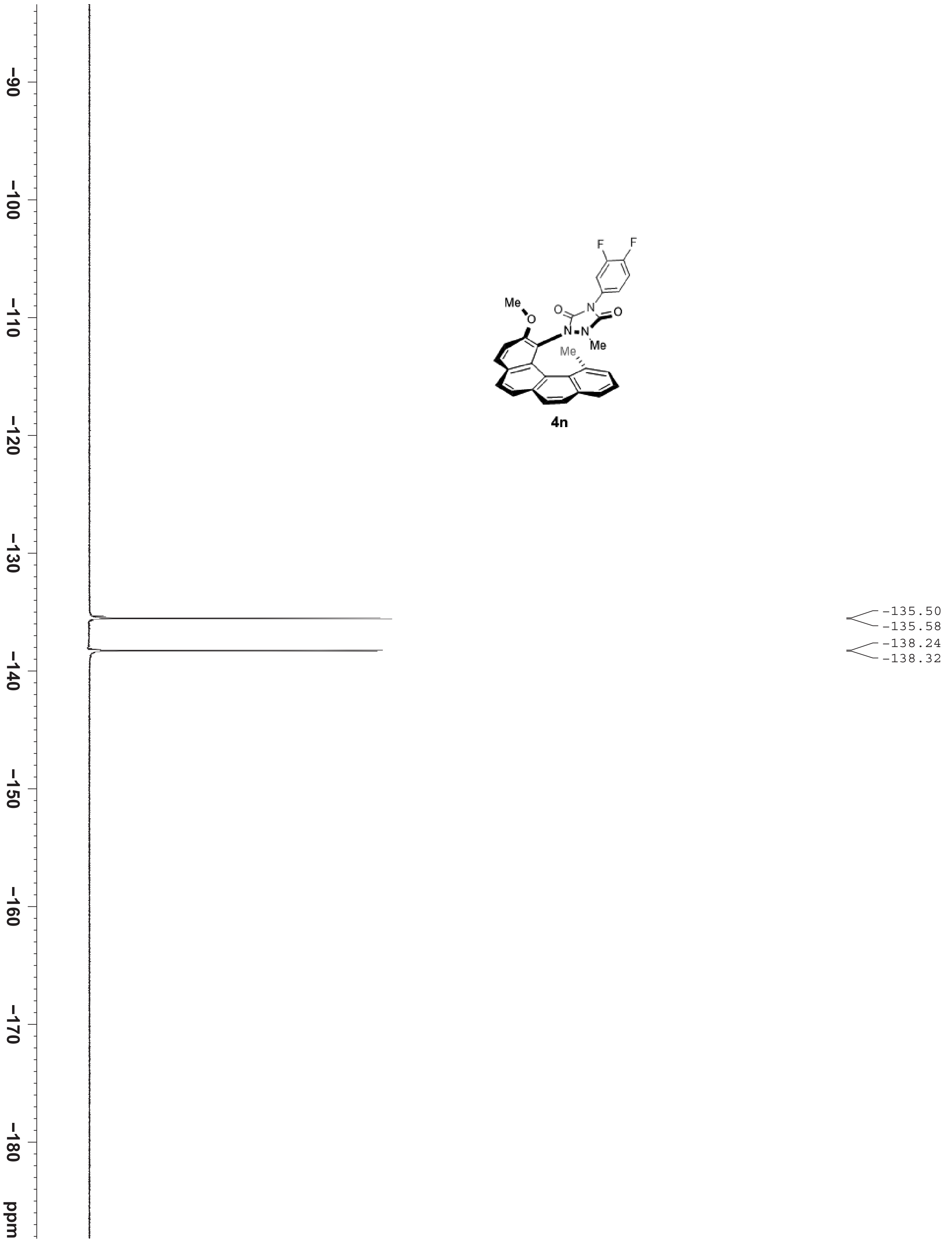

Supplementary Fig. 149. ^{19}F NMR of compound **4n** (282 MHz, $CDCl_3$)

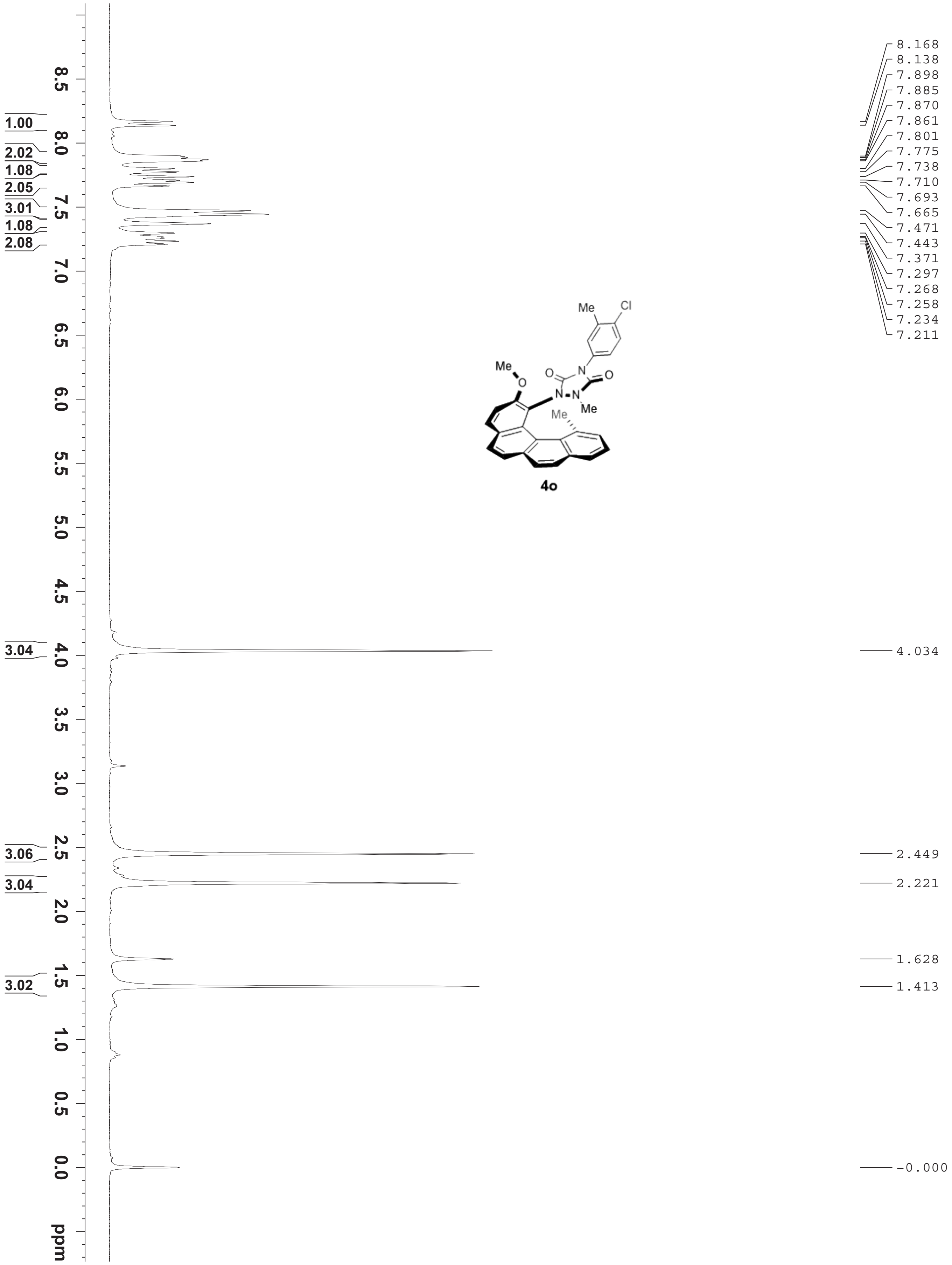

Supplementary Fig. 150. ^{1}H NMR of compound **4o** (300 MHz, $CDCl_3$)

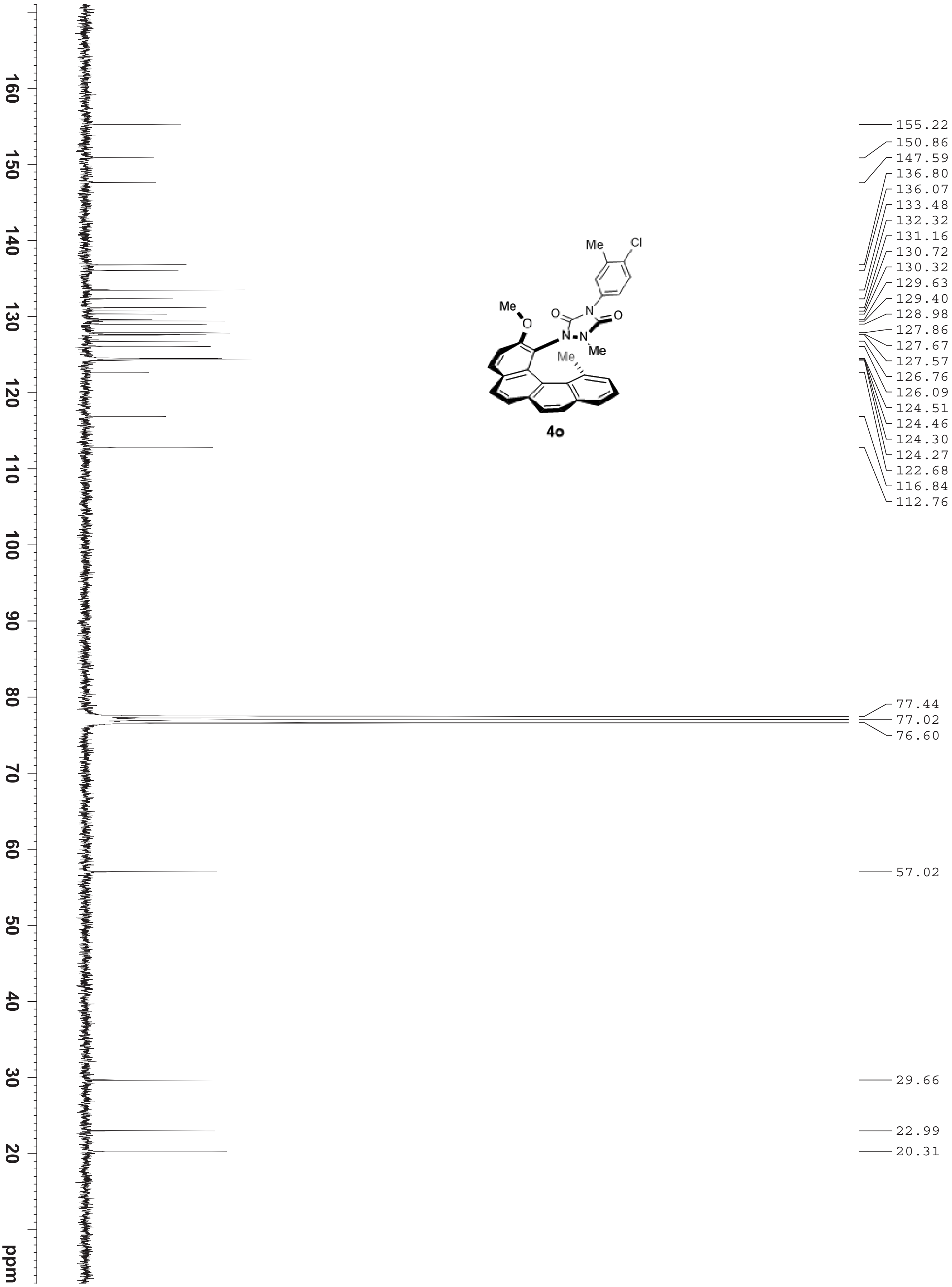

Supplementary Fig. 151. ^{13}C NMR of compound **4o** (75 MHz, $CDCl_3$)

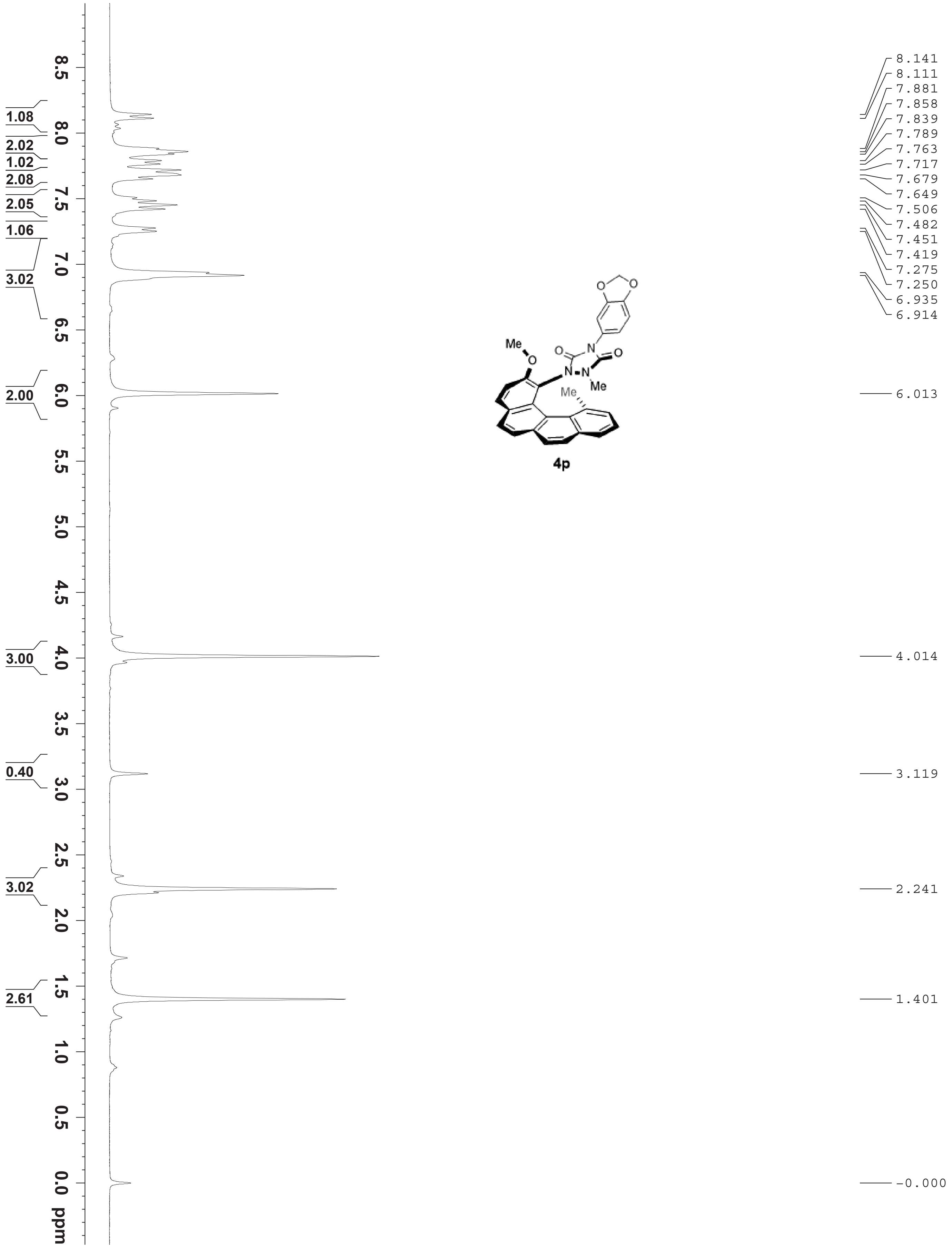

Supplementary Fig. 152. ^{1}H NMR of compound **4p** (300 MHz, $CDCl_3$)

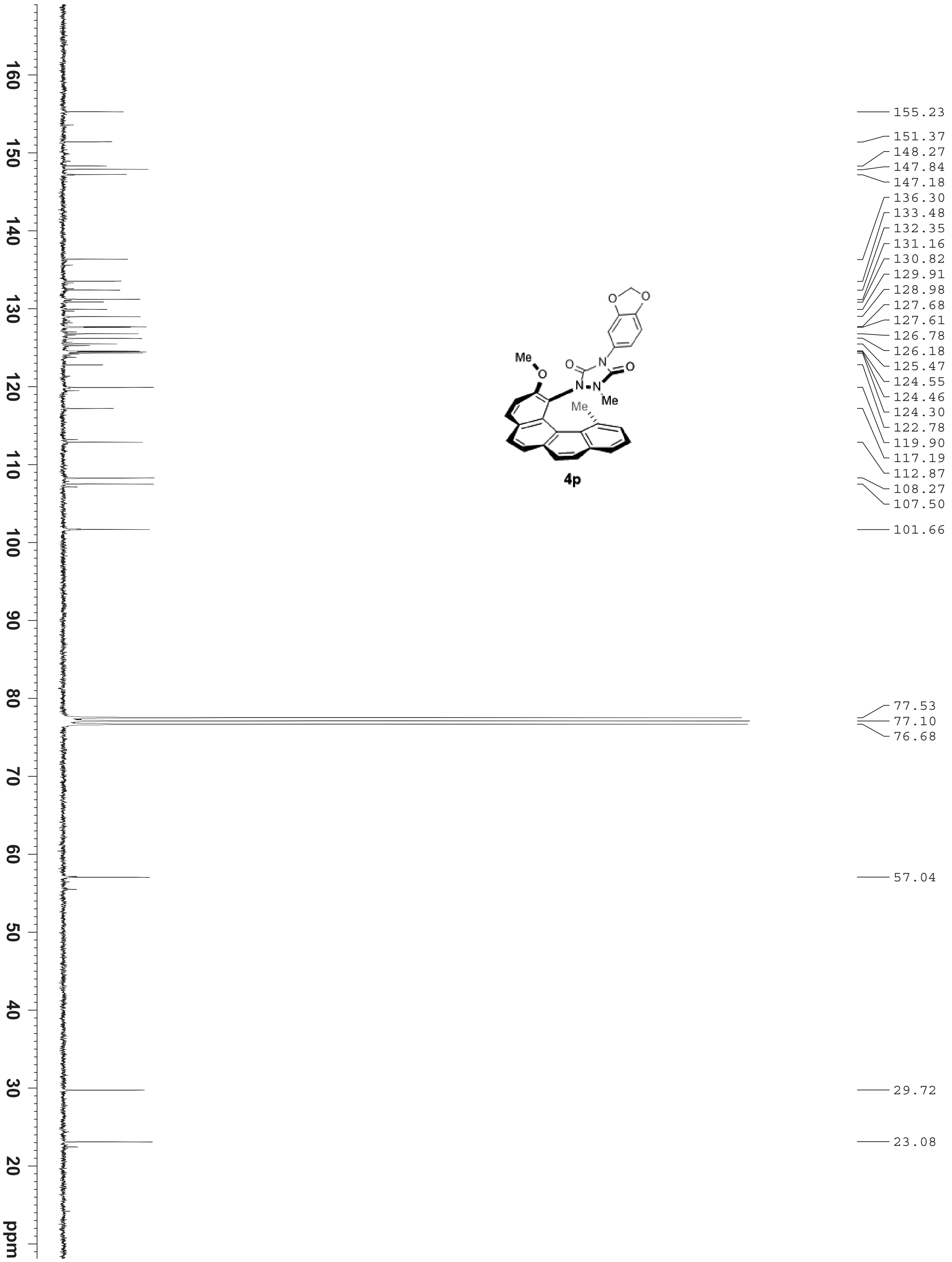

Supplementary Fig. 153. ^{13}C NMR of compound **4p** (75 MHz, $CDCl_3$)

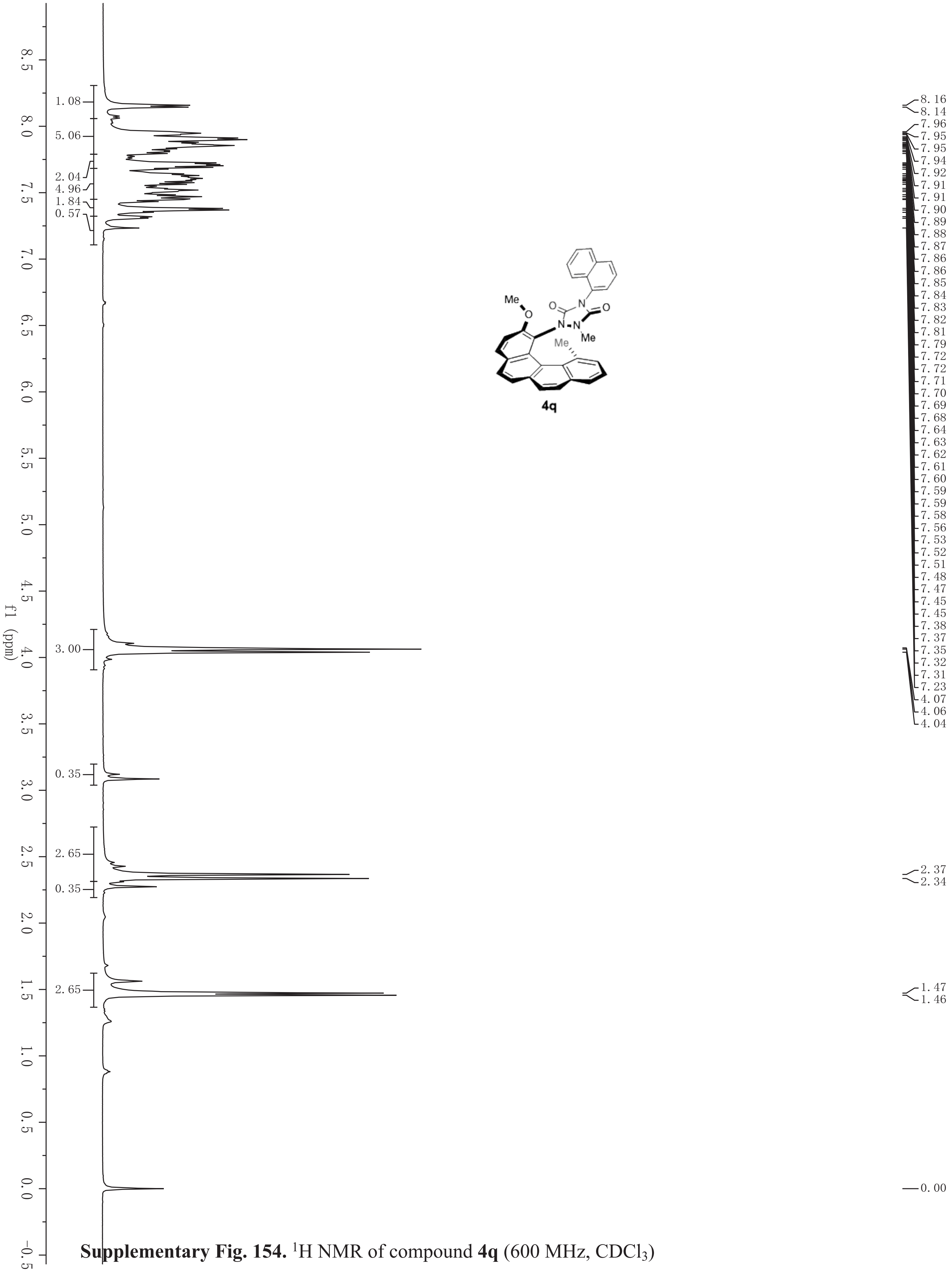

Supplementary Fig. 154. ^{1}H NMR of compound **4q** (600 MHz, $CDCl_3$)

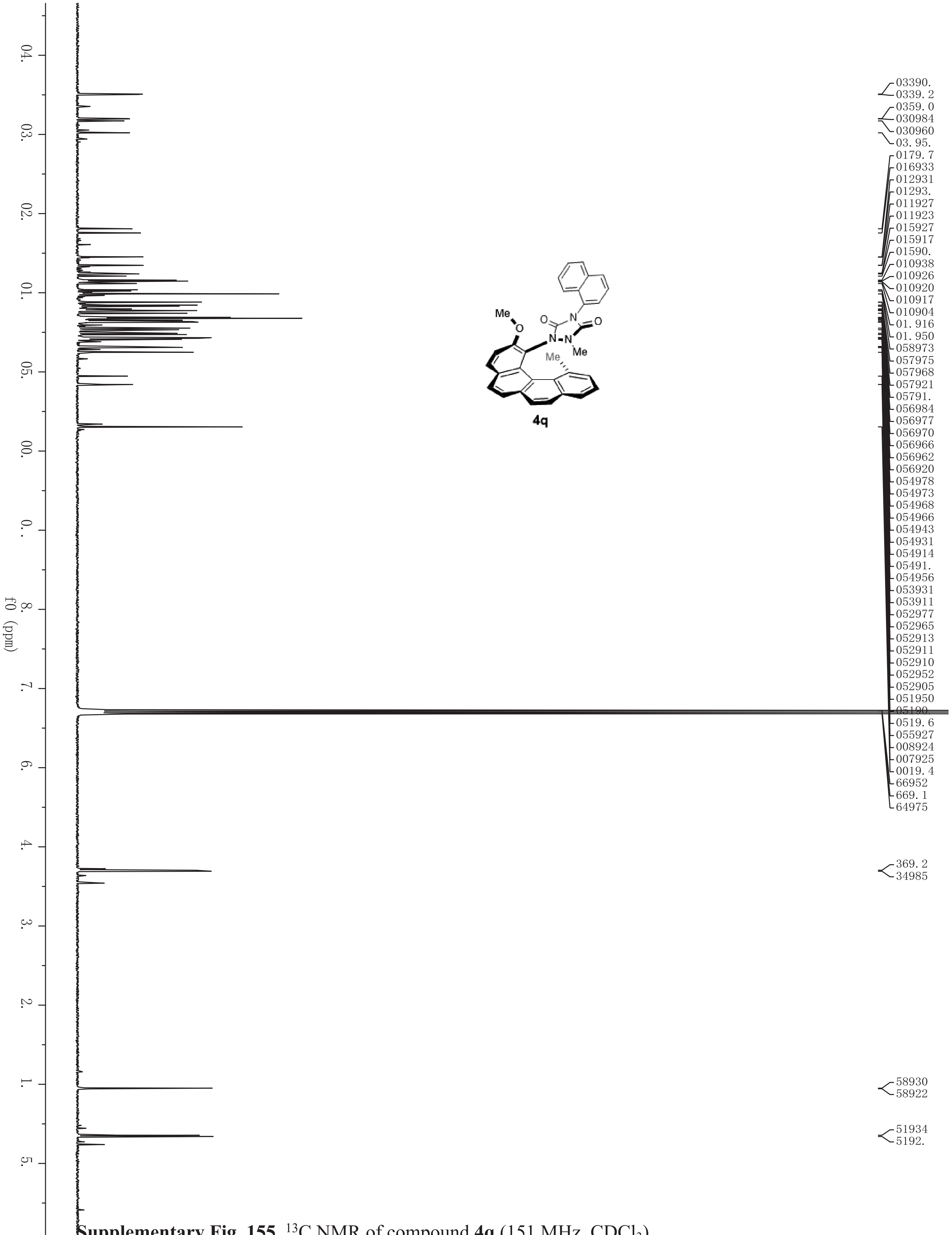

Supplementary Fig. 155. ^{13}C NMR of compound **4q** (151 MHz, $CDCl_3$)

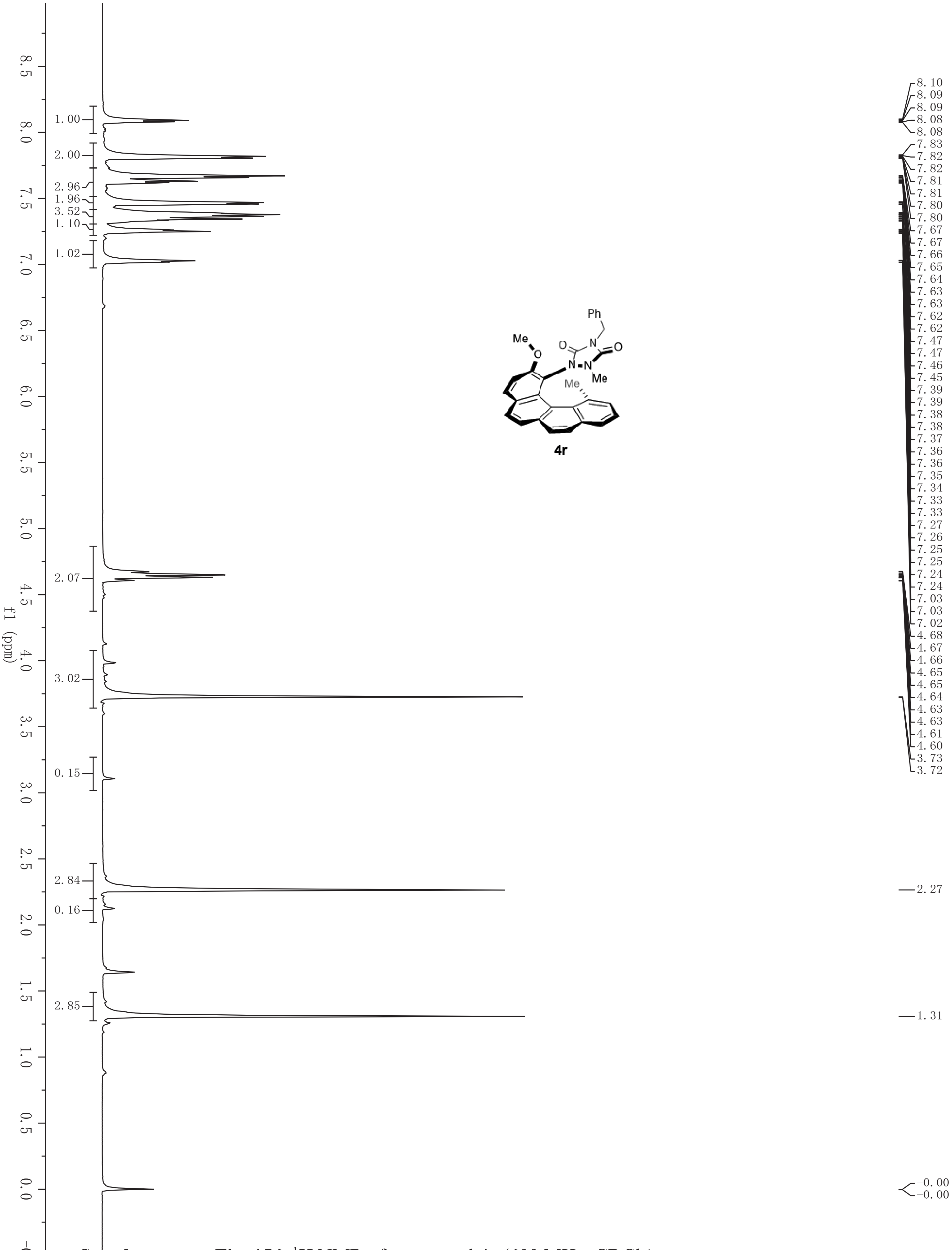

Supplementary Fig. 156. ^{1}H NMR of compound **4r** (600 MHz, $CDCl_3$)

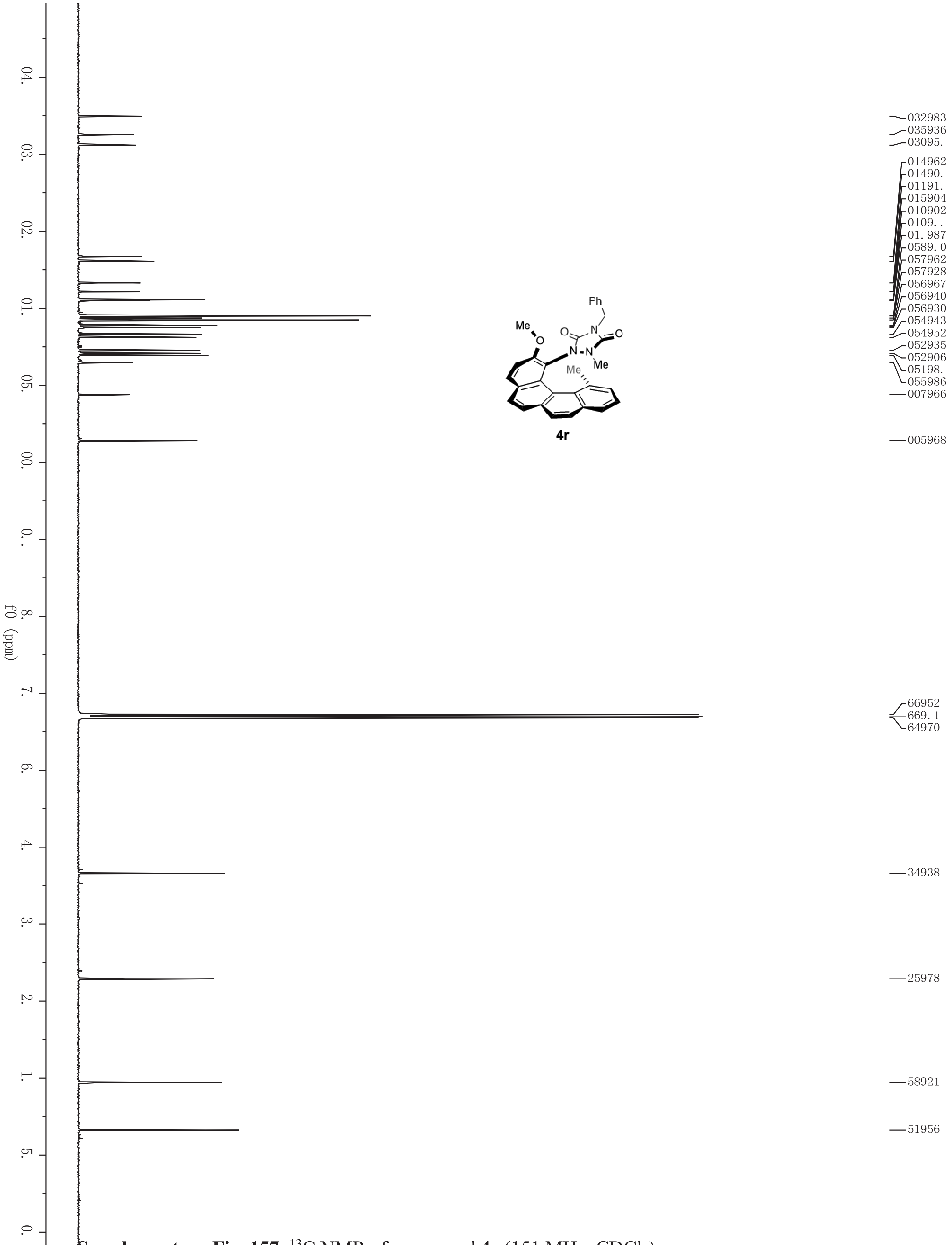

Supplementary Fig. 157. ^{13}C NMR of compound **4r** (151 MHz, $CDCl_3$)

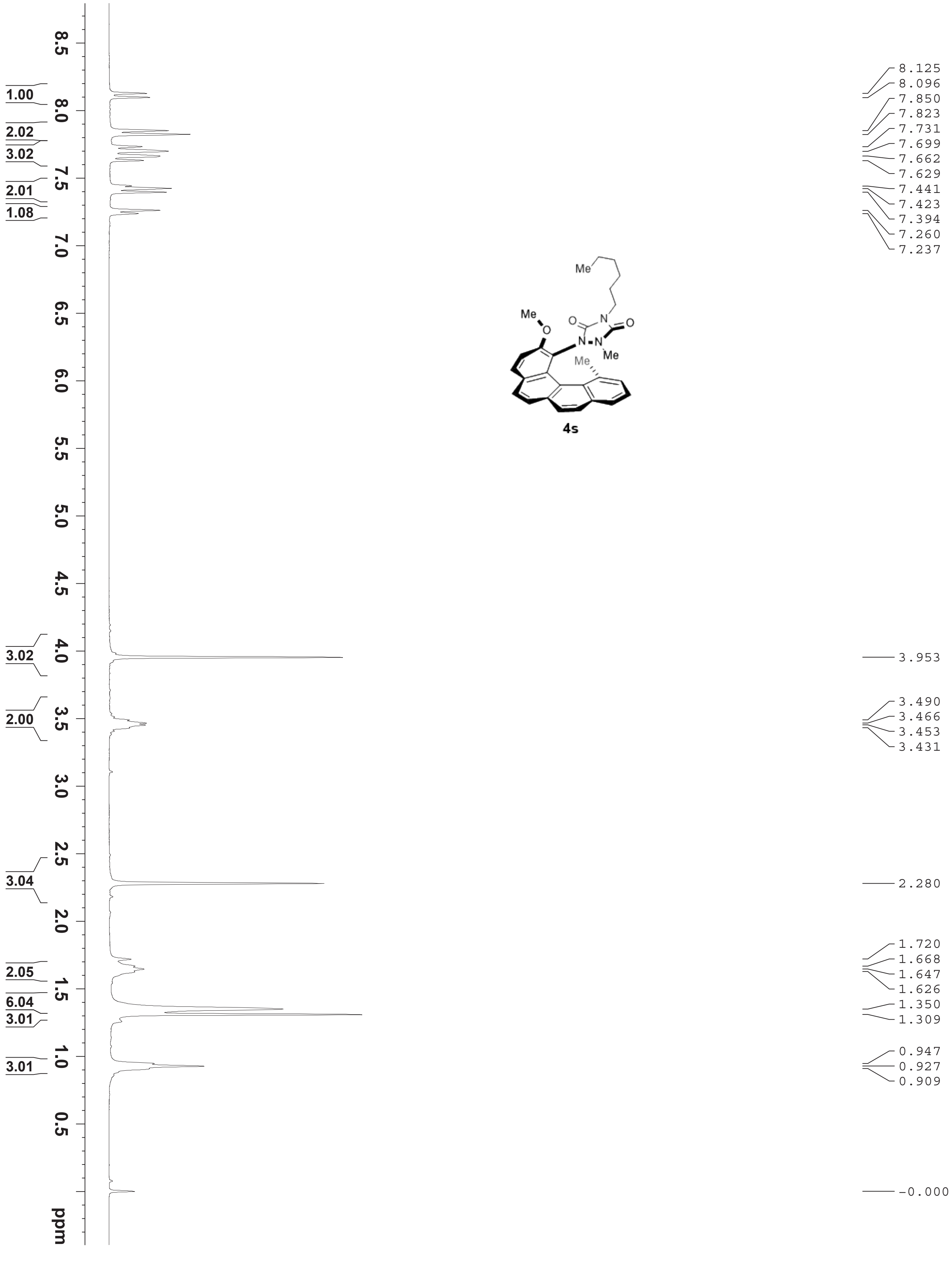

Supplementary Fig. 158. ^{1}H NMR of compound **4s** (300 MHz, $CDCl_3$)

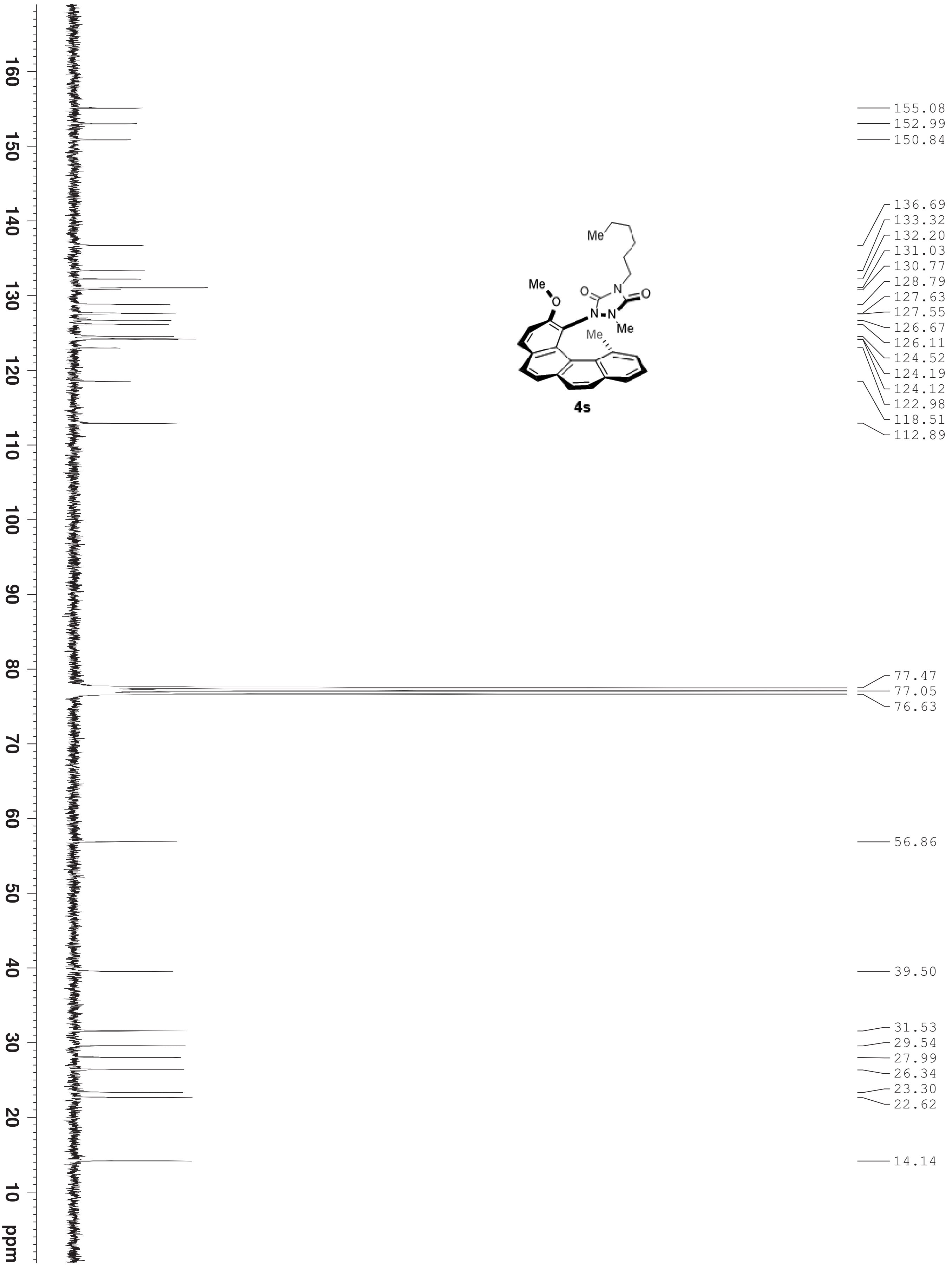

Supplementary Fig. 159. ^{13}C NMR of compound **4s** (75 MHz, $CDCl_3$)

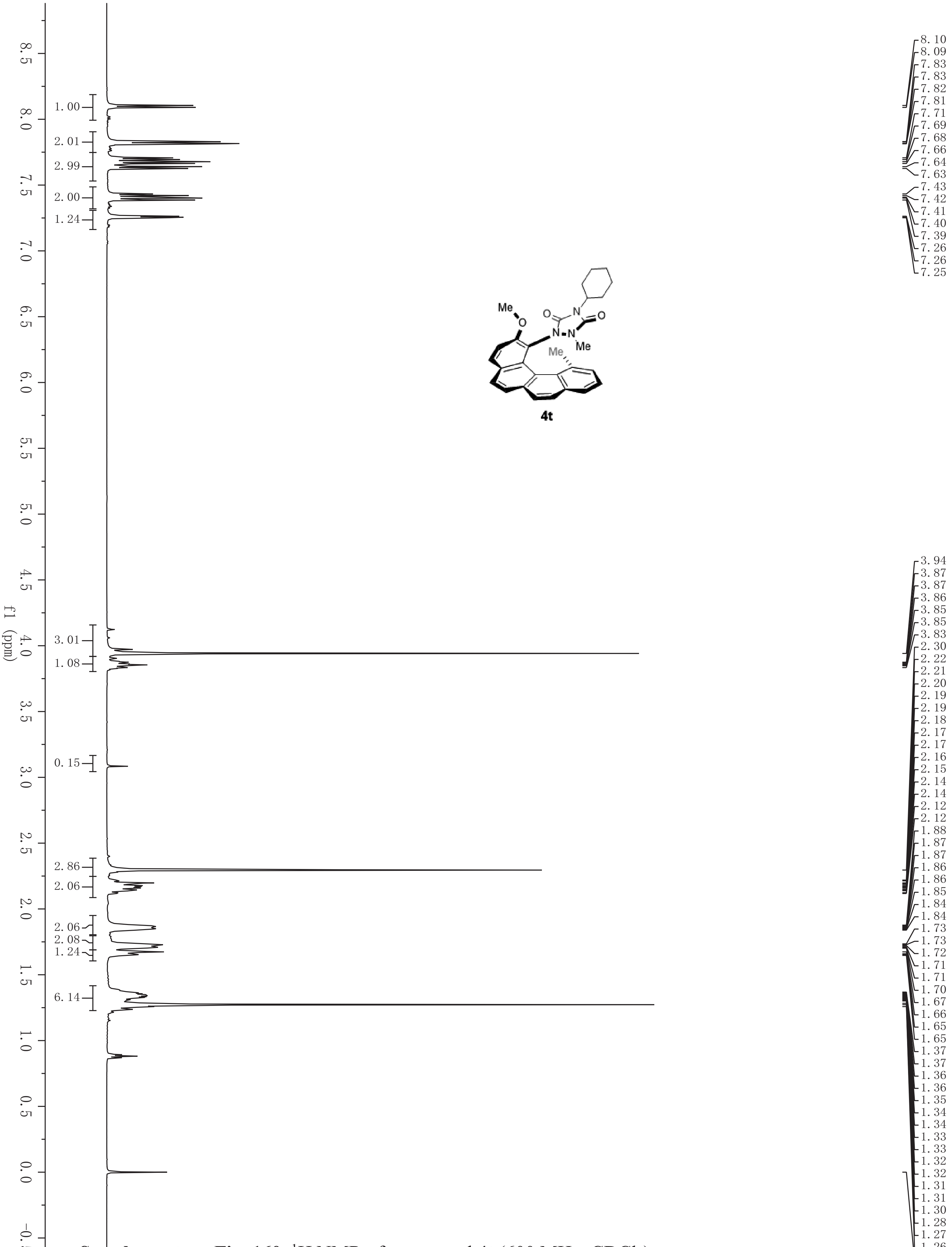

Supplementary Fig. 160. ^{1}H NMR of compound **4t** (600 MHz, $CDCl_3$)

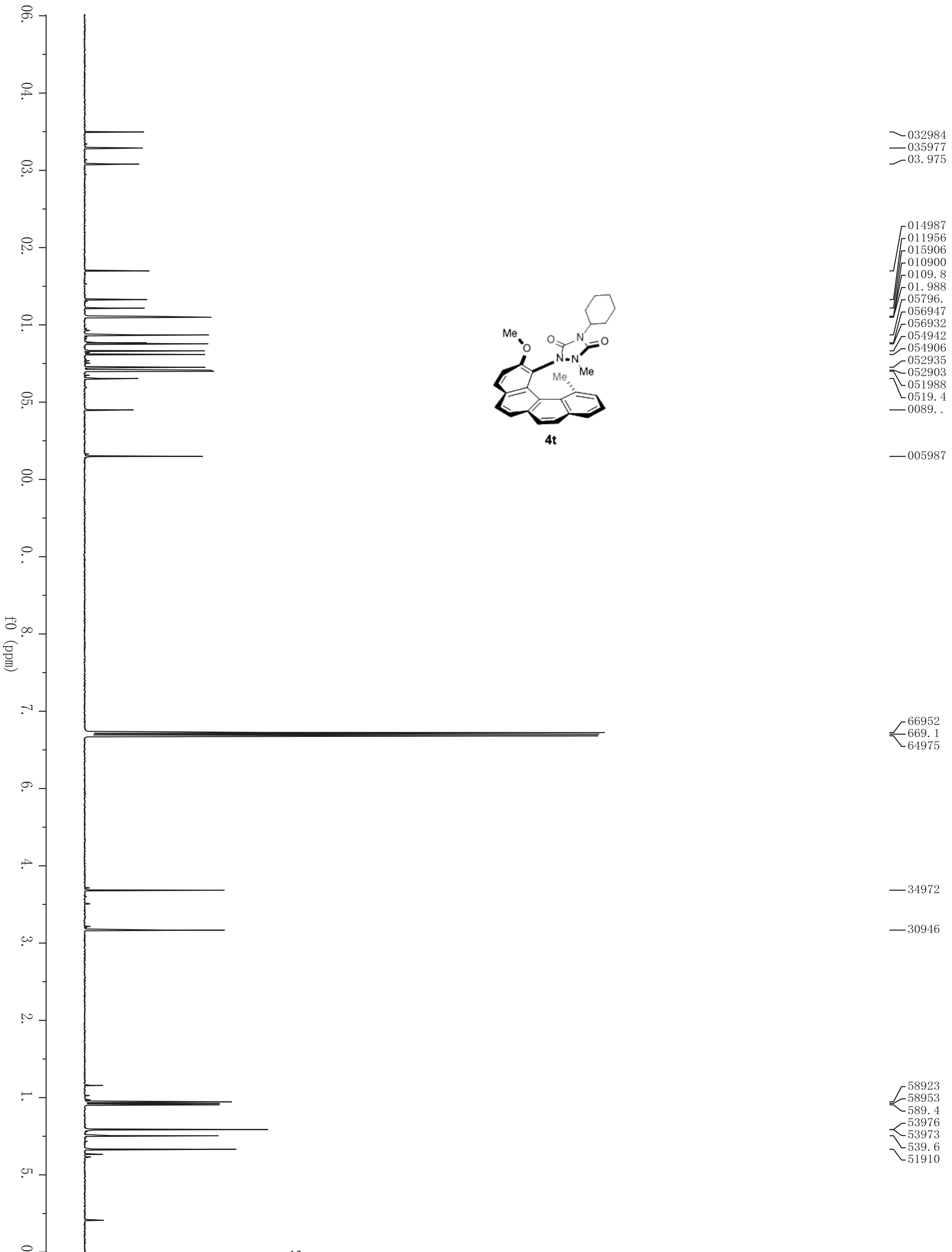

Supplementary Fig. 161. ^{13}C NMR of compound **4t** (151 MHz, $CDCl_3$)

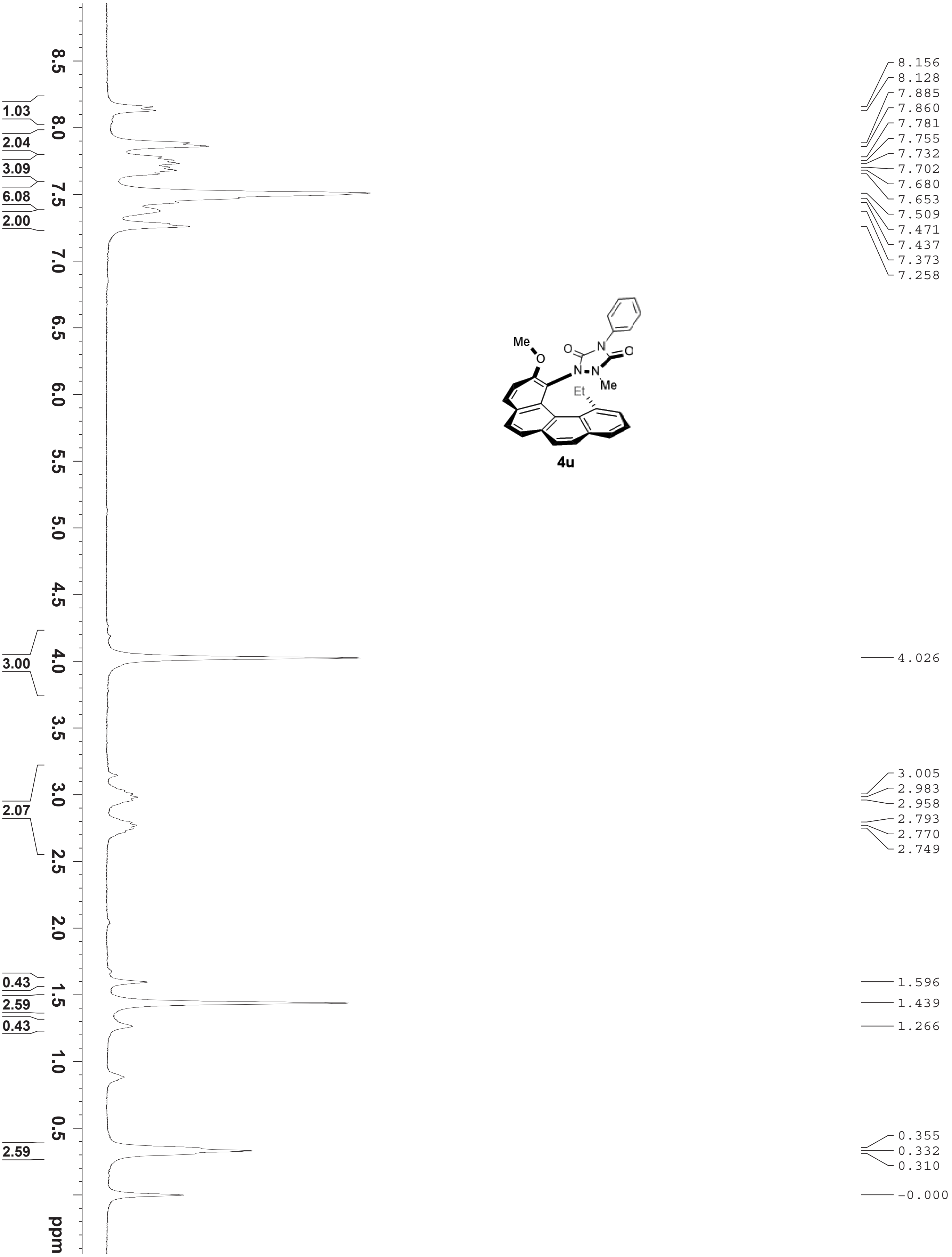

Supplementary Fig. 162. ^{1}H NMR of compound **4u** (300 MHz, $CDCl_3$)

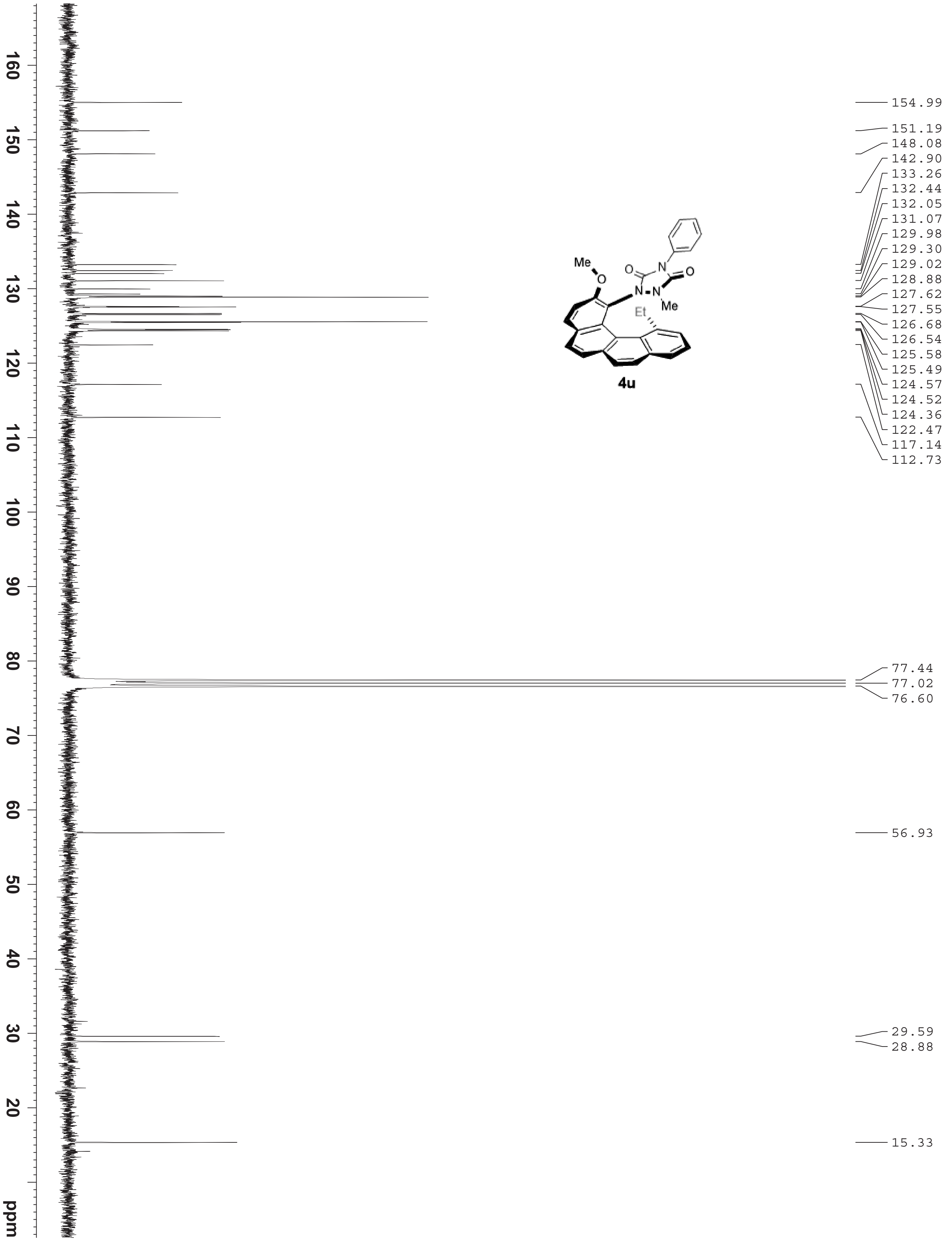

Supplementary Fig. 163. ^{13}C NMR of compound **4u** (75 MHz, $CDCl_3$)

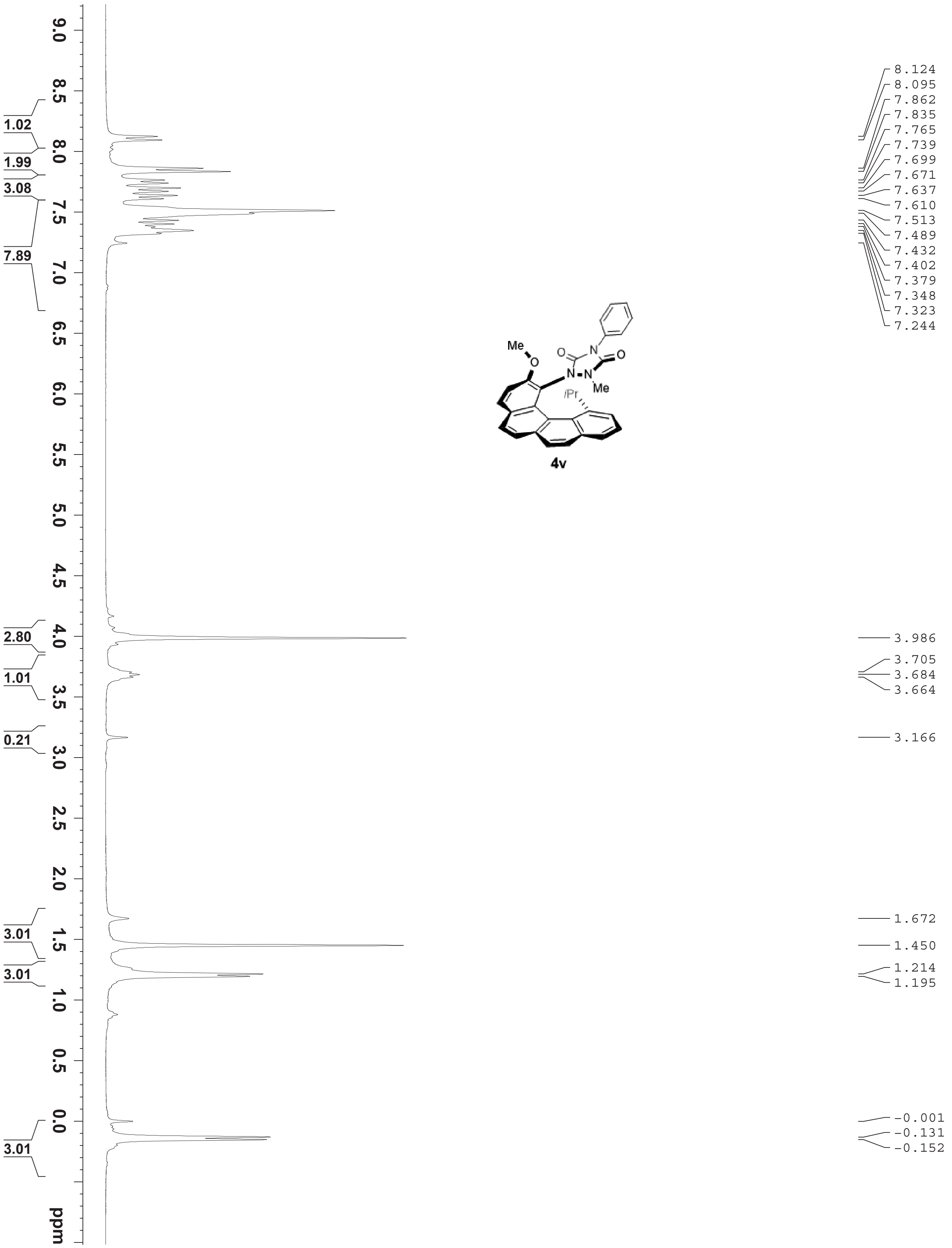

Supplementary Fig. 164. ^{1}H NMR of compound **4v** (300 MHz, $CDCl_3$)

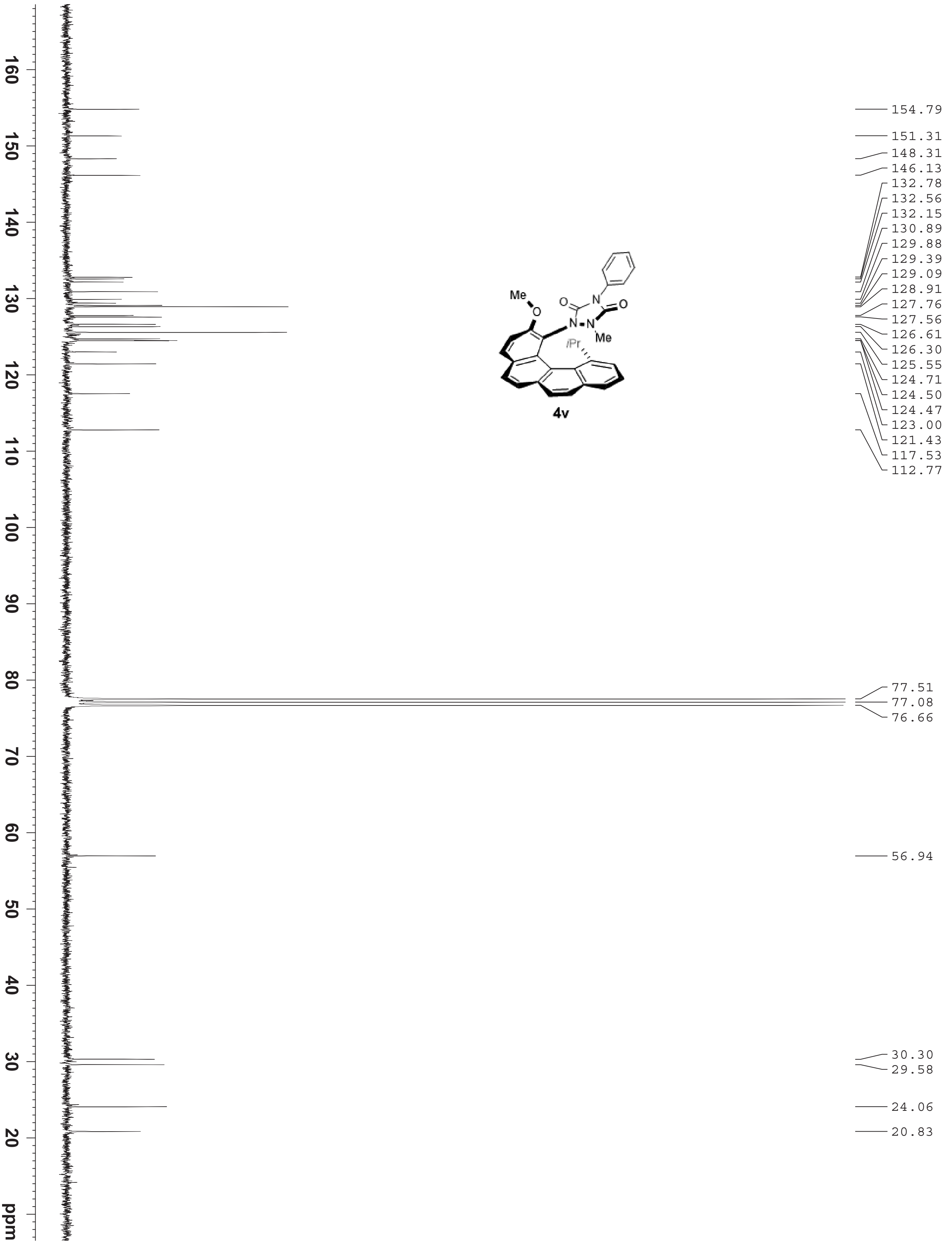

Supplementary Fig. 165. ^{13}C NMR of compound **4v** (75 MHz, $CDCl_3$)

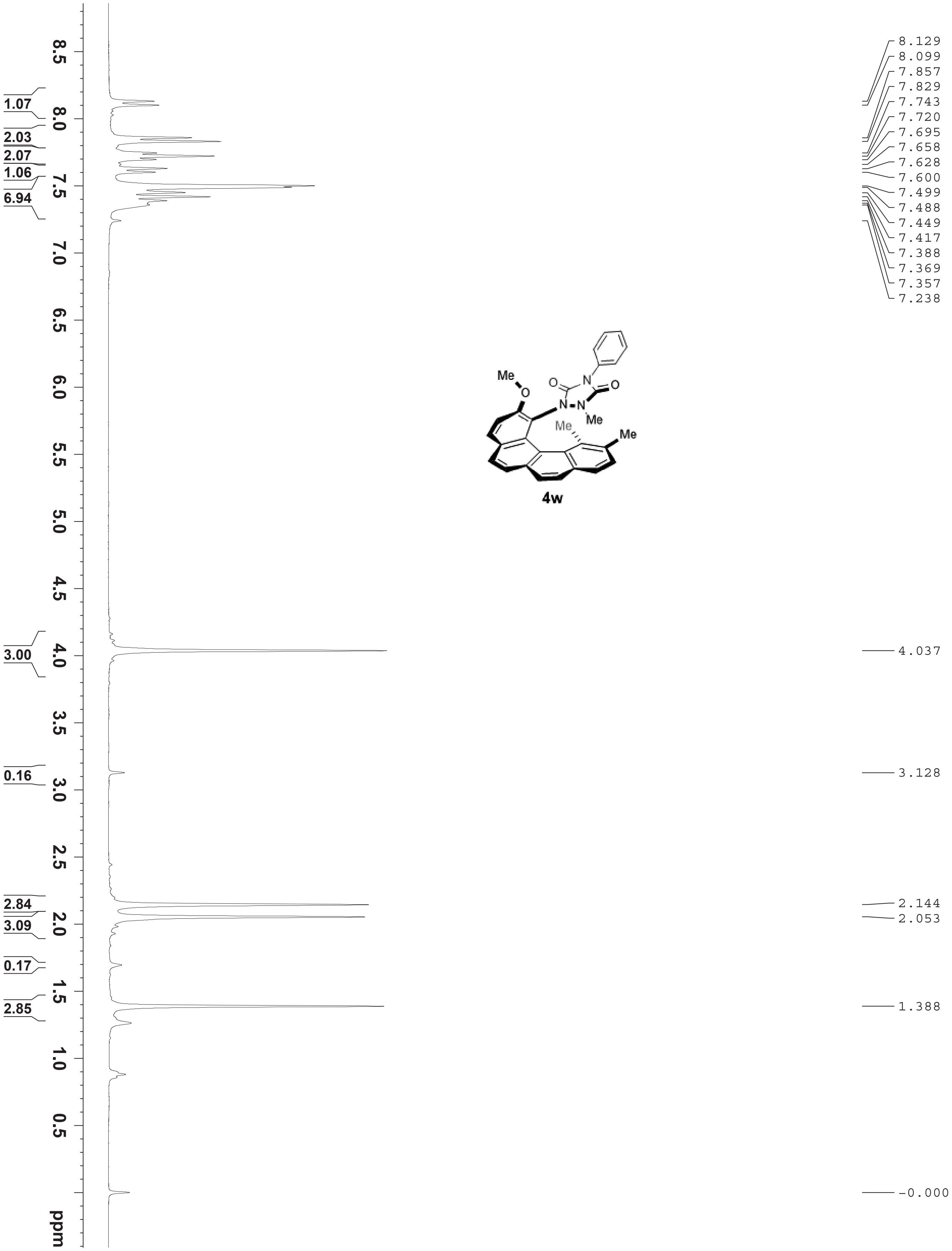

Supplementary Fig. 166. ^{1}H NMR of compound **4w** (300 MHz, $CDCl_3$)

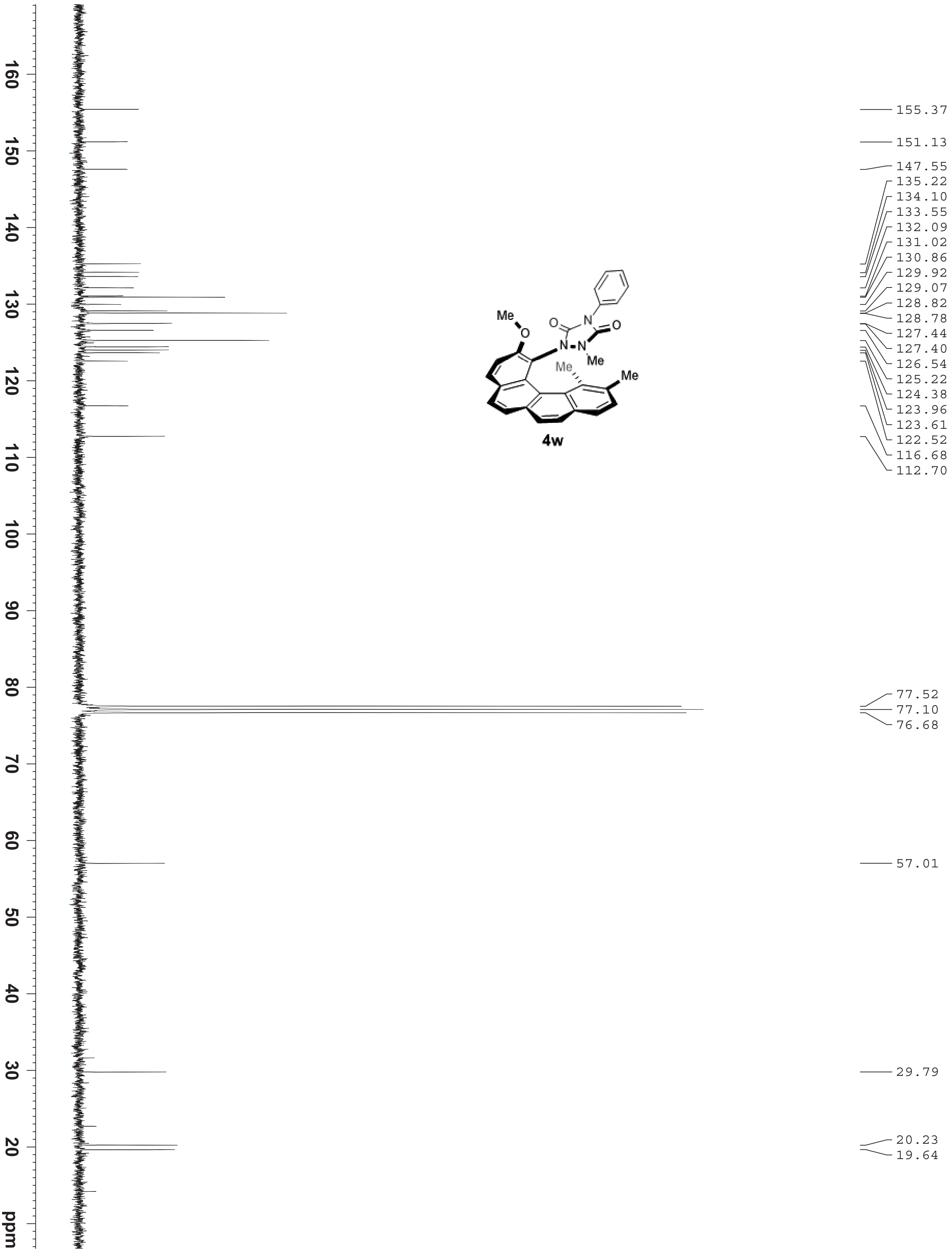

Supplementary Fig. 167. ^{13}C NMR of compound **4w** (75 MHz, $CDCl_3$)

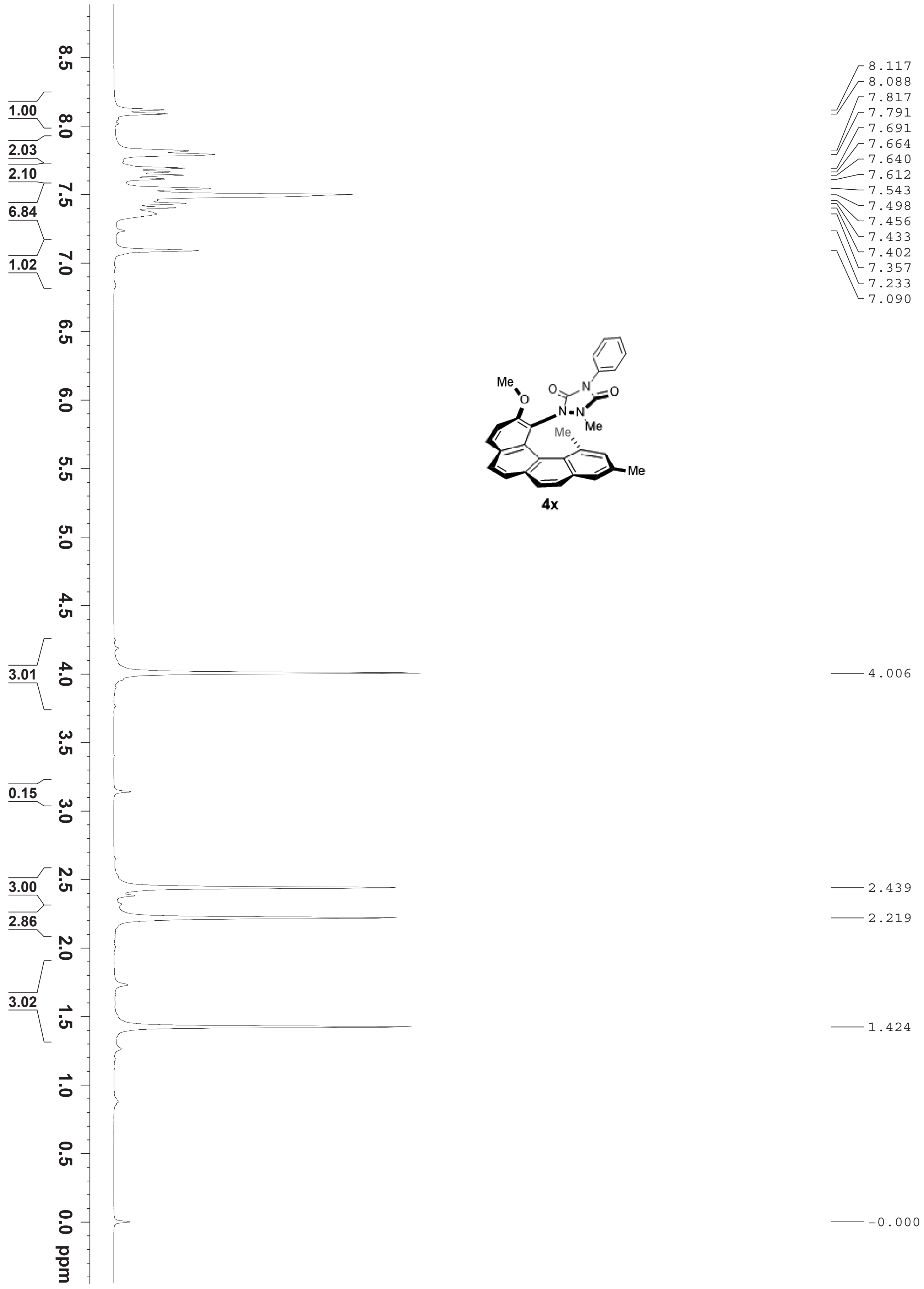

Supplementary Fig. 168. ^{1}H NMR of compound **4x** (300 MHz, $CDCl_3$)

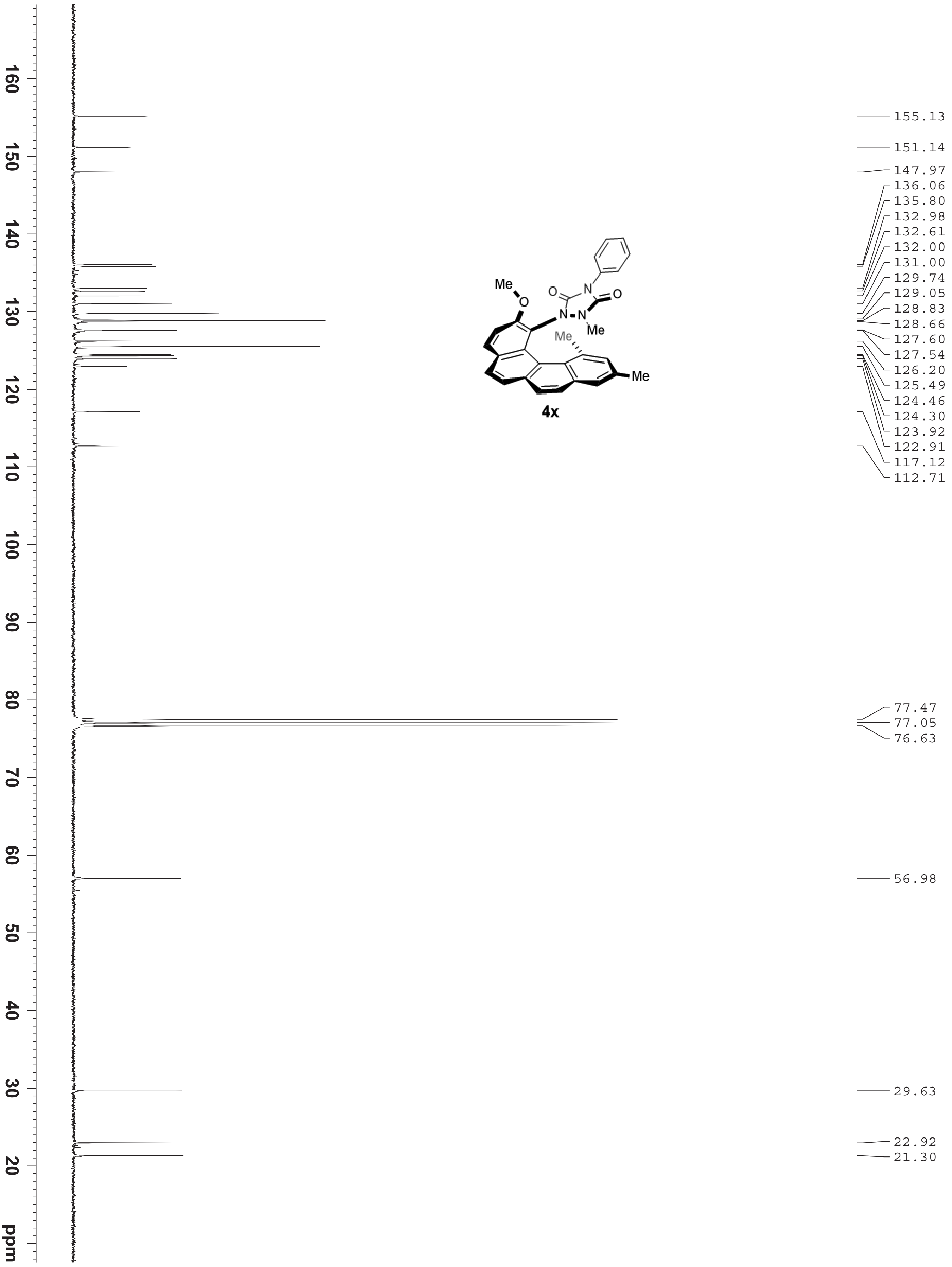

Supplementary Fig. 169. ^{13}C NMR of compound **4x** (75 MHz, $CDCl_3$)

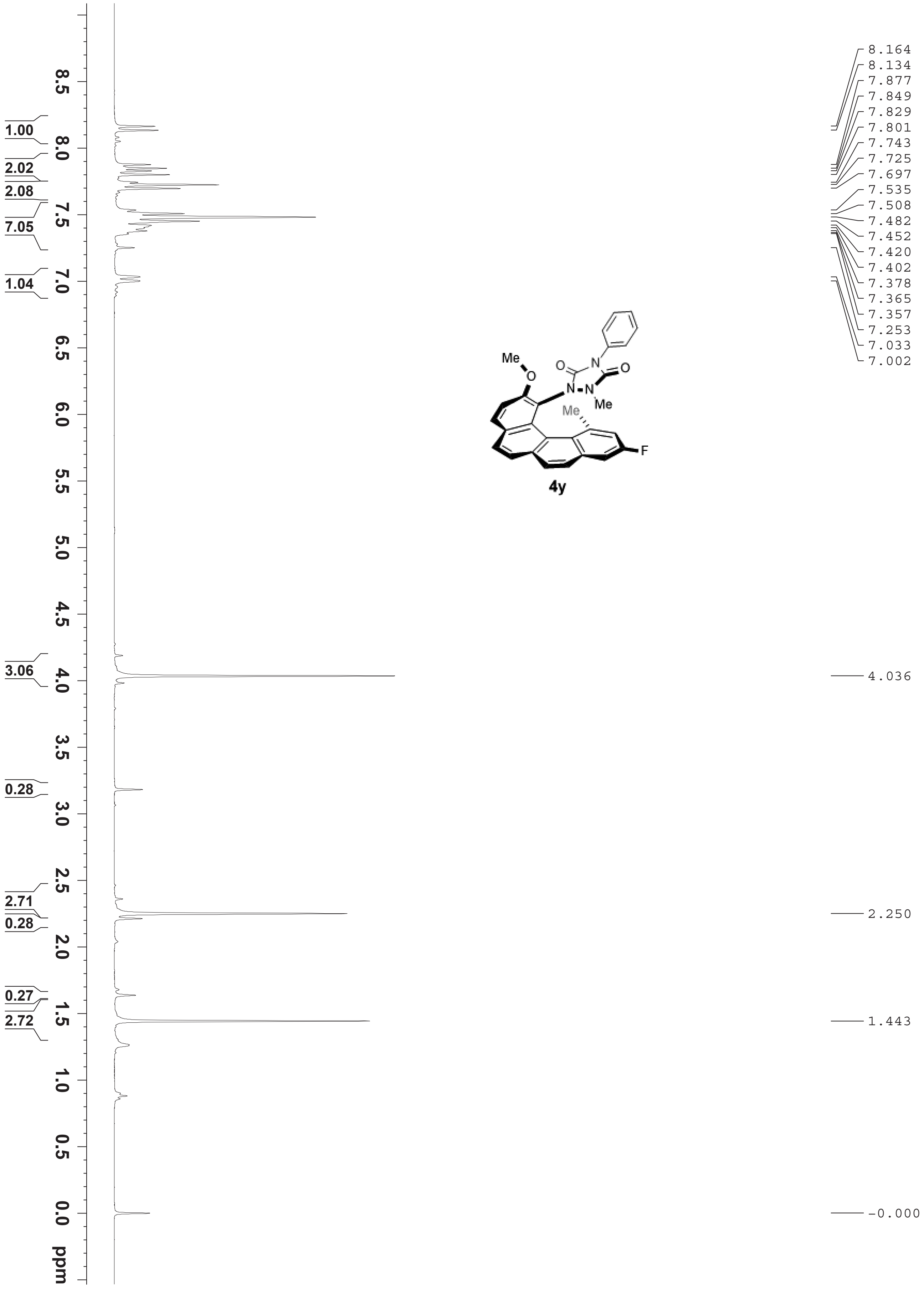

Supplementary Fig. 170. ^{1}H NMR of compound **4y** (300 MHz, $CDCl_3$)

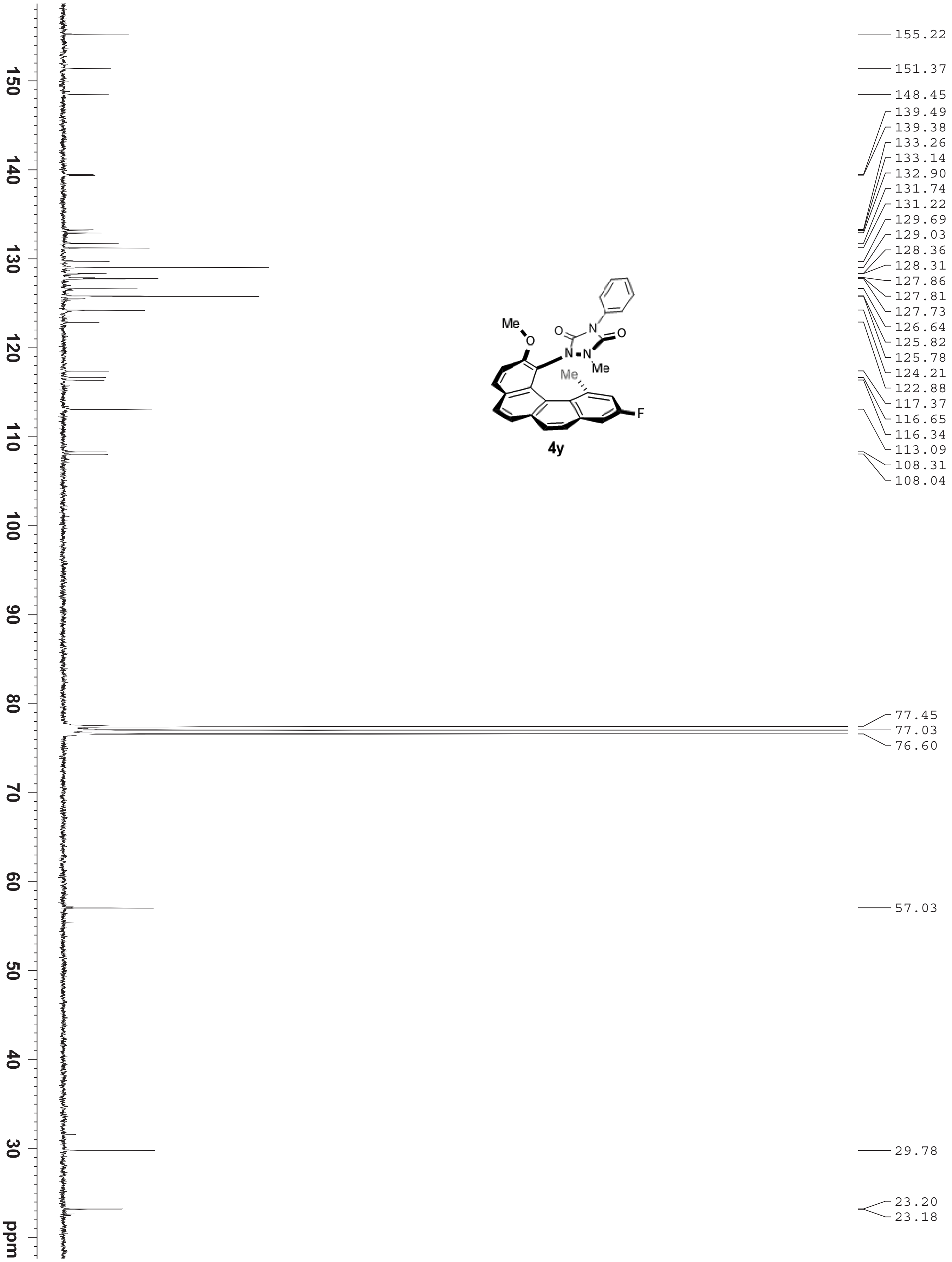

Supplementary Fig. 171. ^{13}C NMR of compound **4y** (75 MHz, $CDCl_3$)

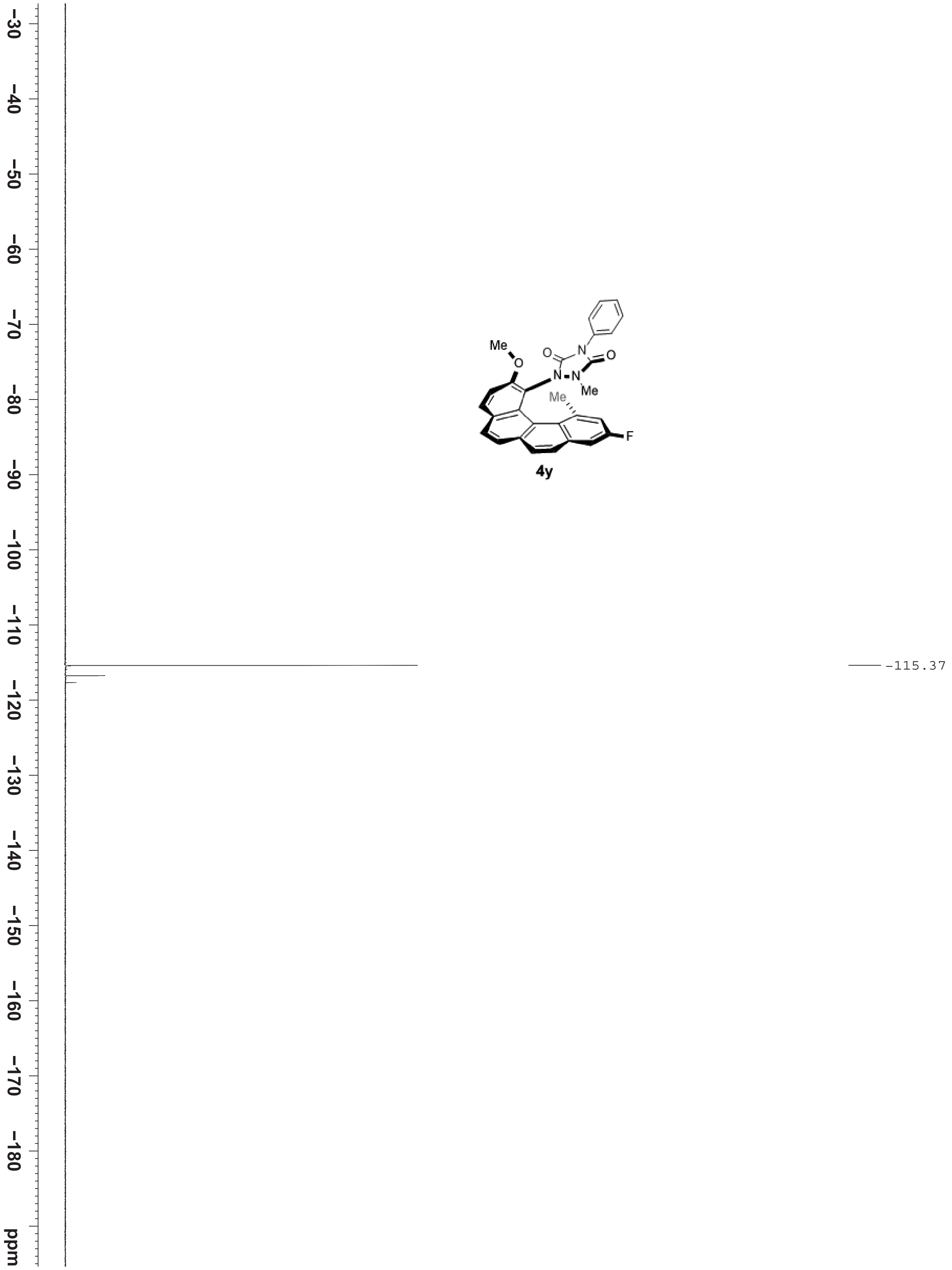

Supplementary Fig. 172. ^{19}F NMR of compound **4y** (282 MHz, $CDCl_3$)

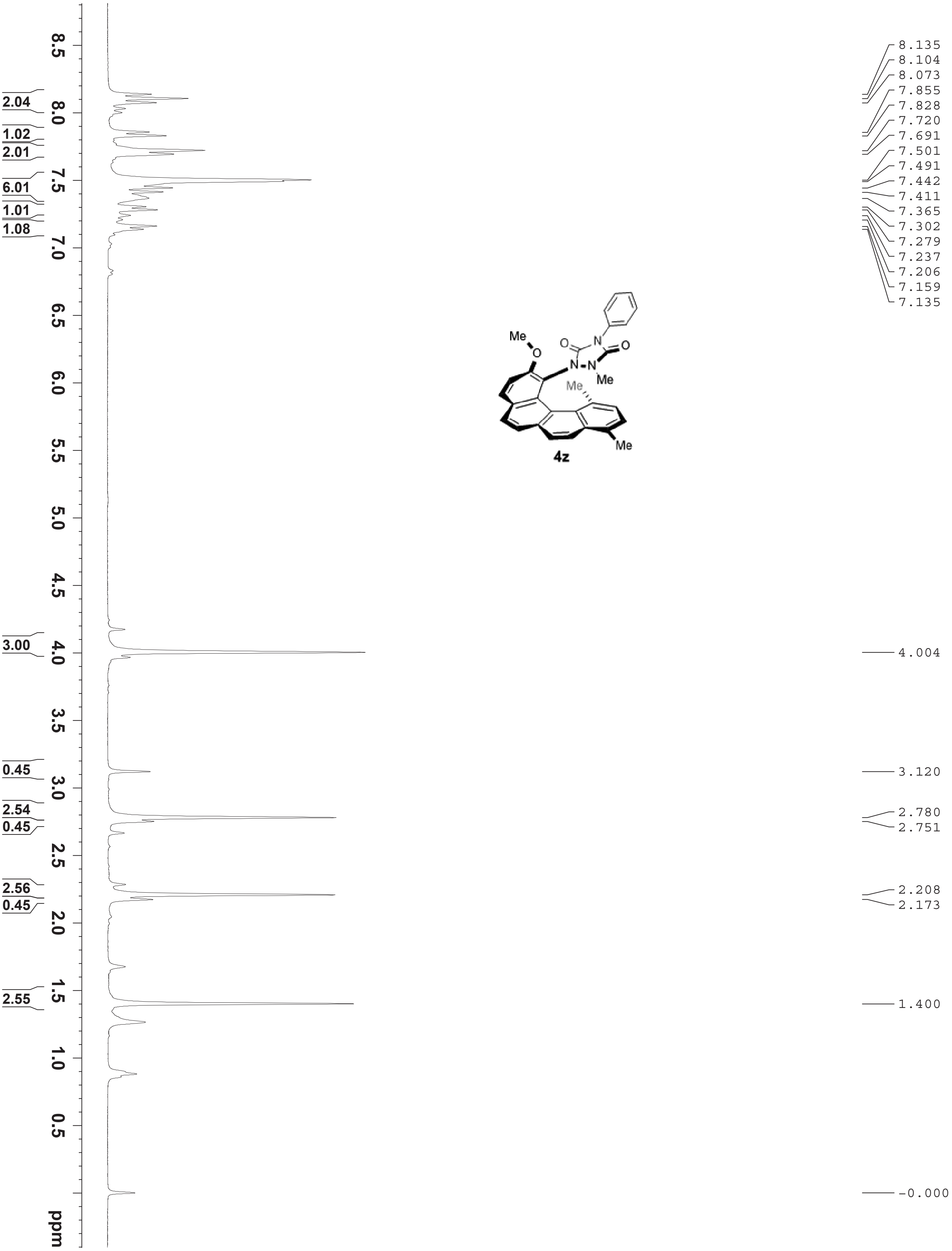

Supplementary Fig. 173. ^{1}H NMR of compound **4z** (300 MHz, $CDCl_3$)

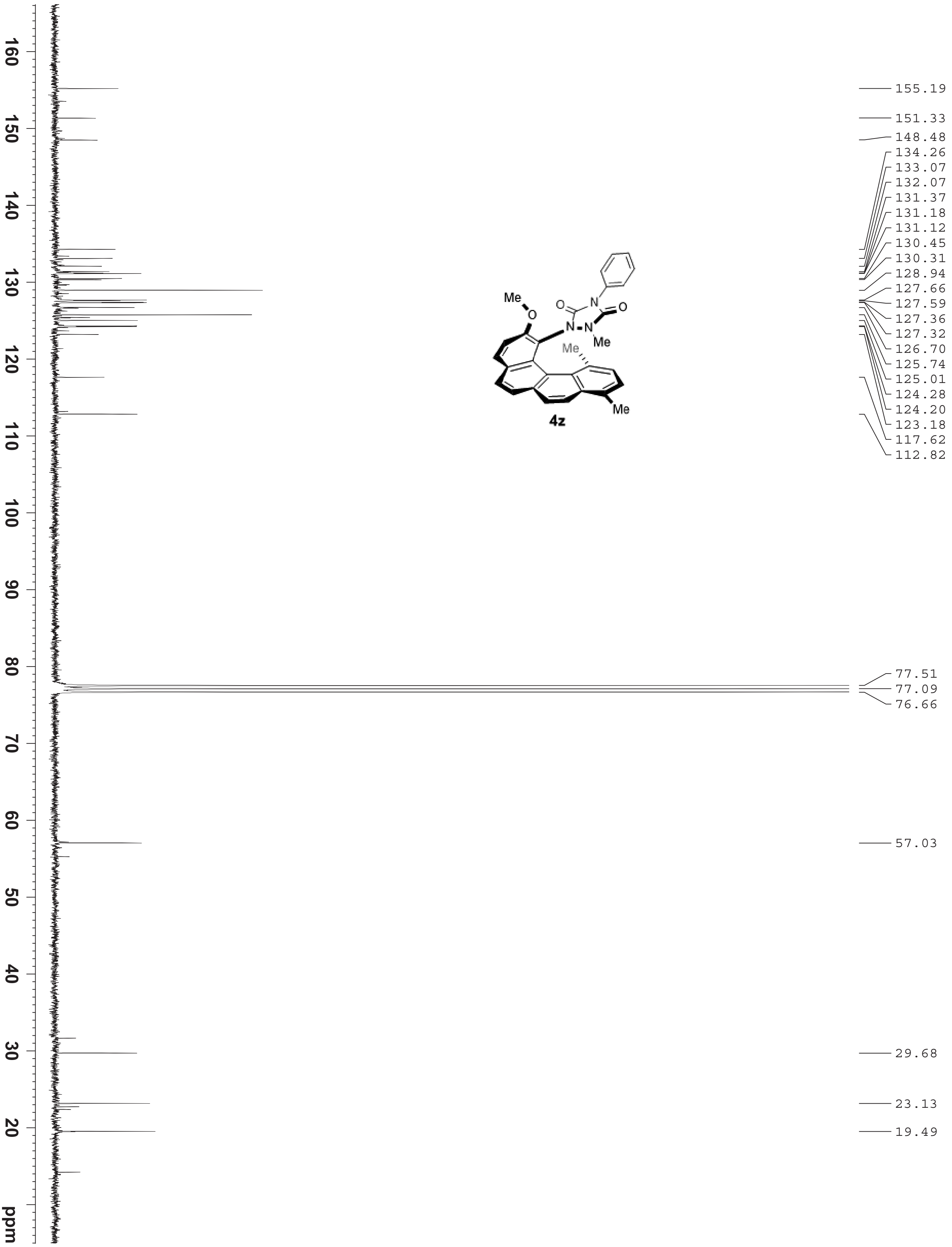

Supplementary Fig. 174. ^{13}C NMR of compound **4z** (75 MHz, $CDCl_3$)

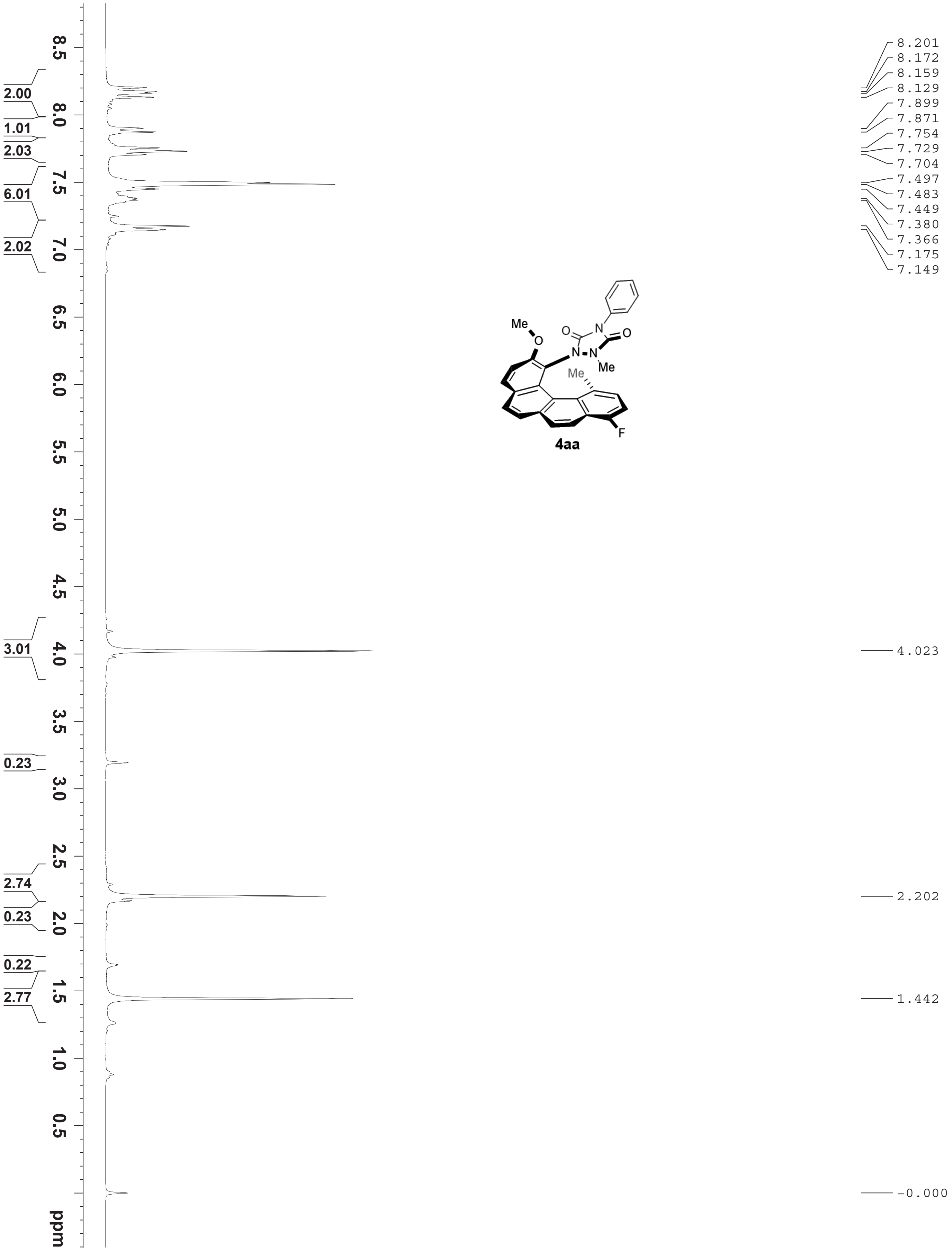

Supplementary Fig. 175. ^{1}H NMR of compound **4aa** (300 MHz, $CDCl_3$)

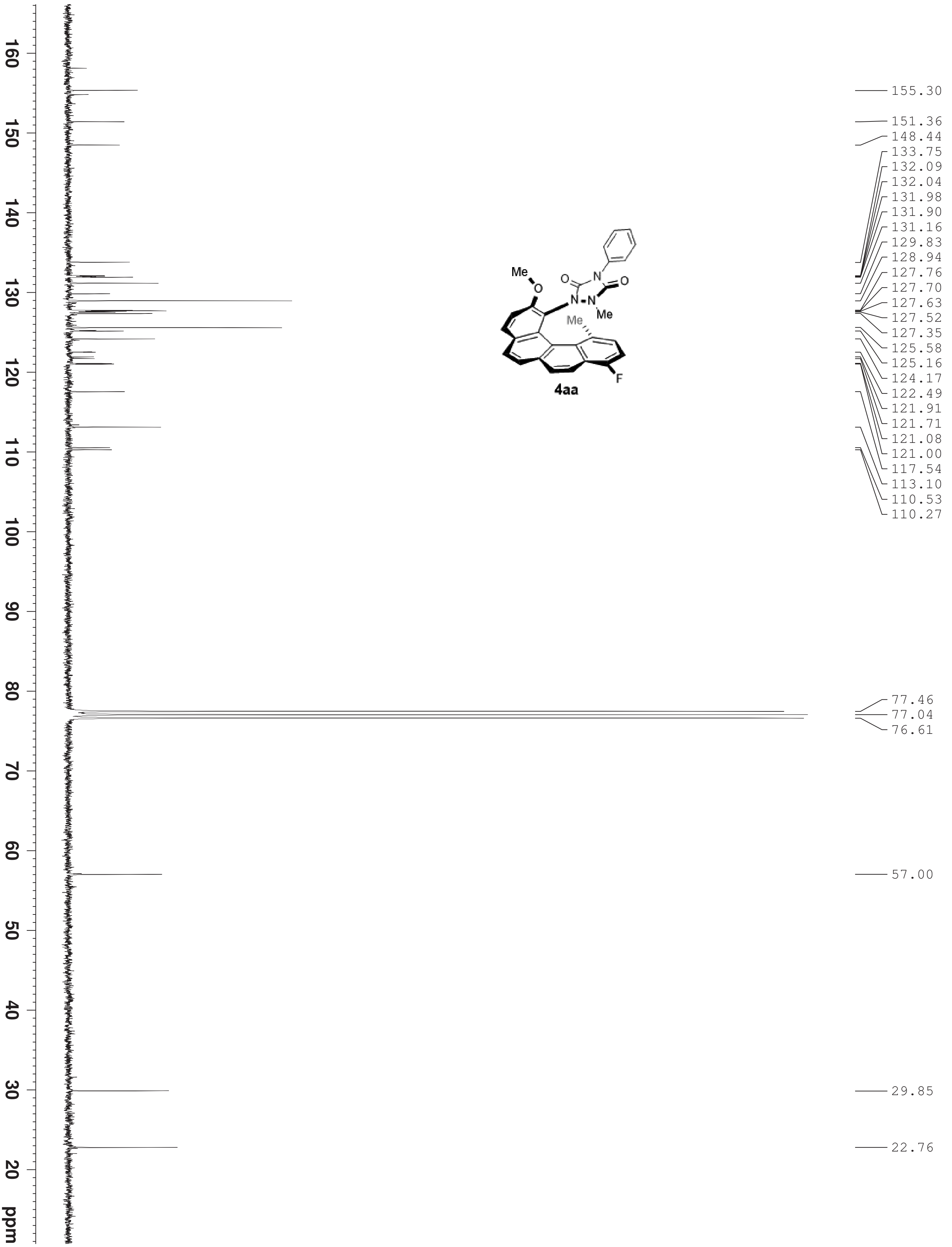

Supplementary Fig. 176. ^{13}C NMR of compound **4aa** (75 MHz, $CDCl_3$)

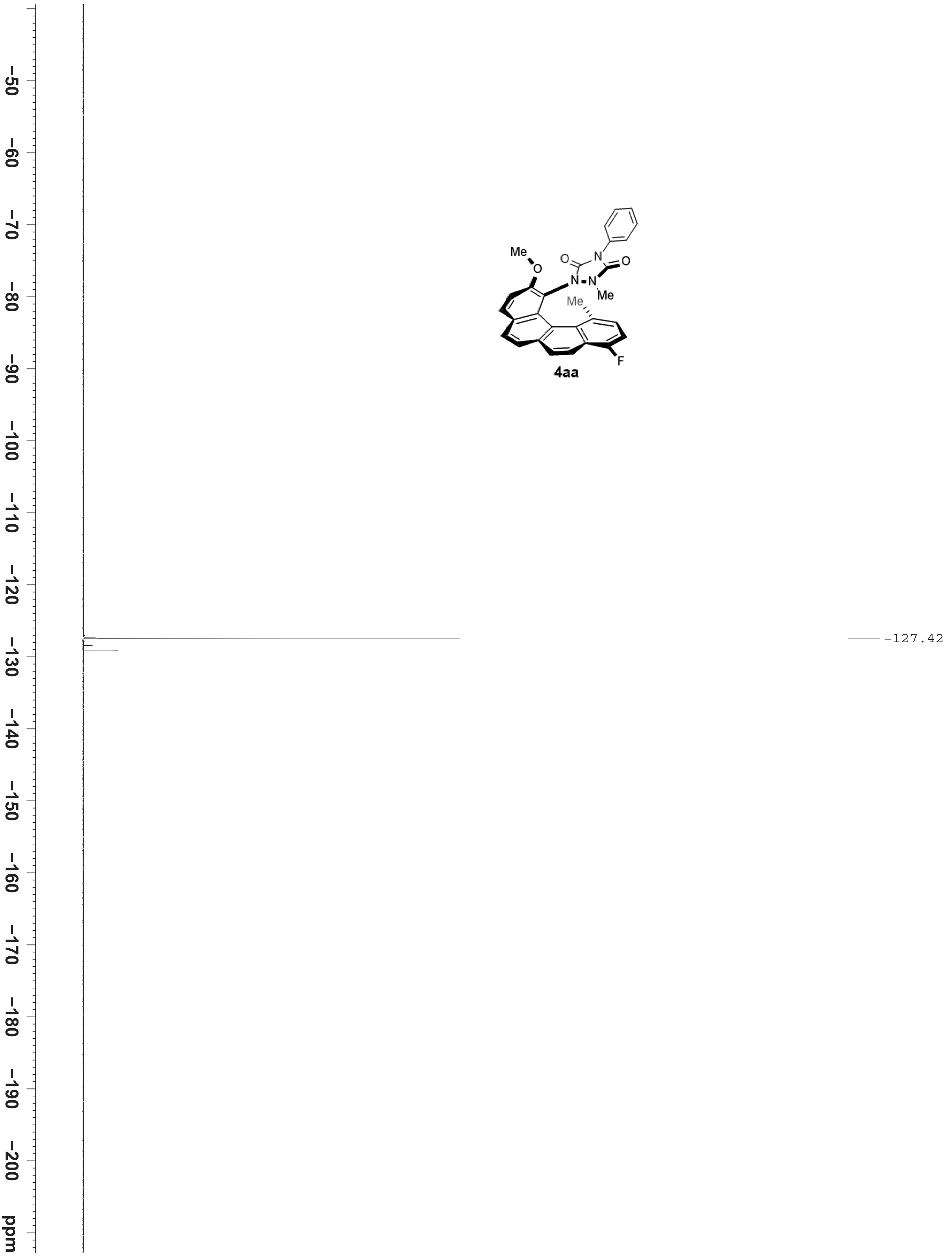

Supplementary Fig. 177. ^{19}F NMR of compound **4aa** (282 MHz, $CDCl_3$)

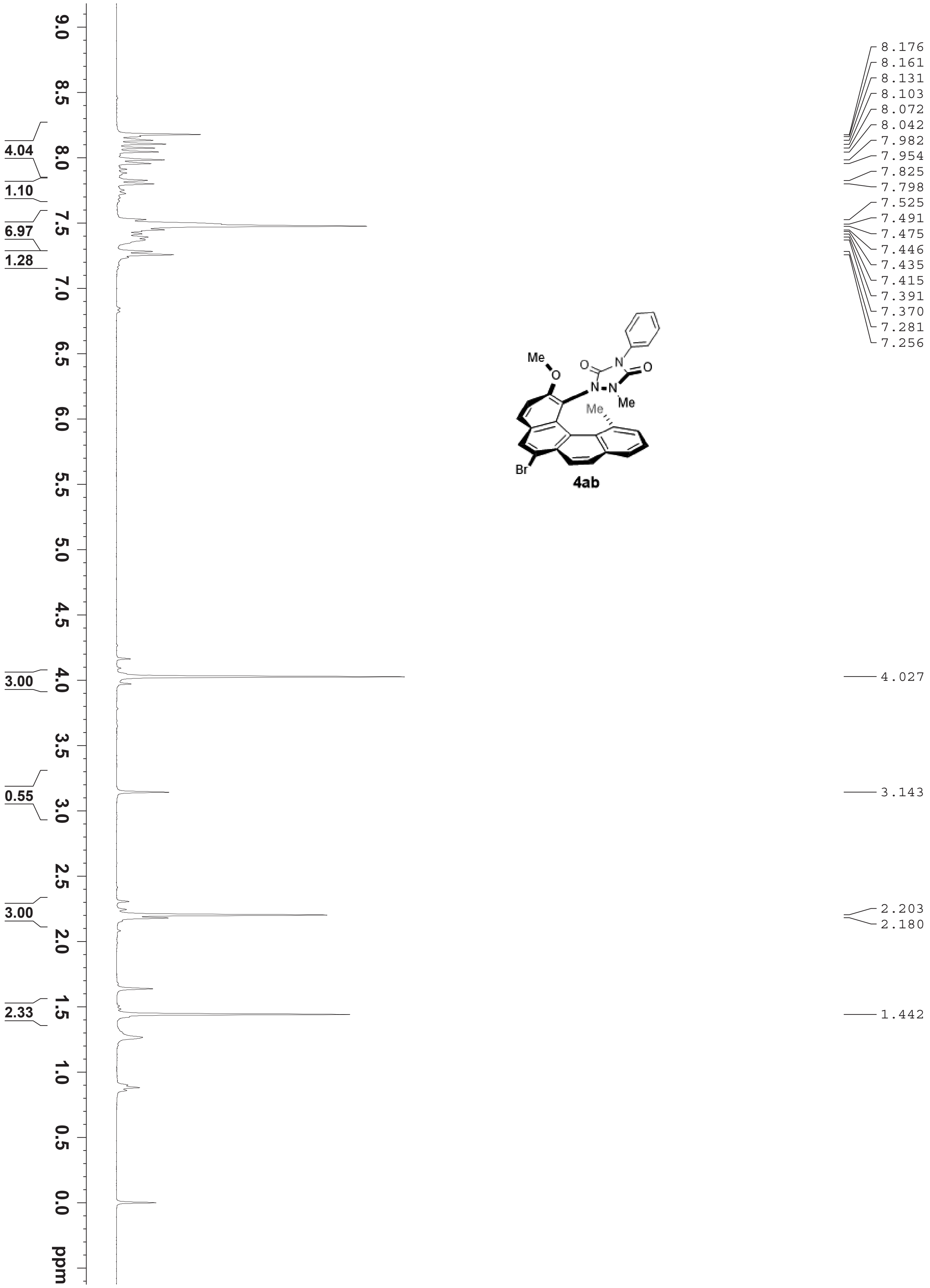

Supplementary Fig. 178. ^{1}H NMR of compound **4ab** (300 MHz, $CDCl_3$)

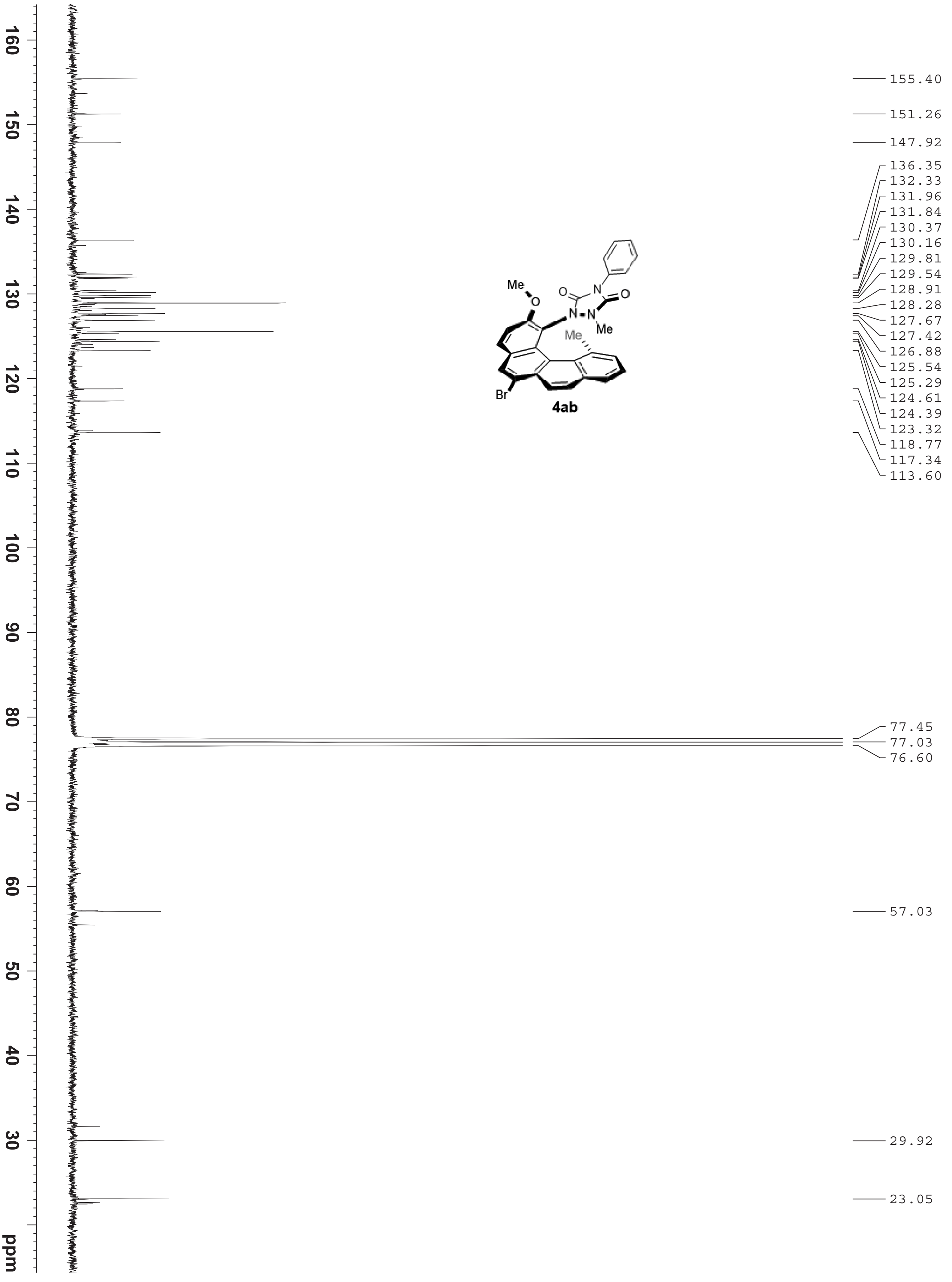

Supplementary Fig. 179. ^{13}C NMR of compound **4ab** (75 MHz, $CDCl_3$)

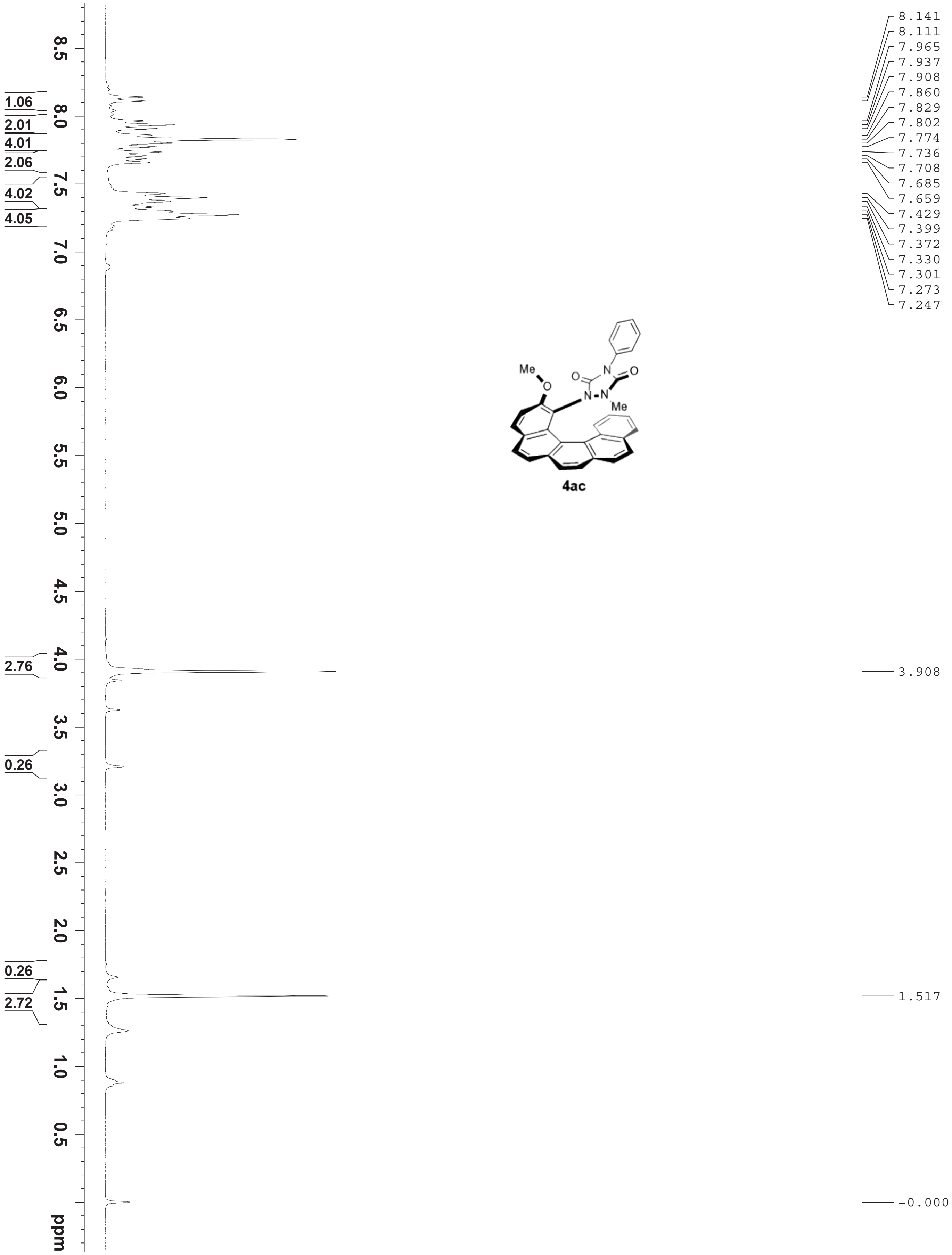

Supplementary Fig. 180. ^{1}H NMR of compound **4ac** (300 MHz, $CDCl_3$)

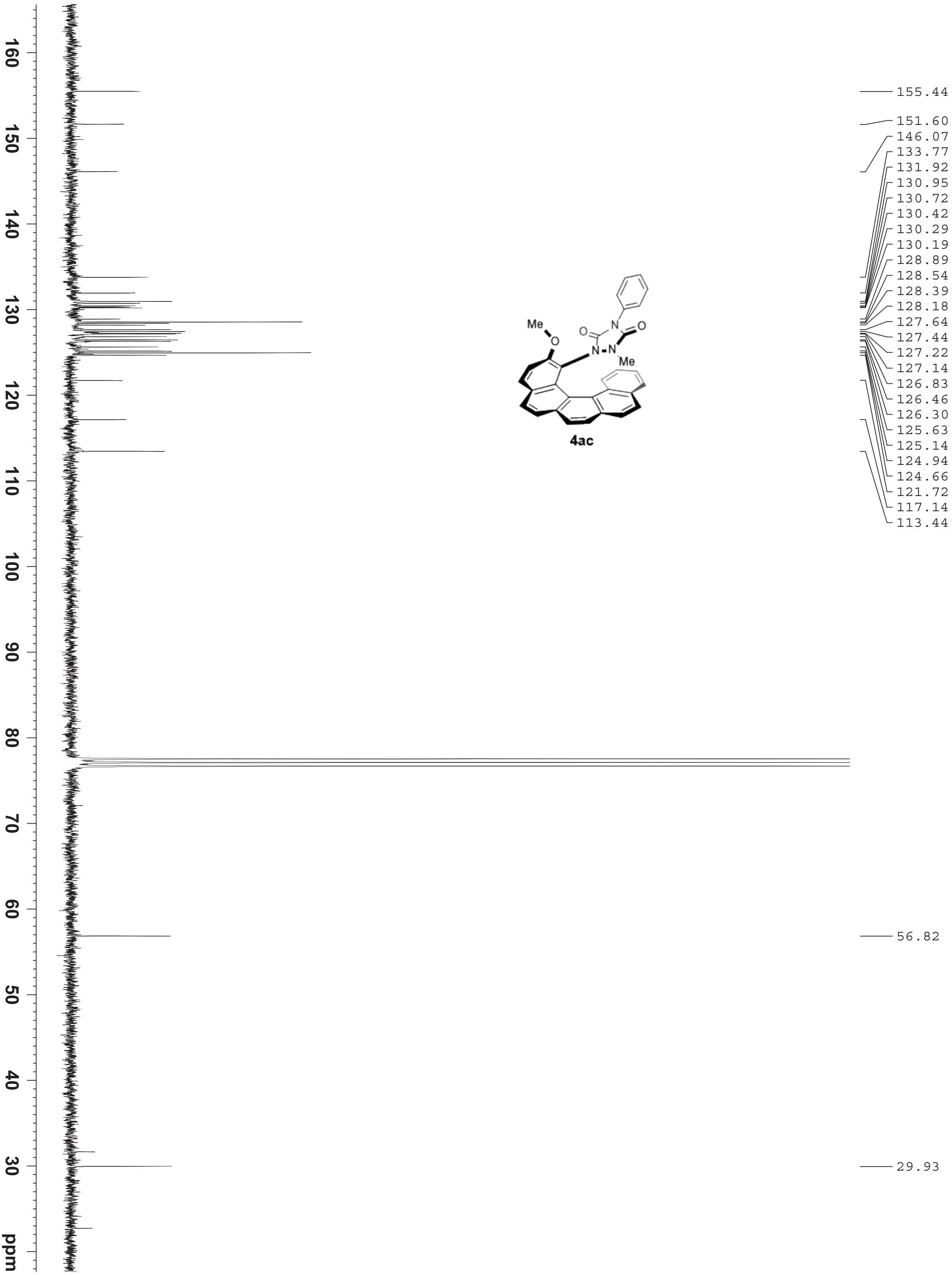

Supplementary Fig. 181. ^{13}C NMR of compound **4ac** (75 MHz, $CDCl_3$)

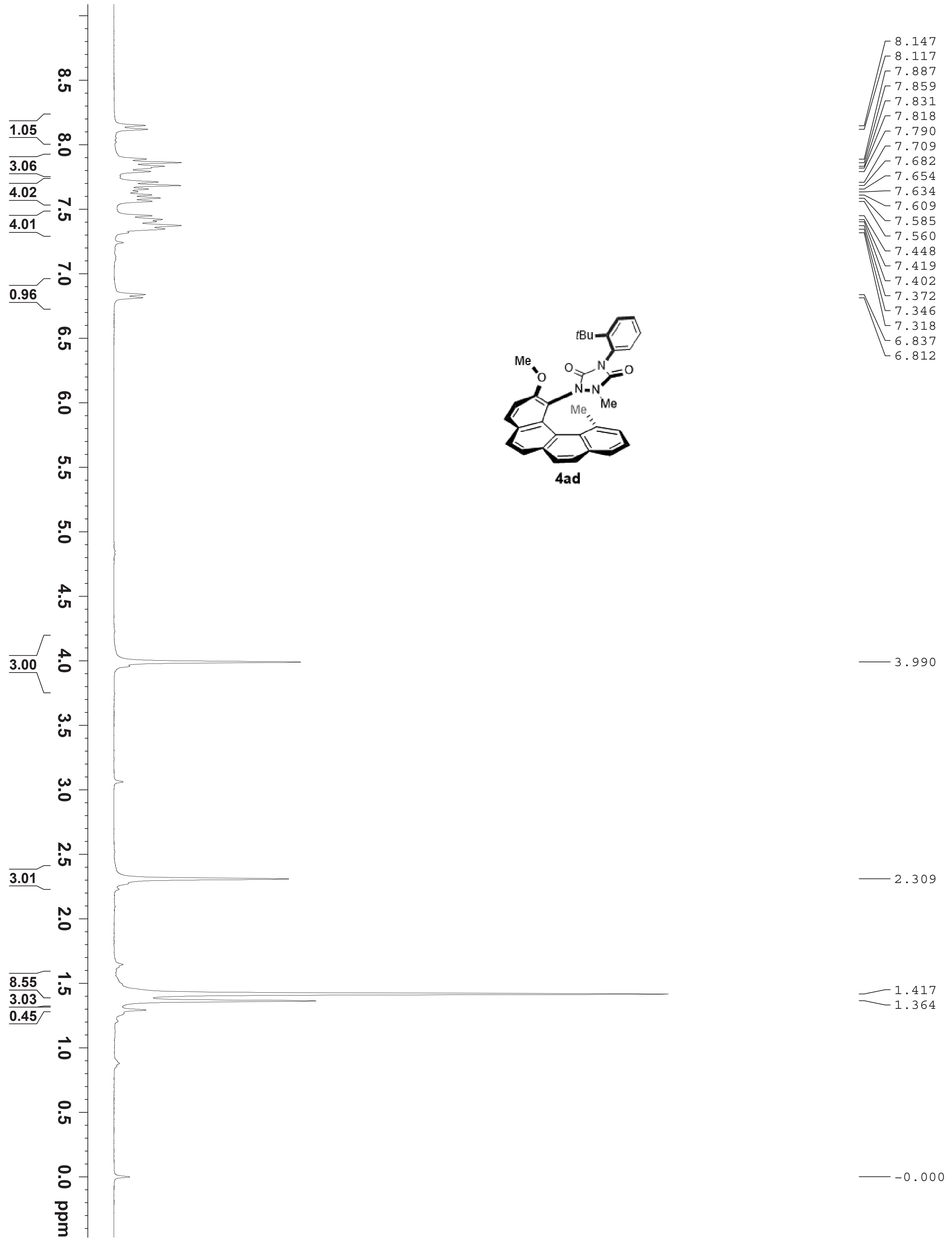

Supplementary Fig. 182. ^{1}H NMR of compound **4ad** (300 MHz, $CDCl_3$)

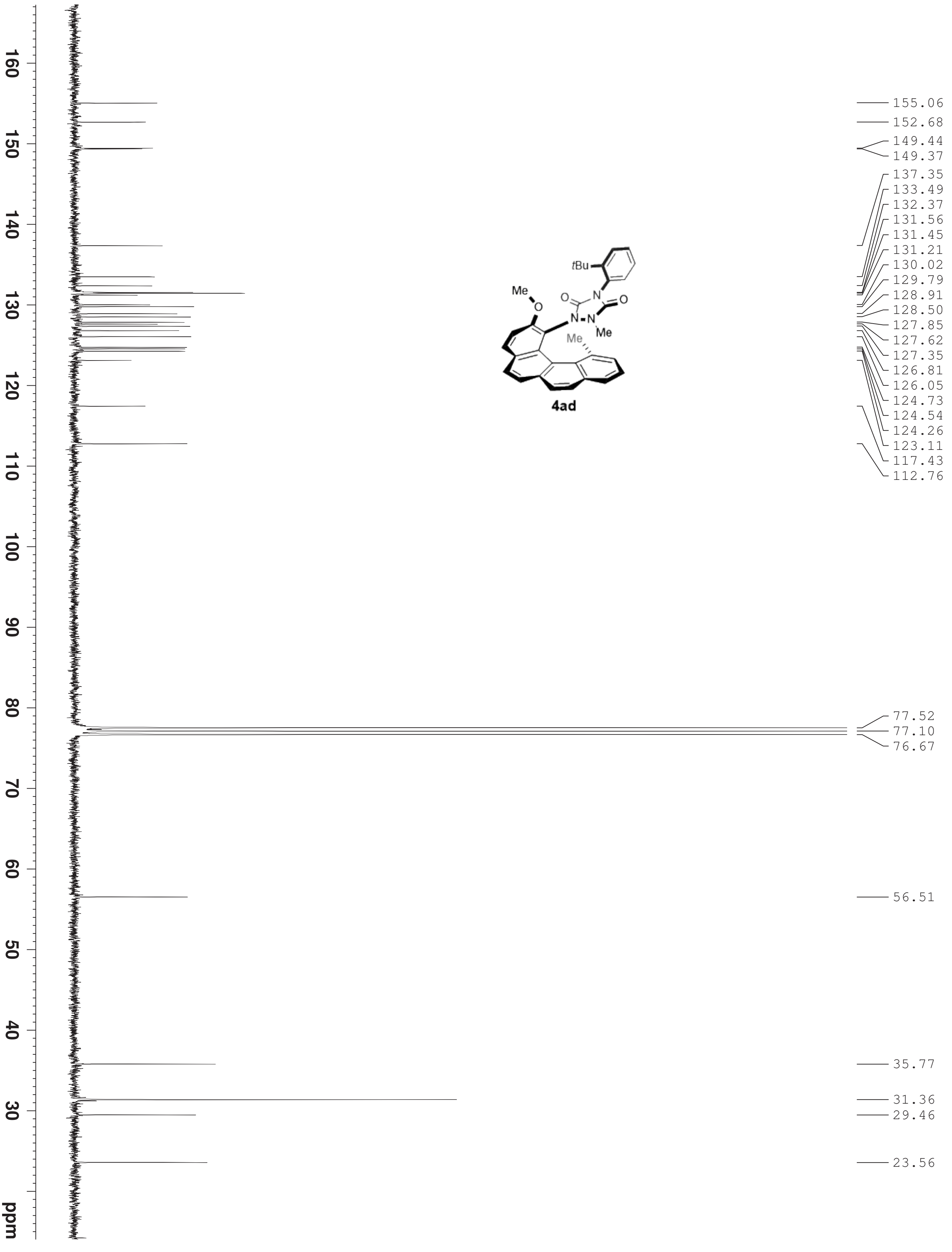

Supplementary Fig. 183. ^{13}C NMR of compound **4ad** (75 MHz, $CDCl_3$)

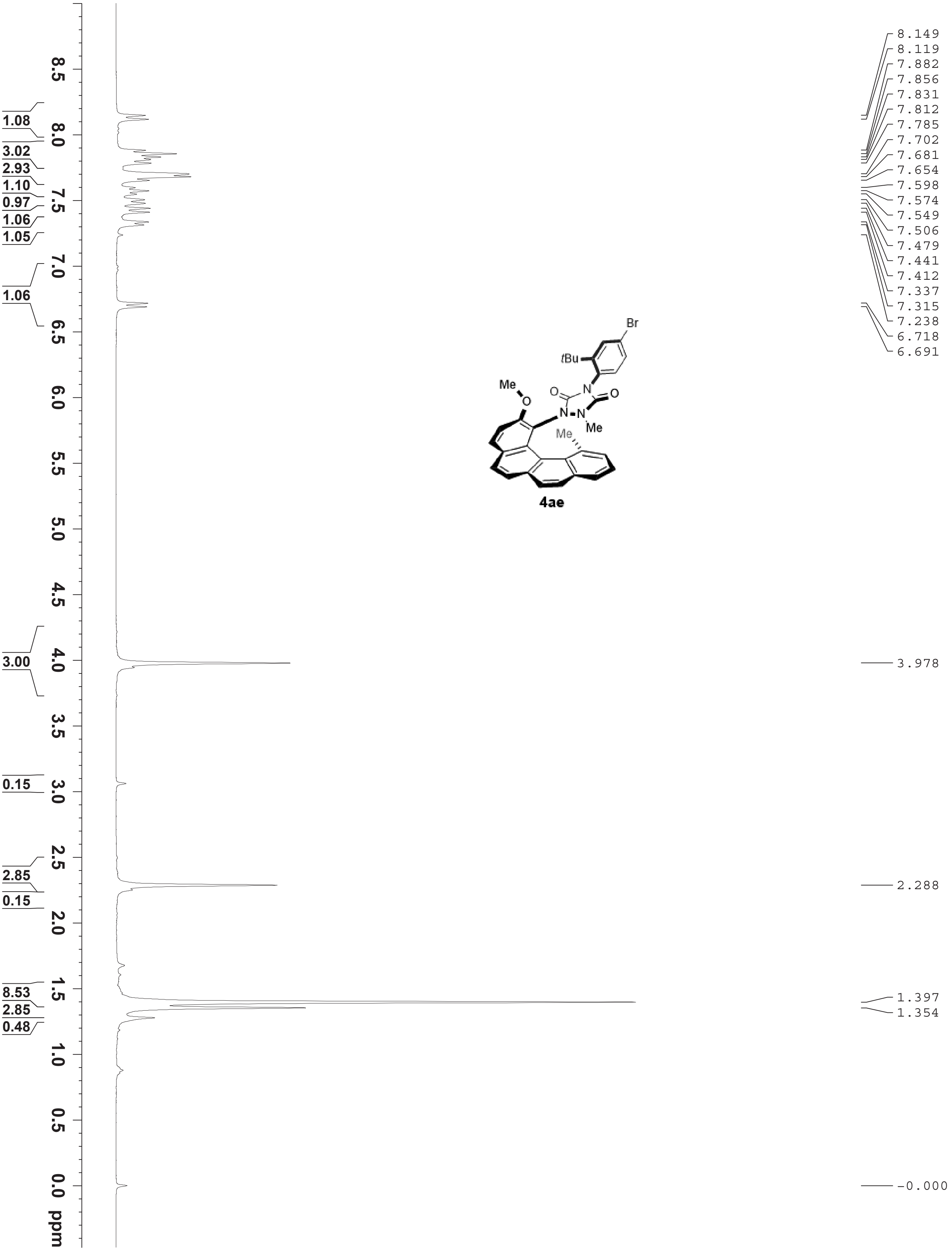

Supplementary Fig. 184. ^{1}H NMR of compound **4ae** (300 MHz, $CDCl_3$)

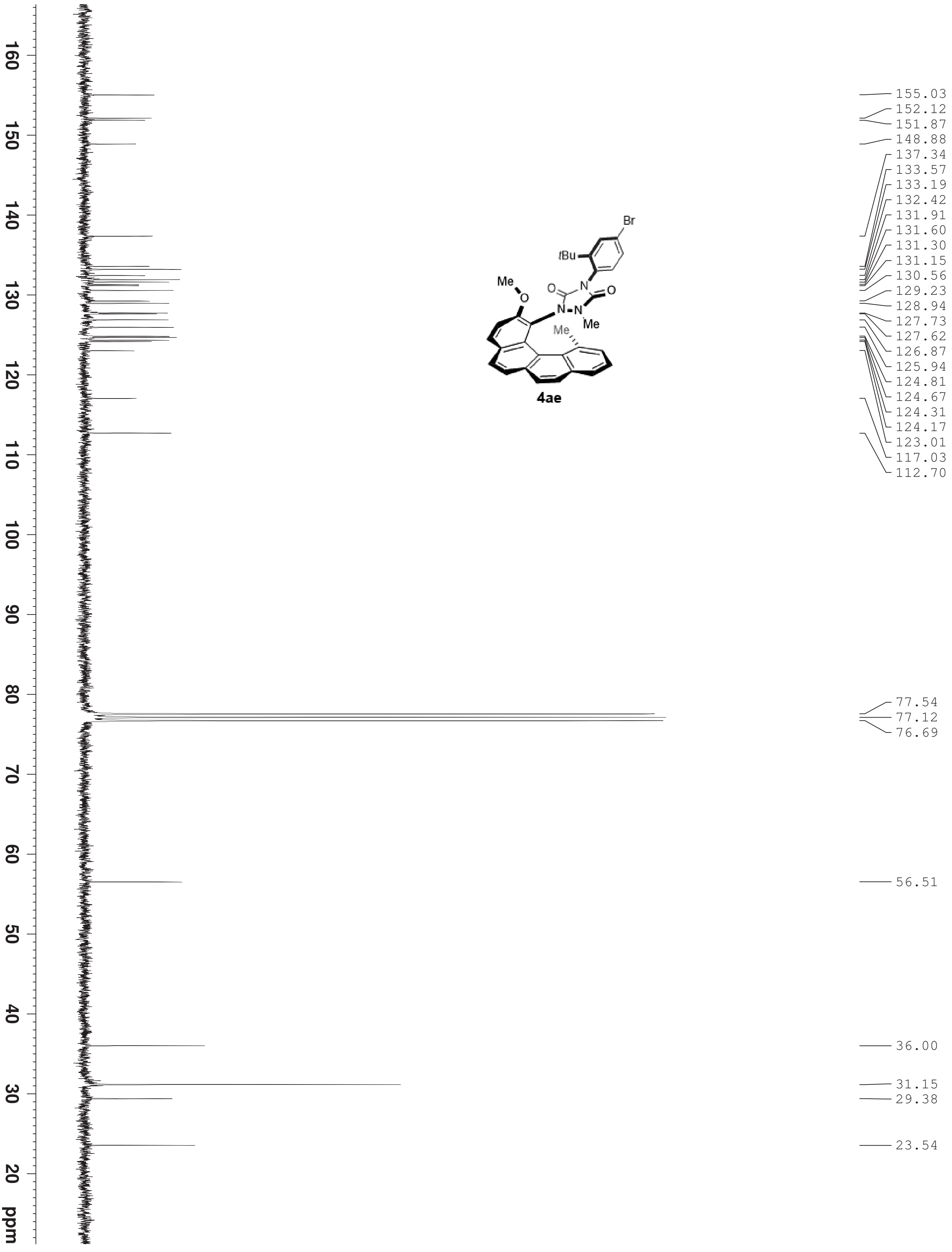

Supplementary Fig. 185. ^{13}C NMR of compound **4ae** (75 MHz, $CDCl_3$)

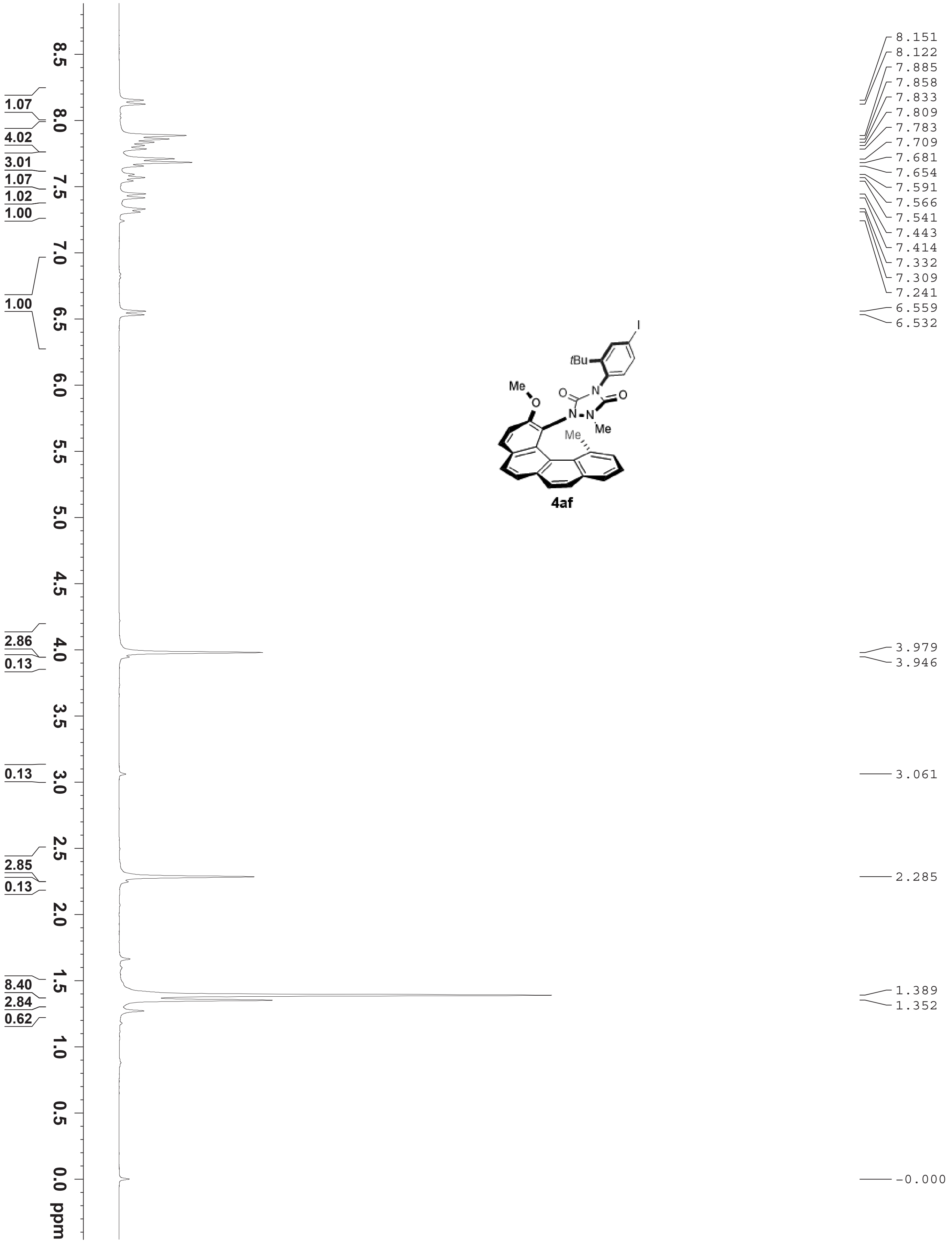

Supplementary Fig. 186. ^{1}H NMR of compound **4af** (300 MHz, $CDCl_3$)

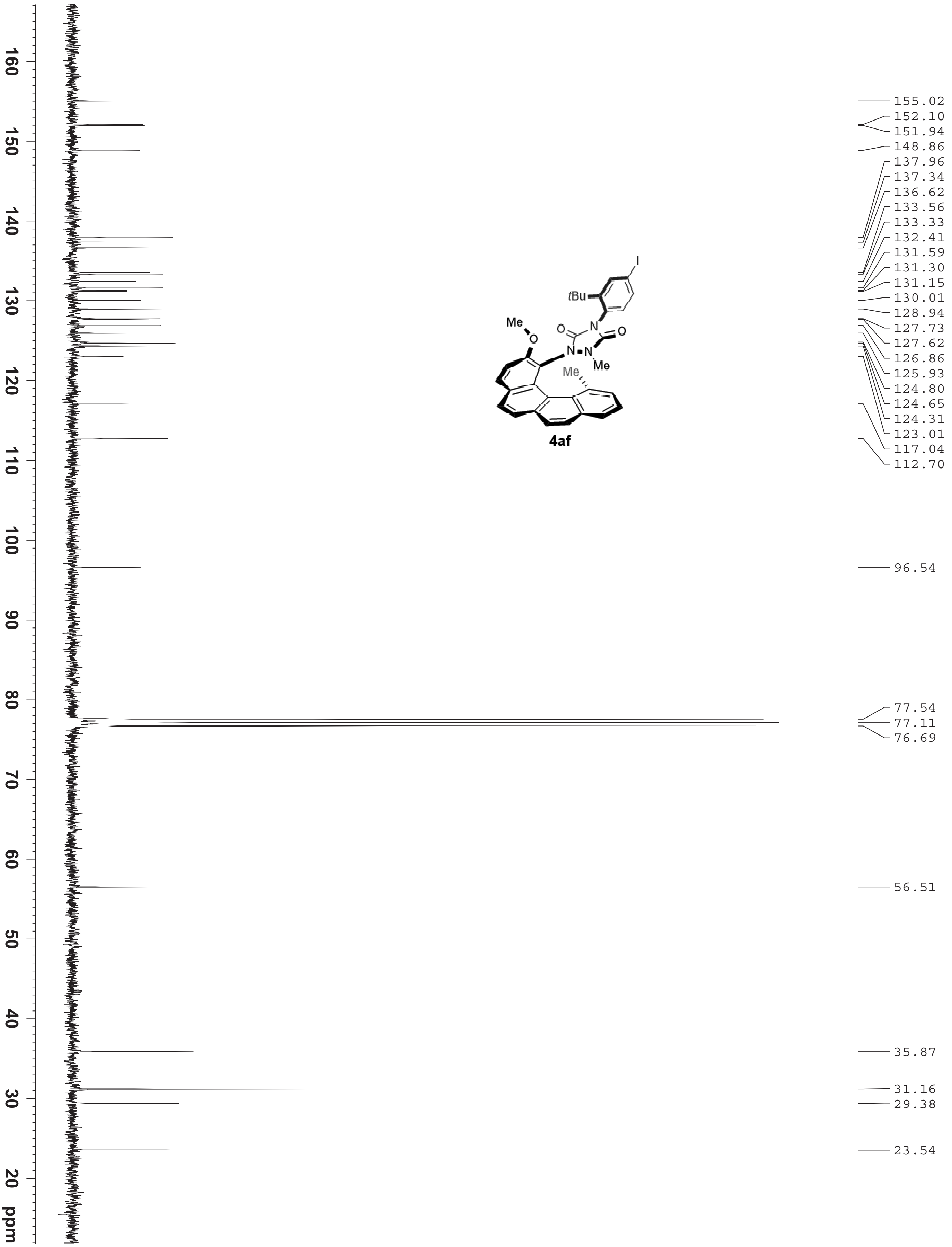

Supplementary Fig. 187. ^{13}C NMR of compound **4af** (75 MHz, $CDCl_3$)

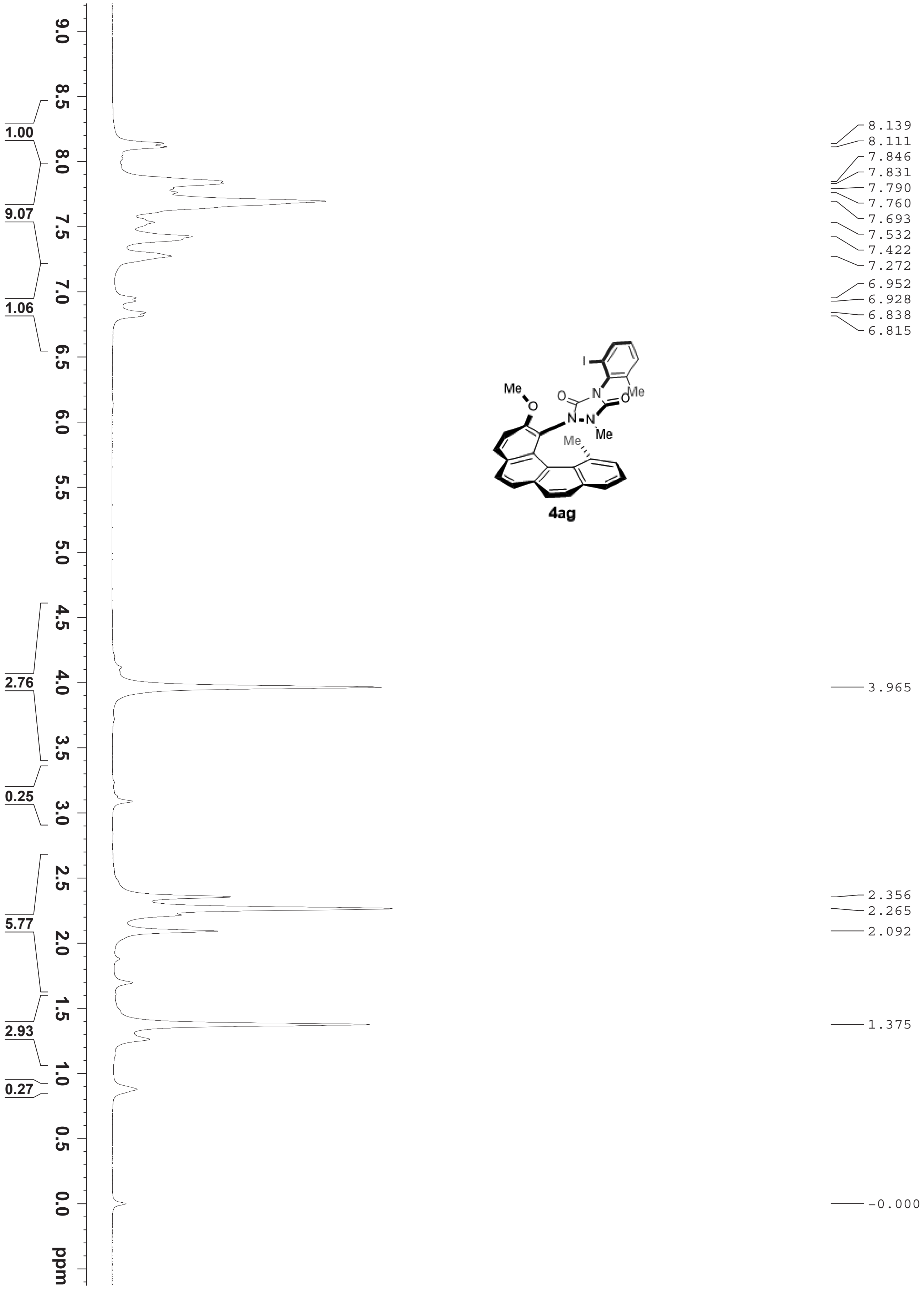

Supplementary Fig. 188. ^{1}H NMR of compound **4ag** (300 MHz, $CDCl_3$)

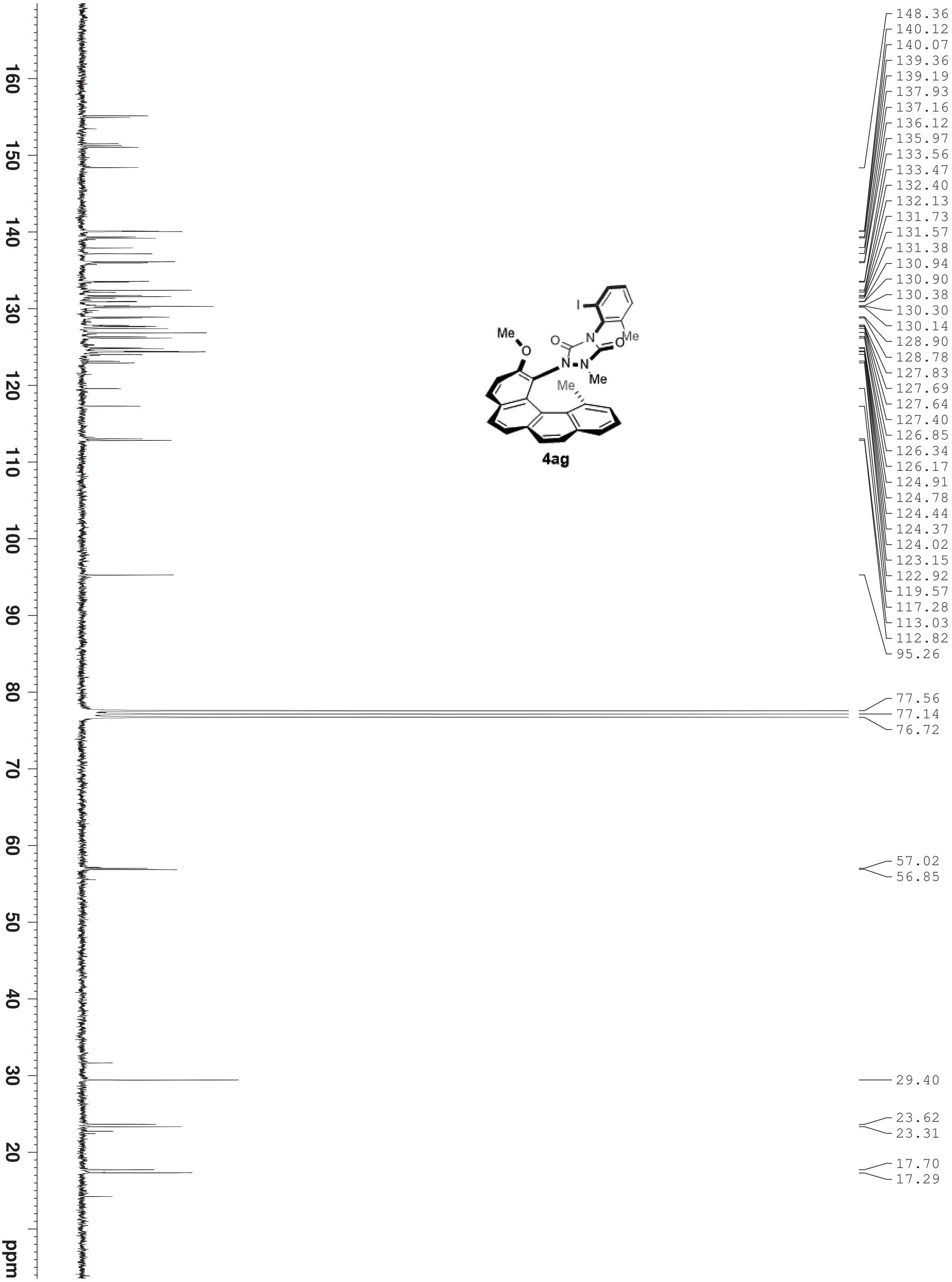

Supplementary Fig. 189. ^{13}C NMR of compound **4ag** (75 MHz, $CDCl_3$)

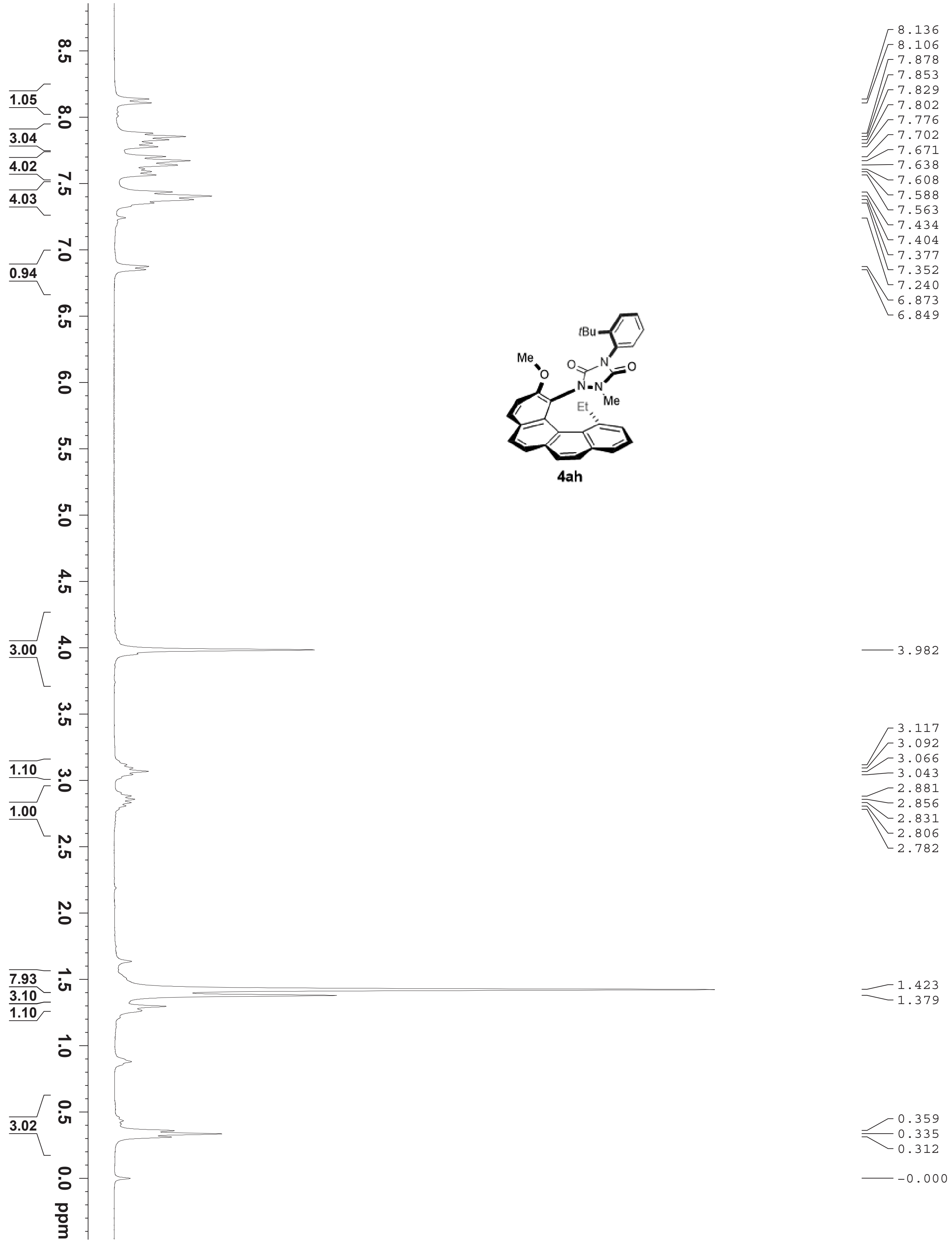

Supplementary Fig. 190. ^{1}H NMR of compound **4ah** (300 MHz, $CDCl_3$)

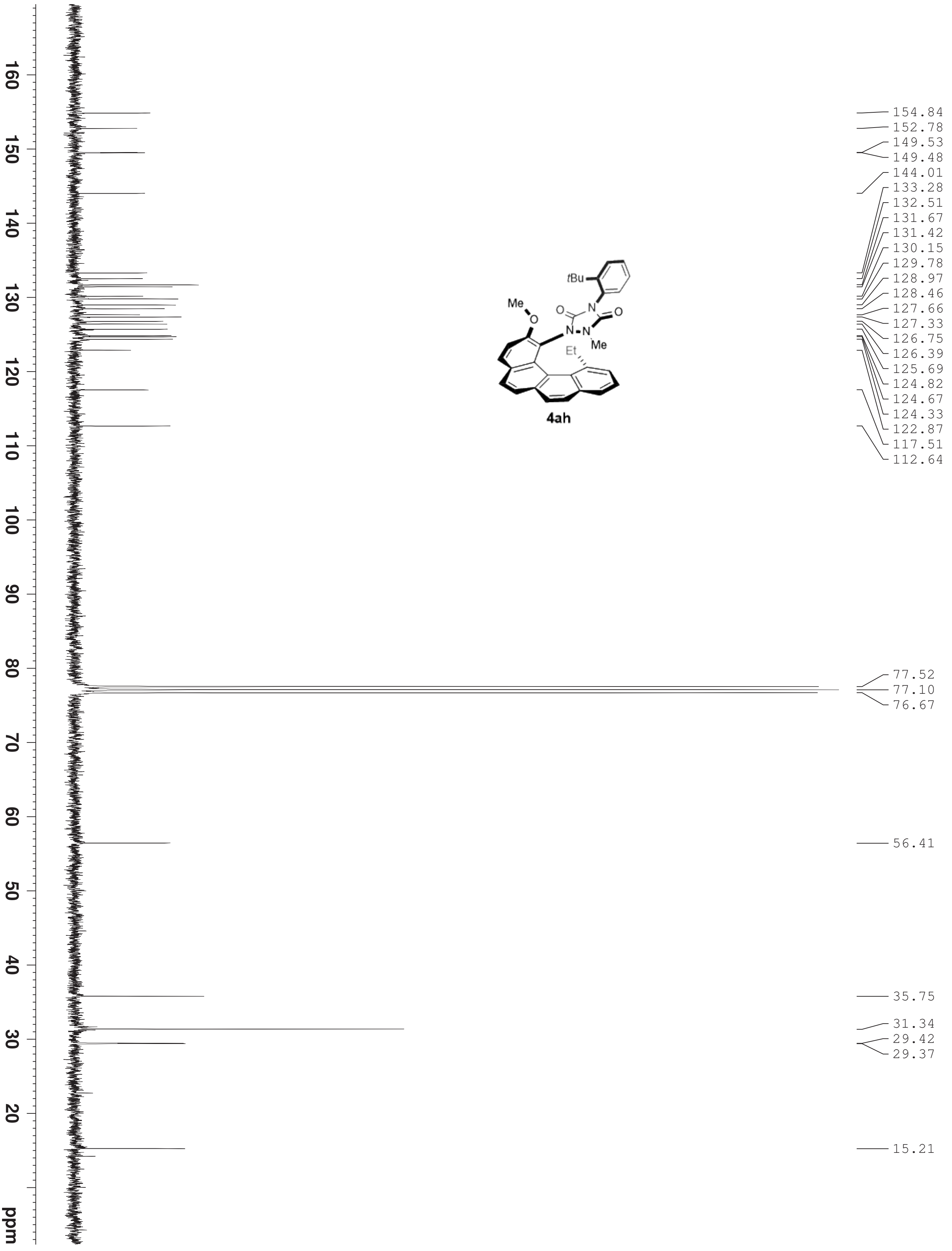

Supplementary Fig. 191. ^{13}C NMR of compound **4ah** (75 MHz, $CDCl_3$)

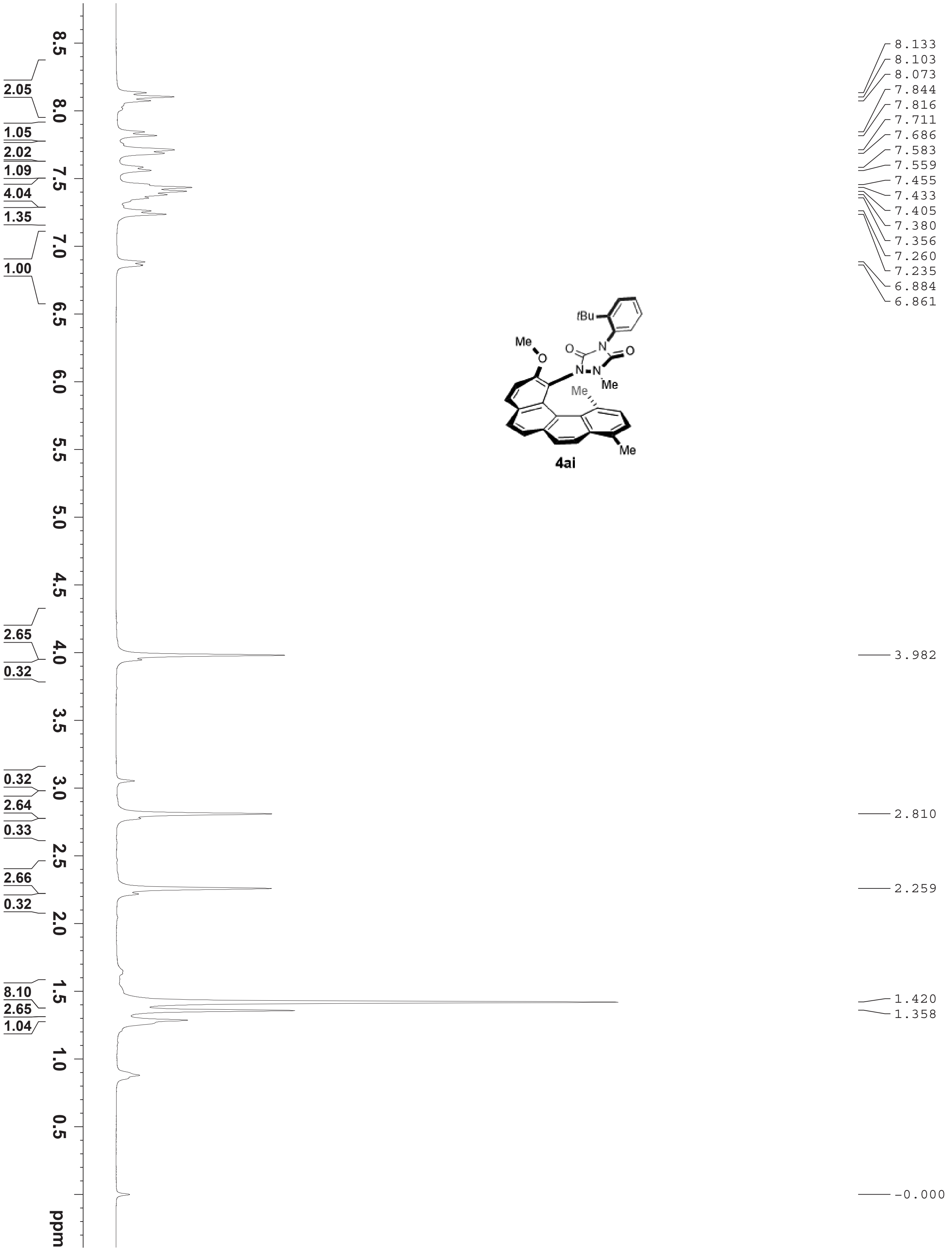

Supplementary Fig. 192. ^{1}H NMR of compound **4ai** (300 MHz, $CDCl_3$)

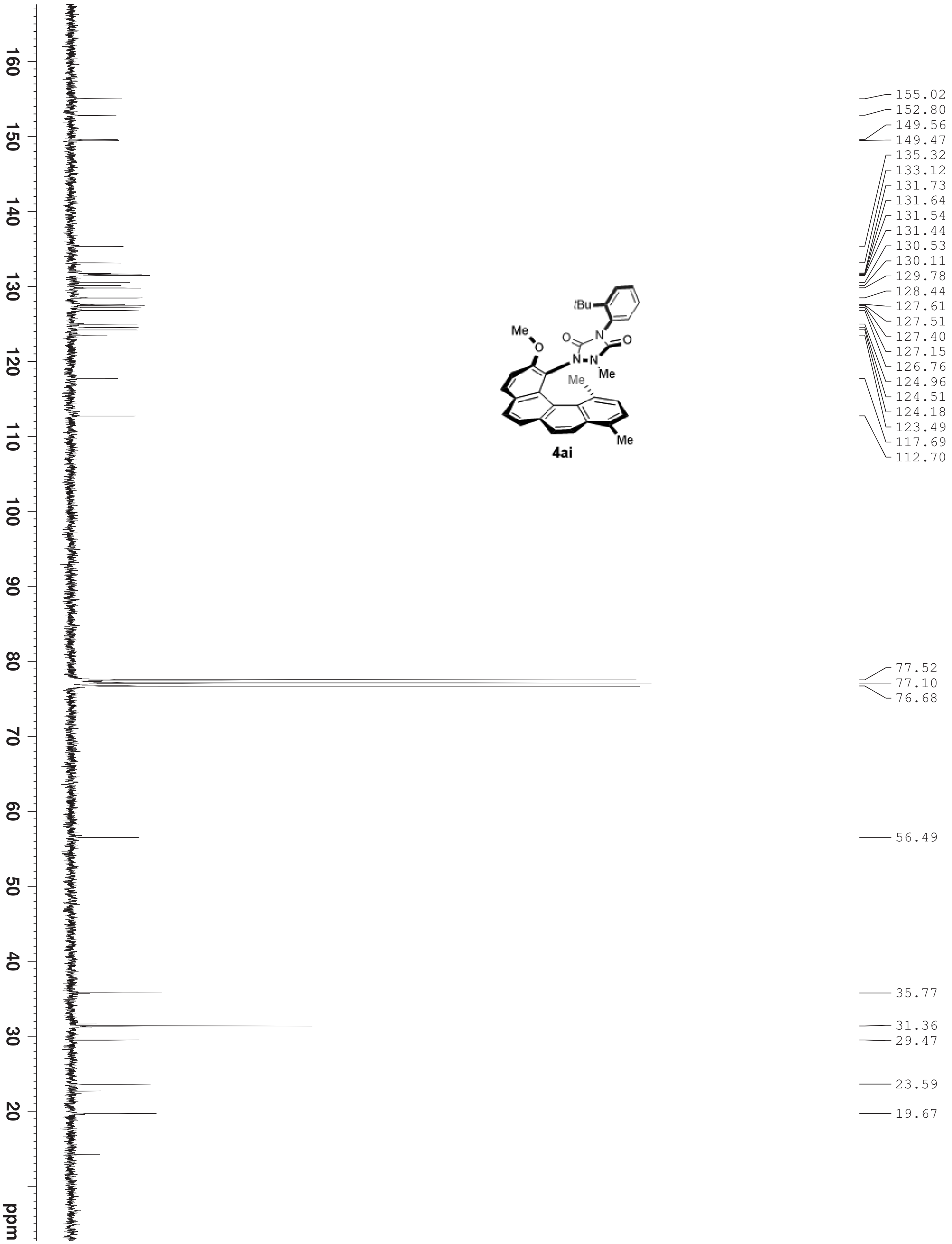

Supplementary Fig. 193. ^{13}C NMR of compound **4ai** (75 MHz, $CDCl_3$)

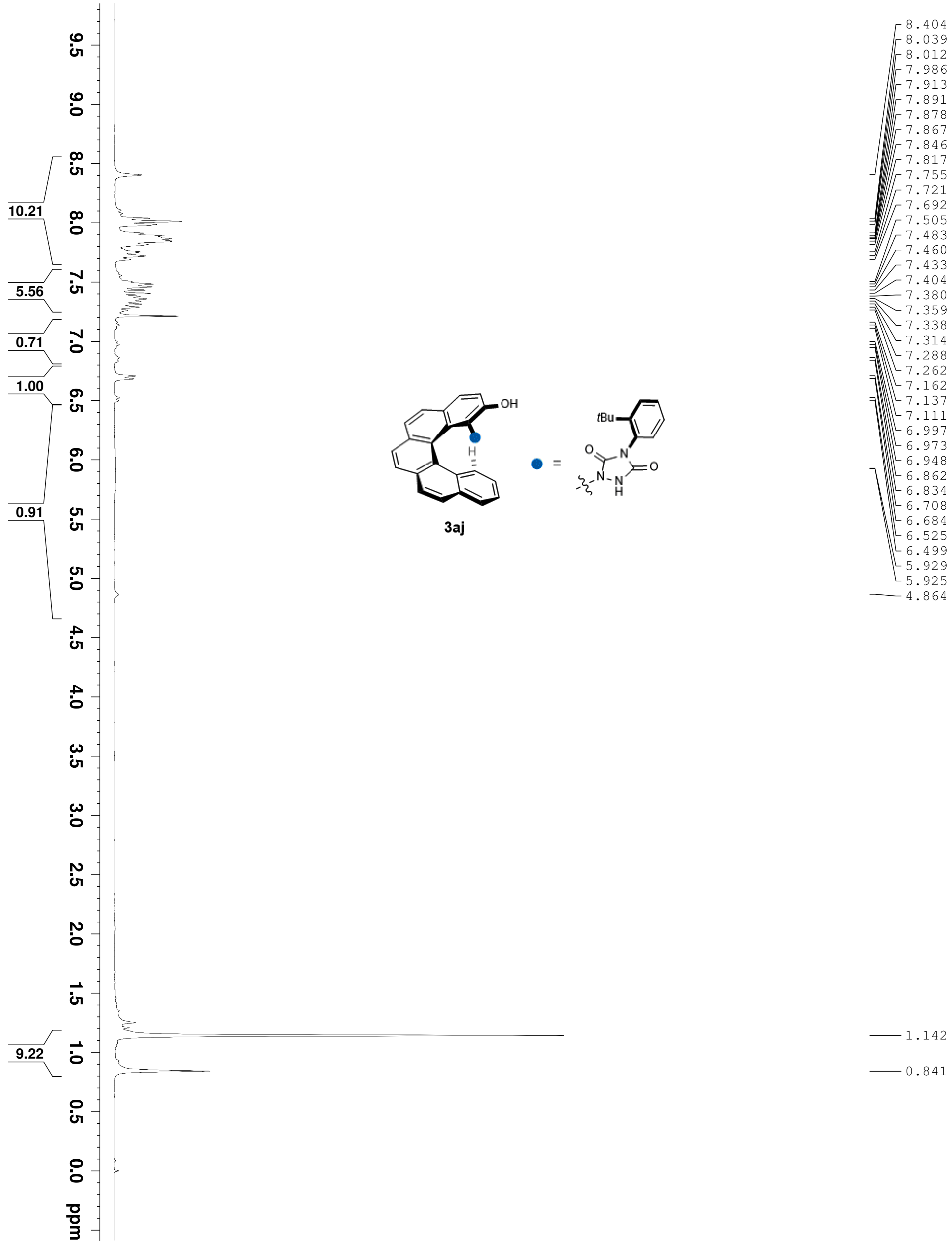

Supplementary Fig. 194. ^{1}H NMR of compound **3aj** (300 MHz, $CDCl_3$)

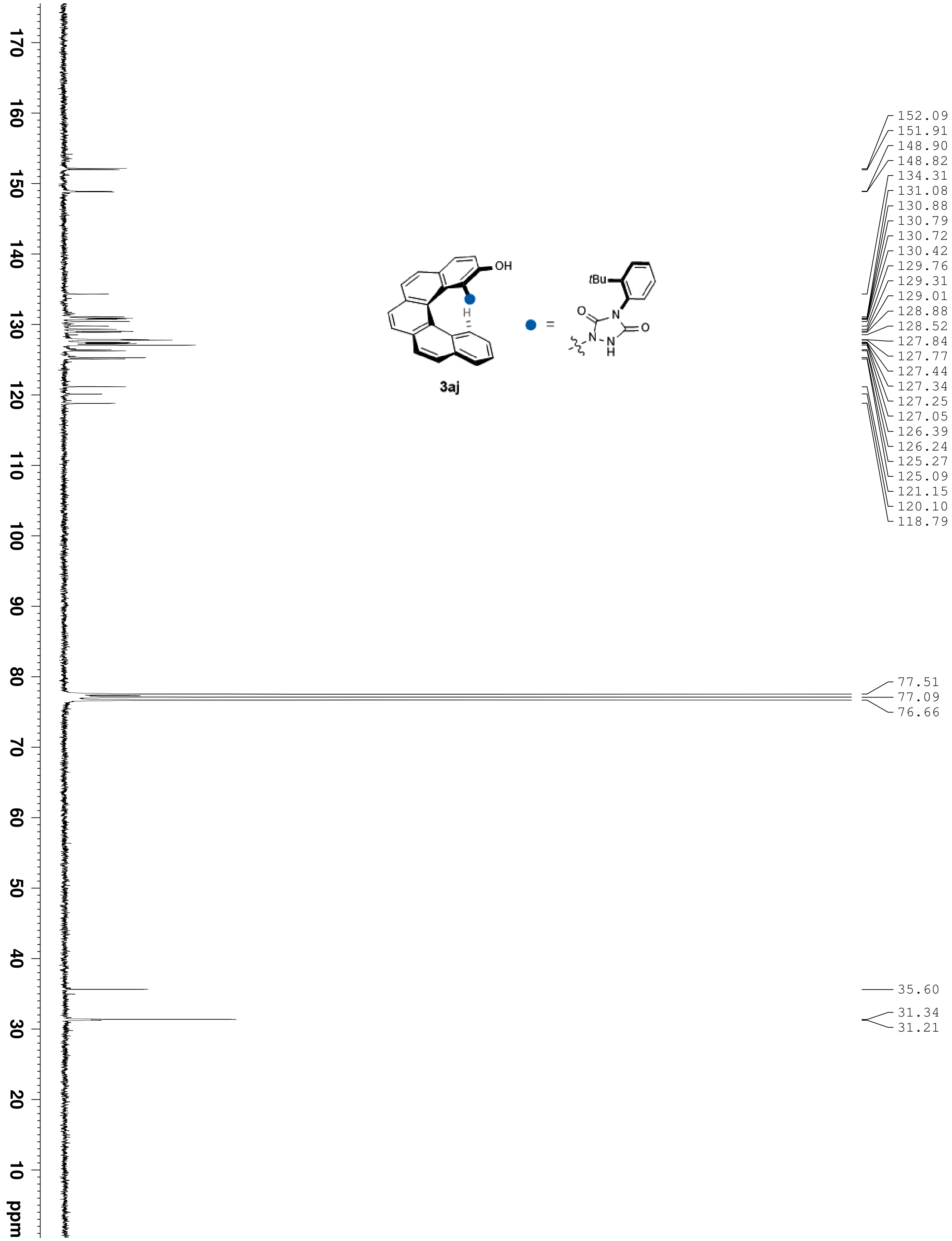

Supplementary Fig. 195. ^{13}C NMR of compound **3aj** (75 MHz, $CDCl_3$)

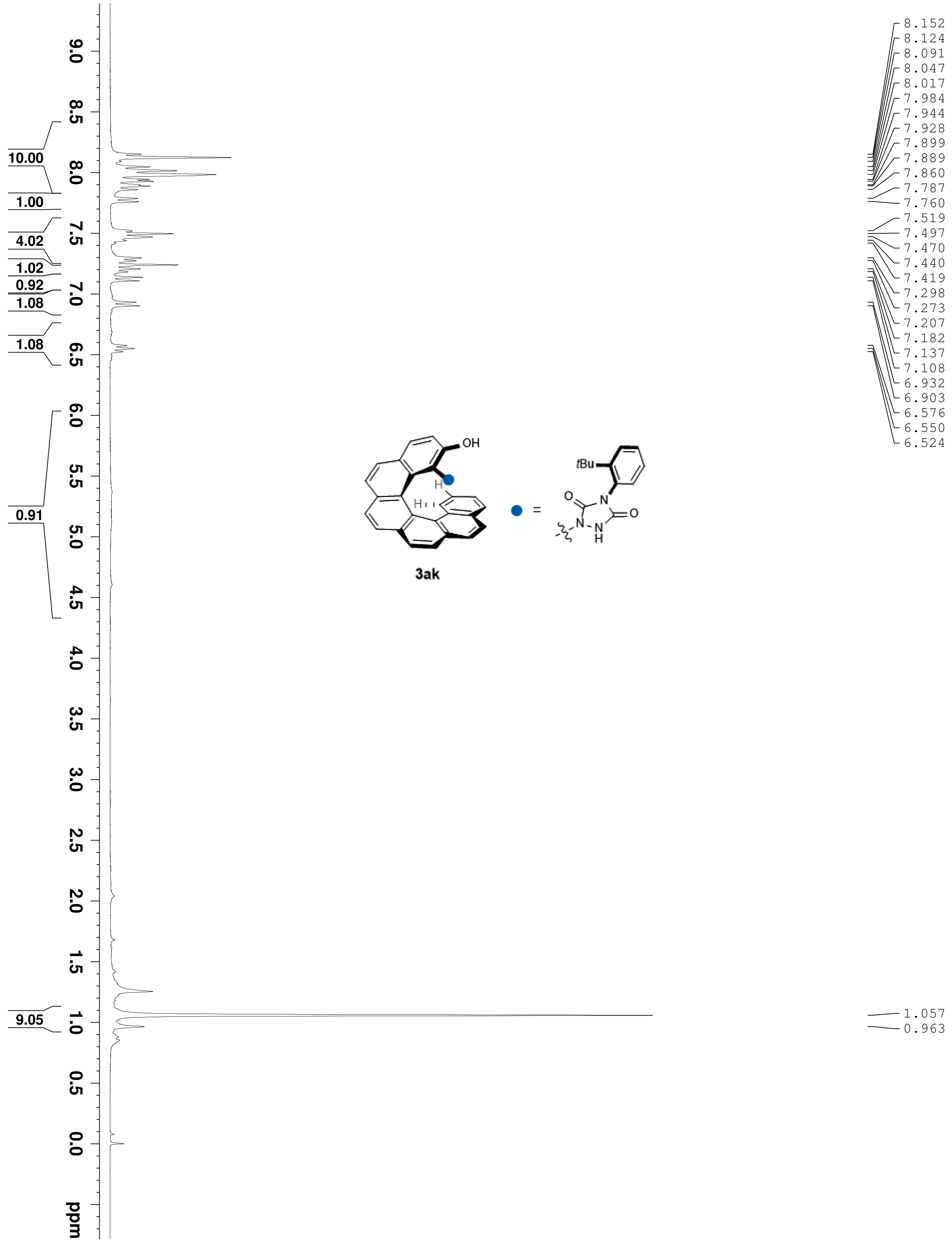

Supplementary Fig. 196. ^{1}H NMR of compound **3ak** (300 MHz, $CDCl_3$)

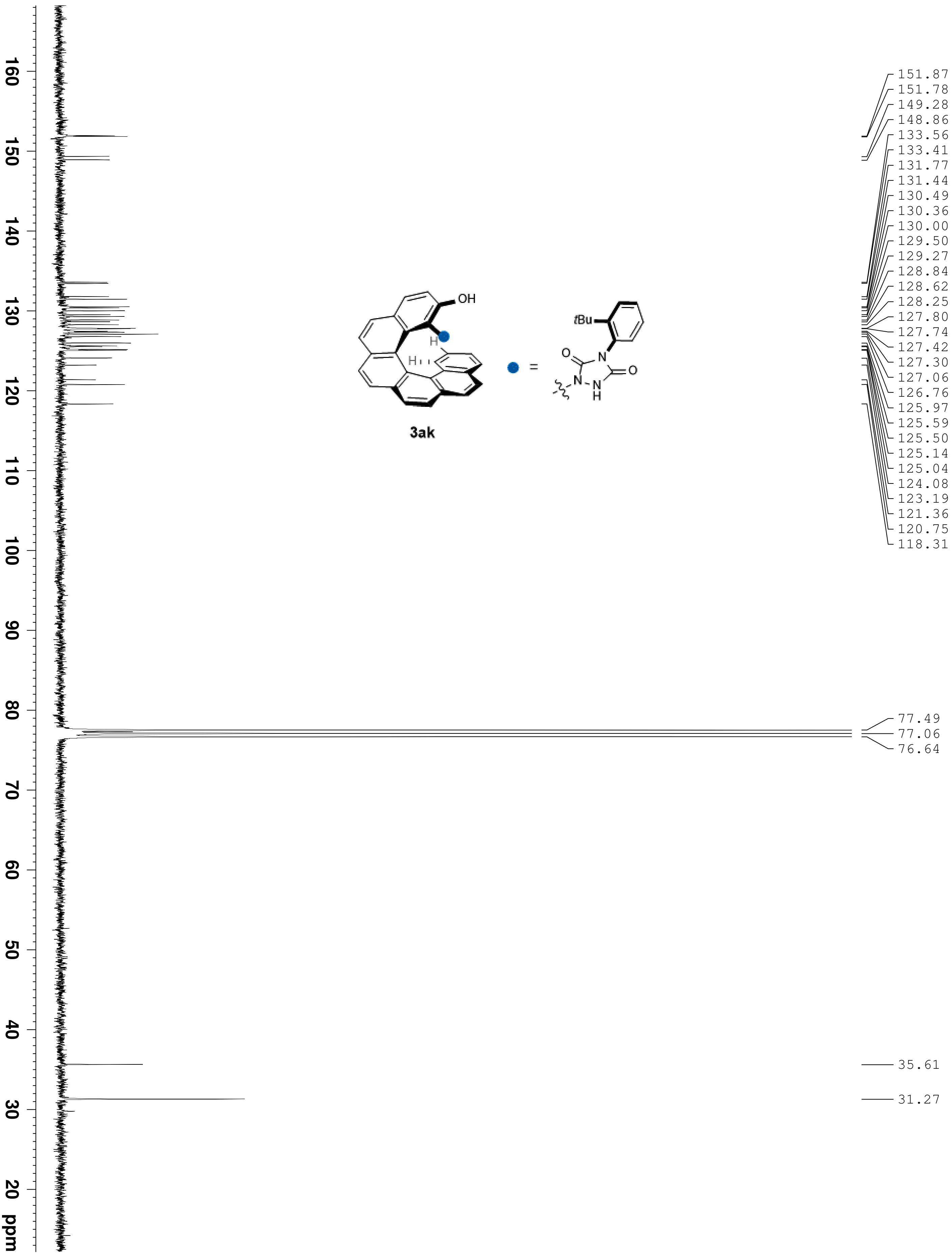

Supplementary Fig. 197. ^{13}C NMR of compound **3ak** (75 MHz, $CDCl_3$)

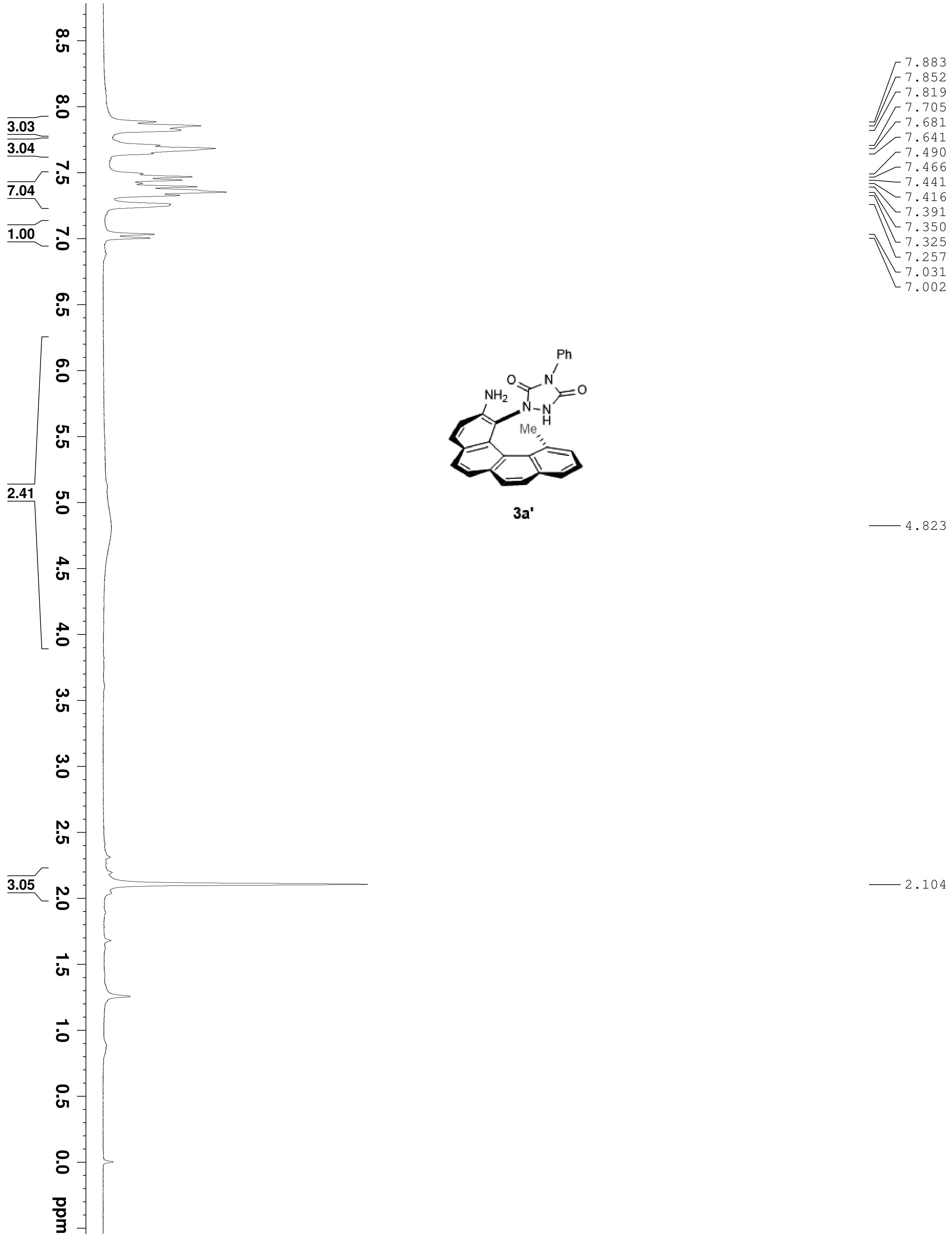

Supplementary Fig. 198. ^{1}H NMR of compound **3a'** (300 MHz, $CDCl_3$)

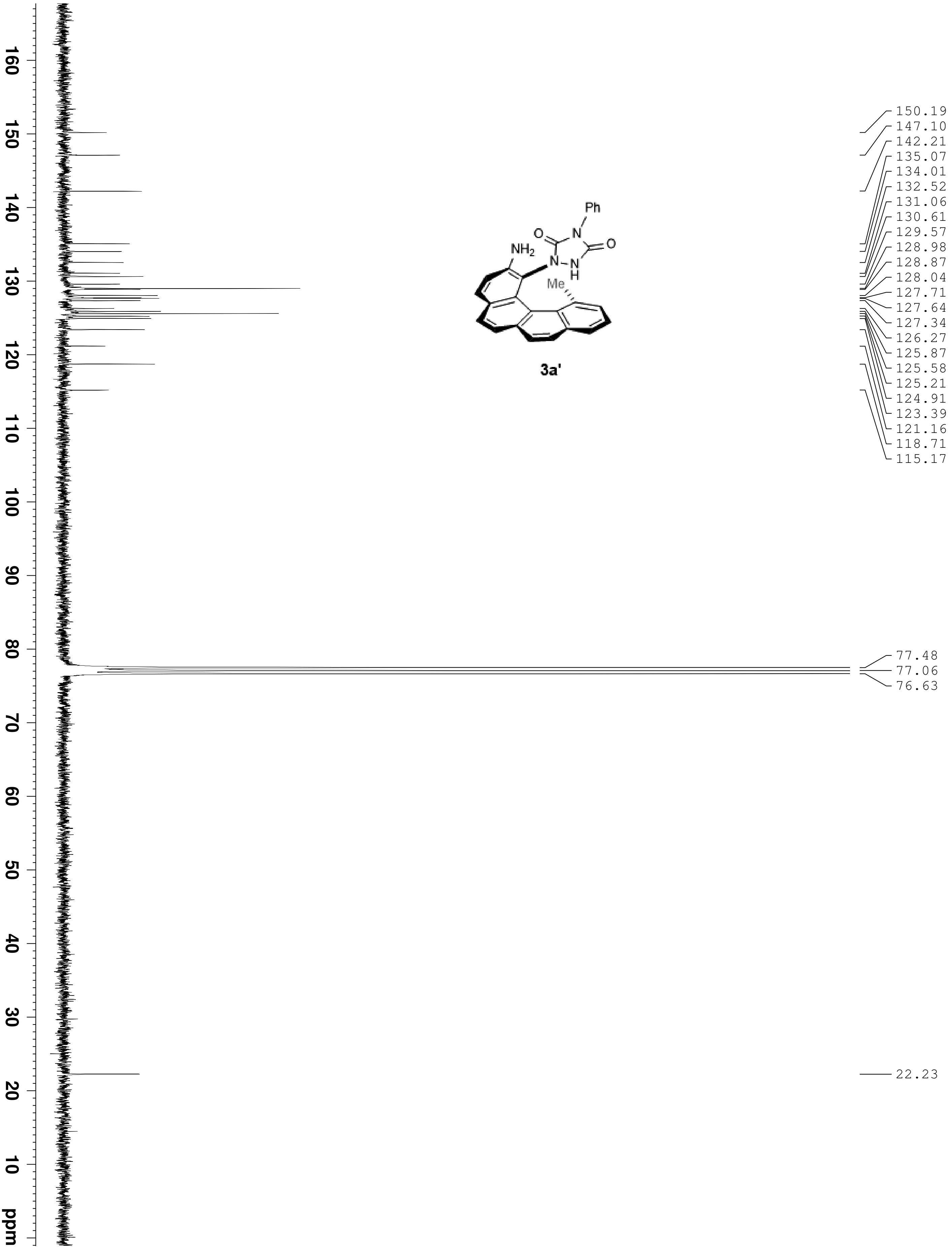

Supplementary Fig. 199. ^{13}C NMR of compound **3a'** (75 MHz, $CDCl_3$)

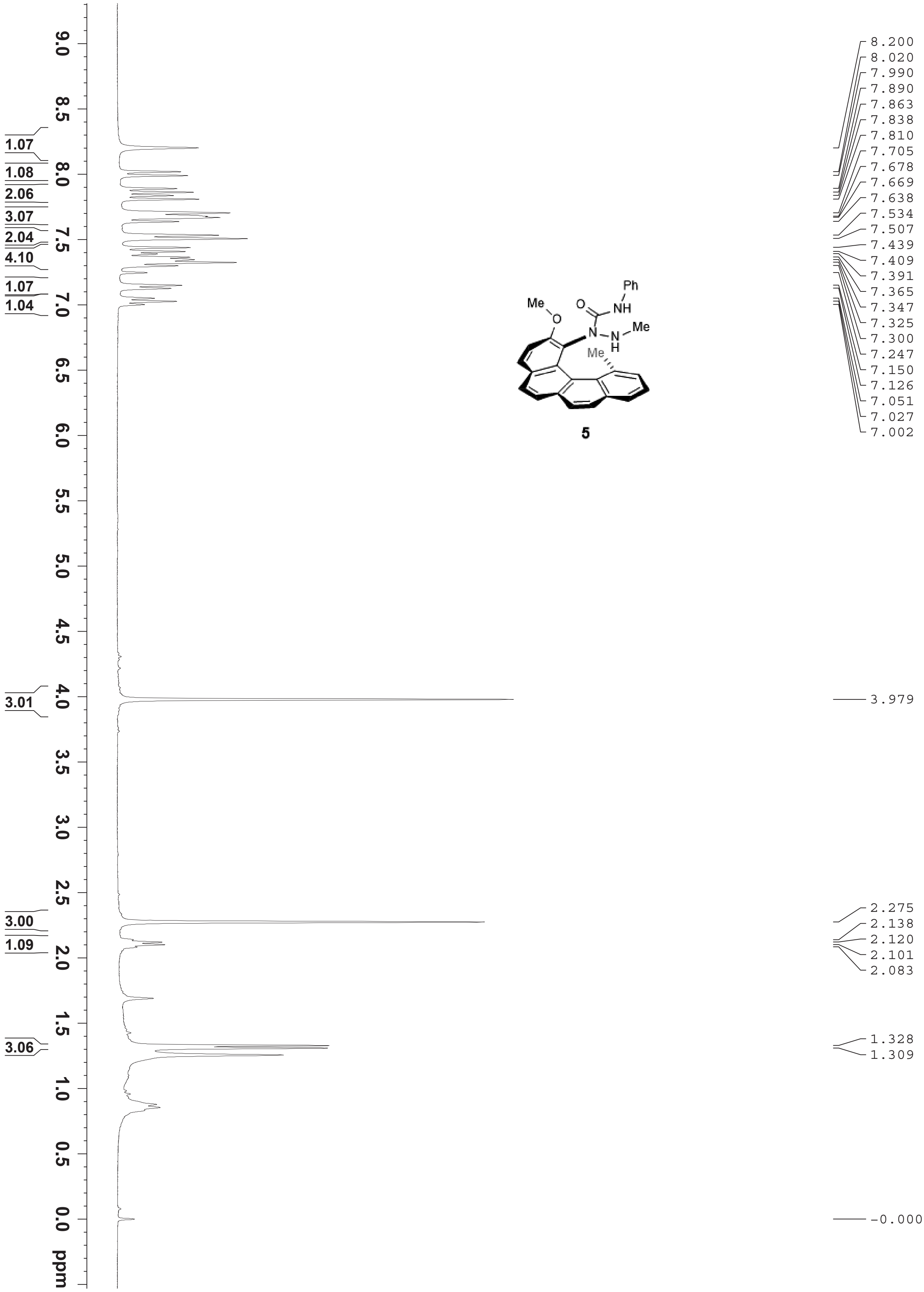

Supplementary Fig. 200. ^{1}H NMR of compound **5** (300 MHz, $CDCl_3$)

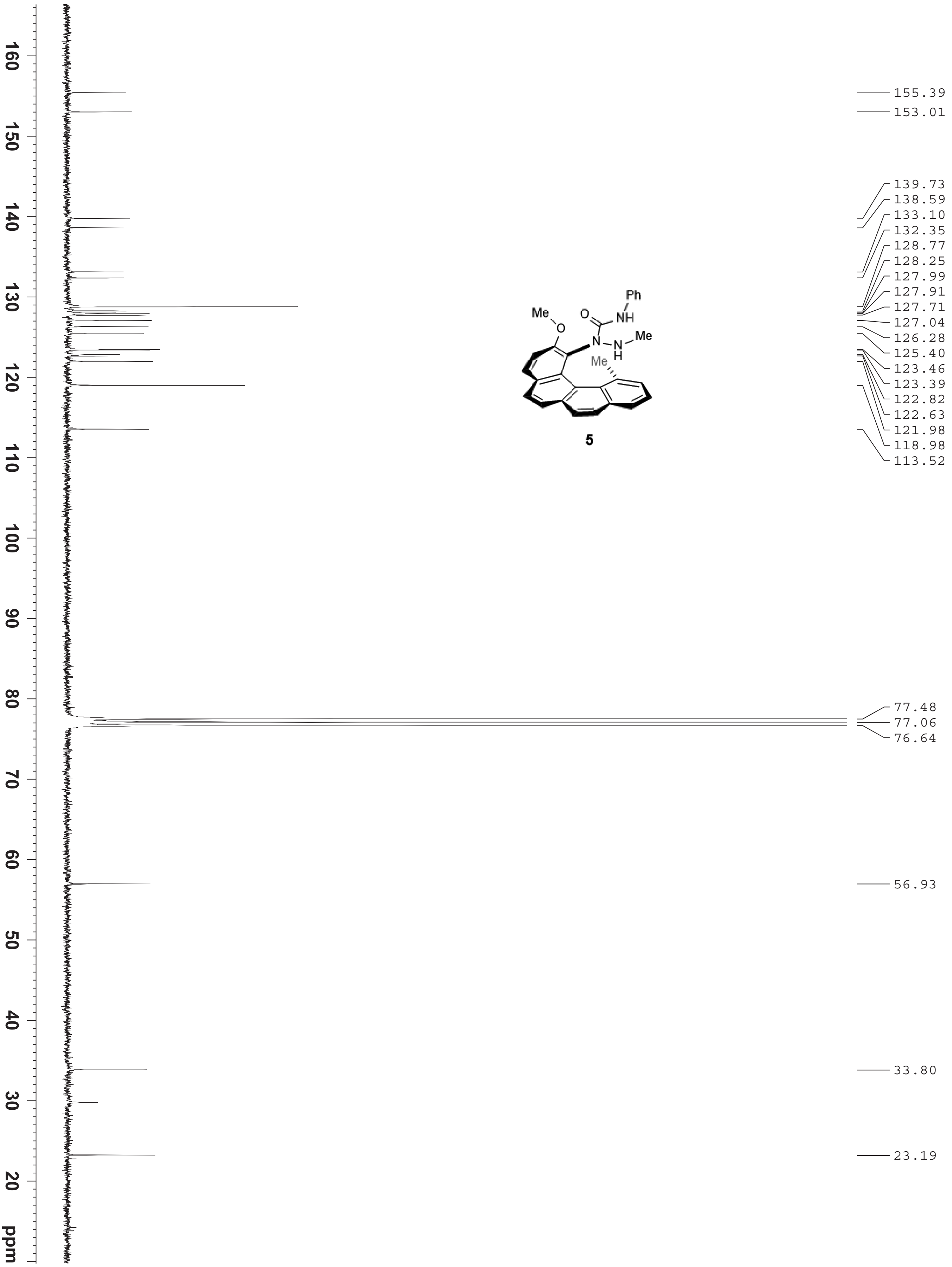

Supplementary Fig. 201. ^{13}C NMR of compound **5** (75 MHz, $CDCl_3$)

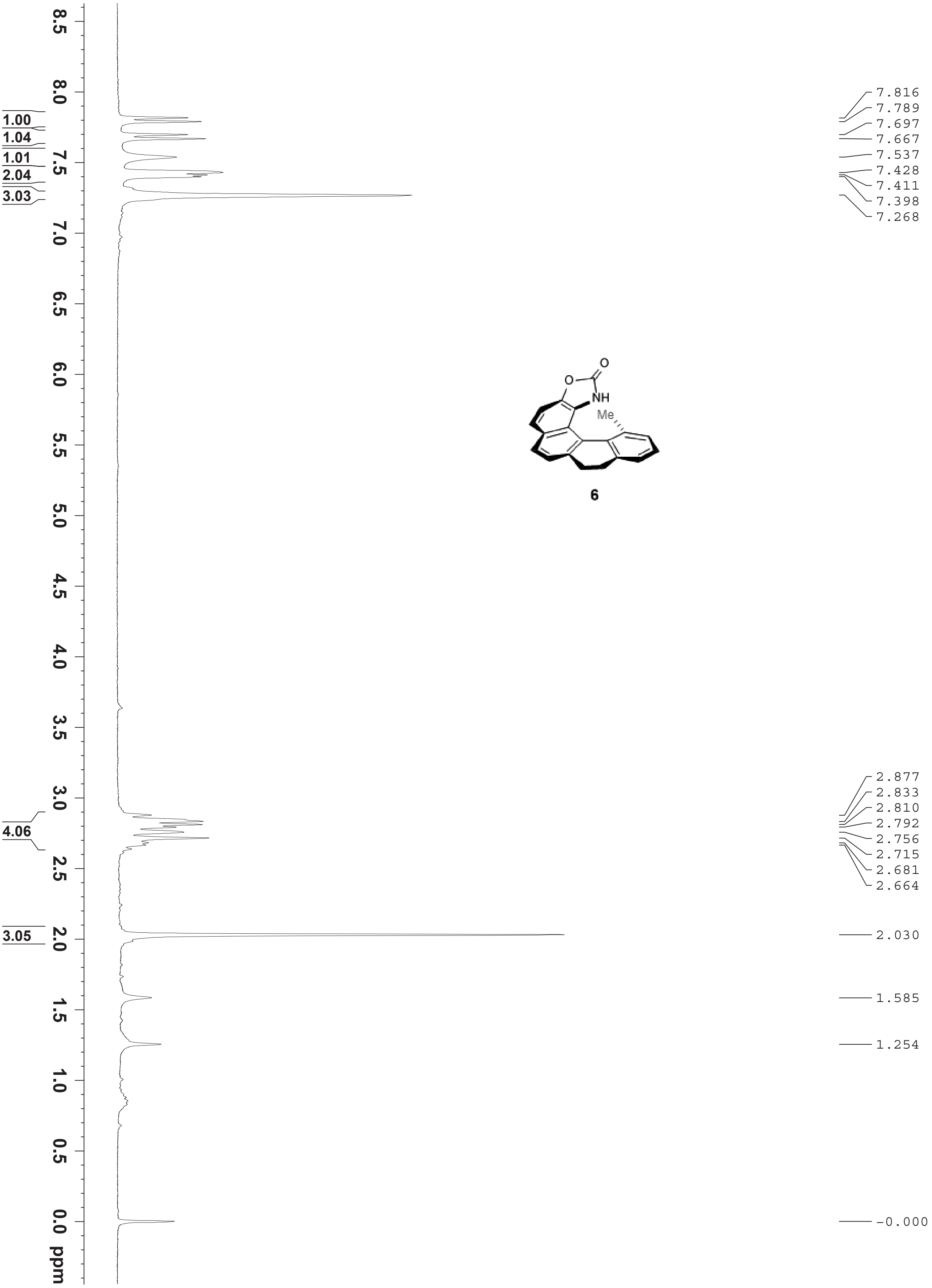

Supplementary Fig. 202. ^{1}H NMR of compound **6** (300 MHz, $CDCl_3$)

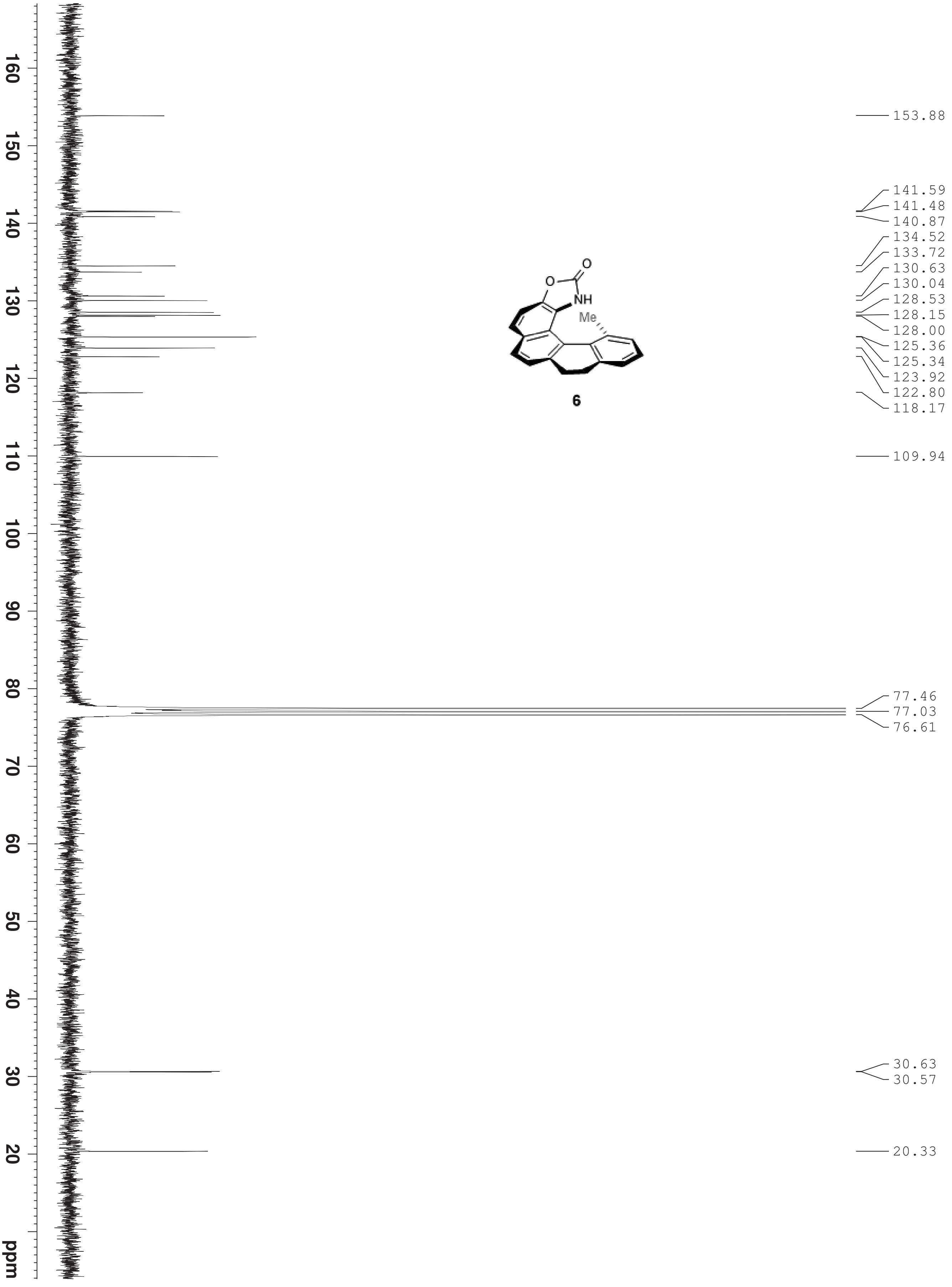

Supplementary Fig. 203. ^{13}C NMR of compound **6** (75 MHz, $CDCl_3$)

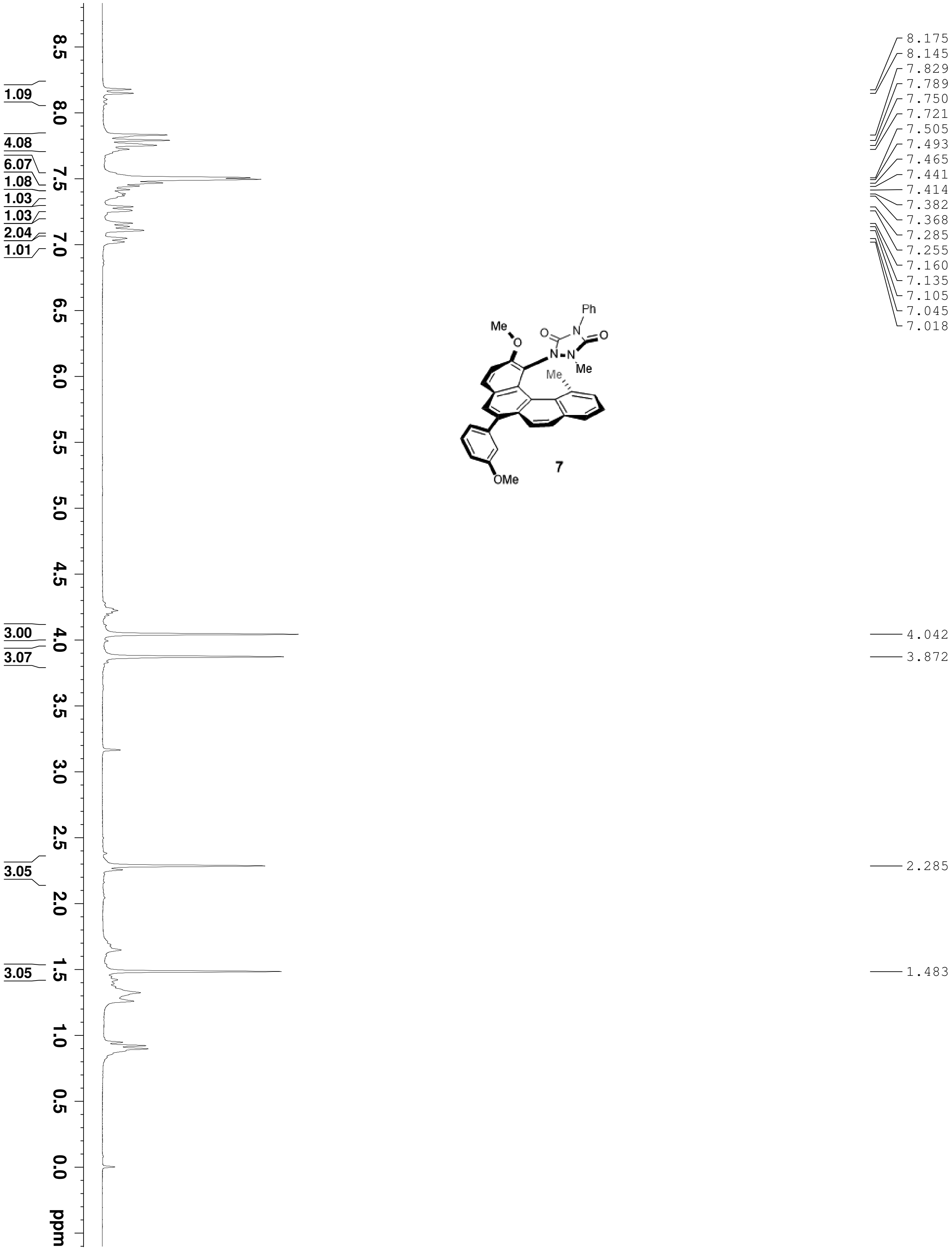

Supplementary Fig. 204. H NMR of compound **7** (300 MHz, $CDCl_3$)

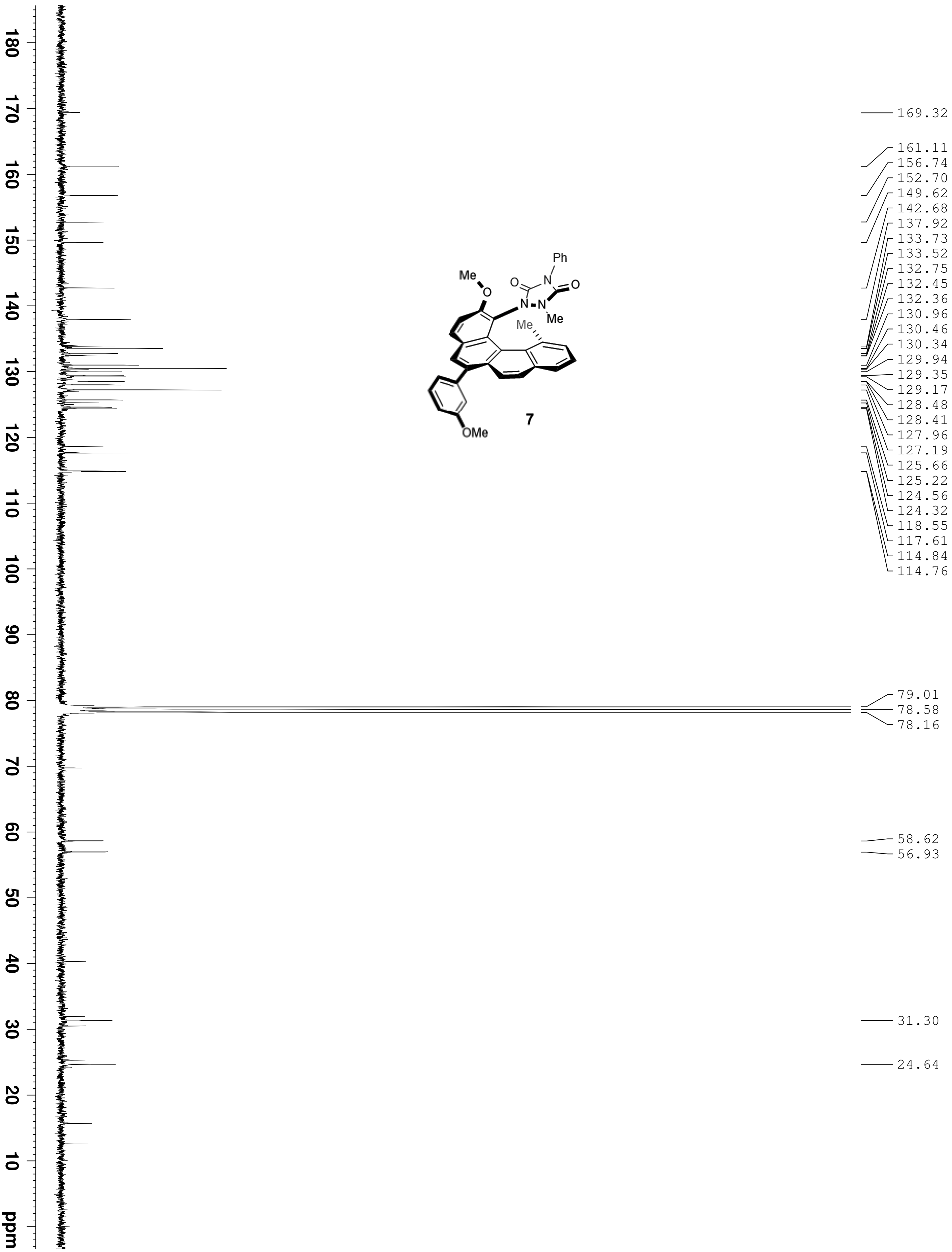

Supplementary Fig. 205. ^{13}C NMR of compound **7** (75 MHz, $CDCl_3$)

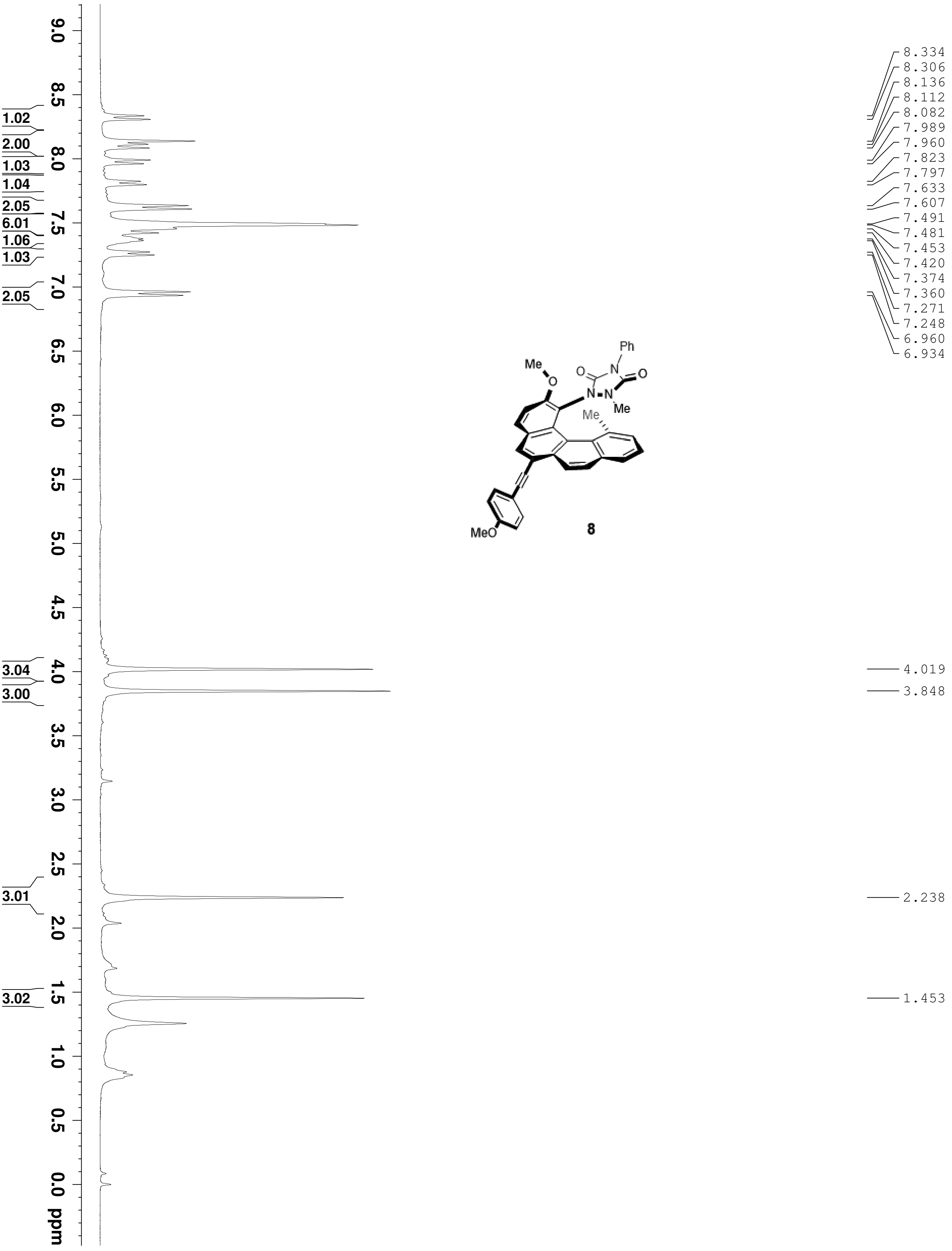

Supplementary Fig. 206. ^{1}H NMR of compound **8** (300 MHz, $CDCl_3$)

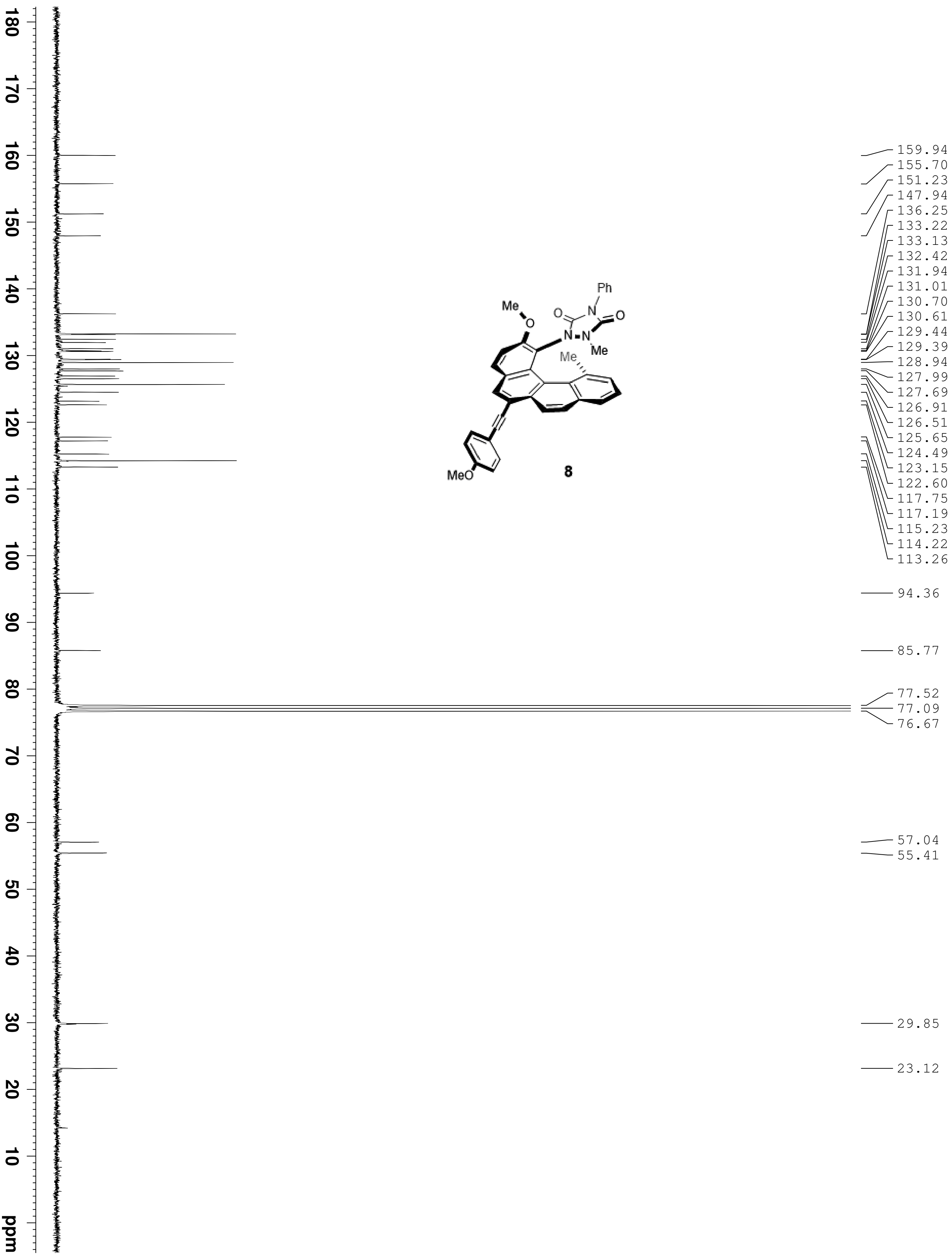

Supplementary Fig. 207. ^{13}C NMR of compound **8** (75 MHz, $CDCl_3$)

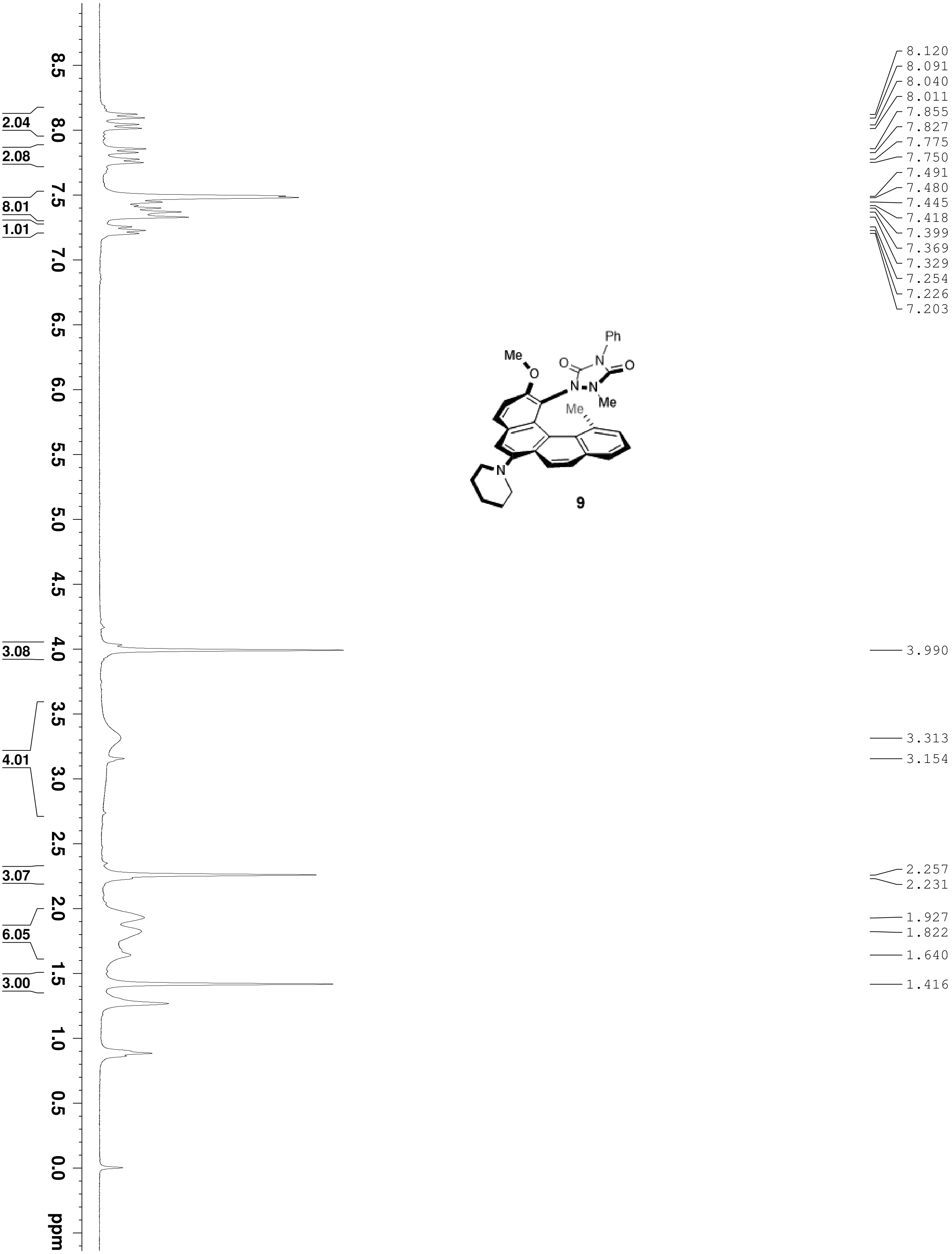

Supplementary Fig. 208. ^{1}H NMR of compound **9** (300 MHz, $CDCl_3$)

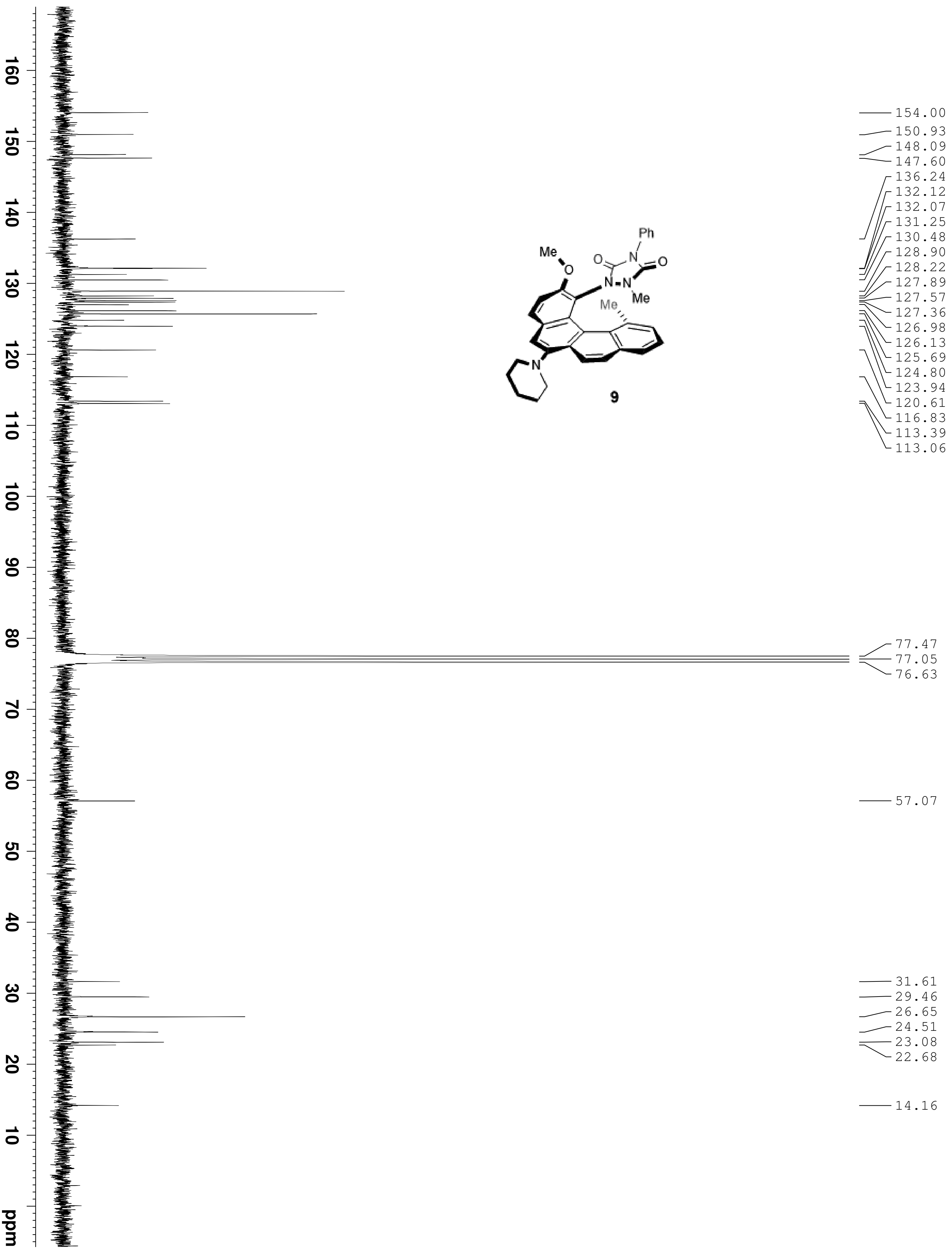

Supplementary Fig. 209. ^{13}C NMR of compound **9** (75 MHz, $CDCl_3$)

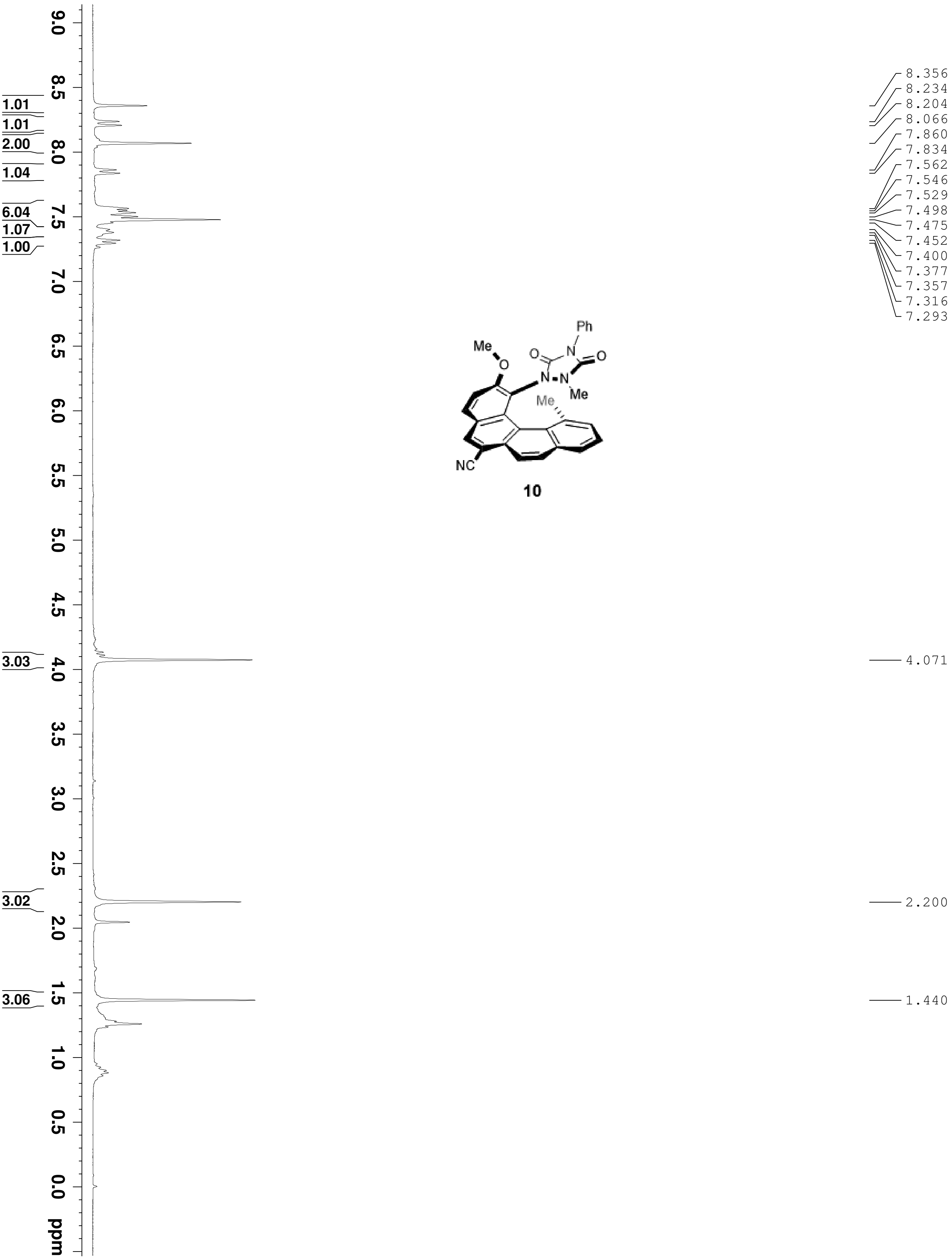

Supplementary Fig. 210. ^{1}H NMR of compound **10** (300 MHz, $CDCl_3$)

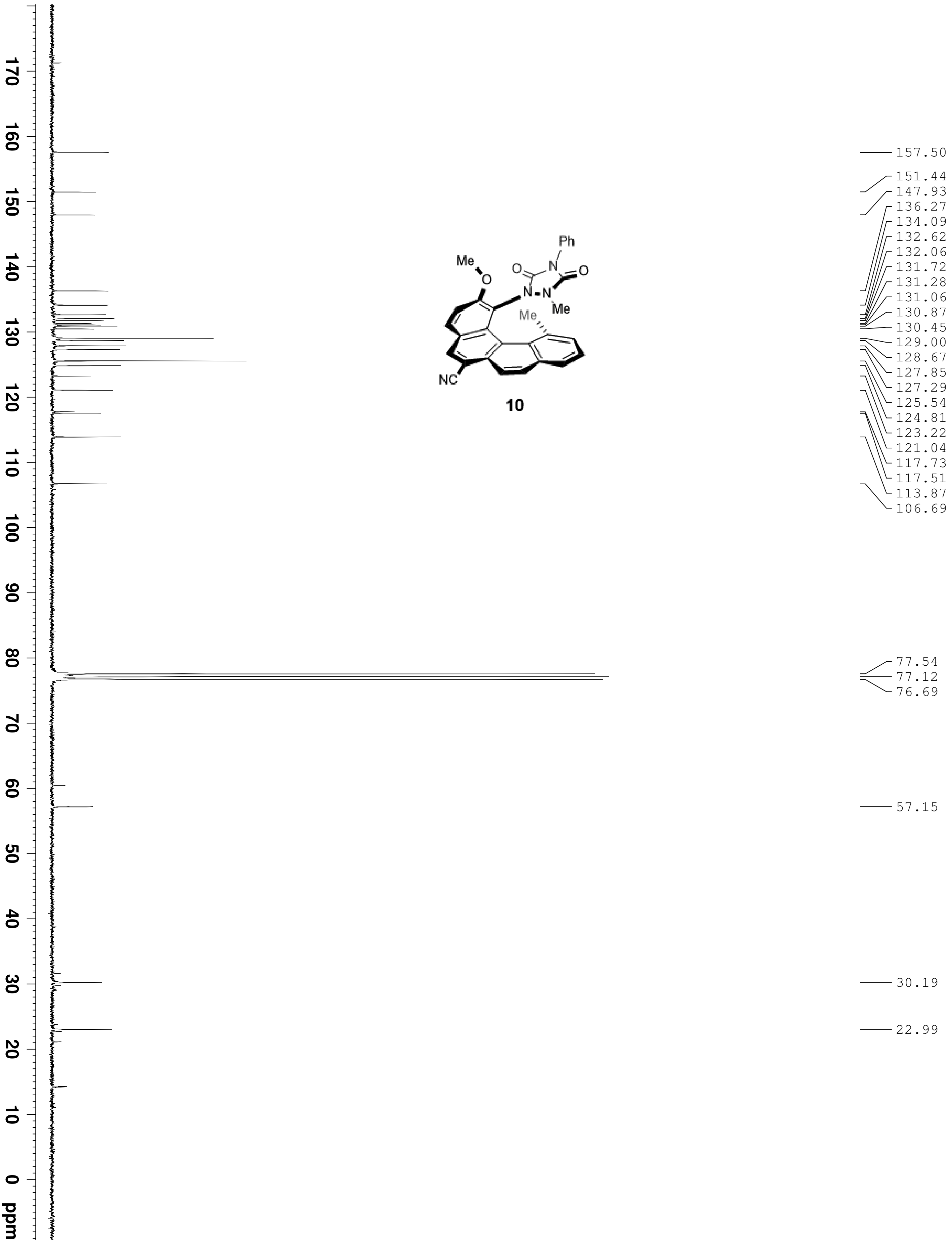

Supplementary Fig. 211. ^{13}C NMR of compound **10** (75 MHz, $CDCl_3$)

3 Supplementary References

1. Bausch, M. J. & David, B. Proton, electron, and hydrogen atom transfers from ions, radicals, and radical ions derived from substituted urazoles and triazolinediones. *J. Org. Chem.* **57**, 1118–1124 (1992).
2. Zhang, J.-W. et al. Discovery and enantiocontrol of axially chiral urazoles via organocatalytic tyrosine click reaction. *Nat. Commun.* **7**, 10677−10686 (2016).
3. Sako, M. et al. Efficient enantioselective synthesis of oxahelicenes using redox/acid cooperative catalysts. *J. Am. Chem. Soc.* **138**, 11481−11484 (2016).
4. Liu, Y.-J., Li, J.-S., Nie, J. & Ma, J.-A. Organocatalytic asymmetric decarboxylative mannich reaction of β-keto acids with cyclic α-ketiminophosphonates: access to quaternary α-aminophosphonates. *Org. Lett.* **20**, 3643−3646 (2018).
5. Zhu, J.-B. & Chen, E. Y.-X. From *meso*-lactide to isotactic polylactide: epimerization by B/N lewis pairs and kinetic resolution by organic catalysts. *J. Am. Chem. Soc.* **137**, 12506−12509 (2015).
6. Frisch, M. J. et al. *Gaussian 16, Revision A. 03*; Gaussian, Inc., Wallingford CT, 2016.
7. Zhao, Y. & Truhlar, D. G. Computational characterization and modeling of buckyball tweezers: density functional study of concave–convex π···π interactions. *Phys. Chem. Chem. Phys.* **10**, 2813-2818 (2008).
8. Marenich, A. V., Cramer, C. J. & Truhlar, D. G. Universal solvation model based on solute electron density and on a continuum model of the solvent defined by the bulk dielectric constant and atomic surface tensions. *J. Phys. Chem. B* **113**, 6378–6396 (2009).
9. Truhlar, D. G., Cramer, C. J., Lewis, A. & Bumpus, J. A. Molecular modeling of environmentally important processes: reduction potentials. *J. Chem. Educ.* **81**, 596–603 (2004).
10. Lu, T. & Chen, Q. Independent gradient model based on Hirshfeld partition: a new method for visual study of interactions in chemical systems. *J. Comput. Chem.* **43**, 539-555 (2022).
11. Lu, T. & Chen, F. Multiwfn: a multifunctional wavefunction analyzer. *J. Comput. Chem.* **33**, 580-592 (2012).
12. Becke, A. D. Density-functional thermochemistry. III. The role of exact exchange. *J. Chem. Phys.* **98**, 5648-5652 (1993).
13. Lee, C., Yang, W. & Parr, R. G. Development of the Colle-Salvetti correlation-energy formula into a functional of the electron density. *Phys. Rev. B* **37**, 785-789 (1988).
14. Gao, Z. et al. Enantioselective synthesis of axially chiral sulfonamides via atroposelective hydroamination of allenes. *ACS Catal.* **11**, 6931−6938 (2021).
